# WNK1 Phosphorylation Sites in TBC1D1 and TBC1D4 Modulate Cell-Surface Abundance of GLUT1

**DOI:** 10.3390/cells15171582

**Published:** 2026-08-31

**Authors:** Andreia F. A. Henriques, Paulo Matos, Ana Sofia Carvalho, Mikel Azkargorta, Felix Elortza, Rune Matthiesen, Peter Jordan

**Affiliations:** 1 Department of Human Genetics, National Health Institute ‘Dr. Ricardo Jorge’, 1649-016 Lisbon, Portugal; andreiaalmeidahenriques@gmail.com (A.F.A.H.); paulo.matos@insa.min-saude.pt (P.M.); 2 BioISI—Instituto de Biossistemas e Ciências Integrativas, Faculdade de Ciências, Universidade de Lisboa, 1749-016 Lisboa, Portugal; 3 Computational and Experimental Biology Group, iNOVA4Health, NOVA Medical School, Faculdade de Ciências Médicas, Universidade NOVA de Lisboa, 1169-056 Lisboa, Portugal; ana.carvalho@nms.unl.pt (A.S.C.); rune.matthiesen@nms.unl.pt (R.M.); 4 Proteomics Platform, CIC bioGUNE, Basque Research and Technology Alliance (BRTA), 48160 Derio, Spain; mazkargorta@cicbiogune.es (M.A.); felortza@cicbiogune.es (F.E.)

**Keywords:** glucose transport, GLUT1, membrane traffic, protein phosphorylation, TBC1D, WNK1, AKT

## Abstract

**Highlights:**

This manuscript is the corrected version of a previous publication that was retracted due to errors detected in the assembly of Figure 3, as documented in the respective publisher’s note at https://doi.org/10.1016/j.abb.2019.108223. Glucose uptake is a key mechanism for cellular growth and survival and involves the regulated insertion of glucose transporter proteins (GLUTs) from storage vesicles into the plasma membrane (PM). Receptor-stimulated signaling pathways control this process, including the insulin receptor that activates protein kinase AKT to phosphorylate TBC1D4, a RAB-GTPase–activating protein involved in membrane traffic regulation.

**What are the main findings?**

- Protein kinase WNK1 modulates cell-surface abundance of the constitutive glucose transporter GLUT1 in human cells and also phosphorylates TBC1D4.
- Mass spectrometry identified serine 704 in TBC1D4, but also serine 565 in its paralogue TBC1D1 as candidate WNK1 phosphorylation sites in vitro, and mutational analysis supports a functional role for these residues in regulating cell-surface GLUT1 abundance.

**What are the implications of the main findings?**

- The results reinforce a regulatory role for WNK1 in glucose metabolism besides AKT.
- Data reveal a better understanding of how GLUT1 trafficking and glucose uptake are regulated, which may have potential relevance for understanding metabolic dysregulation, as observed in many cancer cells or insulin-responsive cell types.

**Abstract:**

This manuscript is the corrected version of a previously published paper. Glucose uptake by mammalian cells is a key mechanism to maintain cell and tissue homeostasis and relies mostly on plasma membrane-localized glucose transporter proteins (GLUTs). Two main cellular mechanisms regulate GLUT proteins in the cell: first, expression of GLUT genes is under dynamic transcriptional control and is used by cancer cells to increase glucose availability. Second, GLUT proteins are regulated by membrane traffic from storage vesicles to the plasma membrane (PM). This latter process is triggered by signaling mechanisms and is well studied in the case of insulin-responsive cells, which activate protein kinase AKT to phosphorylate TBC1D4, a RAB-GTPase–activating protein involved in membrane traffic regulation. Previously, we identified protein kinase WNK1 as another kinase able to phosphorylate TBC1D4 and regulate the surface abundance of the constitutive glucose transporter GLUT1. Here we describe that downregulation of WNK1 through RNA interference in HEK293 cells led to a two-fold decrease in cell-surface GLUT1 abundance, concomitant with a 40% decrease in glucose uptake. By mass spectrometry, we identified serine (S) 704 in TBC1D4 and also S565 in its paralogue TBC1D1 as candidate WNK1 phosphorylation sites. Transfection of the respective phosphomimetic or unphosphorylatable TBC1D mutants into cells revealed that both affected the cell-surface abundance of GLUT1. The results reinforce a regulatory role for WNK1 in GLUT1 trafficking and glucose uptake and may have potential impact for the understanding of metabolic dysregulation, as observed in many cancer cells or insulin-responsive cell types.

## 1. Introduction

The small family of WNK [with no lysine (K)] protein kinases includes four members in the human genome [1], which encode serine/threonine kinases with a specific sequence signature that includes substitution of a highly conserved lysine (hence their name) in the subdomain II of the catalytic domain [2,3] by a cysteine. This conserved lysine residue mediates ATP binding in most kinases, but WNKs use an alternative lysine from subdomain I for this function [4,5].

The four human *WNK* genes (*WNK1*, *WNK2*, *WNK3*, and *WNK4*) are located on different chromosomes [1] and show tissue-specific expression patterns, with *WNK1* being almost ubiquitous. WNK proteins only share about 40% amino acid identity, mostly in their catalytic domains and some coiled-coil domains [6,7,8].

Mutations in the *WNK1* and *WNK4* genes cause the human monogenic disease familial hyperkalemic hypertension (FHHt, also known as pseudohypoaldosteronism type 2 or Gordon syndrome), characterized by excessive renal sodium and potassium retention [6]. WNK1 (similar to WNK3 and WNK4) was identified to act as a multifunctional upstream activator of ion transport proteins in different segments of the nephron, in particular of the sodium–chloride cotransporter NCC [9] and the sodium–potassium–chloride cotransporter NKCC2 [10,11]. Both cotransporters become activated following their phosphorylation at two threonine residues in their amino-terminal cytoplasmic domain, by either the STE20/SPS1-related proline–alanine-rich protein kinase (SPAK) or the oxidative stress-responsive kinase (OSR1). In addition, phosphorylation decreases endocytosis rates, promoting cotransporter retention in the plasma membrane (PM) [12]. The role of WNK1 is to phosphorylate and activate SPAK or OSR1 in hypotonic, low-chloride conditions [9,13,14,15] due to its ability to act as a sensor of low chloride concentration in cells. Chloride usually binds to the WNK1 catalytic domain, leading to inhibition of autophosphorylation and kinase activation [16].

We previously reported that WNK1 is not only linked to ion transport regulation but also has a role in regulating the cell-surface abundance of another PM transporter, the *SLC2A1* gene product glucose transporter GLUT1 [17]. Members of the GLUT family are essential for uptake of extracellular food-derived glucose as the main energy source for most mammalian cell types. Besides an energy-coupled, sodium gradient–driven sugar cotransport in the intestine or kidney, most other tissues rely on facilitative transport by GLUT proteins along a glucose gradient that is established by cells through rapid phosphorylation and glycolysis of glucose [18]. GLUT1 represents the most ubiquitously expressed isoform. Two main cellular mechanisms regulate GLUT1 proteins in the cell: first, expression of the *SLC2A1* gene is under dynamic transcriptional control and is used by cancer cells to increase glucose availability; second, GLUT proteins are regulated by membrane traffic from storage vesicles to the PM. The best-studied example is insulin-stimulated glucose uptake of muscle cells and adipocytes, which occurs through rapid GLUT4 translocation to the PM and proportional increase in glucose uptake [19]. When insulin binds to its receptor on these cells, the phosphatidylinositol-3-kinase (PI3K) is stimulated and triggers activation of protein kinase AKT that then phosphorylates its substrates, the Tre-2-BUB2-CDC16 domain family member (TBC1D4) [20,21,22] or TBC1D1 [21,23,24,25]. These are two paralogous GTPase-activating proteins for RAB-family GTPases, which subsequently mediate translocation of GLUT4 storage vesicles to the PM in oxidative muscle and adipose cells or in glycolytic muscle fibers, respectively [26,27]. Impaired GLUT4 translocation is a hallmark characteristic of insulin resistance leading to diabetes mellitus.

Besides AKT, WNK1 was also identified to phosphorylate TBC1D4 in vitro. WNK1 expression in HEK293 cells promoted binding of TBC1D4 to regulatory 14-3-3 proteins, preventing the inhibitory interaction of TBC1D4 with the small GTPase RAB8A, a positive regulator of exocytic vesicle traffic. In consequence, GLUT1-containing storage vesicles can fuse with the PM and increase the cell-surface abundance of GLUT1 [17]. Similarly, WNK1 was shown to regulate GLUT4 cell-surface abundance in skeletal muscle cells [28].

In addition to WNK1, the serum–glucocorticoid-regulated kinase (SGK1) can also participate in the regulation of TBC1D4. SGK1 was found to phosphorylate TBC1D4 and increase the cell-surface abundance of GLUT1 upon activation by PI3K [29]. Also, SGK1 phosphorylation of TBC1D4 has a role in regulating the endocytosis of the epithelial sodium channel (ENaC) [30]. Thus, WNK1 and SGK1 may represent surrogate pathways to modulate TBC1D4 and glucose uptake in response to insulin.

Here we report the corrected version of our previous results [31] (see publisher’s note at https://doi.org/10.1016/j.abb.2019.108223), showing that both TBC1D4 and TBC1D1 are WNK1 substrates, mapping the respective in vitro phosphorylation sites, and functionally analyzing the effect on cell-surface GLUT1 abundance of two candidate WNK1 phosphorylation sites, TBC1D1-S565 and TBC1D4-S704.

## 2. Materials and Methods

### 2.1. Cell Culture and Transfections

HEK293 (human embryonic kidney 293) cells were obtained from ATCC (CRL-1573) and maintained in Dulbecco’s modified minimal essential medium (DMEM), whereas HT29 colorectal cells (ATCC HTB-38) were maintained in Roswell Park Memorial Institute 1640 Medium (RPMI), both supplemented with 10% (*v*/*v*) of heat inactivated fetal bovine serum (FBS) (all reagents from Gibco, Thermo Fisher Scientific, Waltham, MA, USA). Cells were maintained at 37 °C with 5% CO_2_ and regularly checked for absence of mycoplasma infection by PCR amplification of a 16S ribosomal DNA fragment (primers F: 5′-ACT CCT ACG GGA GGC AGC AGT A and R: 5′-TGC ACC ATC TGT CAC TCT GTT AAC CTC) from lysates of cells harvested from the culture medium.

For ectopic expression of plasmid cDNAs, HEK293 cells were transfected at 80–90% confluence using Metafectene (Biontex, Munich, Germany) according to the manufacturer’s instructions. Transfection efficiencies were found to be around 90%, as determined microscopically using a GFP expression vector. Total amounts of transfected plasmid DNA were kept constant at 6 µg per 60 mm cell culture dish or 2 µg per 35 mm dish and adjusted with empty vector, if required. Cells were analyzed after 20 h for biochemical assays.

For gene-specific transcript silencing, HEK293 cells at 30% confluence were transfected using Metafectene with 200 pmol of small interfering RNA oligonucleotides (siRNAs) per 35 mm dish or 400 pmol per 60 mm dish. Cells transfected with siRNAs were analyzed after 48 h, and the degree of target gene expression depletion was determined in each experiment by Western blot (WB) (see below). All results were confirmed in at least three independent experiments. The siRNA oligos were ordered from Eurofins Genomics (Ebersberg, Germany) with the following sequences: control siLUC: 5′-CGU ACG CGG AAU ACU UCG ATT; siWNK1: 5′-GCA GGA GUG UCU AGU UAU A.

### 2.2. DNA Plasmids and Constructs

All constructs were verified by automated DNA sequencing, and all primer sequences are available in Table S1. TBC1D1 was amplified from pBluescript with the primers KpnTBD1-F and KpnTBD1-R, cloned into the pCR2.1 TOPO-TA vector (Invitrogen), and subcloned as a KpnI fragment into the expression plasmid pcDNA3-Myc. This construct will be referred to as Myc-TBC1D1. To introduce the unphosphorylatable alanine (A) or phosphomimetic aspartate (D) codons, the plasmid was mutated at codon 565 from TCC to GCC or GAC to obtain the constructs Myc-TBC1D1 S565A/D, using the QuickChange Site-Directed Mutagenesis kit (Agilent Technologies, Santa Clara, CA, USA) according to the manufacturer’s instructions.

A previously published construct used in this study was pCR3.1/AS160-2myc [32], here designated as Myc-TBC1D4. To introduce the unphosphorylatable alanine (A) or phosphomimetic aspartate (D) codons, the plasmid was mutated at codon 704 from TCT to GCT or GAT to obtain the constructs Myc-TBC1D4 S704A/D, using site-directed mutagenesis.

### 2.3. In Vitro Protein Kinase Assays

For each condition, 0.5 µg of the recombinant protein kinase (Flag/His-WNK1 (1111-0000-1; N-terminal fragment 171-529), GST-AKT1 (0132-0000-2; full-length protein), GST-SGK1 (0199-0000-2; full-length protein), or GST-SGK3 (0198-0000-2; full-length protein) (all from ProQinase, Freiburg, Germany) were incubated in 20 µL kinase reaction buffer (30 mM Tris-HCl pH 7.5, 10% glycerol, 1 mM DTT, 1 mM Na_3_VO_4_, 10 mM MgCl_2_, 100 µM ATP), mixed with their recombinant substrate (0.5 µg of rMyc-TBC1D1 or rMyc-TBC1D4 (both from Origene Technologies, Rockville, MD, USA) or recombinant kinase-dead OSR1 as a WNK1 positive control [17]), and incubated in the presence of 5 µCi γ^32^P-ATP at 30 °C for 30 min. Finally, 2× SDS sample buffer (100 mM Tris-HCl pH 6.8, 10% glycerol, 4% SDS, 130 mM DTT, 0.5 mg bromophenol blue) was added, and samples were boiled and separated by SDS-PAGE followed by WB. Membranes were exposed to X-ray films and subsequently incubated with the indicated antibodies in order to document that comparable protein quantities were present in each assay.

### 2.4. TBC1D Phosphopeptide Identification

The above protein kinase assays were repeated under non-radioactive conditions using in vitro phosphorylation of 0.5 µg recombinant substrate with 0.5 µg recombinant kinase in duplicate, standard 20 µL-reactions for 1 h at 30 °C. Then, 20 µL iodoacetamide (IAA) for cysteine alkylation was added for 1 h at 21 °C (protected from light), followed by quenching of remaining IAA with 20 µL DTT for 15 min at 21 °C. Duplicates were incubated with either trypsin (cuts after arginine or lysine) or enteropeptidase (cuts only after lysine) and digested overnight at 37 °C. Digested peptides were purified by retention on C12 hydrophobic reverse-phase porous resin filled into previously activated and washed small columns. Resin columns were then shipped for mass spectrometry (MS) to the CIC bioGUNE center in Derio, Spain. Peptide separation was performed on a nanoACQUITY UPLC System (Waters Corporation, Milford, MA, USA) online connected to an LTQ Orbitrap XL mass spectrometer (Thermo Fisher Scientific). An aliquot of each sample was loaded onto a Symmetry 300 C18 UPLC Trap column (180 μm × 20 mm, 5 μm, Waters). MS raw data and a description of their analysis are available in the Supplementary Data.

### 2.5. Biotinylation of Cell-Surface Proteins

HEK293 cells were first transfected with either siWNK1 or siLUC and incubated for 24 h. In a second transfection, the Myc-TBC1D1/4 S-A mutants were transfected into the control (siLUC) dishes, whereas the Myc-TBC1D1/4 S-D mutants were transfected into the siWNK1 dishes. For all conditions, cells were placed on ice, washed three times with ice-cold PBS-CM (PBS pH 8.0 containing 1 mM CaCl_2_ and 1 mM MgCl_2_), and left for 5 min in cold PBS-CM. Cells were then incubated for 45 min with 0.5 mg of EZ-Link Sulfo-NHS-SS-Biotin (Santa Cruz Biotechnology, Heidelberg, Germany) to label all cell-surface proteins. Cells were rinsed twice and left for 15 min on ice with ice-cold Tris-Q [100 mM Tris-HCl pH 8.0, 150 mM NaCl, 0.1 mM CaCl_2_, 1 mM MgCl_2_, 10 mM glycine, 1% (*w*/*v*) BSA] to quench the reaction. Cells were again washed three times with cold PBS-CM and lysed in 250 μL of pull-down buffer (50 mM Tris-HCl pH 7.5, 100 mM NaCl, 10% (*v*/*v*) glycerol, 1% (*v*/*v*) NP40, supplemented with a protease inhibitor cocktail composed of 1 mM PMSF, 1 mM 1,10-phenanthroline, 1 mM EGTA, 10 µM E64, and 10 µg/mL of each aprotinin, leupeptin, and pepstatin A; all from Sigma-Aldrich Quimica, Madrid, Spain). Cell lysates were cleared at 9000× *g* for 5 min at 4 °C. An aliquot of 40 μL, representing total protein levels, was removed and added to 2× SDS modified sample buffer (62.5 mM Tris-HCl pH 6.8, 3% SDS, 10% glycerol, 0.02% bromophenol blue, 196.4 mM DTT), while 200 μL lysate were added to 15 μL of protein G agarose beads (Roche Portugal), rotated for 1 h at 4 °C and centrifuged for 1 min at 3000× *g*. The pre-cleared lysates were recovered, added to 20 μL streptavidin-agarose beads (Sigma-Aldrich), previously incubated for 1 h in 1 mL cold pull-down buffer containing 2% (*w*/*v*) milk, and washed three times in pull-down buffer. Lysate and beads were rotated for 1 h at 4 °C, centrifuged for 1 min at 3000× *g*, and washed five times in cold wash buffer (100 mM Tris-HCl pH 7.5, 300 mM NaCl, 1% (*v*/*v*) Triton X-100). Captured proteins were recovered in 20 μL of 2× SDS-modified sample buffer with 100 mM DTT and analyzed by WB with specific antibodies, as described below.

Protein lysates were separated by 9% (*w*/*v*) SDS-PAGE in 1% (*v*/*v*) glycerol-containing gels run at 4 °C. For detection of specific proteins, SDS-PAGE gels were transferred onto PVDF membranes (Bio-Rad Laboratories, Hercules, FL, USA). WB membranes were blocked in 5% (*w*/*v*) milk powder in TBS with 0.1% (*v*/*v*) Triton X-100, probed using primary antibodies listed below, washed 3×, and incubated with a secondary peroxidase-conjugated antibody. Bands were visualized by chemiluminescence on X-ray films and quantified on digitized images by densitometric analysis with ImageJ 1.53q software (NIH, Bethesda, MD, USA). Primary antibodies used were as follows: mouse anti-PCNA (clone PC10; NA03) from MerckBiosciences (Nottingham, UK); mouse anti-transferrin receptor (clone H68.4; 13-6800), mouse anti-His (Invitrogen 37-2900), and rabbit anti-GST (Chemicon AB3282) from Thermo Fisher Scientific; mouse anti-Tubulin (clone B-5-1-2; T5168) and mouse anti-MYC (clone 9E10; M5546) from Sigma-Aldrich; rabbit anti-GLUT1 (ab652) from Abcam (Cambridge, UK); and sheep anti-WNK1 (S062B) from Dundee University, UK.

### 2.6. Glucose Uptake Assays

HEK293 cells were treated as described, and glucose uptake was measured using the Glucose Uptake-Glo™ Assay (Promega, Madison, WI, USA), according to the manufacturer’s instructions. Briefly, this assay is a luminescence -based method measuring the amount of 2-deoxyglucose (2DG) transported into cells during a period of 10 min. 2DG is transported into cells and phosphorylated to produce 2-deoxyglucose-6-phosphate (2DG6P). The addition of Stop Buffer stops 2DG transport, lyses cells, destroys any NADPH within the cells, and denatures cellular proteins. A fraction of the total cell lysate was collected for Western blotting, while the remaining lysate was mixed with a neutralization buffer before adding the 2DG6P detection reagent, containing glucose-6-phosphate dehydrogenase (G6PDH) that oxidizes 2DG6P to 6-phosphodeoxygluconate (6PDG) and reduces NADP+ to NADPH. The reductase uses the NADPH to convert the proluciferin to luciferin, which is then used by luciferase to produce light measured in the Lucy-2 luminometer (Anthos Mikrosysteme, Friesoythe, Germany). The relative light units (RLUs) were then normalized to the total protein levels based on the PCNA levels in lysates detected by WB. Specifically, PCNA band intensities were first normalized to the mock condition (siLUC) to generate a protein-level correction factor for each sample. Concurrently, the raw luminescence glucose-uptake values were also normalized to their respective mock condition. Finally, the mock-normalized glucose-uptake values were divided by the mock-normalized PCNA ratios, ensuring that the final glucose-uptake readout accurately accounts for any discrepancies in total protein content between samples.

### 2.7. Statistical Analysis

To quantify variations in GLUT1 surface abundance, Western blot band intensities in the biotinylated protein pull-down fractions obtained for GLUT1 were first divided by those obtained for the transferrin receptor (TfR). These ratios were then normalized to their indicated control groups (mock control samples, as indicated, set to value 1.0) and expressed as percentages. For dual group comparisons (control vs. test), statistical outliers were monitored using Grubbs’ test. The Shapiro–Wilk test was then used to assess data normality, followed by a parametric one-sample *t*-test against the hypothetical value of 1.0. ANOVA tests followed by post hoc Tukey’s tests were used when comparing multiple treatments. *p* < 0.05 was accepted as the level of statistical significance. Data shown reflect the mean ± SD from at least three independent experiments (i.e., individual experiments completed on separate days using different cell passages or batches; *n* values are indicated in figure legends). Statistical analyses were performed using GraphPad Prism 5.0 software.

## 3. Results

### 3.1. WNK1 Modulates Glucose Uptake in HEK293 Cells

**Figure 1:**
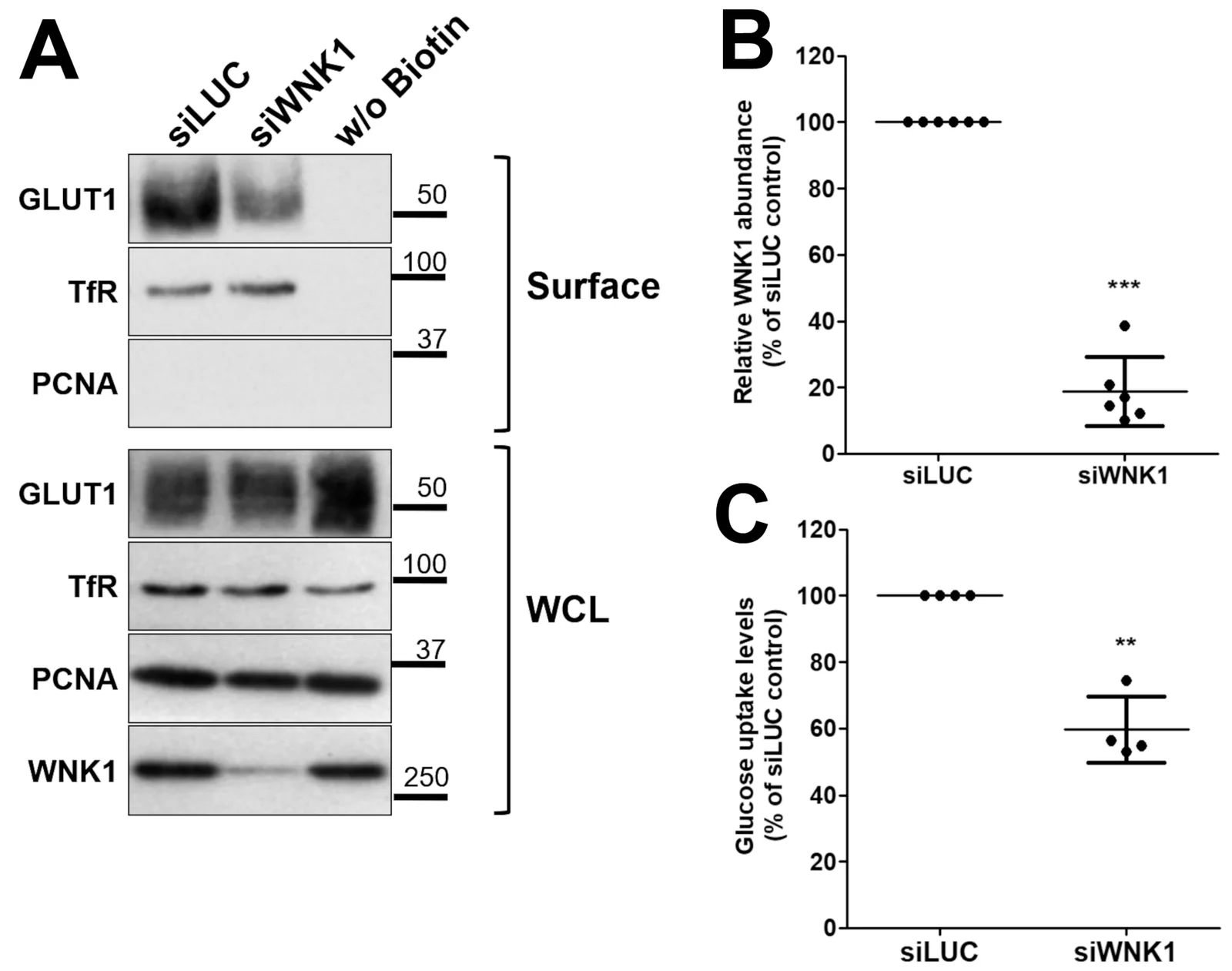
WNK1 modulates glucose uptake in HEK293 cells. Cells were transfected with either siLUC (control) or siWNK1 and incubated for 48 h. Then, cell-surface proteins were biotinylated before cells were lysed, and biotinylated proteins were captured with streptavidin beads, followed by SDS-PAGE and transfer to PVDF blotting membranes. As a further control, cells not incubated with biotin (w/o biotin) were included. (**A**) Detection of the indicated proteins in the whole-cell lysate (WCL) or in the biotinylated protein fraction (Surface). The PCNA protein, as a soluble nuclear and cytoplasmic protein with stable steady-state levels in asynchronous cell cultures, served distinct control purposes in each fraction, documenting either the amount of total protein loaded (WCL) or the absence of contaminating cytosolic proteins (Surface). The transferrin receptor (TfR) served to monitor potential nonspecific effects on endocytosis or recycling (Surface) and further document the amount of loaded protein (Surface and WCL). (**B**) Quantification of WNK1 depletion. Band intensities from the densitometry of membranes shown in (**A**) from independent experiments (*n* = 6) were quantified and graphically displayed. (**C**) Quantification of glucose uptake levels (*n* = 4). The glucose uptake assay was performed using the Glucose Uptake-Glo™ Assay (Promega) following the manufacturer’s instructions, and glucose values were determined by relative light units (RLUs) normalized to total protein values using ImageJ (NIH) for quantification of PCNA Western blot bands [as in (**A**)]. Statistical comparisons were made using a parametric one-sample *t*-test, after confirming data normality via Shapiro–Wilk tests; ** = *p* < 0.01; *** = *p* < 0.001.

Previously, we identified that the expression levels of protein kinase WNK1 regulate cell-surface abundance of the constitutive glucose transporter GLUT1 in HEK293 cells [17]. In pursuit of these studies, we now determined that upon WNK1 depletion, the observed decrease in GLUT1 at the cell surface corresponded to a 40% reduction in cellular glucose uptake (Figure 1C). For this, we transfected HEK293 cells with specific siRNAs to deplete endogenous WNK1 and, 48 h later, measured both their glucose uptake and cell-surface GLUT1 abundance. Under these conditions, the efficiency of WNK1 depletion was around 80%, as confirmed by WB (Figure 1B), and the levels at the PM of the transferrin receptor, which undergoes constitutive endosomal uptake and recycling, were not affected by WNK1 depletion (Figure 1A). A comparable reduction in glucose uptake was also observed upon WNK1 depletion in HT29 colorectal cells (see Supplementary Data, Figure S1). Thus, the expression level of WNK1 contributes to regulating cellular glucose uptake.

### 3.2. Phosphorylation of TBC1D Proteins by Kinases WNK1, AKT and SGK

The mechanism by which WNK1 affected GLUT1 was shown to involve phosphorylation of the RAB-GAP protein TBC1D4 [17]. Phosphorylation of TBC1D4, but also of its paralog TBC1D1, is a key regulatory step in the kinase cascades leading to changes in glucose uptake [21,22]. AKT was the first protein kinase described to phosphorylate the RAB-GAPs TBC1D1 and TBC1D4 in response to insulin stimulation [20,22].

**Figure 2:**
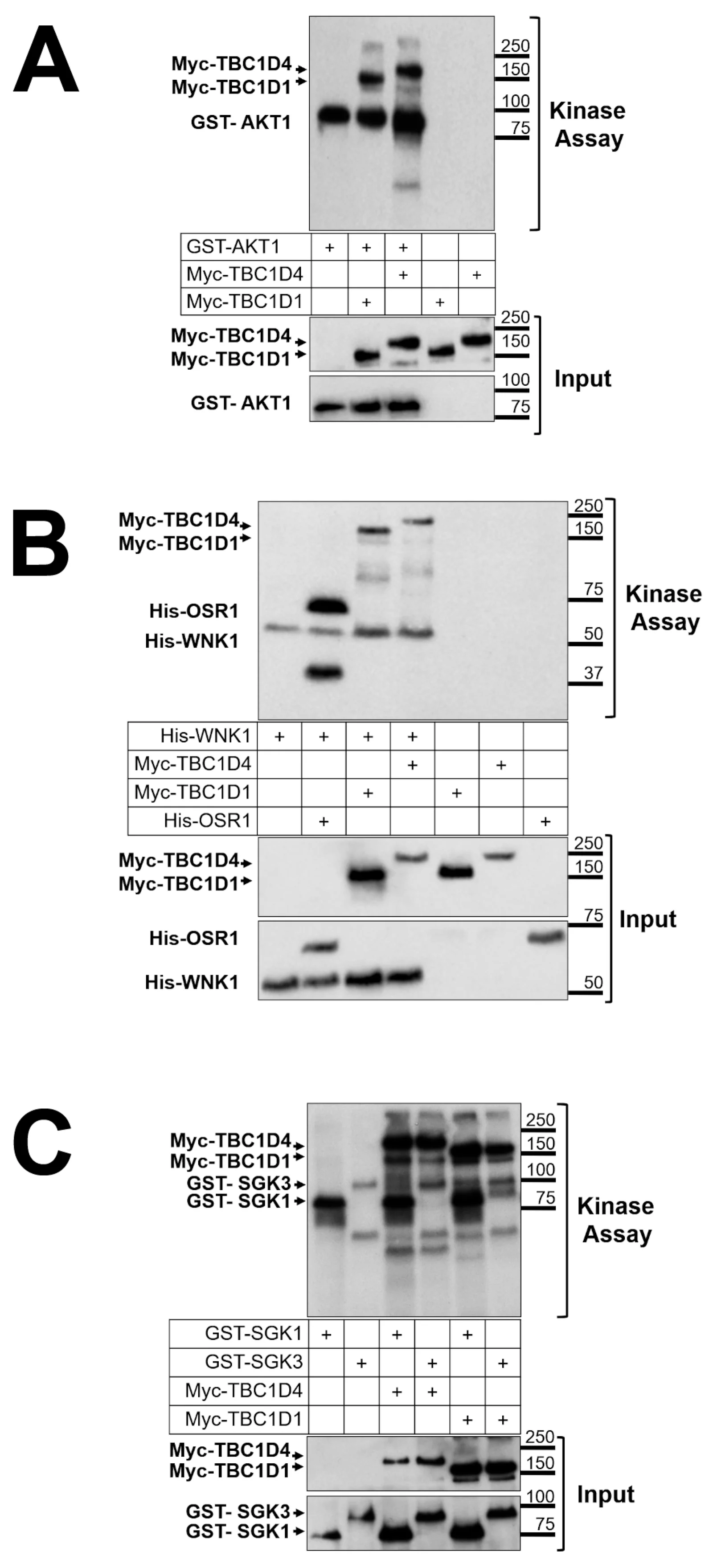
In vitro protein kinase assays to determine the phosphorylation of TBC1D1 and TBC1D4 by the protein kinases (**A**) AKT1, (**B**) WNK1, and (**C**) SGK1 and SGK3. Recombinant tagged kinases were incubated in kinase buffer containing γ^32^P-ATP for 30 min at 30 °C, either alone or with one of the Myc-tagged recombinant TBC1D1 or TBC1D4 substrates. In (**B**), a recombinant kinase-dead mutant of OSR1 (HIS-OSR1) was used as a positive control for WNK1 kinase activity. After incubation with the radio-labeled ATP, the protein samples were separated by SDS-PAGE and transferred to PVDF blotting membranes. First, the incorporated radioactive γ^32^P-phosphate was detected by exposing the membranes for 8 or 24 h to X-ray films (Kinase assay). Subsequently, the quantities of the recombinant proteins present on the radioactive membranes were documented by using anti-GST, anti-Myc, and anti-His antibodies. Note that all studied kinases autophosphorylated, besides phosphorylating the substrate proteins TBC1D1 or TBC1D4.

We therefore compared the sites of phosphorylation in TBC1D4 and TBC1D1 that the kinases AKT and WNK1 would recognize and also included SGK1 for comparison. For this, we performed in vitro protein kinase assays using recombinant RAB-GAP proteins TBC1D1 and TBC1D4 as substrates and incubated them either alone or together with one of the following recombinant protein kinases: AKT1, WNK1, SGK1, and SGK3. As shown in Figure 2A, AKT1 autophosphorylated and, in addition, phosphorylated TBC1D1 and TBC1D4. As expected, both TBC1D proteins were not radio-labeled in the presence of γ^32^P-ATP alone.

For the in vitro WNK1 kinase assay (Figure 2B), recombinant kinase-dead OSR1 (a previously identified physiological WNK1 substrate) served as a positive control [17,33,34], and the negative control, that a kinase-dead WNK1 mutant cannot phosphorylate TBC1D4, was previously shown [17]. His-tagged WNK1 autophosphorylated in the presence of γ^32^P-ATP, but also phosphorylated both TBC1D proteins in vitro. Positive phosphorylation was further observed when GST-SGK1 and GST-SGK3 were used as kinases (Figure 2C), consistent with a previously described role of SGK1 in increasing GLUT1 plasma-membrane abundance in HEK293 cells [29].

### 3.3. Identification of Phosphorylated Sites in TBC1D1 and TBC1D4

Having established the in vitro phosphorylation of both TBC1D1 and TBC1D4, we next determined the specific phosphorylated residues in both RAB-GAPs. Although for AKT, several sites of phosphorylation in both TBC1Ds were already known [20,22,35,36], it was unknown whether WNK1, SGK1, and SGK3 would target the same or unique phosphosites. Phosphorylation sites were determined by MS following kinase assays repeated under non-radioactive conditions, and the results are summarized in Table 1 (raw MS data and their analysis are available as Supplementary Data S2 and Table S2 in Supplementary Data S1, respectively).

The data identified well-known phosphosites for AKT1, such as serine (S) 237 and threonine (T) 596 from TBC1D1 [37], and S588 from TBC1D4 [20], but also that S704 was the most frequently phosphorylated TBC1D4 site by WNK1, SGK1, and SGK3. Importantly, novel TBC1D1 phosphosites were identified: among the four kinases tested, T505 was an SGK1-specific phosphosite, and S565 was a WNK1-specific phosphosite.

### 3.4. Validation of the WNK1-Regulated Phosphosites as Modulators of Cell-Surface Abundance of GLUT1

For further studies of the WNK1-phosphorylated sites TBC1D1-S565 and TBC1D4-S704, their functional relevance for the regulation of cell-surface GLUT1 abundance was validated. First, site-directed mutagenesis was used to introduce the respective phosphomimetic aspartate (D) or unphosphorylatable alanine (A) residues at codons 565 or 704. Then, these Myc-tagged constructs were transfected into HEK293 cells, followed by biotinylation of cell-surface proteins and WB. The unphosphorylatable S565A and S704A mutants were expected to lead to a decrease in the cell-surface abundance of GLUT1, most likely by maintaining the respective TBC1D proteins active. As a positive control and term of comparison, we used WNK1-depleted cells (see Figure 1). By contrast, the phosphomimetic mutants S565D and S704D should promote an increase in cell-surface GLUT1, possibly by inactivating the RAB-GAP activity of the TBC1D proteins. Moreover, the phosphomimetic mutants should also be able to rescue the loss of cell-surface GLUT1 induced by WNK1 depletion alone if they act downstream of WNK1. The transferrin receptor (TfR) was labeled to monitor potential nonspecific effects on endocytosis or recycling.

**Figure 3:**
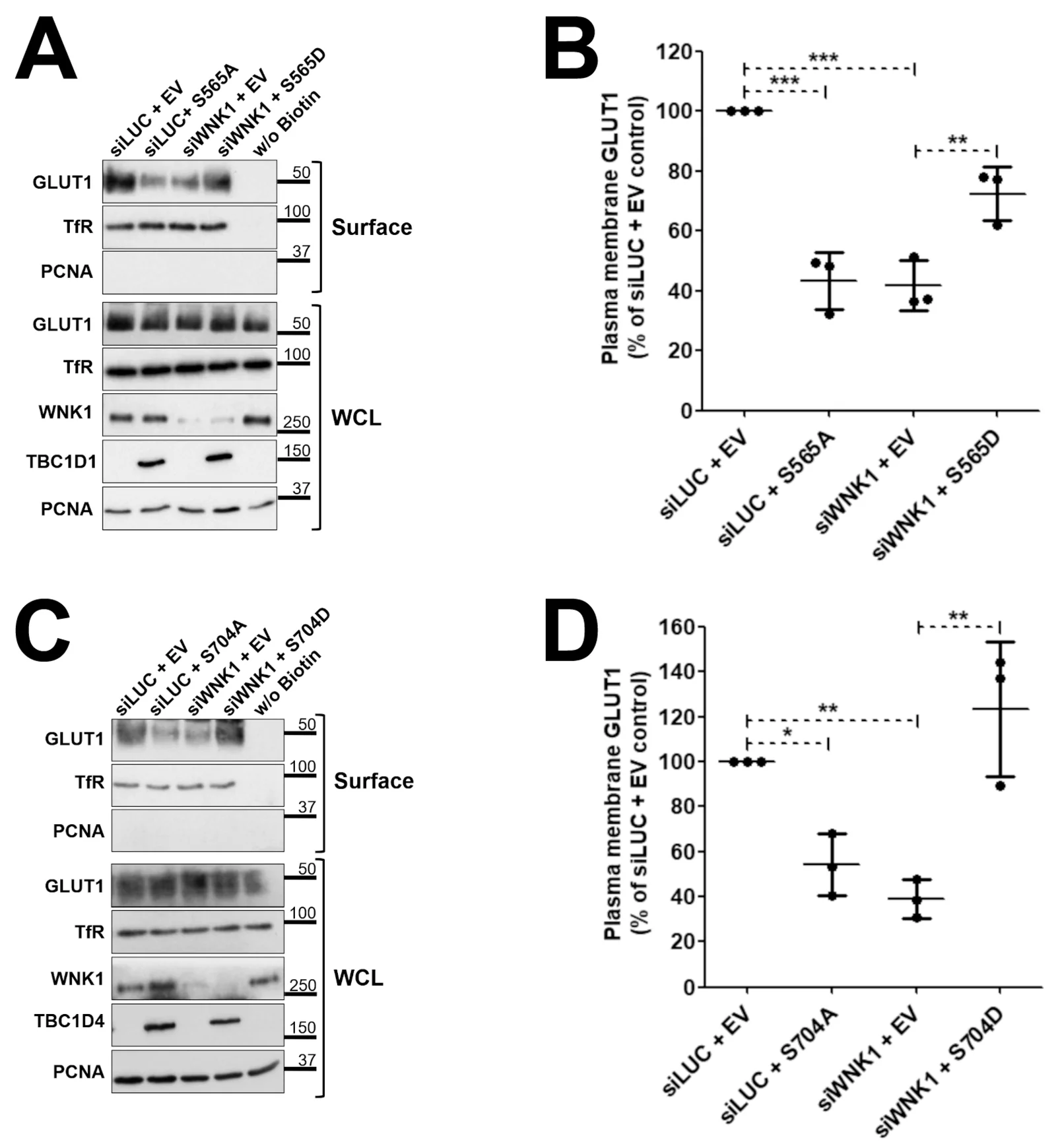
Validation of the effect of the WNK1-targeted phosphosites TBC1D1-S565 (**A**,**B**) and TBC1D4-S704 (**C**,**D**) on GLUT1 cell-surface abundance. HEK293 cells were transfected as indicated either with siLUC and empty-vector (EV) as control, or siLUC and a non-phosphorylatable Myc-tagged TBC1D mutant [(**A**,**B**) TBC1D1-S565A, (**C**,**D**) TBC1D4-S704A)], or siWNK1 and EV as control, or siWNK1 and a phosphomimetic Myc-TBC1D mutant [(**A**,**B**) TBC1D1-S565D, (**C**,**D**) TBC1D4-S704D)]. After 48 h, cell-surface proteins were biotinylated, then cells were lysed and proteins analyzed as described in the legend to Figure 1. (**A**,**C**) Detection of the indicated proteins in the whole-cell lysates (WCL) or in the biotinylated protein fraction (Surface). As a further control, cells were not incubated with biotin (w/o biotin). (**B**,**D**) Corresponding quantification of GLUT1 detection in the biotinylated cell-surface fraction, obtained from three independent experiments and analyzed by ANOVA (*F* = 38.2 and 16.0, respectively, both *p* < 0.001), followed by Tukey’s post-tests. Data are shown as a percentage of siLUC + EV (mock control) and represent means ± SD. Statistical significances are indicated over the comparison bars (|---|) as: * = *p* < 0.05; ** = *p* < 0.01; *** = *p* < 0.001.

As shown in Figure 3A, transfection of HEK293 cells with the non-phosphorylatable TBC1D1-S565A mutant led to a decrease in cell-surface abundance of GLUT1. The quantified magnitude of this effect (Figure 3B) was comparable to the one caused by the depletion of WNK1, used as a positive control. The same decrease in cell-surface GLUT1 was observed in cells transfected with the non-phosphorylatable mutant TBC1D4-S704 (Figure 3C,D).

When cells were then transfected with the phosphomimetic mutants TBC1D1-S565D or TBD1D4-S704D (Figure 3A and Figure 3C, respectively), both were able to counteract the inhibitory effect of siWNK1 and rescue cell-surface GLUT1 abundance, as quantified in Figure 3B,D.

Thus, both phosphorylation sites, TBC1D1-S565 and TBC1D4-S704, were identified as candidate regulatory phosphosites in these RAB-GAP proteins, and their experimental mutation affects cell-surface GLUT1 abundance.

Another phosphorylated TBC1D1 residue identified in Table 1 was T505, which was recognized by SGK1. When the respective unphosphorylatable and phosphomimetic mutants were transfected into HEK293 cells, the analysis of biotinylated cell-surface proteins revealed that neither the TBC1D1-T505A nor the -T505D mutant caused any significant change in the amount of cell-surface abundance of GLUT1 (Figure S2). Thus, in contrast to the two sites TBC1D1-S565 and TBC1D4-S704, the TBC1D1-T505 residue does not seem to play a role in the regulation of GLUT1 abundance at the PM.

## 4. Discussion

The results presented in this work highlight that the regulation of GLUT1, the constitutive glucose transporter expressed in most cell types, through phosphorylation of the RAB-GAP proteins TBC1D4 and TBC1D1 may be more complex than previously reported. We identified not only novel phosphorylation sites in TBC1D4 and TBC1D1, but also two WNK1-phosphorylated sites that are involved in the regulation of GLUT1 plasma membrane abundance.

Although AKT has been predominantly studied as the kinase responsible for the phosphorylation of TBC1D4 and TBC1D1, other kinases, including WNK1, have been shown to contribute to the regulation of glucose uptake [17,28,29,36]. Indeed, phosphorylation at various serine/threonine residues in either TBC1D member is the functionally decisive modification leading to inactivation of their RAB-GAP activity [21,23,36,38]. When their activity is inhibited by phosphorylation, TBC1D members no longer induce GTP hydrolysis by small GTPases such as RAB8A, which allows RAB proteins to adopt their active conformation and promote exocytosis of GLUT-containing storage vesicles. We identified WNK1-regulated phosphorylation sites in each member and demonstrate their involvement in the regulation of cell-surface GLUT1 levels. Consistently, neither the TBC1D1 phosphosite FKLLGS^565^ nor the TBC1D4 motif LHTSFS^704^ contains the consensus AKT recognition motif (R/K)x(R/K)xxS/T. Whereas TBC1D1-S565 was detected only in kinase assays containing WNK1, the phosphorylation of TBC1D4-S704 was detected more frequently in assays containing WNK1, SGK1, or SGK3.

The crucial role of TBC1D4 in GLUT regulation has solid support: experimental suppression of TBC1D4 leads to insulin-independent GLUT4 release to the PM, and mutational inactivation of the GAP domain [39] or of major phosphorylation sites [40] impedes insulin-stimulated GLUT4 translocation. Furthermore, rare human mutations in the *TBC1D4* gene severely affect the carriers’ insulin response [41,42]. TBC1D1 shares high sequence homology and domain structure with TBC1D4 [43], including multiple homologous phosphorylation sites. Both contribute to insulin-dependent glucose uptake, although TBC1D1 was reported to be predominantly expressed in glycolytic muscle [27]. Nevertheless, TBC1D1 has been reported to increase the GLUT1 cell-surface levels in adipocytes [44], and TBC1D4 contributes to the muscle cell response [45].

Although the TBC1D1-S565A mutant transfection caused a significant decrease in cell-surface GLUT1 abundance that was comparable to the effect observed in WNK1-depleted cells, the transfection of the S565D mutant into WNK1-depleted cells was not enough to restore the levels of cell-surface GLUT1 to those observed in control conditions (Figure 3B). Although our results can only provide candidate kinase-associated phosphosites involved in regulating cell-surface GLUT1 abundance, they might indicate that phosphorylation on additional residues might be required for full TBC1D1 inhibition. However, it cannot be excluded that the aspartate in the S565D mutant is an imperfect phosphomimetic. The S-D alteration might also change local protein conformation of a recognition motif for adaptor proteins, causing reduced binding rather than mimicking the presence of a phosphorylated amino acid [46].

By contrast, transfection of mutant TBC1D4 S704D reverted the decrease in cell-surface GLUT1 abundance caused by WNK1 depletion. Phosphorylation at S704 was also reported in skeletal muscle following physical exercise and shown to result from a PI3K-independent mechanism that requires activation of AMPK [47,48]. However, regarding non-exercise-related phosphorylation, little was known about this phosphosite. In the present work, we related the phosphorylation of S704 in TBC1D4 to the protein kinases WNK1, SGK1, and SGK3.

Interestingly, the transfection of the mutants TBC1D1 T505A (non-phosphorylatable mutant) and TBC1D1 T505D (phosphomimetic mutant) did not cause a significant change in the cell-surface GLUT1 abundance (Figure S2). Therefore, we were not able to validate T505 as a determinant phosphosite for GLUT1 localization. Interestingly, proteogenomic studies revealed a TBC1D1 T505 phosphosite enrichment in breast cancer samples [49].

Together, these data provide additional insights into glucose uptake regulation through protein kinase WNK1-mediated phosphorylation of TBC1D1 and TBC1D4, two key regulators of glucose transporters. We showed that WNK1 depletion reduced cellular glucose uptake by 40% (Figure 1), consistent with the fact that several regulatory kinases, including AKT, AMPK, and SGK, have been identified to converge on TBC1D proteins as a common regulatory target.

Dysregulation of glucose uptake in human tissues is of high importance in two major illnesses, type 2 diabetes and cancer. Our findings have potential biomedical implications. First, the biochemical response to insulin is lost in type 2 diabetes, a leading health problem worldwide. Therefore, more detailed knowledge about signaling and trafficking pathways regulating GLUT4 and GLUT1, and how they are coordinated, is essential to understand the pathogenesis of diabetes and to generate new therapeutic options for the restoration of insulin response in the growing population of type 2 diabetes patients. The International Diabetes Federation predicted that the total number of patients with diabetes would rise to over 600 million by the year 2045 [50]. Second, reprogramming energy metabolism is one of the hallmarks of cancer cells in order to fuel their rapid cell growth and division [51]. Otto Warburg observed that cancer cells use the less energy-efficient glycolysis despite the presence of oxygen [52]. In this process, cancer cells compensate for the lower yield in ATP production by upregulating glucose transporters, notably GLUT1, which substantially increases the import of extracellular glucose into the cytoplasm [53]. Overexpression of GLUT1 is correlated with poor survival in most solid tumors [54]. Interestingly, S565 in TBC1D1, a phosphosite described in this work as a regulator of cell-surface GLUT1 abundance, was found to be phosphorylated in samples from non-small-cell lung carcinoma (NSCLC) cell models [55]. Here we showed that WNK1 can phosphorylate TBC1D1 at S565. It is therefore possible that a deregulation of WNK1 expression or activity in cancer cells could promote glucose uptake, besides affecting other signaling pathways, including MAPK cascades, PI3K-AKT, and TGF-β [8,56]. It remains to be explored whether the other three WNK family members also participate in the regulation of cell-surface GLUT1 abundance. WNK-specific inhibitors that are being developed based on the distinct catalytic domain features [57,58] may help to increase the knowledge about WNK signaling in glucose uptake and its dysregulation in disease.

A limitation of our study is that it relies predominantly on experiments performed in HEK293 cells, identifies phosphosites using in vitro kinase reactions not yet validated in vivo, and infers that the phosphorylation state of TBC1D proteins affects RAB-GAP activity.

## 5. Conclusions

In conclusion, we showed that protein kinase WNK1 contributes to glucose uptake regulation in human cells and is capable of phosphorylating in vitro the RAB-GAP proteins TBC1D4 and TBC1D1, which regulate endosomal traffic to the PM of GLUT1, the constitutive glucose transporter expressed in most cell types. We identified phosphorylation sites in TBC1D4 and TBC1D1 recognized by AKT and WNK1 in vitro and demonstrated by mutational analysis a functional role for two candidate WNK1-phosphorylated sites in the regulation of cell-surface GLUT1 abundance. Because dysregulation of glucose uptake in human cells is of high importance in cancer and type 2 diabetes, further research into WNK1 signaling holds promise for potential biomedical implications.

## Figures and Tables

**Table 1:** Major phosphorylated sites identified by mass spectrometry in the indicated human TBC1D family member after in vitro protein kinase reactions with the indicated kinases (S = serine; T = threonine).

human TBC1D1											
AKT1	S237							S585		T596	
WNK1	S237				S565			S585			
SGK1	S237		T505							T596	
SGK3										T596	
human TBC1D4											
AKT1	T568	S588					S725		S751		T752
WNK1						S704	S725				
SGK1	T568	S588		S673		S704					
SGK3	T568	S588				S704	S725				

## Data Availability

Details regarding the mass spectrometry analyses are available in one of the accompanying Supplementary Data files.

## Supplementary Materials

The following supporting information can be downloaded at: https://www.mdpi.com/article/10.3390/cells15171582/s1, Supplementary Data S1 (Figure S1: WNK1 modulates glucose uptake in HT29 cancer cells; Figure S2: Effect of the SGK1-targeted phosphosite TBC1D1-T505 on cell surface GLUT1 abundance, Table S1: Table of the primers used for cloning and site-directed mutagenesis, Table S2: Summary of the mass spectrometry-derived phosphosite analysis after in vitro phosphorylation); Supplementary Data S2 (Mass spectrometry raw data and analysis); Supplementary Data S3 (Raw Western blot images used in the indicated Figures).

## Abbreviations

The following abbreviations are used in this manuscript:

FBS	Fetal bovine serum
GLUT	Glucose transporter
PM	Plasma membrane
siRNA	Small interfering RNA
TBC1D	(Tre-2, BUB2, CDC16) domain family member
WNK	With-no-lysine
wt	Wild type
