# Supplementary material for: WNK1 Phosphorylation Sites in TBC1D1 and TBC1D4 Modulate Cell-Surface Abundance of GLUT1
Source: Cells. 2026 Aug 31;15(17):1582. doi: 10.3390/cells15171582 (PMC13564549; doi:10.3390/cells15171582)
Supplement: Supplementary file 1 [file cells-15-01582-s001.zip › Supplemental Data S1_Figures and Tables.pdf]

Supplementary Data S1

to manuscript

WNK1 phosphorylation sites in TBC1D1 and TBC1D4 modulate cell surface abundance of GLUT1

Andreia F A Henriques, Paulo Matos, Ana Sofia Carvalho, Mikel Azkargorta, Felix Elortza, Rune Matthiesen, Peter Jordan

Fig. S1. WNK1 modulates glucose uptake in HT29 cancer cells. HT29 cells were were transfected with either siLUC (control) or siWNK1 and incubated for 48 h. (**Left**) Quantification of WNK1 depletion in HT29 cells by WB, as described in the legend to Fig. 1. (**Right**) Quantification of glucose uptake levels. The glucose uptake assay was performed using the Glucose Uptake-Glo™ Assay (Promega) following the manufacturer's instructions and as described in the legend to Fig. 1. All data shown represent means ± SEM; *= P< 0.05*, *= P< 0.01.

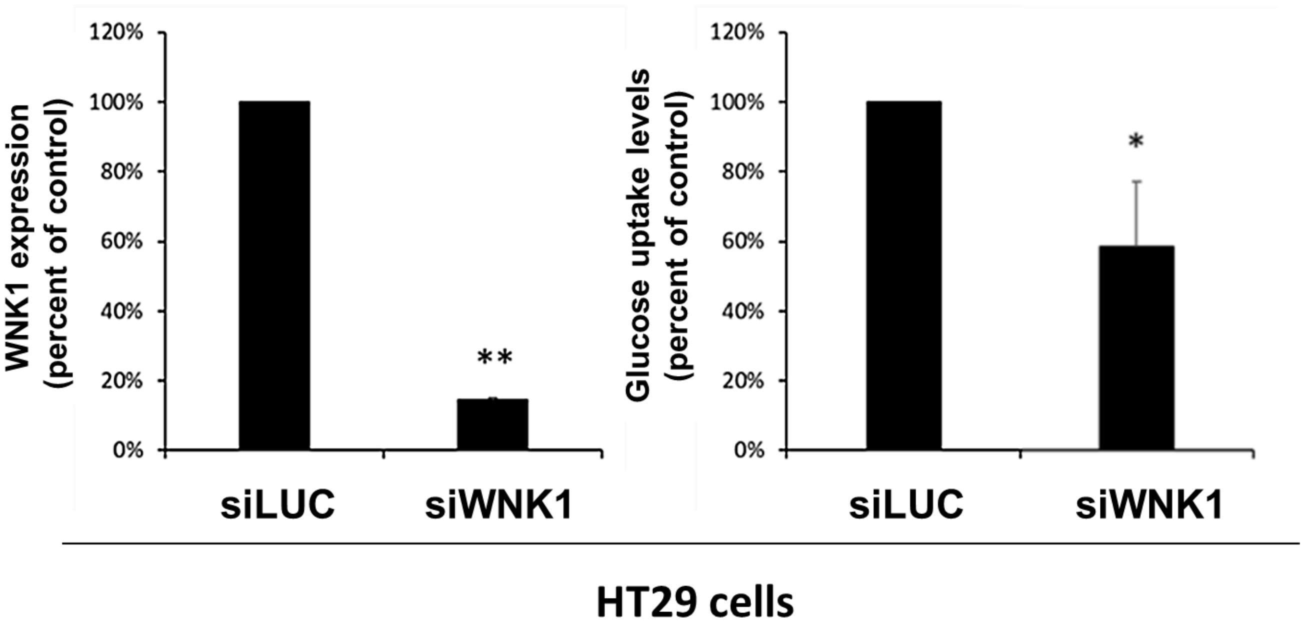

Fig. S2. Effect of the SGK1-targeted phosphosite TBC1D1-T505 on cell surface GLUT1 abundance. HEK293 cells were transfected with either empty vector (EV) or the non-phosphorylatable mutant TBC1D1-T505A, or the phosphomimetic mutant TBC1D1-T505D. After 48 h, cell surface proteins were biotinylated, cells were lysed and proteins analyzed by SDS-PAGE and WB, as described in the legend to Fig. 3. Shown is the corresponding quantification of GLUT1 detection in the biotinylated cell surface fraction as percentage relative to EV, obtained from three independent experiments. Data represent means ± SEM. Note that this phosphosite had no effect on cell surface GLUT1.

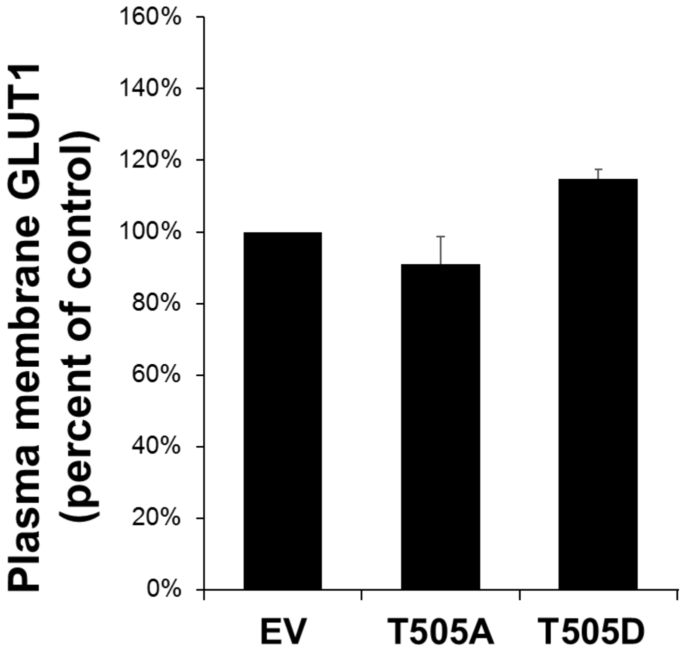

Table S1 Table of the primers used for cloning and site-directed mutagenesis

construct	Primer name	Sequence
pcDNA3-Myc TBC1D1	KpnTBD1-F	5’ GGTACCGAACCAATAACATTCACAGCAA
	KpnTBD1-R	5’ GGTACCTCAGTCGCCCGTGGGCT
MYC-TBC1D1 site-directed mutagenesis	Tbd1 mut T505-A_F	5’ GCAAAGAGATCTTTAGCAGAGTCTTTAG
	Tbd1 mut T505-A_R	5’ CTAAAGACTCTGCTAAAGATCTCTTTGC
	Tbd1 mut T505-D_F	5’ GCAAAGAGATCTTTAGCTGAGTCTTTAG
	Tbd1 mut T505-D_R	5’ CTAAAGACTCAGCTAAAGATCTCTTTGC
	Tbd1 mut S565-A_F	5’ CTTTAAGCTCCTCGGCGCCTCGGAGGAC
	Tbd1 mut S565-A_R	5’ GTCCTCCGAGGCGCCGAGGAGCTTAAAG
	Tbd1 mut S565-D_F	5’ CTTTAAGCTCCTCGGCGACTCGGAGGAC
	Tbd1 mut S565-D_R	5’ GTCCTCCGAGTCGCCGAGGAGCTTAAAG
MYC-TBC1D4 site-directed mutagenesis	Tbd4 mut S704-A_F	5’ GTCTTCACACTTCCTTCGCTGCCCCTTCCTTCAC
	Tbd4 mut S704-A_R	5’ GTGAAGGAAGGGGCAGCGAAGGAAGTGTGAAGAC
	Tbd4 mut S704-D_F	5’ GTCTTCACACTTCCTTCGATGCCCCTTCCTTCACTG
	Tbd4 mut S704-D_R	5’ CAGTGAAGGAAGGGGCATCGAAGGAAGTGTGAAGAC

Table S2 Summary of the mass spectrometry-derived phosphosite analysis after in vitro phosphorylation

(Some sites were searched in two runs: first assuming collision-induced dissociation (CID) and then Electron-transfer dissociation (ETD) ion fragmenting)

Amino acid position	Detection Frequency	Protein	Assay ID
T596	14	TBCD1_HUMAN	Substrate TBC1D1 Kinase AKT1 Replicate #1
S585	2	TBCD1_HUMAN	
S237	2	TBCD1_HUMAN	
S124	5	AKT1_HUMAN	
S146	5	AKT1_HUMAN	
S122	1	AKT1_HUMAN	
T126	1	AKT1_HUMAN	
T596	18	TBCD1_HUMAN	Substrate TBC1D1 Kinase AKT1 Replicate #2
S237	2	TBCD1_HUMAN	
S585	2	TBCD1_HUMAN	
S565	1	TBCD1_HUMAN	
S124	8	AKT1_HUMAN	
T146	6	AKT1_HUMAN	
S122	2	AKT1_HUMAN	
S126	1	AKT1_HUMAN	
CID			Substrate TBC1D1 Kinase WNK1 Replicate #1
S585	10	TBCD1_HUMAN	
S565	8	TBCD1_HUMAN	
S570	4	TBCD1_HUMAN	
S571	2	TBCD1_HUMAN	
ETD			
S585	6	TBCD1_HUMAN	
S570	2	TBCD1_HUMAN	
S571	1	TBCD1_HUMAN	
S585	10	TBCD1_HUMAN	Substrate TBC1D1 Kinase WNK1 Replicate #2
S565	2	TBCD1_HUMAN	
S573	2	TBCD1_HUMAN	
S566	1	TBCD1_HUMAN	
S237	7	TBCD1_HUMAN	Substrate TBC1D1 Kinase WNK1 Replicate #3
S237	10	TBCD1_HUMAN	Substrate TBC1D1 Kinase SGK1 Replicate #1
T596	9	TBCD1_HUMAN	
T505	3	TBCD1_HUMAN	
S503	2	TBCD1_HUMAN	
S598	1	TBCD1_HUMAN	
T596	1	TBCD1_HUMAN	Substrate TBC1D1 Kinase SGK3 Replicate #1

CID			Substrate TBC1D4 Kinase AKT1 Replicate #1
S751	5	TBCD4_HUMAN	
T752	4	TBCD4_HUMAN	
T568	3	TBCD4_HUMAN	
S588	2	TBCD4_HUMAN	
S725	2	TBCD4_HUMAN	
S702	1	TBCD4_HUMAN	
S704	1	TBCD4_HUMAN	
S740	1	TBCD4_HUMAN	
S741	1	TBCD4_HUMAN	
ETD			
T568	3	TBCD4_HUMAN	
S588	2	TBCD4_HUMAN	
S566	1	TBCD4_HUMAN	
S704	1	TBCD4_HUMAN	
S673	1	TBCD4_HUMAN	
S566	1	TBCD4_HUMAN	
T568	3	TBCD4_HUMAN	Substrate TBC1D4 Kinase AKT1 Replicate #2
S725	3	TBCD4_HUMAN	
CID			Substrate TBC1D4 Kinase WNK1 Replicate #1
S725	4	TBCD4_HUMAN	
S704	2	TBCD4_HUMAN	
S106	1	TBCD4_HUMAN	
S30	1	WNK1_HUMAN	
S31	1	WNK1_HUMAN	
S183	1	WNK1_HUMAN	
S185	1	WNK1_HUMAN	
ETD			
S704	1	TBCD4_HUMAN	
S725	1	TBCD4_HUMAN	
S725	3	TBCD4_HUMAN	Substrate TBC1D4 Kinase WNK1 Replicate #2
S704	3	TBCD4_HUMAN	
S106	2	TBCD4_HUMAN	
T105	1	TBCD4_HUMAN	
S716	1	TBCD4_HUMAN	
Y718	1	TBCD4_HUMAN	
S721	1	TBCD4_HUMAN	
S704	4	TBCD4_HUMAN	Substrate TBC1D4 Kinase WNK1 Replicate #3
S725	2	TBCD4_HUMAN	
S672	1	TBCD4_HUMAN	
S673	1	TBCD4_HUMAN	
T701	1	TBCD4_HUMAN	
S702	1	TBCD4_HUMAN	
S183/S185/S189	3	WNK1_HUMAN	
S443	2	WNK1_HUMAN	
S588	13	TBCD4_HUMAN	Substrate TBC1D4 Kinase SGK1 Replicate #1
T568	8	TBCD4_HUMAN	
S704	6	TBCD4_HUMAN	

S673	5	TBCD4_HUMAN	
S106	2	TBCD4_HUMAN	
S566	2	TBCD4_HUMAN	
S672	2	TBCD4_HUMAN	
T749	1	TBCD4_HUMAN	
S750	1	TBCD4_HUMAN	
S751	1	TBCD4_HUMAN	
S588	7	TBCD4_HUMAN	Substrate TBC1D1 Kinase SGK3 Replicate #1
S704	4	TBCD4_HUMAN	
T568	2	TBCD4_HUMAN	
S725	2	TBCD4_HUMAN	
S106	1	TBCD4_HUMAN	
S566	1	TBCD4_HUMAN	
T701	1	TBCD4_HUMAN	
S702	1	TBCD4_HUMAN	
S15	1	SGK3_HUMAN	
S20	1	SGK3_HUMAN	
