# Supplementary material for: WNK1 Phosphorylation Sites in TBC1D1 and TBC1D4 Modulate Cell-Surface Abundance of GLUT1
Source: Cells. 2026 Aug 31;15(17):1582. doi: 10.3390/cells15171582 (PMC13564549; doi:10.3390/cells15171582)

Mass spectrometry raw data and analysis

Supplementary Material to manuscript

WNK1 phosphorylation sites in TBC1D1 and TBC1D4 modulate cell surface expression of GLUT1

Andreia F A Henriques, Paulo Matos, Ana Sofia Carvalho, Mikel Azkargorta, Felix Elortza, Rune Matthiesen, Peter Jordan

MS data analysis

LC-MS raw data files were converted to mgf files using RawConverter [L. He, J.K. Diedrich, Y.Y. Chu, J.R. Yates, Extracting Accurate Precursor Information for Tandem Mass Spectra by RawConverter. Anal Chem. 87 (2015) 11361-7. doi: 10.1021/acs.analchem.5b02721.]. The mgf files were then searched using X!TANDEM against human proteins from UniProt 3AUP000005640 [R. Craig, R.C. Beavis, TANDEM: matching proteins with mass spectra, Bioinformatics. 20 (2004) 1466-7. doi: 10.1093/bioinformatics/bth092]. The sequences in 3AUP000005640 were reversed by maintaining lysine and arginine in place as previously described [R. Matthiesen, G. Prieto, H.C. Beck, Comparing Peptide Spectra Matches Across Search Engines, in: Matthiesen R. (eds) Mass Spectrometry Data Analysis in Proteomics. Methods in Molecular Biology 2051 (2020) 133-143. doi: 10.1007/978-1-4939-9744-2_5.133-143]. The settings used were 10 ppm for parent ions, 0.5 Da for fragment ions, 2 missed cleavages were allowed and cleavage specificity was set to trypsin. Fix modification was set to iodoacetamide and variable modifications to N-terminal protein acetylation, oxidation of methionine and phosphorylation of S, T and Y. Peptides and proteins were filtered on a 1% FDR value. The data were searched in two runs: first assuming collision-induced dissociation (CID) and then Electron-transfer dissociation (ETD) ion fragmenting. The raw MSMS spectra were annotated for all the phospho peptides identified with a 1% FDR cut off. Next ion counts were extracted for all identified peptides using match between runs. The samples were run in four batches and only match between runs from the same batch were allowed. The ion counts were normalized to the average ion count of all unmodified peptides from a given protein. Several MSMS spectra could be associated to the same quantitative LC-MS peak with different position of phosphorylation modifications based on the X!Tandem search result. Therefore, an additional analysis matching all identified phospho peptides back to the raw MSMS data were performed. The scoring of the phosho-peptides with all possible combination of phosphorylation sites against the raw MSMS spectra were performed based on the hypergeometric probability function considering neutral losses of phospho, b- and y-ions for CID and c- and z-ions for ETD data (table S2). The matching of the phospho peptides against the raw data gave less redundancy in terms of the number of possible phosphorylation sites. However, some LC-MS peaks were still associated with MSMS spectra matching the peptide with different phosphorylation sites.

Raw data have been submitted to the Pride archive (Vizcaíno et al. (2016). Nucleic Acids Res 44: D447-D456; doi: 10.1093/nar/gkv1145) under accession number PXD016218. Below follow the annotated raw MSMS spectra for all the phospho-peptides identified with a 1% FDR cut off. A summary of the major phosphorylation sites identified in each sample is provided in Table S2.

annotated raw MSMS spectra for assay:

Protein kinase: AKT1

Substrate: TBC1D1

sample name: TBCD1_AKT1_RS1

LLGSSEDLSSDSESHLPEEPAPLSPQQAFR 24−S_Phospho z=+3 Raw scan=3727
TBCD1_HUMAN S570; ; Q= 0 ; score= 113.3 ; E= 1e−15 m/z=1101.8499

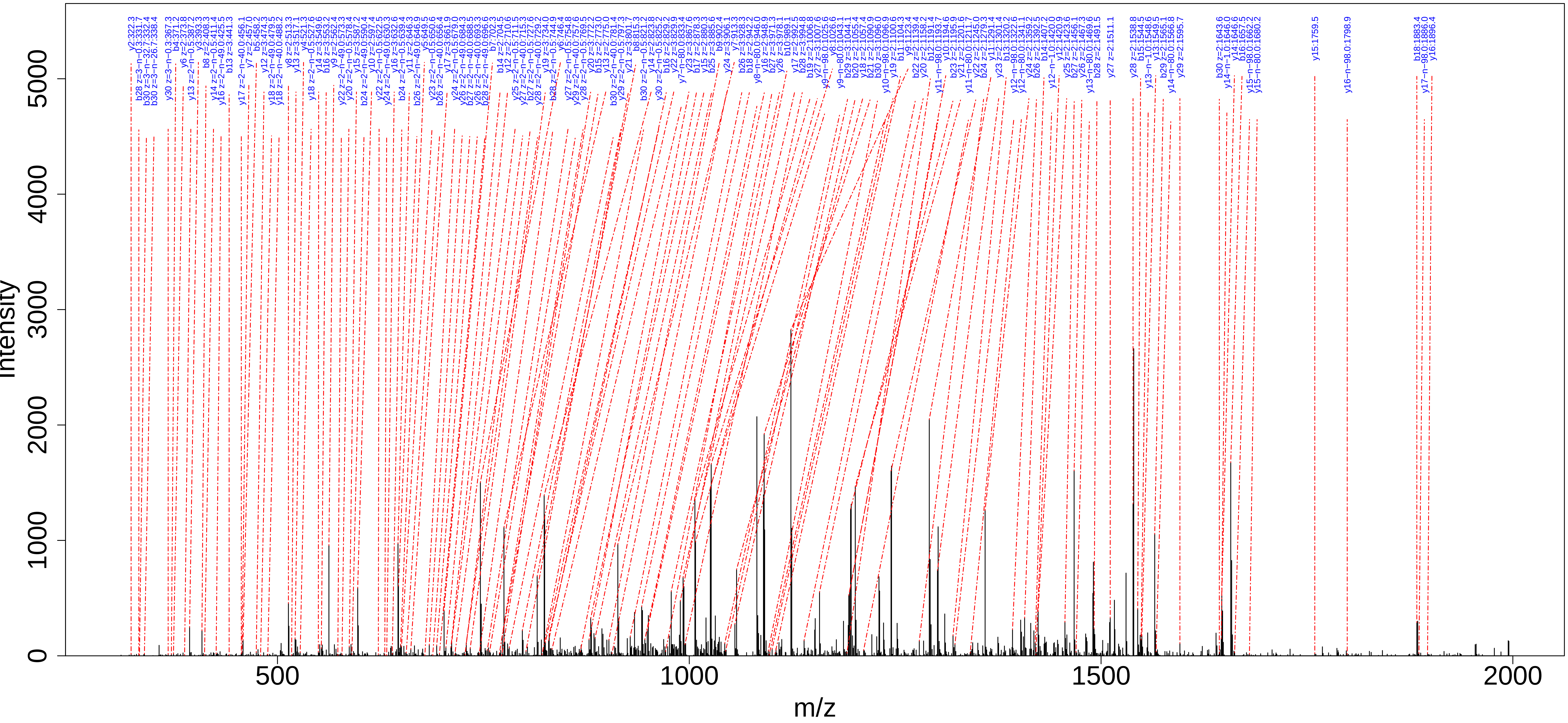

LLGSSEDLSSDSESHLPEEPAPLSPQQAFR 24−S_Phospho z=+3 Raw scan=3719
TBCD1_HUMAN S585; ; Q= 0 ; score= 107.2 ; E= 1e−15 m/z=1101.8469

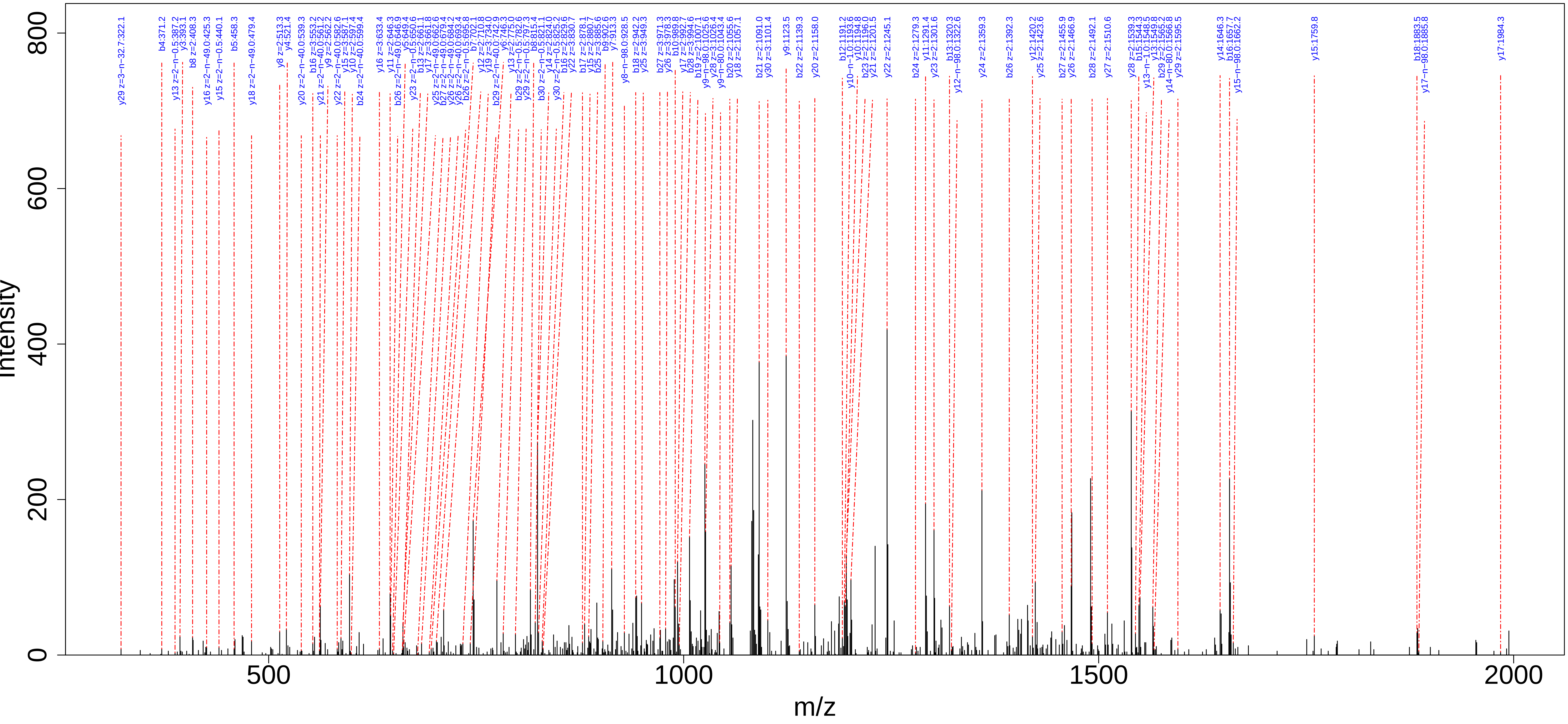

ANTLSHFPIECQEPPQPAR 3−T_Phospho z=+3 Raw scan=3116
TBCD1_HUMAN T596; ; Q= 0 ; score= 82.5 ; E= 5.5e−13 m/z=758.0153

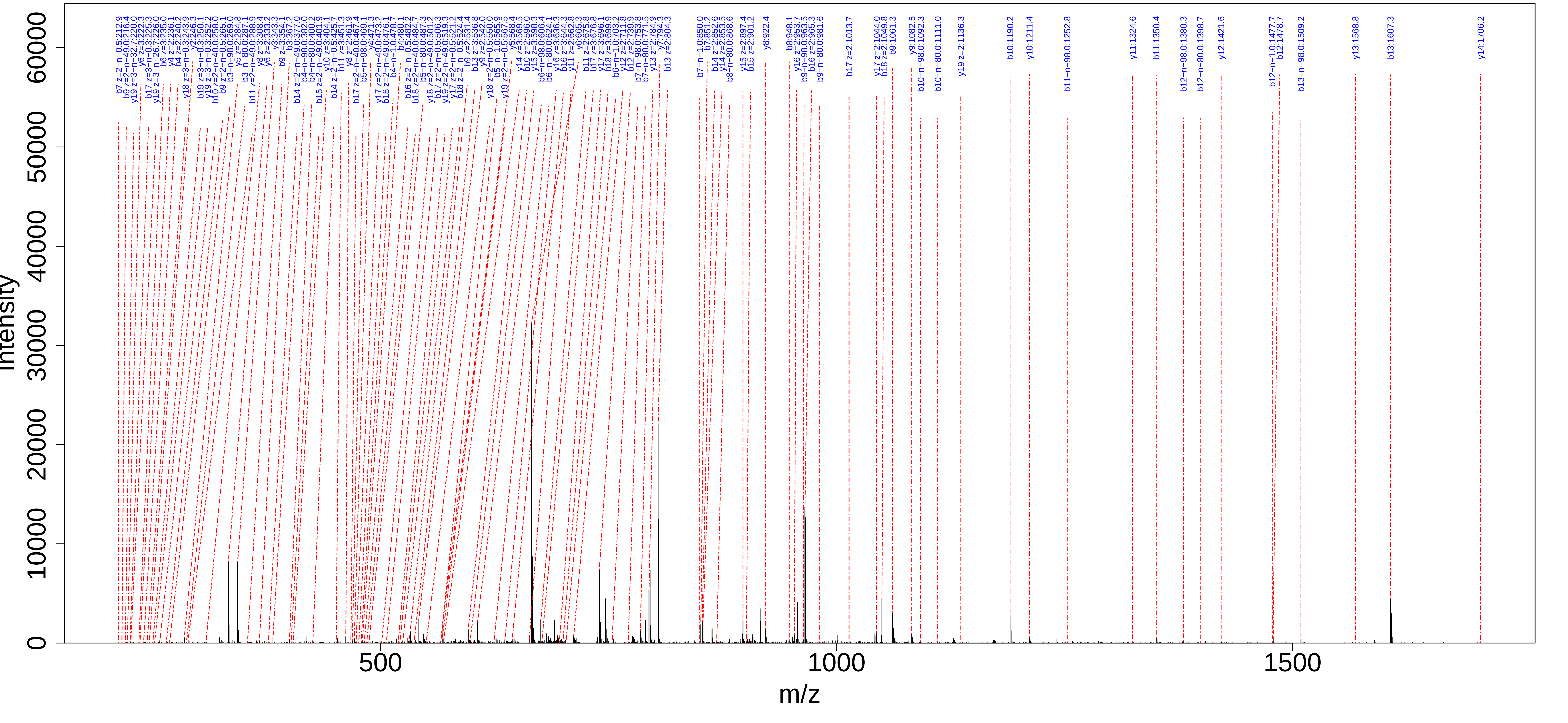

RANTLSHFPIECQEPPQPAR 4−T_Phospho z=+3 Raw scan=2806
TBCD1_HUMAN T596; ; Q= 0 ; score= 71.9 ; E= 1.4e−10 m/z=810.0482

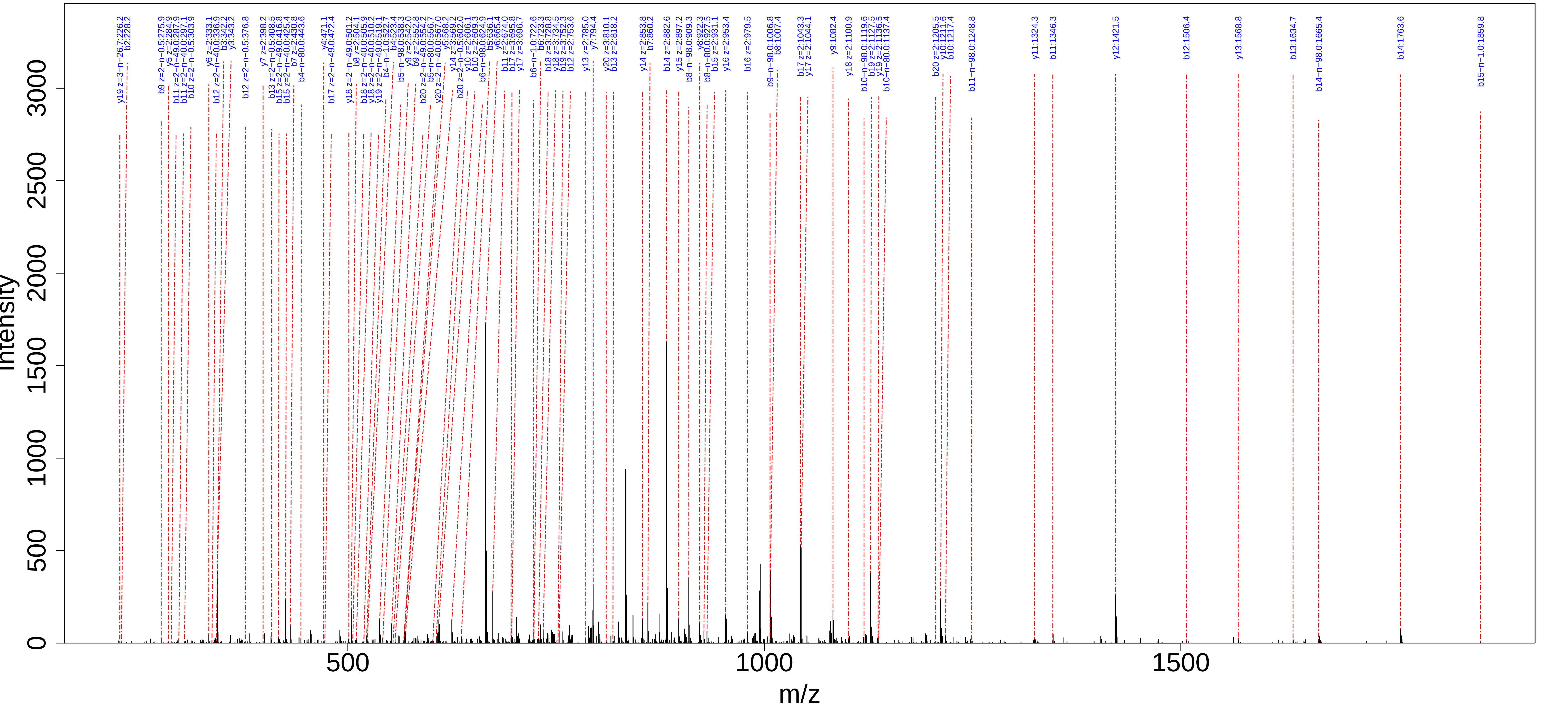

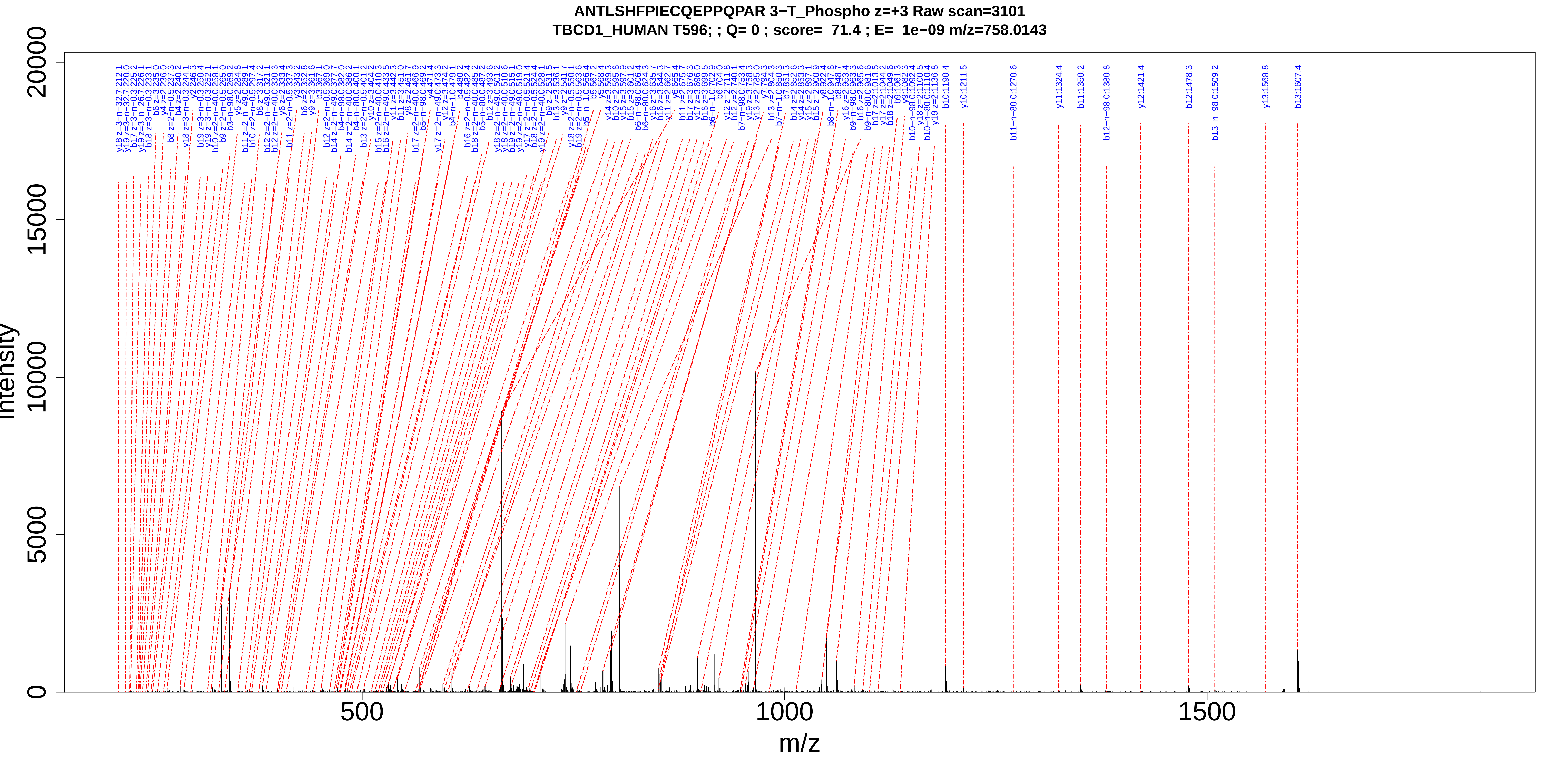
ANTLSHFPIECQEPPQPAR 3−T_Phospho z=+3 Raw scan=3101
TBCD1_HUMAN T596; ; Q= 0 ; score= 71.4 ; E= 1e−09 m/z=758.0143
Intensity
m/z
0
5000
10000
15000
20000
500
1000
1500

ANTLSHFPIECQEPPQPAR 3−T_Phospho z=+3 Raw scan=3108
TBCD1_HUMAN T596; ; Q= 0 ; score= 71.3 ; E= 1.1e−10 m/z=758.0166

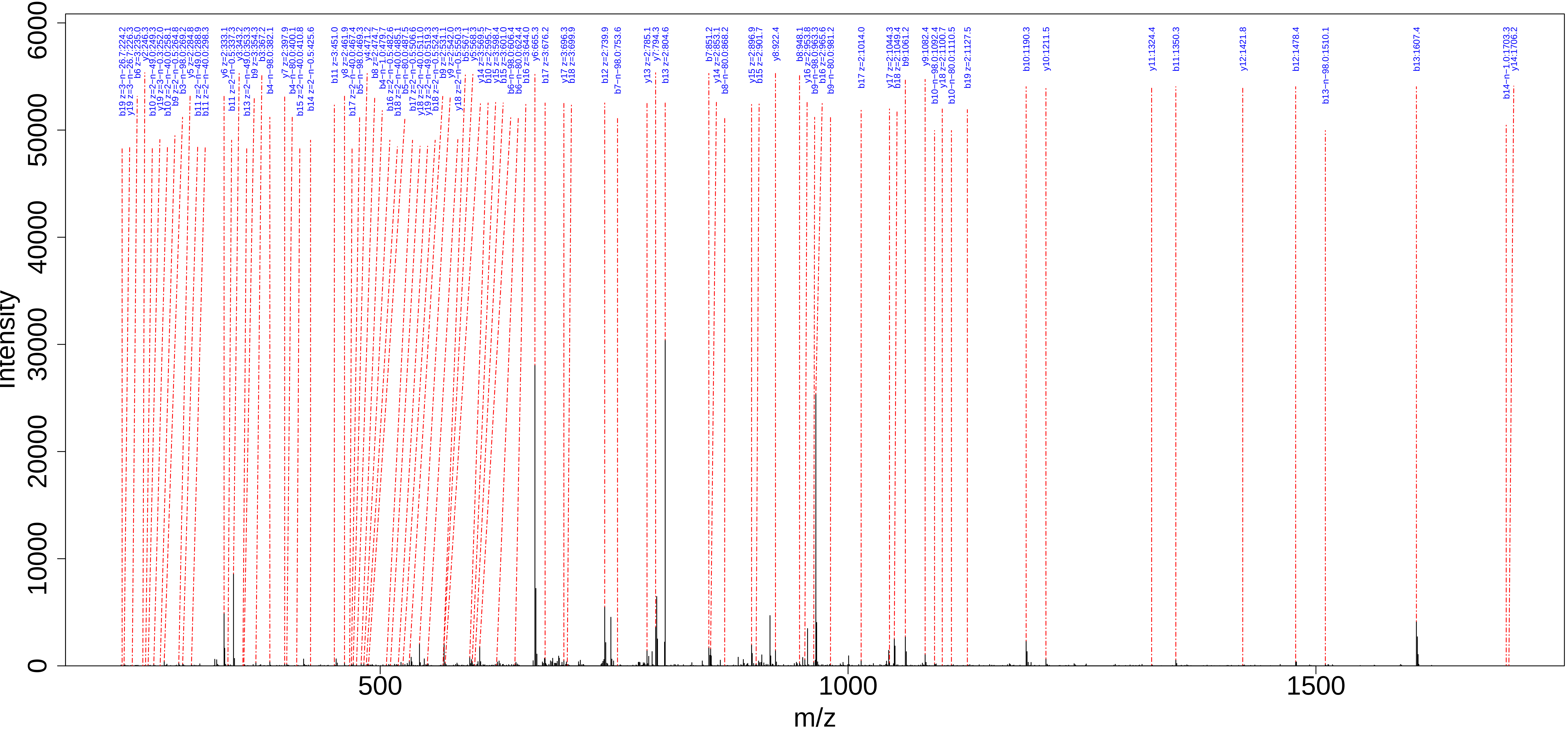

ANTLSHFPIECQEPPQPAR 3−T_Phospho z=+3 Raw scan=3129
TBCD1_HUMAN T596; ; Q= 0 ; score= 69.4 ; E= 8e−08 m/z=758.0146

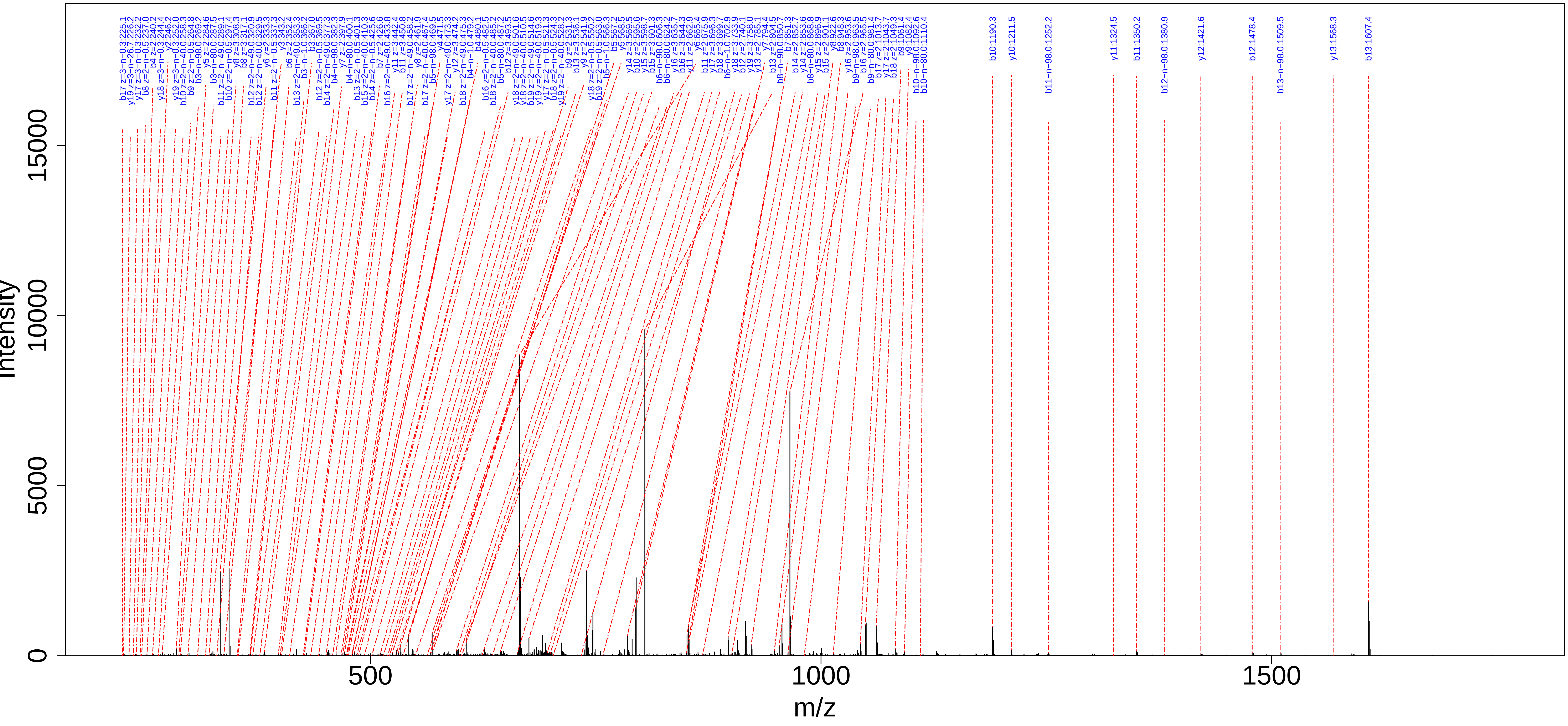

ANTLSHFPIECQEPPQPAR 3−T_Phospho z=+3 Raw scan=3136
TBCD1_HUMAN T596; ; Q= 0 ; score= 68.2 ; E= 2.1e−09 m/z=758.0157

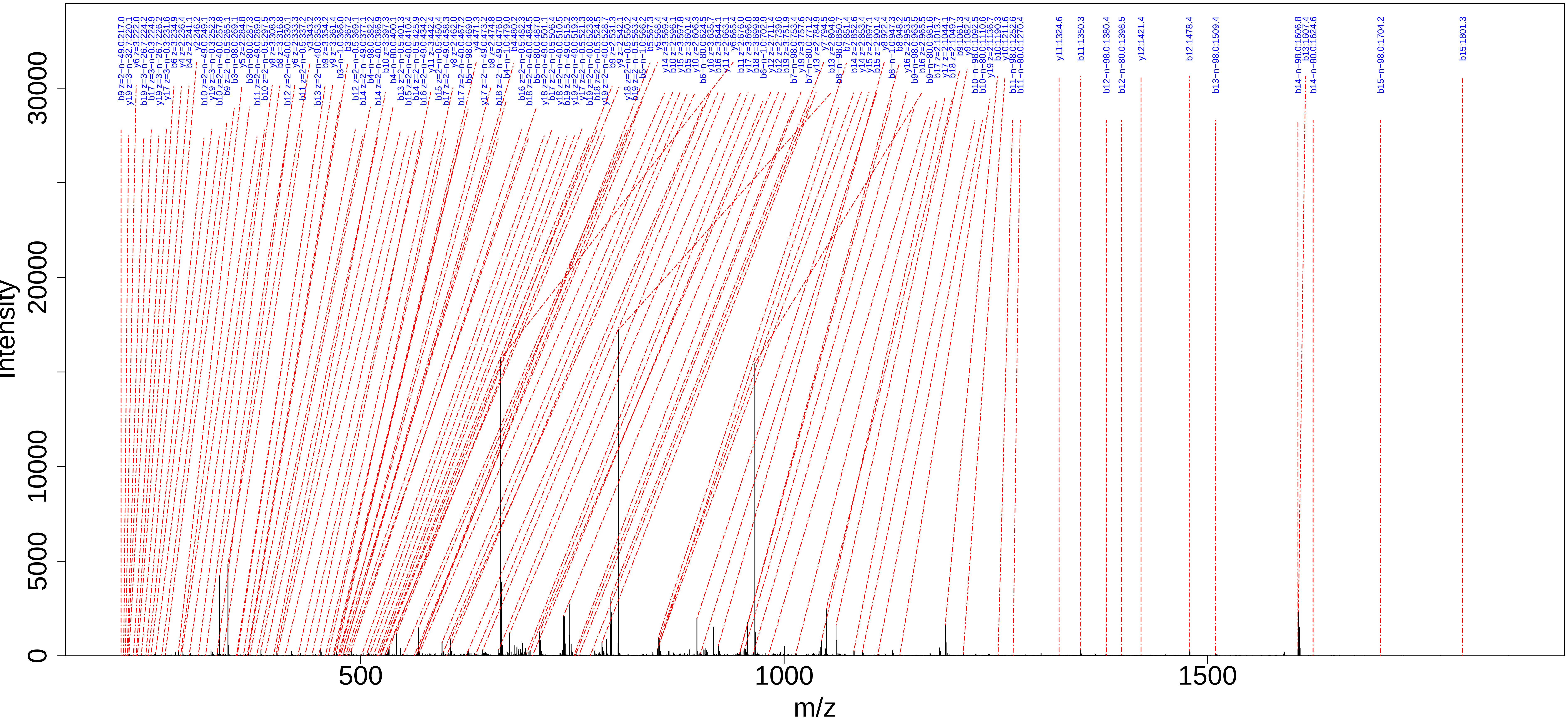

RANTLSHFPIECQEPPQPAR 4−T_Phospho z=+3 Raw scan=2773
TBCD1_HUMAN T596; ; Q= 0 ; score= 68.1 ; E= 1.2e−09 m/z=810.0489

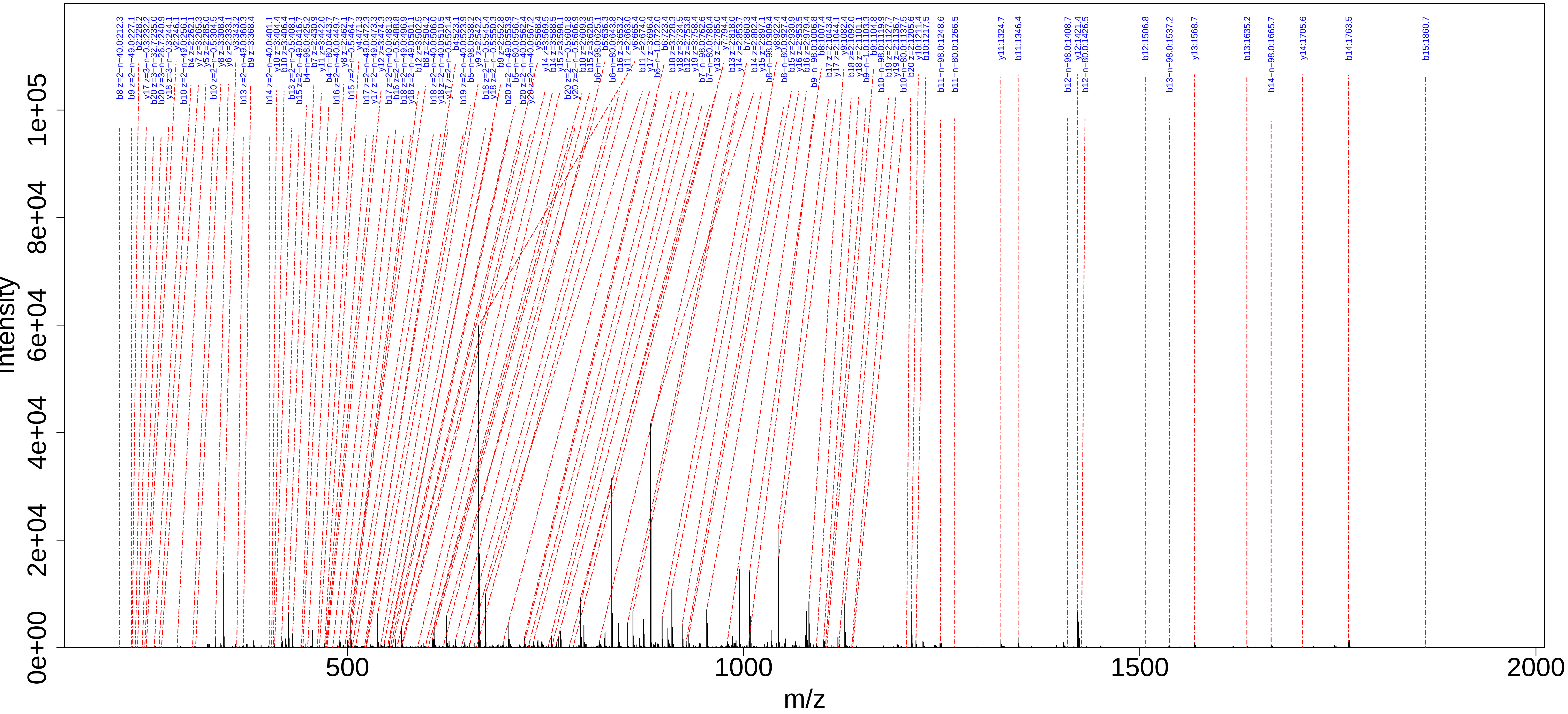

ANTLSHFPIECQEPPQPAR 3−T_Phospho z=+3 Raw scan=3122
TBCD1_HUMAN T596; ; Q= 0 ; score= 67.2 ; E= 2.4e−06 m/z=758.0152

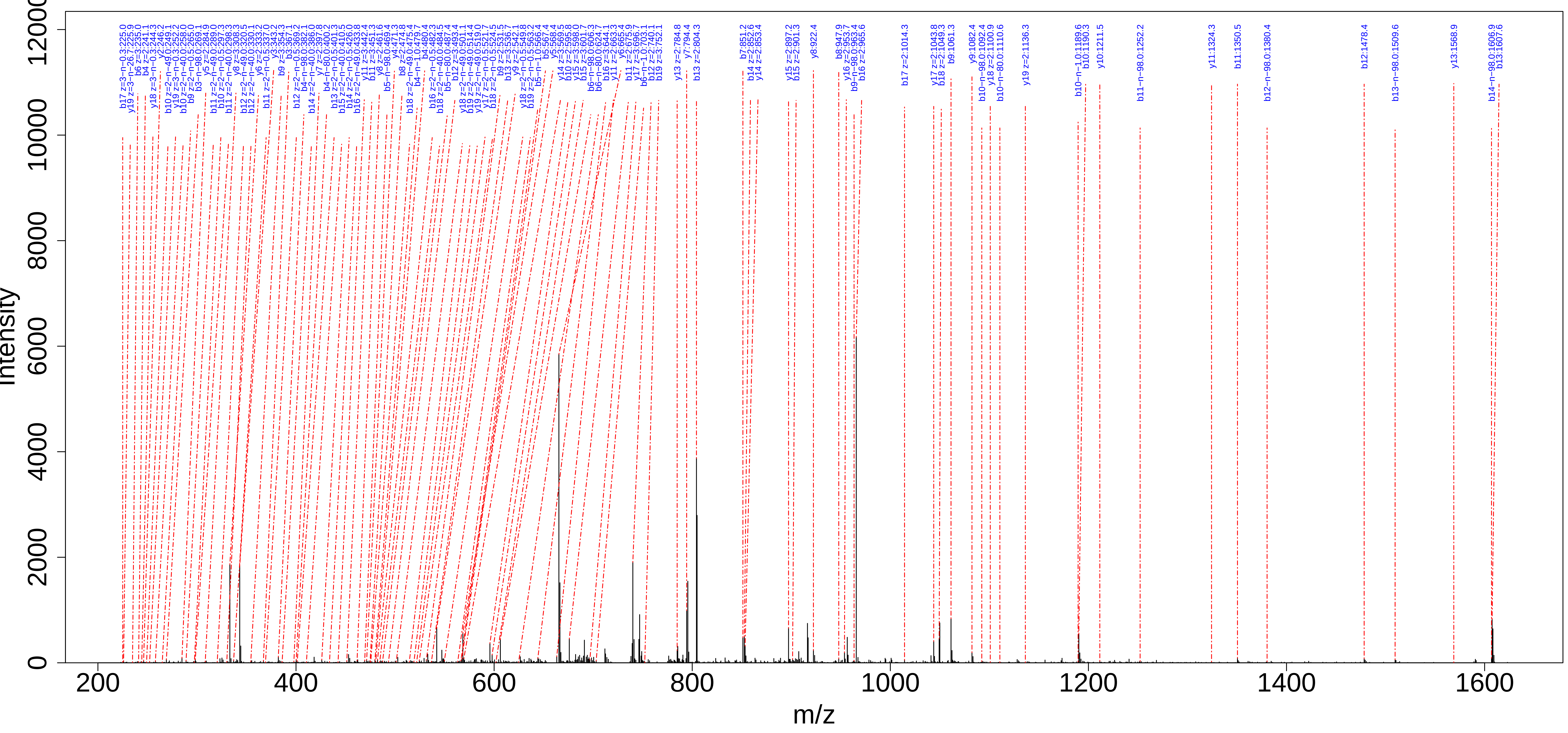

RANTLSHFPIECQEPPQPAR 4−T_Phospho z=+3 Raw scan=2798
TBCD1_HUMAN T596; ; Q= 0 ; score= 63.9 ; E= 2.5e−08 m/z=810.048

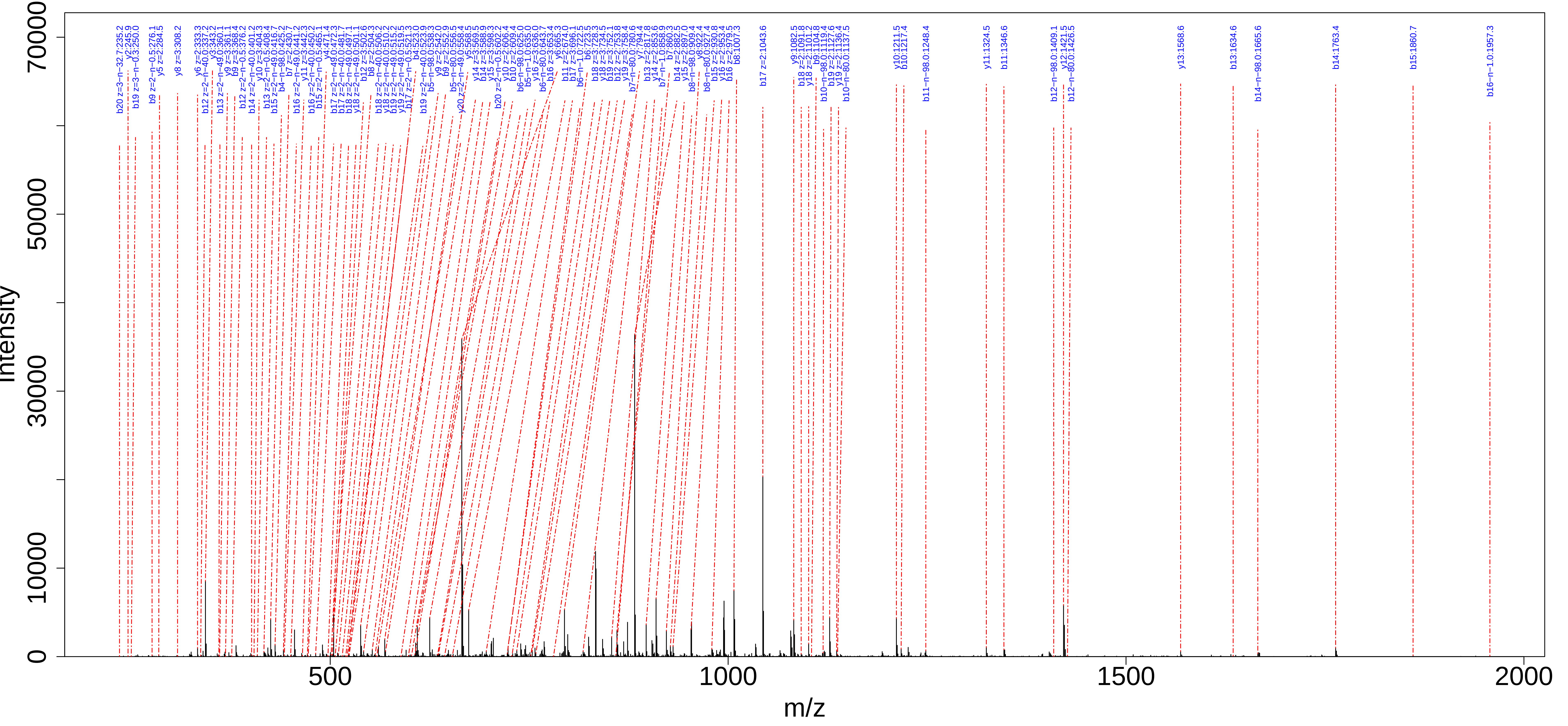

SGSPSDNSGAEEMEVSLAKPK 3−S_Phospho z=+2 Raw scan=2732
AKT1_HUMAN S126; ; Q= 0 ; score= 63.4 ; E= 6e−10 m/z=1100.4834

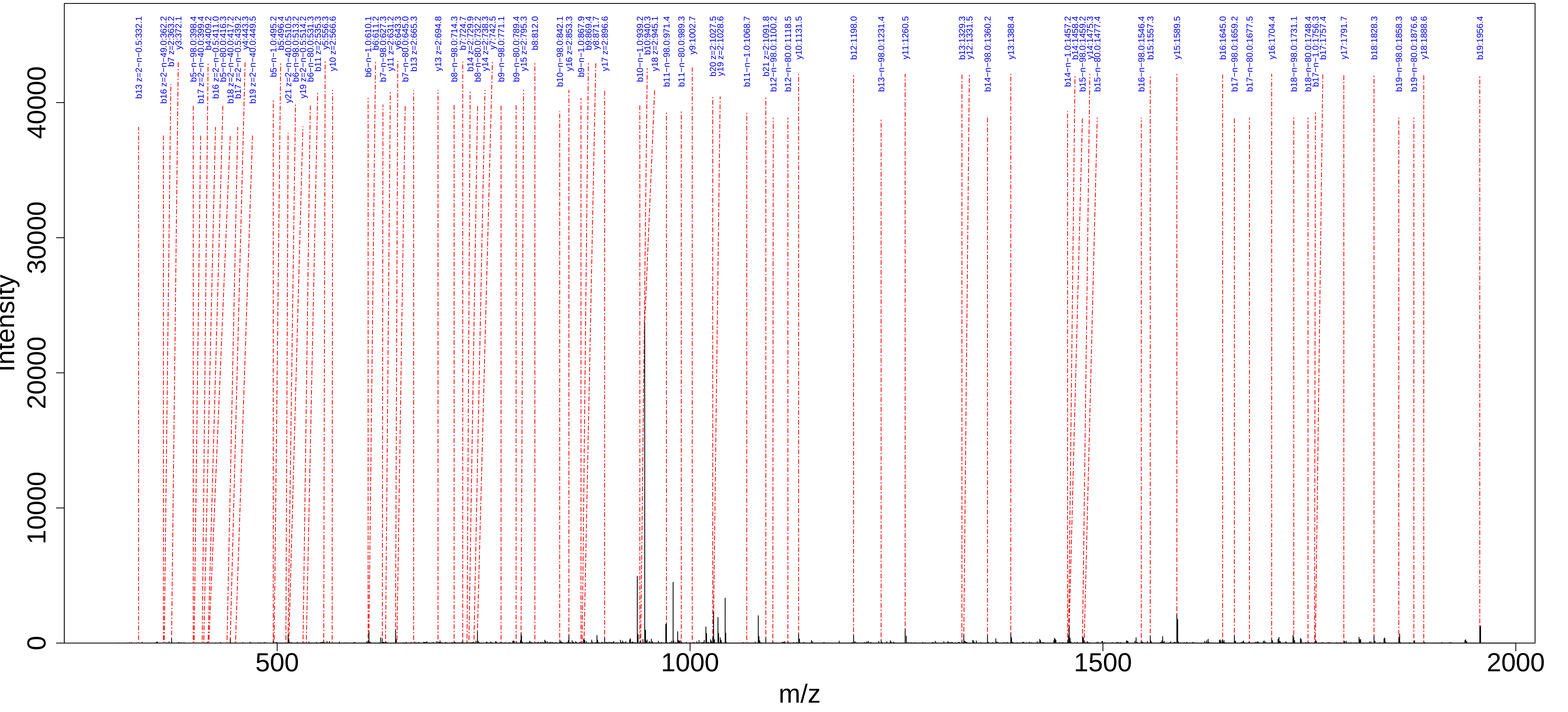

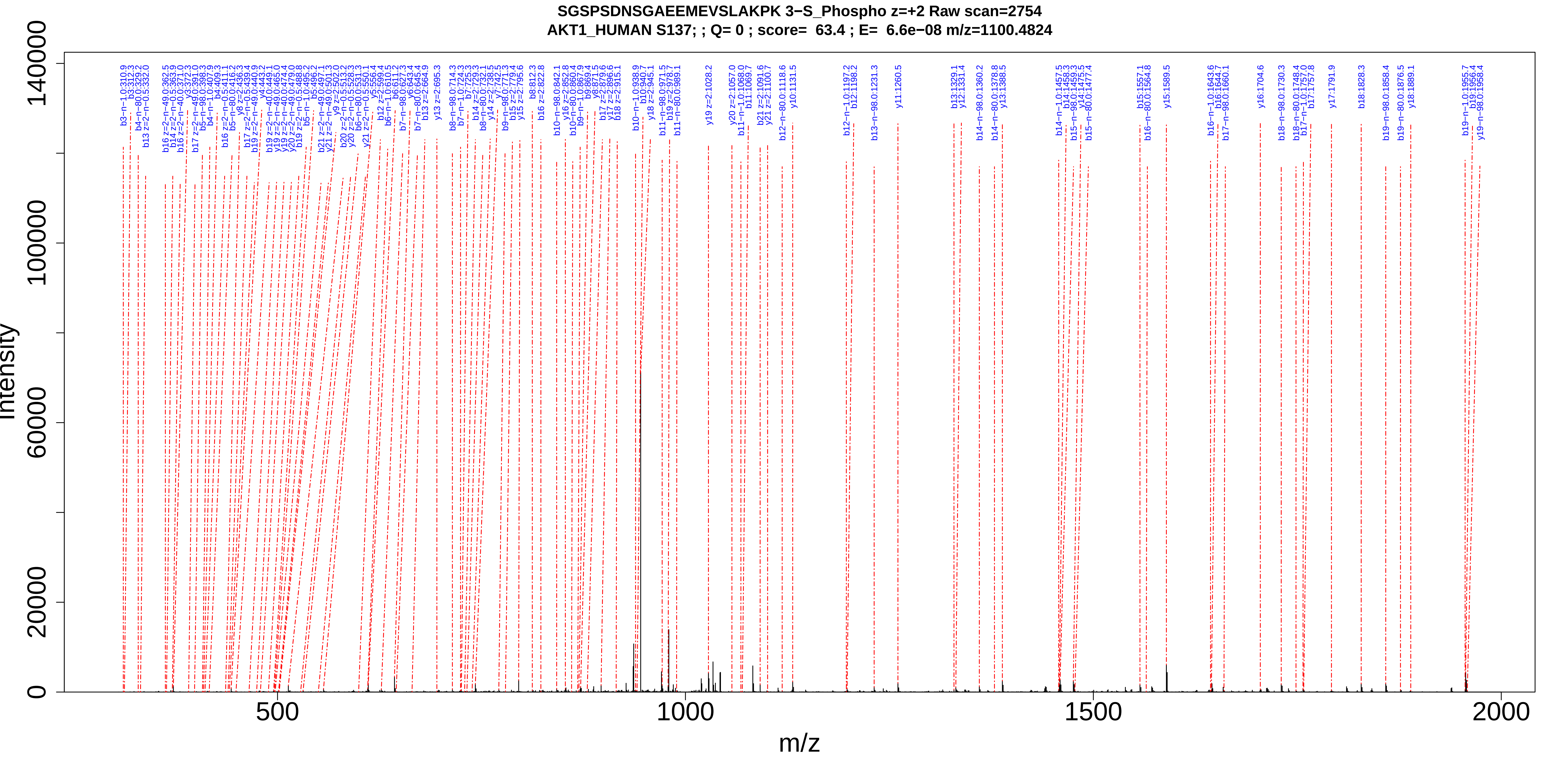
SGSPSDNSGAEEMEVSLAKPK 3−S_Phospho z=+2 Raw scan=2754
AKT1_HUMAN S137; ; Q= 0 ; score= 63.4 ; E= 6.6e−08 m/z=1100.4824
Intensity
m/z
0
20000
60000
100000
140000
500
1000
1500
2000

SGSPSDNSGAEEMEVSLAKPK 3−S_Phospho z=+2 Raw scan=2724
AKT1_HUMAN S137; ; Q= 0 ; score= 63.4 ; E= 2.8e−08 m/z=1100.4817

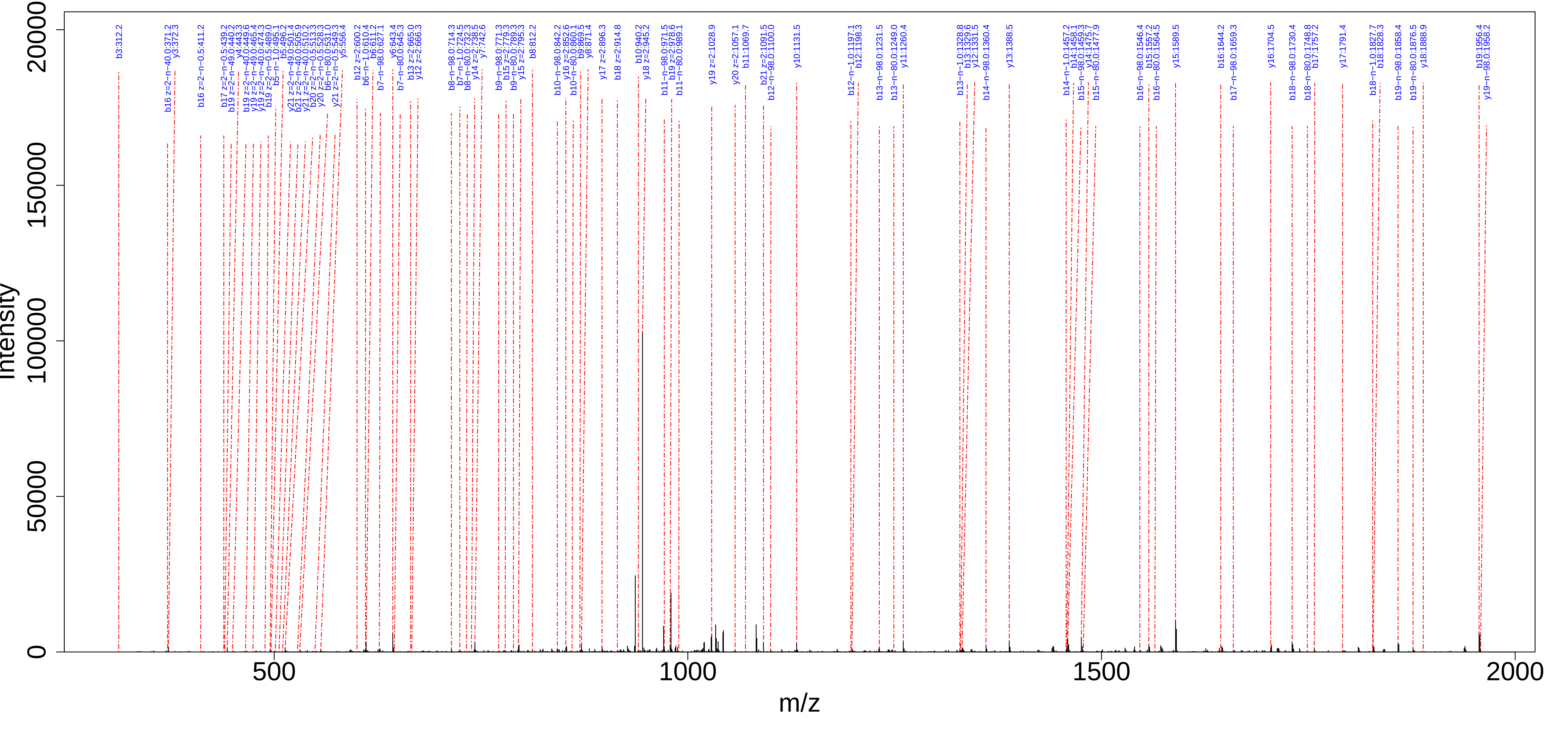

SGSPSDNSGAEEMEVSLAKPK 3−S_Phospho z=+2 Raw scan=2745

AKT1_HUMAN S126; ; Q= 0 ; score= 63.4 ; E= 2.9e−07 m/z=1100.4816

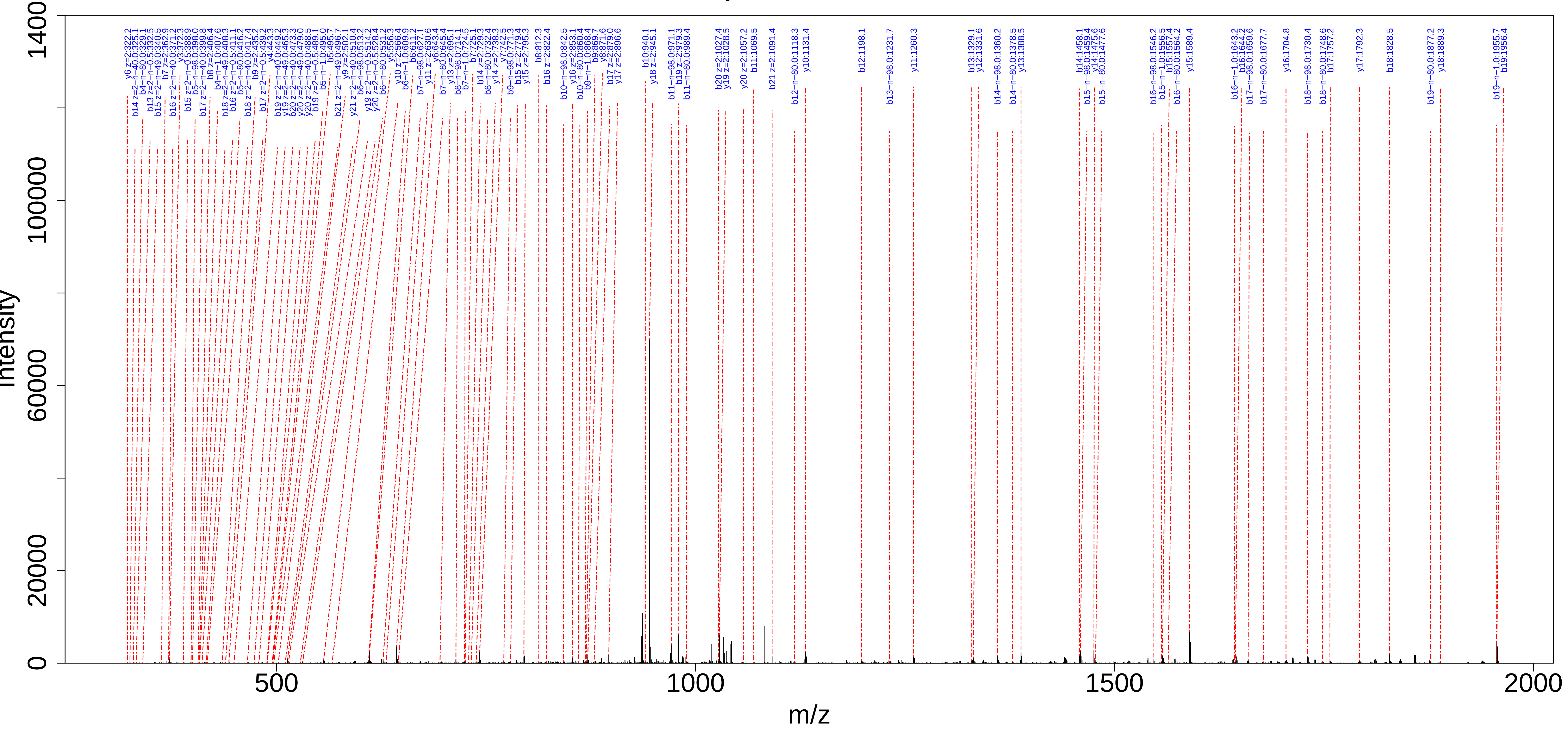

RANTLSHFPIECQEPPQPAR 4−T_Phospho z=+3 Raw scan=2791
TBCD1_HUMAN T596; ; Q= 0 ; score= 62.3 ; E= 4.2e−06 m/z=810.0502

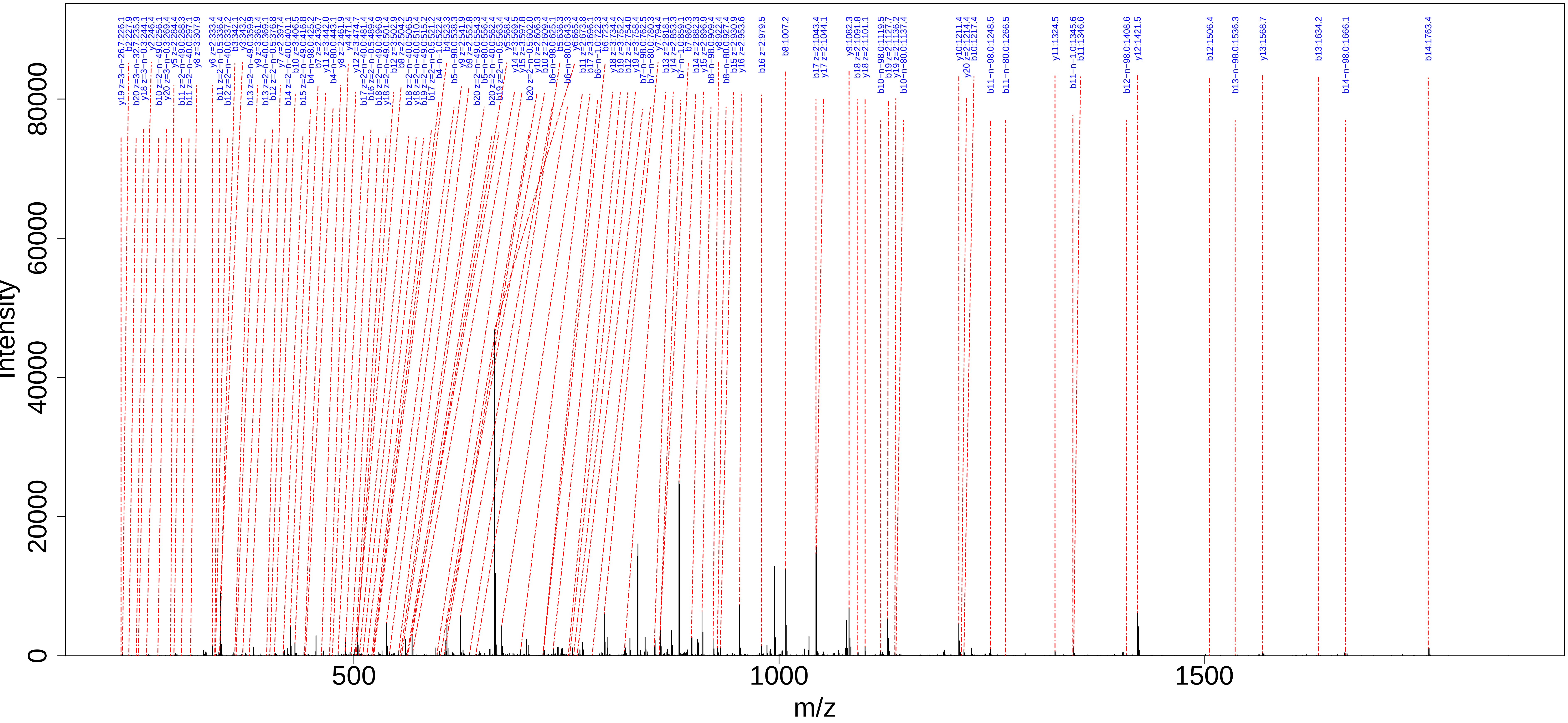

SGSPSDNSGAEEMEVSLAKPK 3−S_Phospho z=+2 Raw scan=2761
AKT1_HUMAN S124; ; Q= 0 ; score= 61.9 ; E= 2.2e−08 m/z=1100.4814

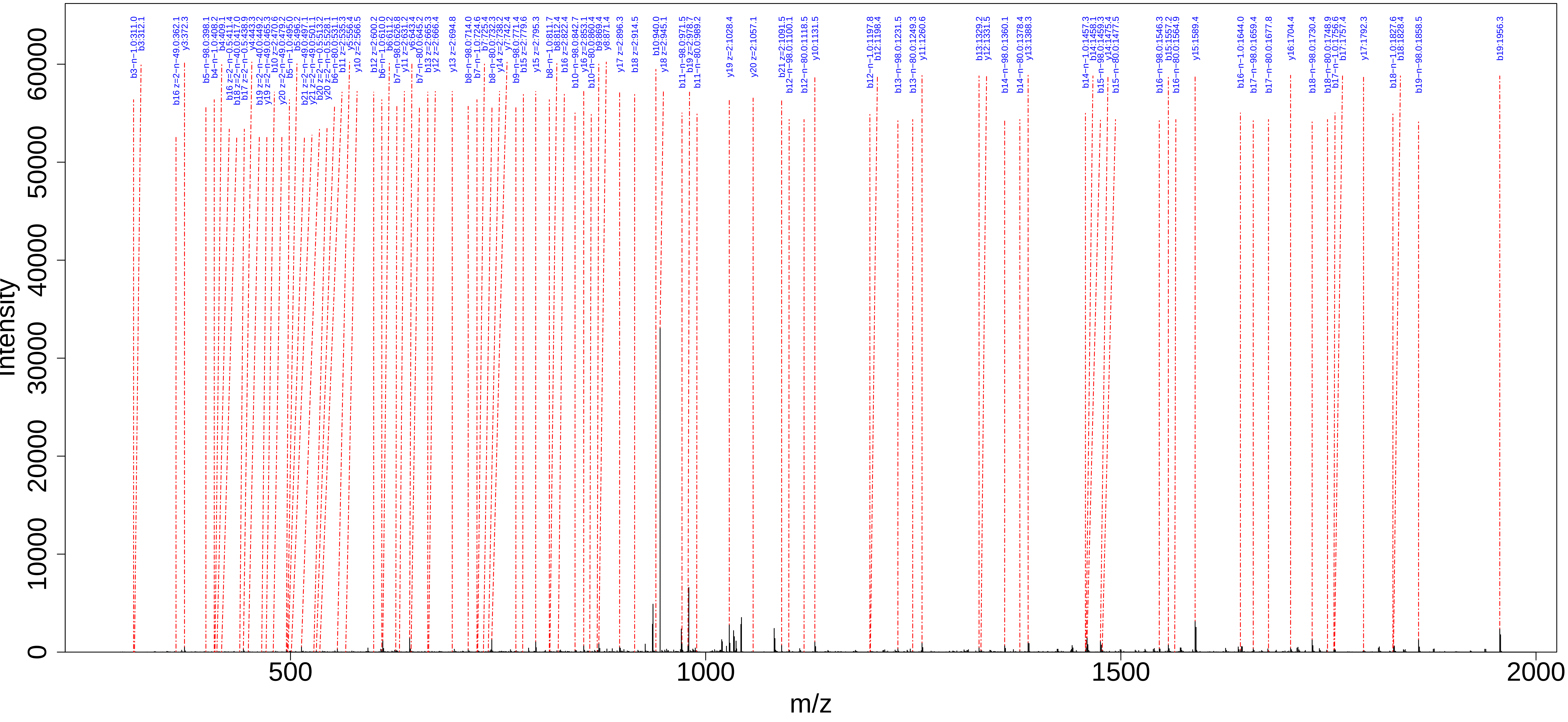

ANTLSHFPIECQEPPQPAR 3−T_Phospho z=+3 Raw scan=3094
TBCD1_HUMAN T596; ; Q= 0 ; score= 61.7 ; E= 3.6e−05 m/z=758.0164

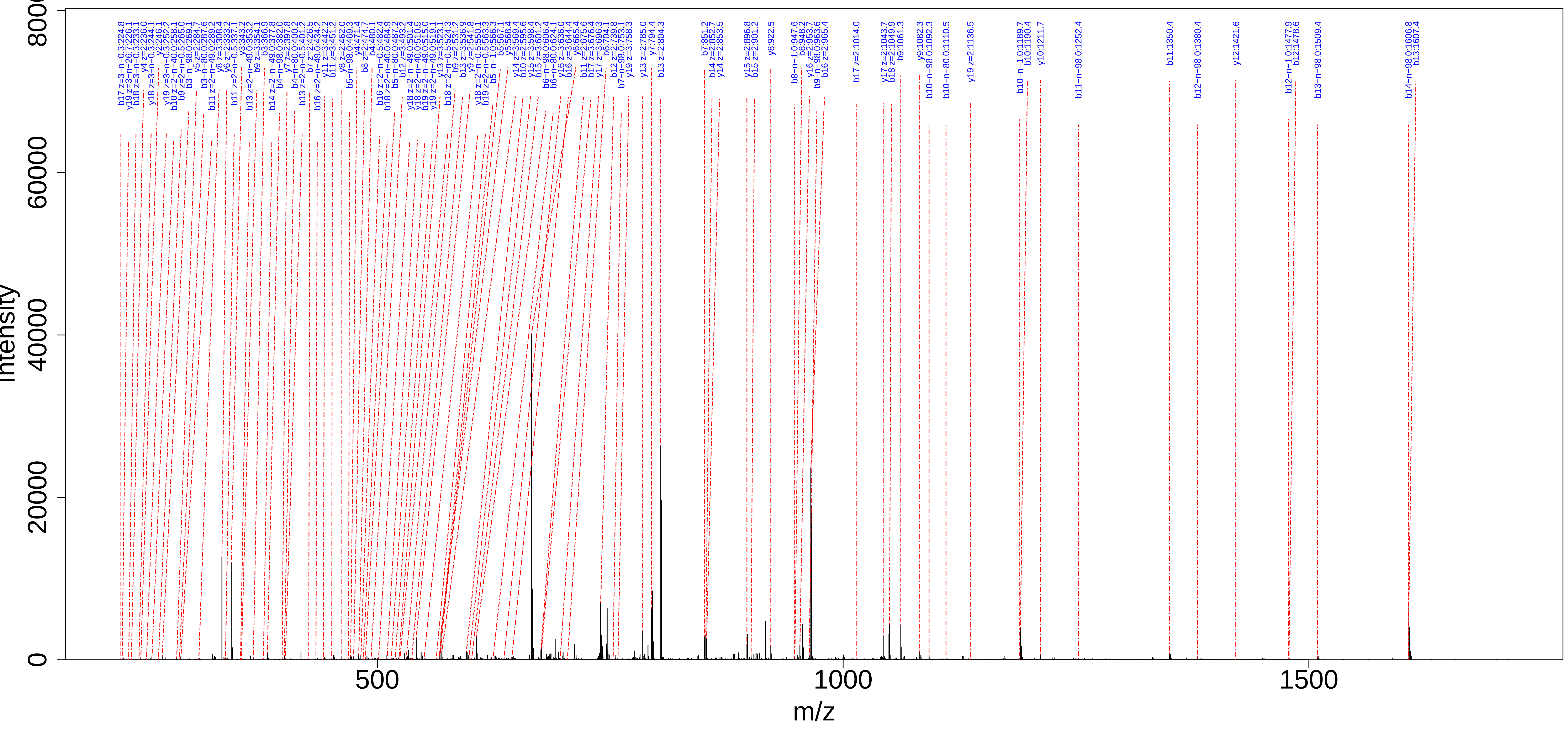

RANTLSHFPIECQEPPQPAR 4−T_Phospho z=+3 Raw scan=2768
TBCD1_HUMAN T596; ; Q= 0 ; score= 61.6 ; E= 2.2e−05 m/z=810.0498

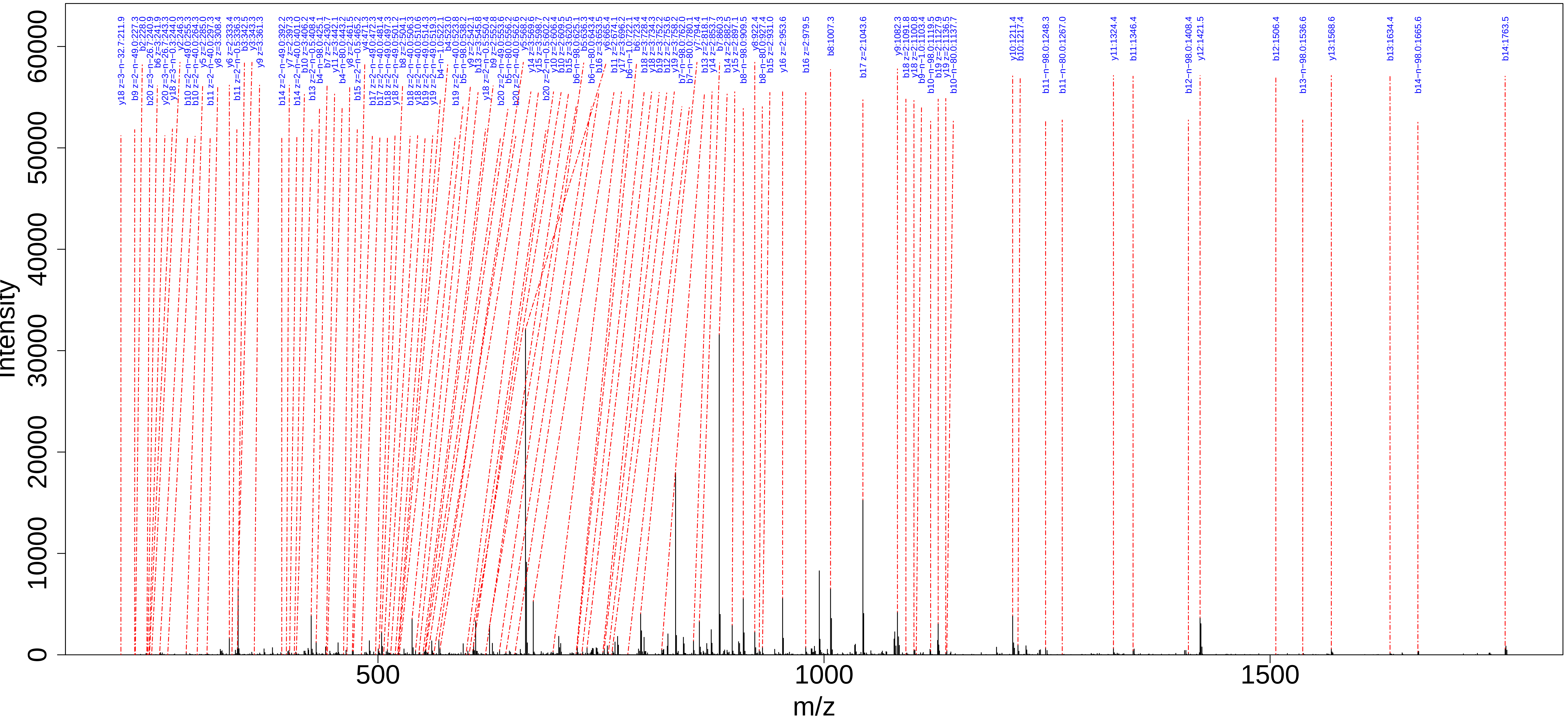

RANTLSHFPIECQEPPQPAR 4−T_Phospho z=+3 Raw scan=2813
TBCD1_HUMAN T596; ; Q= 0 ; score= 56.8 ; E= 0.00041 m/z=810.0478

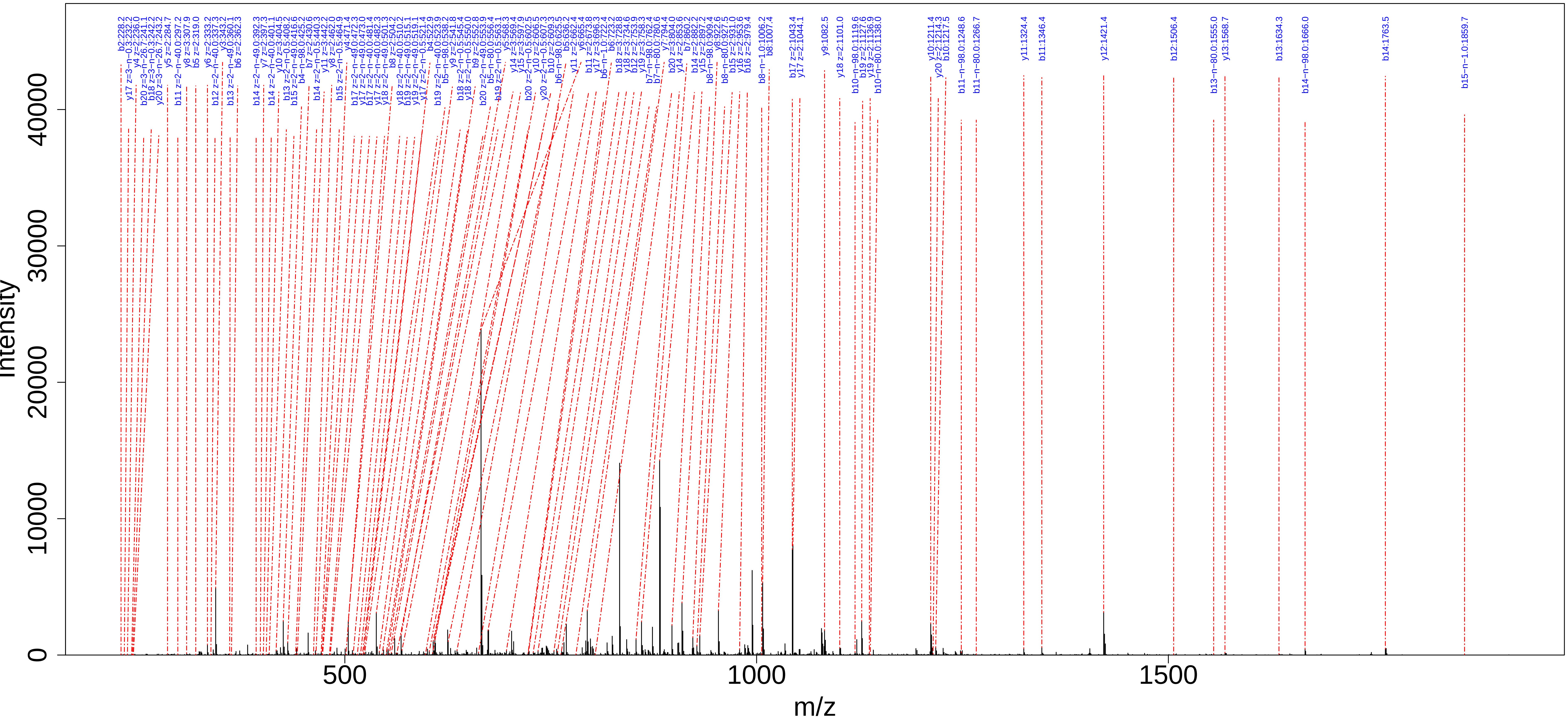

SGSPSDNSGAEEMEVSLAKPK 13−M_Oxidation/1−S_Phospho z=+2 Raw scan=2744
AKT1_HUMAN S137; ; Q= 0 ; score= 56.1 ; E= 2.9e−07 m/z=1108.4796

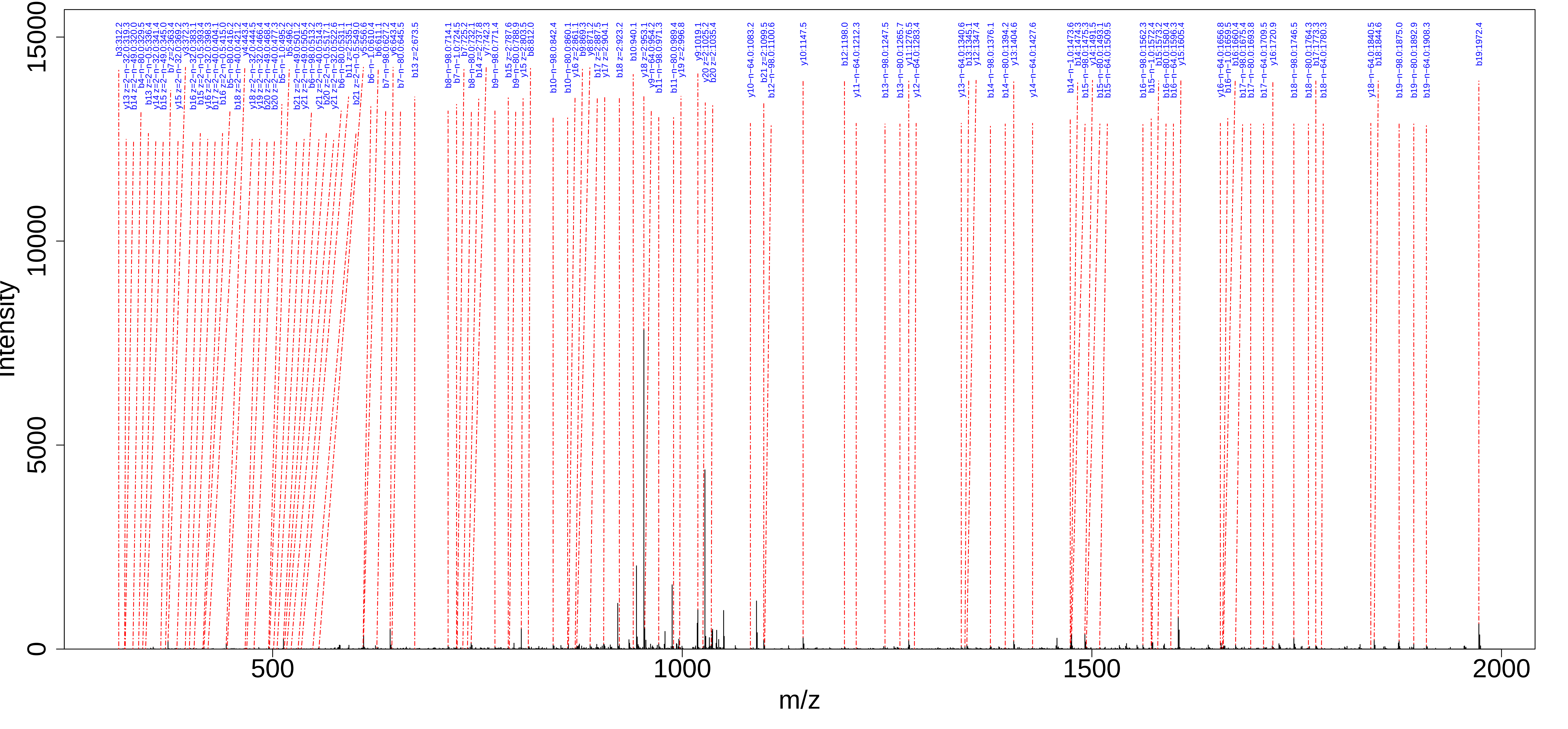

HRVTMNEFEYLK 4−T_Phospho z=+3 Raw scan=3077
AKT1_HUMAN T146; ; Q= 0 ; score= 49.2 ; E= 5e−04 m/z=549.5848

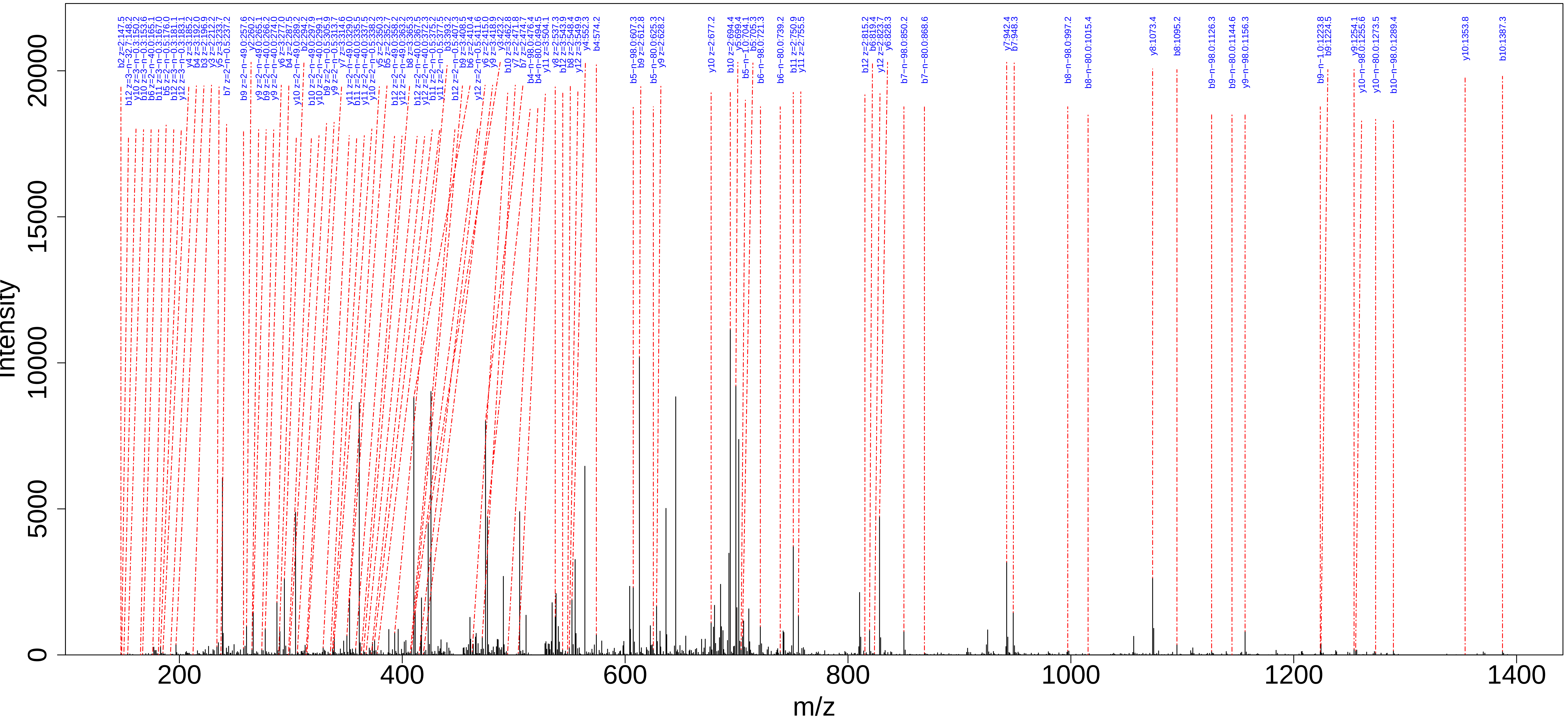

HRVTMNEFEYLK 4−T_Phospho z=+3 Raw scan=3093
AKT1_HUMAN T146; ; Q= 0 ; score= 47.8 ; E= 0.00023 m/z=549.5874

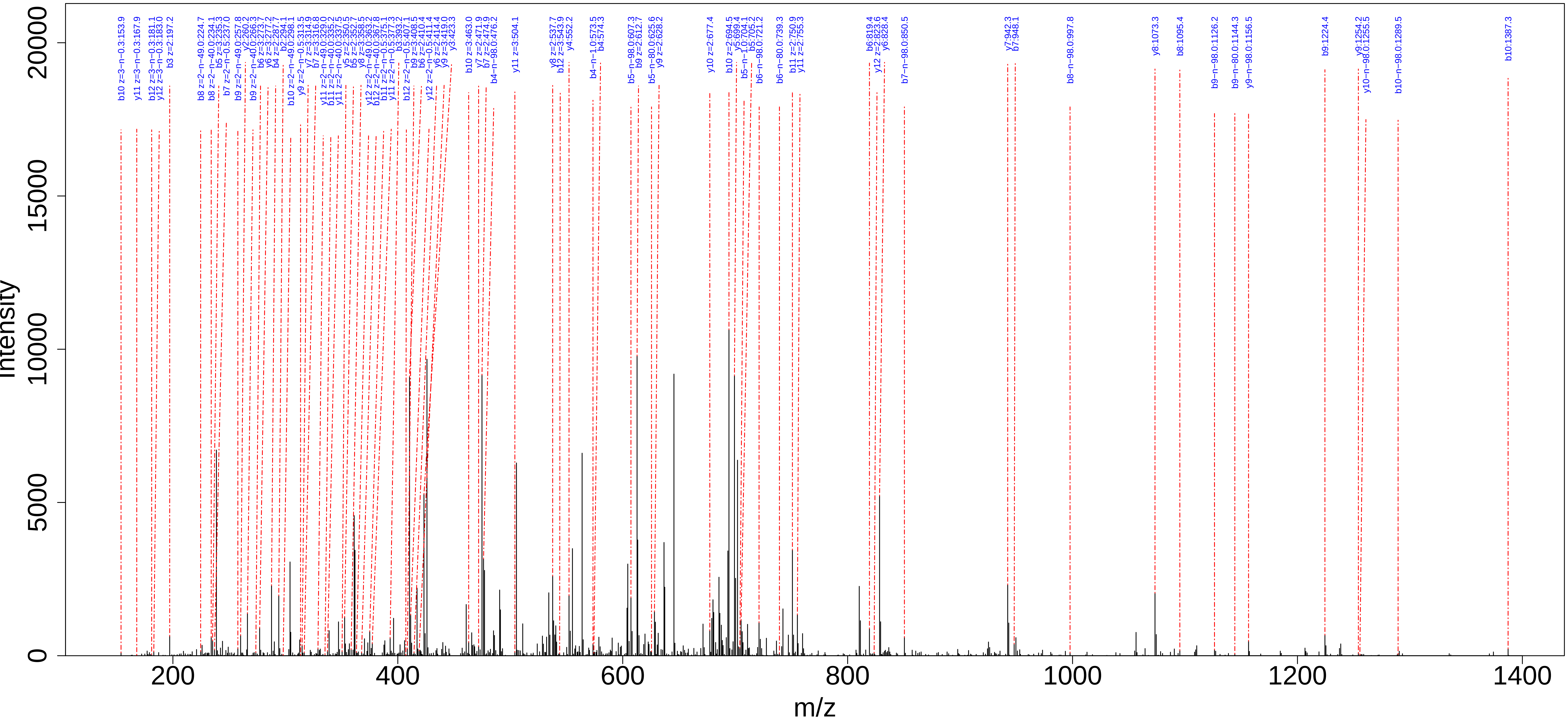

SGSPSDNSGAEEMEVSLAKPK 13−M_Oxidation/5−S_Phospho z=+2 Raw scan=2741
AKT1_HUMAN S126; ; Q= 0.00392156862745098 ; score= 41.7 ; E= 0.00055 m/z=1108.4814

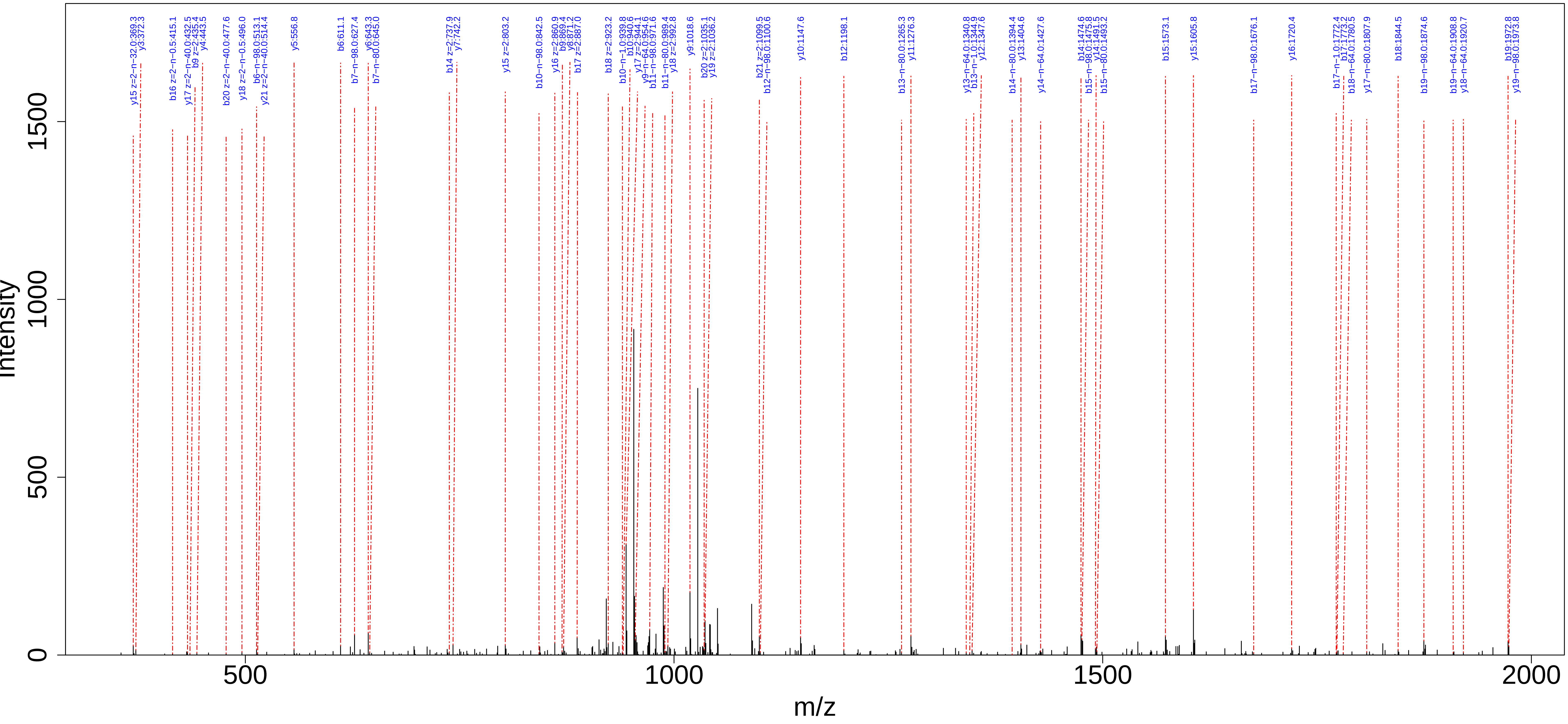

ANTLSHFPIECQEPPQPAR 3−T_Phospho z=+3 Raw scan=3153
TBCD1_HUMAN T596; ; Q= 0.00386473429951691 ; score= 41.5 ; E= 0.0042 m/z=758.0153

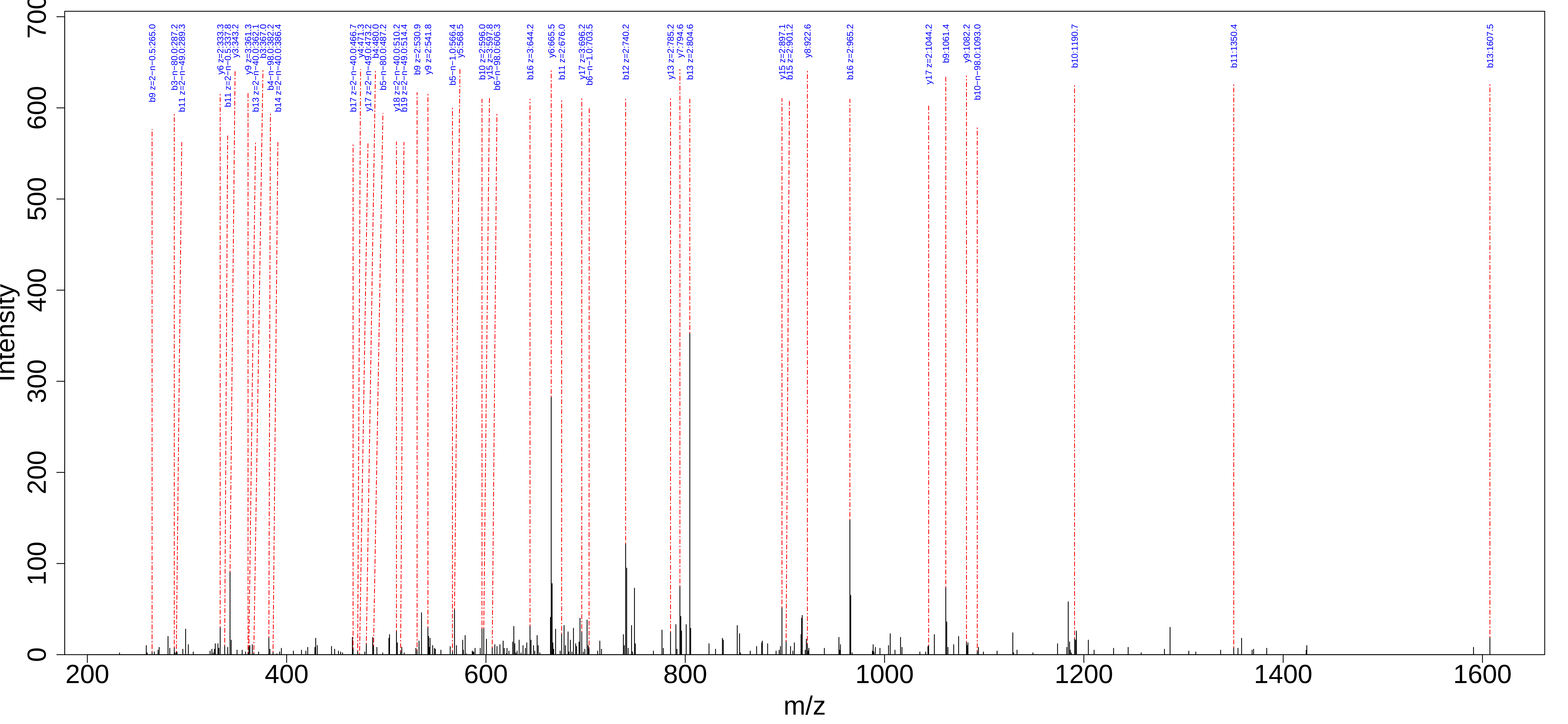

HRVTMNEFEYLK 4−T_Phospho z=+3 Raw scan=3085

AKT1_HUMAN T146; ; Q= 0.00382043935052531 ; score= 41.2 ; E= 0.0031 m/z=549.5869

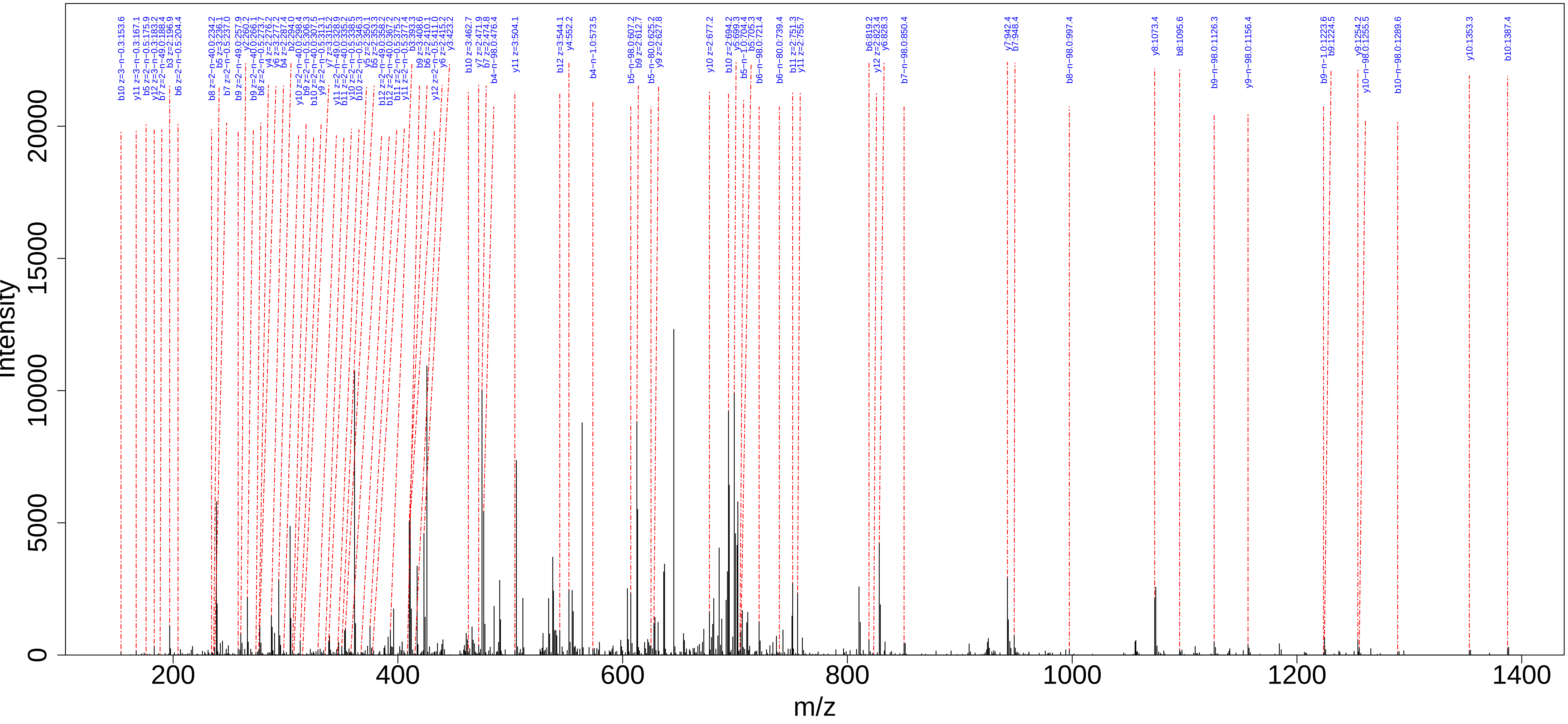

SFSQPGLR 3−S_Phospho z=+2 Raw scan=2316
TBCD1_HUMAN S237; ; Q= 0.0037914691943128 ; score= 40.6 ; E= 9.7e−05 m/z=486.2219

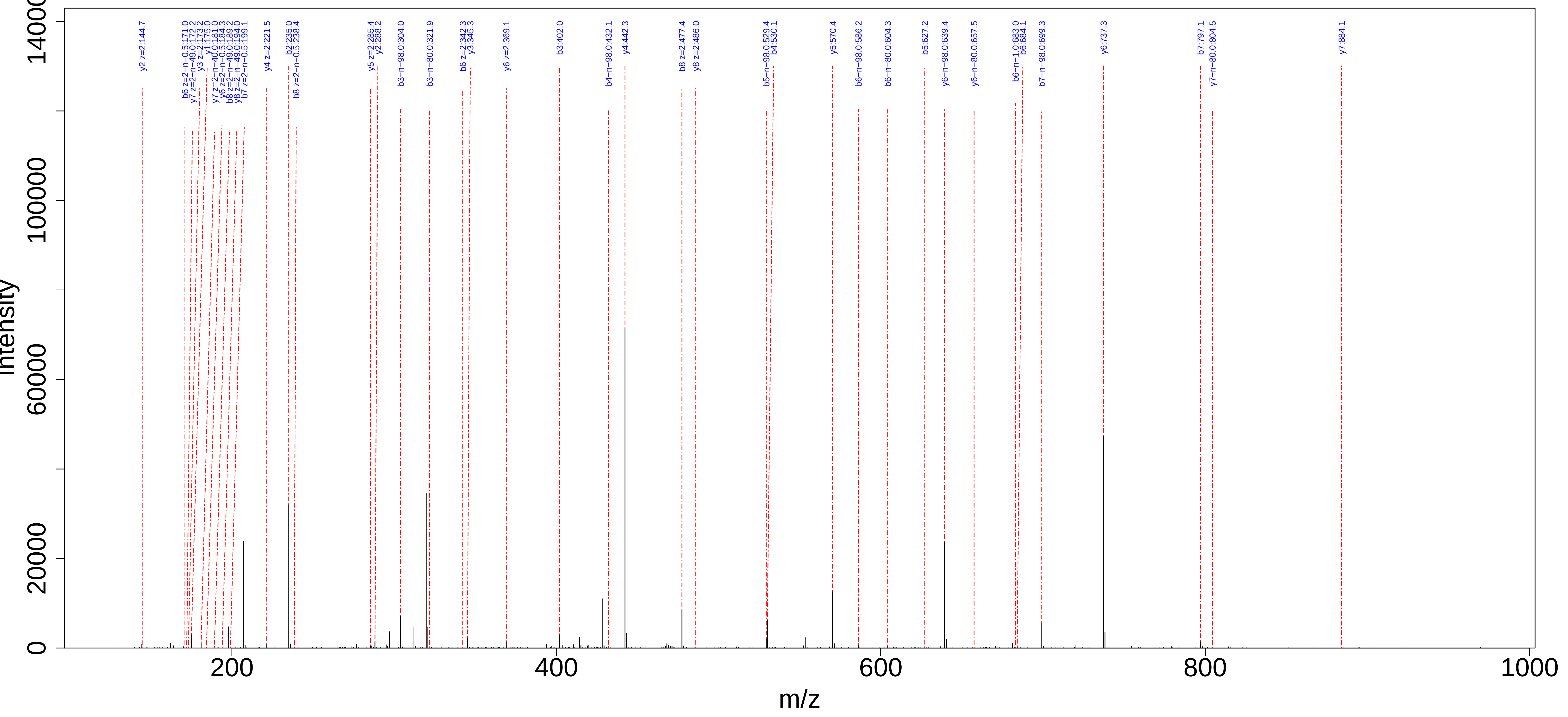

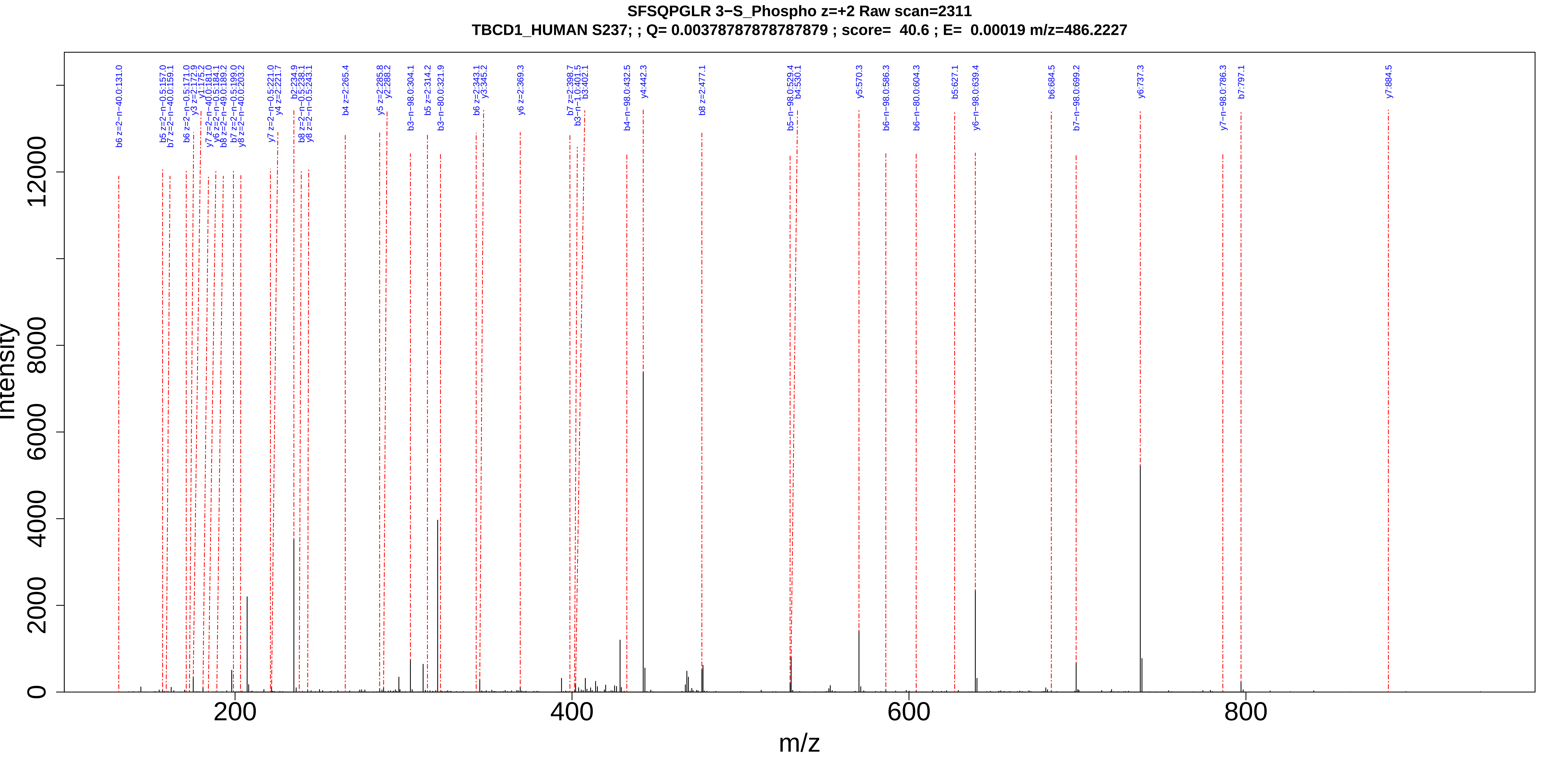
SFSQPGLR 3−S_Phospho z=+2 Raw scan=2311
TBCD1_HUMAN S237; ; Q= 0.00378787878787879 ; score= 40.6 ; E= 0.00019 m/z=486.2227
Intensity
0
2000
4000
6000
8000
12000
200
400
600
800
m/z

HRVTMNEFEYLK 4−T_Phospho z=+2 Raw scan=3078
AKT1_HUMAN T146; ; Q= 0.0036003600360036 ; score= 39.6 ; E= 0.0099 m/z=823.8735

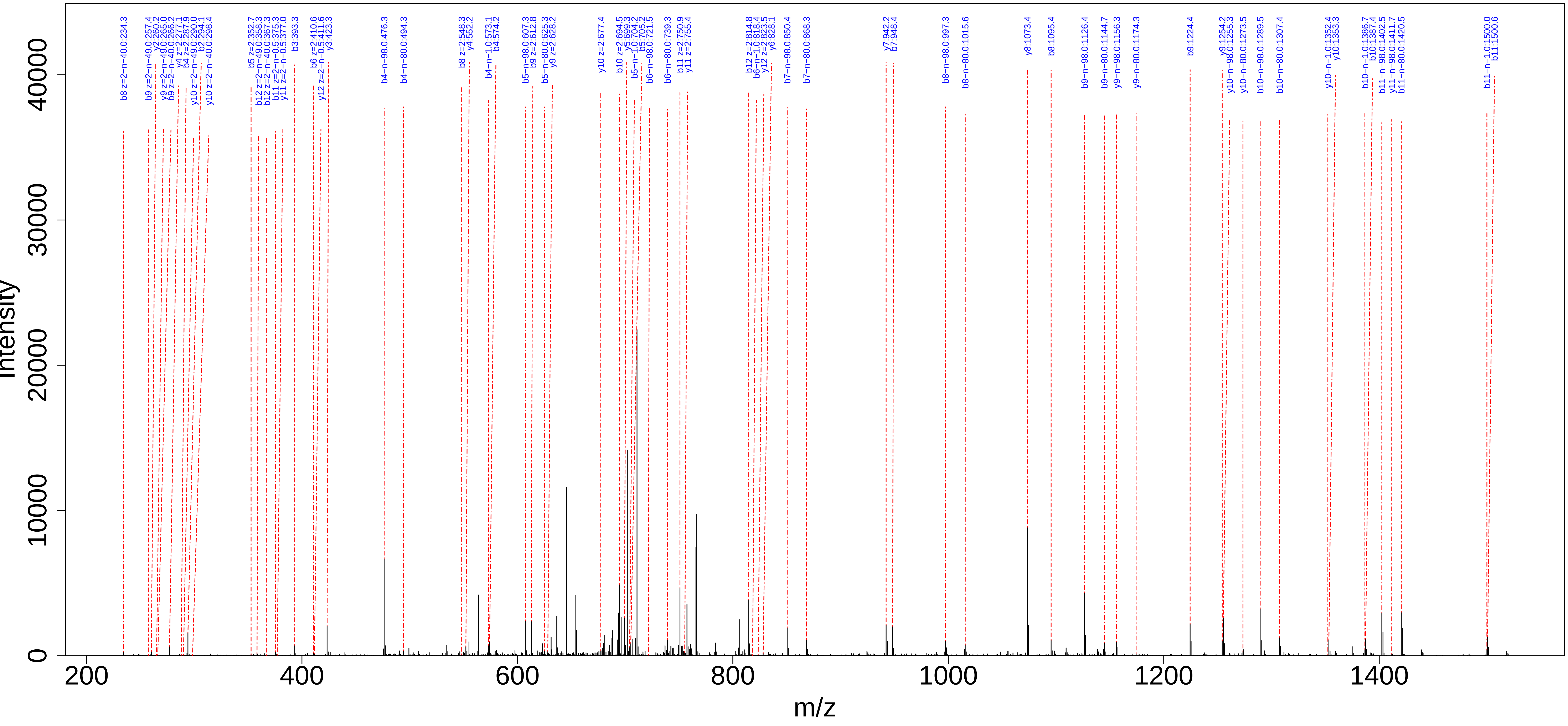

HRVTMNEFEYLK 4−T_Phospho z=+3 Raw scan=3103
AKT1_HUMAN T146; ; Q= 0.00503778337531486 ; score= 39 ; E= 0.021 m/z=549.5856

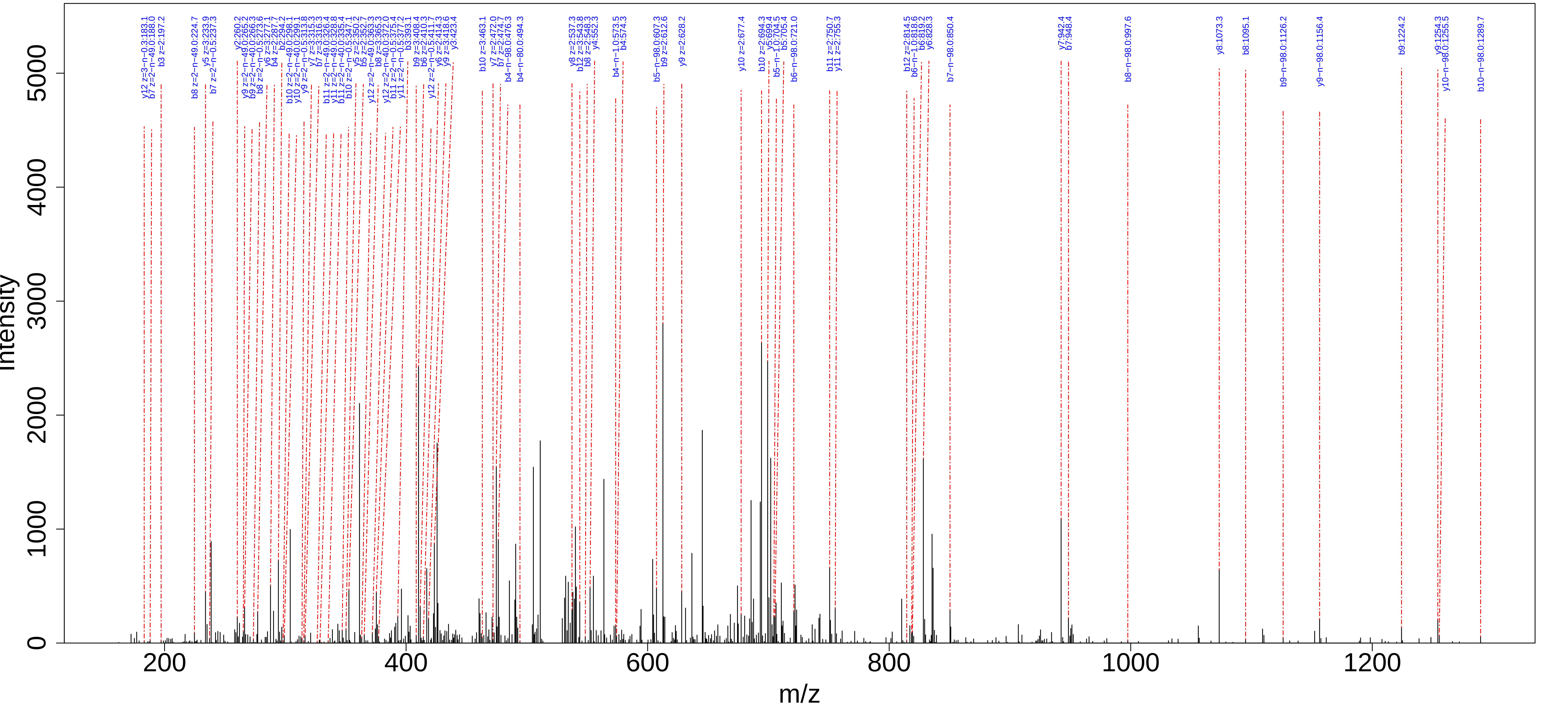

SFSQPGLR 3−S_Phospho z=+2 Raw scan=2347
TBCD1_HUMAN S237; ; Q= 0.0122230710466005 ; score= 36.7 ; E= 0.00044 m/z=486.2221

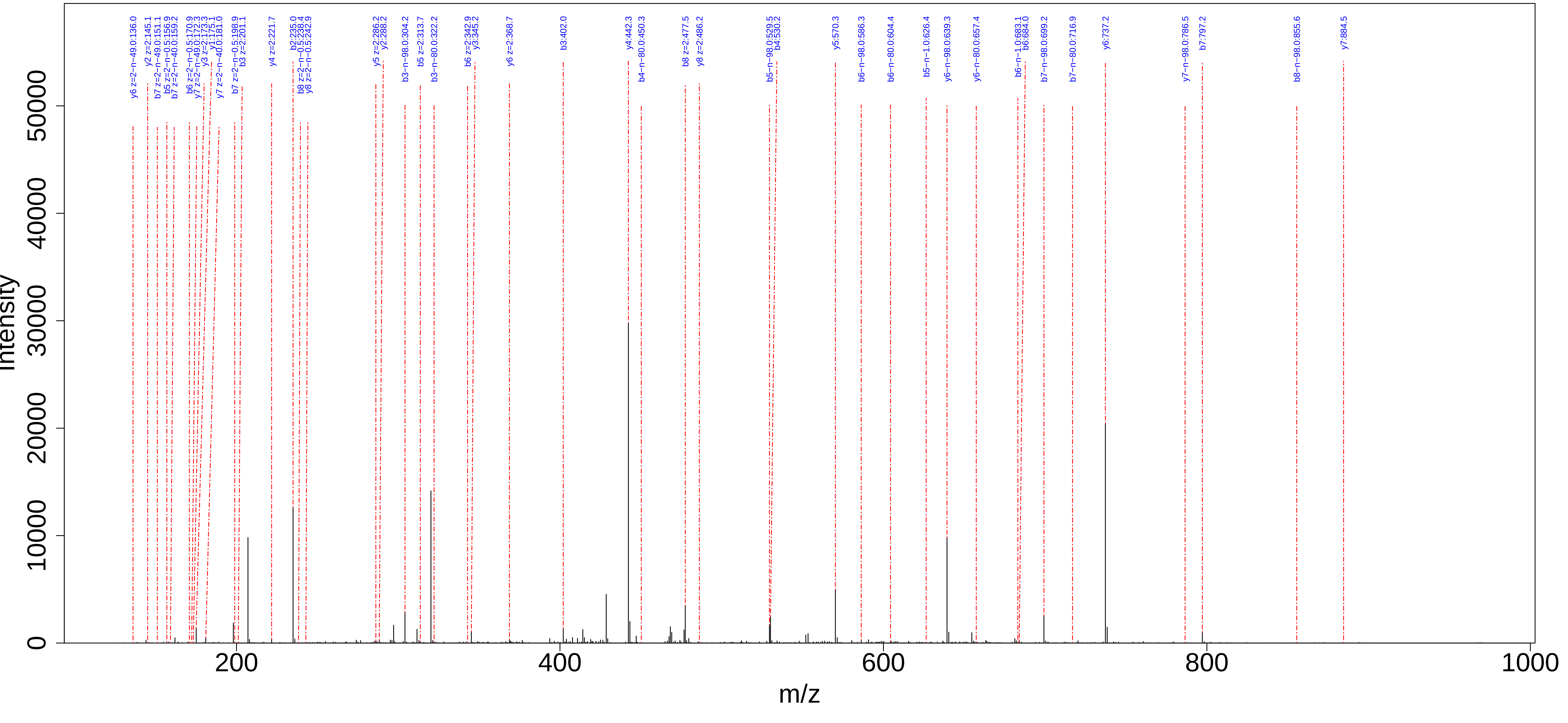

SFSQPGLR 3−S_Phospho z=+2 Raw scan=2355

TBCD1_HUMAN S237; ; Q= 0.0122137404580153 ; score= 36.7 ; E= 0.0011 m/z=486.2222

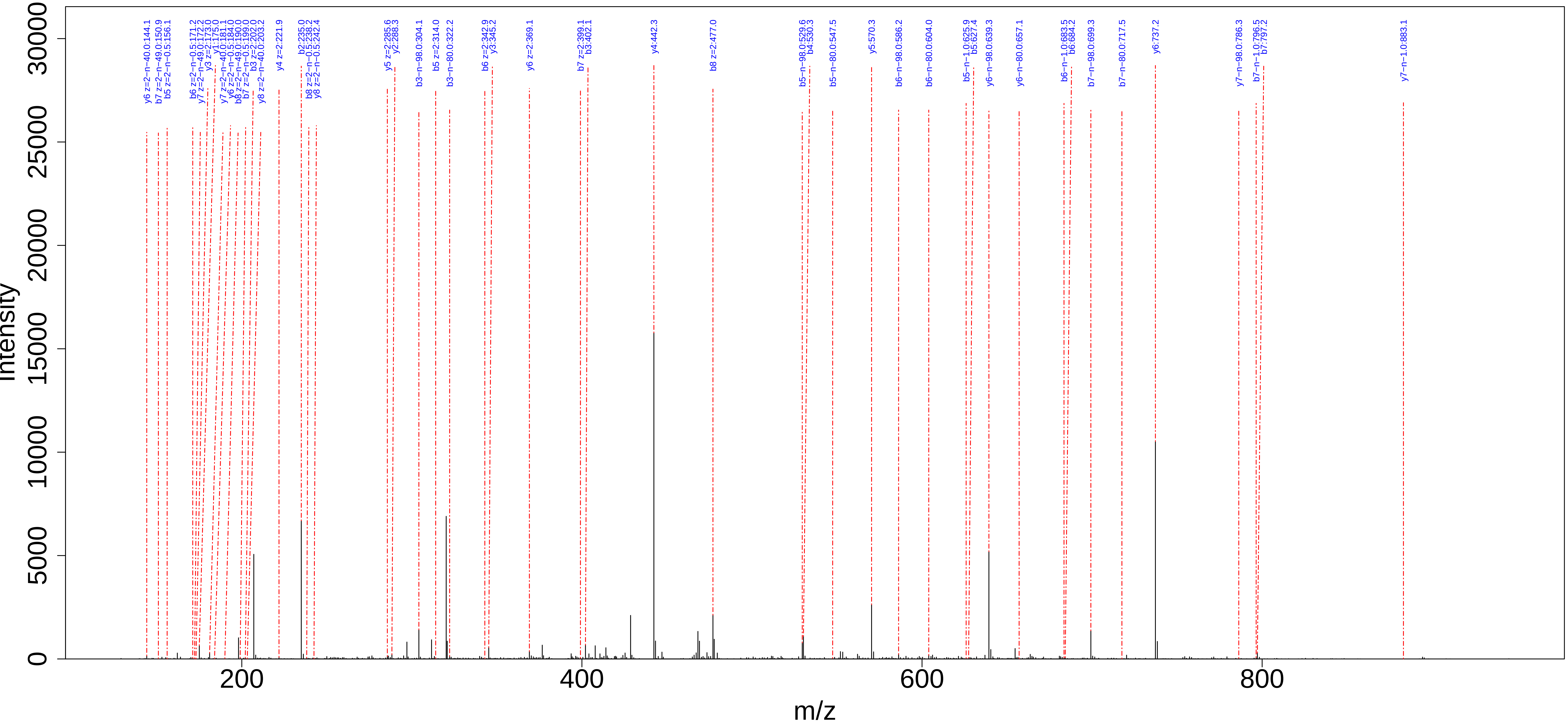

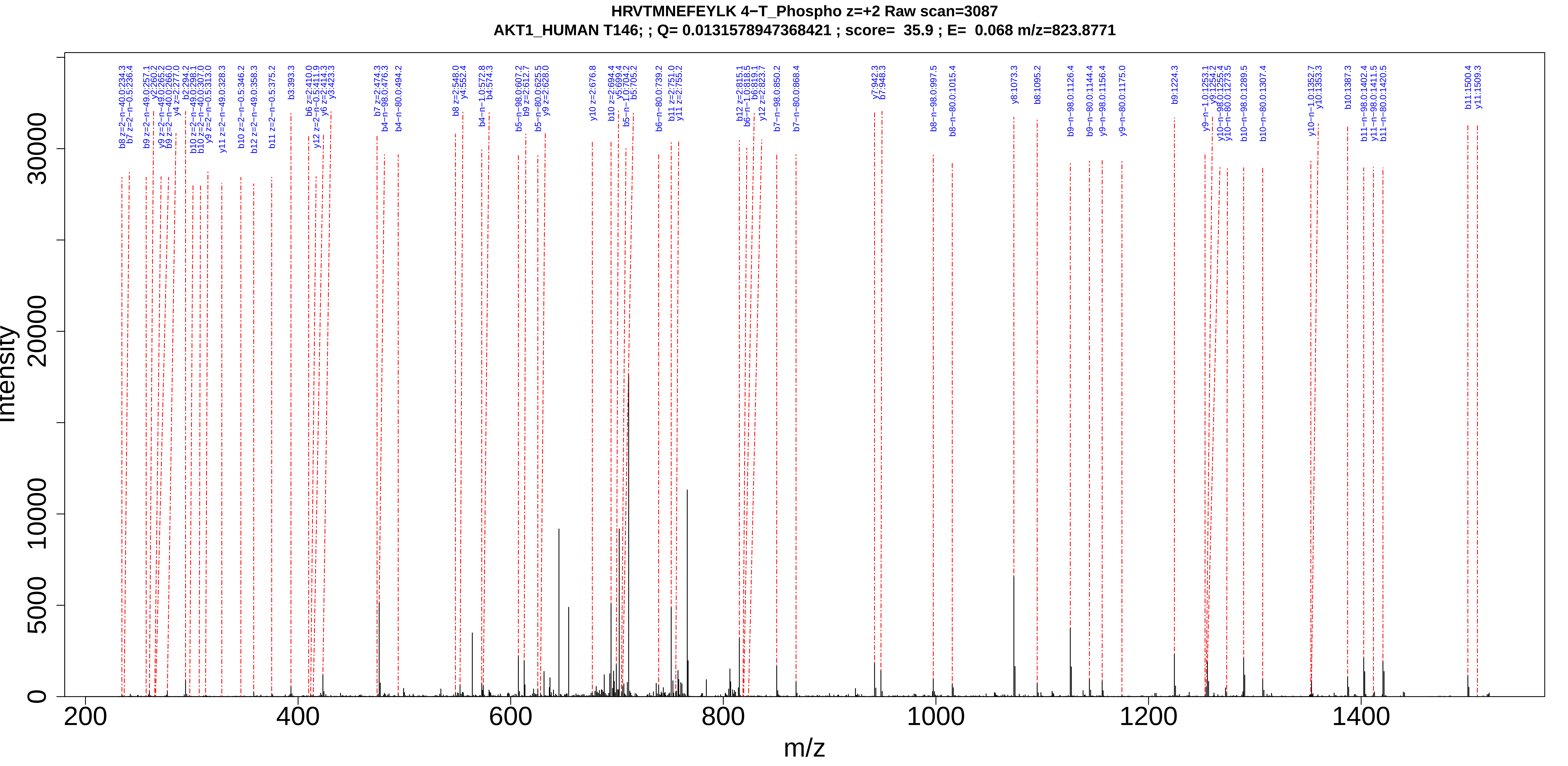
HRVTMNEFEYLK 4−T_Phospho z=+2 Raw scan=3087
AKT1_HUMAN T146; ; Q= 0.0131578947368421 ; score= 35.9 ; E= 0.068 m/z=823.8771
Intensity
m/z

QKLQASENDLLNKR 6−S_Phospho z=+3 Raw scan=2195
TBCD1_HUMAN S747; ; Q= 0.0123537061118336 ; score= 34 ; E= 0.021 m/z=579.9625

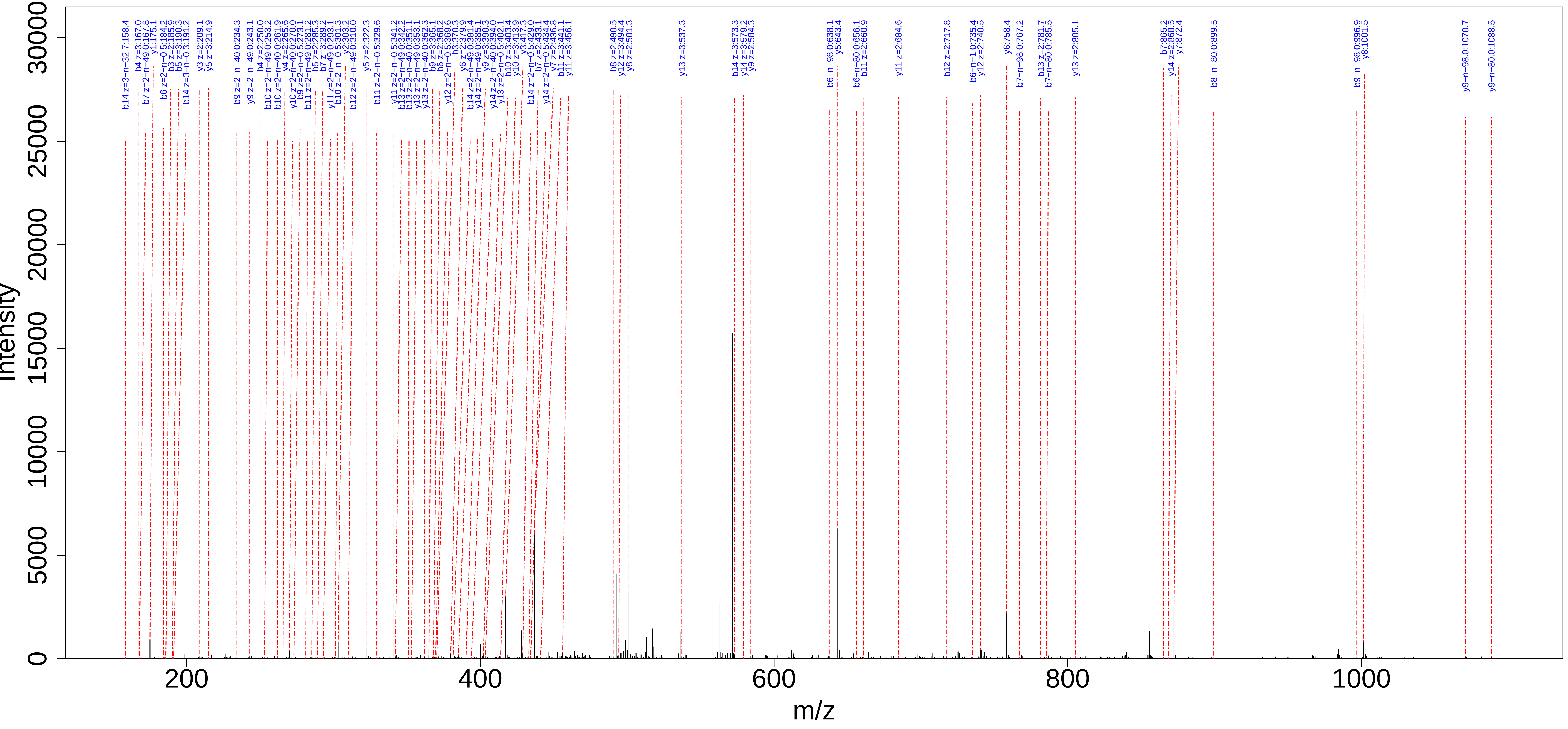

QKLQASENDLLNKR 6−S_Phospho z=+3 Raw scan=2187
TBCD1_HUMAN S747; ; Q= 0.0150753768844221 ; score= 31.8 ; E= 0.088 m/z=579.9608

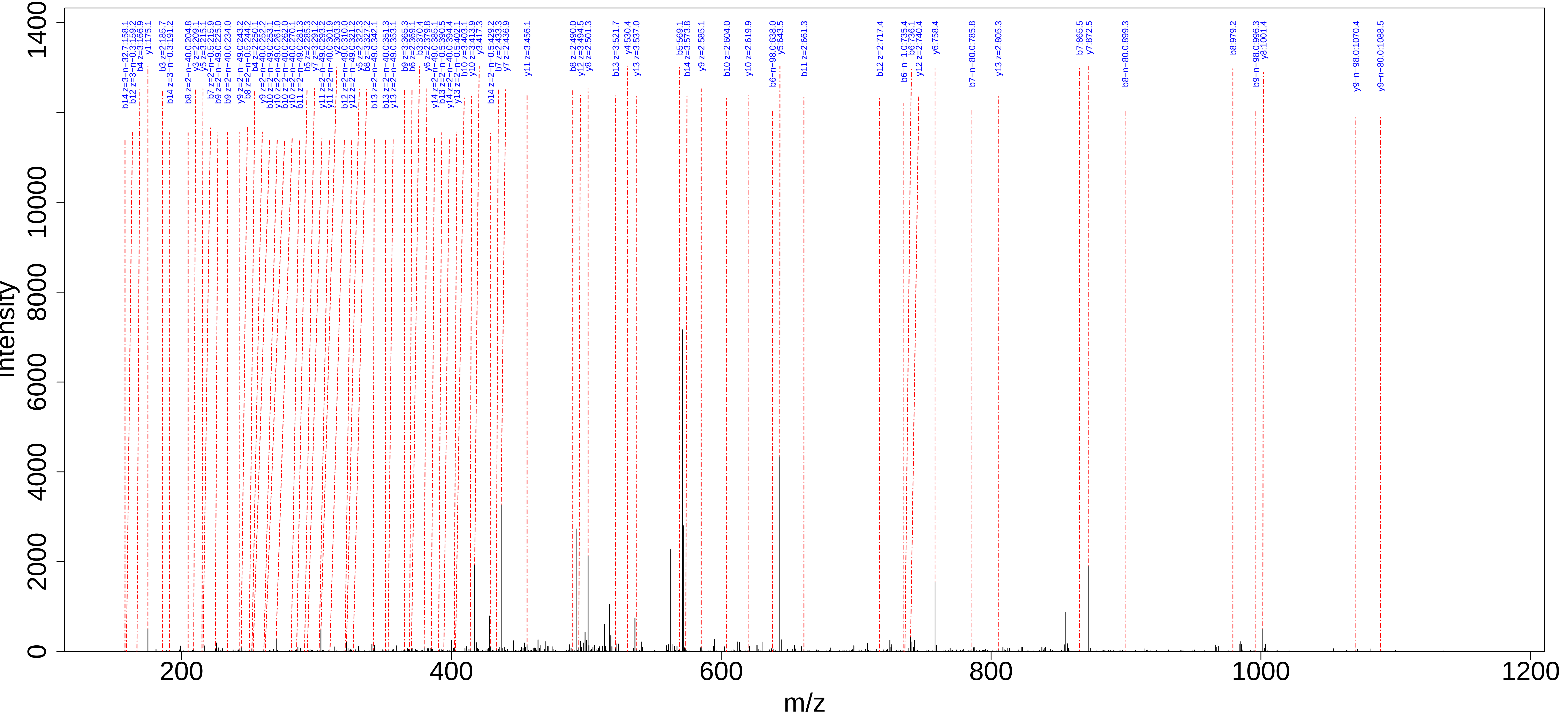

annotated raw MSMS spectra for assay:

Protein kinase: AKT1

Substrate: TBC1D1

sample name: TBCD1_AKT1_S1

LLGSSEDLSSDSESHLPEEPAPLSPQQAFR 24−S_Phospho z=+3 Raw scan=3708
TBCD1_HUMAN S585; ; Q= 0 ; score= 103.3 ; E= 1e−15 m/z=1101.8433

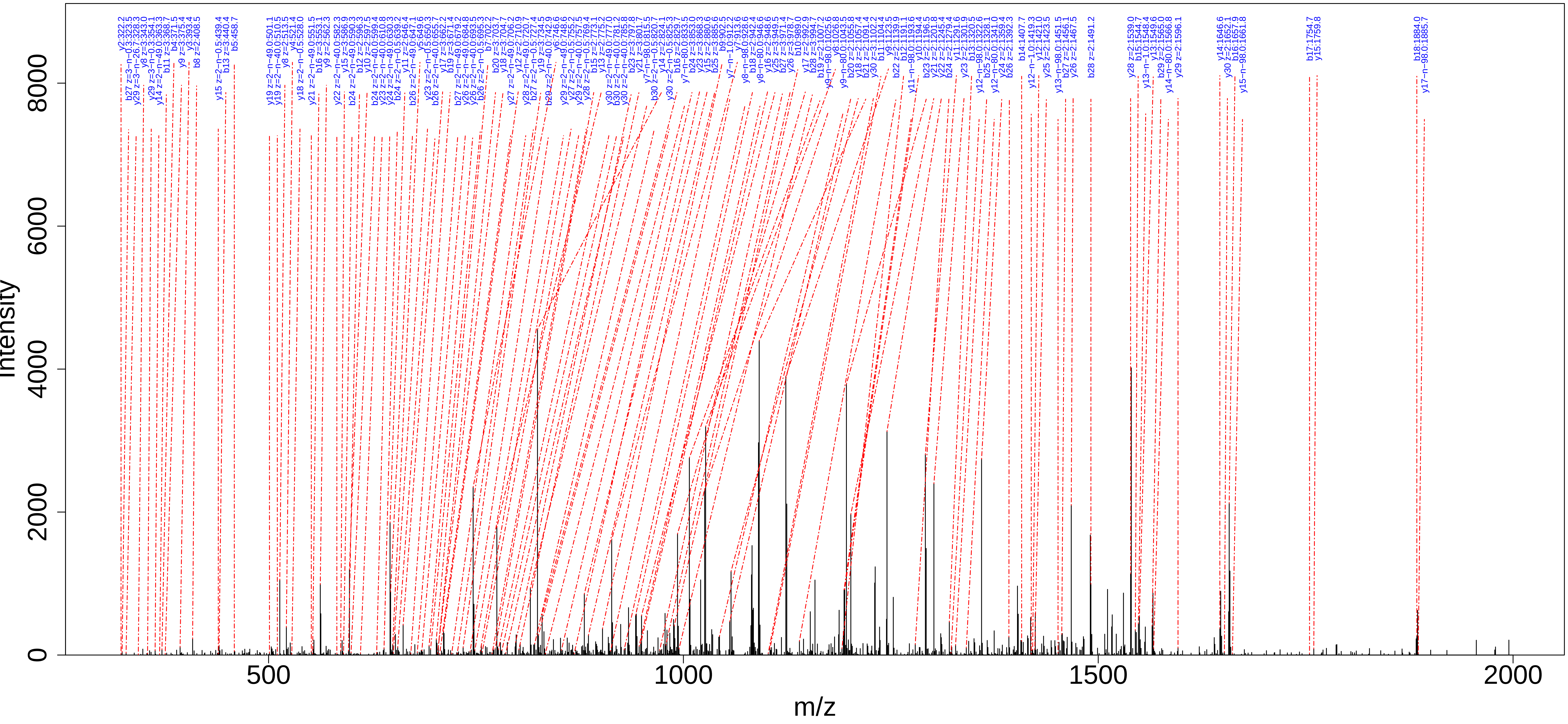

LLGSSEDLSSDSESHLPEEPAPLSPQQAFR 24−S_Phospho z=+3 Raw scan=3716
TBCD1_HUMAN S585; ; Q= 0 ; score= 98.9 ; E= 1e−15 m/z=1101.8419

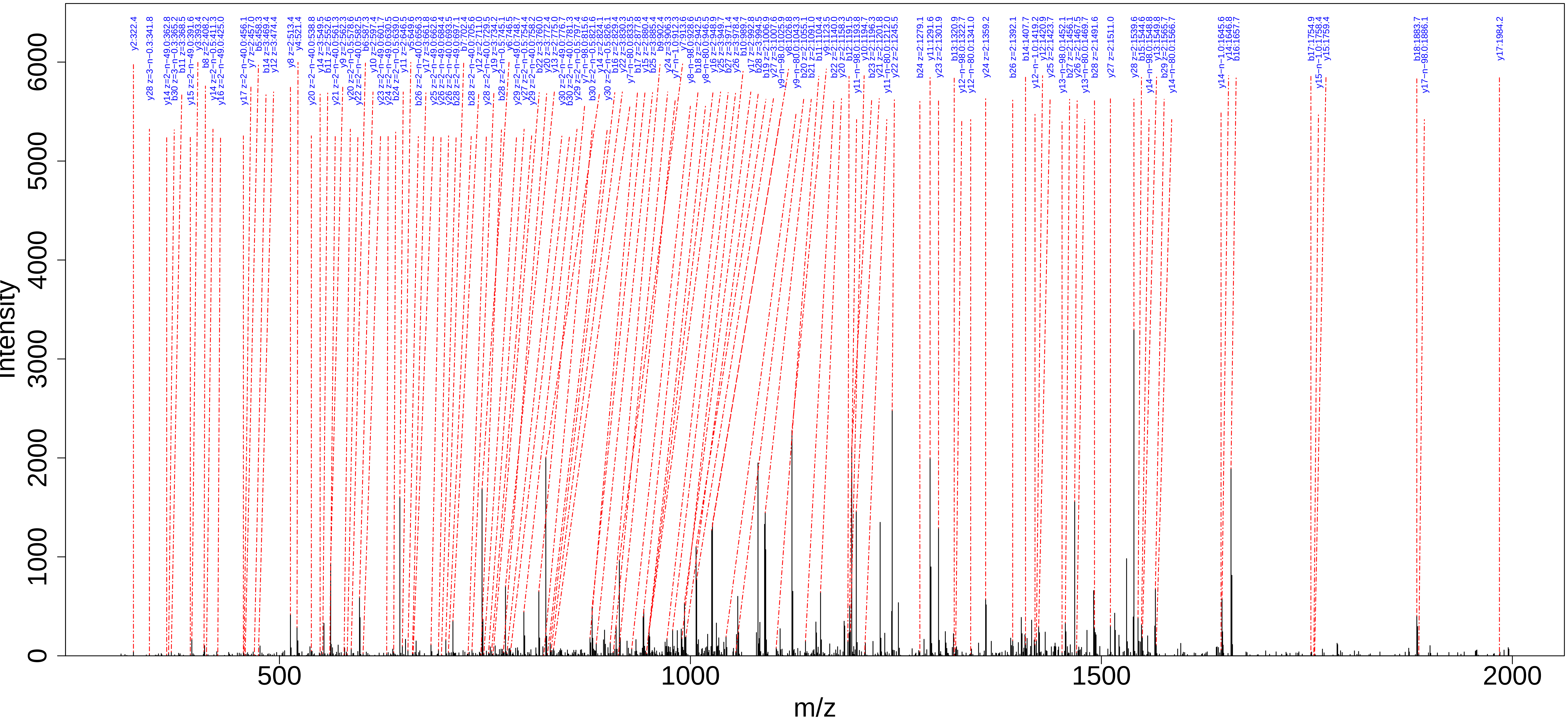

LLGSSEDLSSDSESHLPEEPAPLSPQQAFR 4−S_Phospho z=+3 Raw scan=3787
TBCD1_HUMAN S570; ; Q= 0 ; score= 81.7 ; E= 9.1e−13 m/z=1101.8424

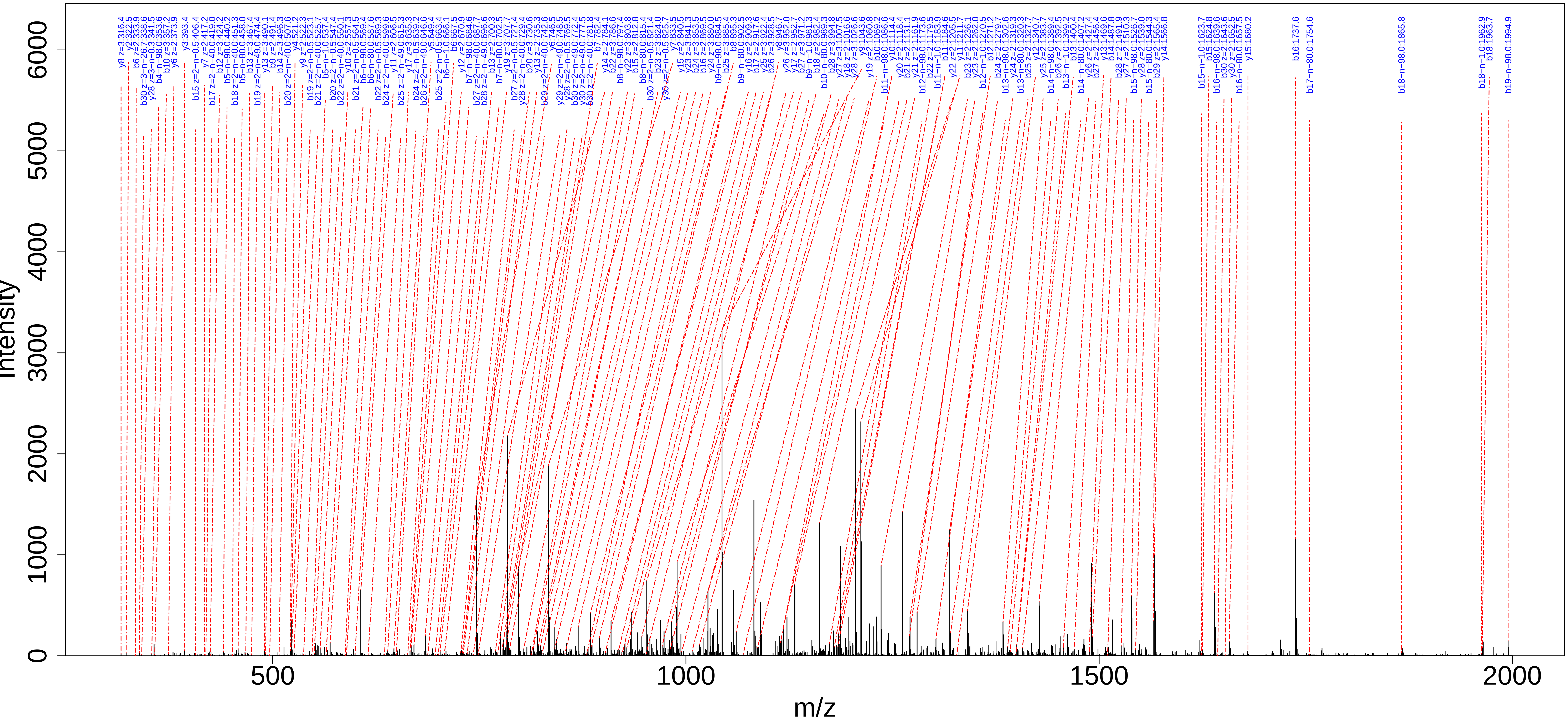

SGSPSDNSGAEEMEVSLAKPK 1−S_Phospho z=+2 Raw scan=2607
AKT1_HUMAN S137; ; Q= 0 ; score= 74.6 ; E= 9.2e−10 m/z=1100.478

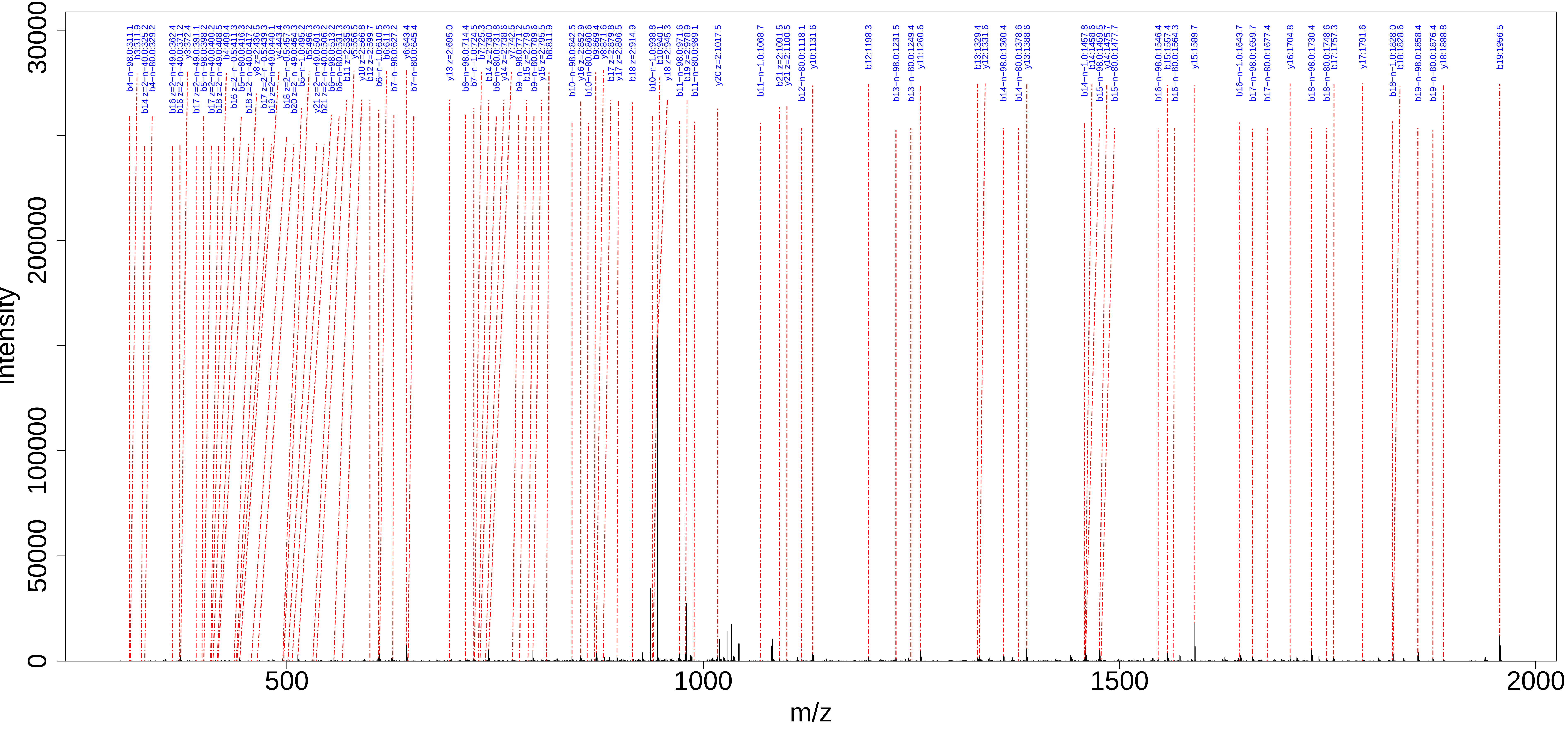

ANTLSHFPIECQEPPQPAR 3−T_Phospho z=+3 Raw scan=3001
TBCD1_HUMAN T596; ; Q= 0 ; score= 74.5 ; E= 3.1e−11 m/z=758.0106

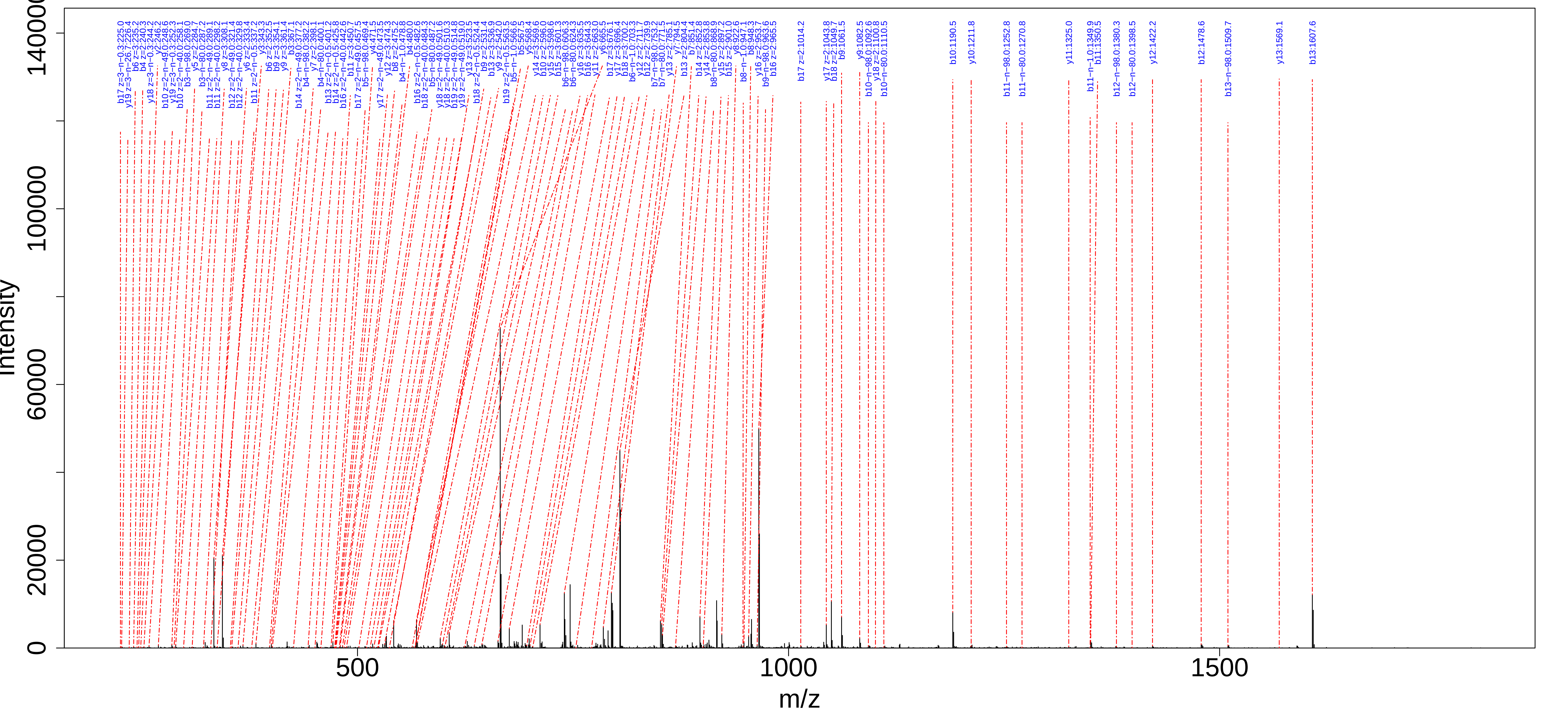

ANTLSHFPIECQEPPQPAR 3−T_Phospho z=+3 Raw scan=2994
TBCD1_HUMAN T596; ; Q= 0 ; score= 72.1 ; E= 2.2e−10 m/z=758.0138

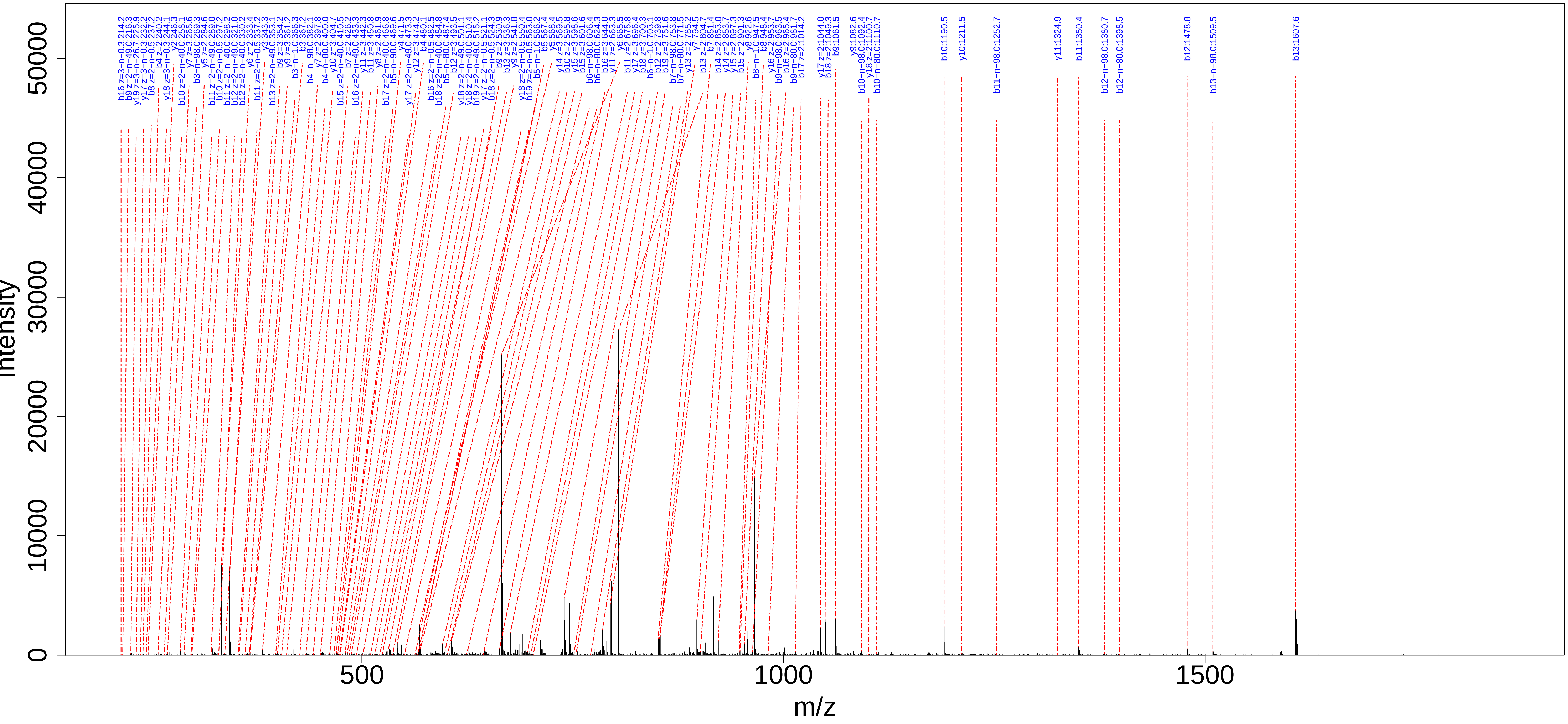

ANTLSHFPIECQEPPQPAR 3−T_Phospho z=+3 Raw scan=3006
TBCD1_HUMAN T596; ; Q= 0 ; score= 71.4 ; E= 1.9e−10 m/z=758.0134

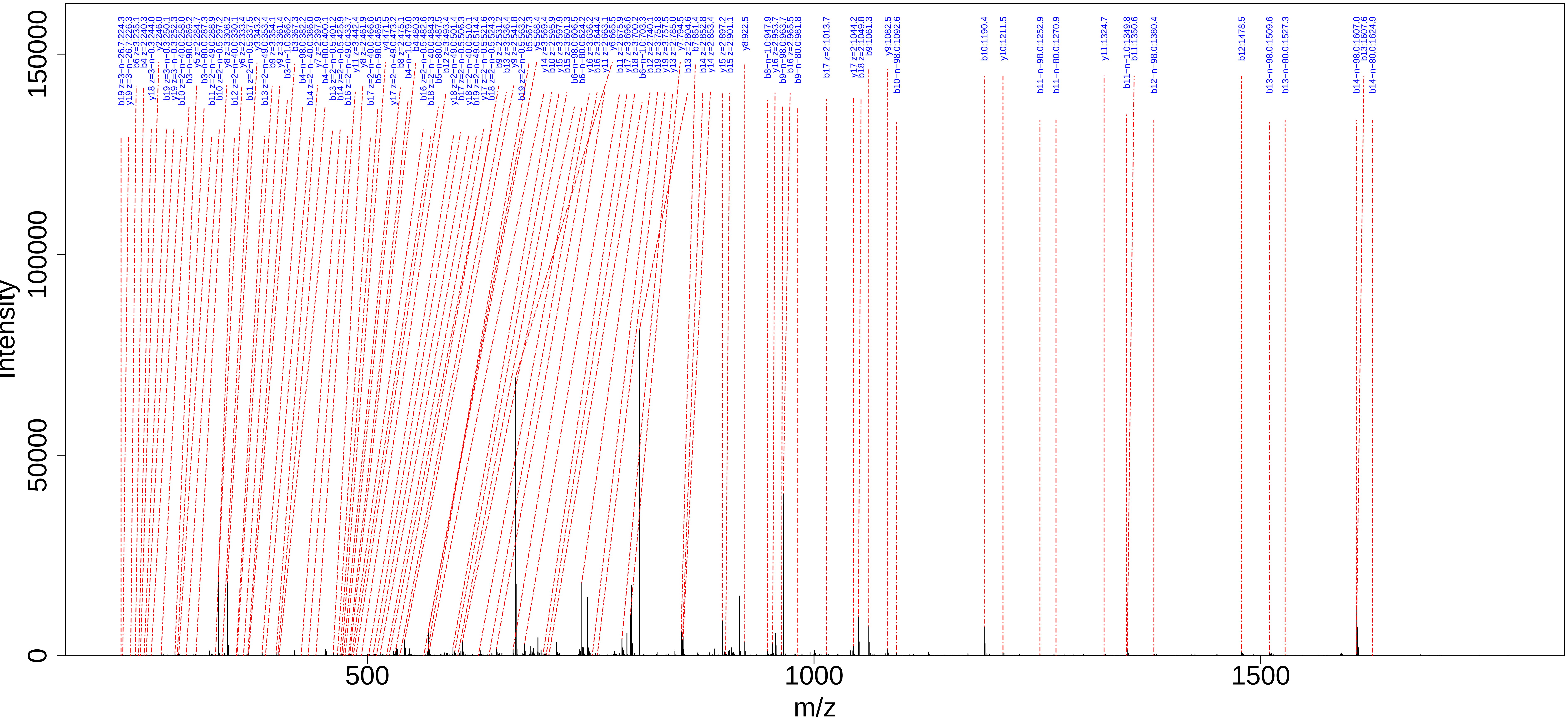

ANTLSHFPIECQEPPQPAR 3−T_Phospho z=+3 Raw scan=3022
TBCD1_HUMAN T596; ; Q= 0 ; score= 68.7 ; E= 1.6e−08 m/z=758.012

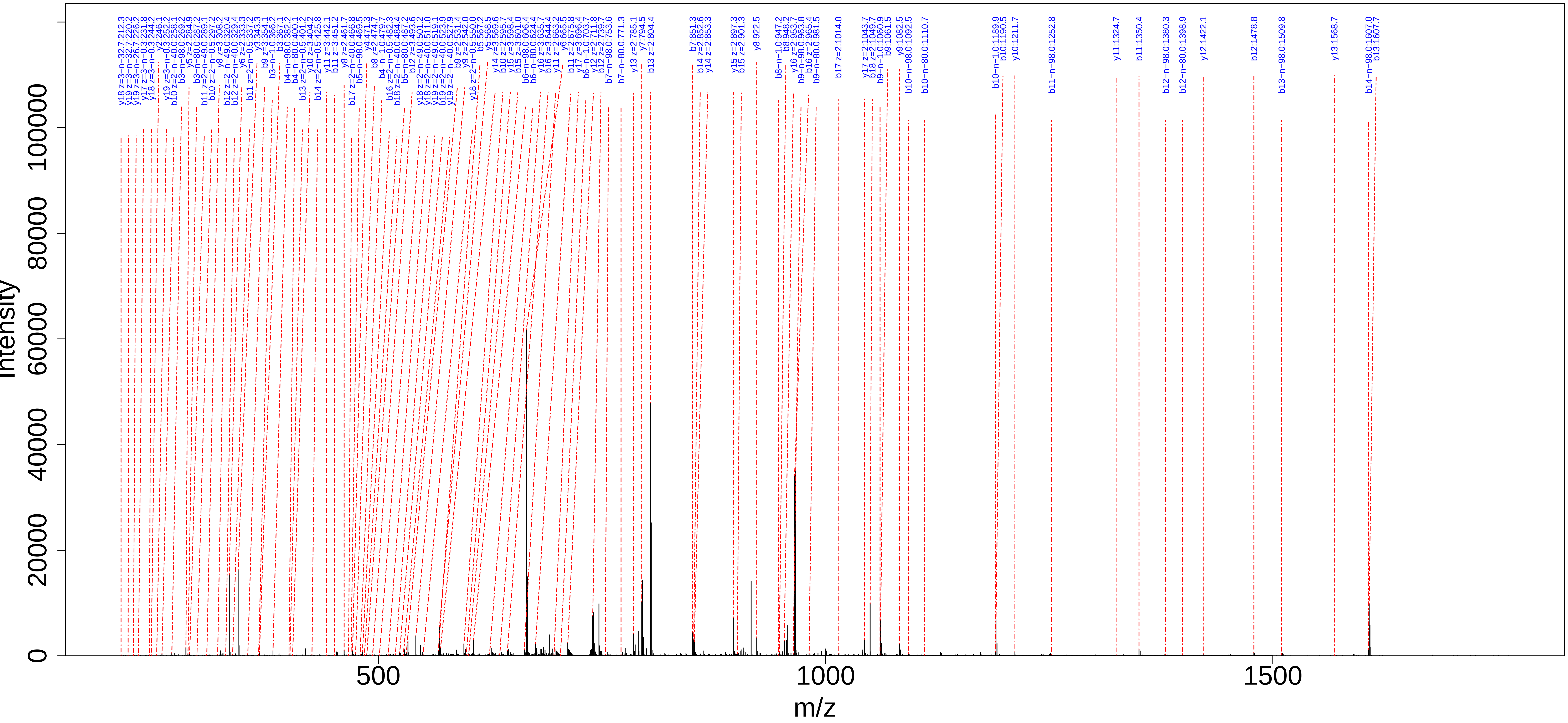

ANTLSHFPIECQEPPQPAR 3−T_Phospho z=+3 Raw scan=3051
TBCD1_HUMAN T596; ; Q= 0 ; score= 68.4 ; E= 3.4e−09 m/z=758.0126

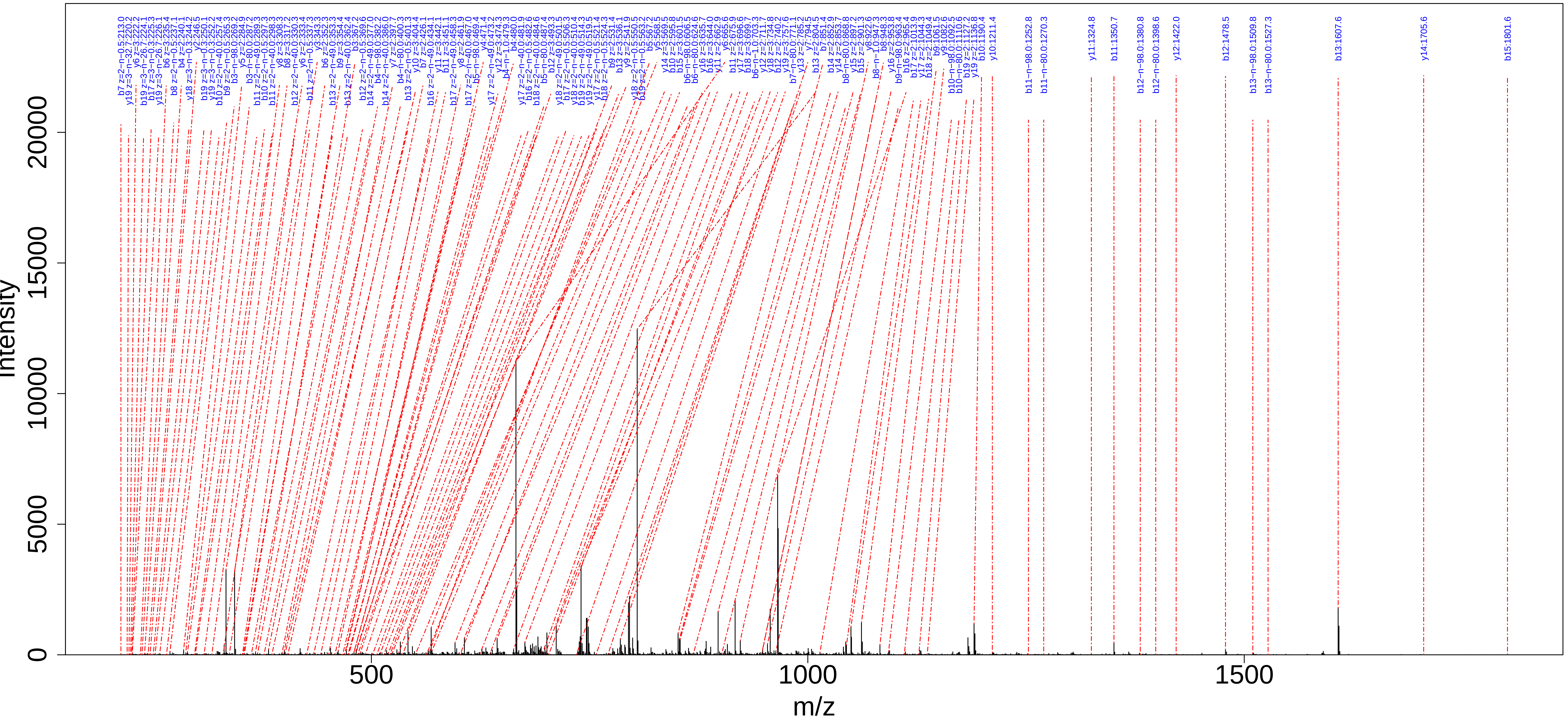

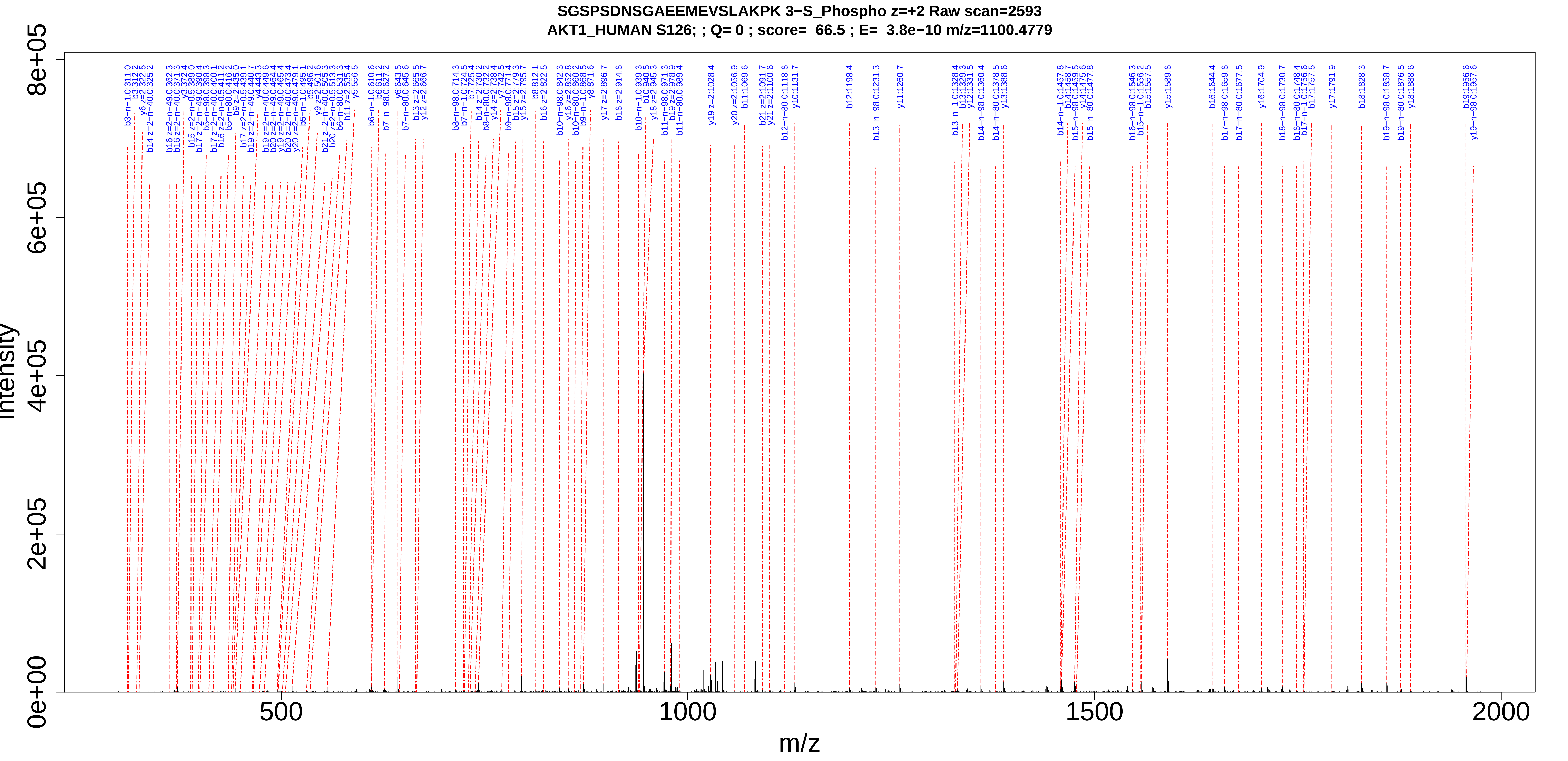

SGSPSDNSGAEEMEVSLAKPK 3−S_Phospho z=+2 Raw scan=2593
AKT1_HUMAN S126; ; Q= 0 ; score= 66.5 ; E= 3.8e−10 m/z=1100.4779
Intensity
0e+00
2e+05
4e+05
6e+05
8e+05
m/z
500
1000
1500
2000

SGSPSDNSGAEEMEVSLAKPK 3−S_Phospho z=+2 Raw scan=2629
AKT1_HUMAN S129; ; Q= 0 ; score= 66.5 ; E= 1.2e−10 m/z=1100.478

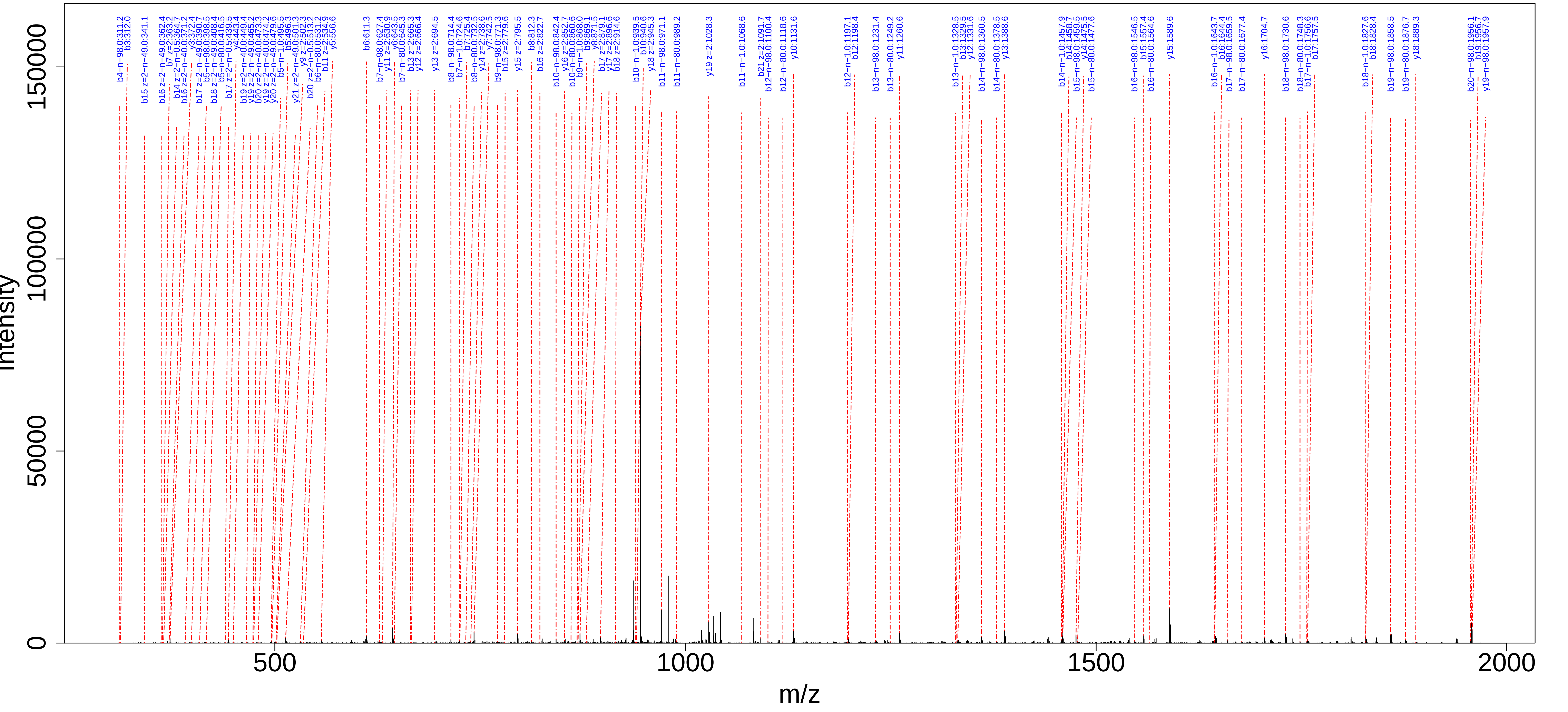

RANTLSHFPIECQEPPQPAR 4−T_Phospho z=+3 Raw scan=2656
TBCD1_HUMAN T596; ; Q= 0 ; score= 65.9 ; E= 1.7e−06 m/z=810.0464

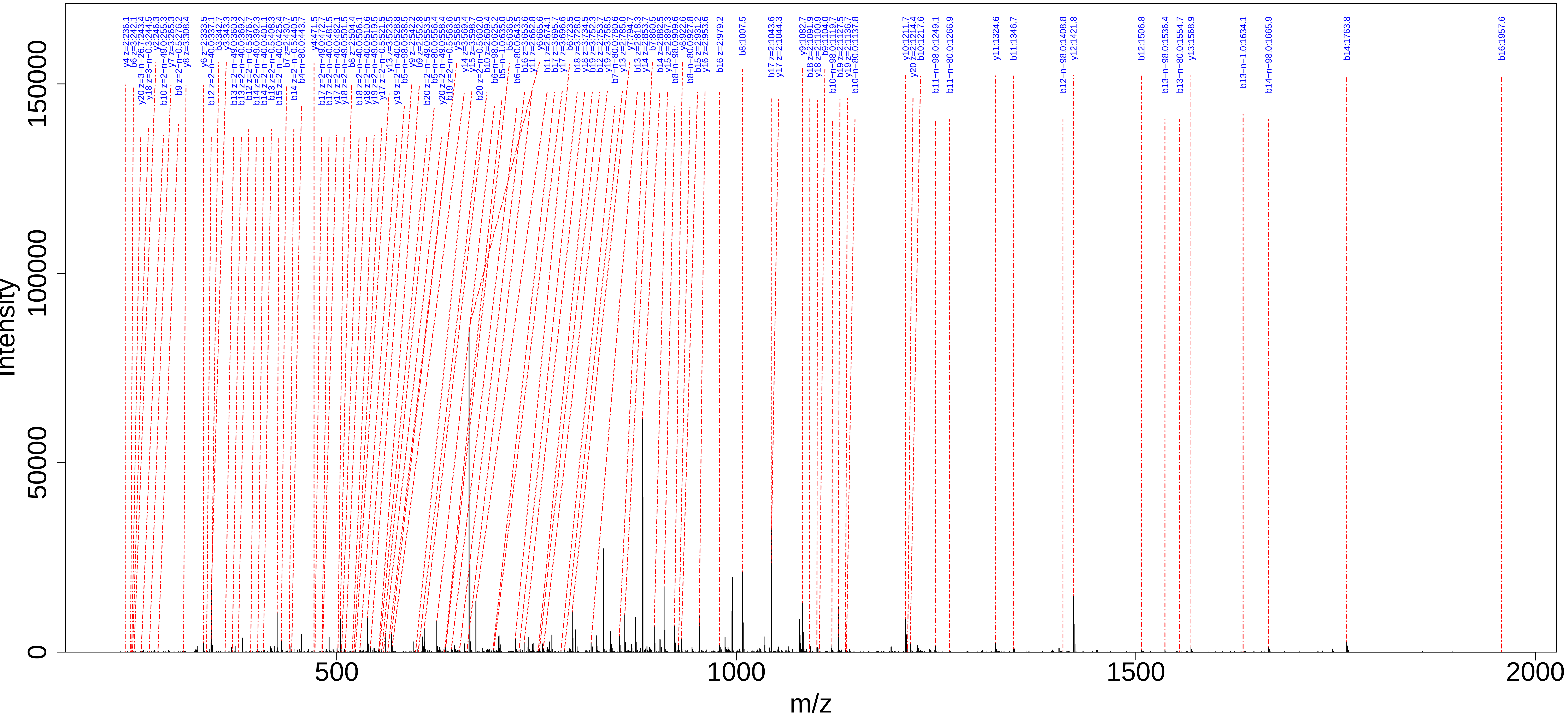

RANTLSHFPIECQEPPQPAR 4−T_Phospho z=+3 Raw scan=2677
TBCD1_HUMAN T596; ; Q= 0 ; score= 65.4 ; E= 2.5e−06 m/z=810.0469

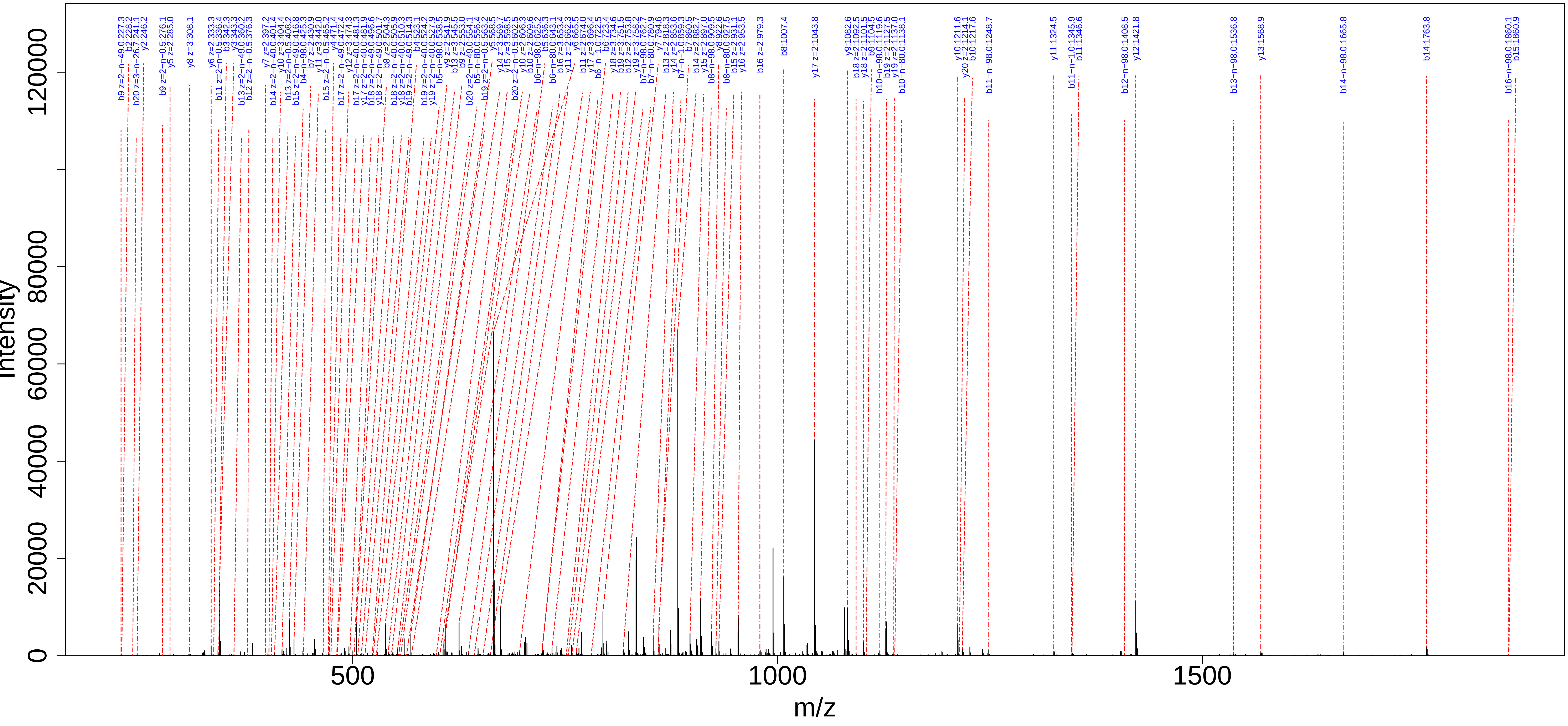

ANTLSHFPIECQEPPQPAR 3−T_Phospho z=+3 Raw scan=3036
TBCD1_HUMAN T596; ; Q= 0 ; score= 64.9 ; E= 7.9e−09 m/z=758.015

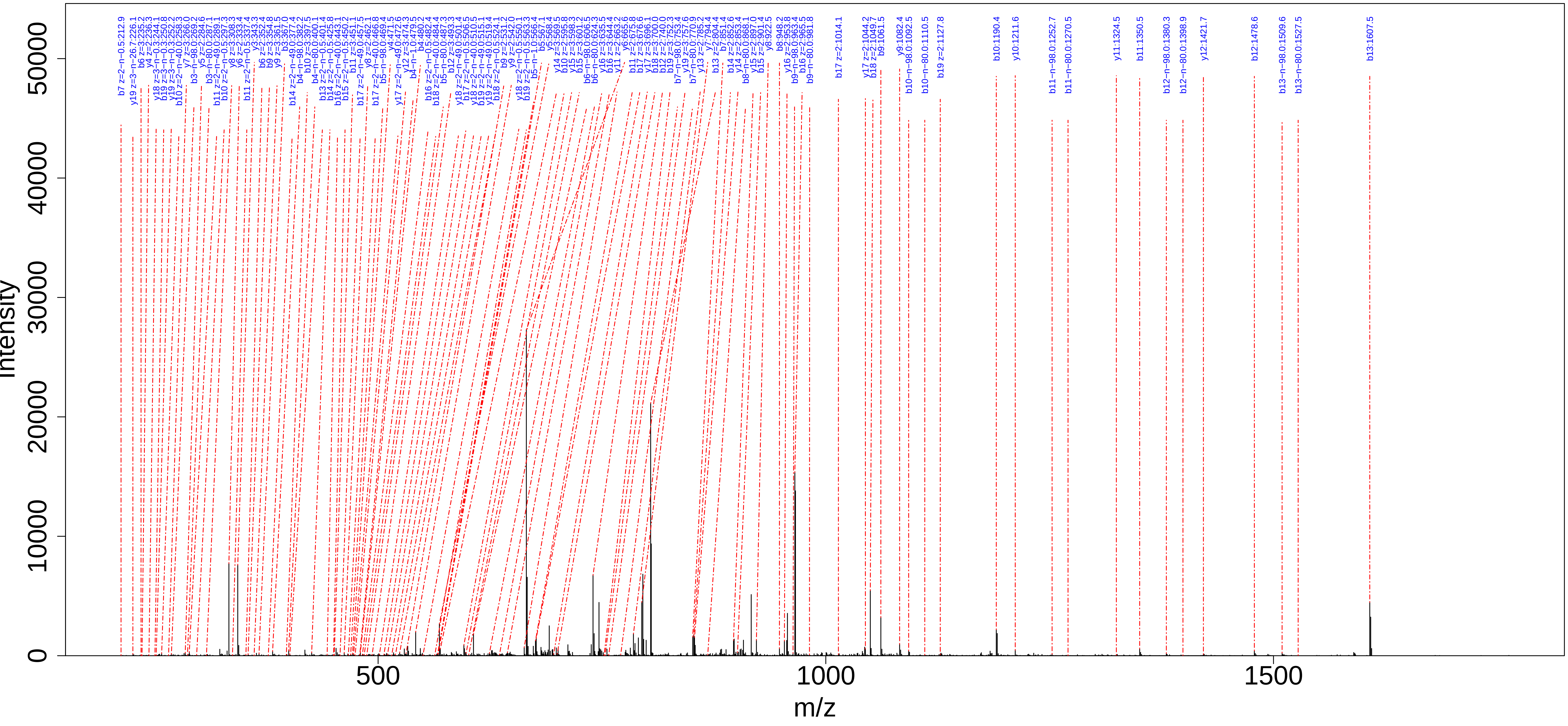

LLGSSEDLSSDSESHLPEEPAPLSPQQAFR 10−S_Phospho z=+3 Raw scan=3750
TBCD1_HUMAN S571; ; Q= 0 ; score= 64.7 ; E= 3.1e−06 m/z=1101.8425

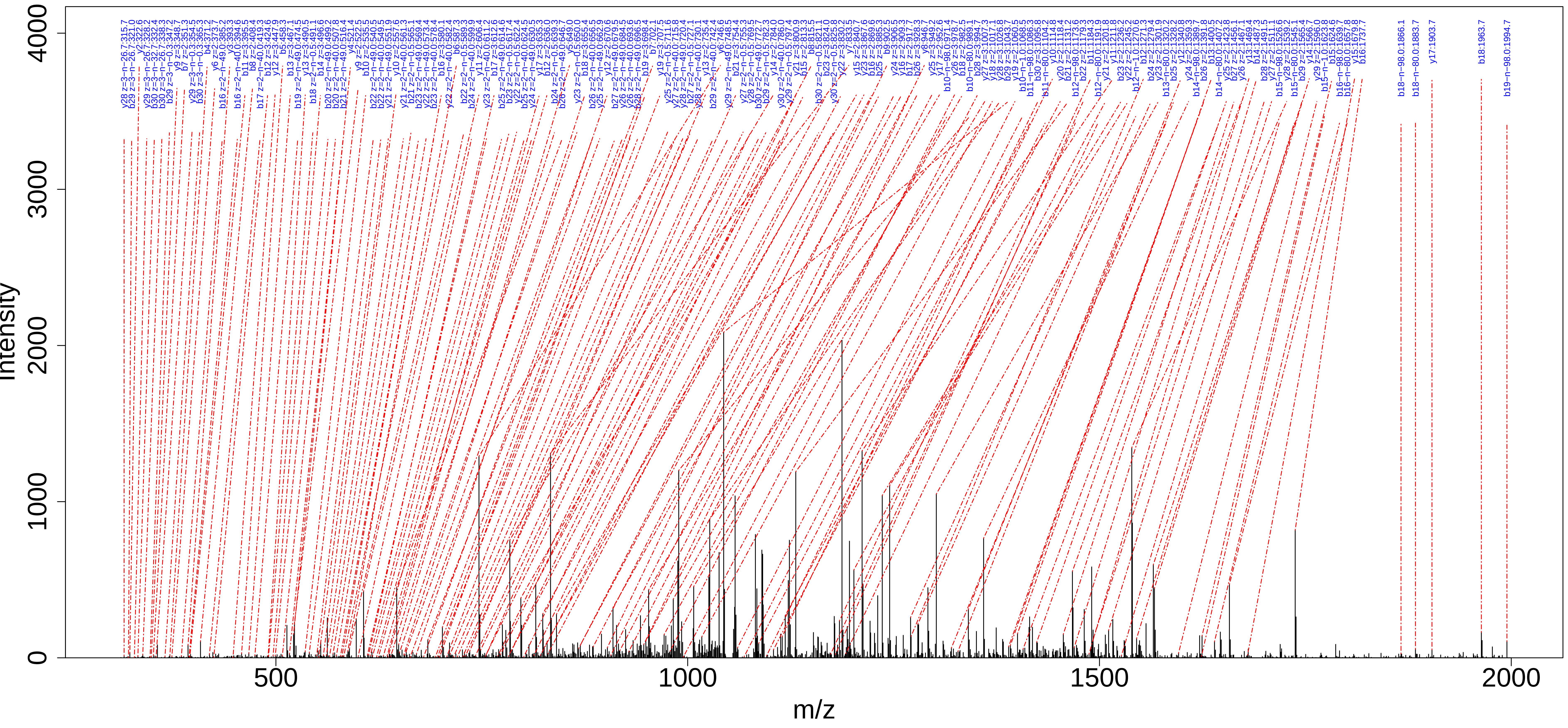

RANTLSHFPIECQEPPQPAR 4−T_Phospho z=+3 Raw scan=2663
TBCD1_HUMAN T596; ; Q= 0 ; score= 63.7 ; E= 1.3e−06 m/z=810.0467

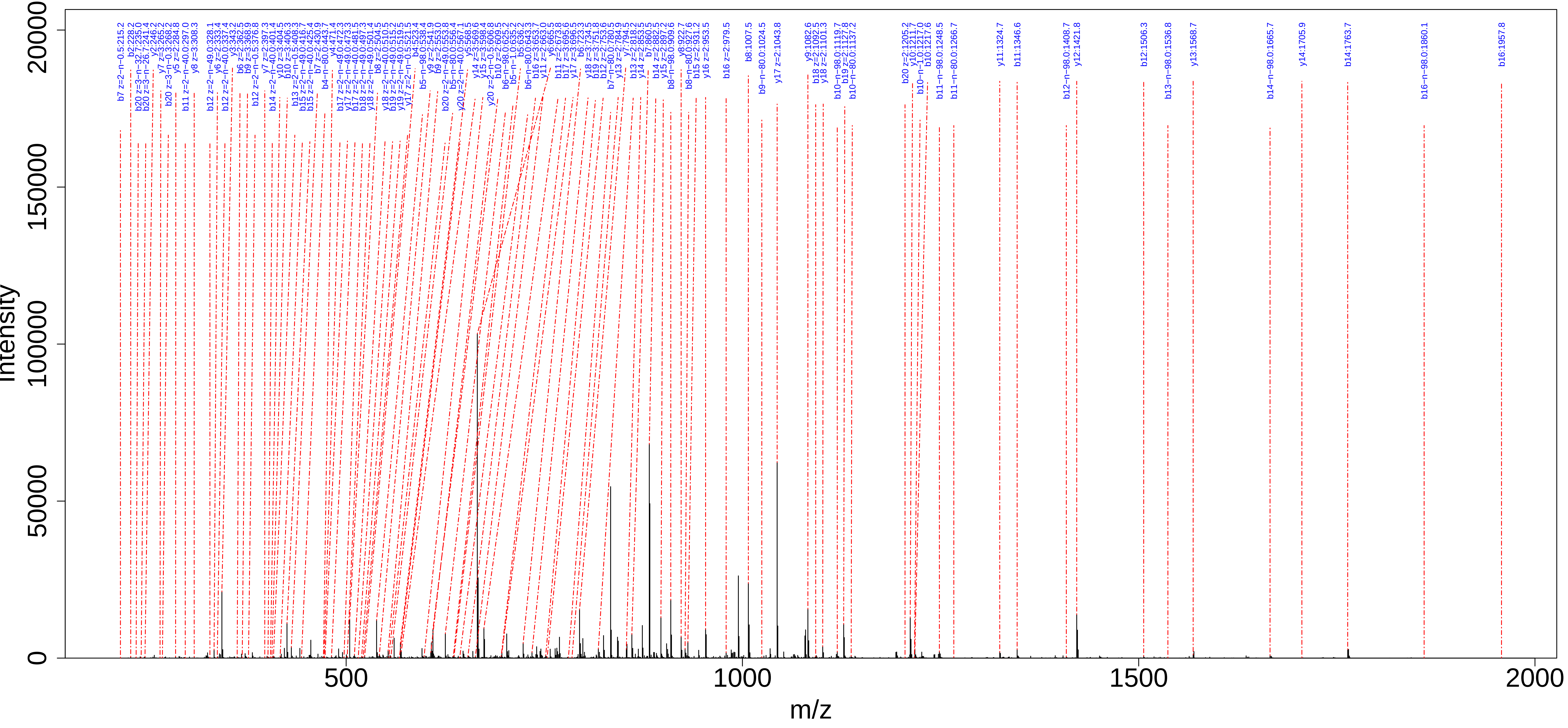

SGSPSDNSGAEEMEVSLAKPK 3−S_Phospho z=+2 Raw scan=2600
AKT1_HUMAN S126; ; Q= 0 ; score= 63.4 ; E= 2.1e−09 m/z=1100.4772

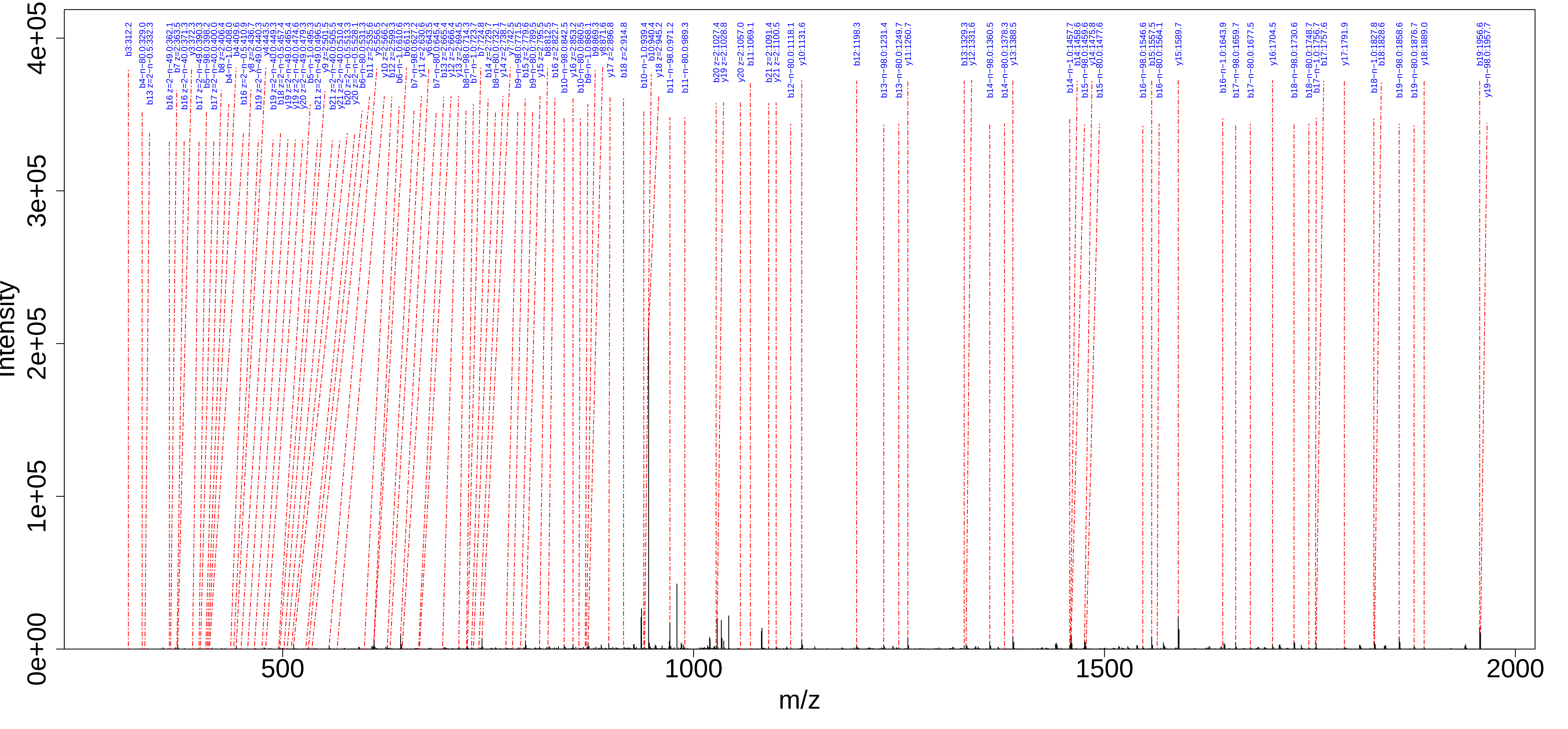

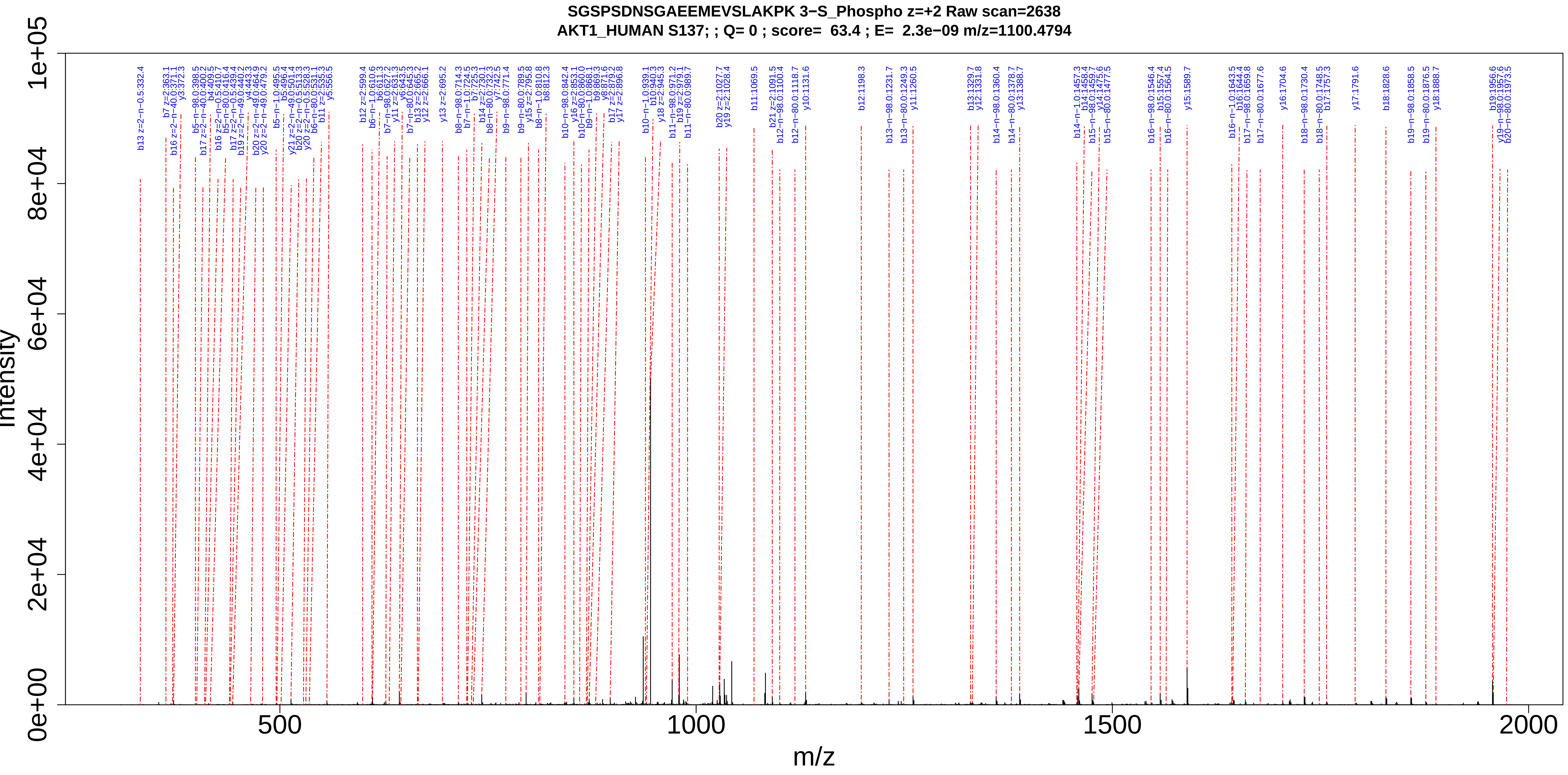
SGSPSDNSGAEEMEVSLAKPK 3−S_Phospho z=+2 Raw scan=2638
AKT1_HUMAN S137; ; Q= 0 ; score= 63.4 ; E= 2.3e−09 m/z=1100.4794
Intensity
m/z
0e+00
2e+04
4e+04
6e+04
8e+04
1e+05
500
1000
1500
2000

SGSPSDNSGAEEMEVSLAKPK 3−S_Phospho z=+2 Raw scan=2621
AKT1_HUMAN S137; ; Q= 0 ; score= 63.4 ; E= 6e−08 m/z=1100.478

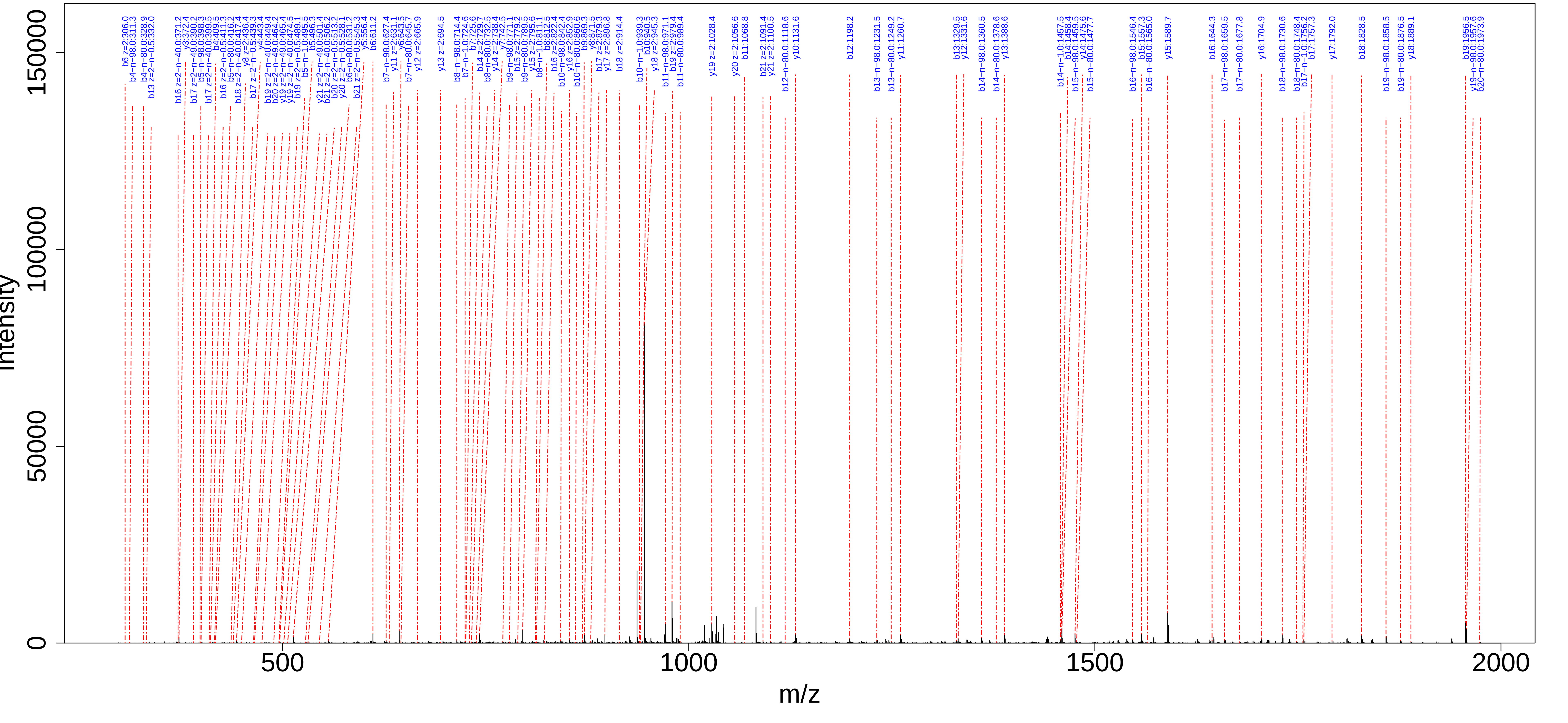

RANTLSHFPIECQEPPQPAR 4−T_Phospho z=+3 Raw scan=2692
TBCD1_HUMAN T596; ; Q= 0 ; score= 62.3 ; E= 2.5e−06 m/z=810.0468

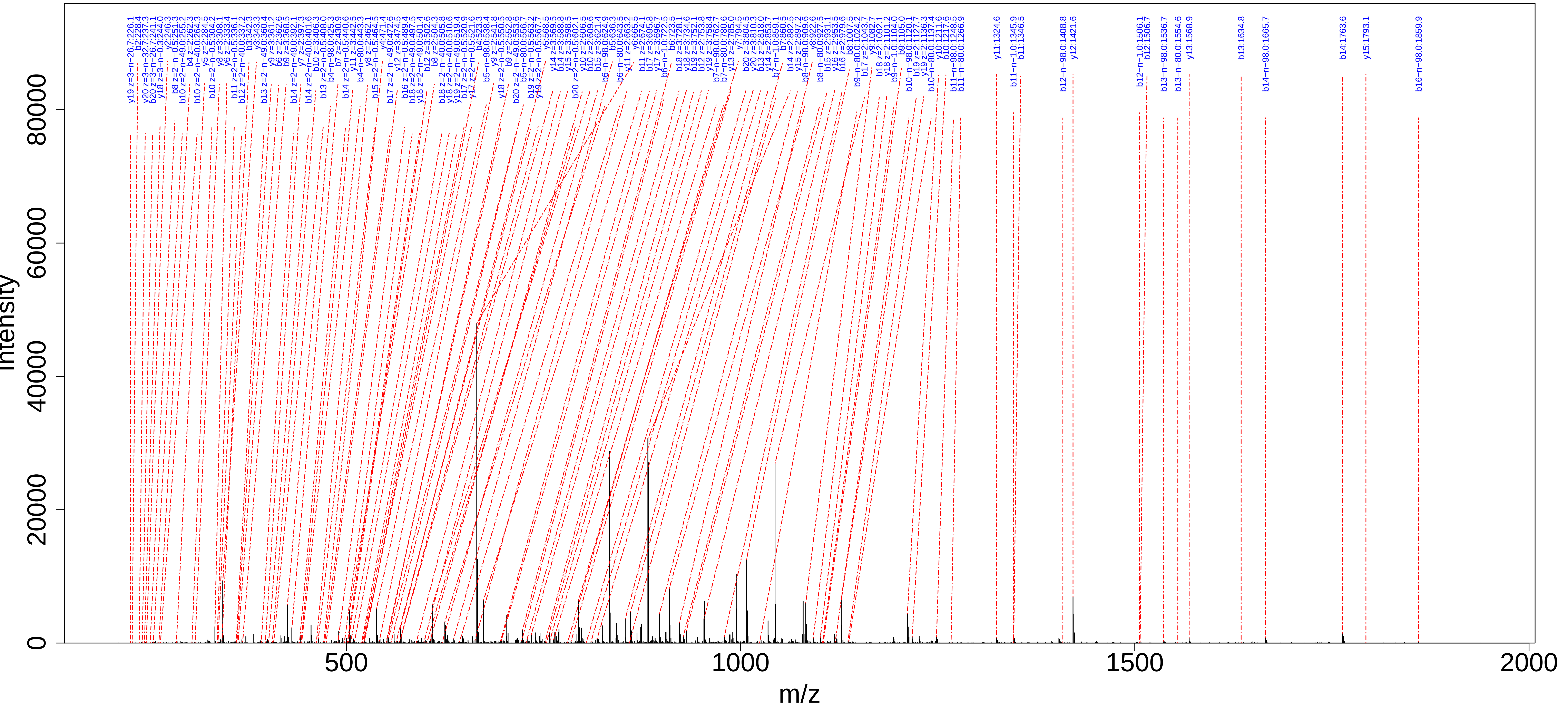

ANTLSHFPIECQEPPQPAR 3−T_Phospho z=+3 Raw scan=3029
TBCD1_HUMAN T596; ; Q= 0 ; score= 61.9 ; E= 2.5e−08 m/z=758.0131

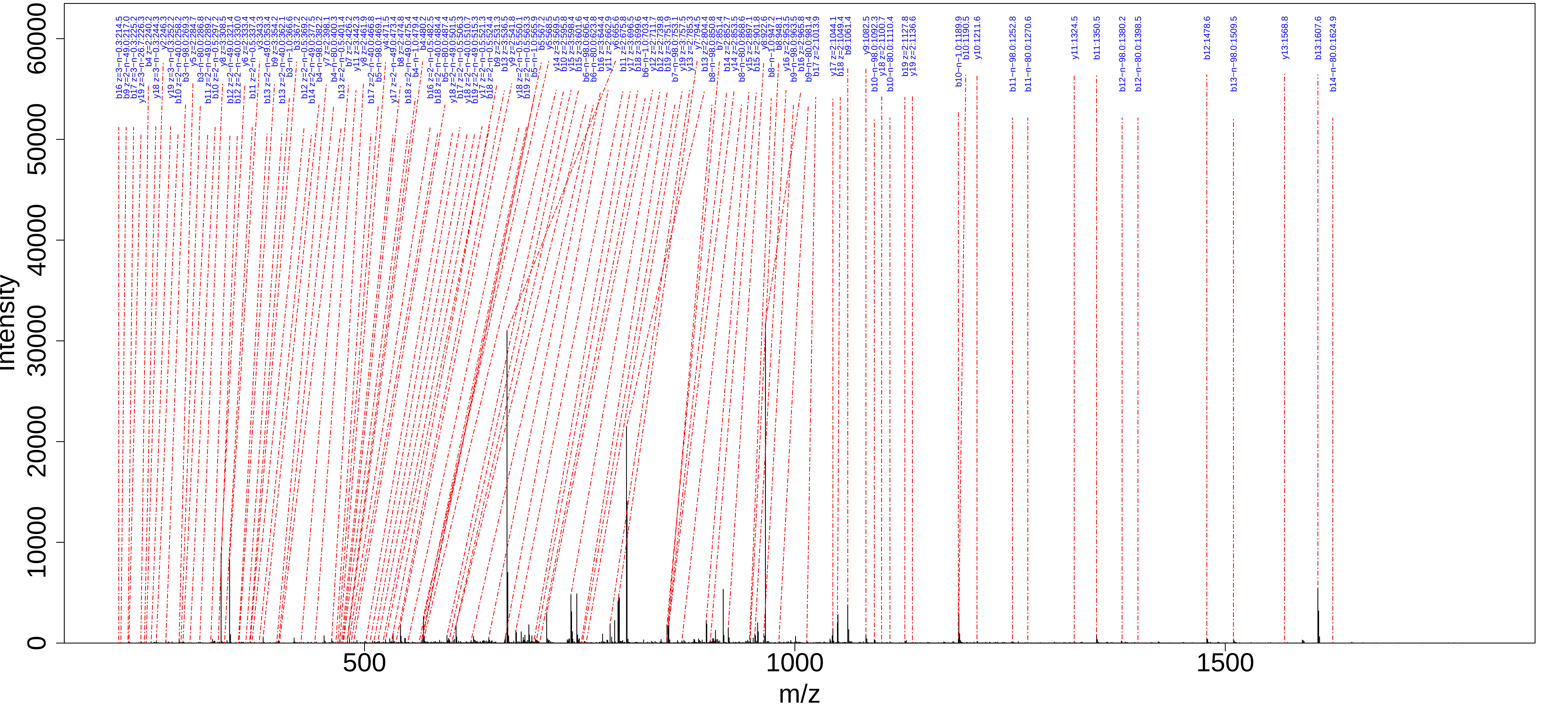

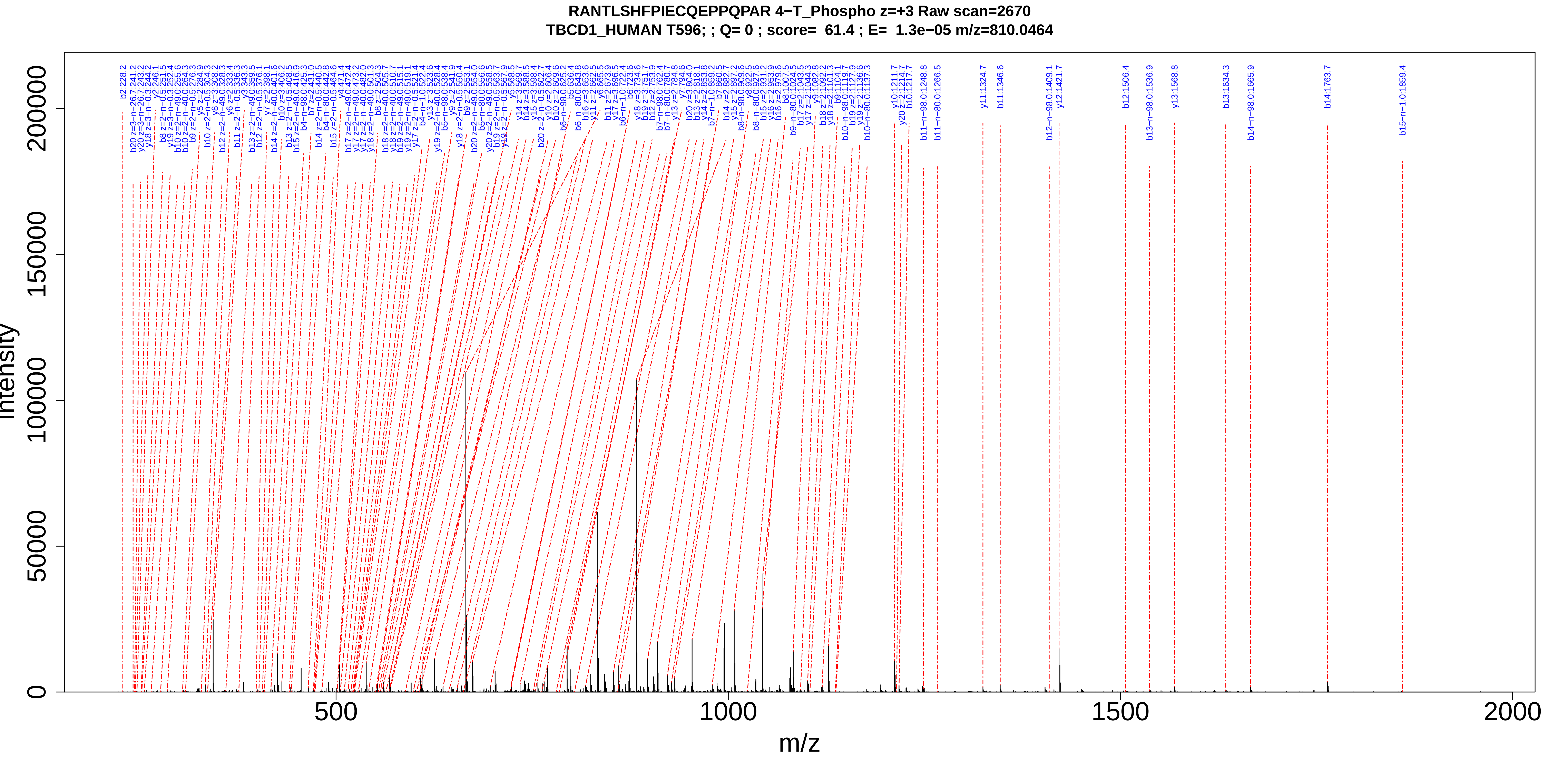
RANTLSHFPIECQEPPQPAR 4−T_Phospho z=+3 Raw scan=2670
TBCD1_HUMAN T596; ; Q= 0 ; score= 61.4 ; E= 1.3e−05 m/z=810.0464
Intensity
0
50000
100000
150000
200000
m/z
500
1000
1500
2000

ANTLSHFPIECQEPPQPAR 3−T_Phospho z=+3 Raw scan=3044
TBCD1_HUMAN T596; ; Q= 0 ; score= 61.1 ; E= 1e−05 m/z=758.0163

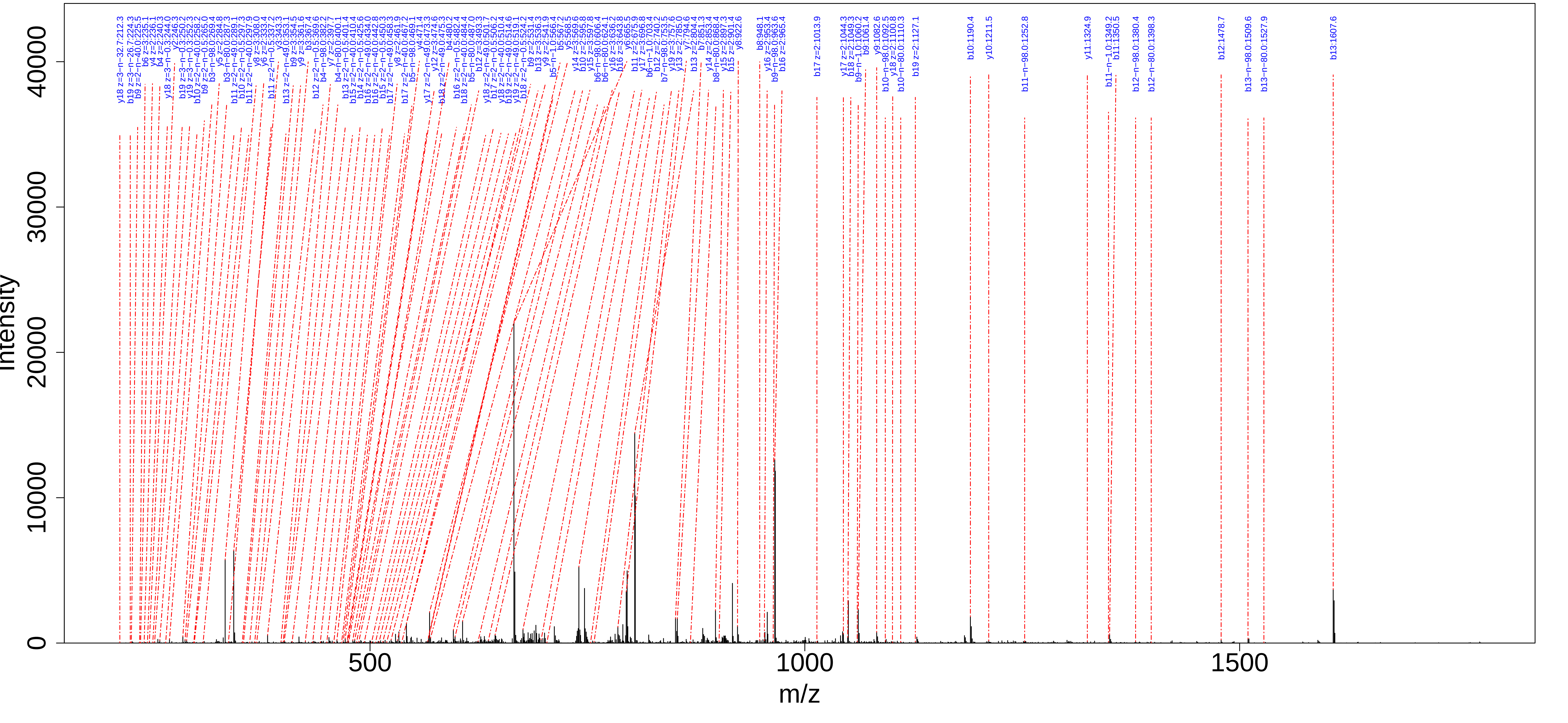

RANTLSHFPIECQEPPQPAR 4−T_Phospho z=+3 Raw scan=2684
TBCD1_HUMAN T596; ; Q= 0 ; score= 60.6 ; E= 3e−08 m/z=810.0477

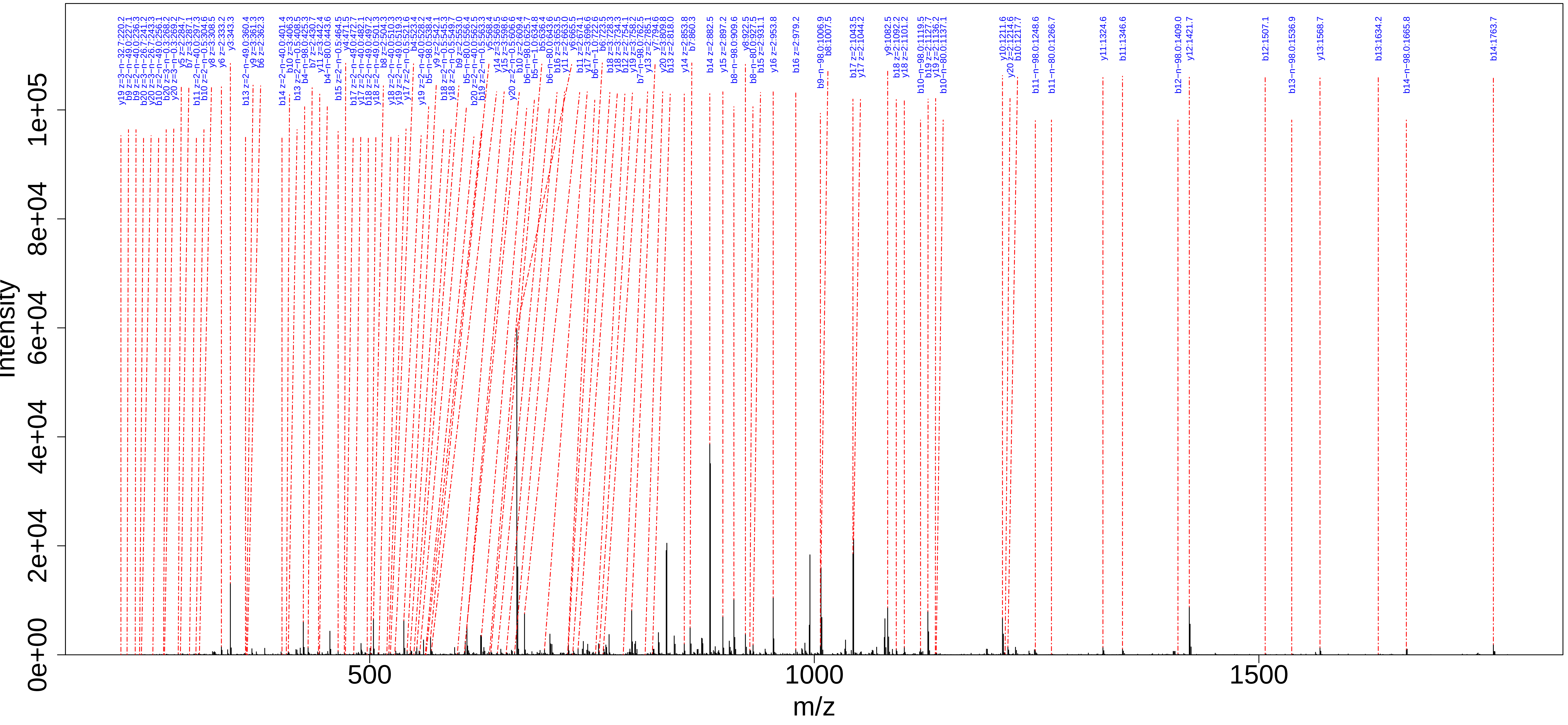

ANTLSHFPIECQEPPQPAR 3−T_Phospho z=+2 Raw scan=3002
TBCD1_HUMAN S598; ; Q= 0 ; score= 60.1 ; E= 1.3e−08 m/z=1136.5137

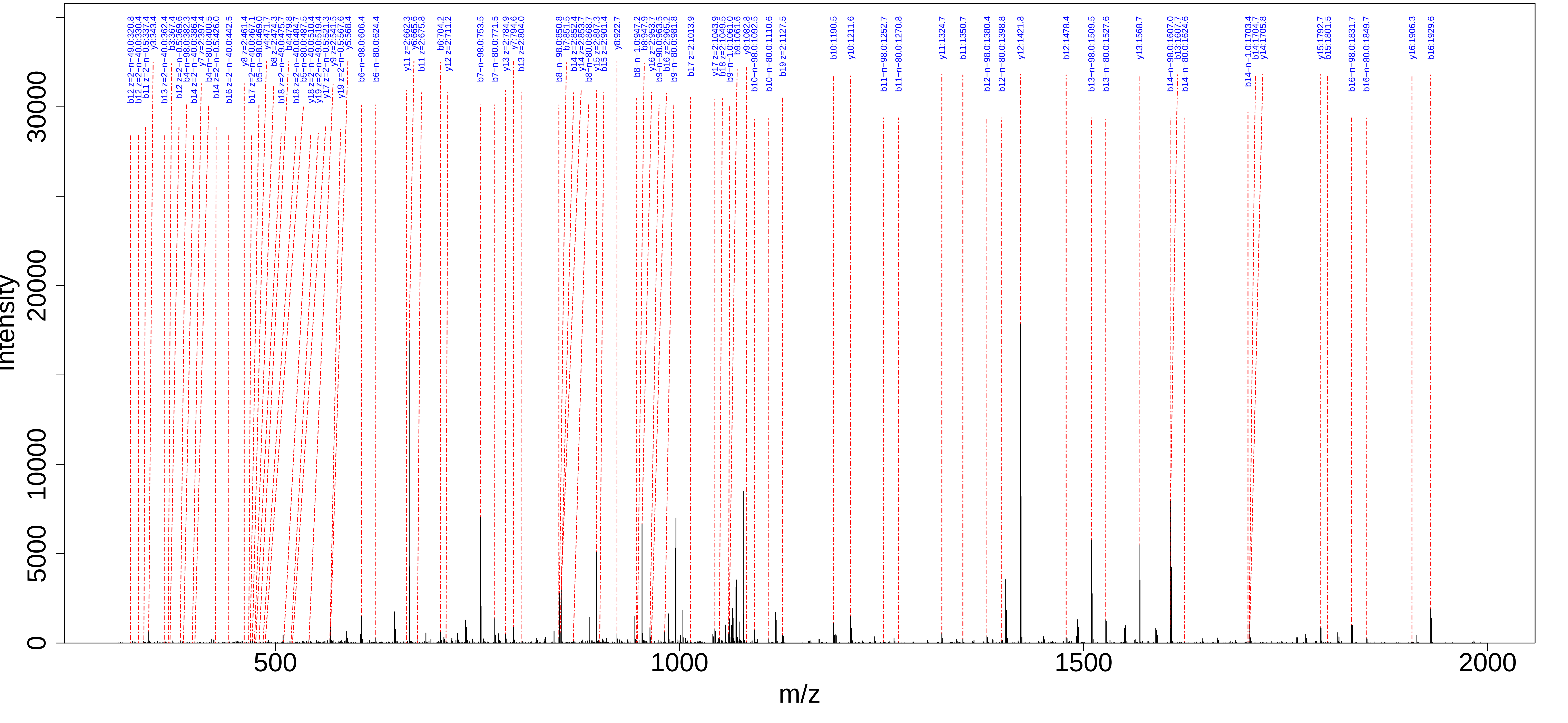

SGSPSDNSGAEEMEVSLAKPK 1−S_Phospho z=+2 Raw scan=2643
AKT1_HUMAN S126; ; Q= 0 ; score= 58.8 ; E= 1.7e−09 m/z=1100.4812

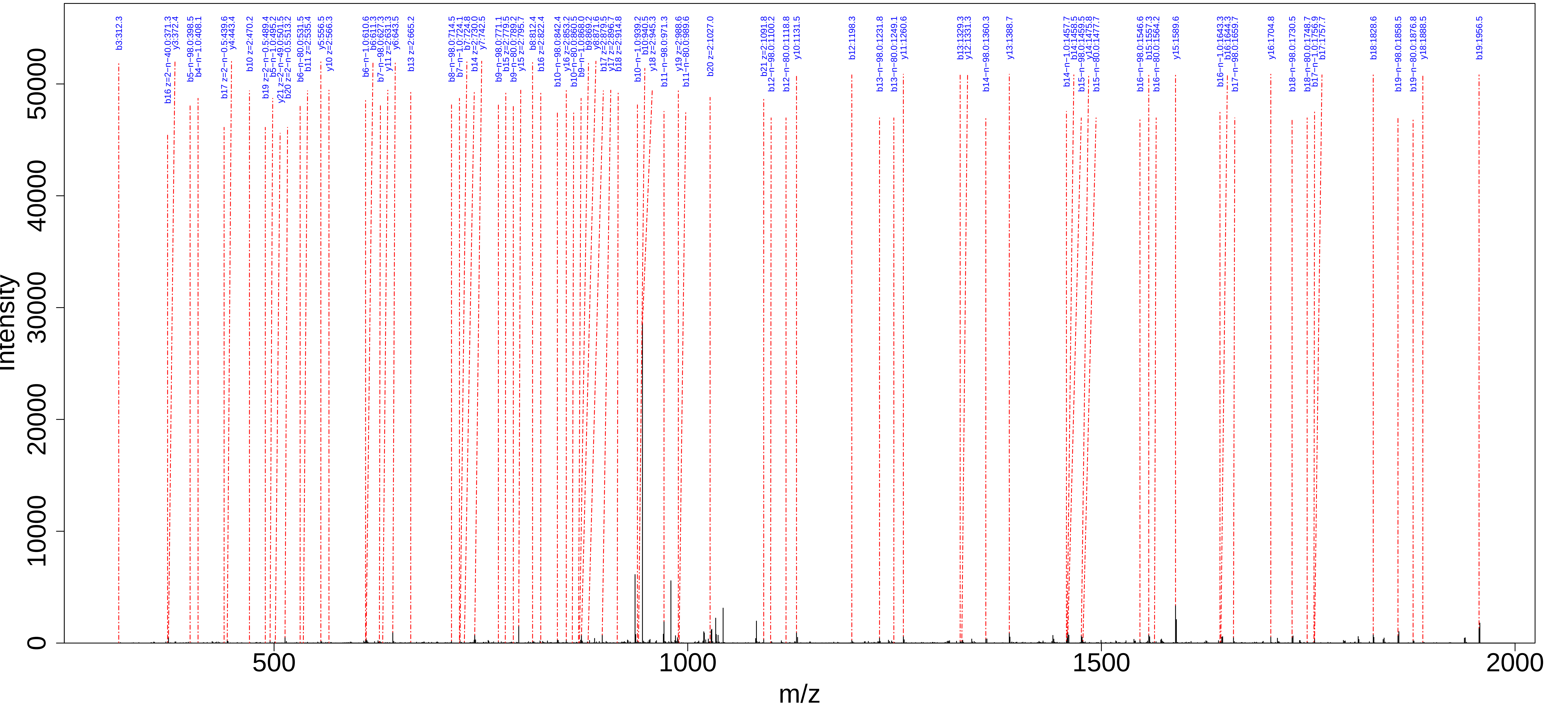

ANTLSHFPIECQEPPQPAR 3−T_Phospho z=+3 Raw scan=3014

TBCD1_HUMAN T596; ; Q= 0 ; score= 58.3 ; E= 9.8e−06 m/z=758.0123

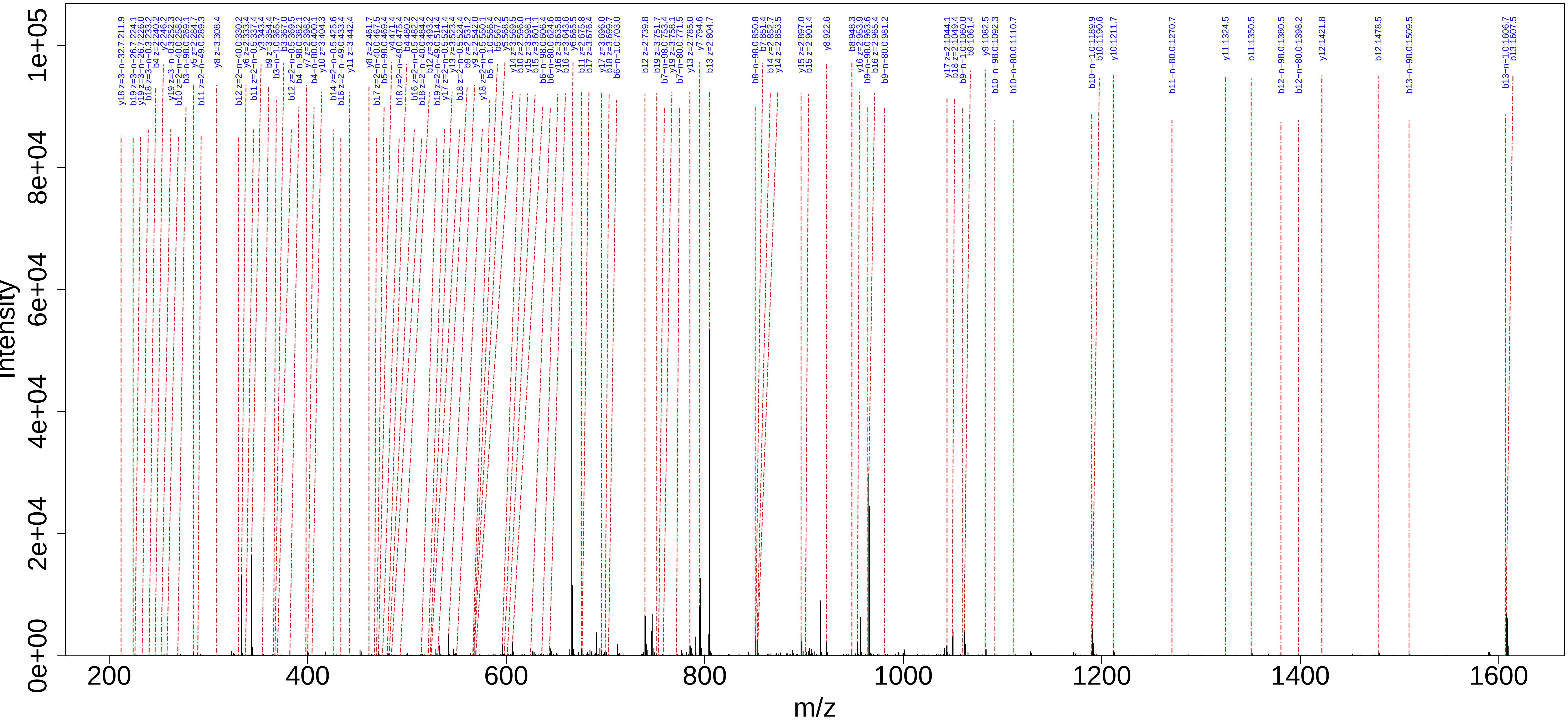

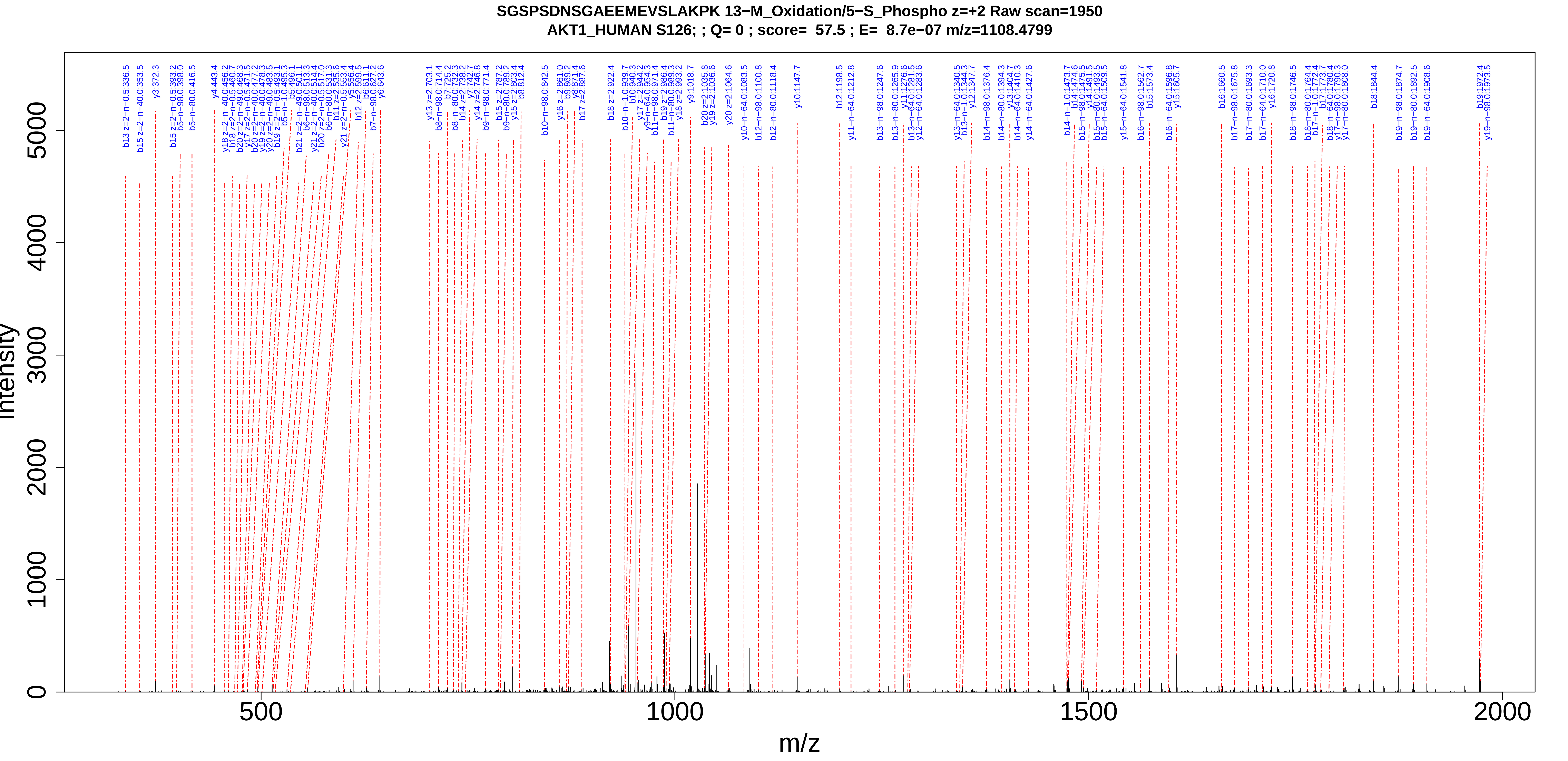
SGSPSDNSGAEEMEVSLAKPK 13−M_Oxidation/5−S_Phospho z=+2 Raw scan=1950
AKT1_HUMAN S126; ; Q= 0 ; score= 57.5 ; E= 8.7e−07 m/z=1108.4799
Intensity
m/z

RANTLSHFPIECQEPPQPAR 4−T_Phospho z=+3 Raw scan=2701
TBCD1_HUMAN T596; ; Q= 0 ; score= 56.2 ; E= 9.1e−05 m/z=810.0464

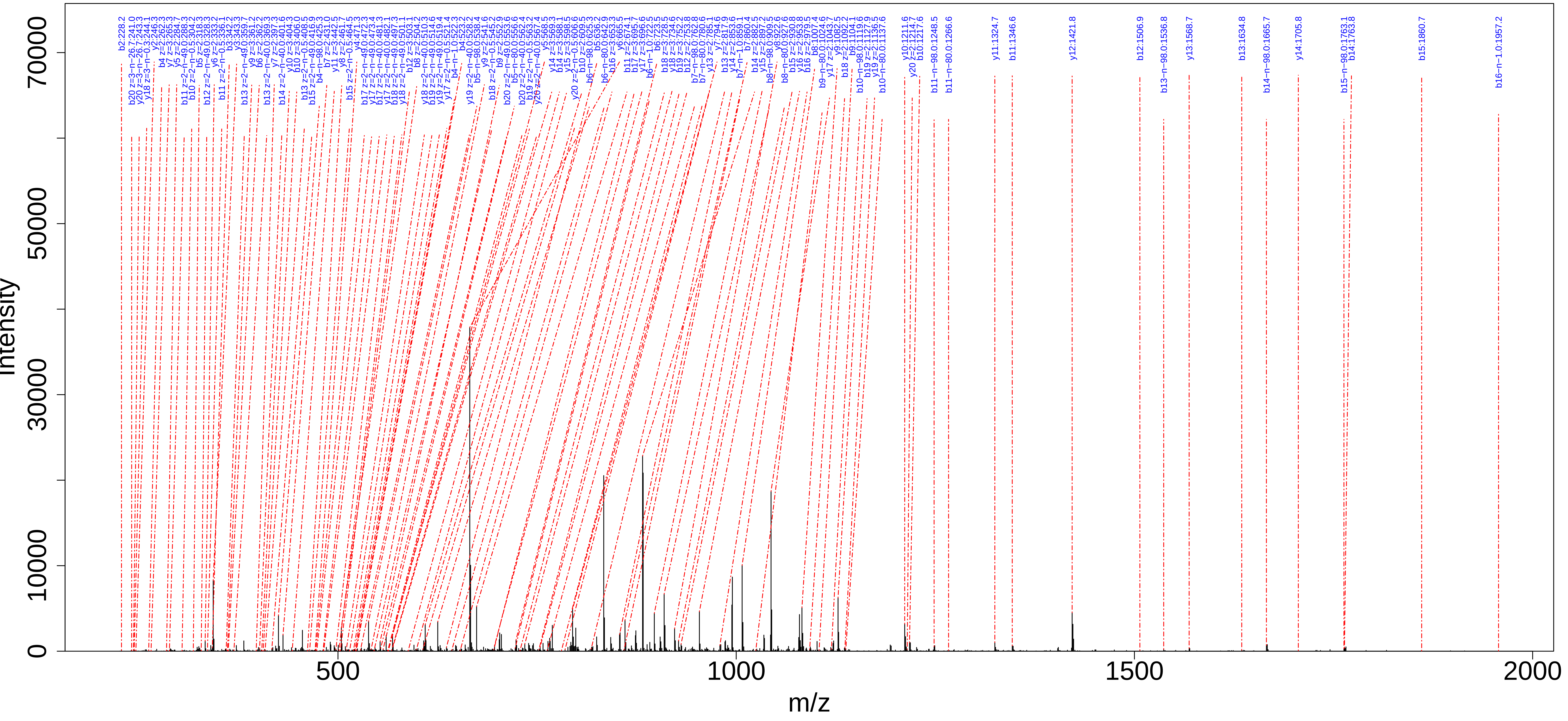

SGSPSDNSGAEEMEVSLAKPK 13−M_Oxidation/3−S_Phospho z=+2 Raw scan=1943
AKT1_HUMAN S126; ; Q= 0 ; score= 56.1 ; E= 8.1e−09 m/z=1108.4762

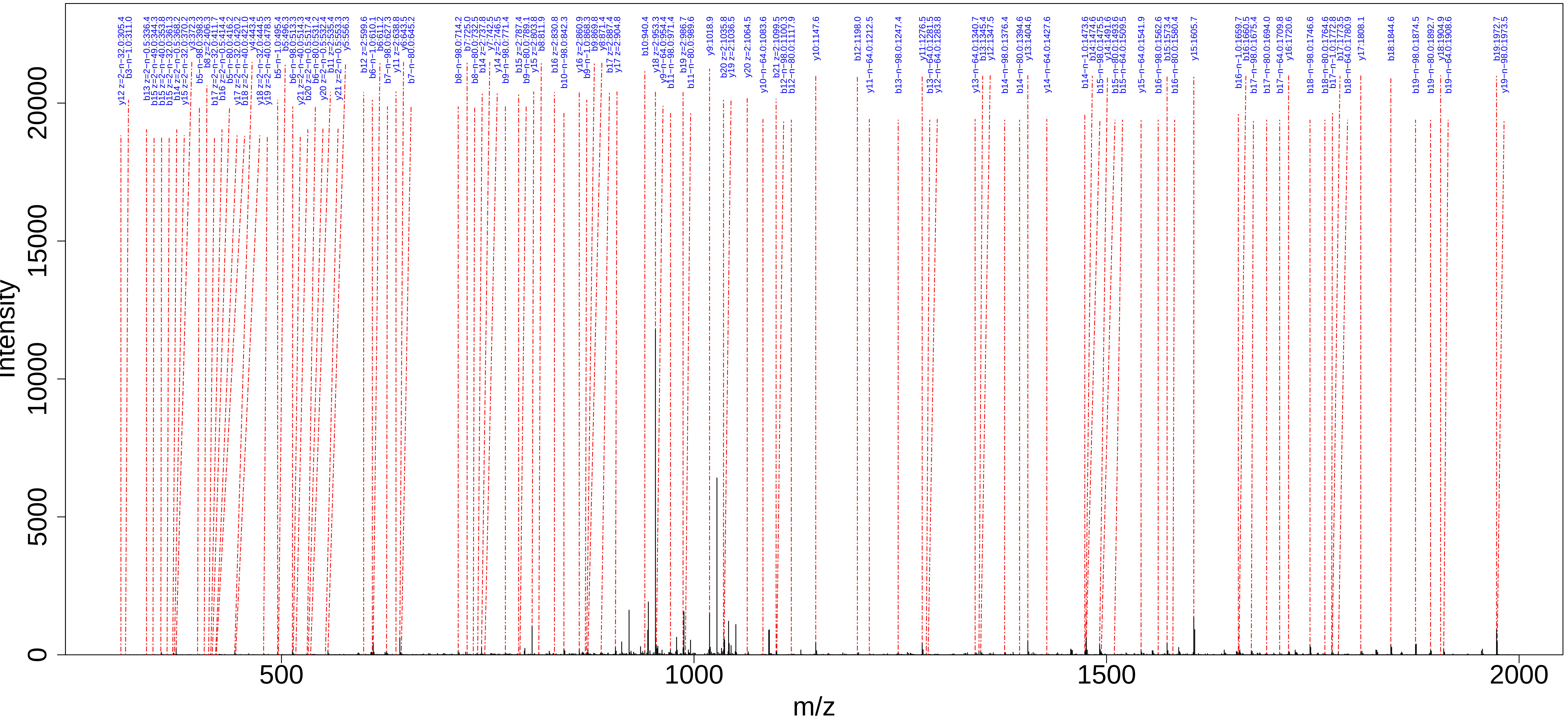

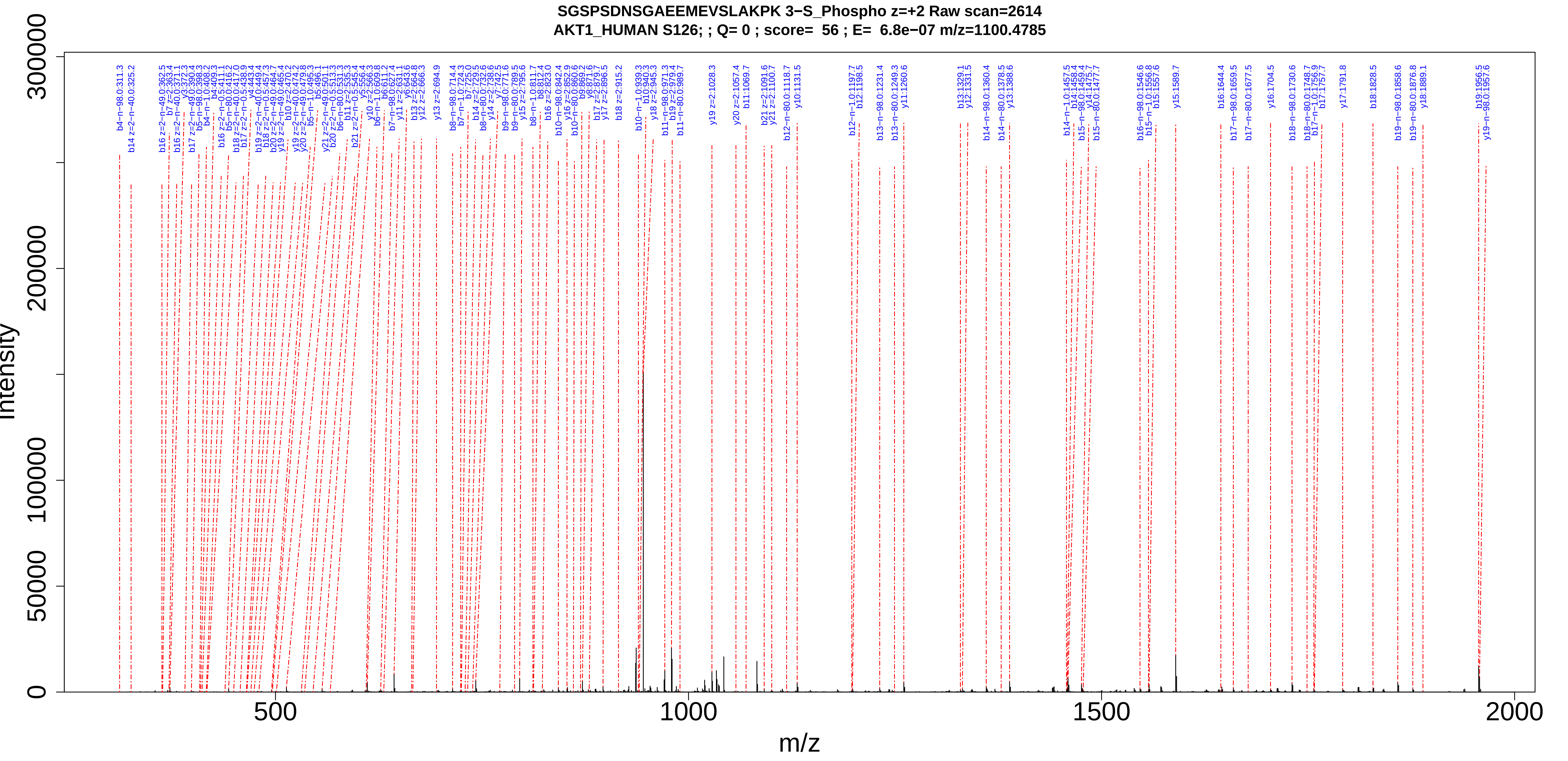
SGSPSDNSGAEEMEVSLAKPK 3−S_Phospho z=+2 Raw scan=2614
AKT1_HUMAN S126; ; Q= 0 ; score= 56 ; E= 6.8e−07 m/z=1100.4785
Intensity
0
50000
100000
200000
300000
m/z
500
1000
1500
2000

RANTLSHFPIECQEPPQPAR 4−T_Phospho z=+3 Raw scan=2652
TBCD1_HUMAN T596; ; Q= 0 ; score= 53.8 ; E= 0.00068 m/z=810.0464

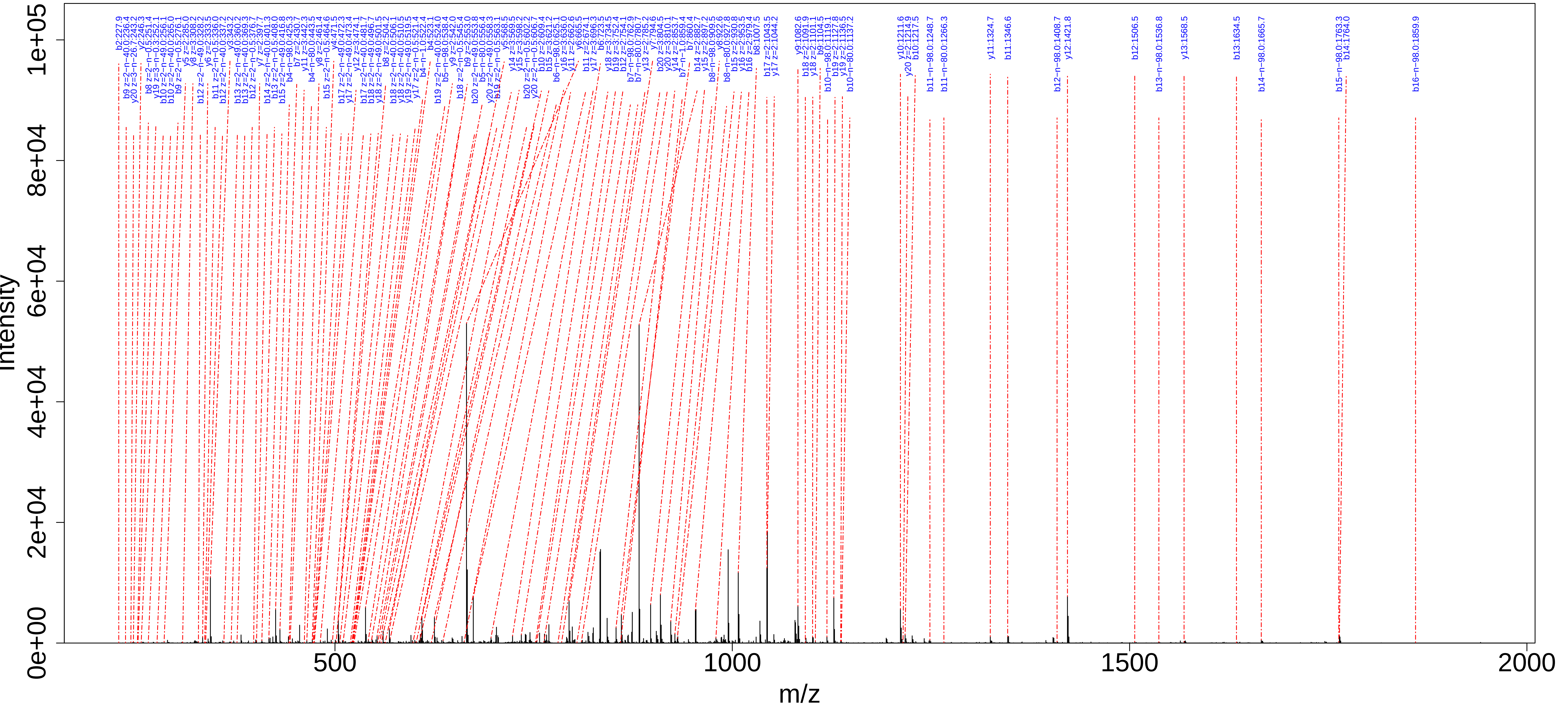

SGSPSDNSGAEEMEVSLAKPK 13−M_Oxidation/3−S_Phospho z=+2 Raw scan=1959
AKT1_HUMAN S124; ; Q= 0 ; score= 53.7 ; E= 2e−06 m/z=1108.4774

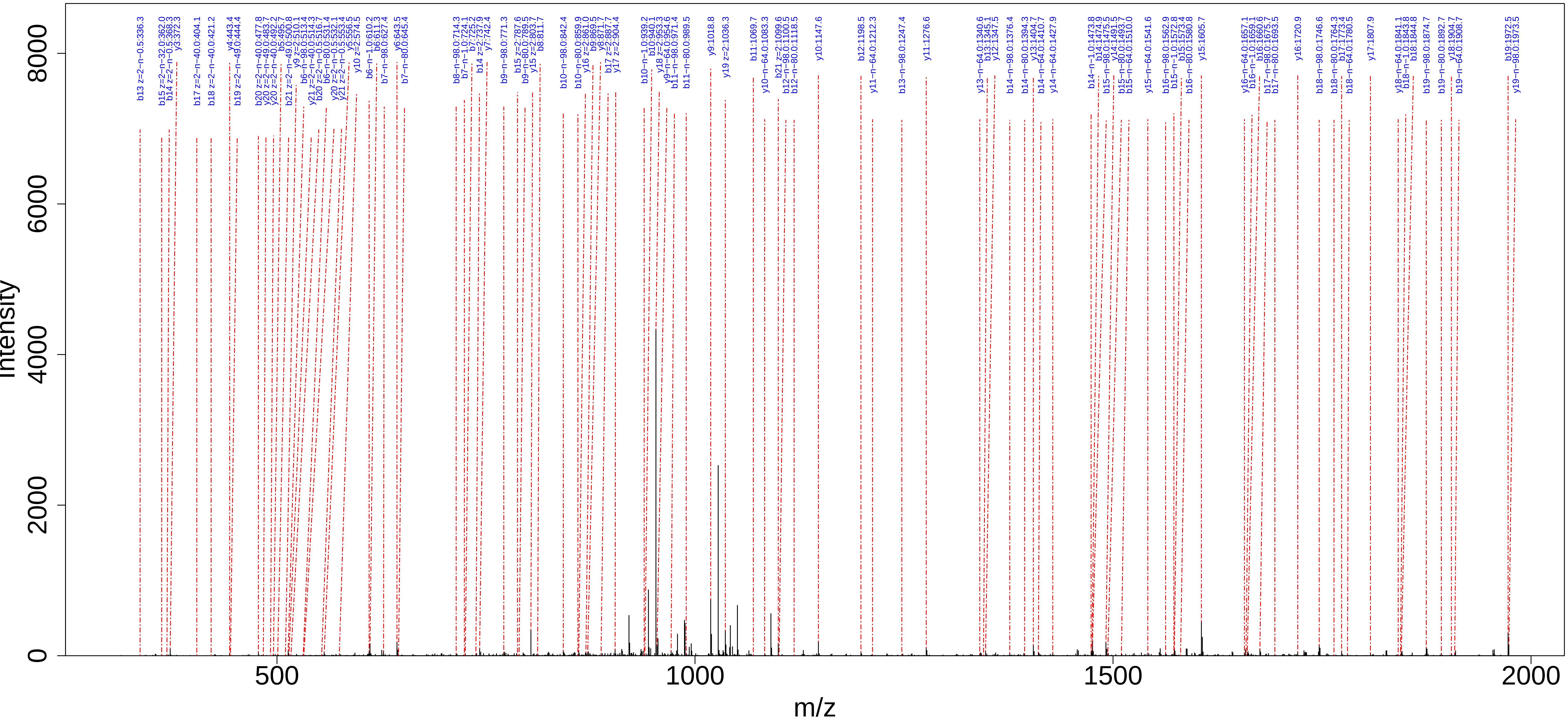

HRVTMNEFEYLK 4−T_Phospho z=+3 Raw scan=2977
AKT1_HUMAN T146; ; Q= 0 ; score= 51.8 ; E= 3e−05 m/z=549.5864

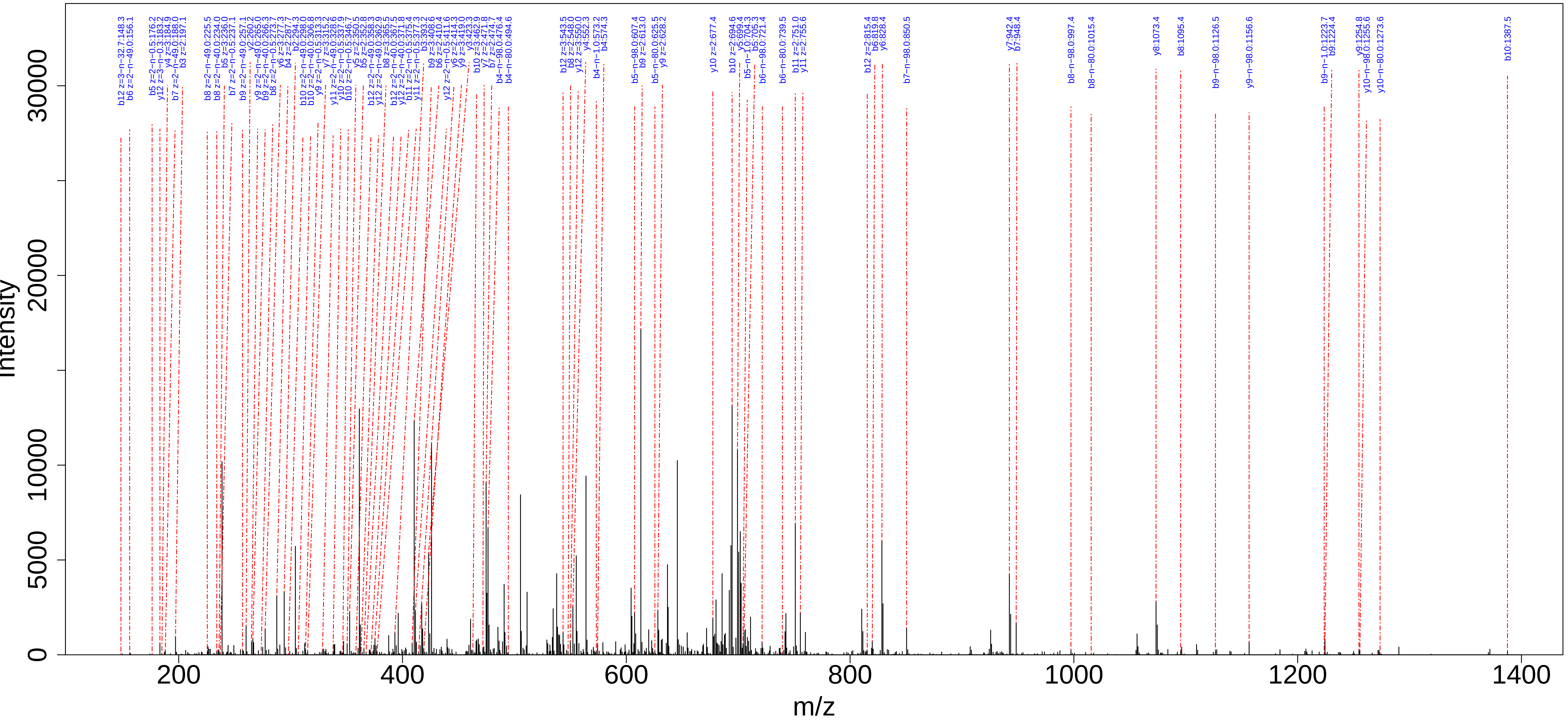

HRVTMNEFEYLK 4−T_Phospho z=+3 Raw scan=2991
AKT1_HUMAN T146; ; Q= 0 ; score= 45.2 ; E= 0.003 m/z=549.5852

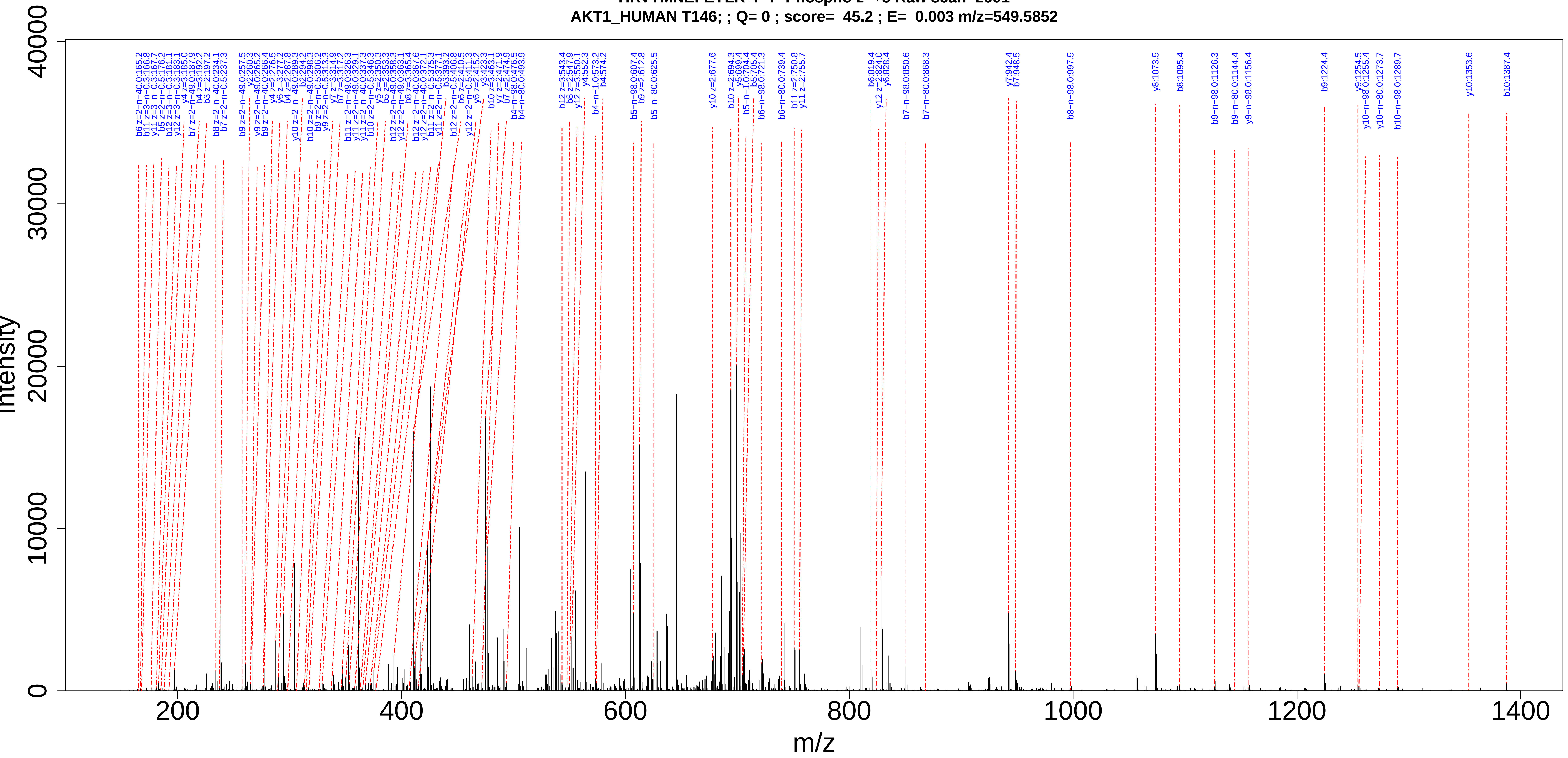

SFSQPGLR 3−S_Phospho z=+2 Raw scan=2147
TBCD1_HUMAN S237; ; Q= 0 ; score= 44.6 ; E= 5.2e−05 m/z=486.2201

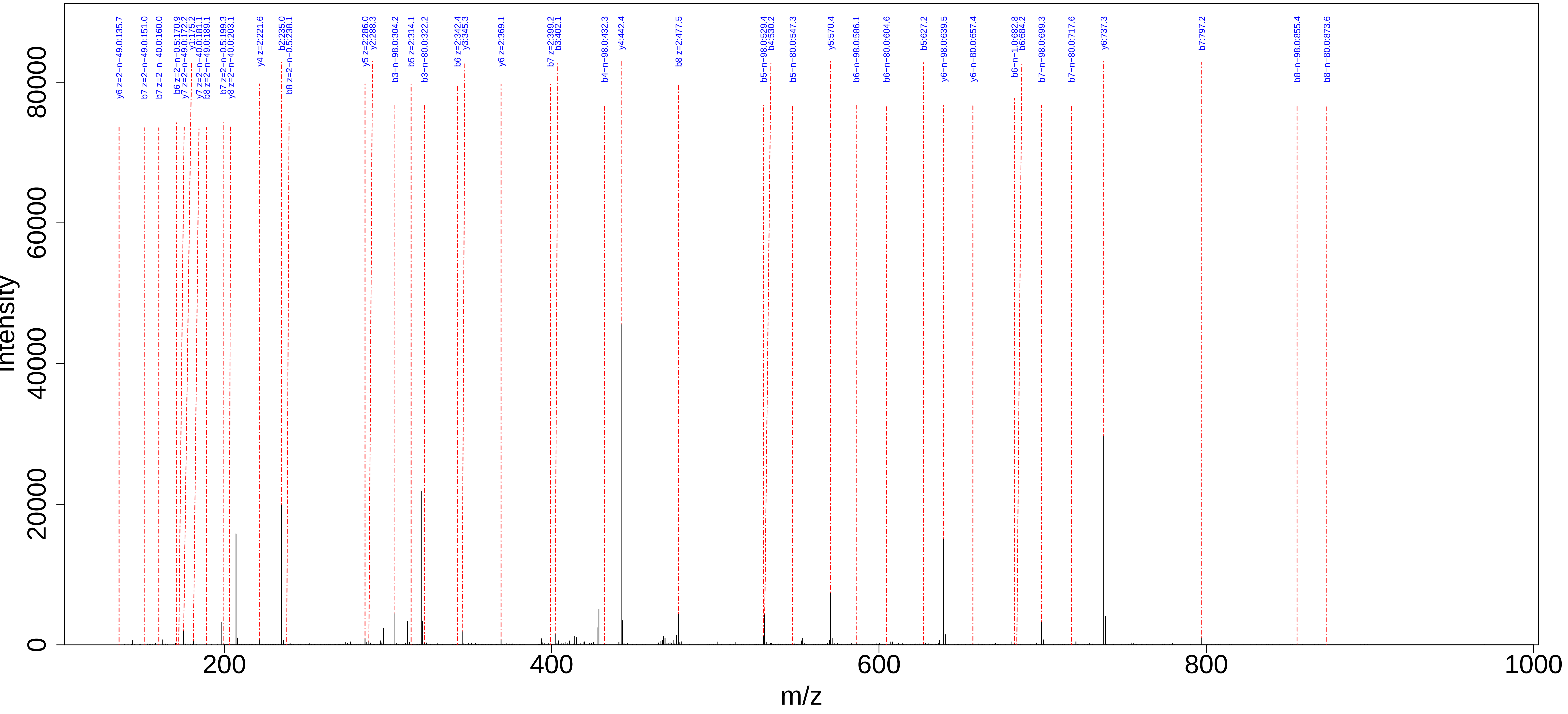

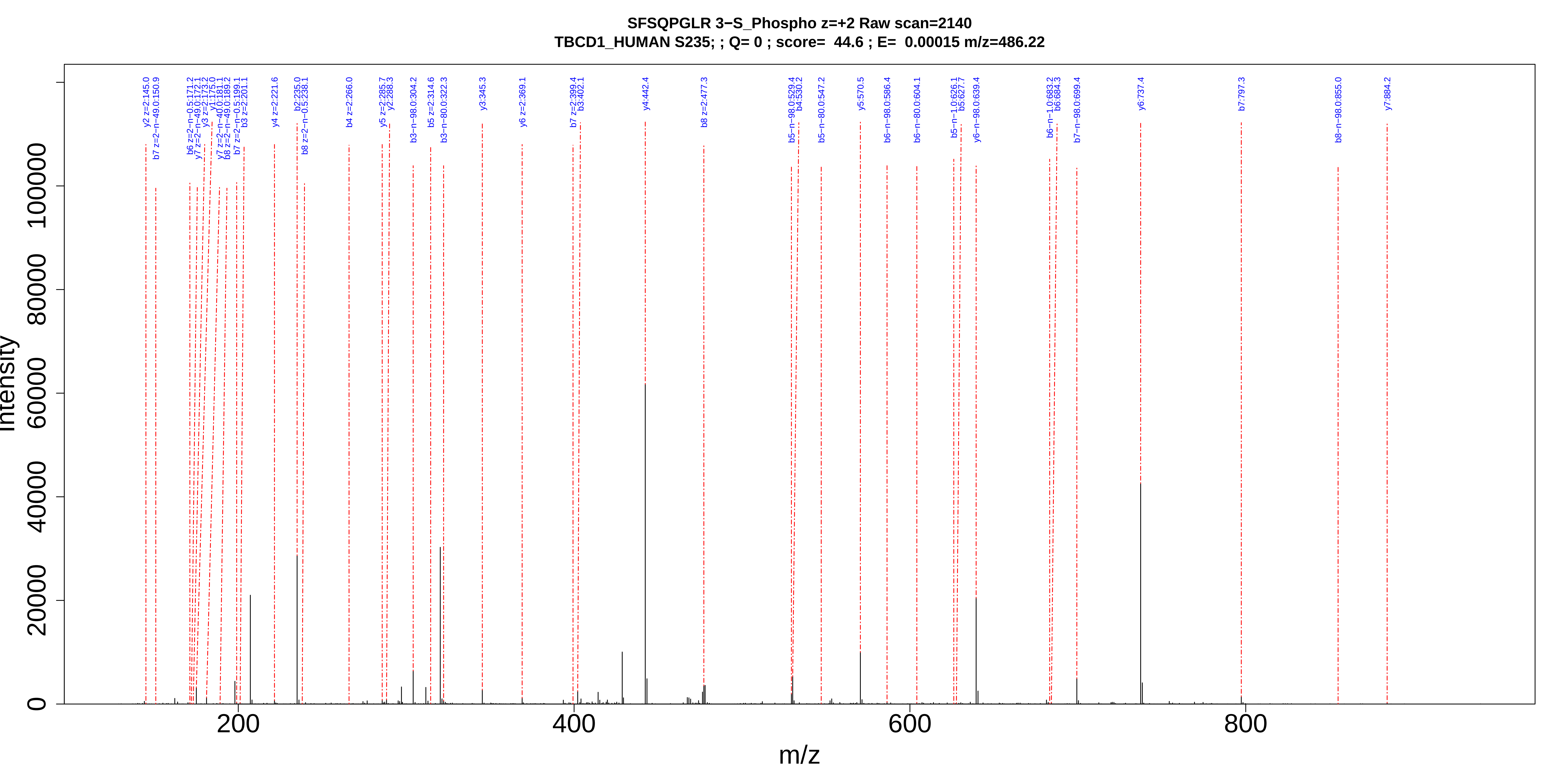
SFSQPGLR 3−S_Phospho z=+2 Raw scan=2140
TBCD1_HUMAN S235; ; Q= 0 ; score= 44.6 ; E= 0.00015 m/z=486.22
Intensity
m/z

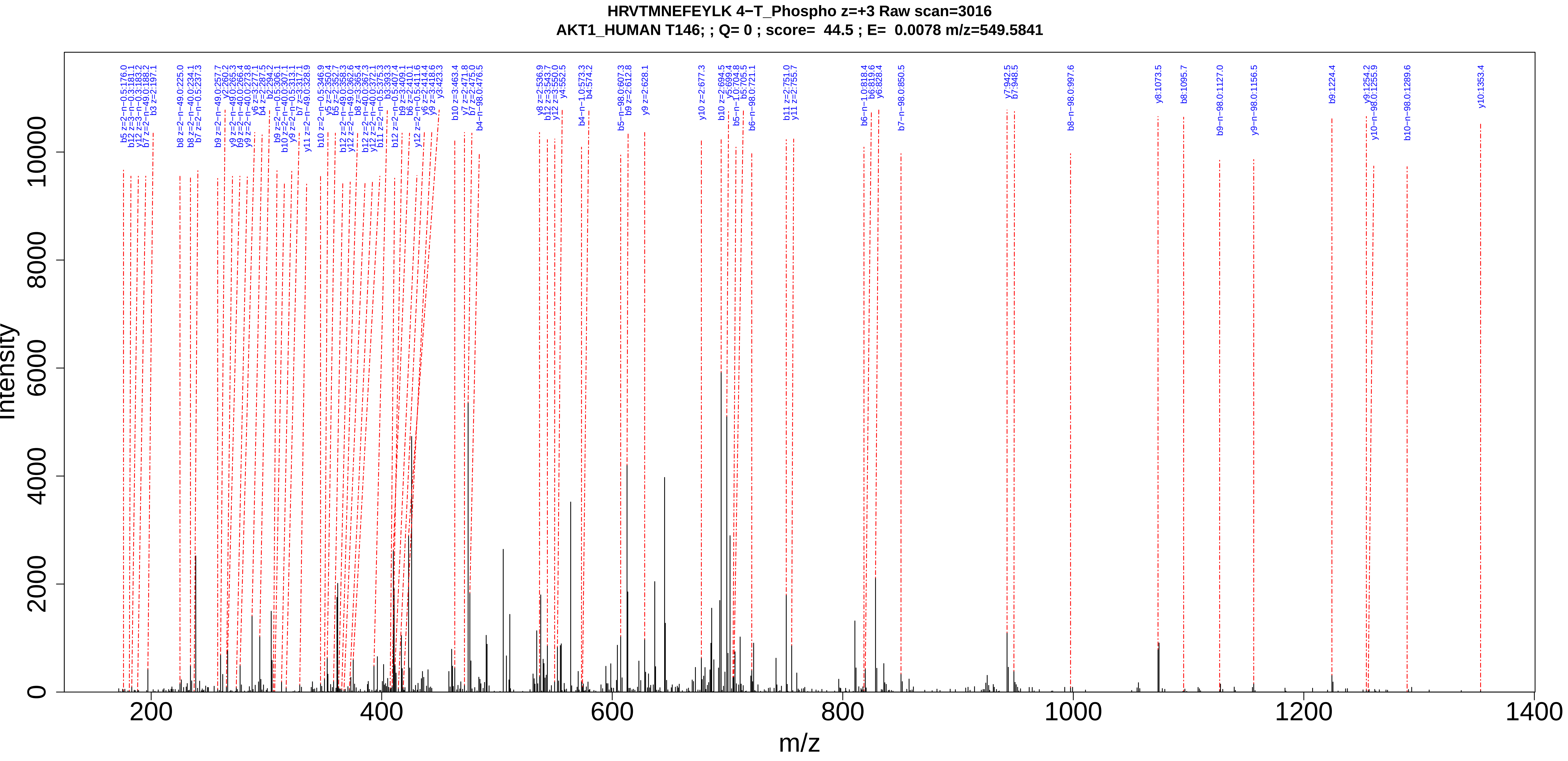
HRVTMNEFEYLK 4−T_Phospho z=+3 Raw scan=3016
AKT1_HUMAN T146; ; Q= 0 ; score= 44.5 ; E= 0.0078 m/z=549.5841
Intensity
0
2000
4000
6000
8000
10000
m/z
200
400
600
800
1000
1200
1400

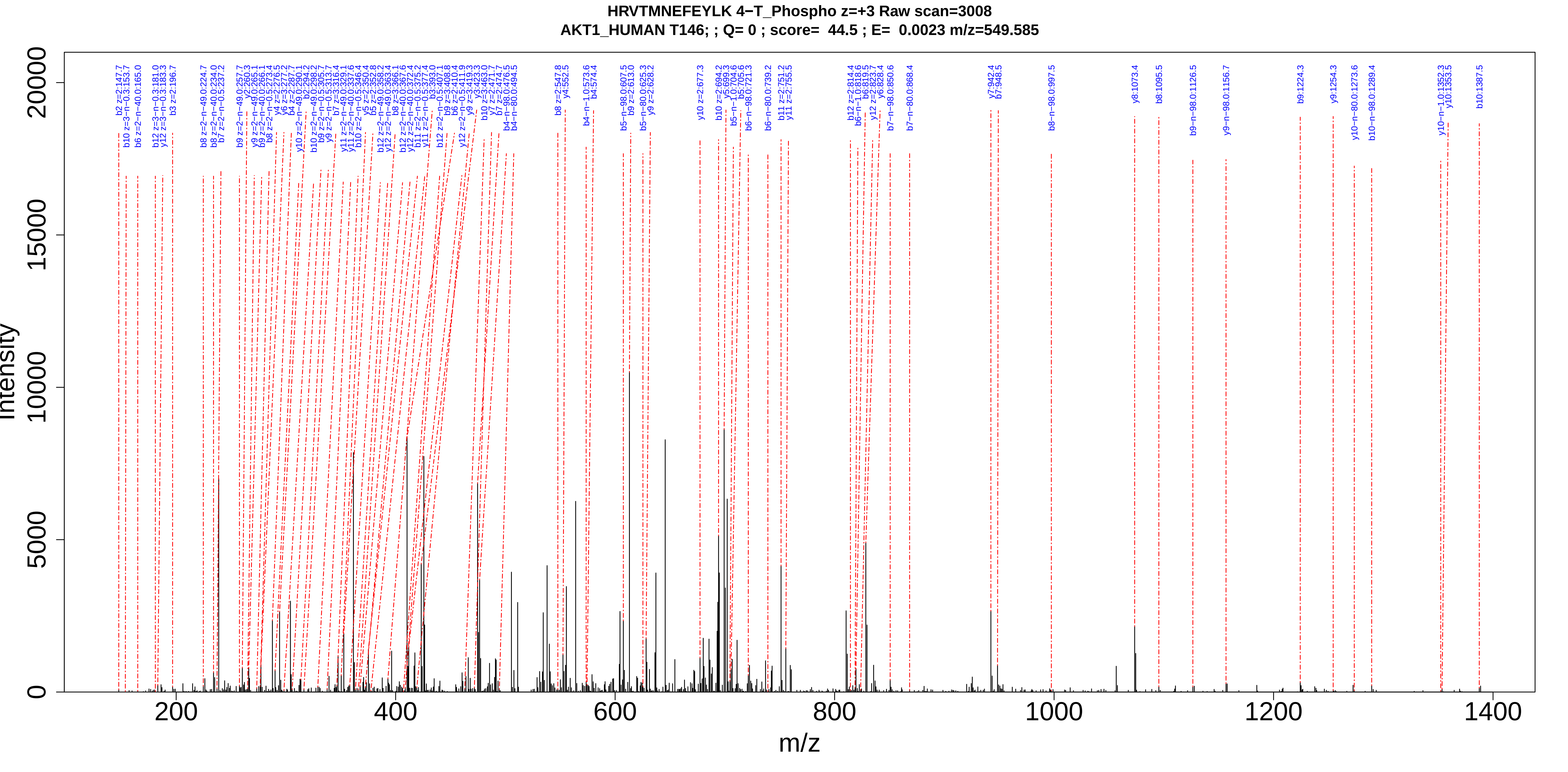
HRVTMNEFEYLK 4−T_Phospho z=+3 Raw scan=3008
AKT1_HUMAN T146; ; Q= 0 ; score= 44.5 ; E= 0.0023 m/z=549.585
Intensity
m/z
0
5000
10000
15000
20000
200
400
600
800
1000
1200
1400

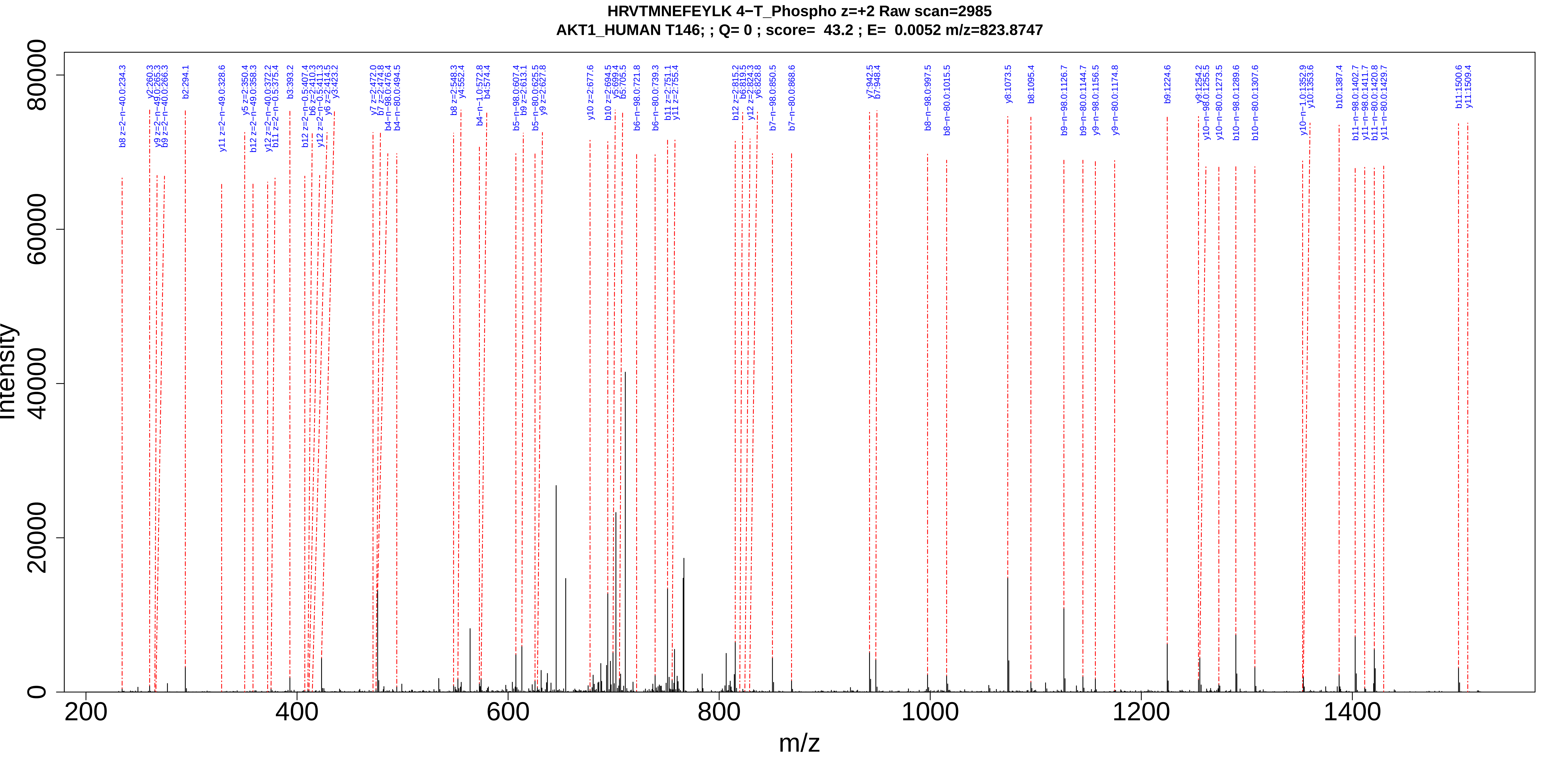

HRVTMNEFEYLK 4−T_Phospho z=+2 Raw scan=2985
AKT1_HUMAN T146; ; Q= 0 ; score= 43.2 ; E= 0.0052 m/z=823.8747
Intensity
m/z

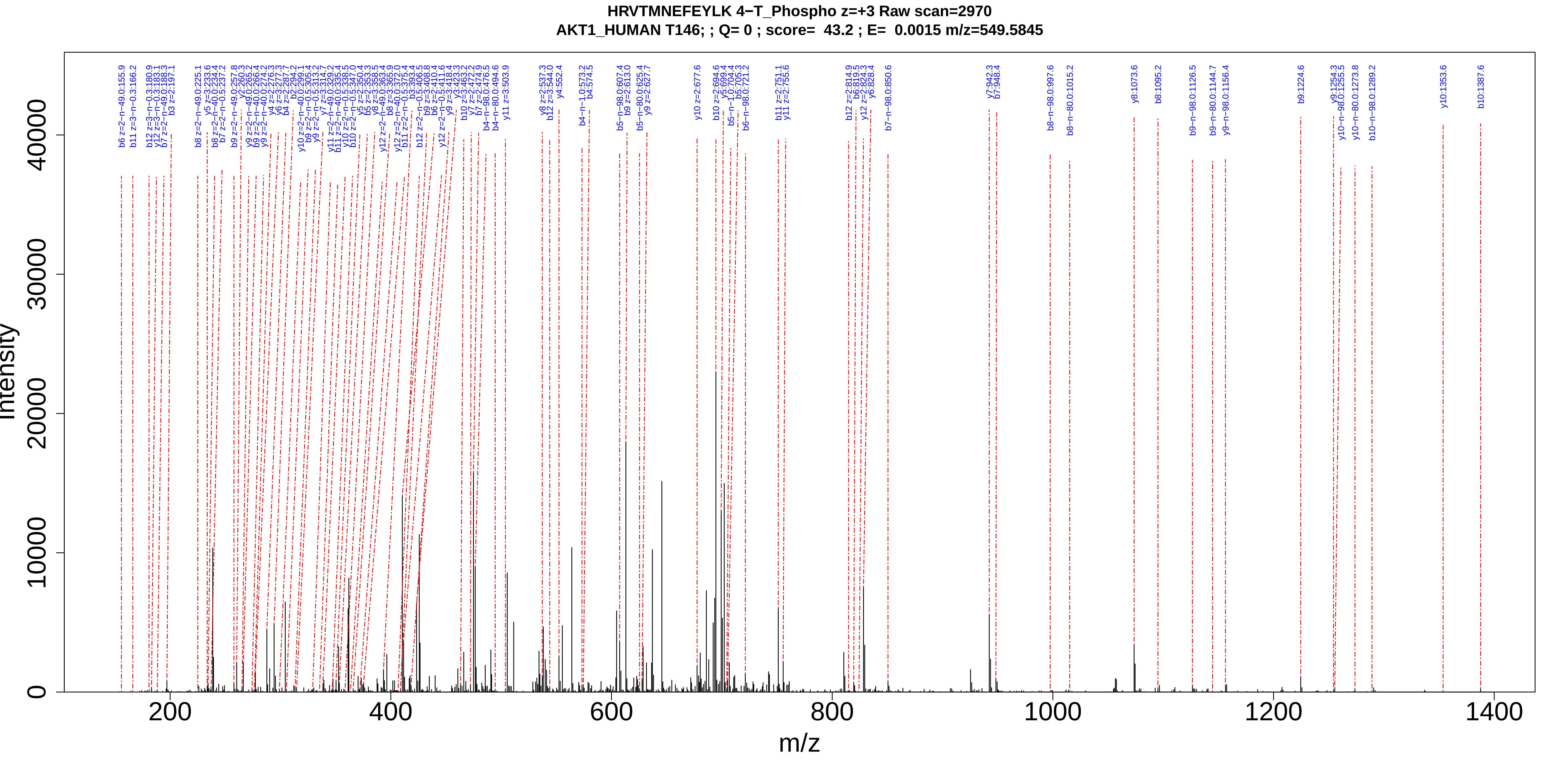
HRVTMNEFEYLK 4−T_Phospho z=+3 Raw scan=2970
AKT1_HUMAN T146; ; Q= 0 ; score= 43.2 ; E= 0.0015 m/z=549.5845
Intensity
m/z
0
10000
20000
30000
40000
200
400
600
800
1000
1200
1400

SFSQPGLR 3−S_Phospho z=+2 Raw scan=2116
TBCD1_HUMAN S237; ; Q= 0.01328125 ; score= 40.6 ; E= 7e−05 m/z=486.2207

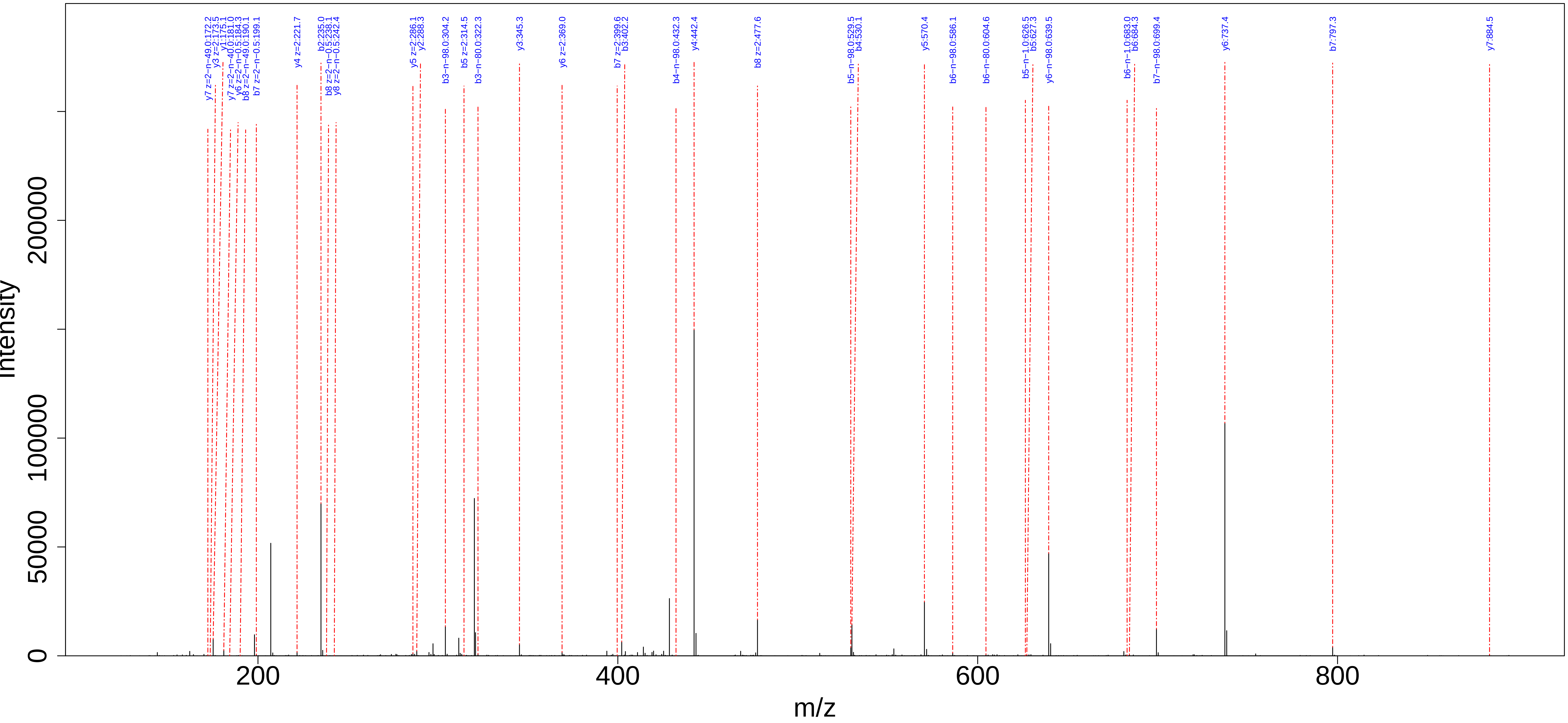

SFSQPGLR 3−S_Phospho z=+2 Raw scan=2123

TBCD1_HUMAN S237; ; Q= 0.0132760640374854 ; score= 40.6 ; E= 5.3e−05 m/z=486.2204

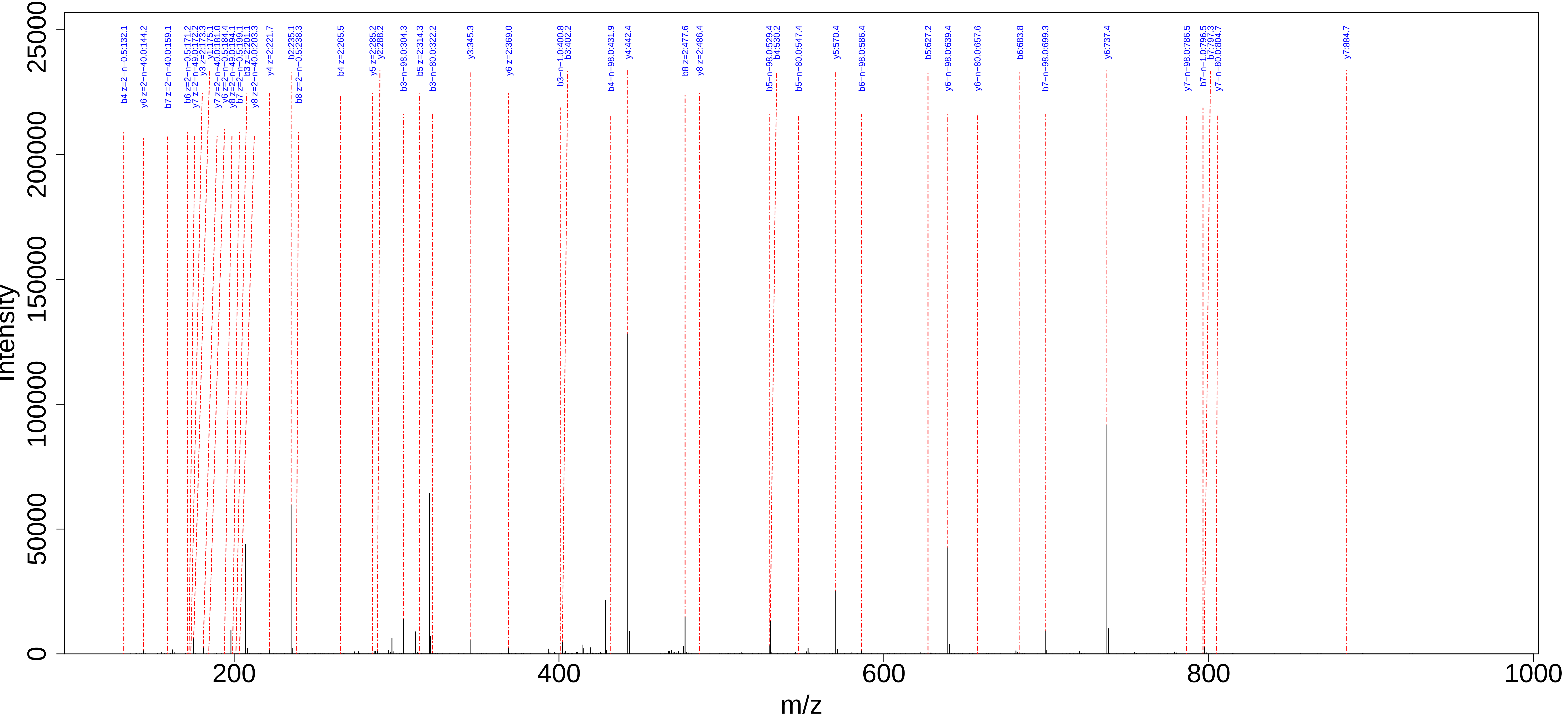

SFSQPGLR 3−S_Phospho z=+2 Raw scan=2102
TBCD1_HUMAN S237; ; Q= 0.0132708821233411 ; score= 40.6 ; E= 4.3e−05 m/z=486.2198

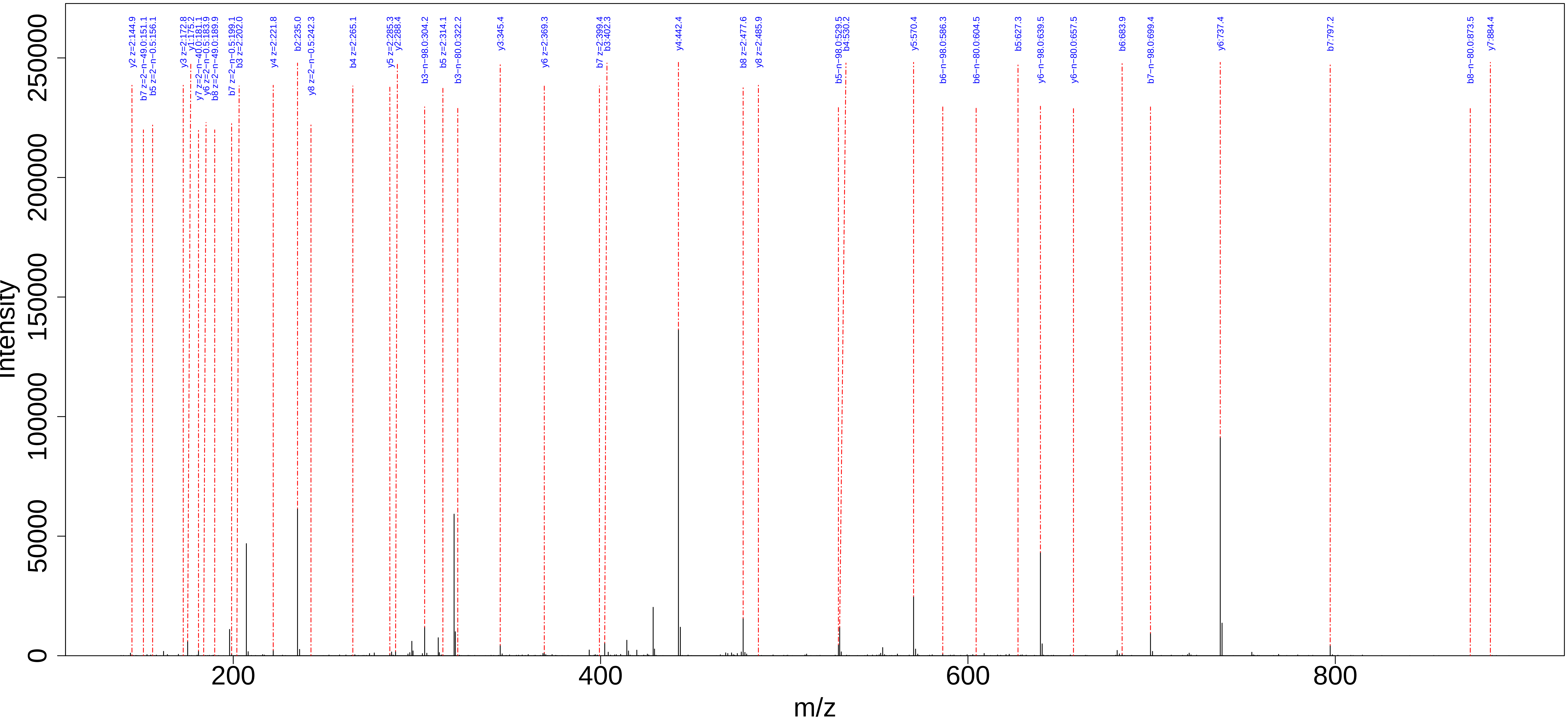

SFSQPGLR 3−S_Phospho z=+2 Raw scan=2109
TBCD1_HUMAN S237; ; Q= 0.0132657042528287 ; score= 40.6 ; E= 4.2e−05 m/z=486.2214

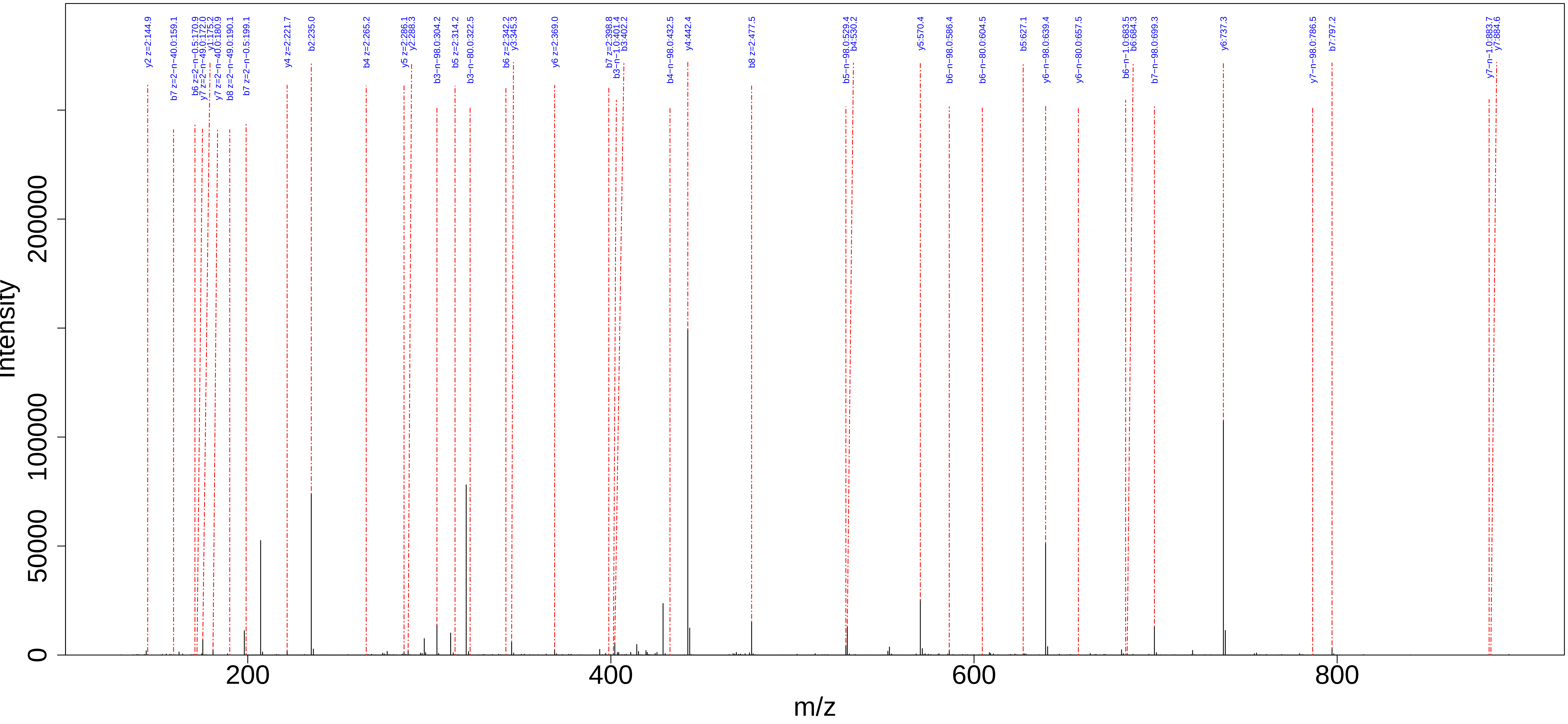

SFSQPGLR 3−S_Phospho z=+2 Raw scan=2130
TBCD1_HUMAN S237; ; Q= 0.0132605304212168 ; score= 40.6 ; E= 8.1e−05 m/z=486.2202

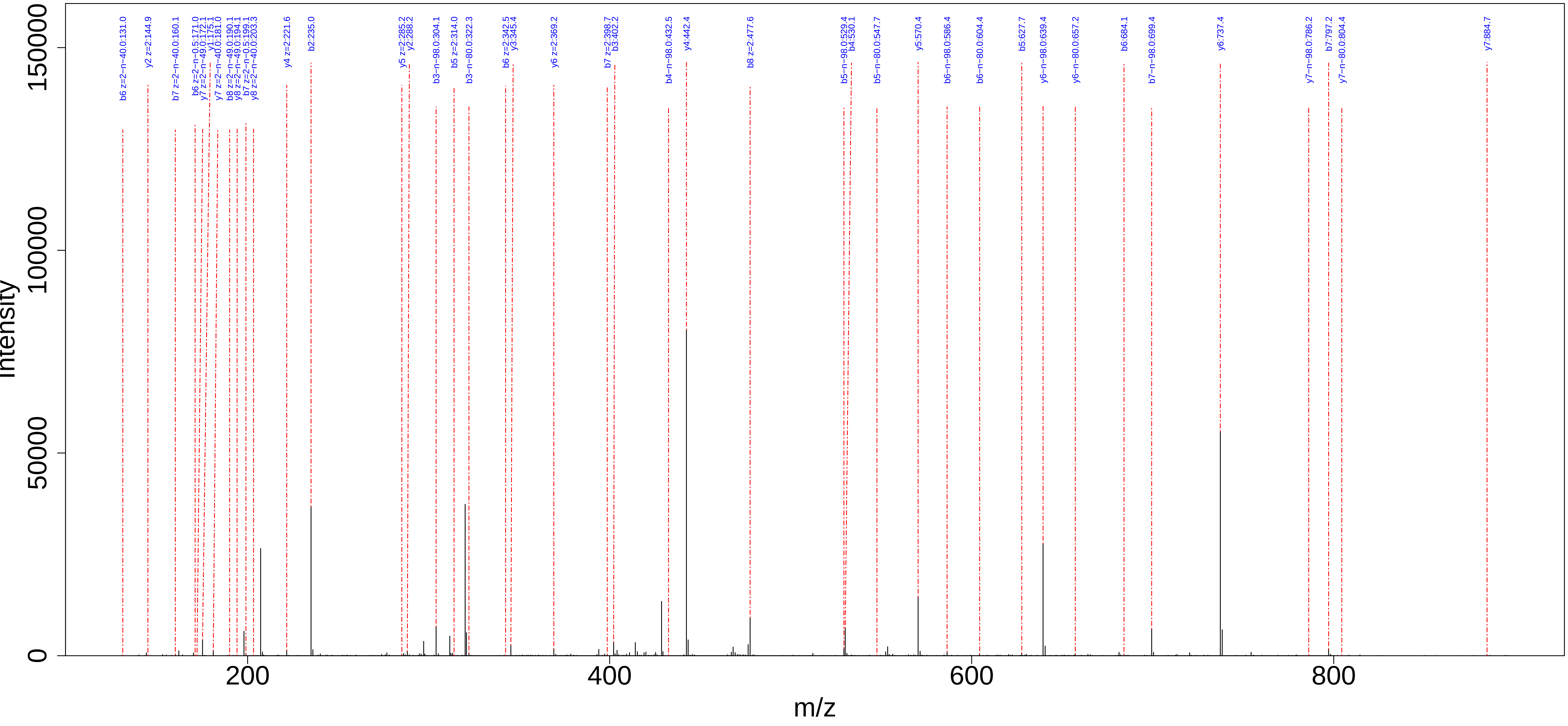

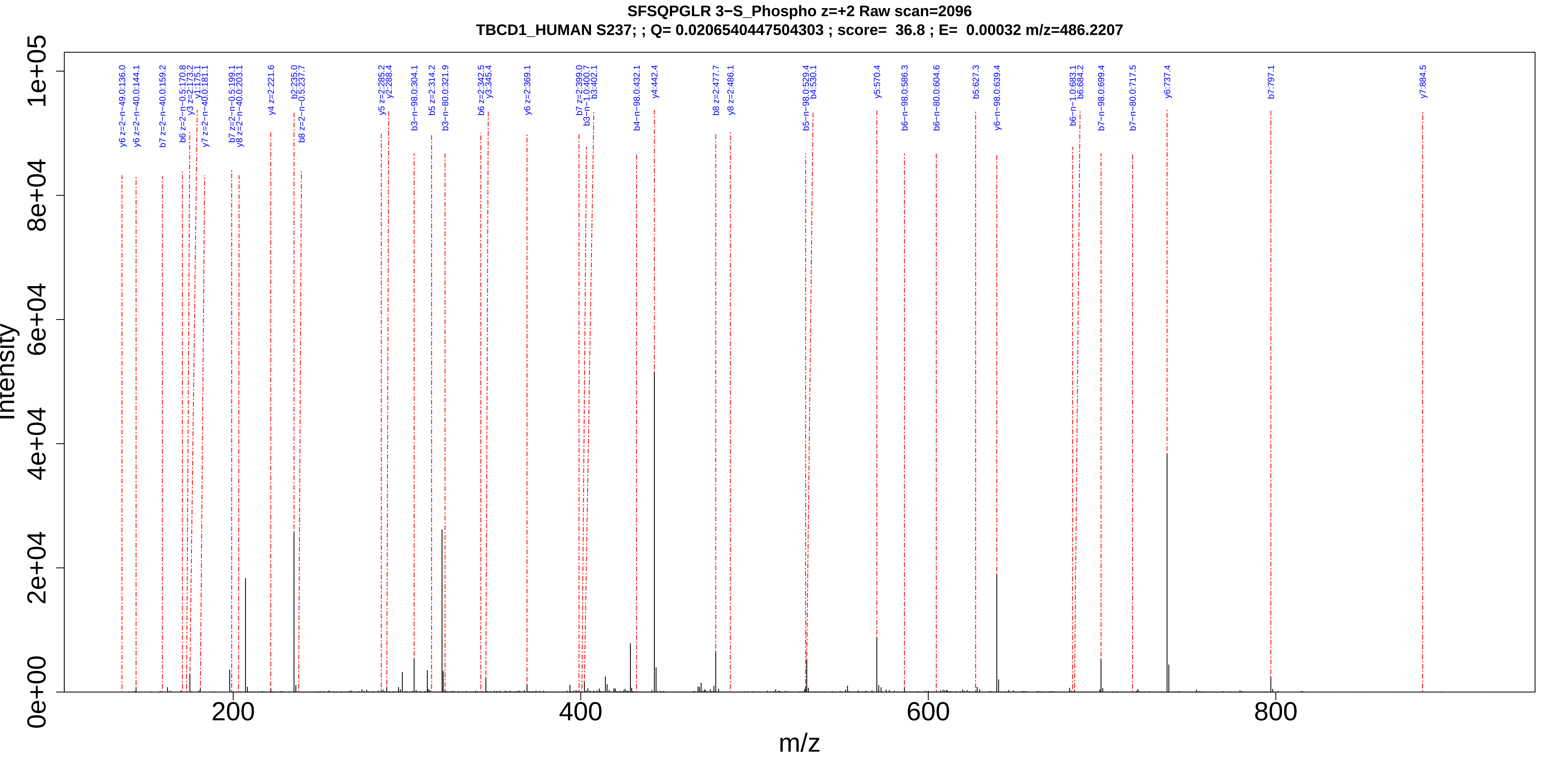
SFSQPGLR 3−S_Phospho z=+2 Raw scan=2096
TBCD1_HUMAN S237; ; Q= 0.0206540447504303 ; score= 36.8 ; E= 0.00032 m/z=486.2207
Intensity
m/z
0e+00
2e+04
4e+04
6e+04
8e+04
1e+05
200
400
600
800
y6 z=2−n−49.0:136.0
y6 z=2−n−40.0:144.1
b7 z=2−n−40.0:159.2
b6 z=2−n−0.5:170.8
y3 z=2:173.2
y1:175.1
y7 z=2−n−40.0:181.1
b7 z=2−n−0.5:199.1
y8 z=2−n−40.0:203.1
y4 z=2:221.6
b2:235.0
b8 z=2−n−0.5:237.7
y5 z=2:285.2
y2:288.4
b3−n−98.0:304.1
b5 z=2:314.2
b3−n−80.0:321.9
b6 z=2:342.5
y3:345.4
y6 z=2:369.1
b7 z=2:399.0
b3−n−1.0:400.7
b3:402.1
b4−n−98.0:432.1
y4:442.4
b8 z=2:477.7
y8 z=2:486.1
b5−n−98.0:529.4
b4:530.1
y5:570.4
b6−n−98.0:586.3
b6−n−80.0:604.6
b5:627.3
y6−n−98.0:639.4
b6−n−1.0:683.1
b6:684.2
b7−n−98.0:699.4
b7−n−80.0:717.5
y6:737.4
b7:797.1
y7:884.5

HRVTMNEFEYLK 4−T_Phospho z=+3 Raw scan=2984
AKT1_HUMAN T146; ; Q= 0.0219704771713011 ; score= 36.7 ; E= 0.031 m/z=549.5849

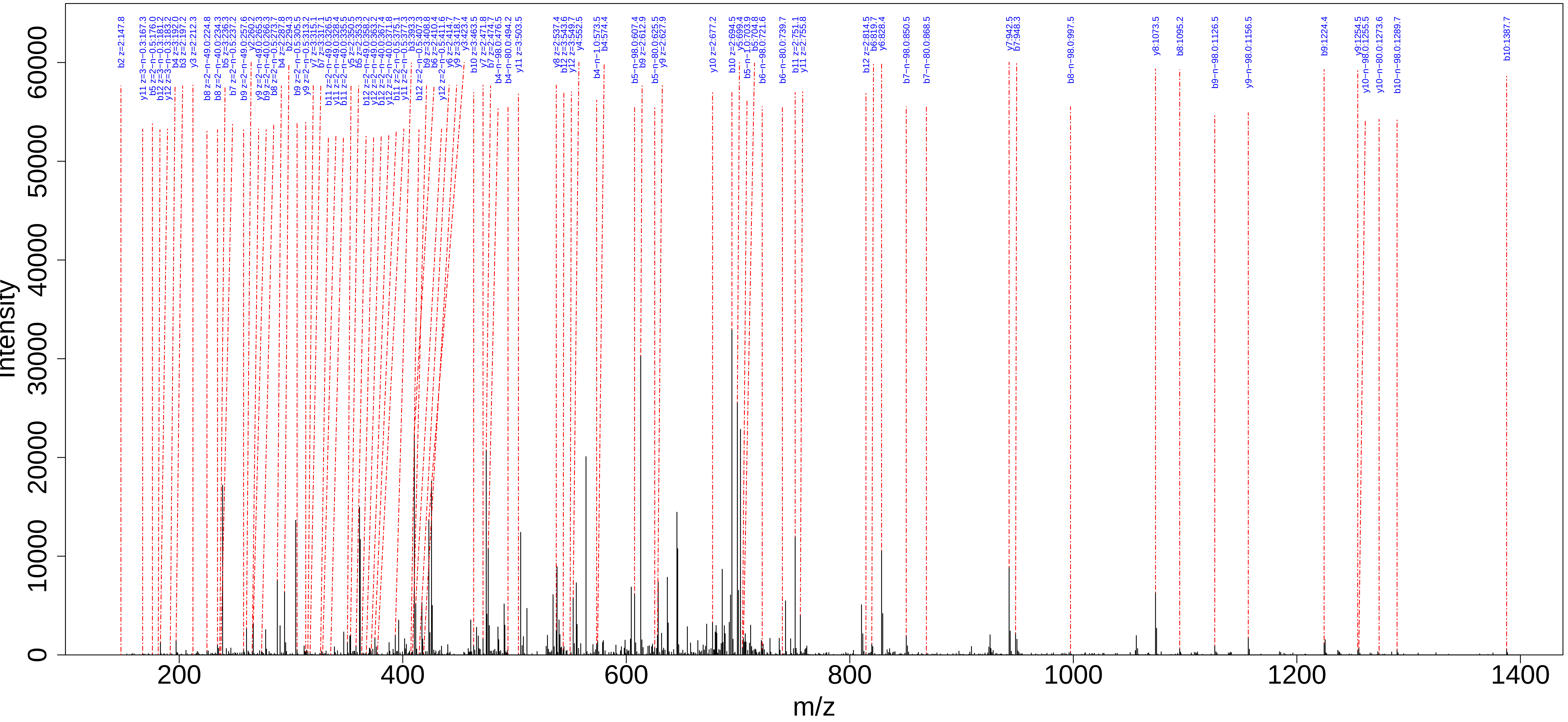

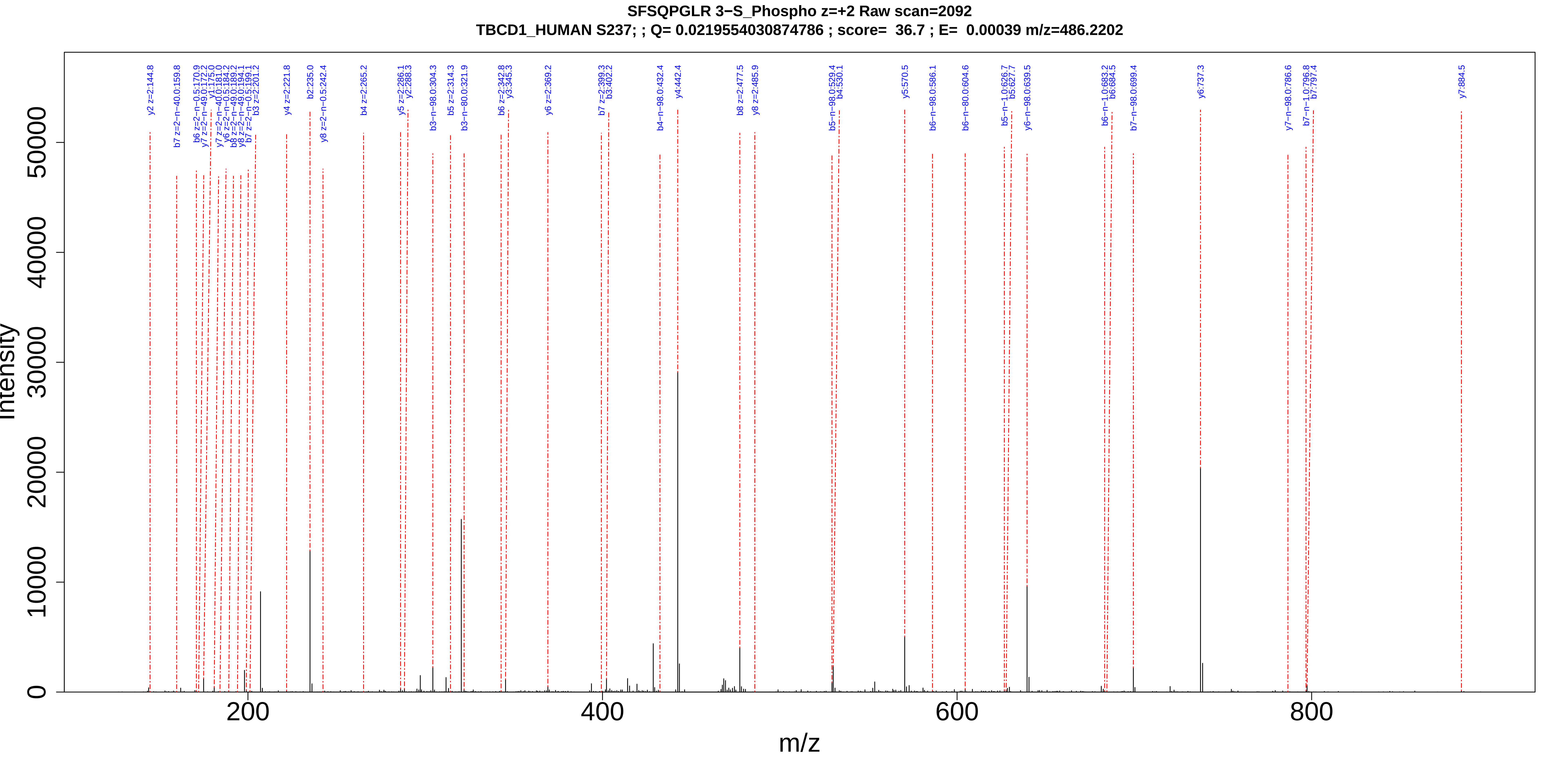

SFSQPGLR 3−S_Phospho z=+2 Raw scan=2092
TBCD1_HUMAN S237; ; Q= 0.0219554030874786 ; score= 36.7 ; E= 0.00039 m/z=486.2202
Intensity
m/z

HRVTMNEFEYLK 4−T_Phospho z=+2 Raw scan=3000
AKT1_HUMAN T146; ; Q= 0.0212201591511936 ; score= 35.8 ; E= 0.052 m/z=823.8723

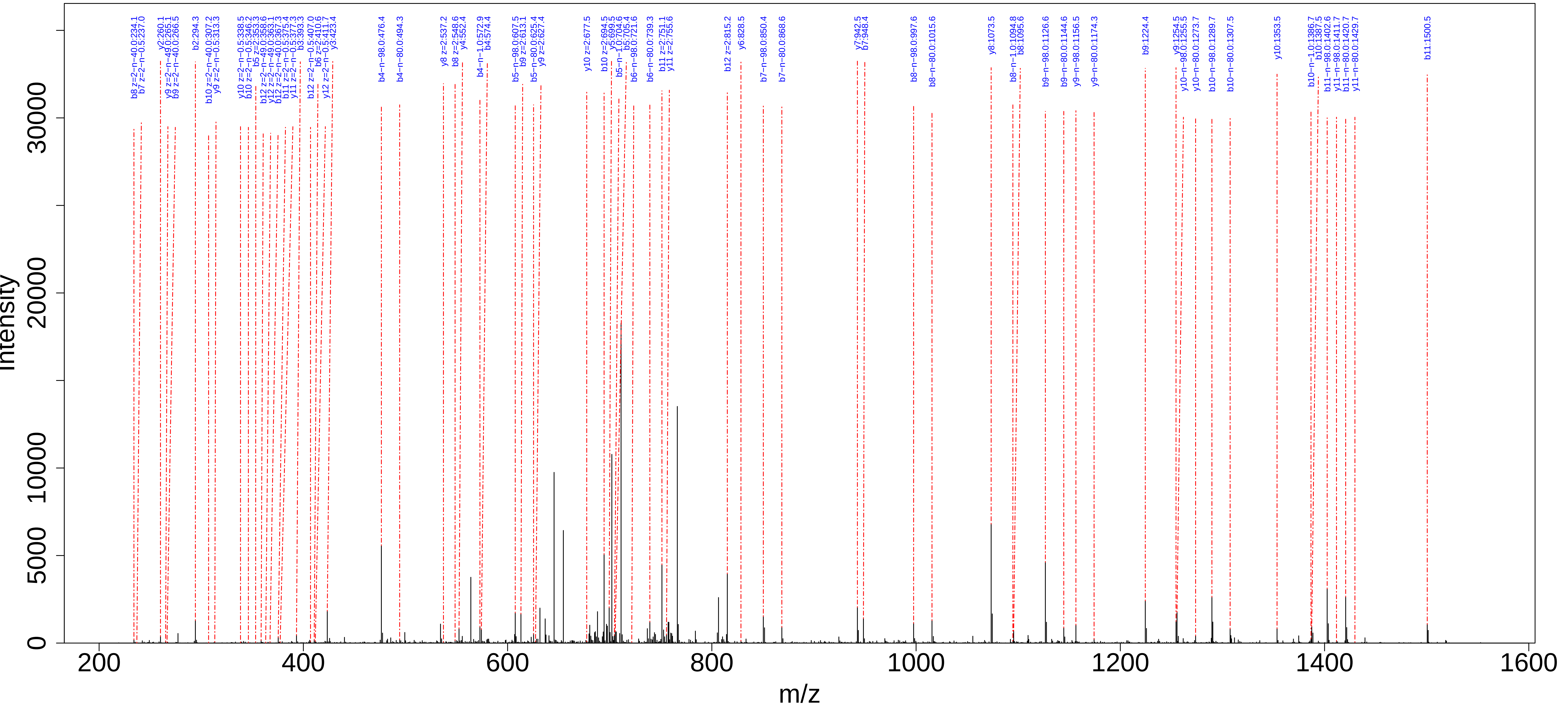

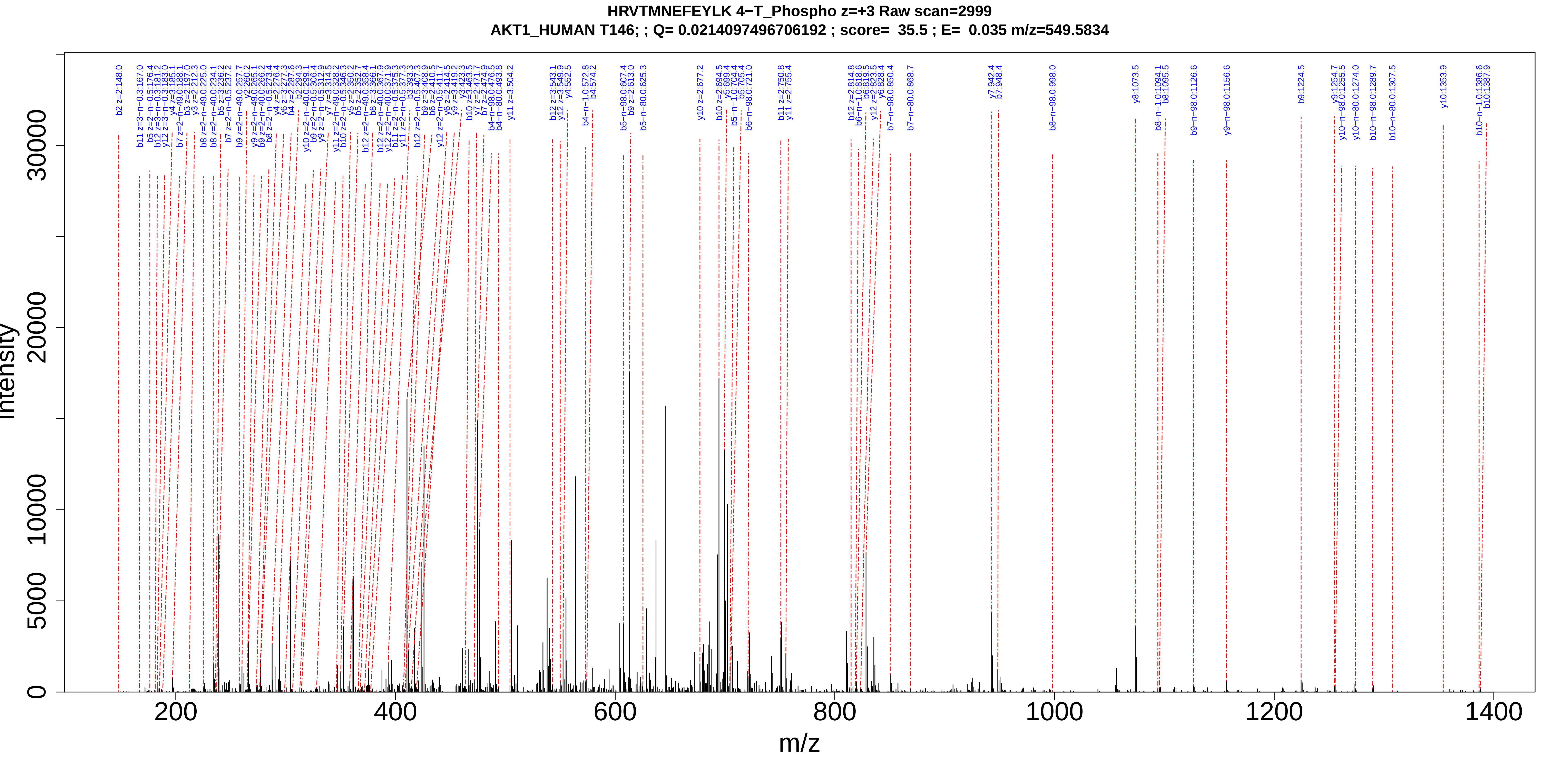
HRVTMNEFEYLK 4−T_Phospho z=+3 Raw scan=2999
AKT1_HUMAN T146; ; Q= 0.0214097496706192 ; score= 35.5 ; E= 0.035 m/z=549.5834
Intensity
m/z

annotated raw MSMS spectra for assay:

Protein kinase: AKT1

Substrate: TBC1D4

sample name: TBCD4_AKT1_phos_1

SFYQNSGRLSPQYENEIRQDTASESSDGEGR 10−S_Phospho z=+3 Raw scan=2799
TBCD4_HUMAN S725; ; Q= 0 ; score= 49.8 ; E= 6.6e−05 m/z=1196.5231

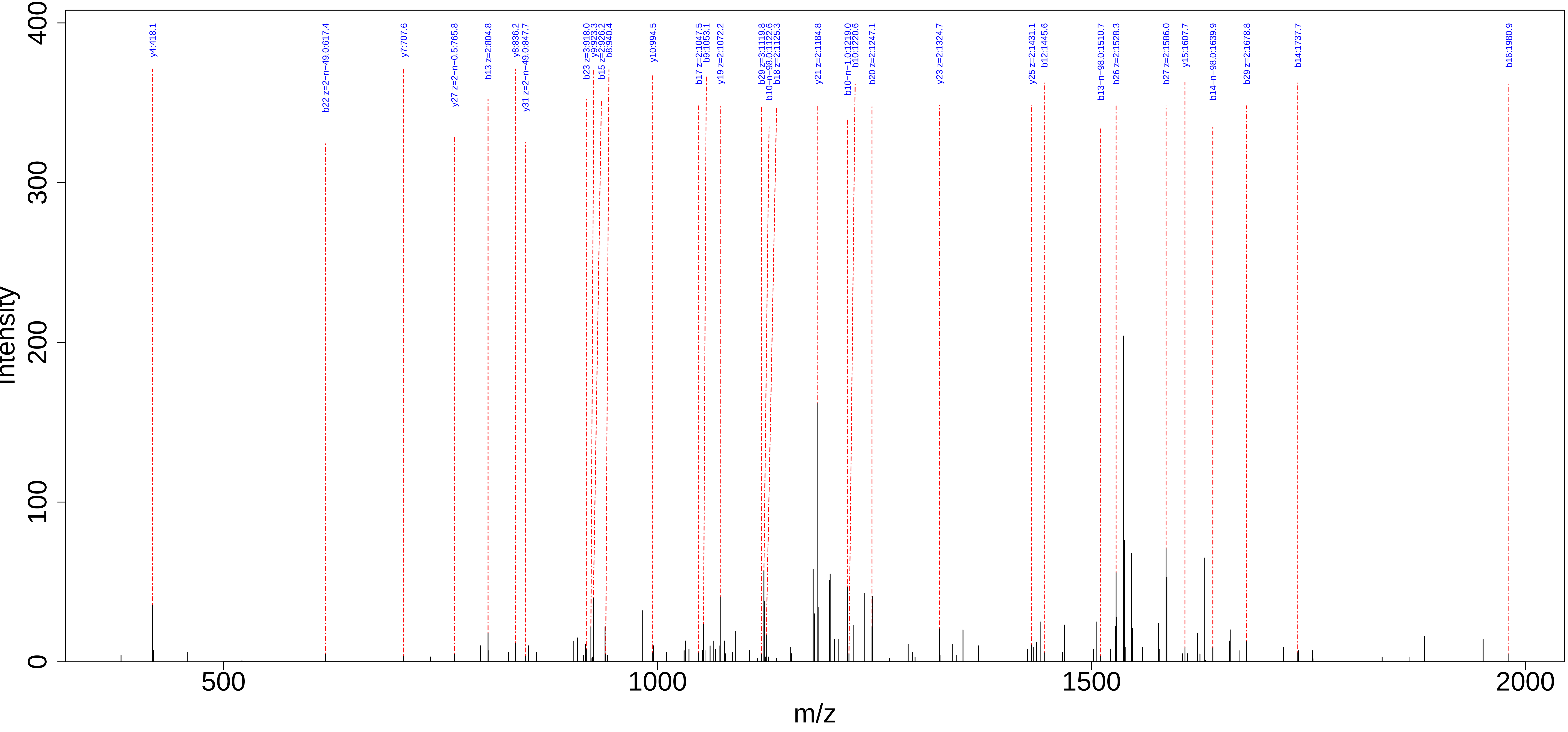

SFYQNSGRLSPQYENEIRQDTASESSDGEGR 10−S_Phospho z=+3 Raw scan=2822
TBCD4_HUMAN S725; ; Q= 0 ; score= 42.4 ; E= 0.017 m/z=1196.5208

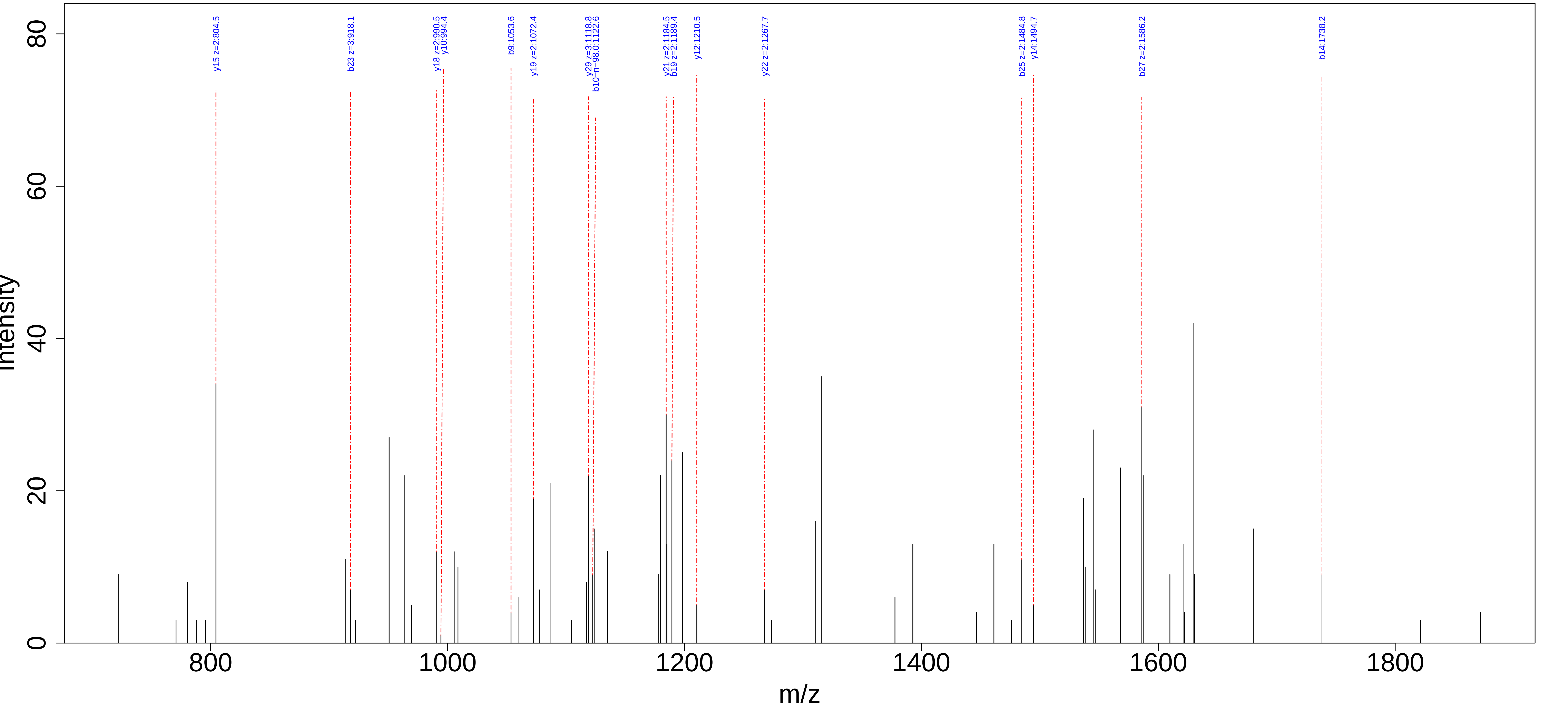

SFYQNSGRLSPQYENEIRQDTASESSDGEGR 10−S_Phospho z=+3 Raw scan=2792
TBCD4_HUMAN S721; ; Q= 0 ; score= 40.9 ; E= 0.033 m/z=1196.5231

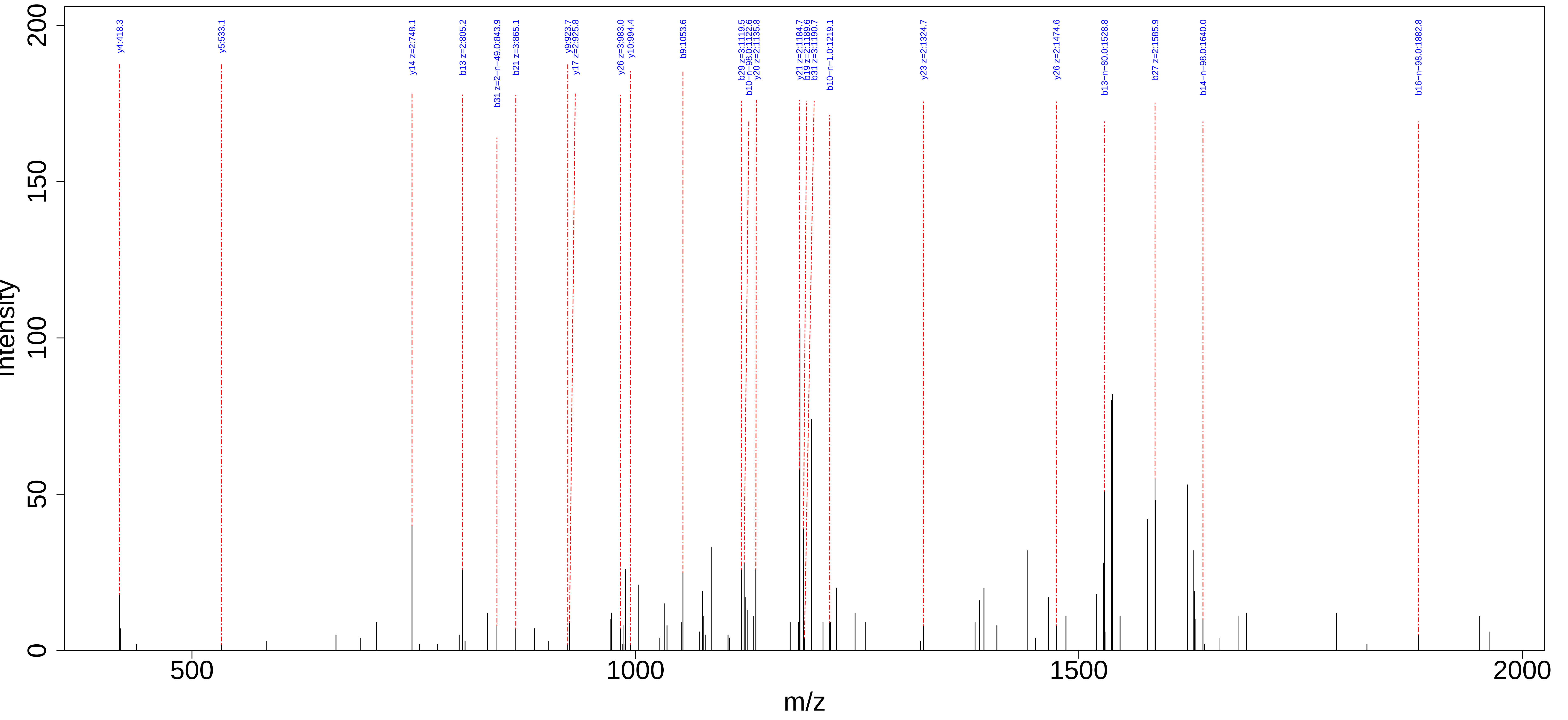

SLTSSLENIFSR 3−T_Phospho z=+2 Raw scan=4494
TBCD4_HUMAN T568; ; Q= 0 ; score= 36.3 ; E= 0.0034 m/z=717.3382

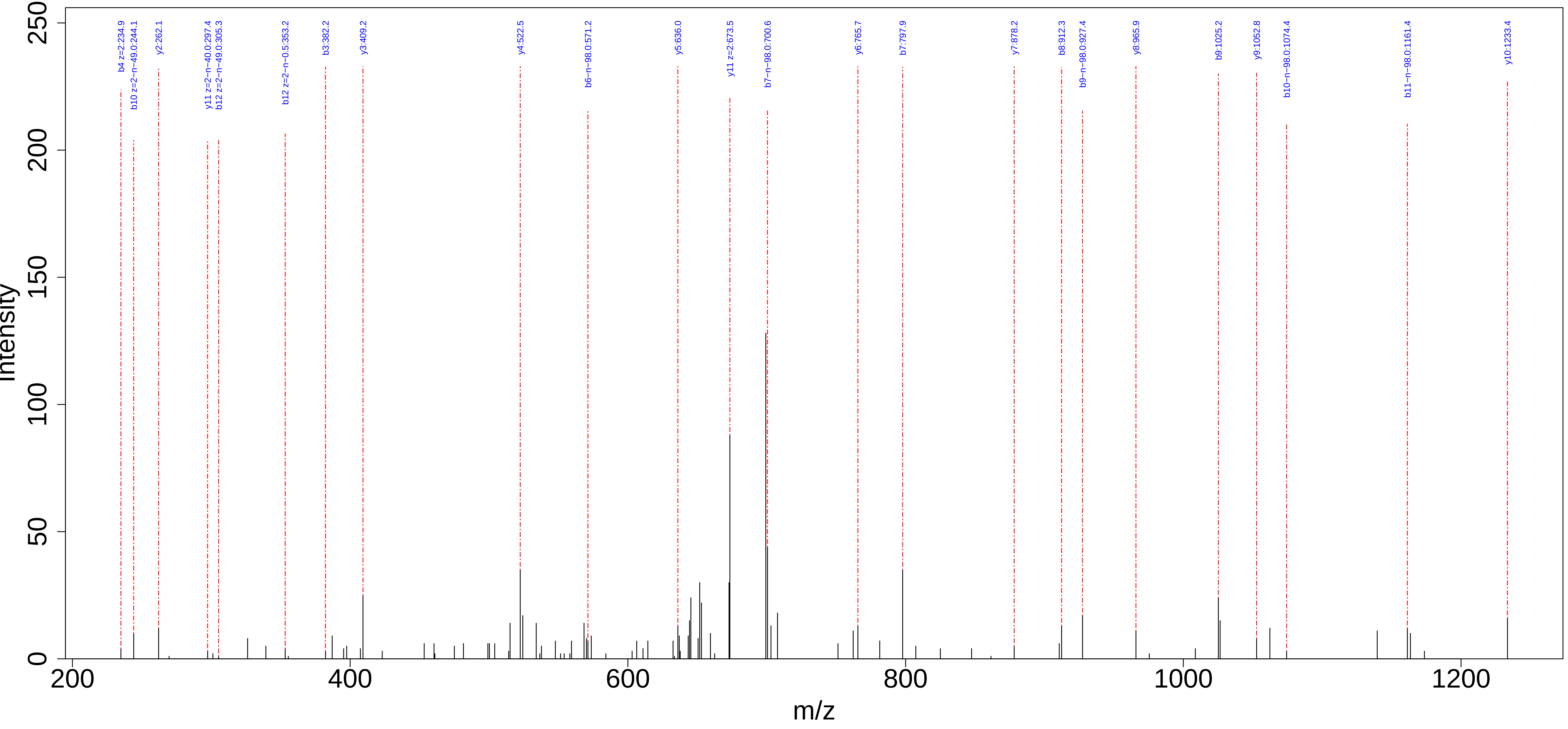

SLTSSLENIFSR 3−T_Phospho z=+2 Raw scan=4472
TBCD4_HUMAN T568; ; Q= 0 ; score= 31.5 ; E= 0.017 m/z=717.3365

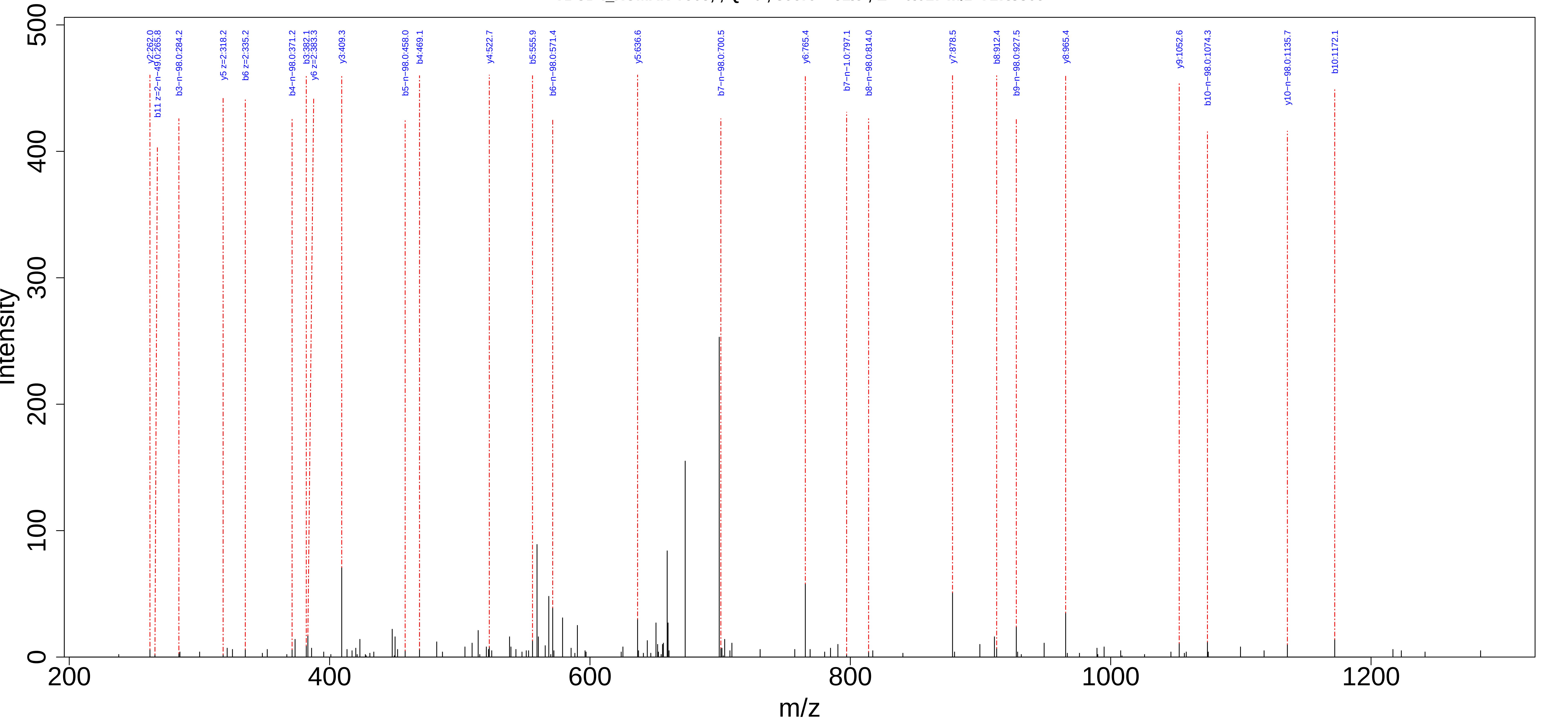

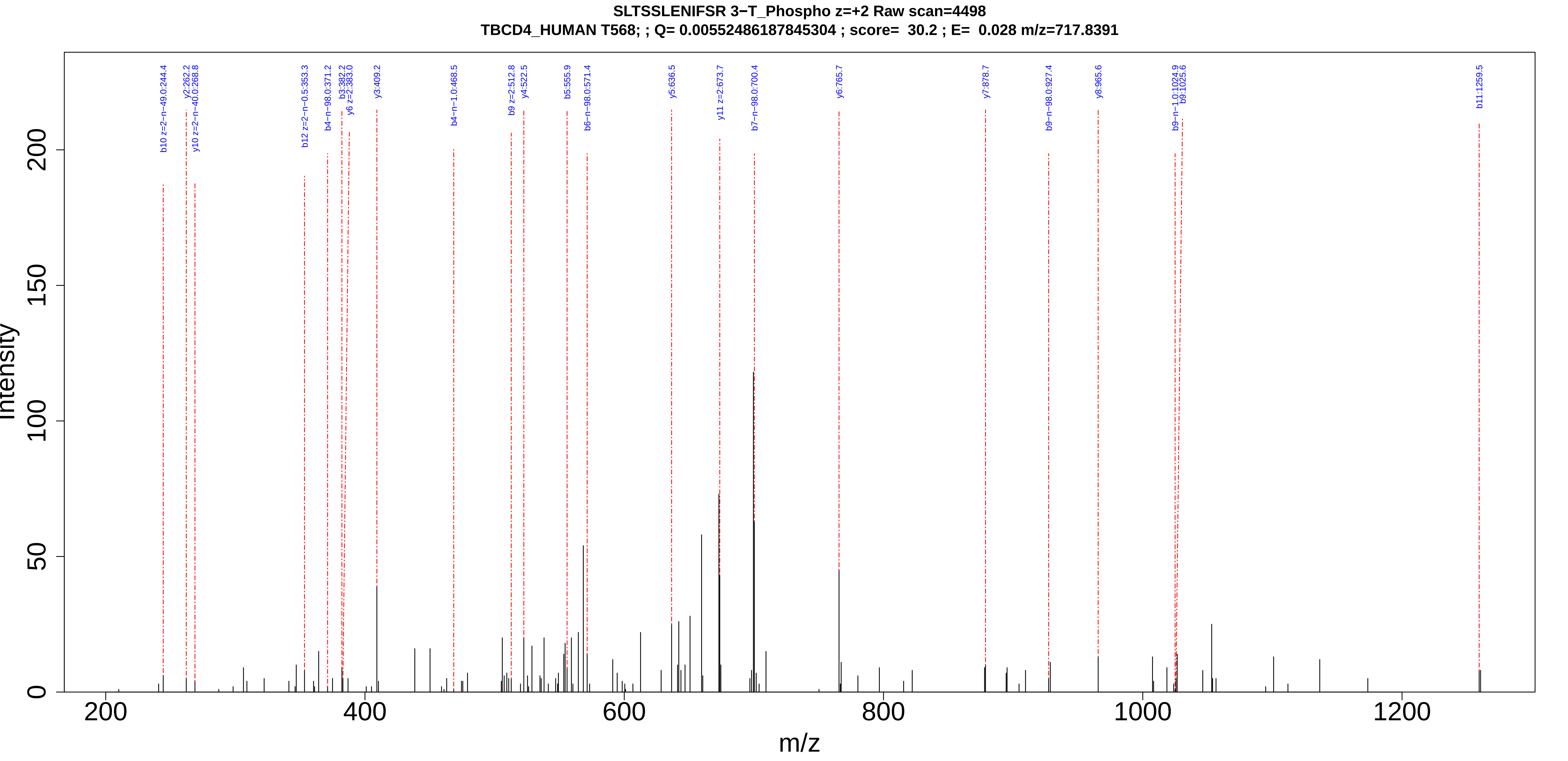

SLTSSLENIFSR 3−T_Phospho z=+2 Raw scan=4498
TBCD4_HUMAN T568; ; Q= 0.00552486187845304 ; score= 30.2 ; E= 0.028 m/z=717.8391
Intensity
m/z

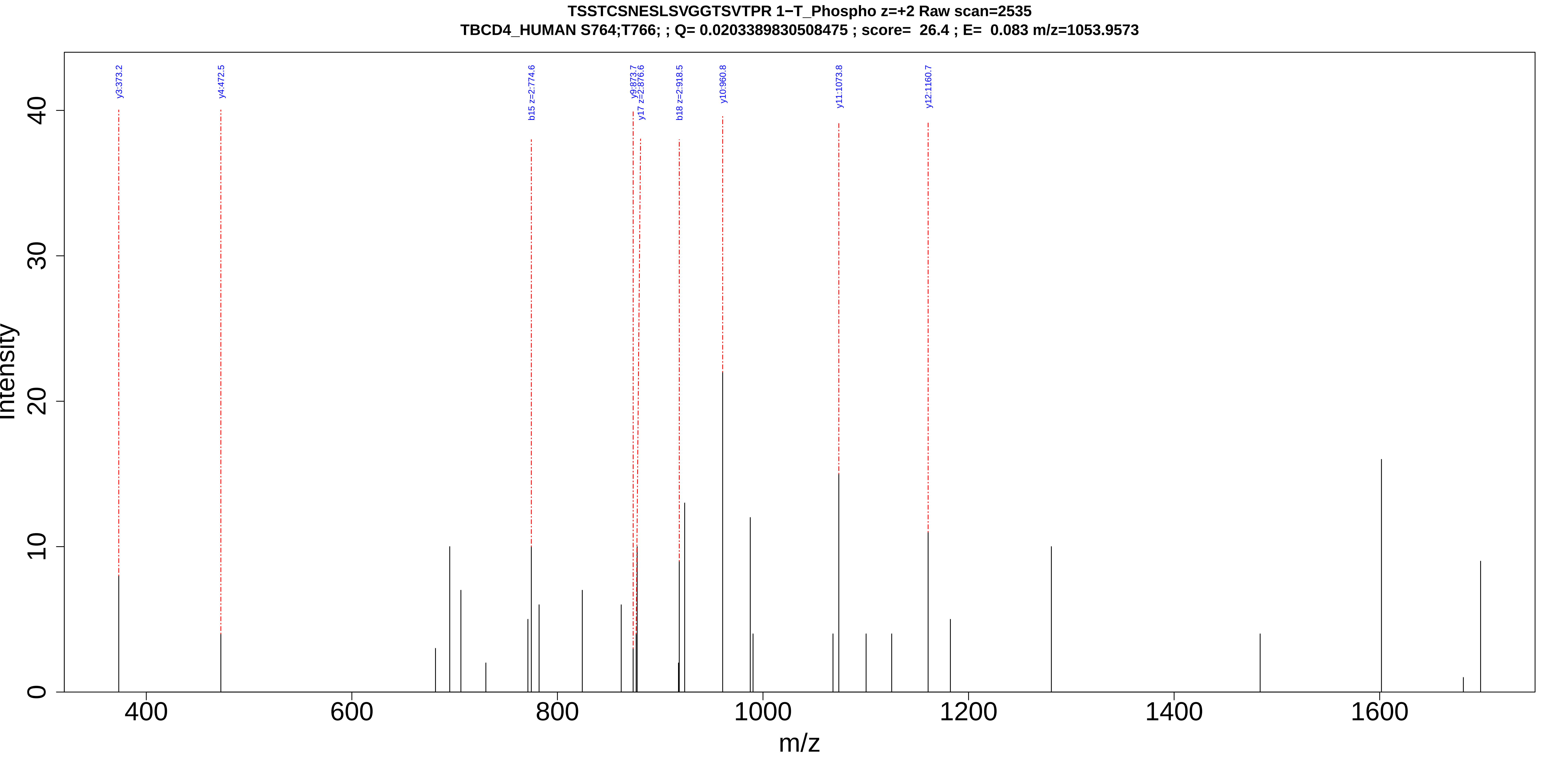
TSSTCSNESLSVGGTSVTPR 1−T_Phospho z=+2 Raw scan=2535
TBCD4_HUMAN S764;T766; ; Q= 0.0203389830508475 ; score= 26.4 ; E= 0.083 m/z=1053.9573
y3:373.2
y4:472.5
b15 z=2:774.6
y9:873.7
y17 z=2:876.6
b18 z=2:918.5
y10:960.8
y11:1073.8
y12:1160.7
Intensity
0
10
20
30
40
400
600
800
1000
1200
1400
1600
m/z

TSSTCSNESLSVGGTSVTPR 2−S_Phospho z=+2 Raw scan=2535
TBCD4_HUMAN S764;T766; ; Q= 0.0203045685279188 ; score= 26.4 ; E= 0.083 m/z=1053.9573

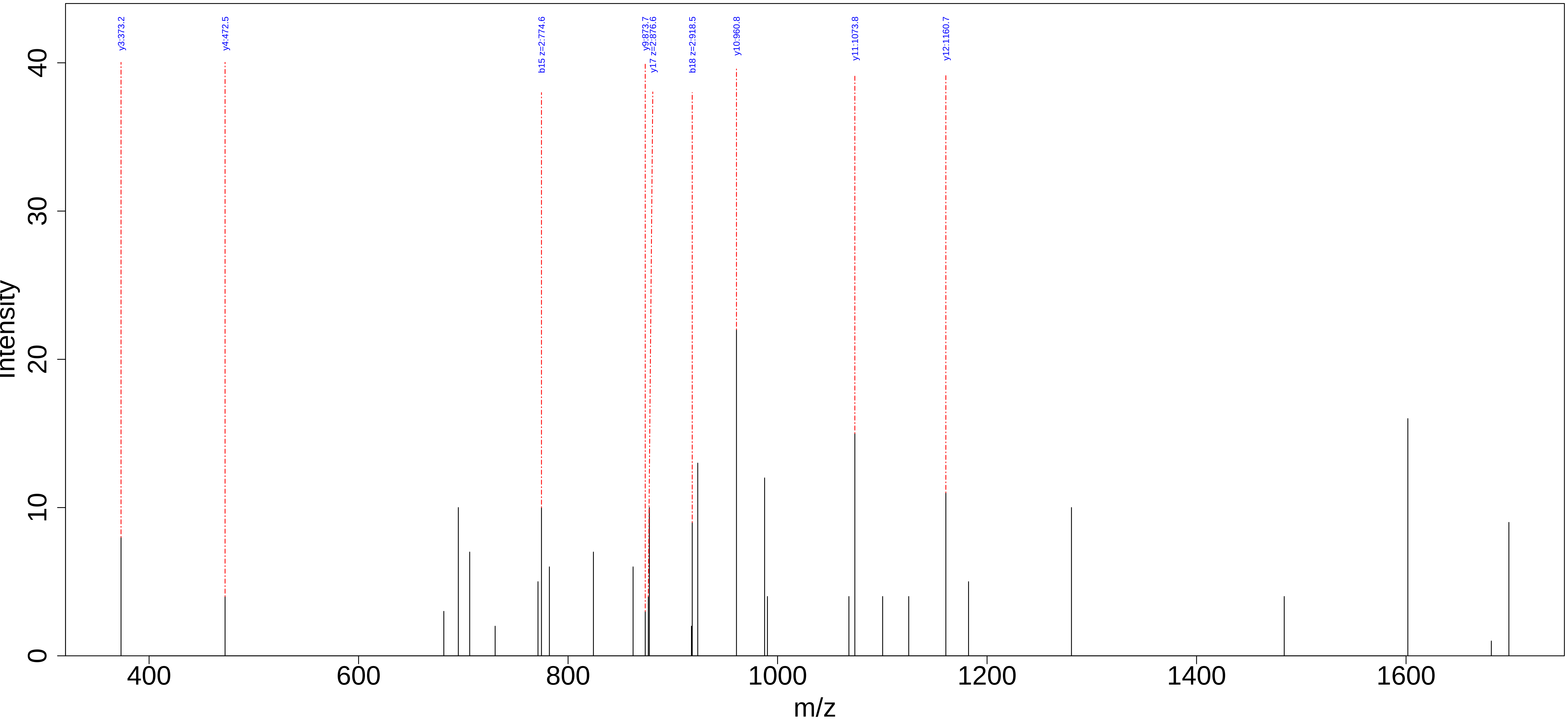

TSSTCSNESLSVGGTSVTPR 3−S_Phospho z=+2 Raw scan=2535
TBCD4_HUMAN S764;T766; ; Q= 0.0202702702702703 ; score= 26.4 ; E= 0.083 m/z=1053.9573

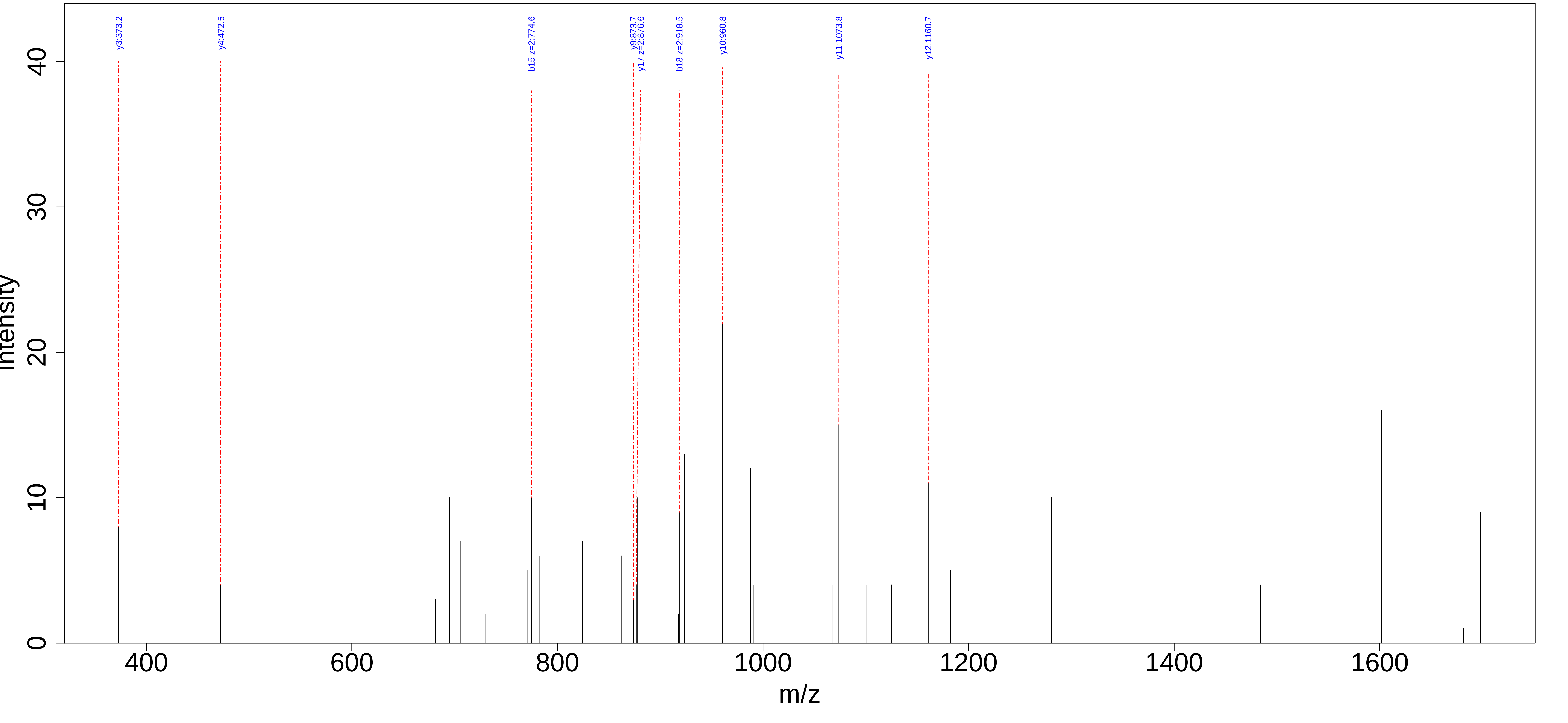

TSSTCSNESLSVGGTSVTPR 4−T_Phospho z=+2 Raw scan=2535
TBCD4_HUMAN S764;T766; ; Q= 0.0202360876897133 ; score= 26.4 ; E= 0.083 m/z=1053.9573

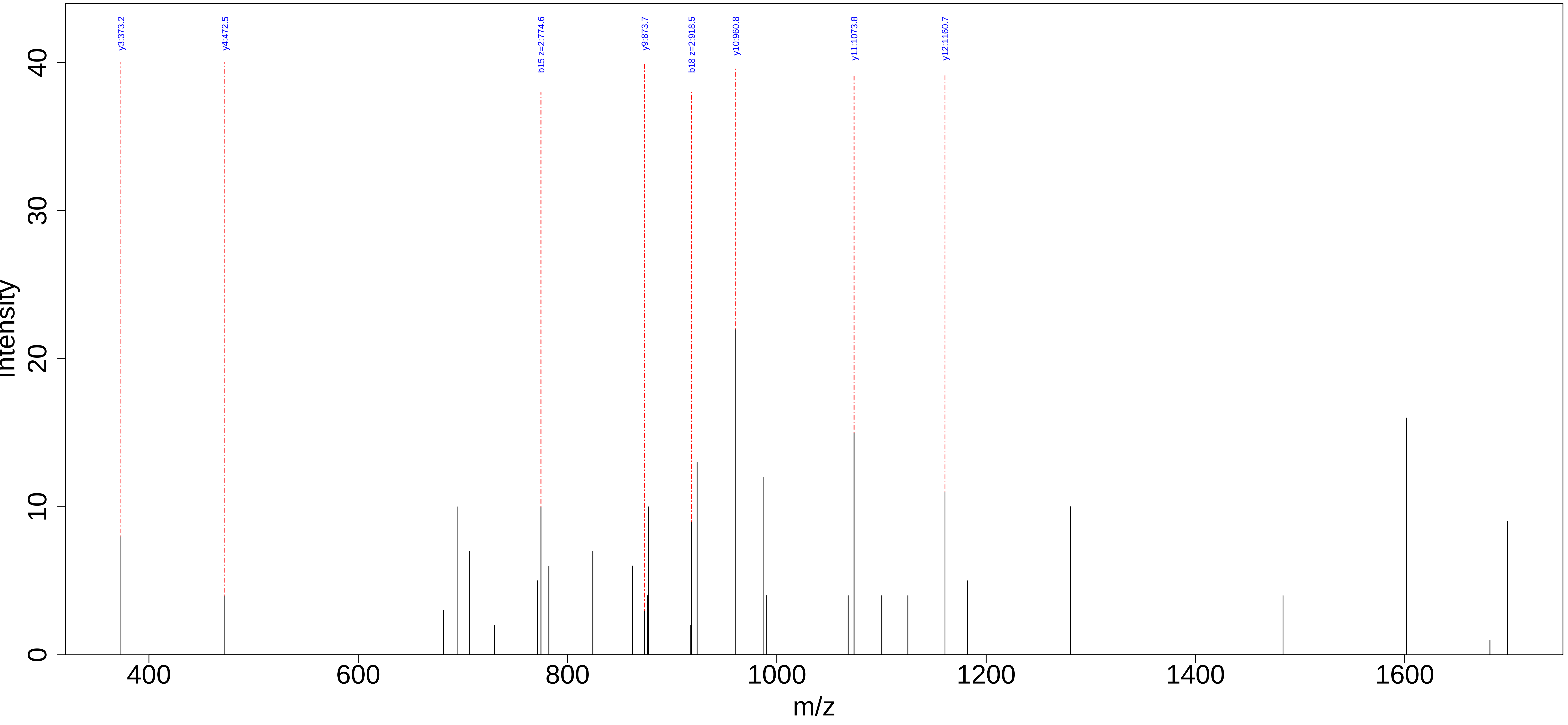

TSSTCSNESLSVGGTSVTPR 6−S_Phospho z=+2 Raw scan=2535
TBCD4_HUMAN S764;T766; ; Q= 0.0202020202020202 ; score= 26.4 ; E= 0.083 m/z=1053.9573

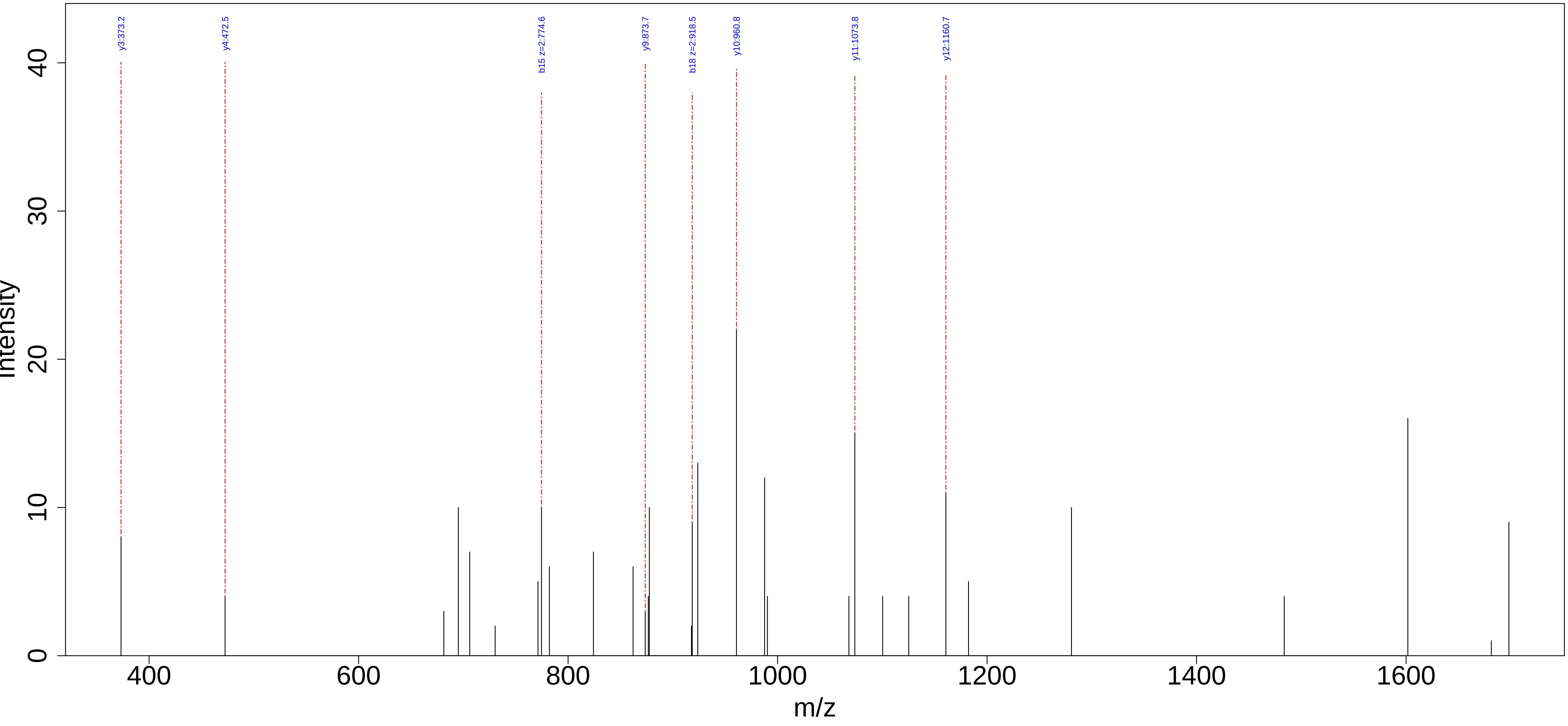

LKNDGTFIGYKER 10−Y_Phospho/1−n_proAcetyl z=+2 Raw scan=3139
AKT1_HUMAN T34; ; Q= 0.0198347107438017 ; score= 26 ; E= 0.082 m/z=831.9059

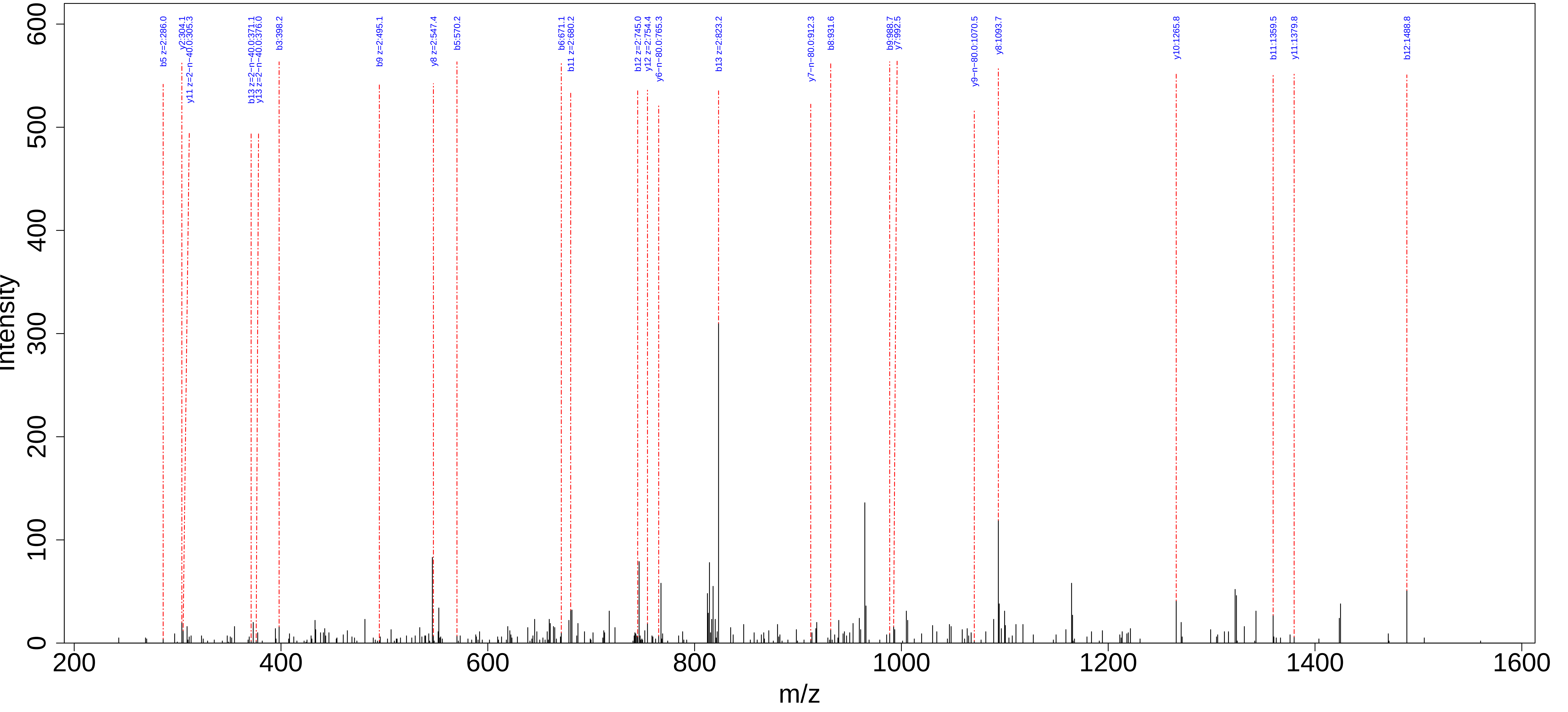

annotated raw MSMS spectra for assay:

Protein kinase: WNK1

Substrate: TBC1D1

sample name: TBCD1_WNK1_3

LLGSSEDLSSDSESHLPEEPAPLSPQQAFR 24−S_Phospho z=+3 Raw scan=3571
TBCD1_HUMAN S585; ; Q= 0 ; score= 121 ; E= 1e−15 m/z=1101.8461

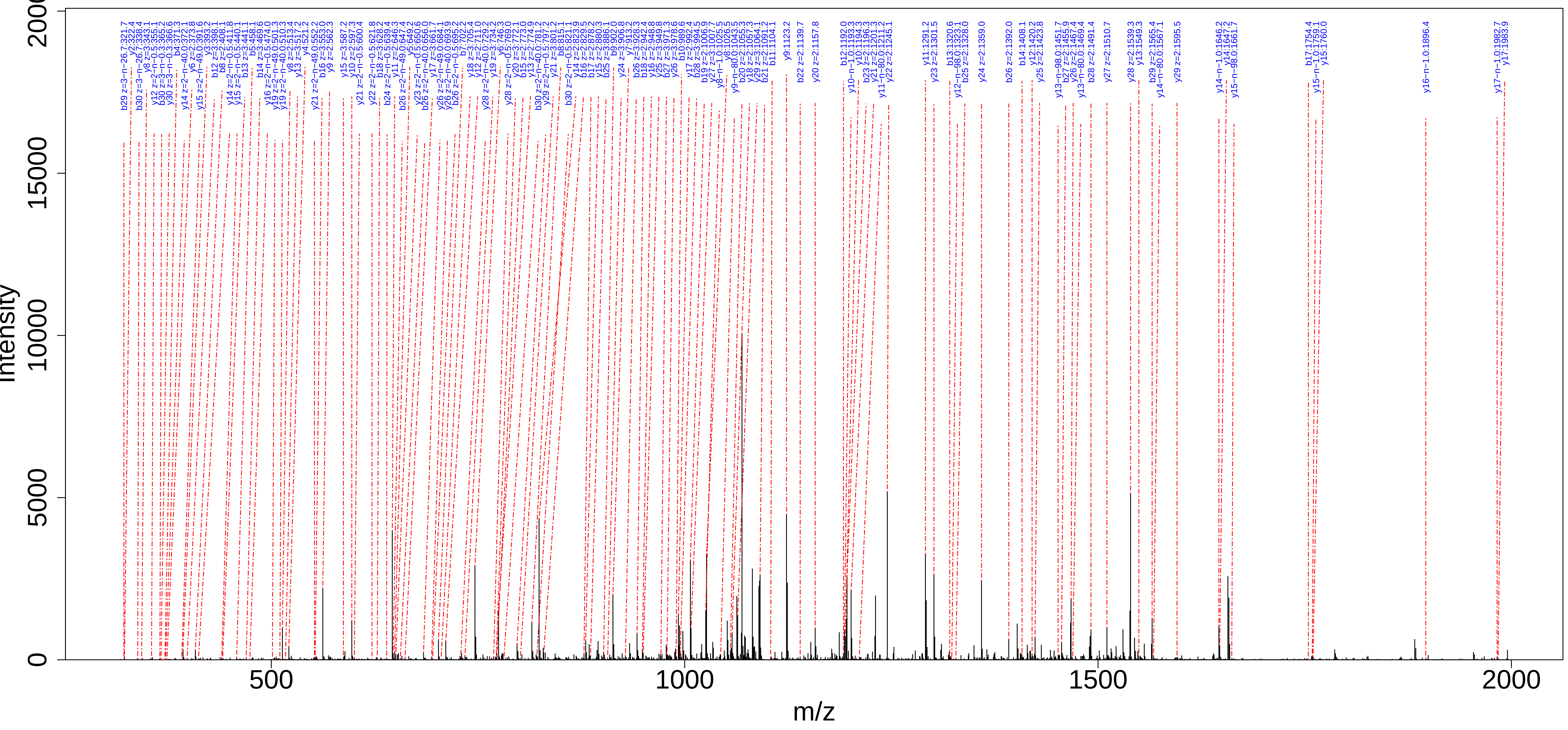

LLGSSEDLSSDSESHLPEEPAPLSPQQAFR 24−S_Phospho z=+3 Raw scan=3562
TBCD1_HUMAN S570; ; Q= 0 ; score= 101.6 ; E= 1e−15 m/z=1101.8488

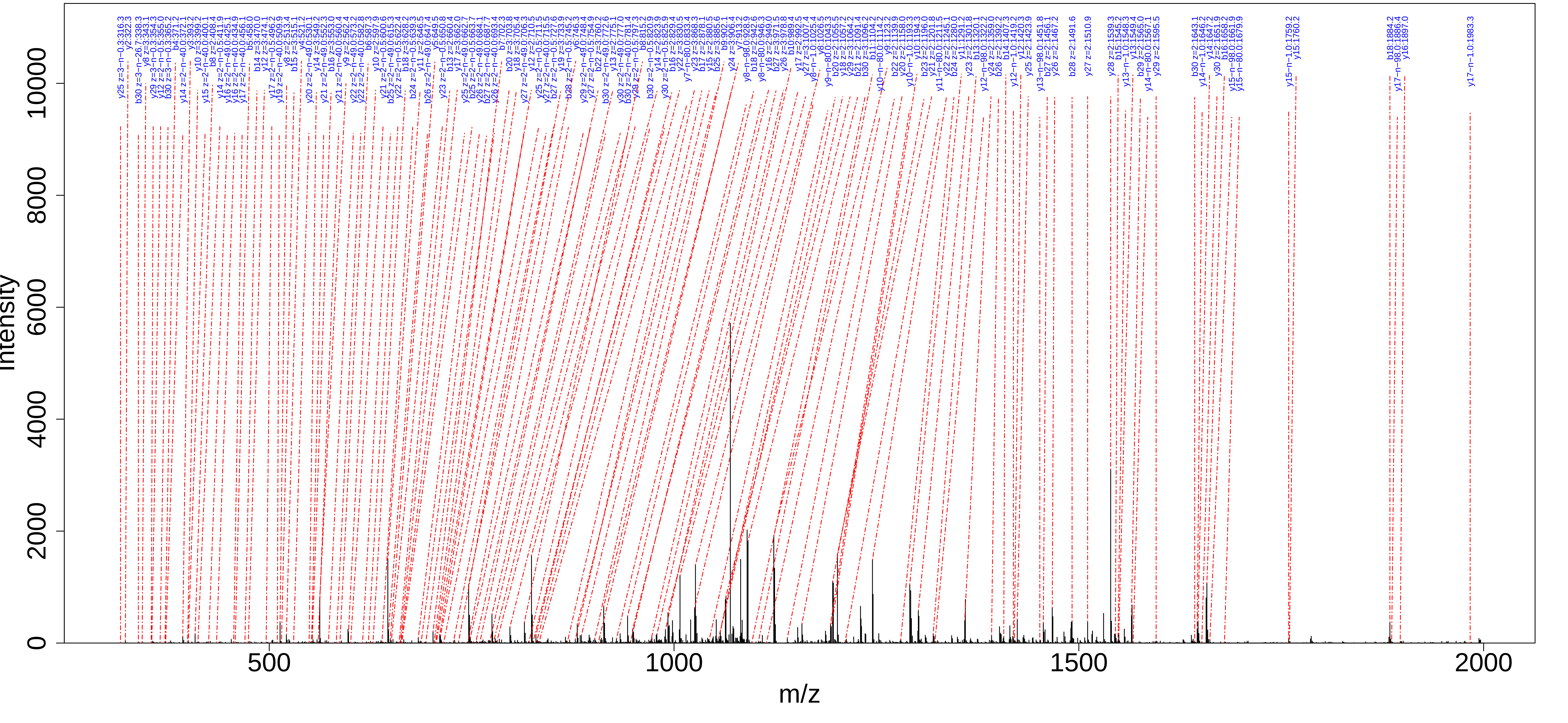

LLGSSEDLSSDSESHLPEEPAPLSPQQAFR 4−S_Phospho/24−S_Phospho z=+3 Raw scan=3705
TBCD1_HUMAN S565;S585; ; Q= 0 ; score= 85.7 ; E= 4.9e−13 m/z=1128.5012

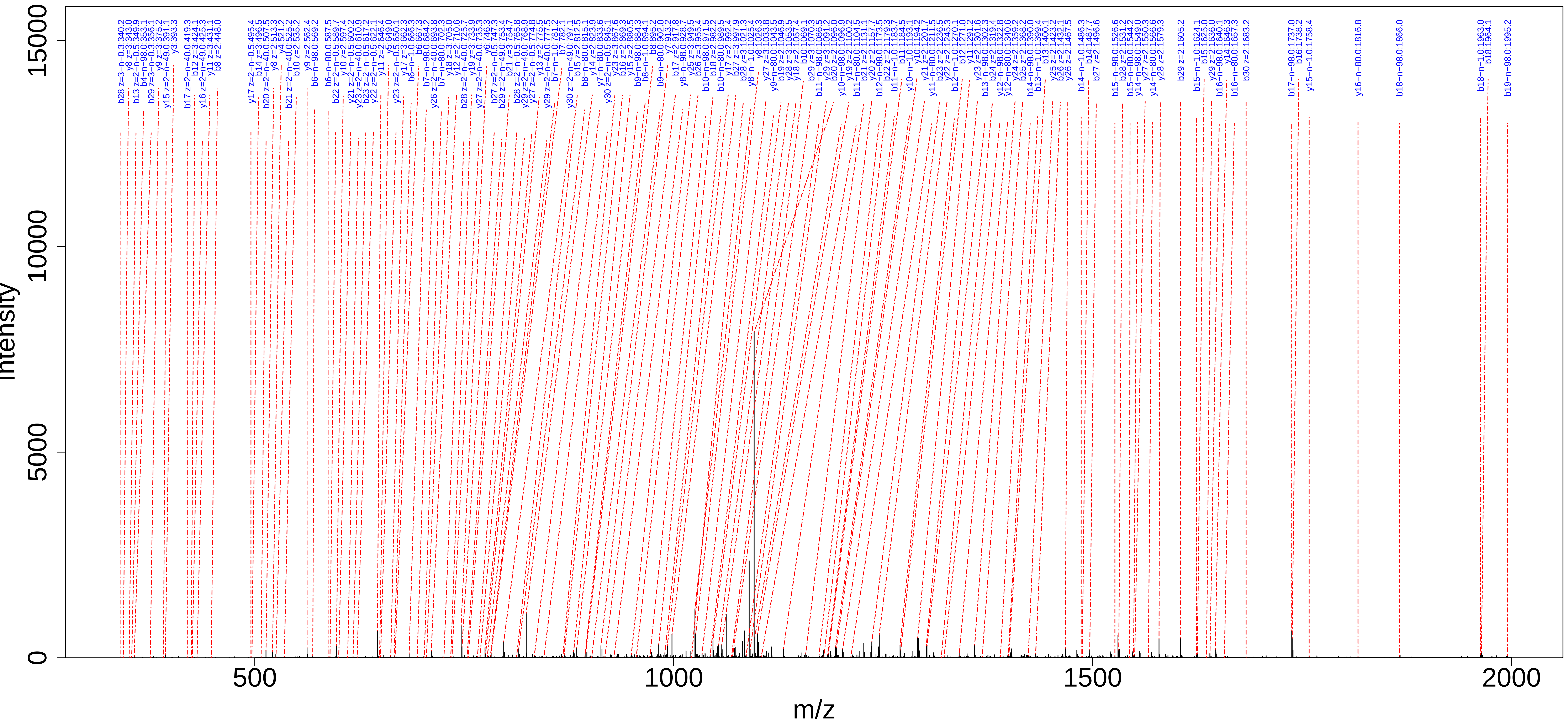

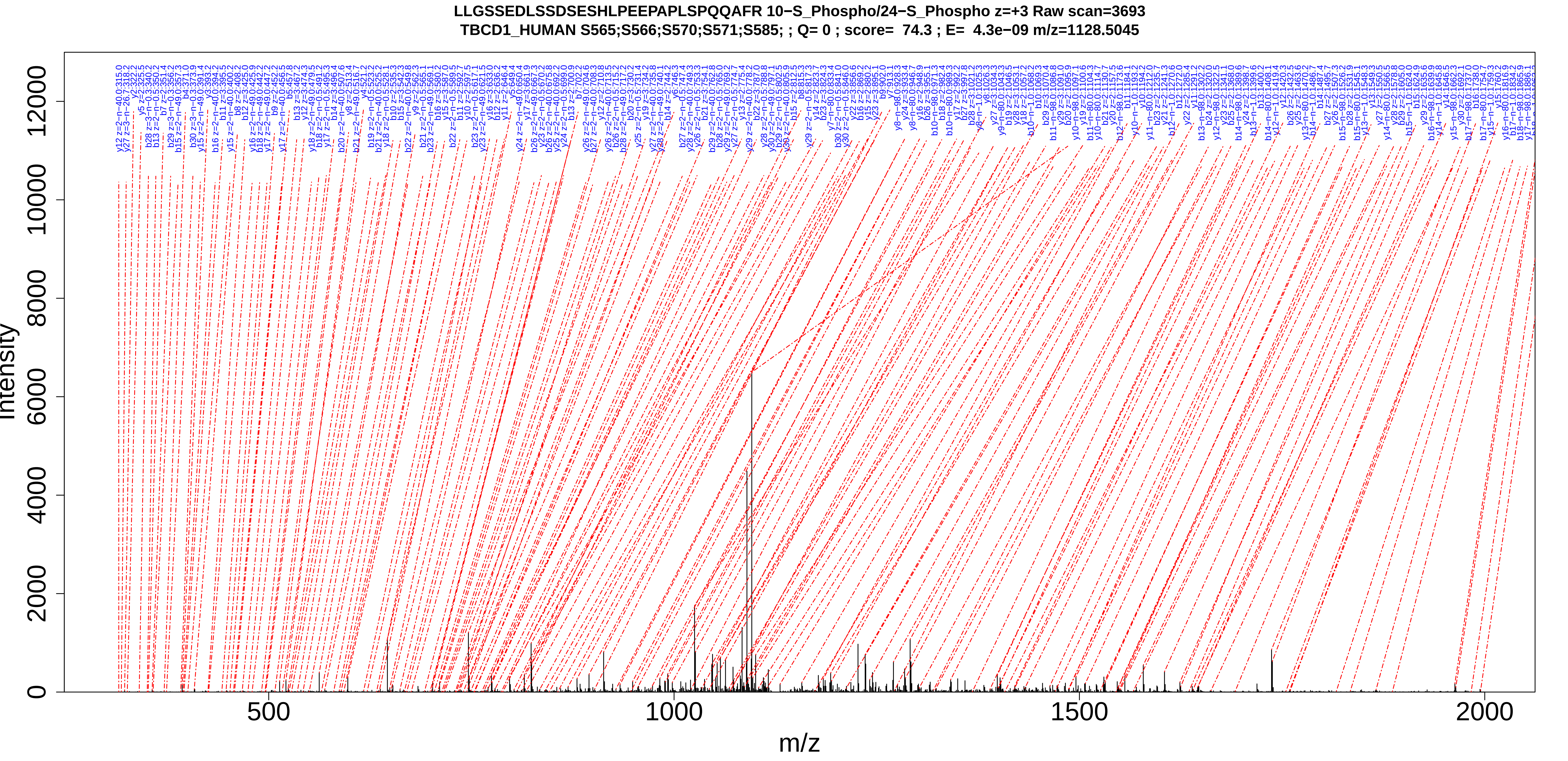
LLGSSEDLSSDSESHLPEEPAPLSPQQAFR 10−S_Phospho/24−S_Phospho z=+3 Raw scan=3693
TBCD1_HUMAN S565;S566;S570;S571;S585; ; Q= 0 ; score= 74.3 ; E= 4.3e−09 m/z=1128.5045
Intensity
m/z

LLGSSEDLSSDSESHLPEEPAPLSPQQAFRR 24−S_Phospho z=+3 Raw scan=3427
TBCD1_HUMAN S570; ; Q= 0 ; score= 71.5 ; E= 8.9e−09 m/z=1153.8817

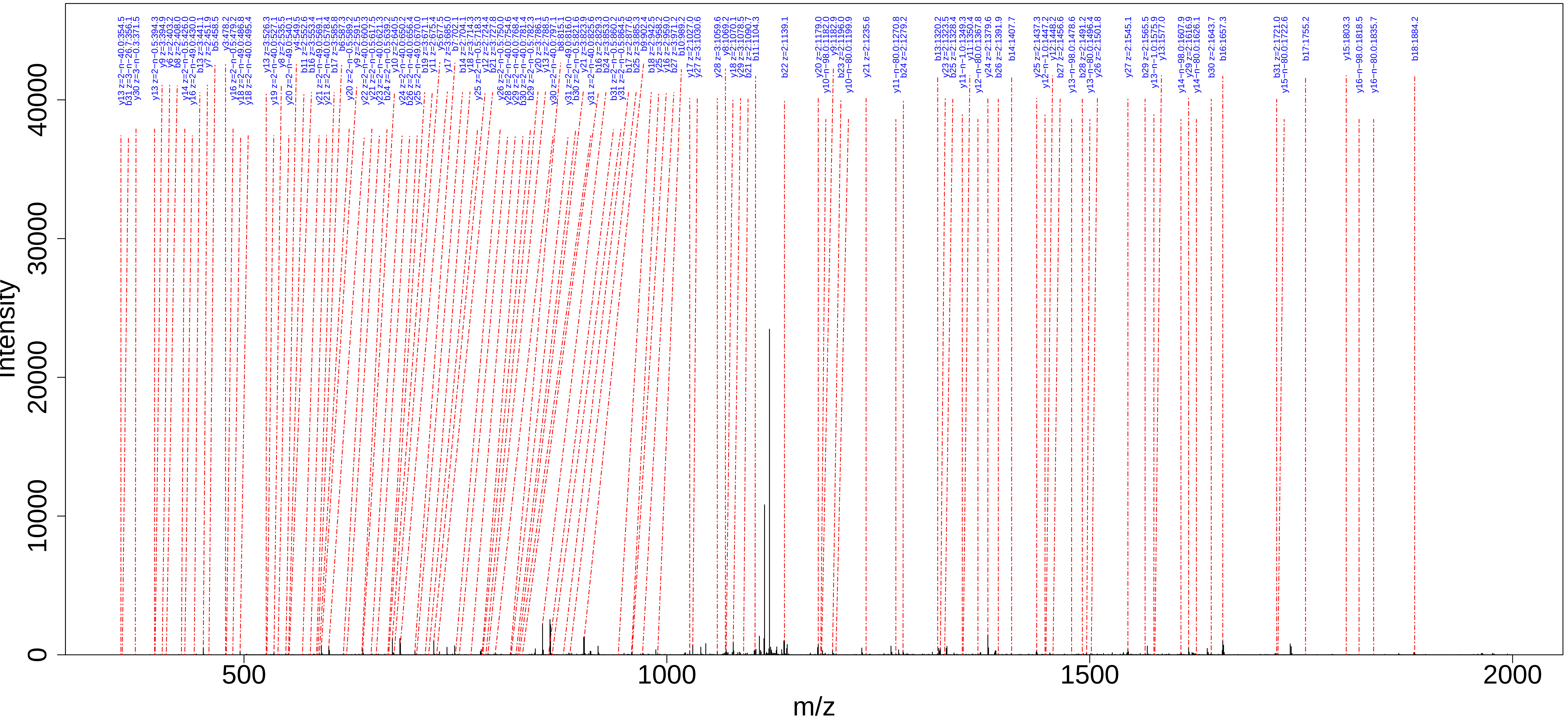

LLGSSEDLSSDSESHLPEEPAPLSPQQAFR 9−S_Phospho/24−S_Phospho z=+3 Raw scan=3679
TBCD1_HUMAN S565;S585; ; Q= 0 ; score= 67.4 ; E= 9.6e−07 m/z=1128.5045

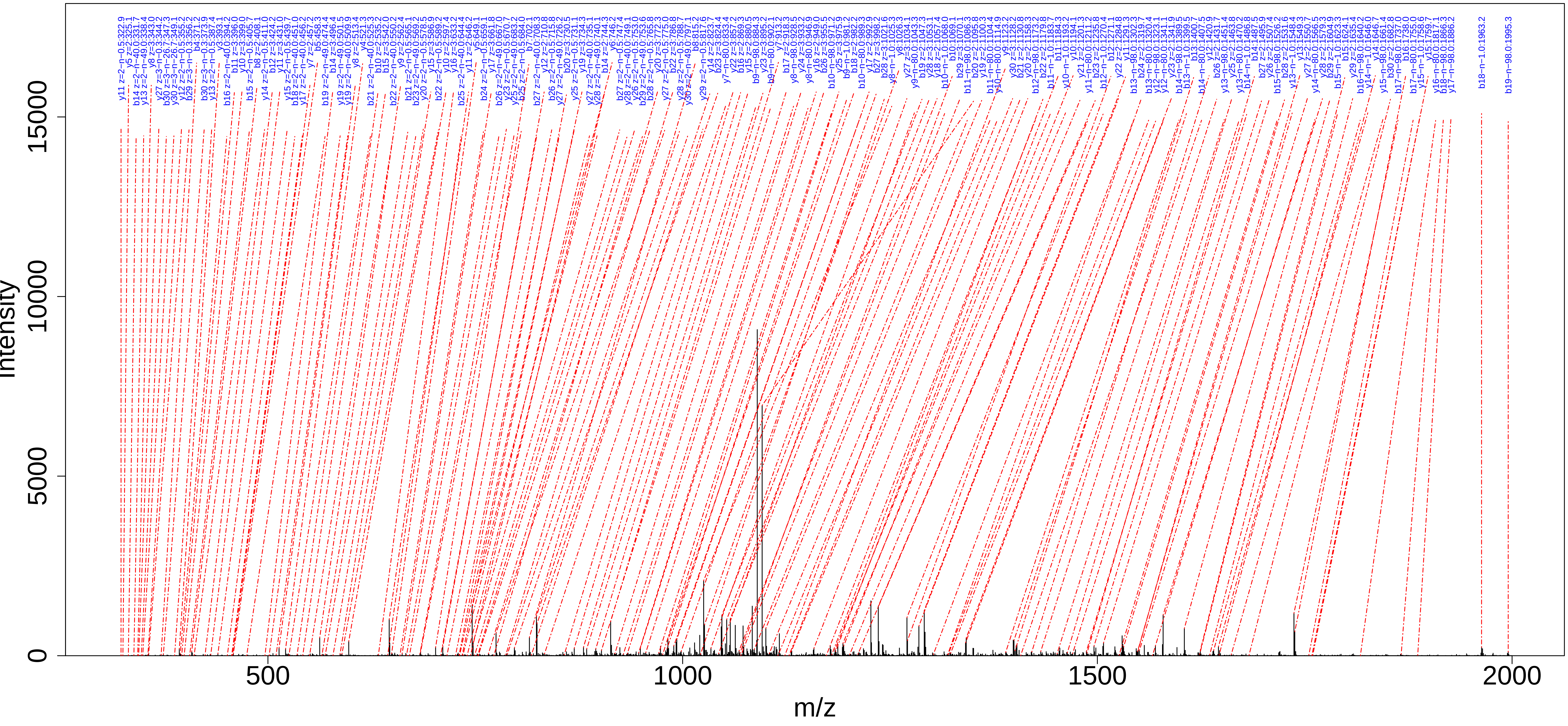

LLGSSEDLSSDSESHLPEEPAPLSPQQAFRR 9−S_Phospho/24−S_Phospho z=+3 Raw scan=3466
TBCD1_HUMAN S566;S585; ; Q= 0 ; score= 60.6 ; E= 1.8e−05 m/z=1180.5322

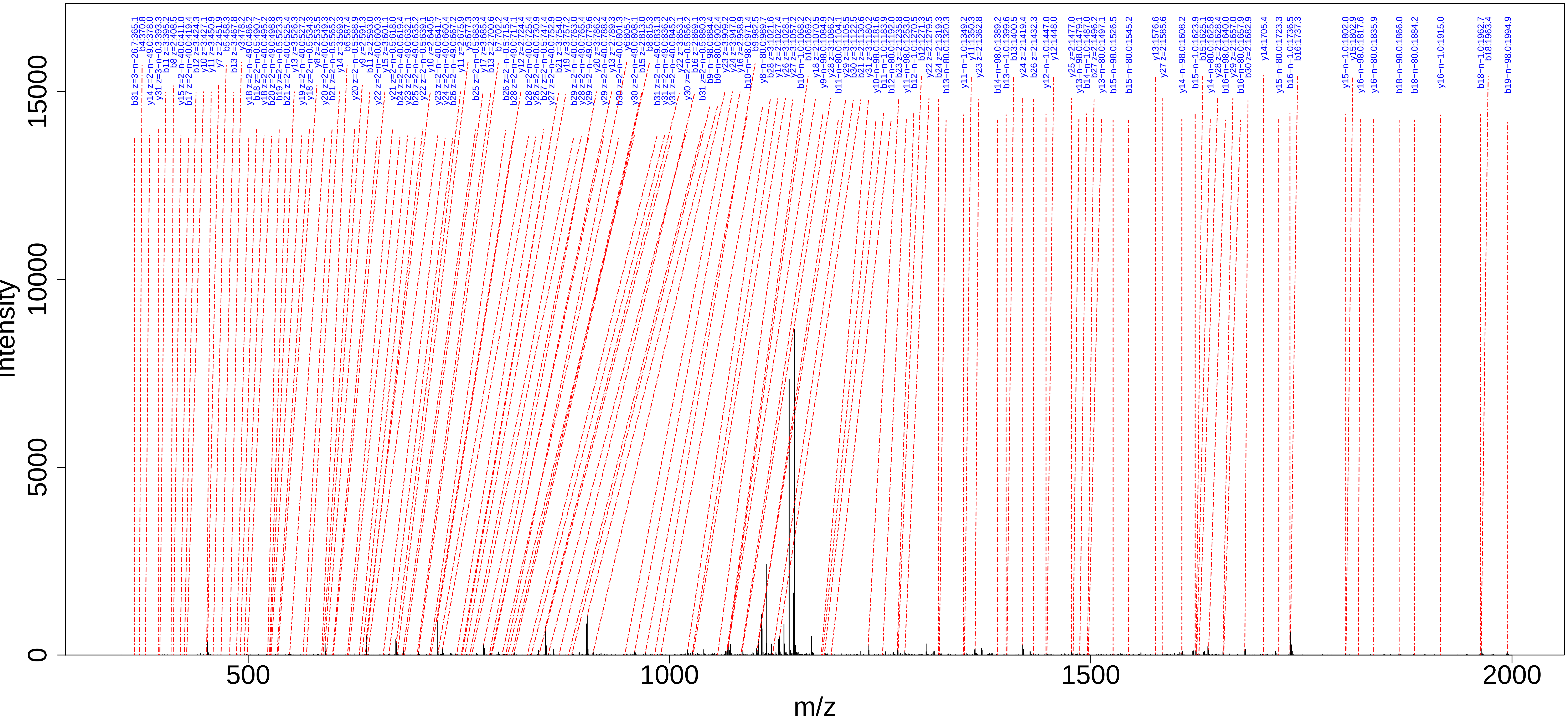

LLGSSEDLSSDSESHLPEEPAPLSPQQAFRR 9−S_Phospho/24−S_Phospho z=+3 Raw scan=3483
TBCD1_HUMAN S565;S585; ; Q= 0 ; score= 57.2 ; E= 4.9e−05 m/z=1180.5349

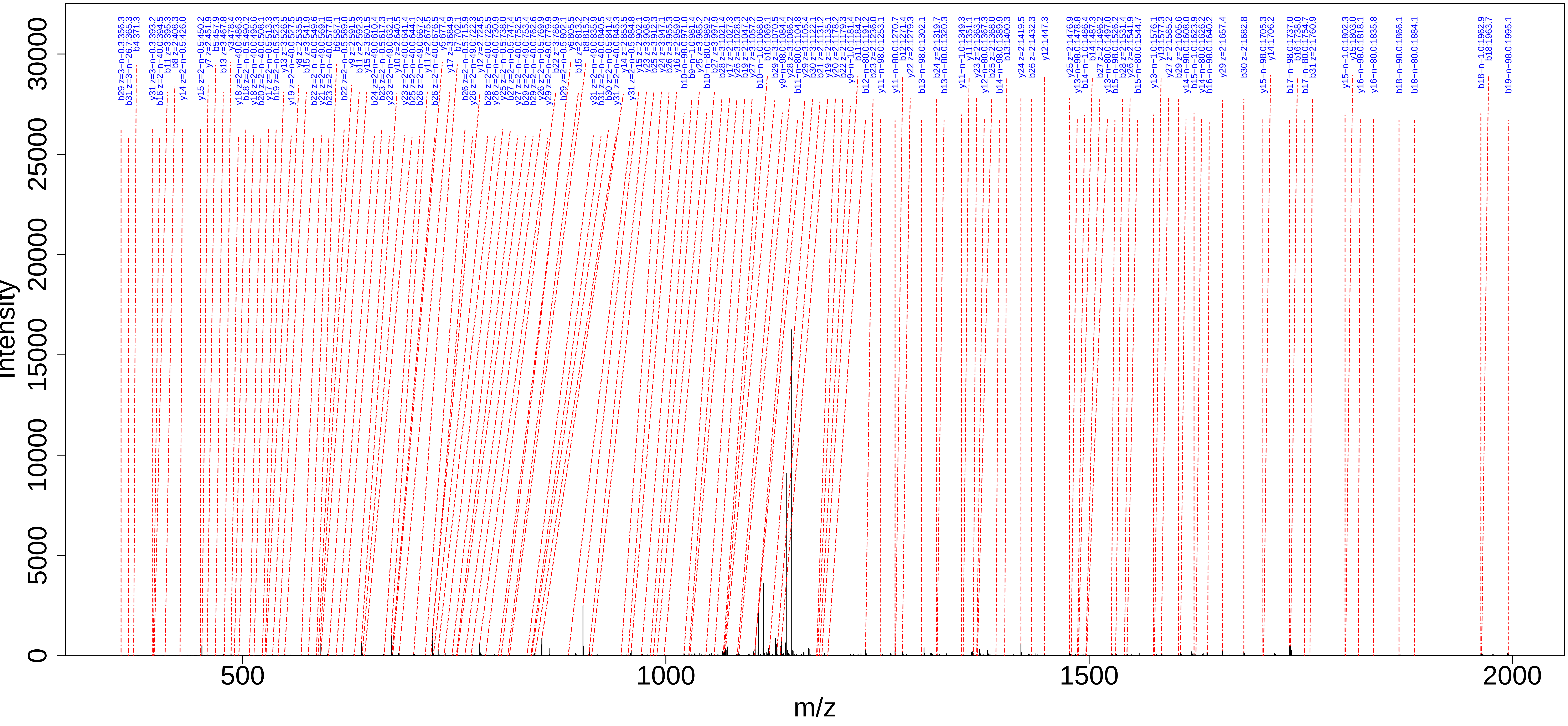

LLGSSEDLSSDSESHLPEEPAPLSPQQAFRR 4−S_Phospho z=+3 Raw scan=3468
TBCD1_HUMAN S570; ; Q= 0 ; score= 48.8 ; E= 4.5e−05 m/z=1153.8726

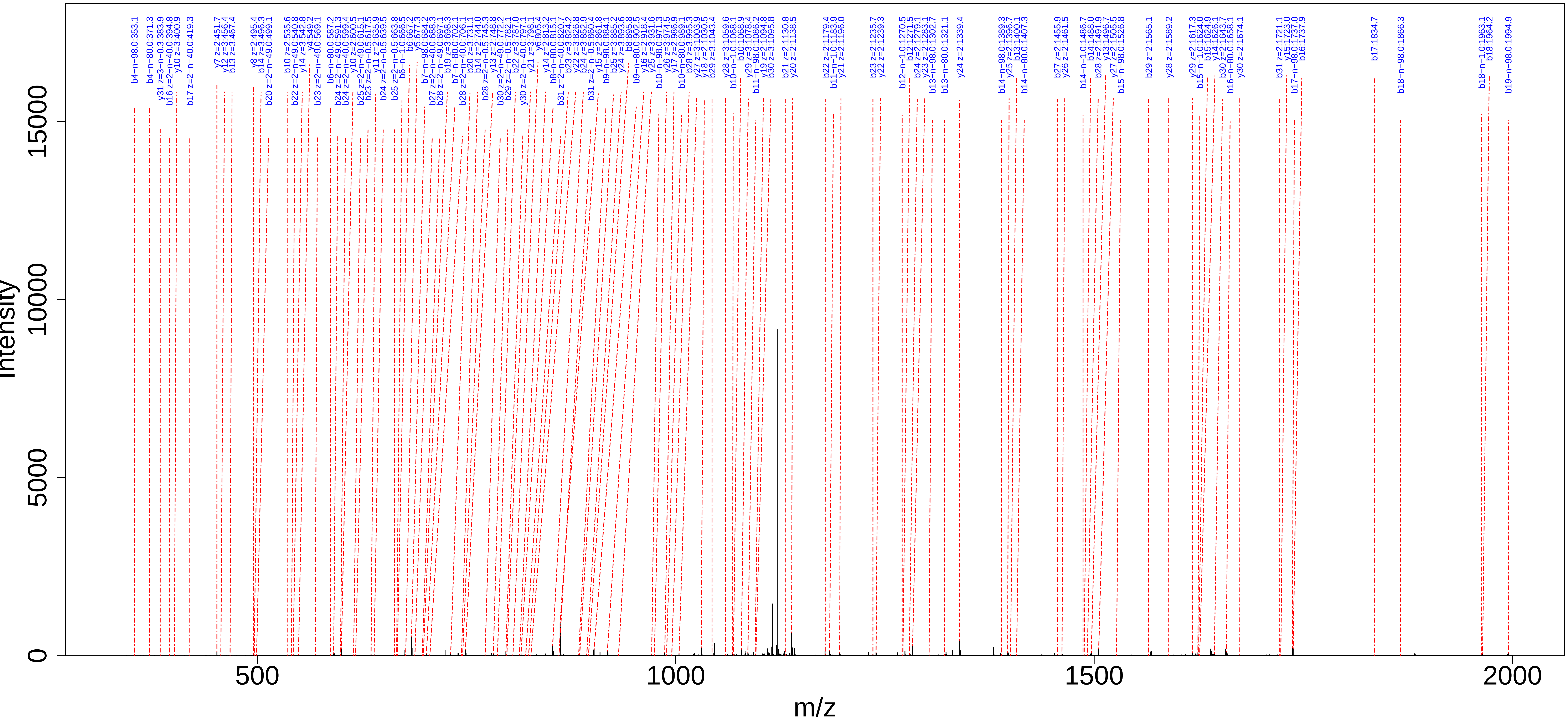

LLGSSEDLSSDSESHLPEEPAPLSPQQAFRR 4−S_Phospho z=+3 Raw scan=3457
TBCD1_HUMAN S565; ; Q= 0 ; score= 47.3 ; E= 7e−07 m/z=1153.8816

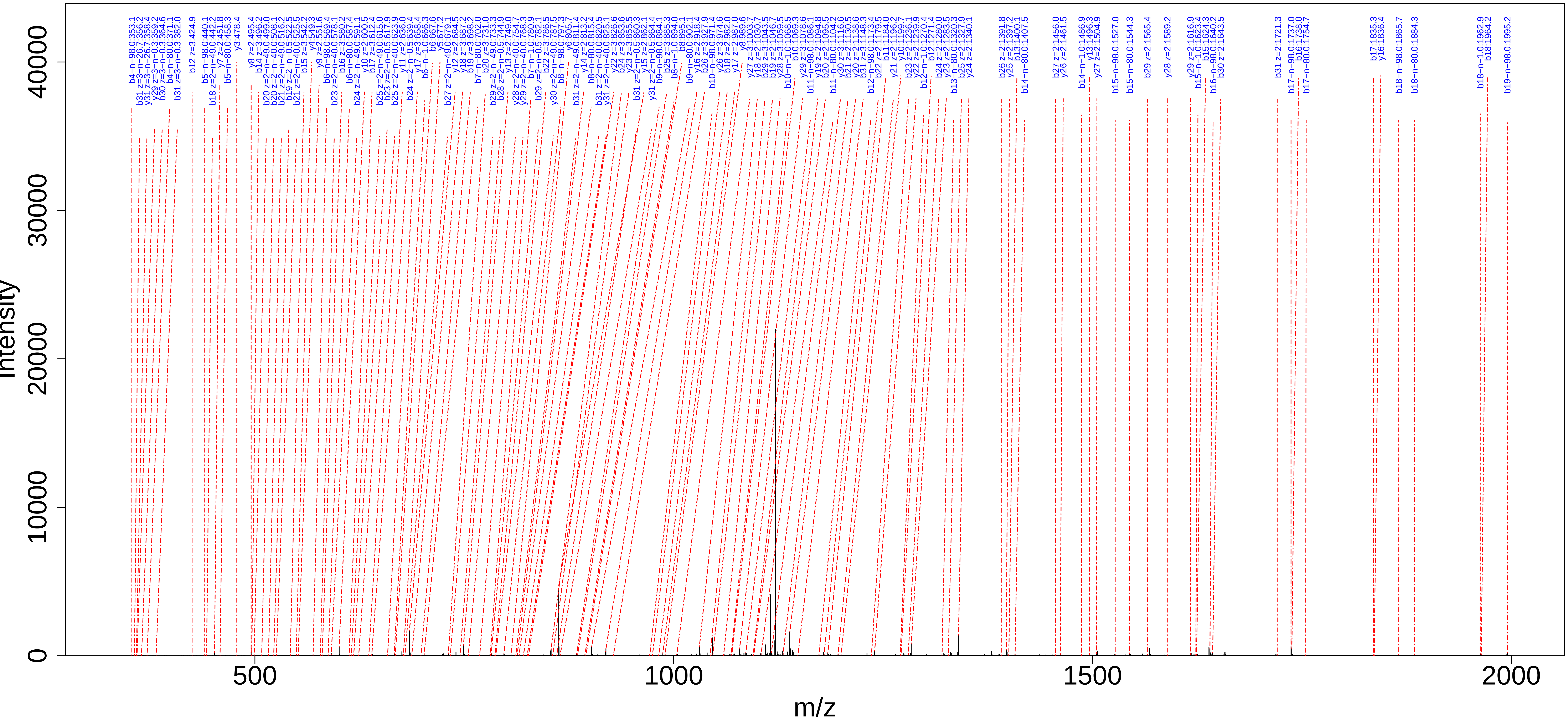

LLGSSEDLSSDSESHLPEEPAPLSPQQAFRR 4−S_Phospho/24−S_Phospho z=+3 Raw scan=3527
TBCD1_HUMAN S566;S585; ; Q= 0 ; score= 46.5 ; E= 0.00088 m/z=1180.5372

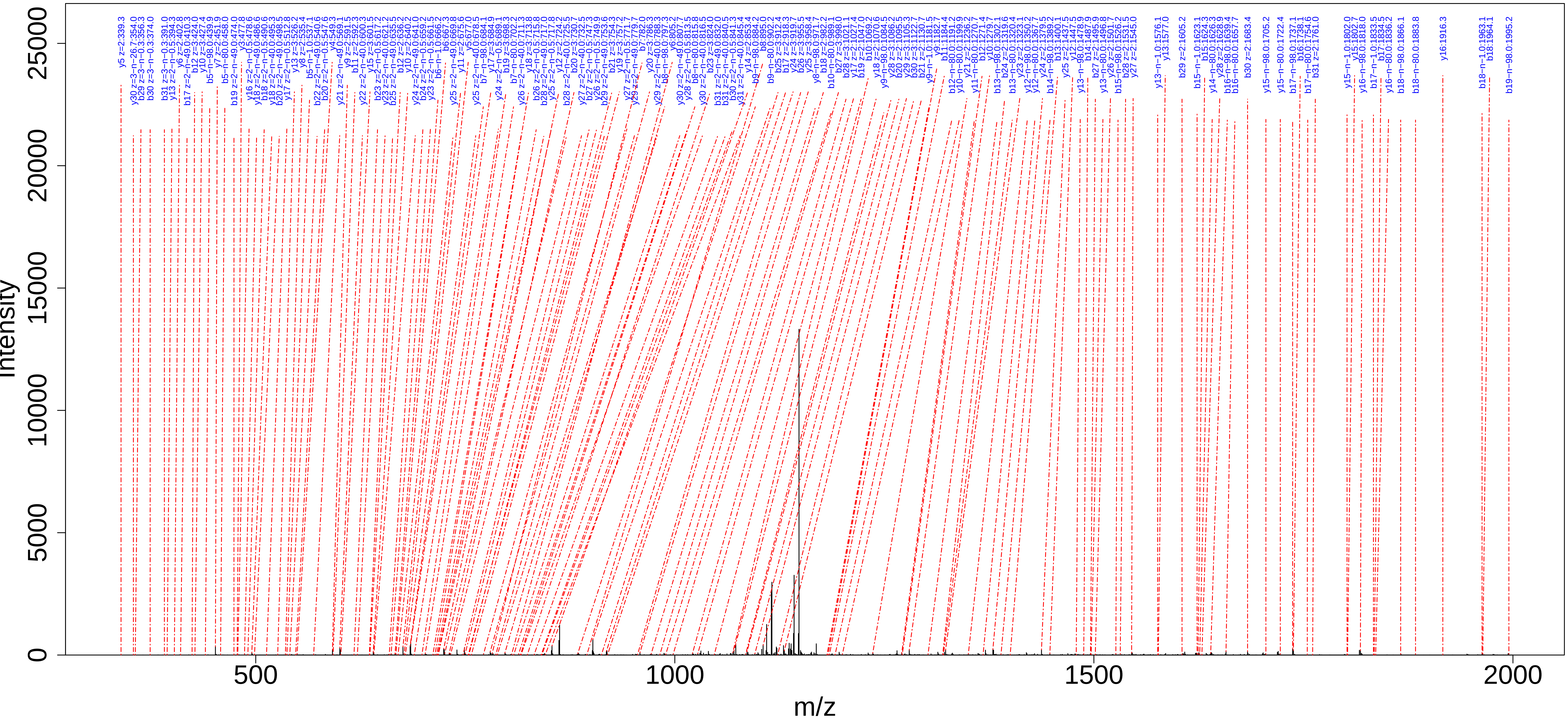

LLGSSEDLSSDSESHLPEEPAPLSPQQAFRR 4−S_Phospho z=+3 Raw scan=3449
TBCD1_HUMAN S570; ; Q= 0 ; score= 45.8 ; E= 1.3e−06 m/z=1153.8843

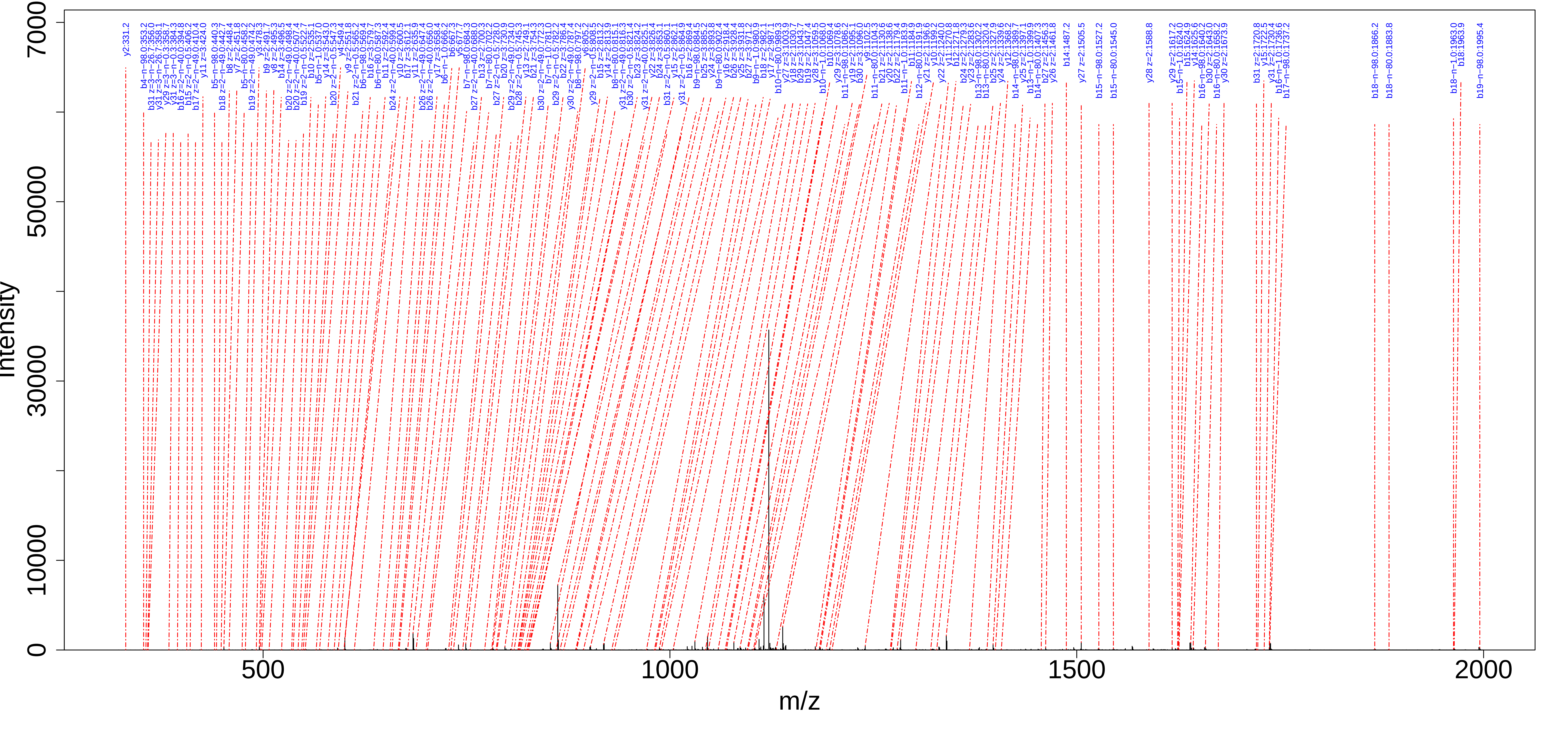

LLGSSEDLSSDSESHLPEEPAPLSPQQAFRR 4−S_Phospho/24−S_Phospho z=+3 Raw scan=3508
TBCD1_HUMAN S566;S585; ; Q= 0 ; score= 41.3 ; E= 6.2e−05 m/z=1180.5388

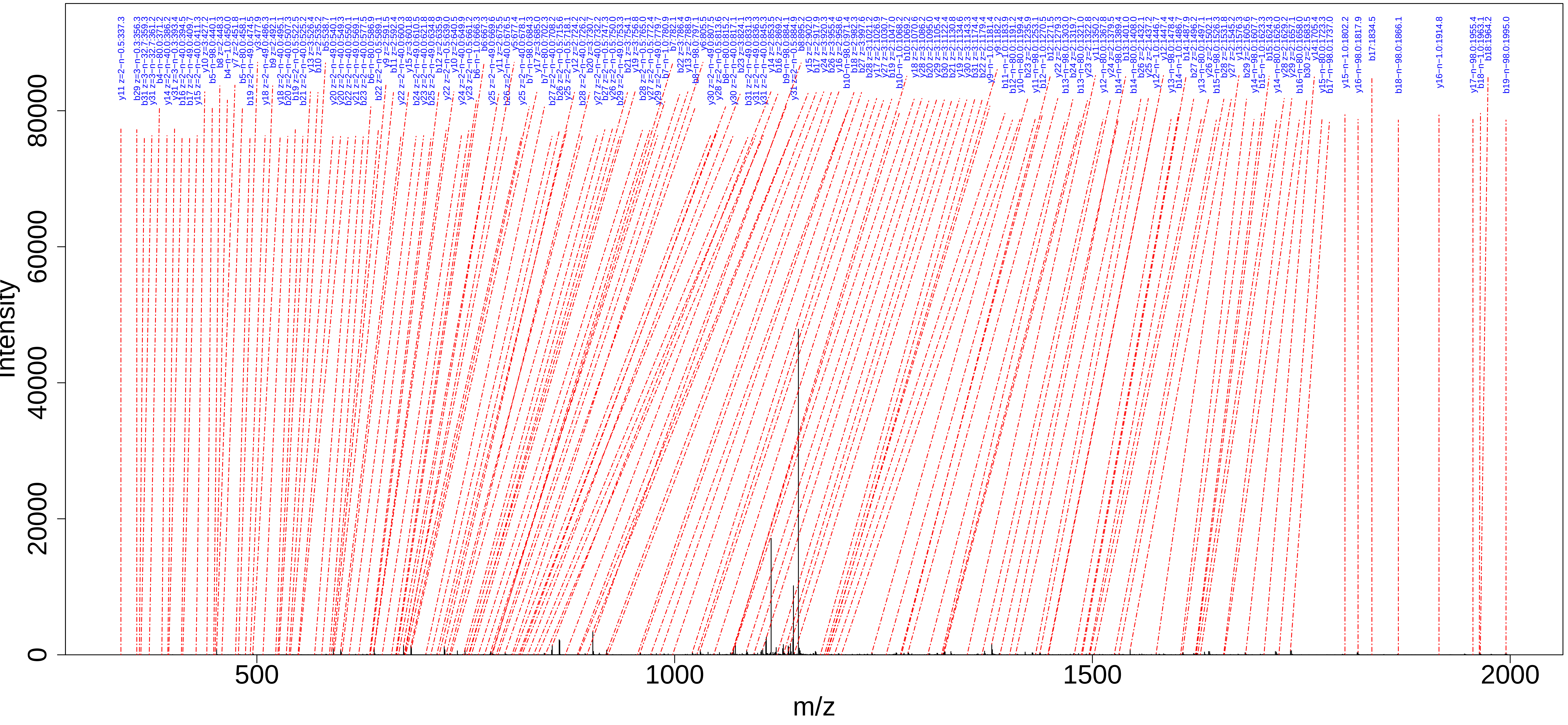

LLGSSEDLSSDSESHLPEEPAPLSPQQAFRR 4−S_Phospho/9−S_Phospho z=+3 Raw scan=3517
TBCD1_HUMAN S565;S585; ; Q= 0 ; score= 40.3 ; E= 9.7e−06 m/z=1180.5371

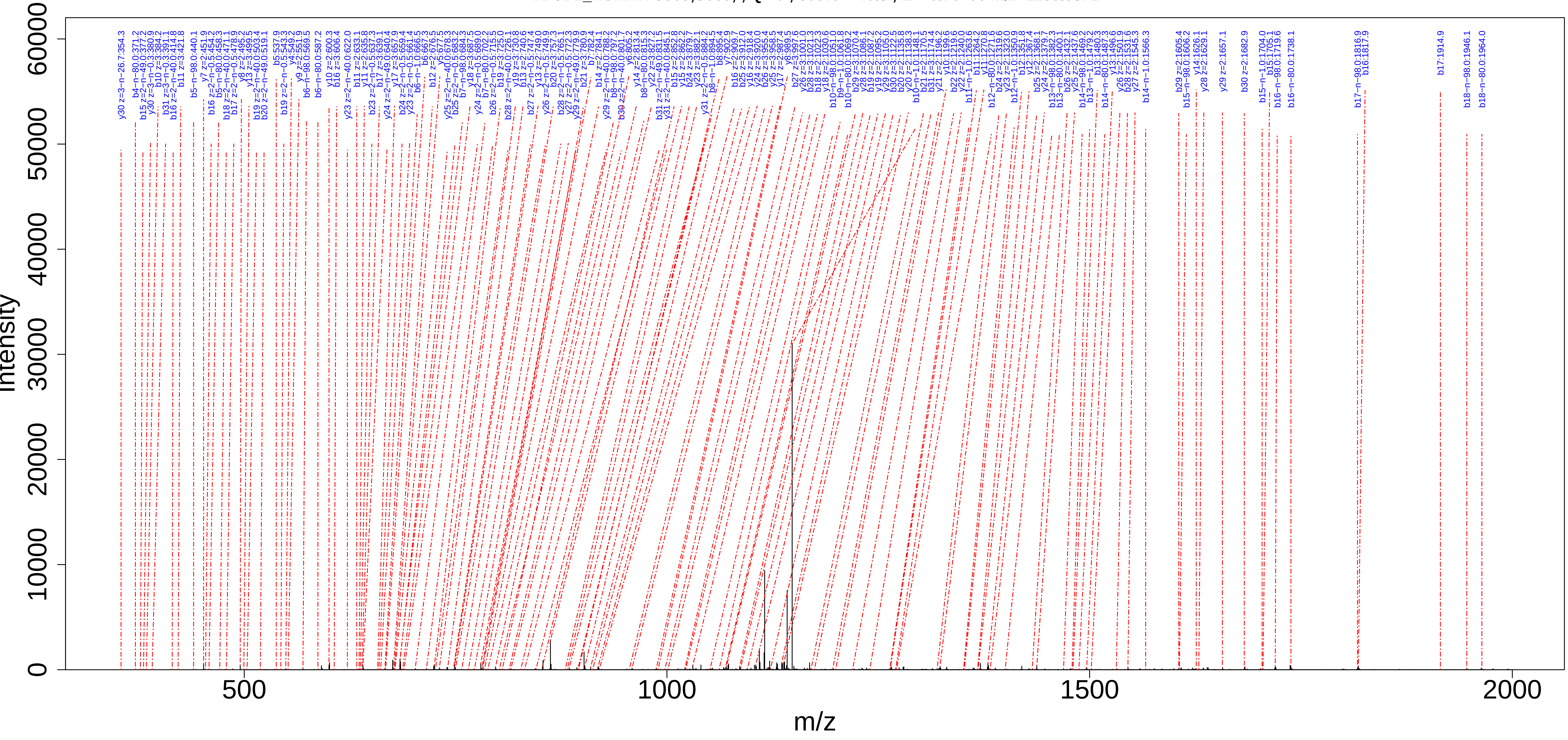

LLGSSEDLSSDSESHLPEEPAPLSPQQAFRR 4−S_Phospho/10−S_Phospho z=+3 Raw scan=3517
TBCD1_HUMAN S565;S585; ; Q= 0 ; score= 40.3 ; E= 9.7e−06 m/z=1180.5371

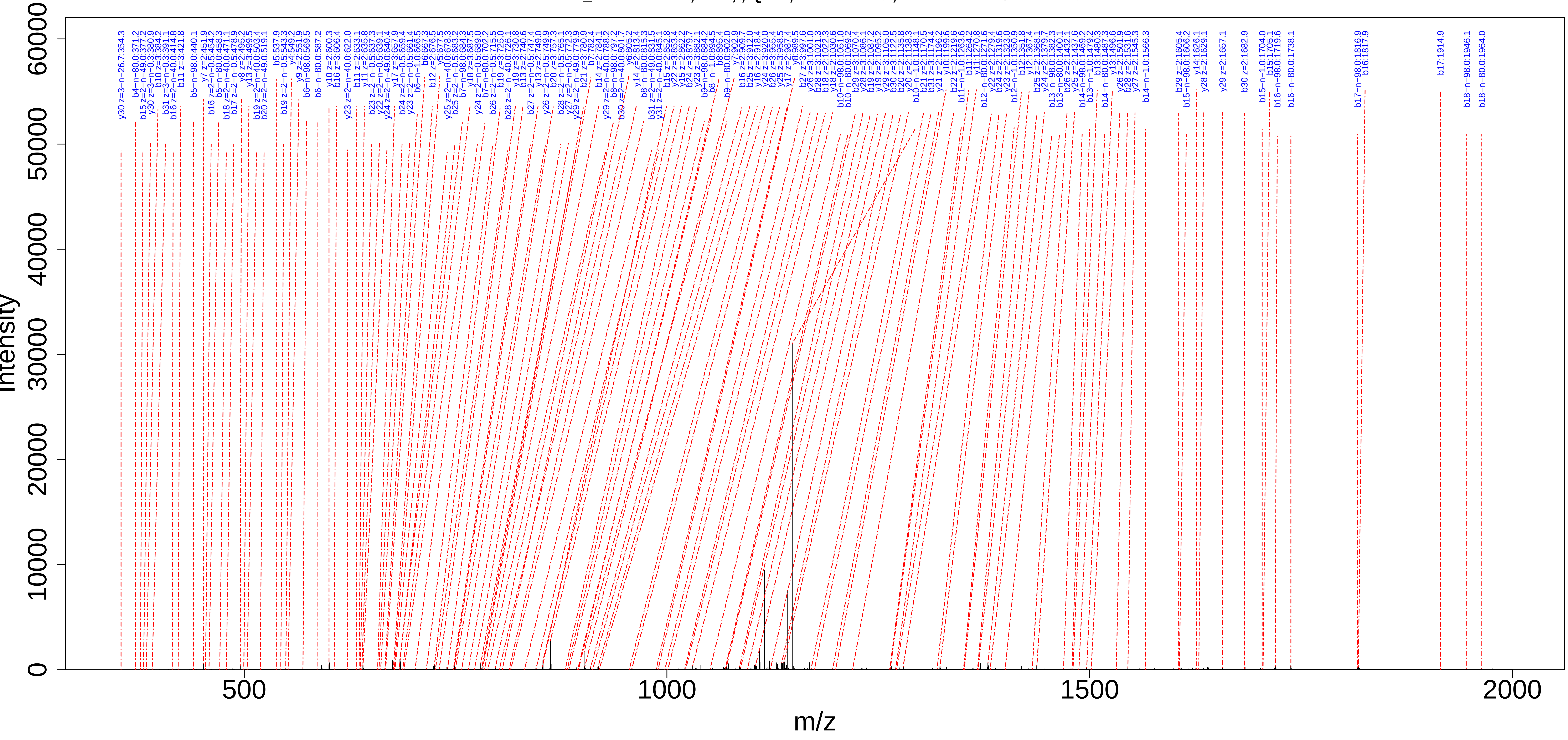

annotated raw MSMS spectra for assay:

Protein kinase: WNK1

Substrate: TBC1D1

sample name: TBCD1_WNK1_phos_3

LLGSSEDLSSDSESHLPEEPAPLSPQQAFR 24−S_Phospho z=+3 Raw scan=3497
TBCD1_HUMAN S566; ; Q= 0 ; score= 114.7 ; E= 1e−15 m/z=1101.844

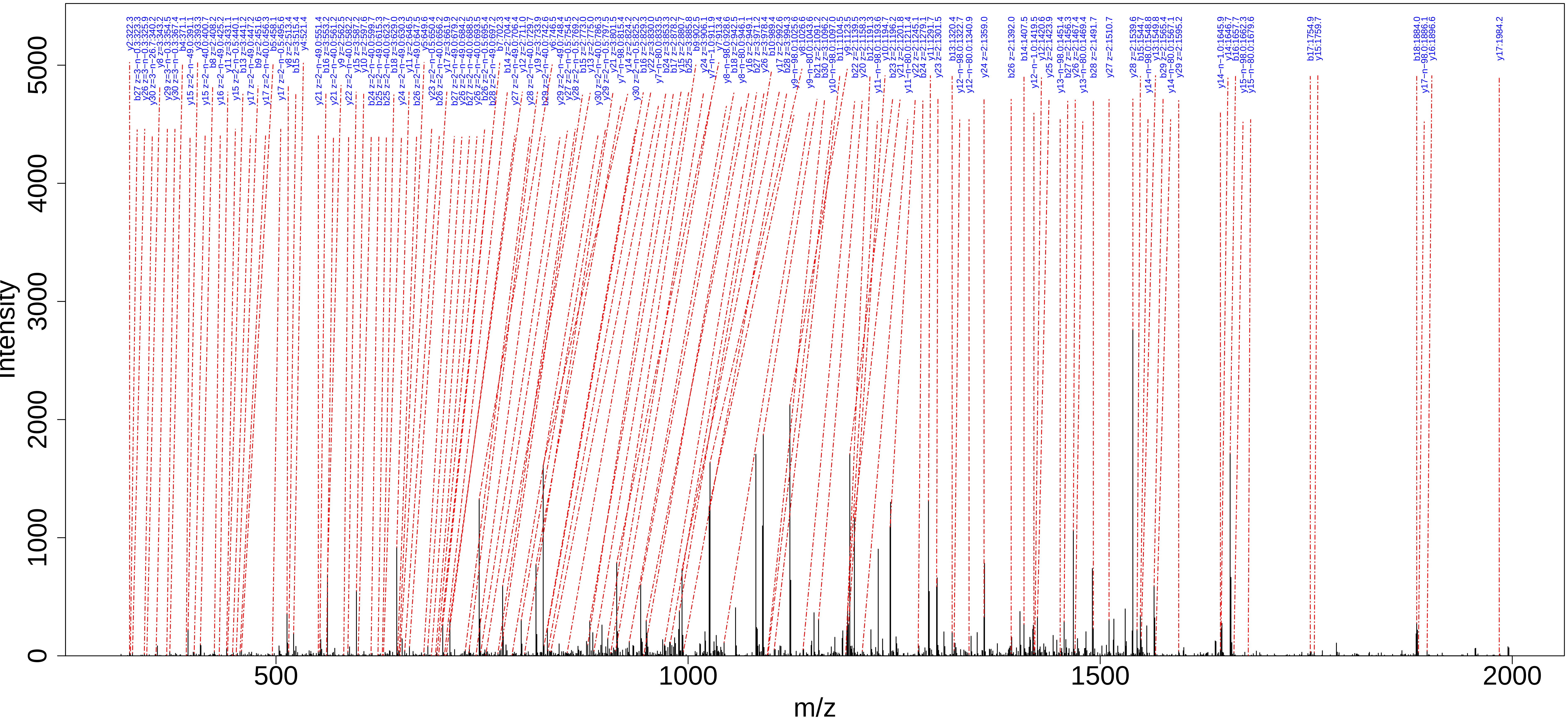

LLGSSEDLSSDSESHLPEEPAPLSPQQAFR 24−S_Phospho z=+3 Raw scan=3503
TBCD1_HUMAN S571; ; Q= 0 ; score= 107.1 ; E= 1e−15 m/z=1101.8424

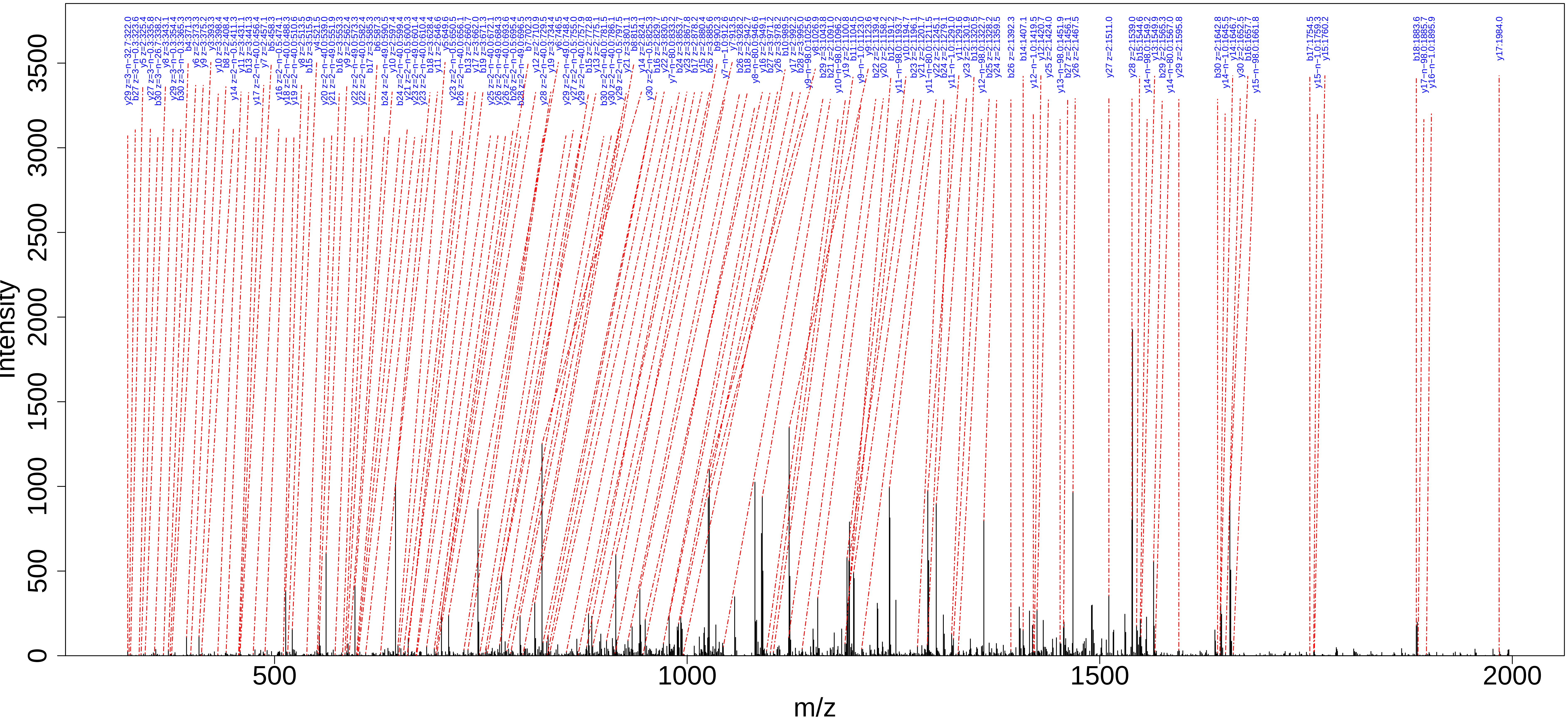

LLGSSEDLSSDSESHLPEEPAPLSPQQAFR 24−S_Phospho z=+3 Raw scan=3518

TBCD1_HUMAN S585; ; Q= 0 ; score= 106.9 ; E= 1e−15 m/z=1101.8441

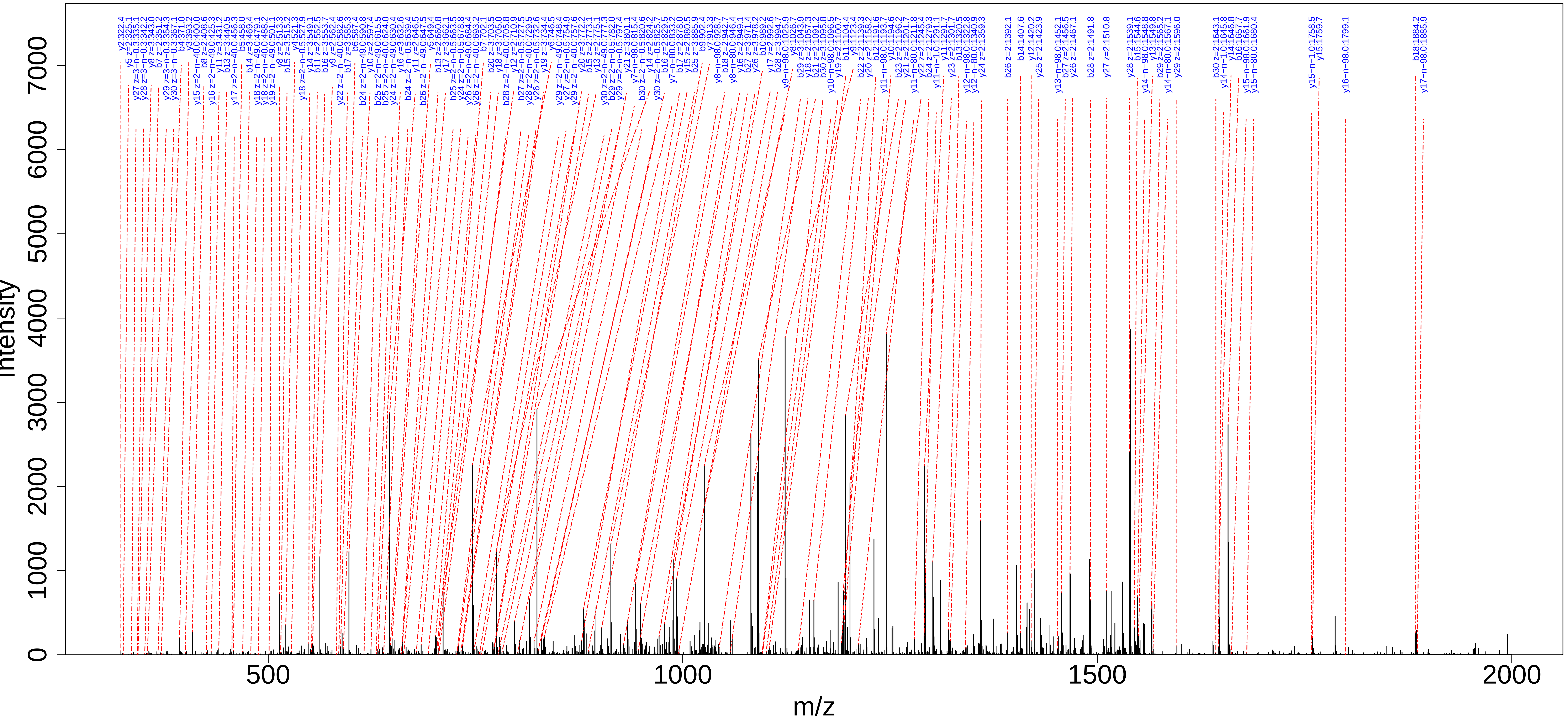

LLGSSEDLSSDSESHLPEEPAPLSPQQAFR 24−S_Phospho z=+3 Raw scan=3512
TBCD1_HUMAN S585; ; Q= 0 ; score= 106.2 ; E= 1e−15 m/z=1101.8419

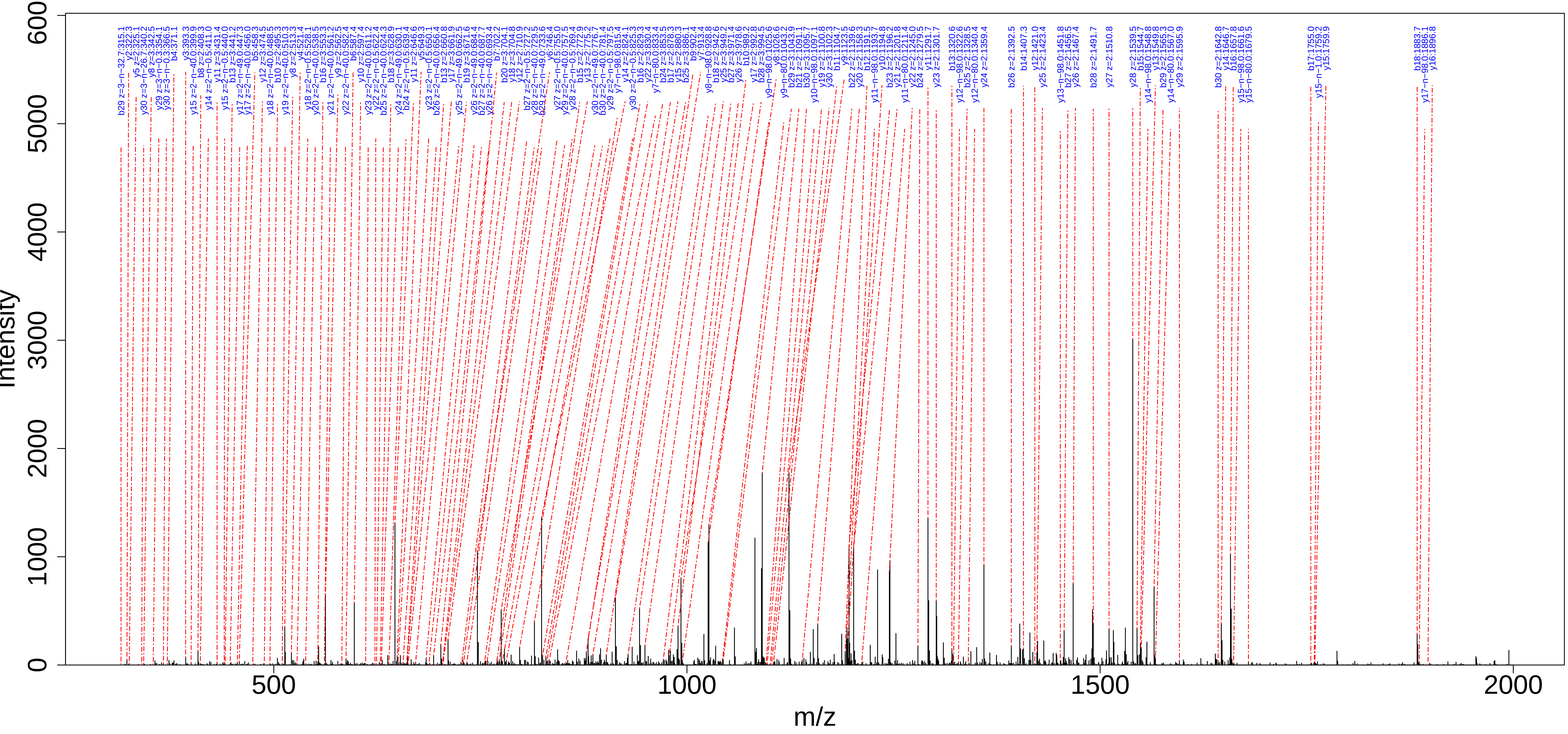

LLGSSEDLSSDSESHLPEEPAPLSPQQAFR 24−S_Phospho z=+3 Raw scan=3525
TBCD1_HUMAN S570; ; Q= 0 ; score= 96.4 ; E= 1e−15 m/z=1101.8438

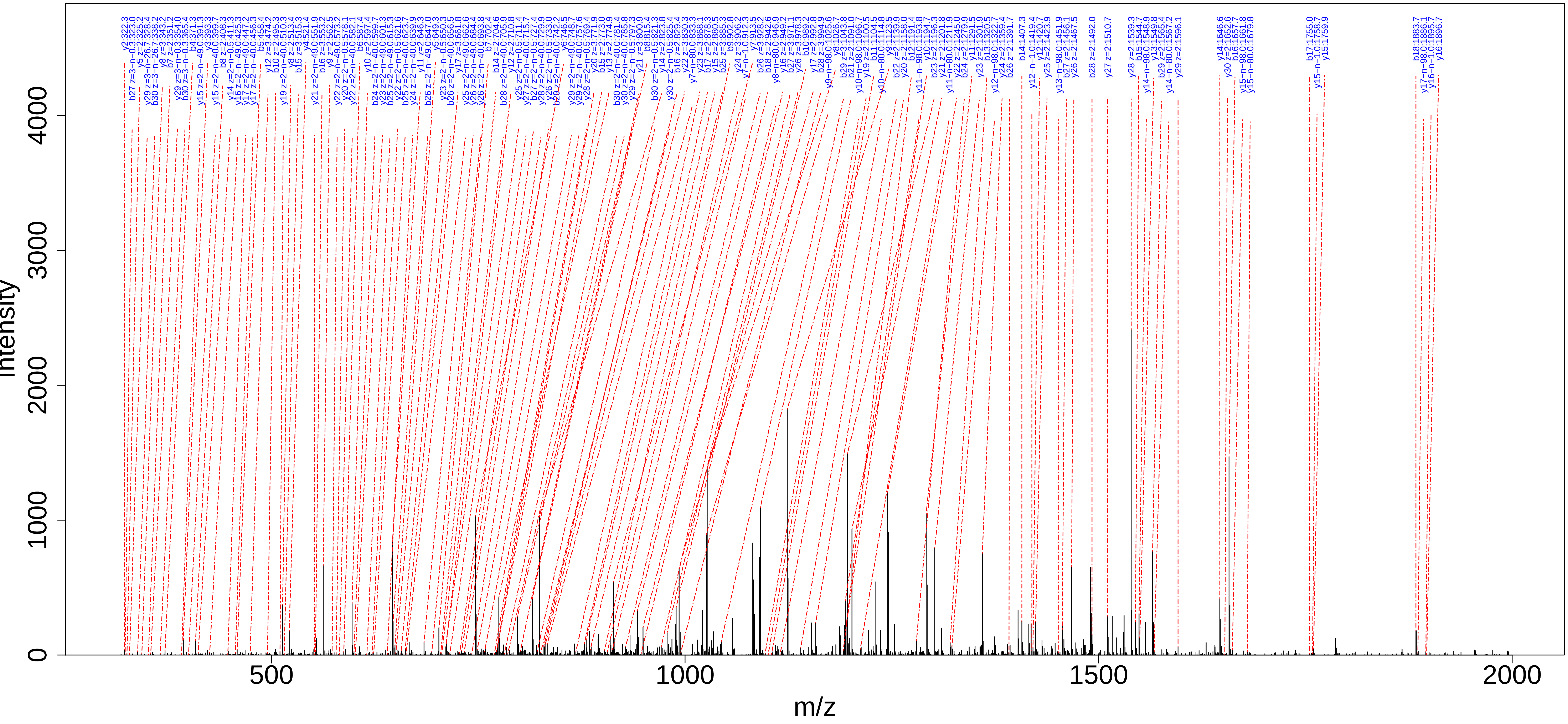

LLGSSEDLSSDSESHLPEEPAPLSPQQAFRR 24−S_Phospho z=+3 Raw scan=3321
TBCD1_HUMAN S570; ; Q= 0 ; score= 82.2 ; E= 1.2e−12 m/z=1153.8773

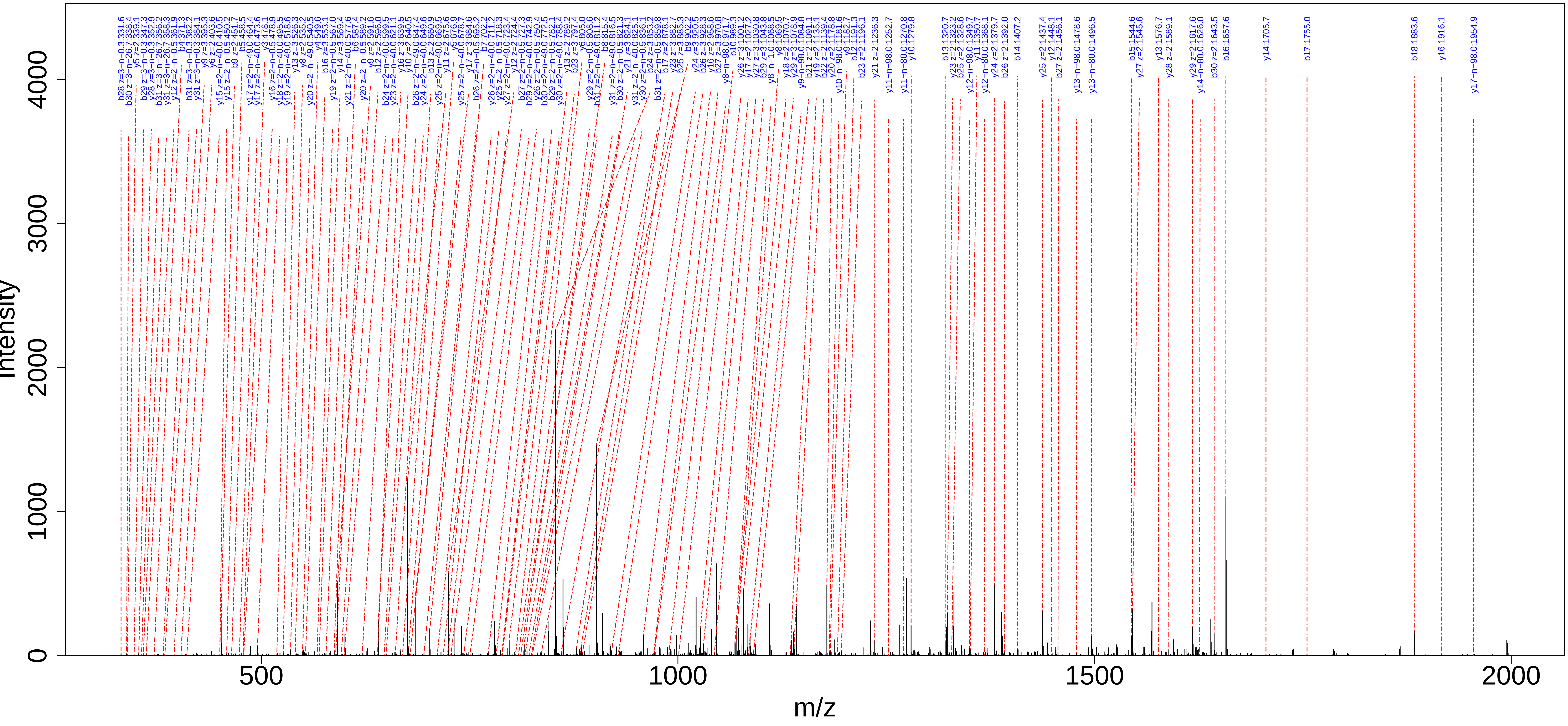

LLGSSEDLSSDSESHLPEEPAPLSPQQAFRR 24−S_Phospho z=+3 Raw scan=3309
TBCD1_HUMAN S570; ; Q= 0 ; score= 77 ; E= 1.4e−08 m/z=1153.8777

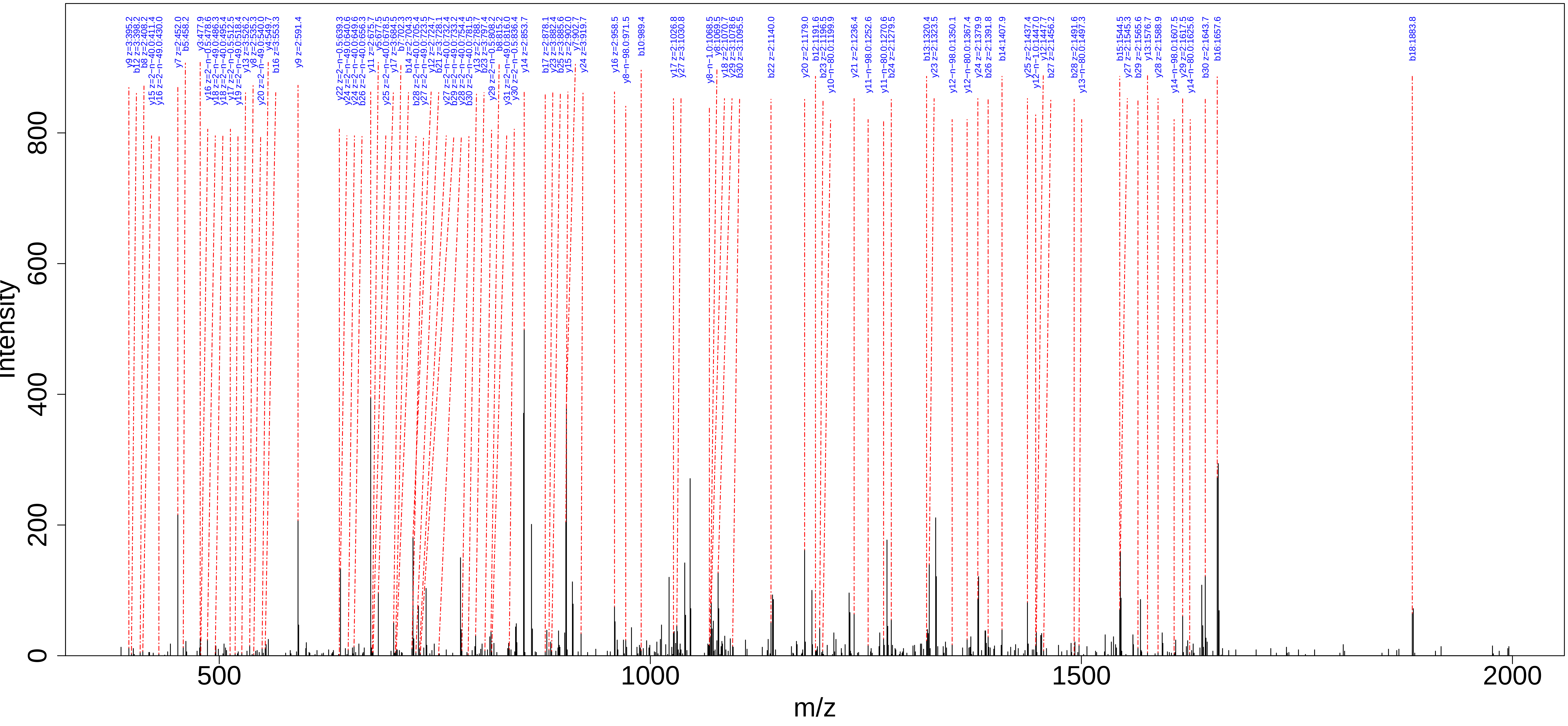

LLGSSEDLSSDSESHLPEEPAPLSPQQAFR 4−S_Phospho z=+3 Raw scan=3582
TBCD1_HUMAN S565; ; Q= 0 ; score= 75.2 ; E= 7.3e−08 m/z=1101.8428

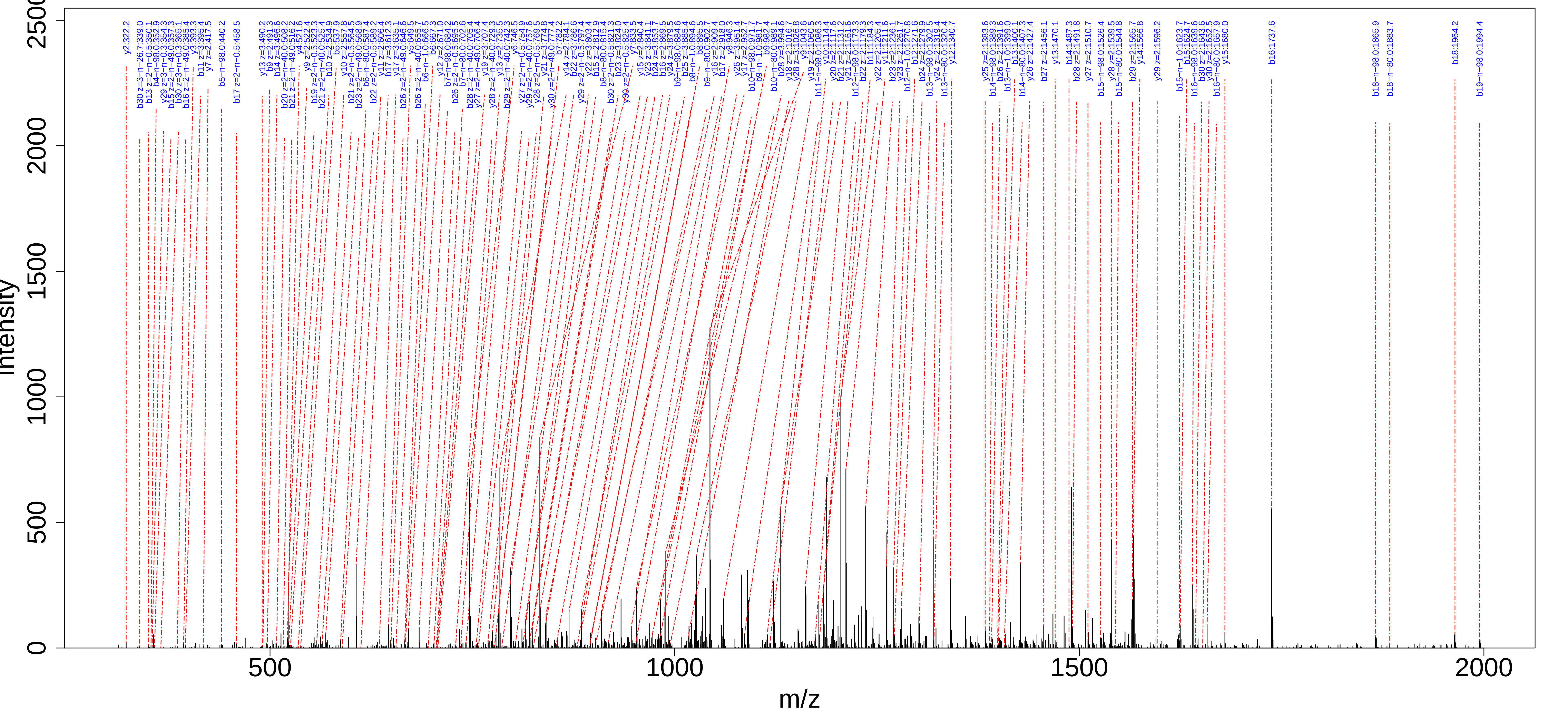

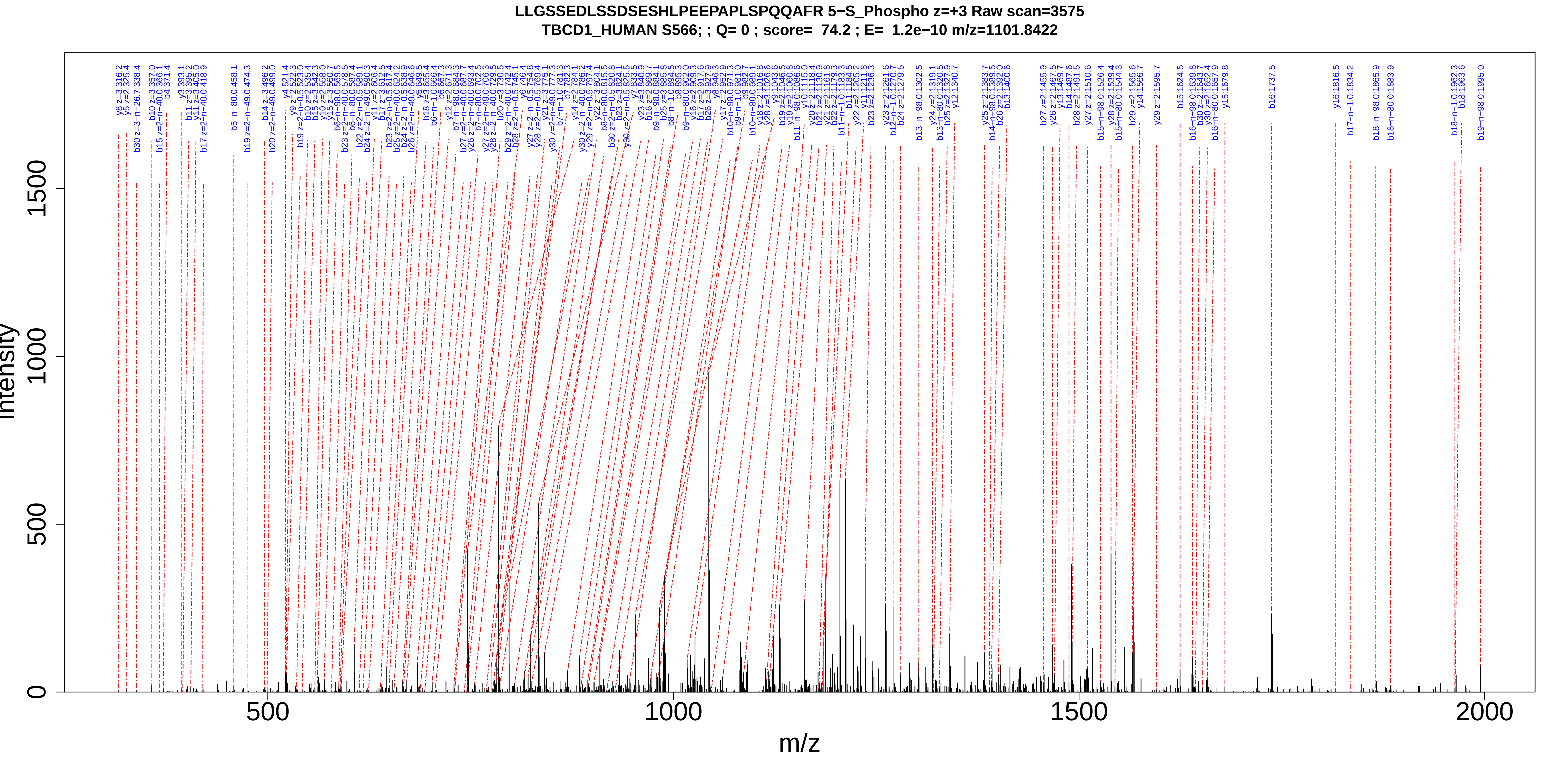
LLGSSEDLSSDSESHLPEEPAPLSPQQAFR 5−S_Phospho z=+3 Raw scan=3575
TBCD1_HUMAN S566; ; Q= 0 ; score= 74.2 ; E= 1.2e−10 m/z=1101.8422
Intensity
0
500
1000
1500
500
1000
1500
2000
m/z

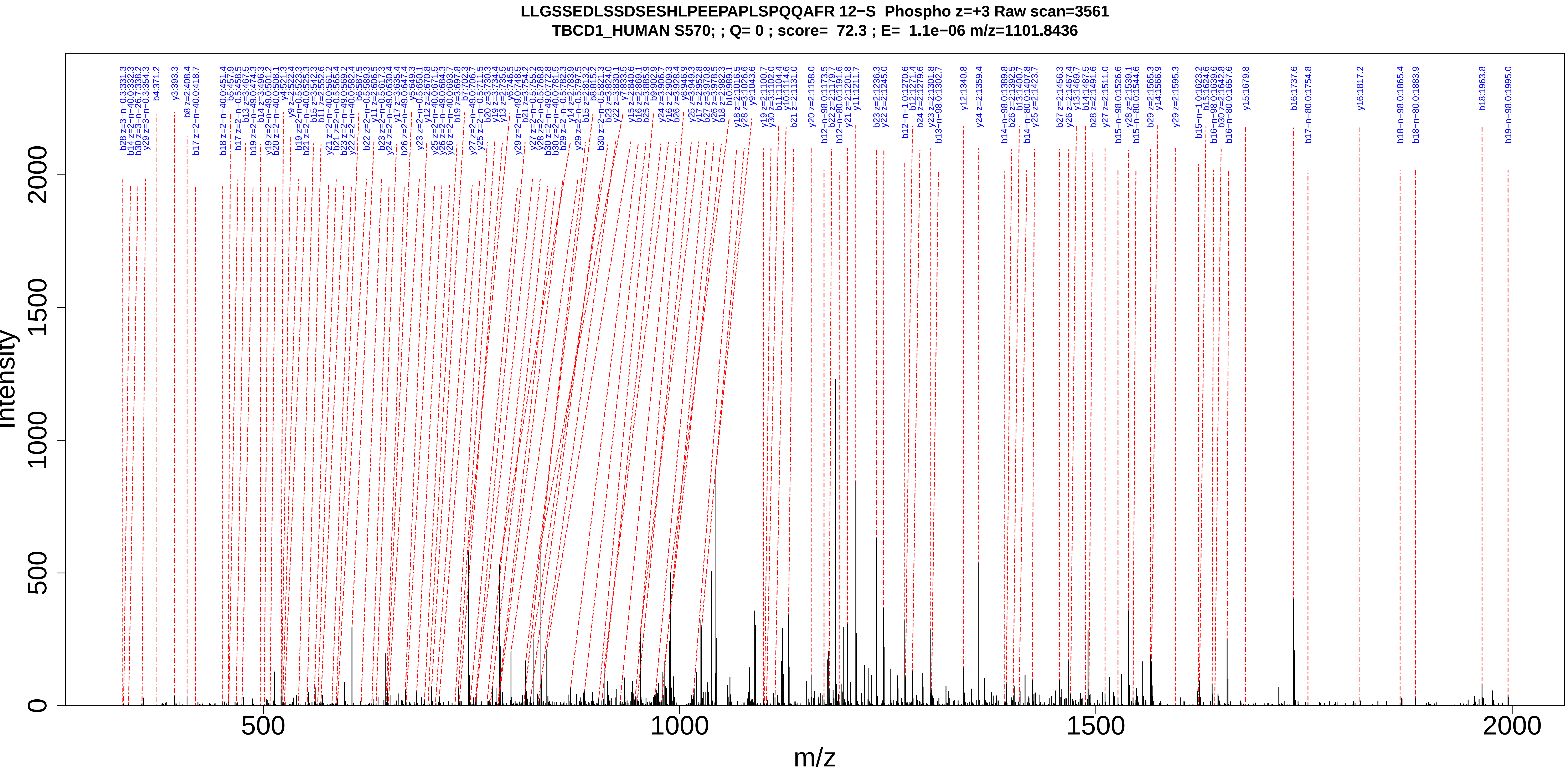
LLGSSEDLSSDSESHLPEEPAPLSPQQAFR 12−S_Phospho z=+3 Raw scan=3561
TBCD1_HUMAN S570; ; Q= 0 ; score= 72.3 ; E= 1.1e−06 m/z=1101.8436
Intensity
m/z
0
500
1000
1500
2000

LLGSSEDLSSDSESHLPEEPAPLSPQQAFR 24−S_Phospho z=+3 Raw scan=3547
TBCD1_HUMAN S570; ; Q= 0 ; score= 69 ; E= 3e−07 m/z=1101.8436

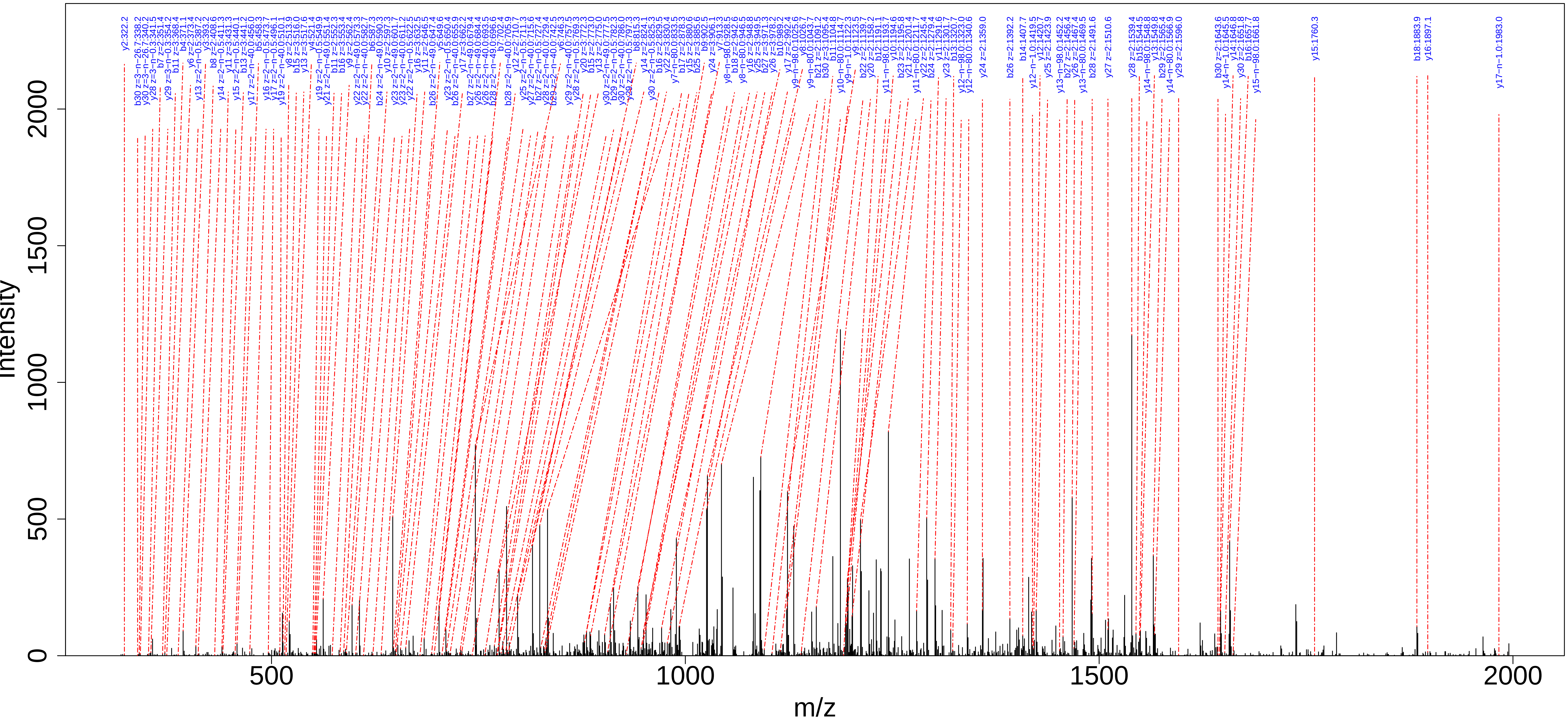

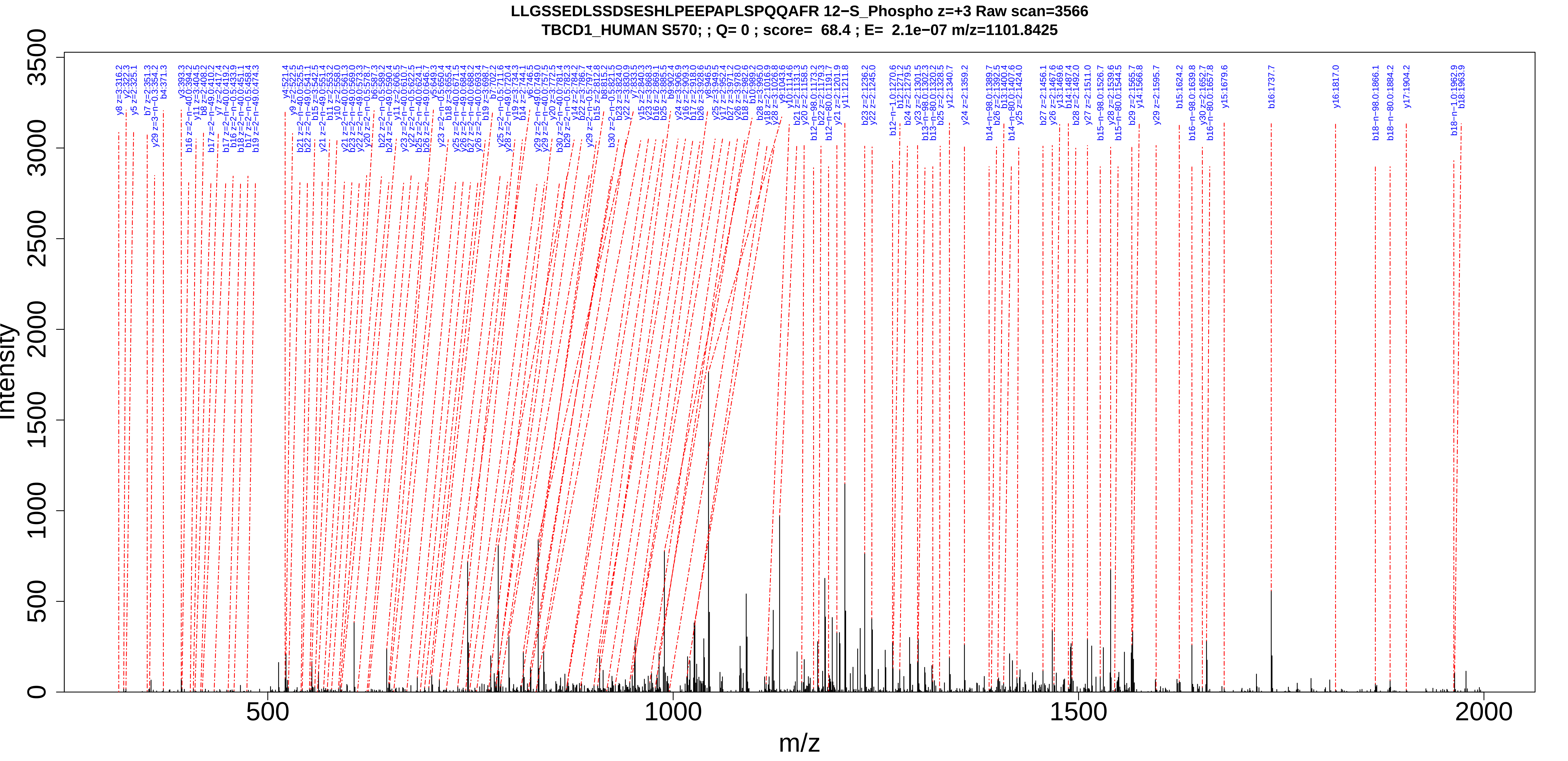
LLGSSEDLSSDSESHLPEEPAPLSPQQAFR 12−S_Phospho z=+3 Raw scan=3566
TBCD1_HUMAN S570; ; Q= 0 ; score= 68.4 ; E= 2.1e−07 m/z=1101.8425
Intensity
m/z
0
500
1000
1500
2000
2500
3000
3500
500
1000
1500
2000

LLGSSEDLSSDSESHLPEEPAPLSPQQAFR 24−S_Phospho z=+3 Raw scan=3535
TBCD1_HUMAN S585; ; Q= 0 ; score= 68.1 ; E= 1e−05 m/z=1101.8381

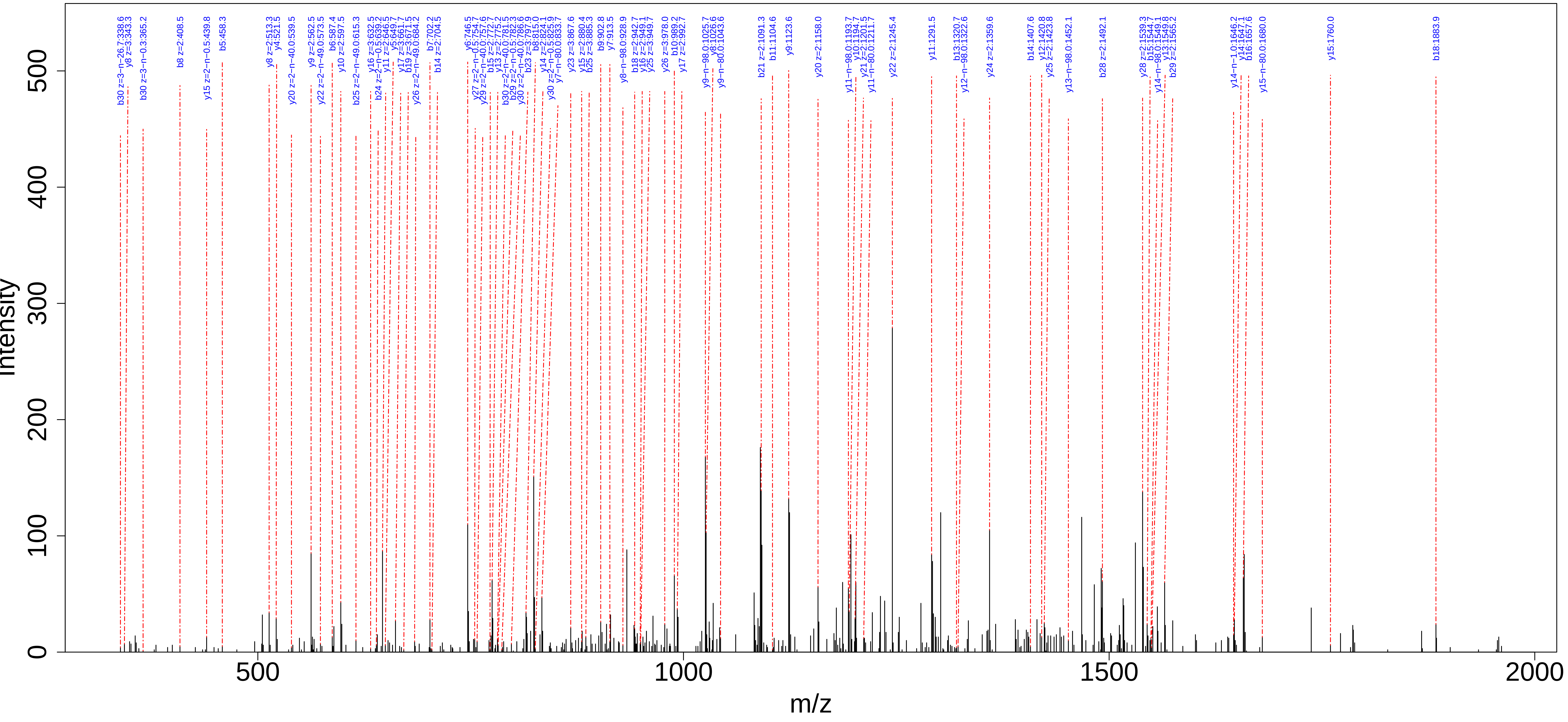

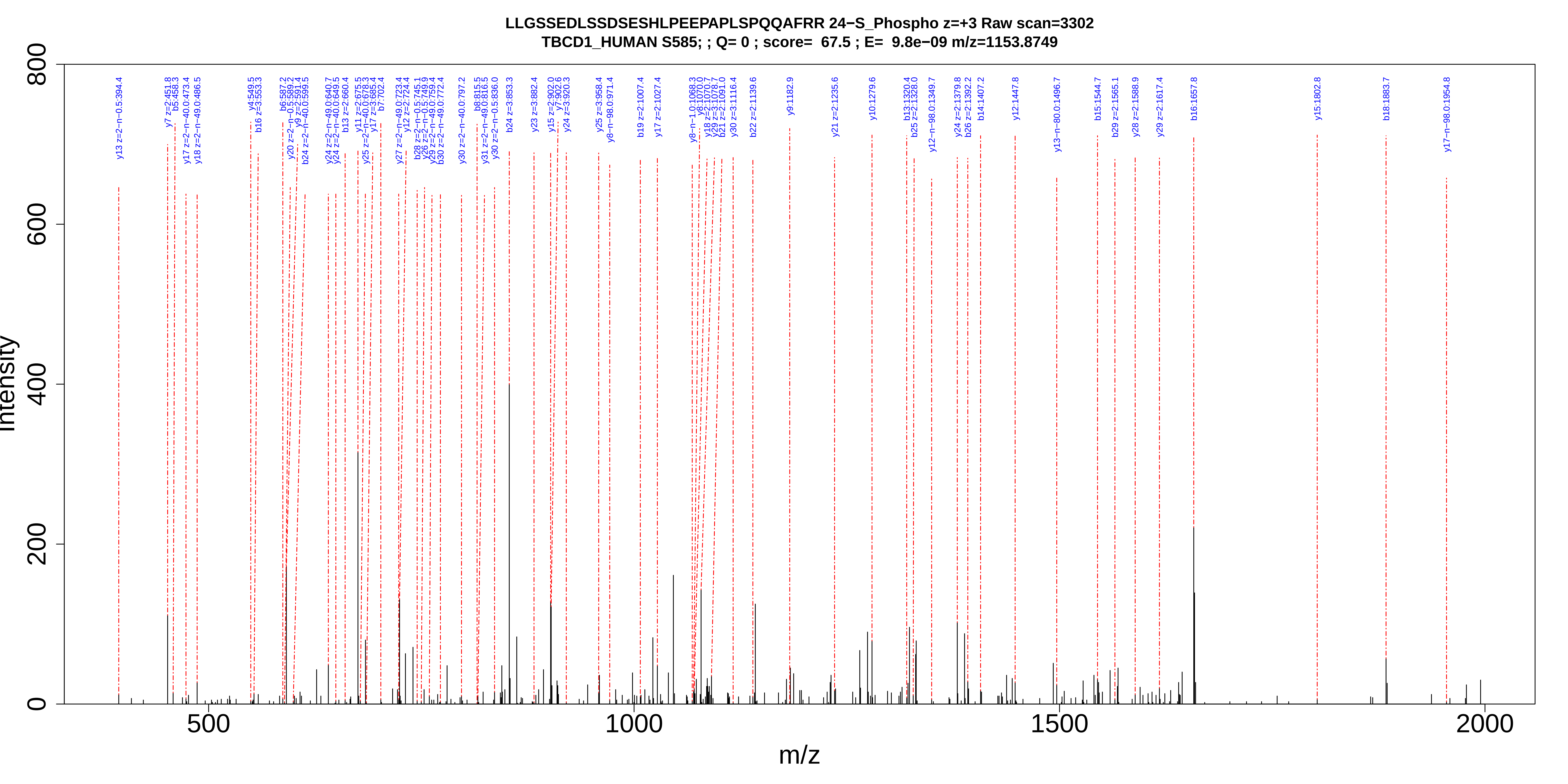
LLGSSEDLSSDSESHLPEEPAPLSPQQAFRR 24−S_Phospho z=+3 Raw scan=3302
TBCD1_HUMAN S585; ; Q= 0 ; score= 67.5 ; E= 9.8e−09 m/z=1153.8749
Intensity
m/z
0
200
400
600
800
500
1000
1500
2000

LLGSSEDLSSDSESHLPEEPAPLSPQQAFRR 4−S_Phospho z=+3 Raw scan=3370
TBCD1_HUMAN S565; ; Q= 0 ; score= 66.7 ; E= 9e−07 m/z=1153.8755

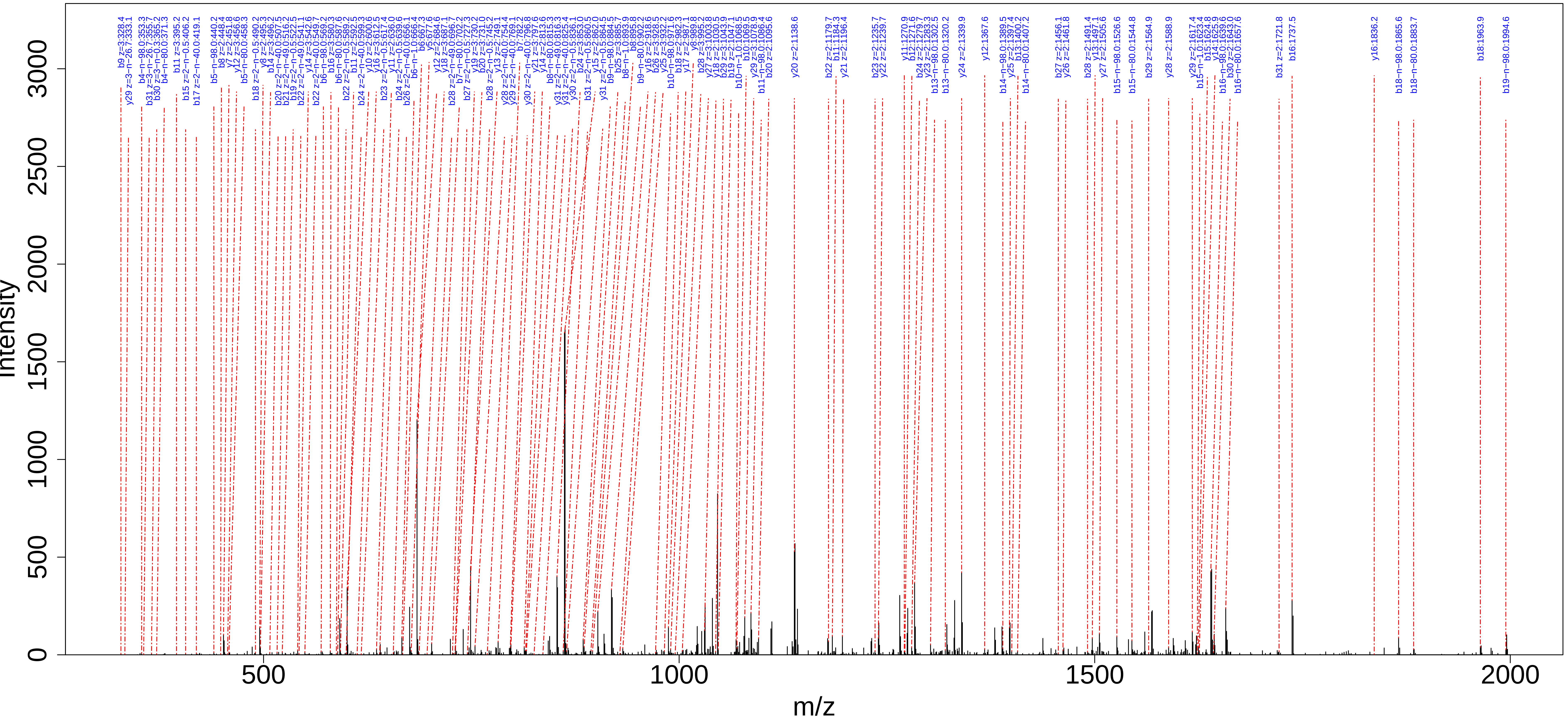

annotated raw MSMS spectra for assay:

Protein kinase: WNK1

Substrate: TBC1D1

sample name: TBCD1_WNK1_S3

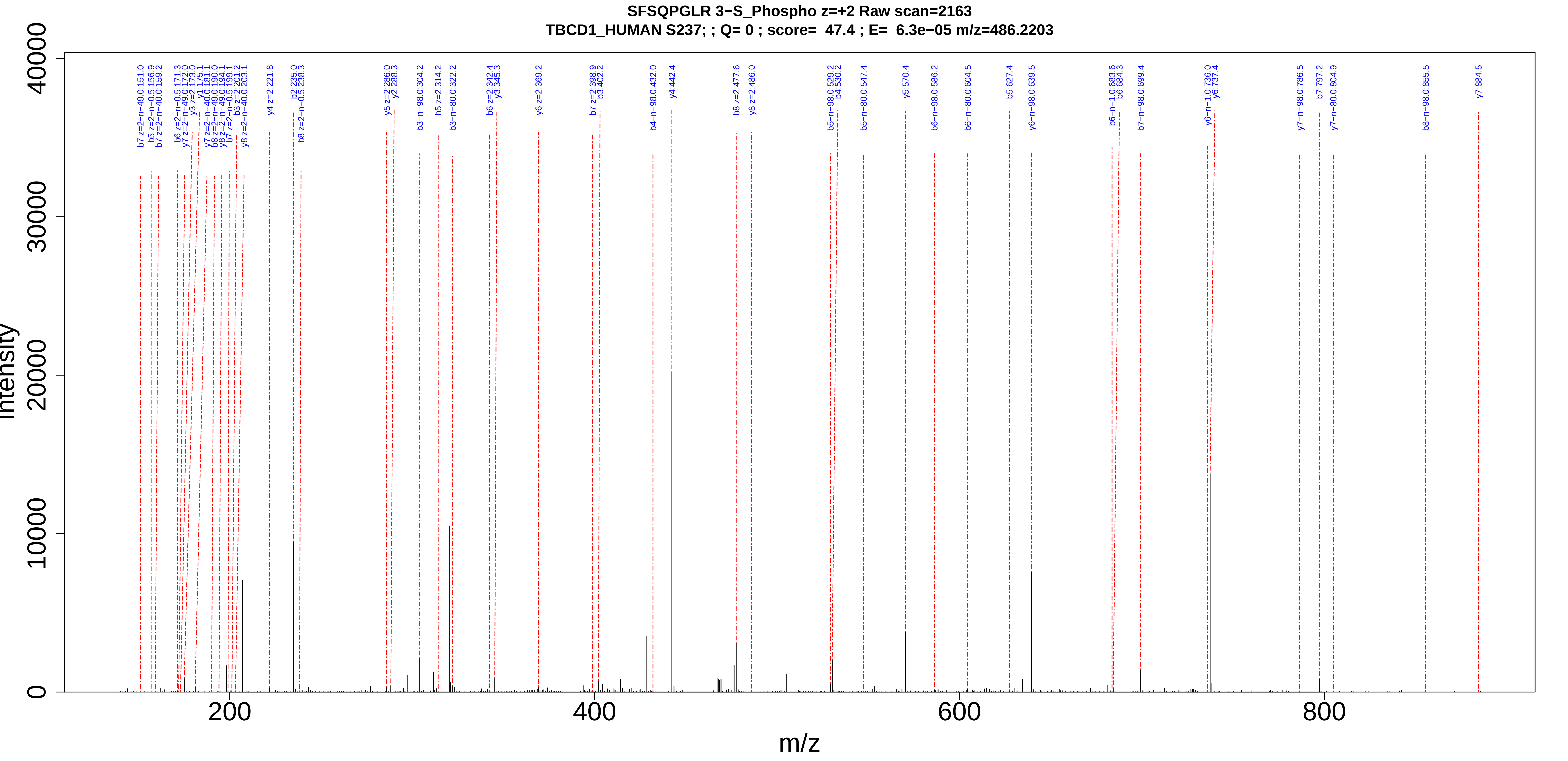
SFSQPGLR 3−S_Phospho z=+2 Raw scan=2163
TBCD1_HUMAN S237; ; Q= 0 ; score= 47.4 ; E= 6.3e−05 m/z=486.2203
Intensity
m/z

SFSQPGLR 3−S_Phospho z=+2 Raw scan=2170
TBCD1_HUMAN S235; ; Q= 0 ; score= 46.6 ; E= 0.00018 m/z=486.2196

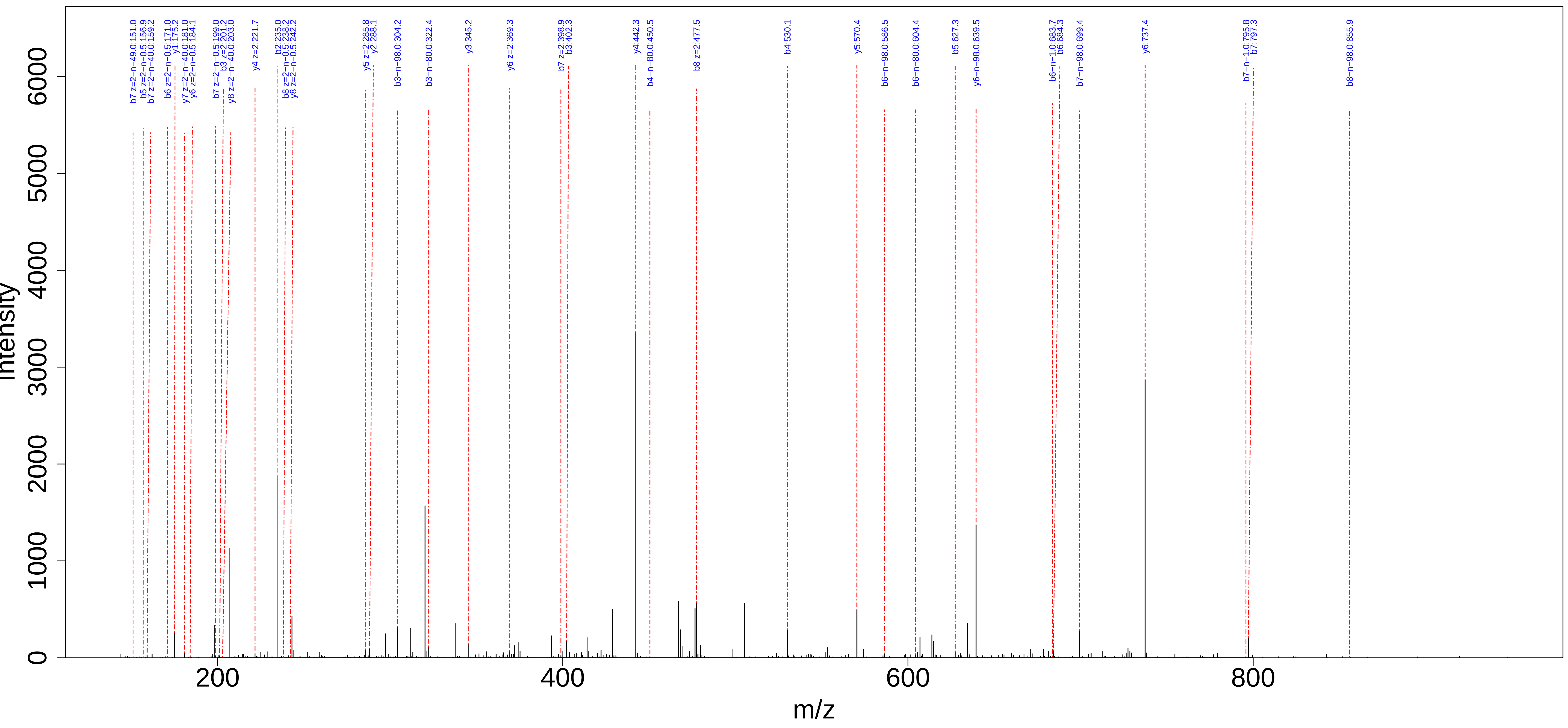

SFSQPGLR 3−S_Phospho z=+2 Raw scan=2128
TBCD1_HUMAN S237; ; Q= 0 ; score= 40.6 ; E= 0.00026 m/z=486.2209

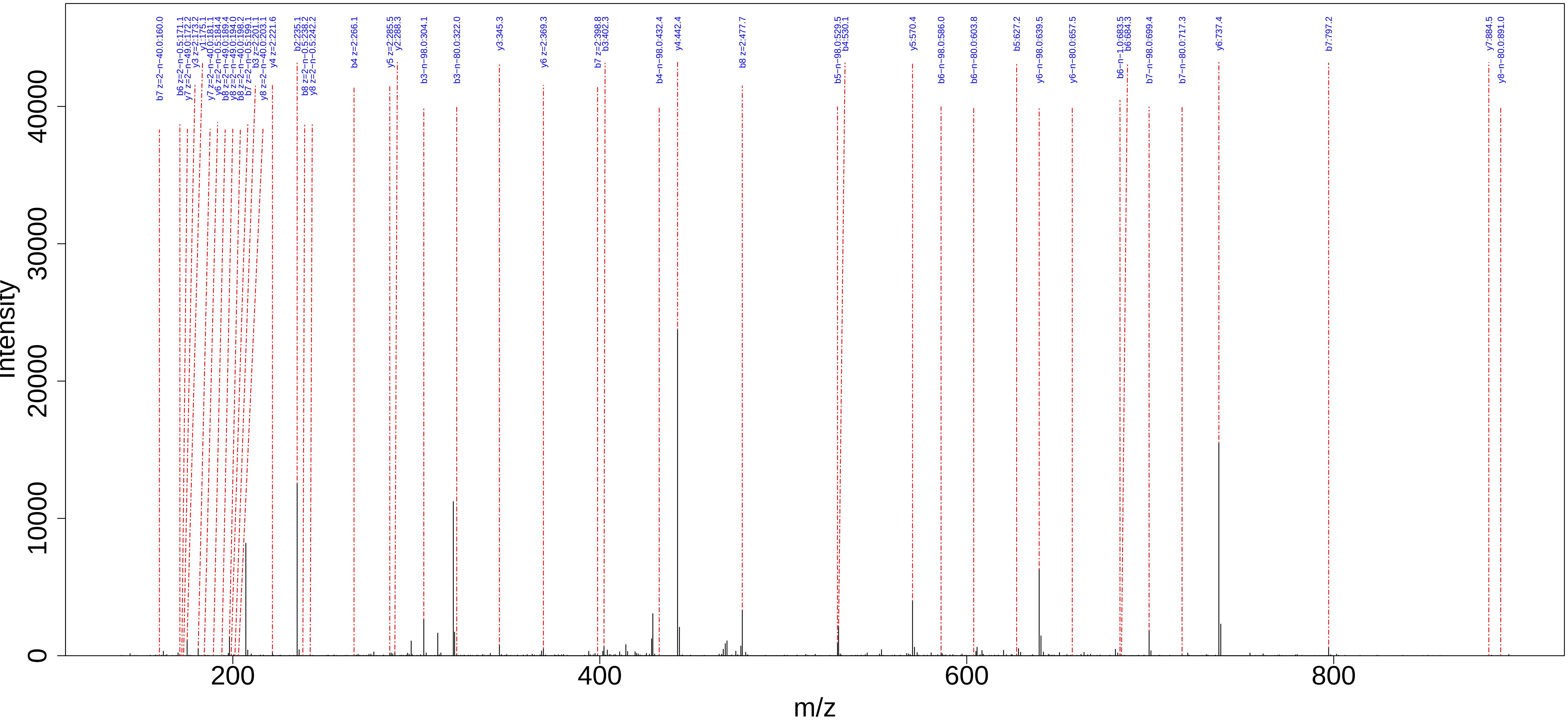

SFSQPGLR 3−S_Phospho z=+2 Raw scan=2147
TBCD1_HUMAN S237; ; Q= 0 ; score= 40.6 ; E= 0.00017 m/z=486.2207

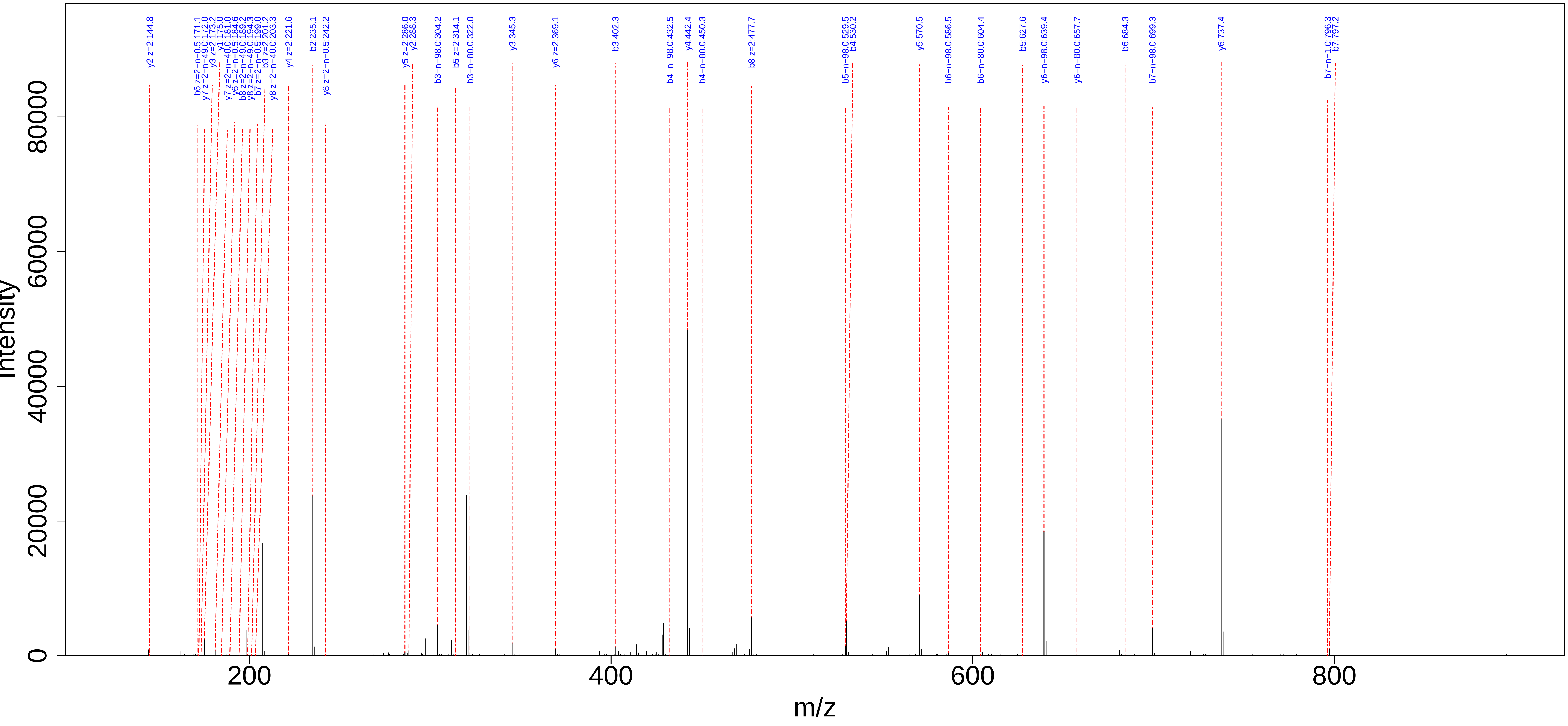

SFSQPGLR 3−S_Phospho z=+2 Raw scan=2134
TBCD1_HUMAN S237; ; Q= 0 ; score= 40.6 ; E= 7.3e−05 m/z=486.2202

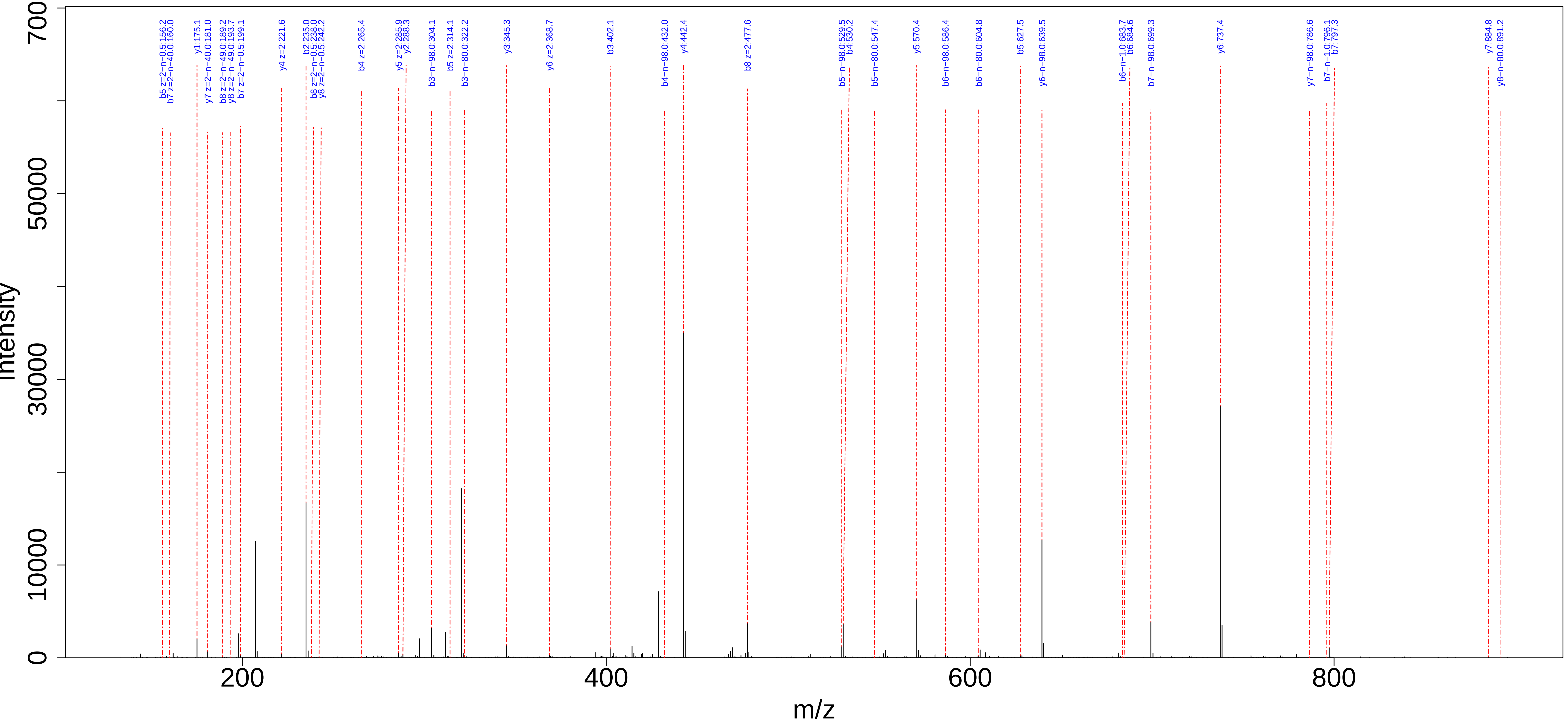

SFSQPGLR 3−S_Phospho z=+2 Raw scan=2141
TBCD1_HUMAN S235; ; Q= 0 ; score= 36.8 ; E= 4e−04 m/z=486.2206

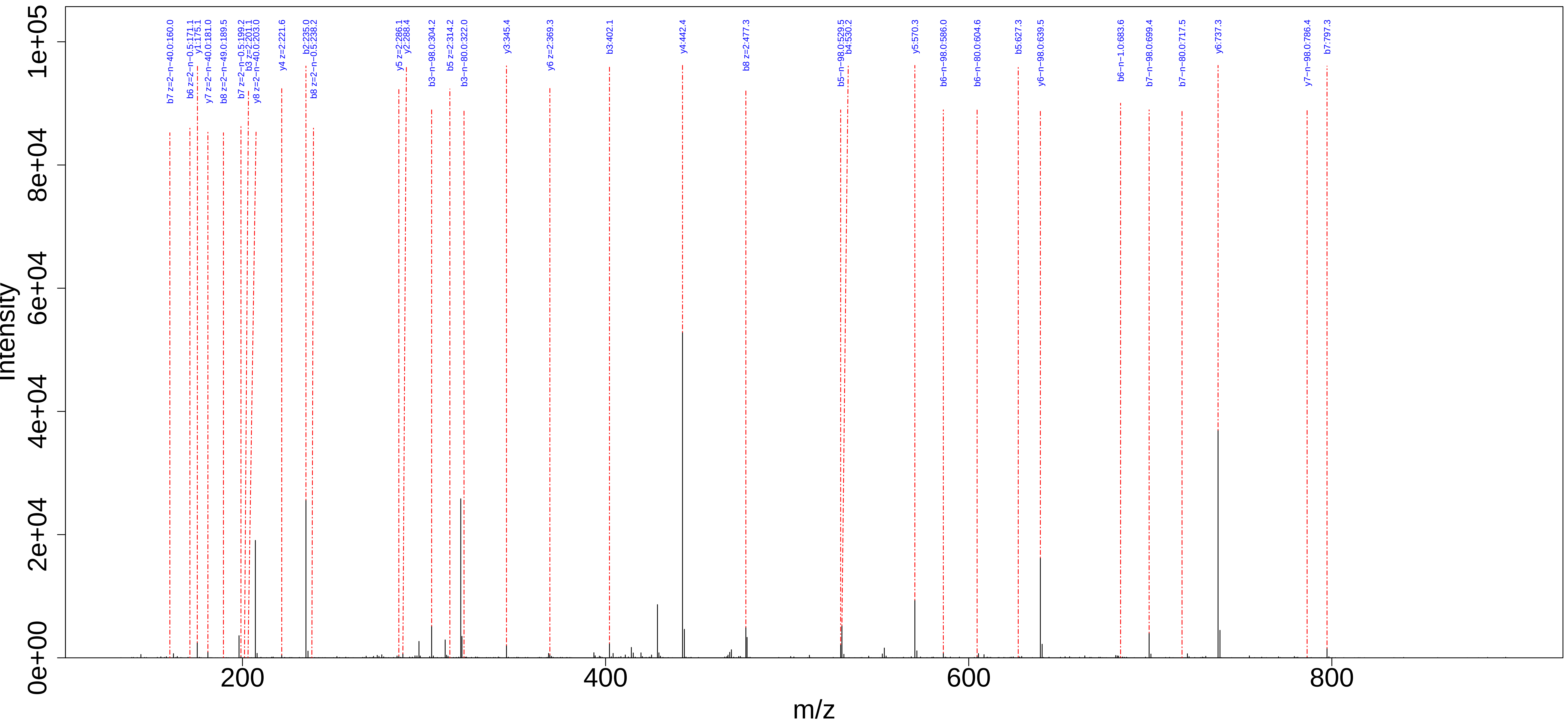

SFSQPGLR 3−S_Phospho z=+2 Raw scan=2154
TBCD1_HUMAN S237; ; Q= 0.0039544080018609 ; score= 33.1 ; E= 0.0013 m/z=486.2204

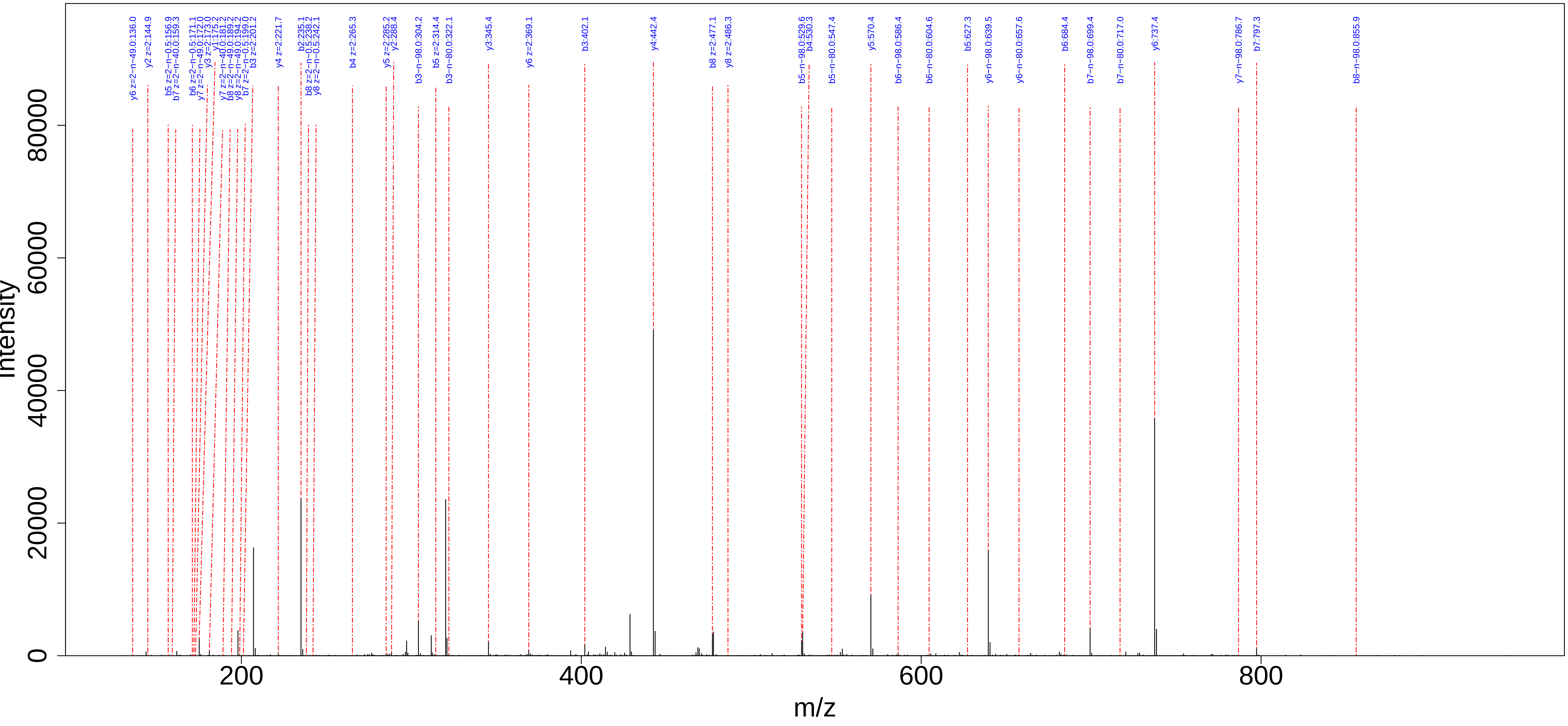

annotated raw MSMS spectra for assay:

Protein kinase: WNK1

Substrate: TBC1D4

sample name: TBCD4_WNK1_2

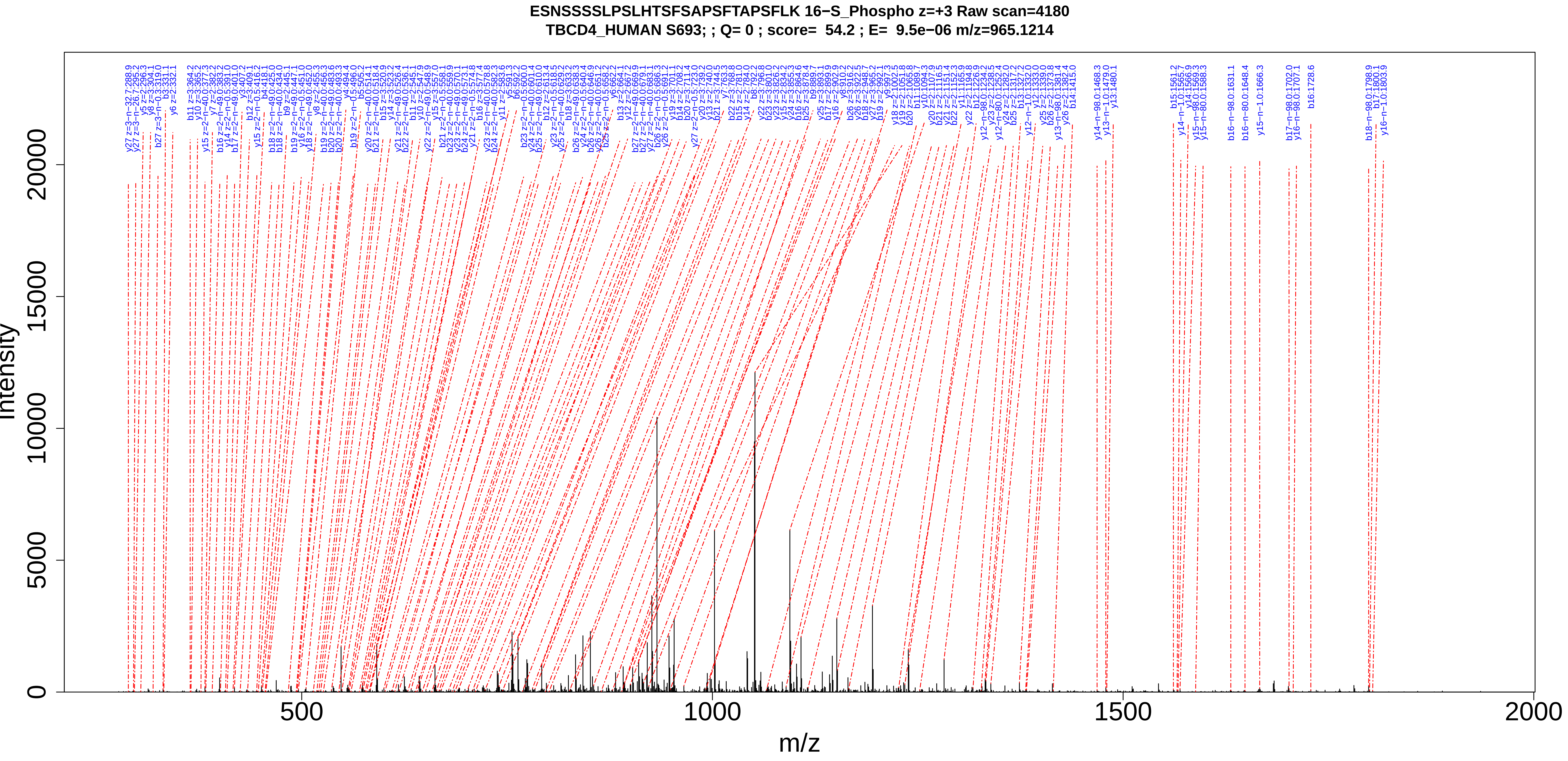

ESNSSSSLPSLHTSFSAPSFTAPSFLK 16−S_Phospho z=+3 Raw scan=4180
TBCD4_HUMAN S693; ; Q= 0 ; score= 54.2 ; E= 9.5e−06 m/z=965.1214
Intensity
m/z
0
5000
10000
15000
20000
500
1000
1500
2000

CVPAPGAGASGGTSPSATQPNPAVFIFEHK 14−S_Phospho z=+3 Raw scan=3841
TBCD4_HUMAN T105; ; Q= 0 ; score= 45.3 ; E= 3.6e−06 m/z=1011.4759

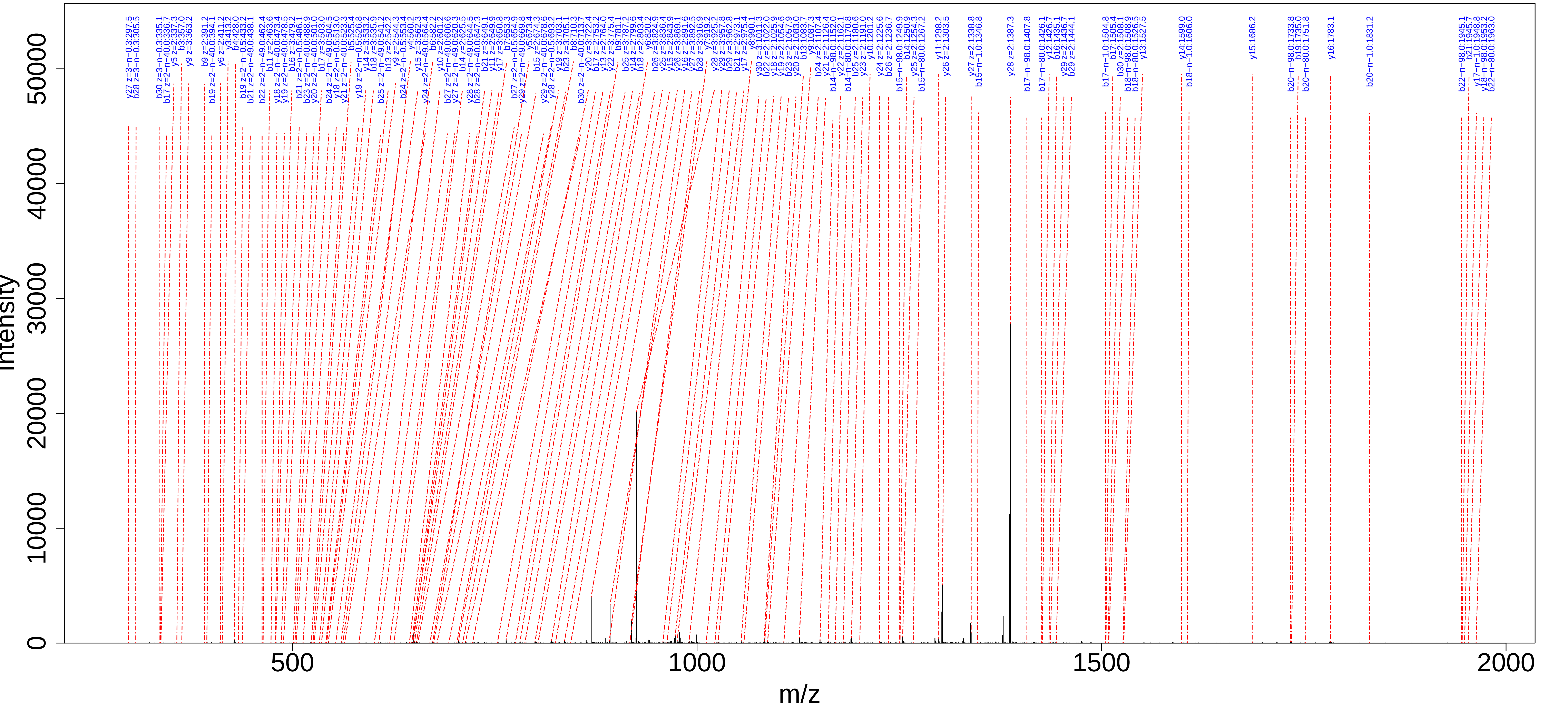

SFYQNSGRLSPQYENEIRQDTASESSDGEGR 10−S_Phospho z=+3 Raw scan=2796
TBCD4_HUMAN S721; ; Q= 0 ; score= 39.8 ; E= 1.4e−06 m/z=1196.5106

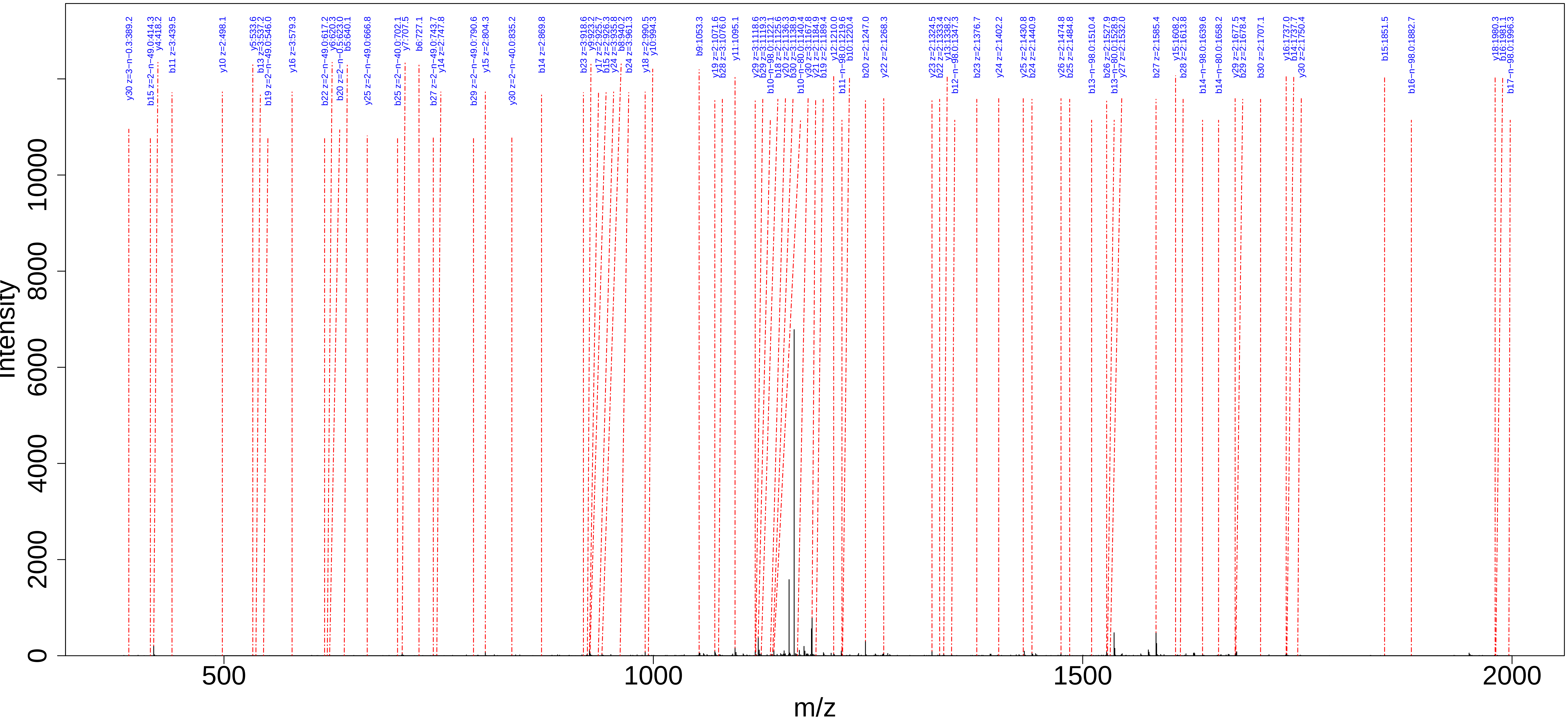

SFYQNSGRLSPQYENEIRQDTASESSDGEGR 10−S_Phospho z=+3 Raw scan=2801
TBCD4_HUMAN S721; ; Q= 0.000914913083257091 ; score= 38.7 ; E= 4.4e−05 m/z=1196.5096

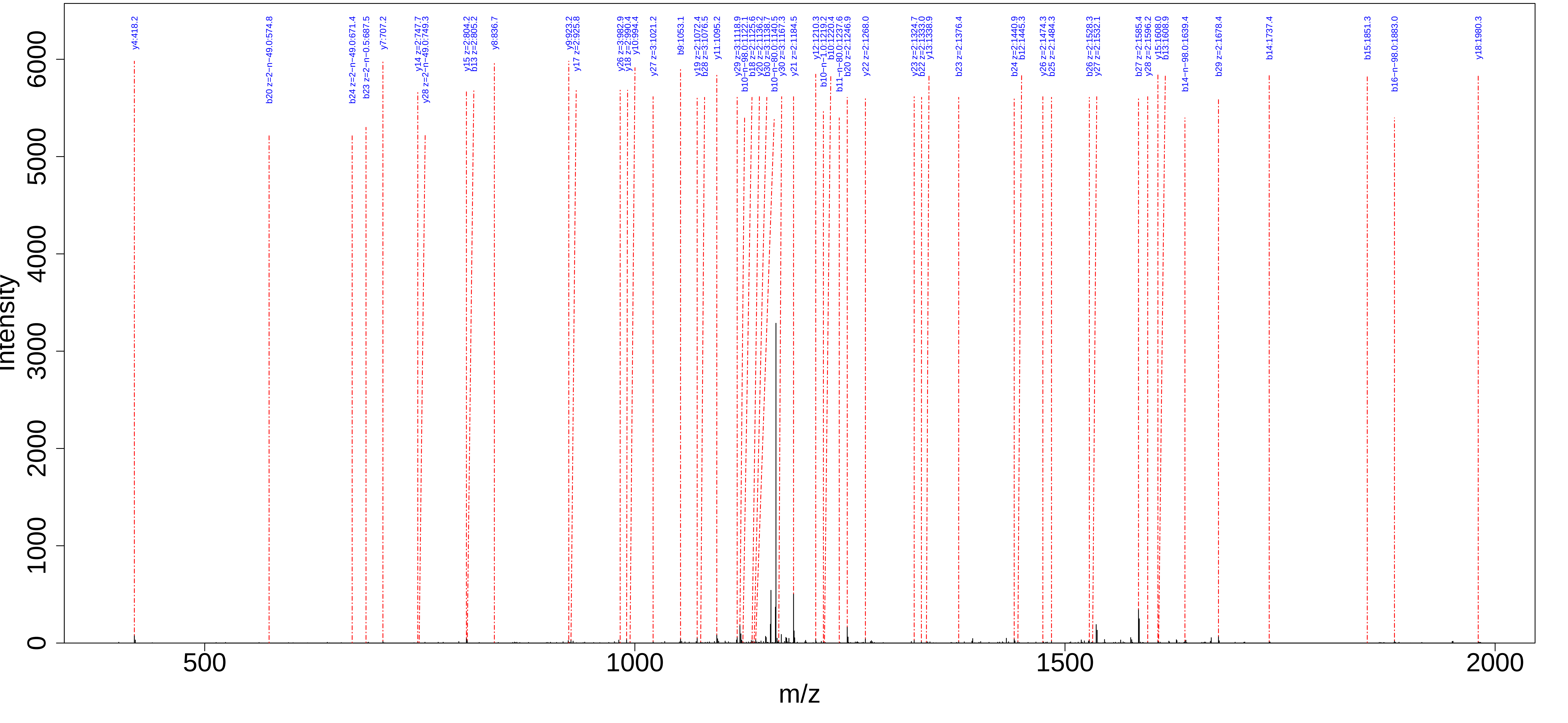

SFYQNSGRLSPQYENEIR 10−S_Phospho z=+3 Raw scan=2842
TBCD4_HUMAN S721; ; Q= 0.00177777777777778 ; score= 36.8 ; E= 0.027 m/z=756.6749

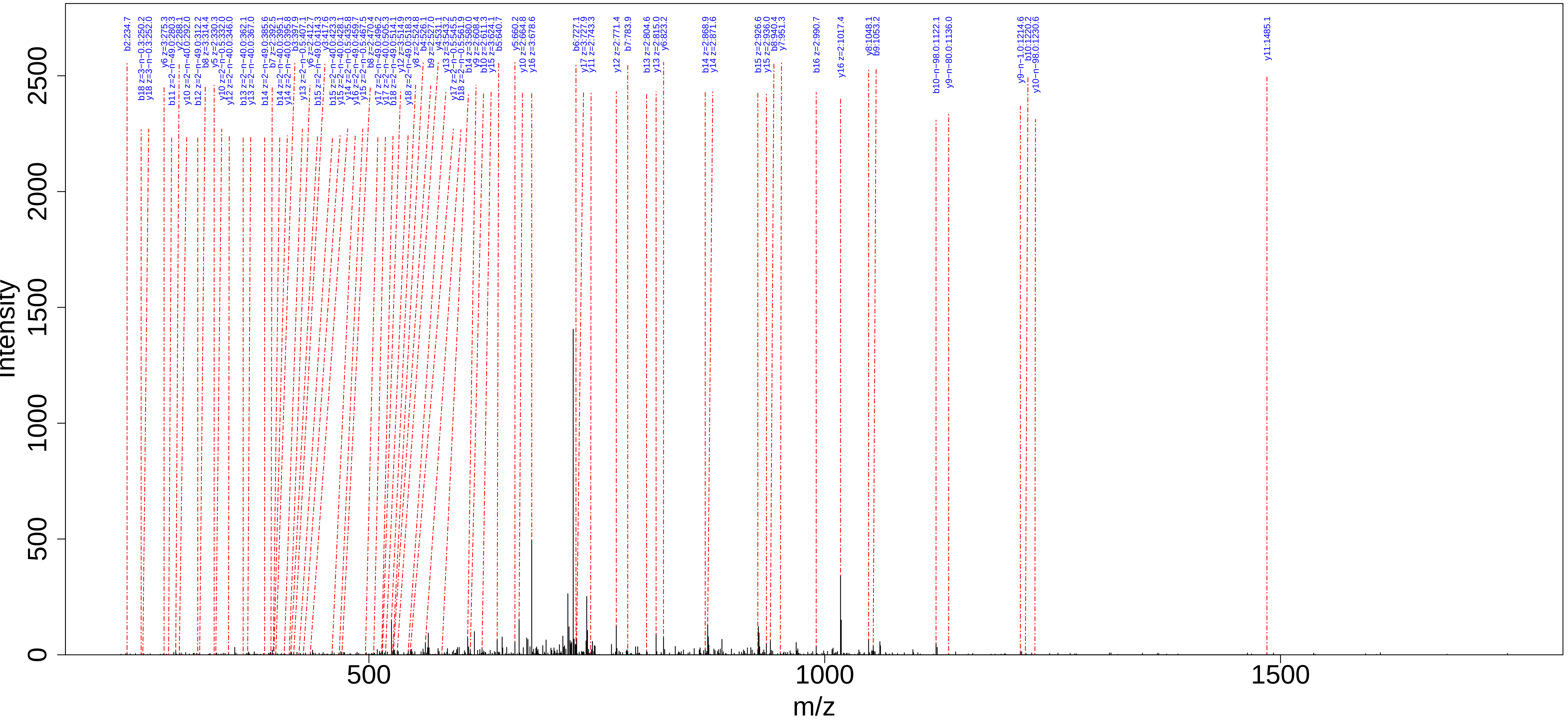

SGSGGGSAKEPQEERSQQQDDIEELETK 1−S_Phospho z=+3 Raw scan=2504
WNK1_HUMAN S185; ; Q= 0.00174978127734033 ; score= 36.3 ; E= 0.0031 m/z=1034.1213

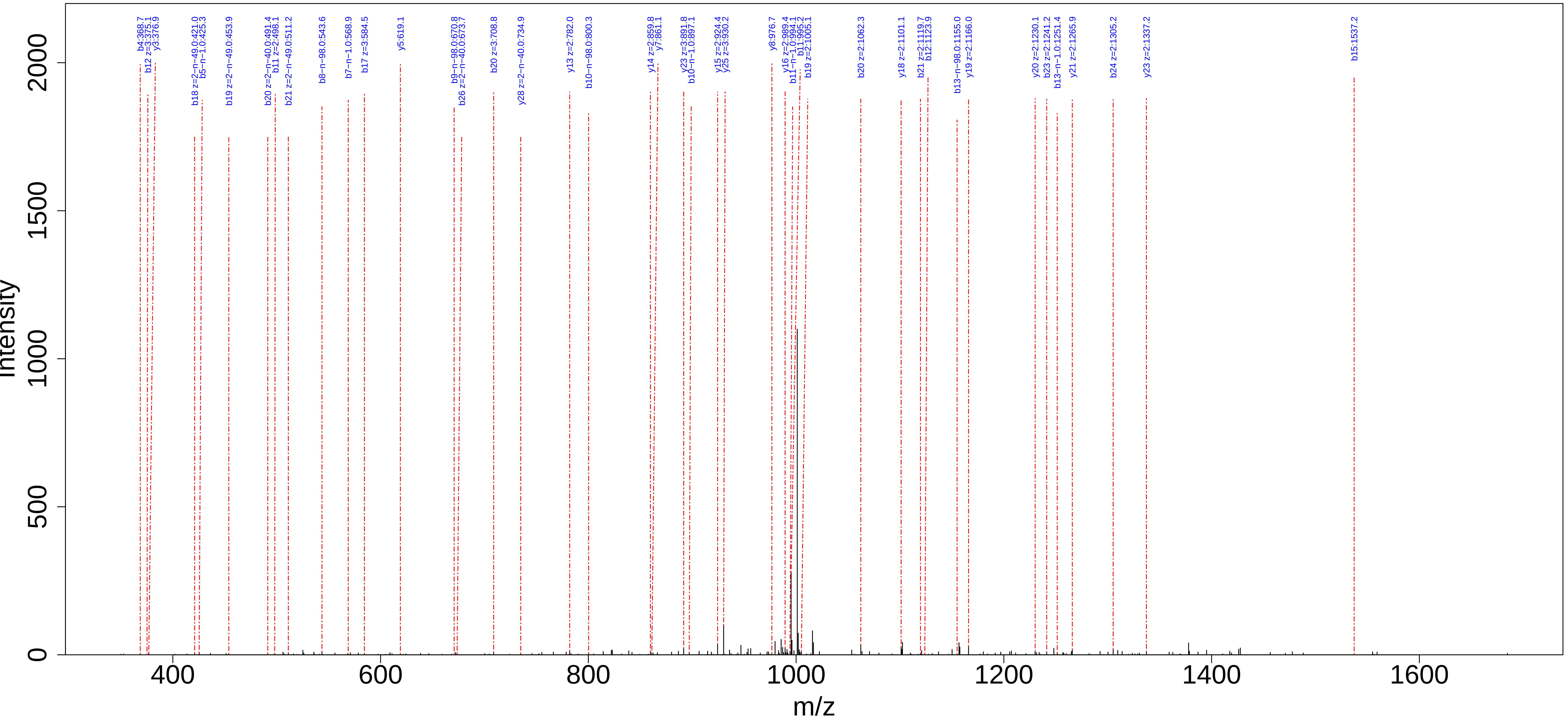

SGSGGGSAKEPQEERSQQQDDIEELETK 3−S_Phospho z=+3 Raw scan=2504
WNK1_HUMAN S185; ; Q= 0.00174825174825175 ; score= 36.3 ; E= 0.0031 m/z=1034.1213

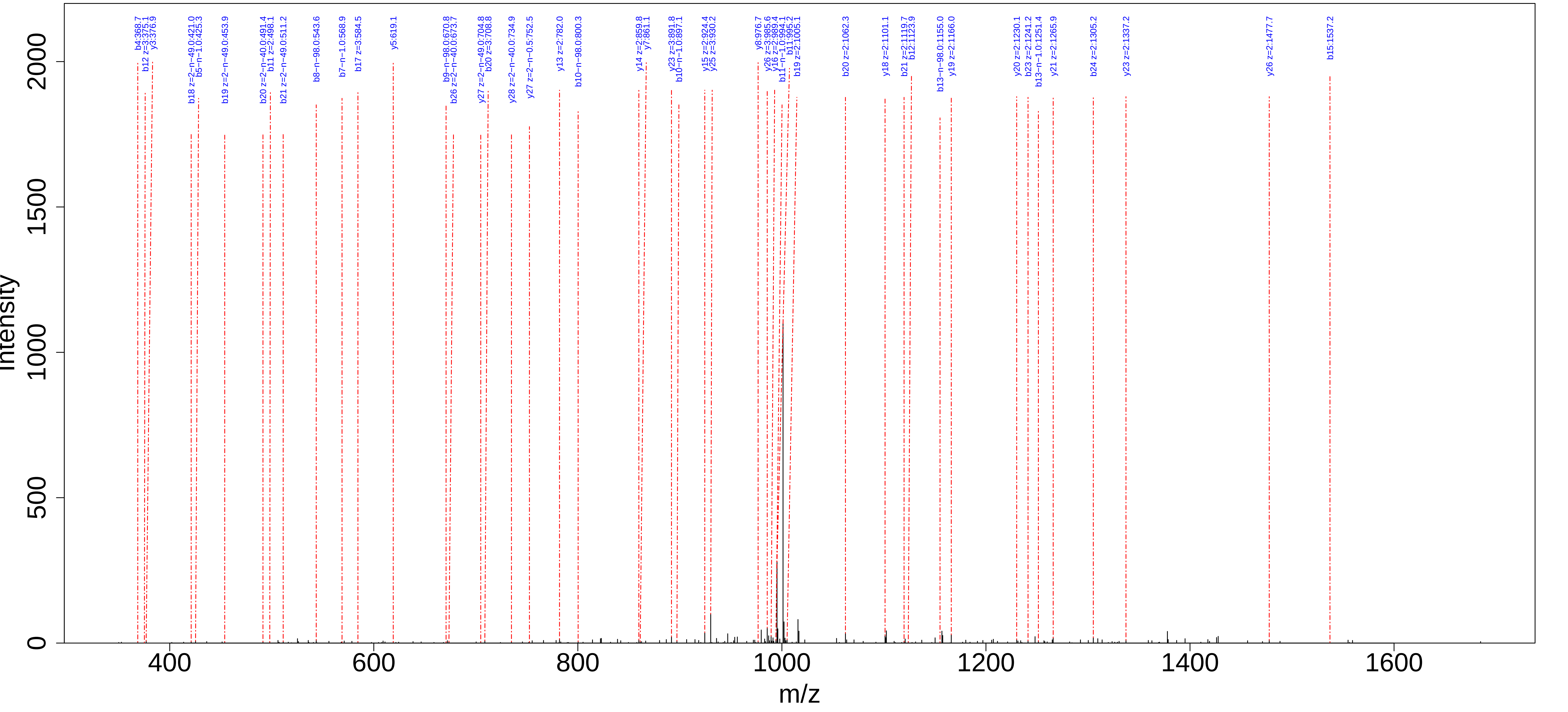

SFYQNSGRLSPQYENEIRQDTASESSDGEGR 10−S_Phospho z=+3 Raw scan=2791
TBCD4_HUMAN S721; ; Q= 0.00168208578637511 ; score= 35 ; E= 0.00011 m/z=1196.5166

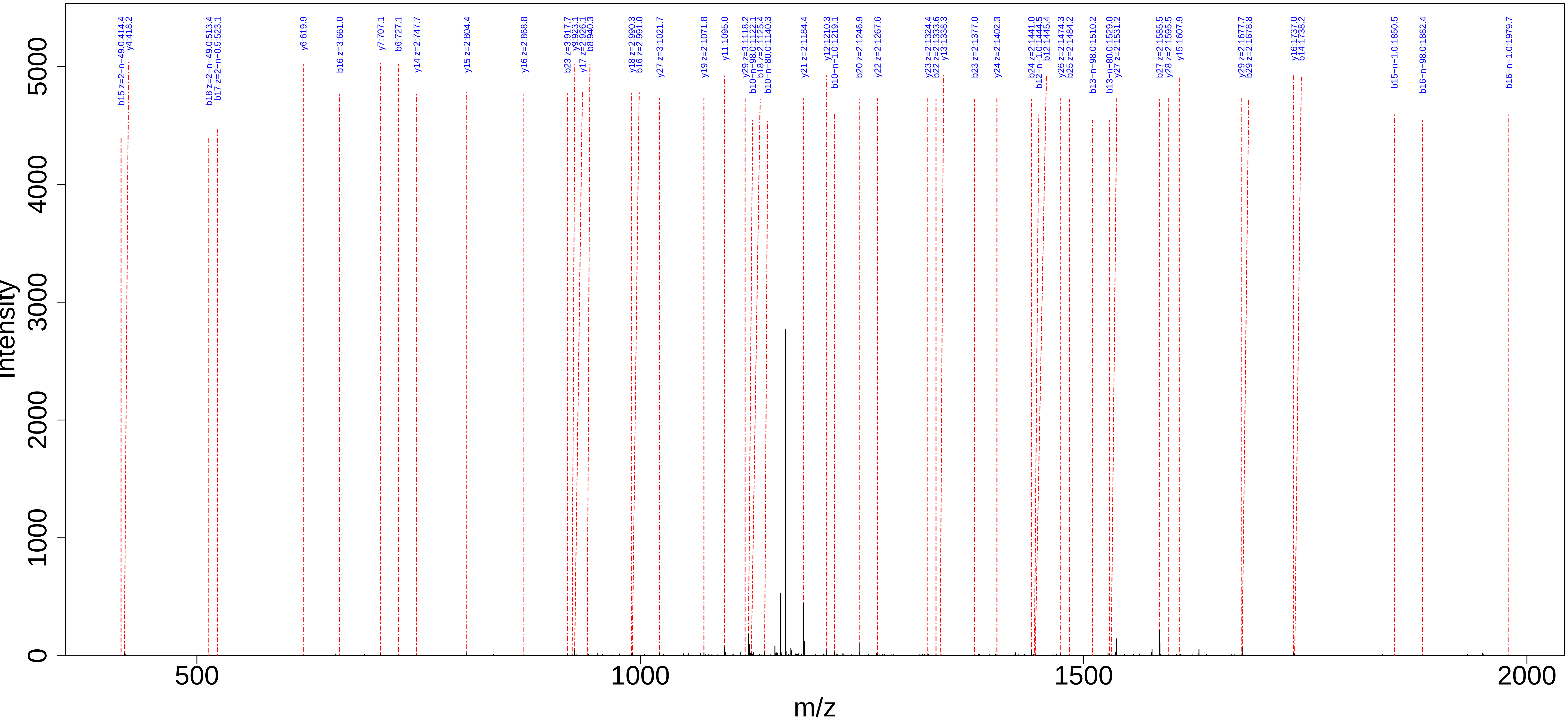

LSPPAPAPKNGSSSDSSVGEKLGAAA 13−S_Phospho/14−S_Phospho/1−n_proAcetyl z=+3 Raw scan=5031
WNK1_HUMAN S19;S33; ; Q= 0.00557324840764331 ; score= 28.6 ; E= 0.0063 m/z=866.3956

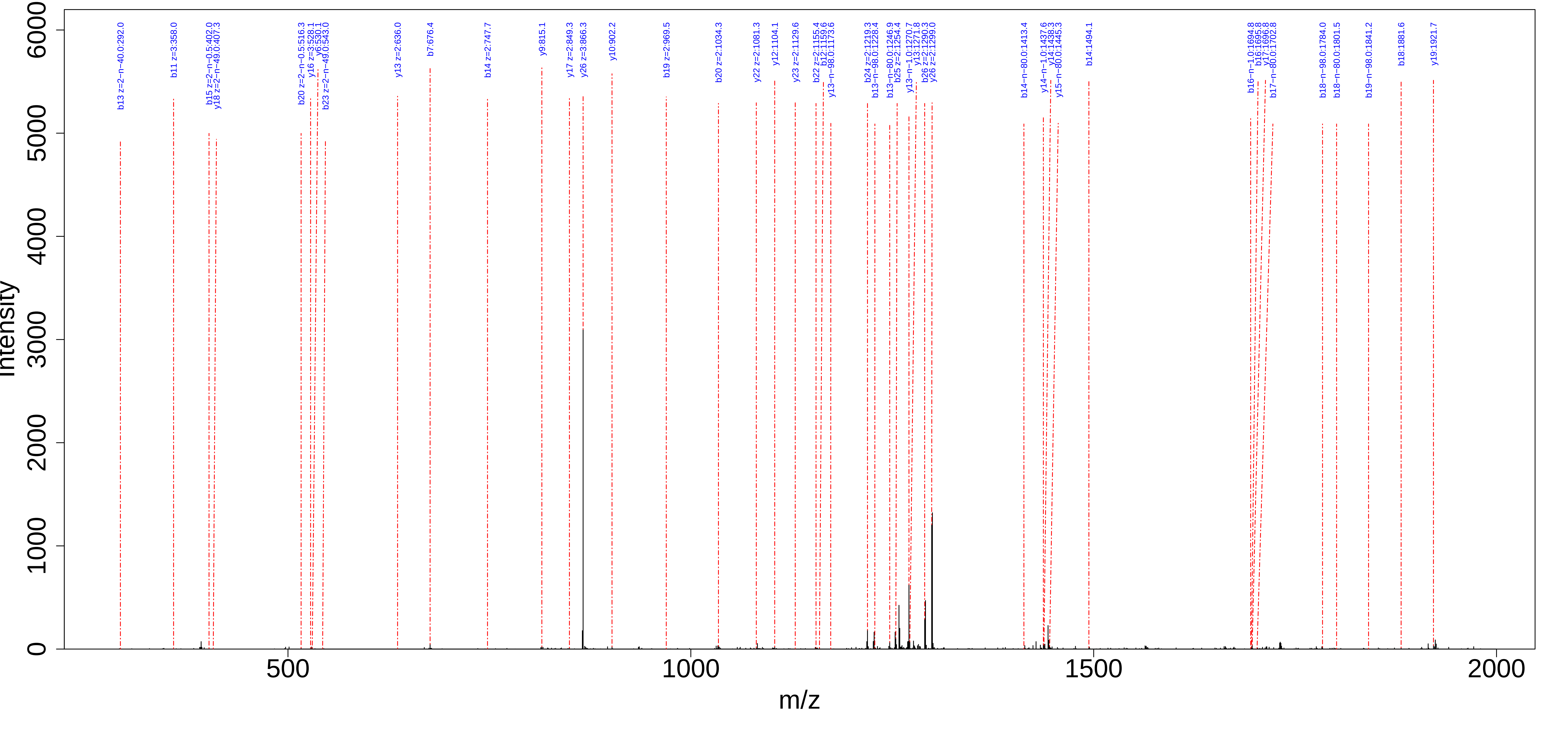

annotated raw MSMS spectra for assay:

Protein kinase: WNK1

Substrate: TBC1D4

sample name: TBCD4_WNK1_phos_2

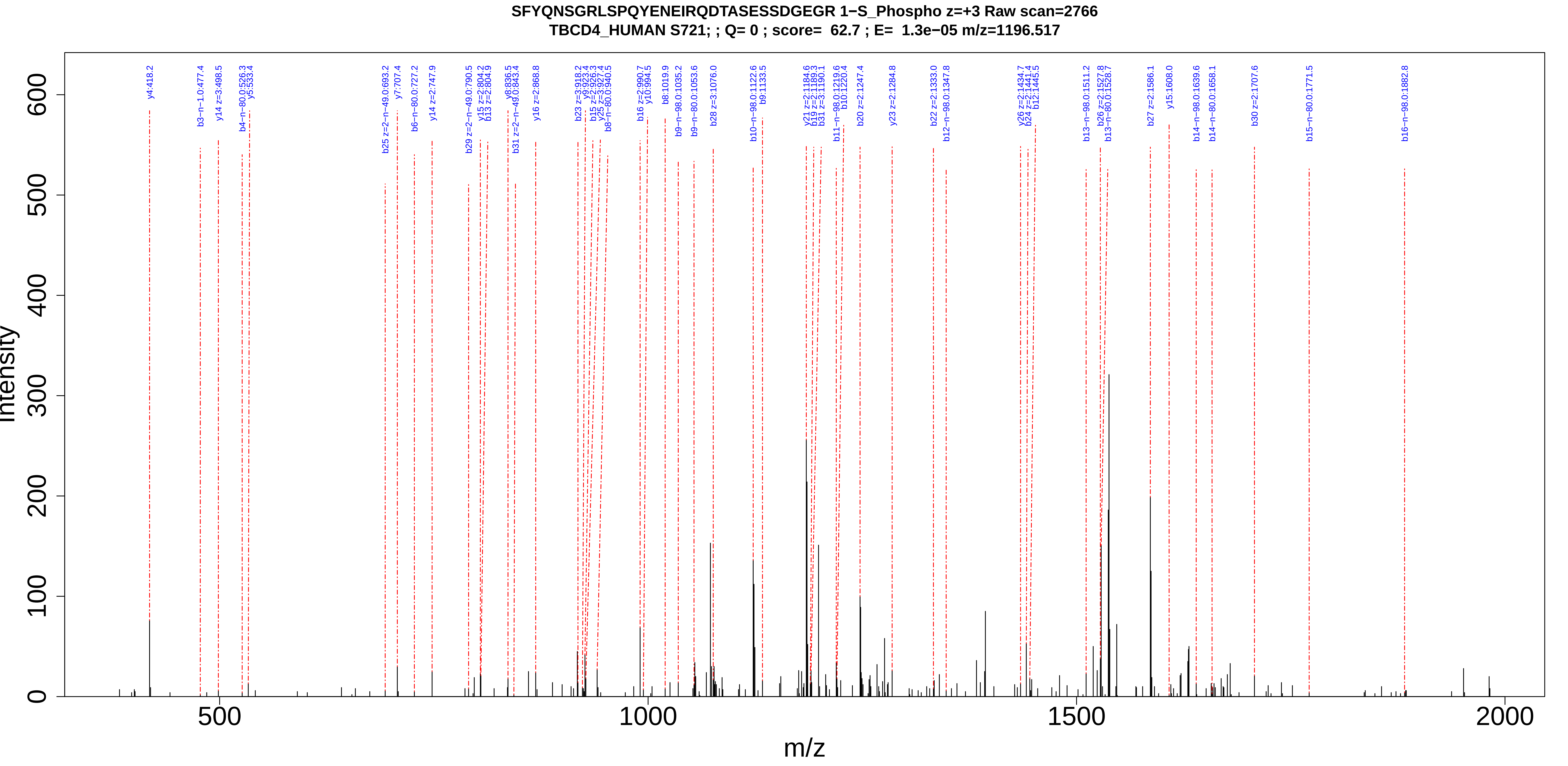
SFYQNSGRLSPQYENEIRQDTASESSDGEGR 1−S_Phospho z=+3 Raw scan=2766
TBCD4_HUMAN S721; ; Q= 0 ; score= 62.7 ; E= 1.3e−05 m/z=1196.517
Intensity
m/z

SFYQNSGRLSPQYENEIRQDTASESSDGEGR 3−Y_Phospho z=+3 Raw scan=2766
TBCD4_HUMAN S721; ; Q= 0 ; score= 62.7 ; E= 1.3e−05 m/z=1196.517

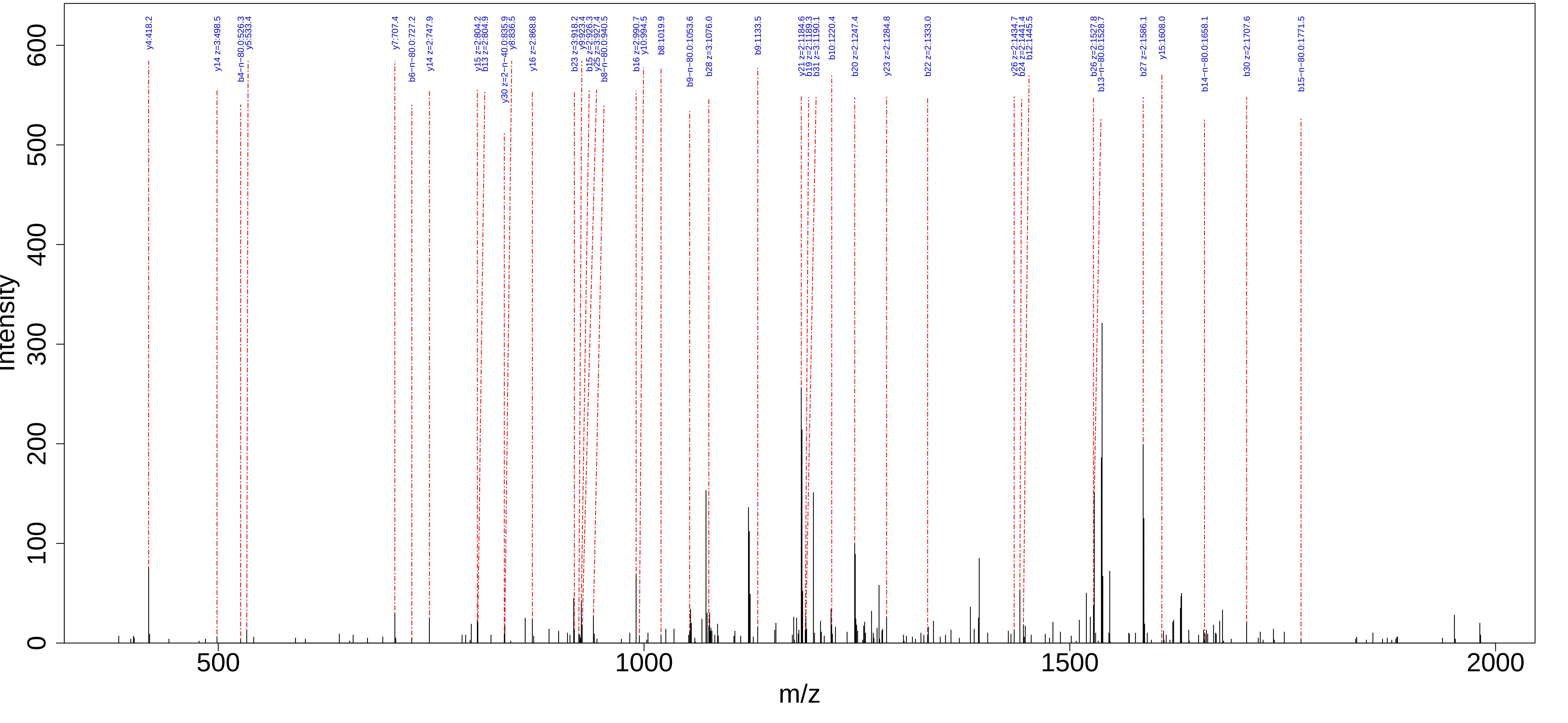

SFYQNSGRLSPQYENEIRQDTASESSDGEGR 6−S_Phospho z=+3 Raw scan=2766
TBCD4_HUMAN S721; ; Q= 0 ; score= 62.7 ; E= 1.3e−05 m/z=1196.517

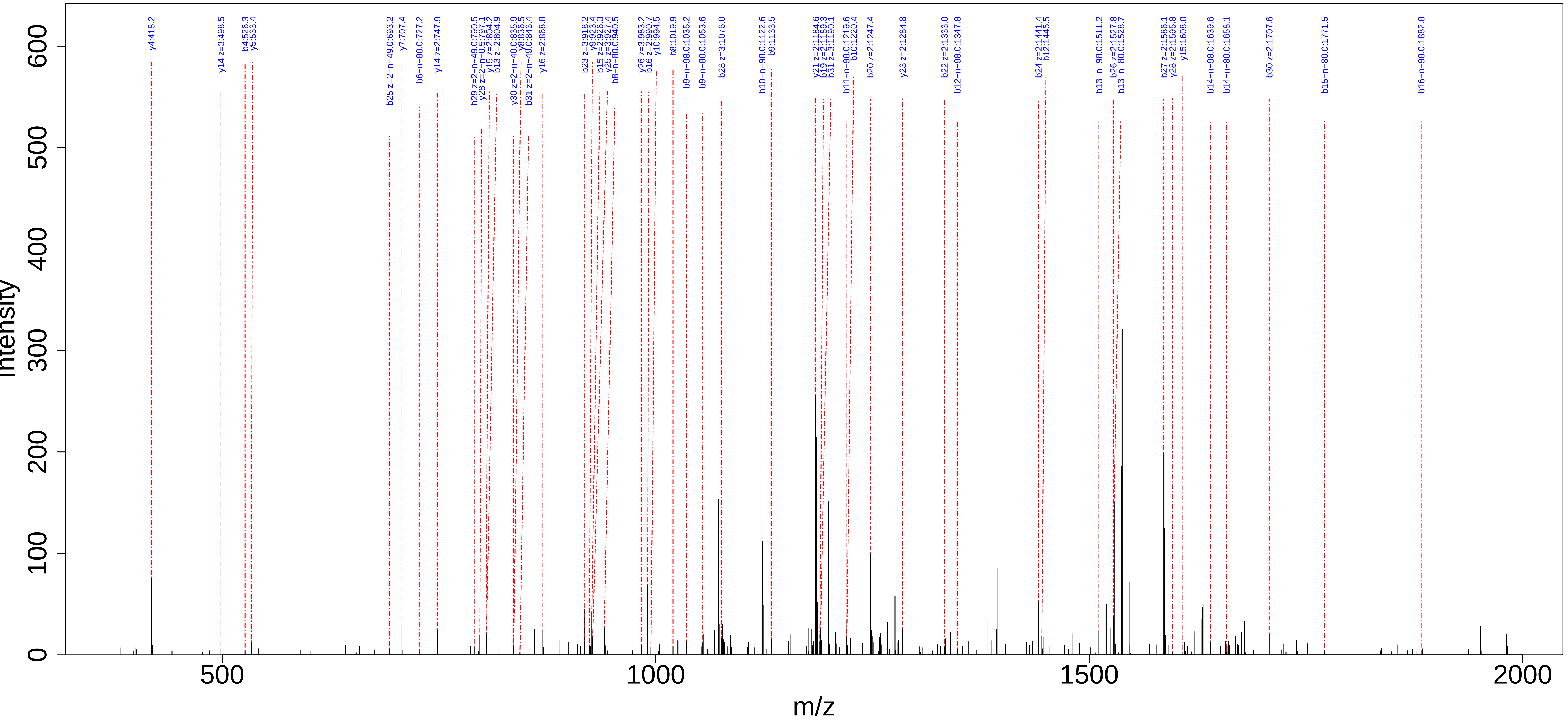

CVPAPGAGASGGTSPSATQPNPAVFIFEHK 13−T_Phospho z=+3 Raw scan=4305
TBCD4_HUMAN S106; ; Q= 0 ; score= 54.4 ; E= 1.5e−06 m/z=1005.7972

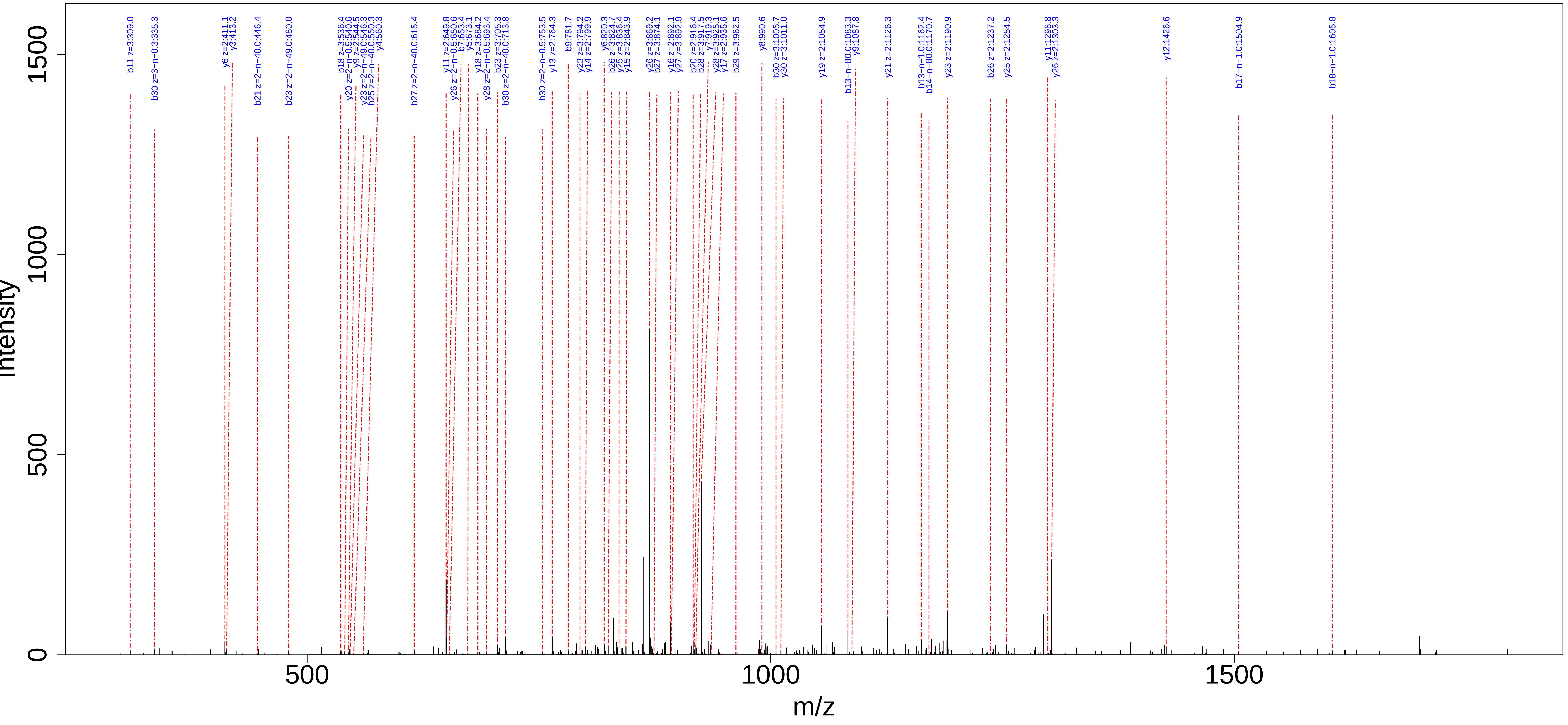

SFYQNSGRLSPQYENEIRQDTASESSDGEGR 10−S_Phospho z=+3 Raw scan=2781
TBCD4_HUMAN S721; ; Q= 0 ; score= 54.3 ; E= 0.00031 m/z=1196.5221

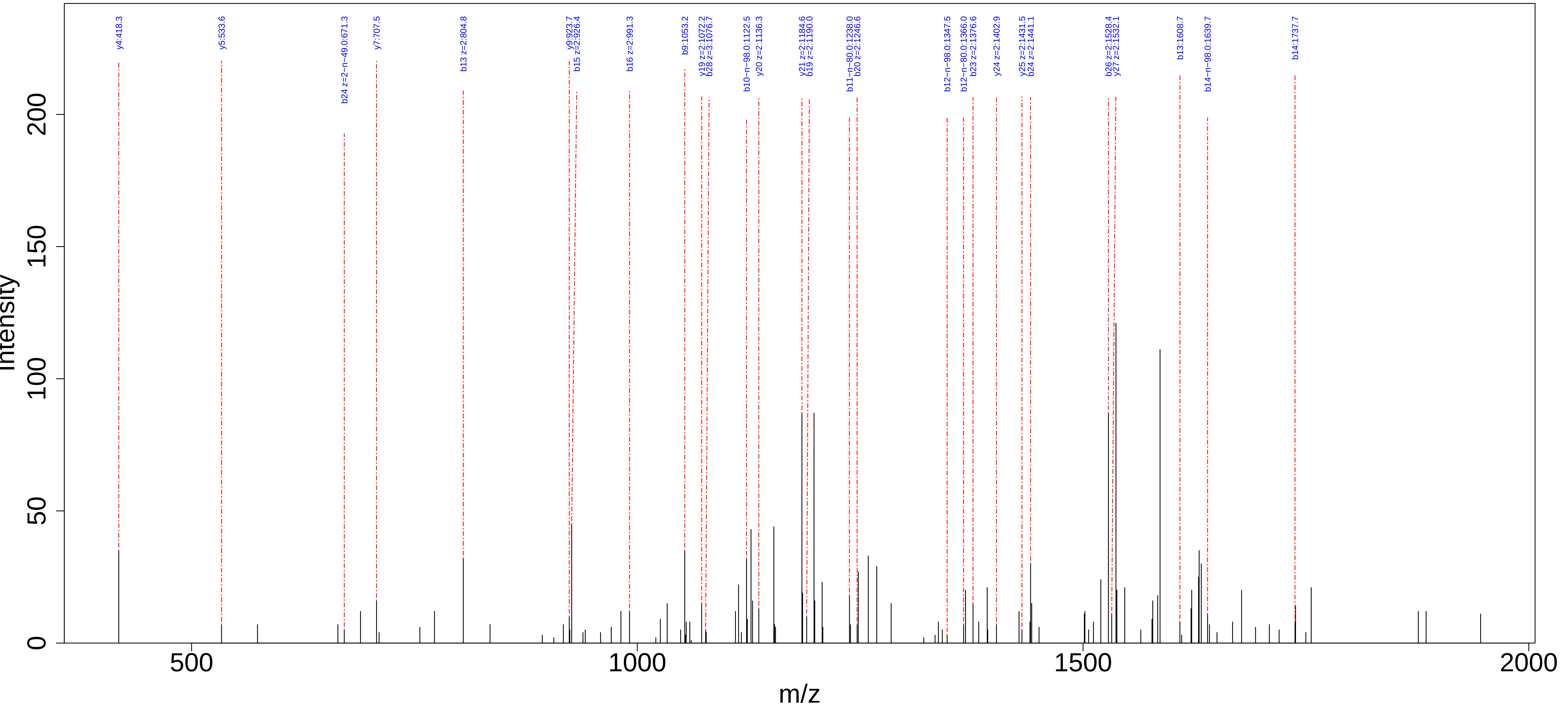

SFYQNSGRLSPQYENEIRQDTASESSDGEGR 10−S_Phospho z=+3 Raw scan=2786
TBCD4_HUMAN S721; ; Q= 0 ; score= 52.8 ; E= 0.00019 m/z=1196.517

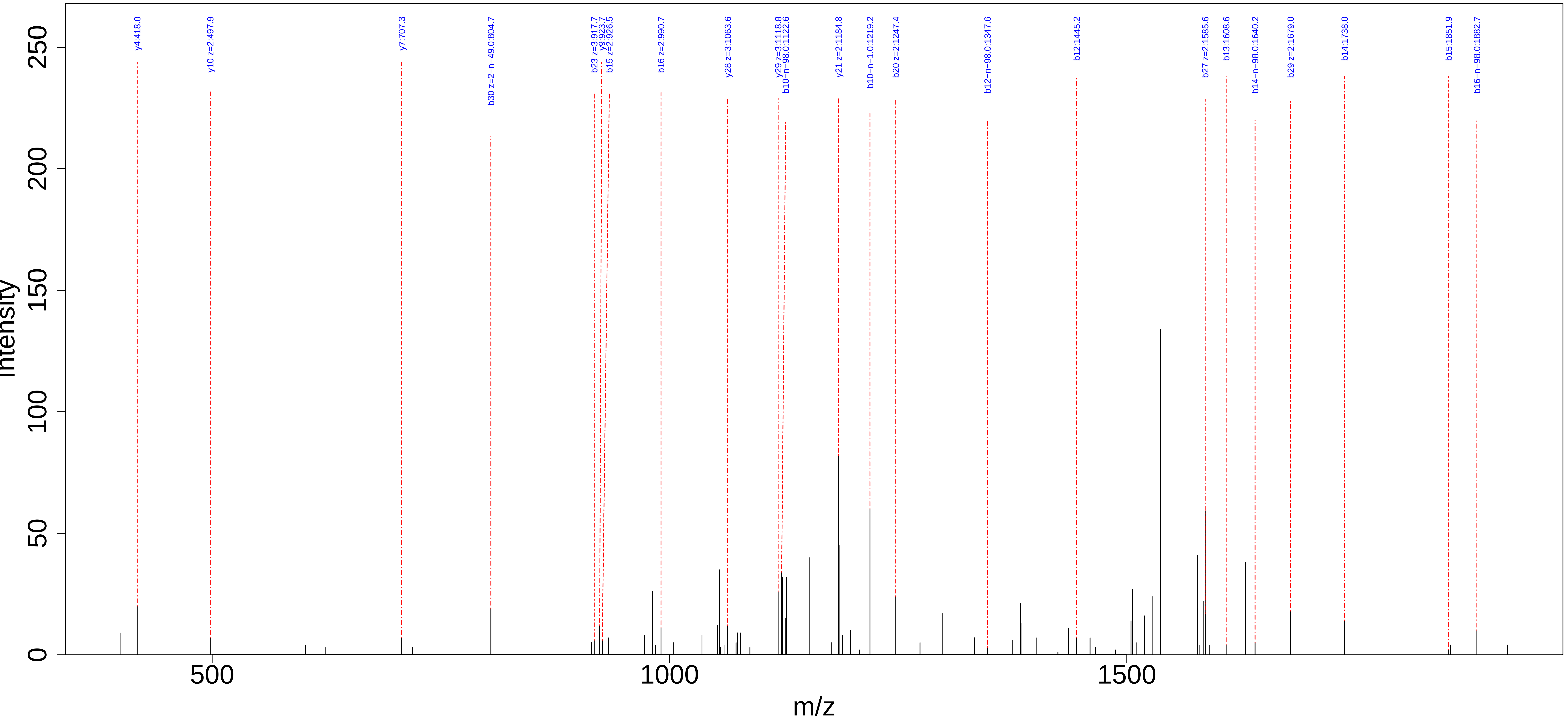

ESNSSSSLPSLHTSFSAPSFTAPSFLK 16−S_Phospho z=+3 Raw scan=4193
TBCD4_HUMAN S692; ; Q= 0 ; score= 52.5 ; E= 5.2e−05 m/z=965.1166

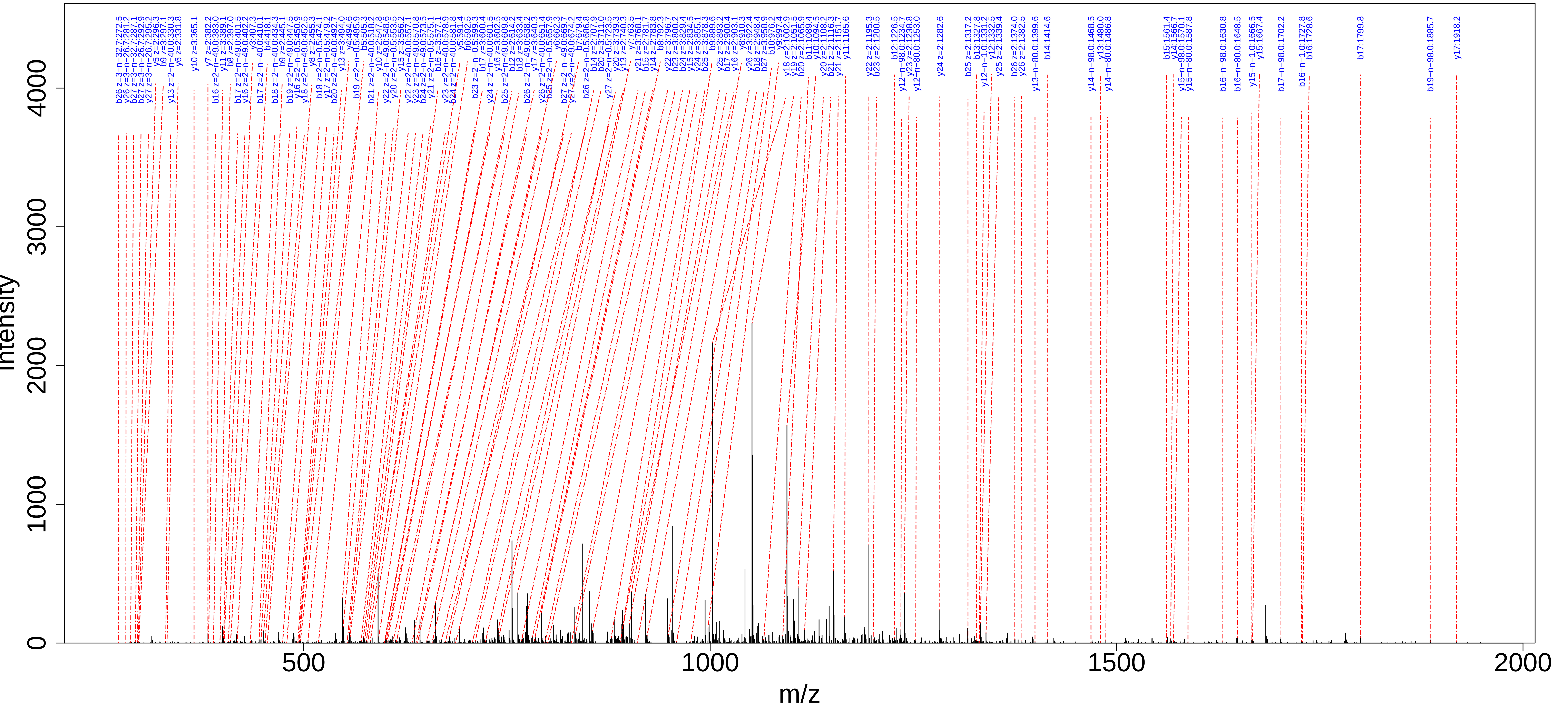

CVPAPGAGASGGTSPSATQPNPAVFIFEHK 14−S_Phospho z=+3 Raw scan=4296
TBCD4_HUMAN S106; ; Q= 0 ; score= 52.4 ; E= 1.5e−05 m/z=1005.796

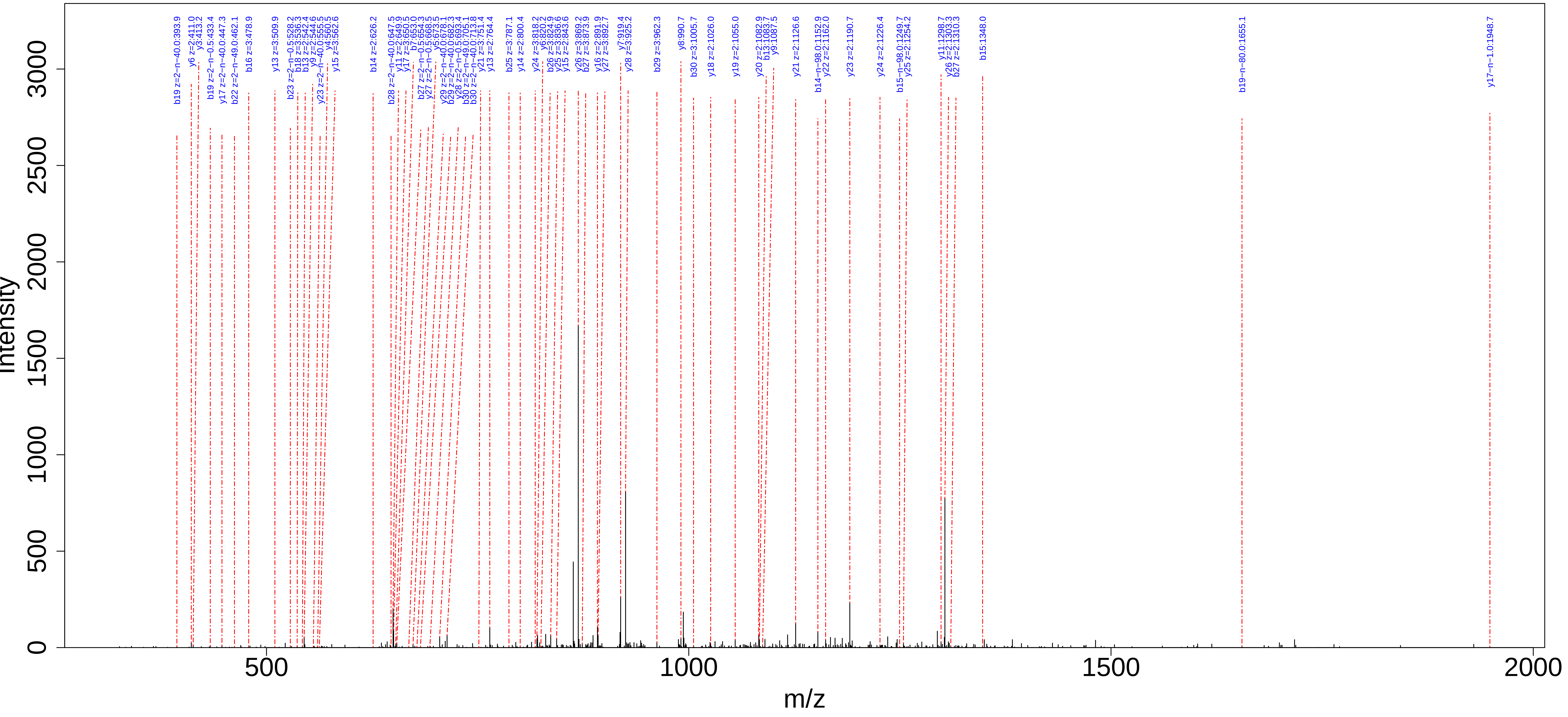

CVPAPGAGASGGTSPSATQPNPAVFIFEHK 14−S_Phospho z=+3 Raw scan=4289
TBCD4_HUMAN S108; ; Q= 0 ; score= 50.6 ; E= 3.8e−05 m/z=1005.7966

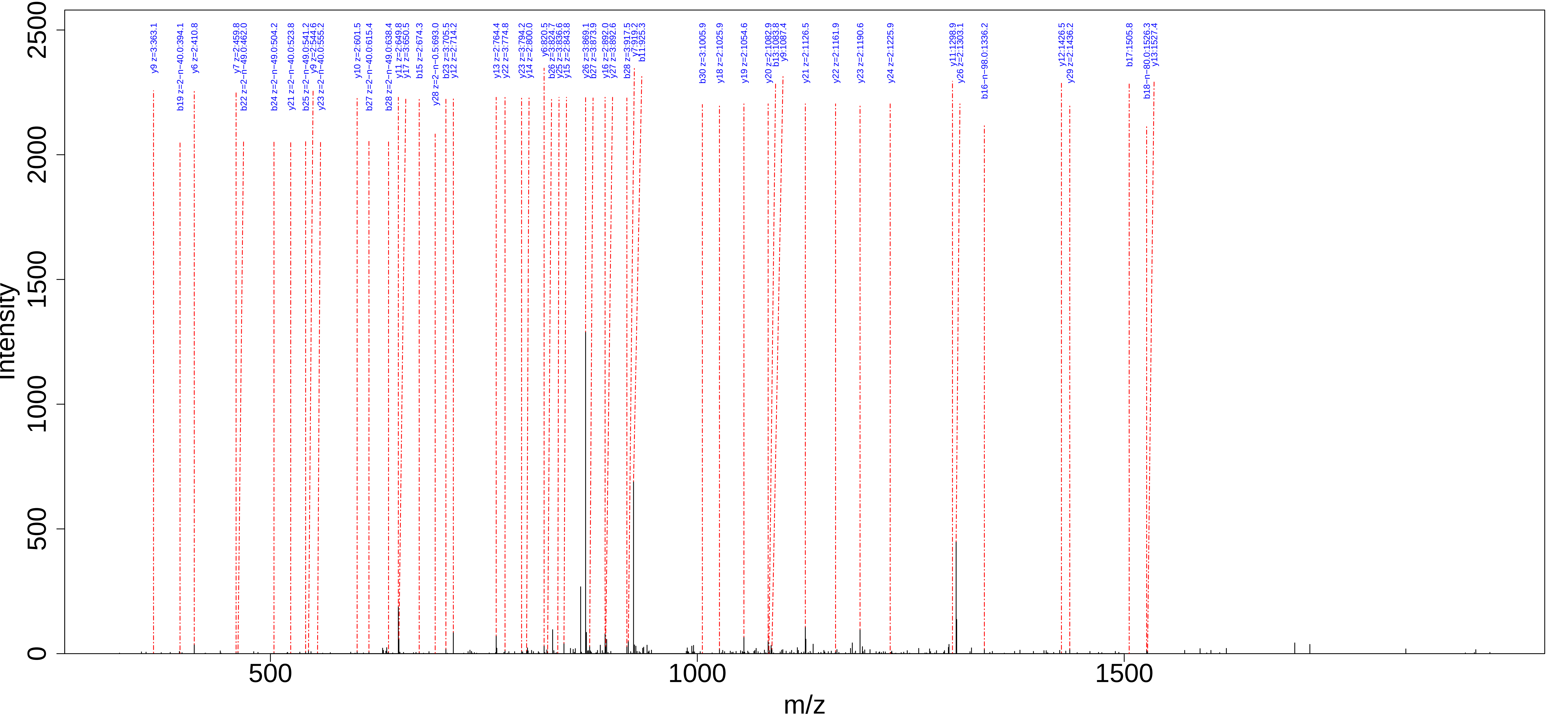

ESNSSSSLPSLHTSFSAPSFTAPSFLK 16−S_Phospho z=+3 Raw scan=4187
TBCD4_HUMAN S692; ; Q= 0 ; score= 49.2 ; E= 0.00031 m/z=965.1167

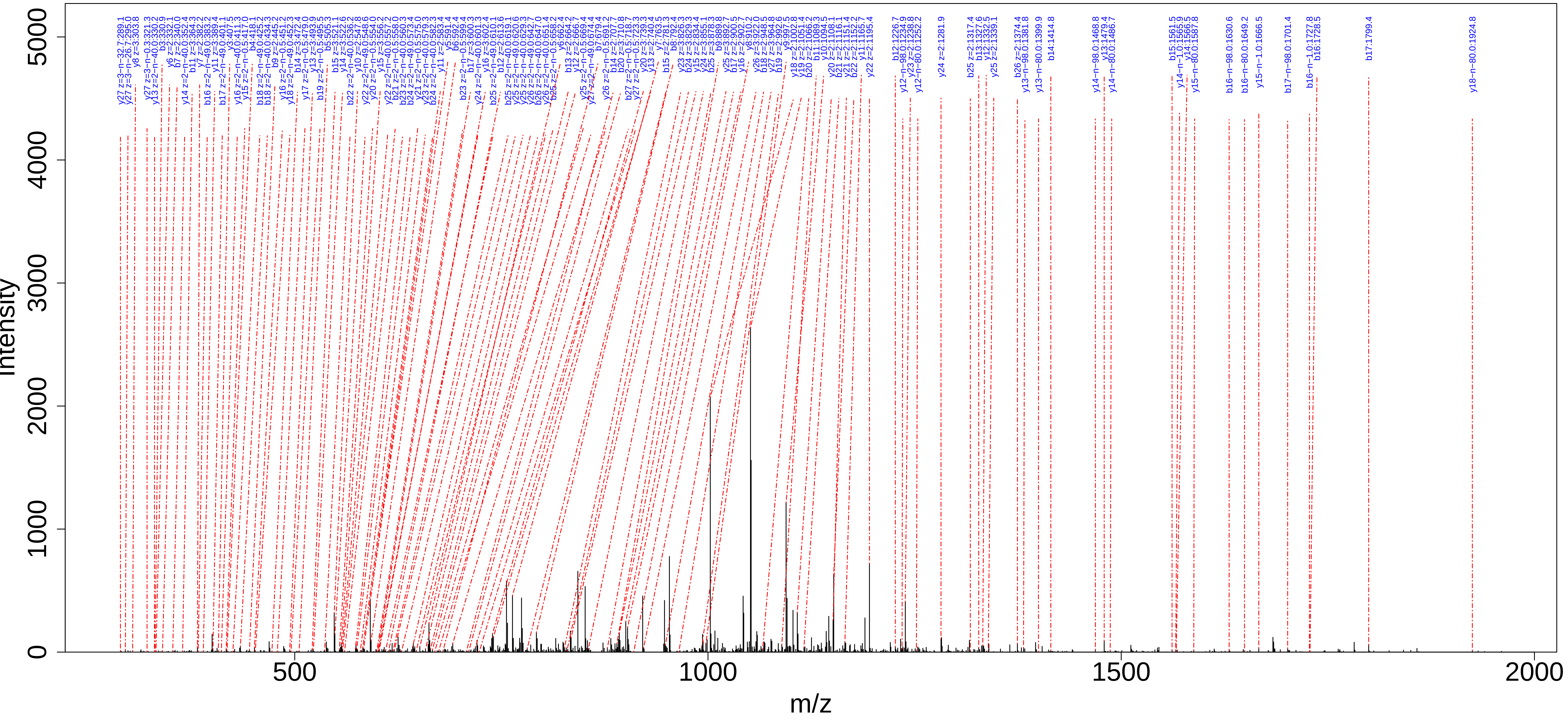

ESNSSSSLPSLHTSFSAPSFTAPSFLK 16−S_Phospho z=+3 Raw scan=4199
TBCD4_HUMAN S712; ; Q= 0 ; score= 48.6 ; E= 0.0019 m/z=965.1165

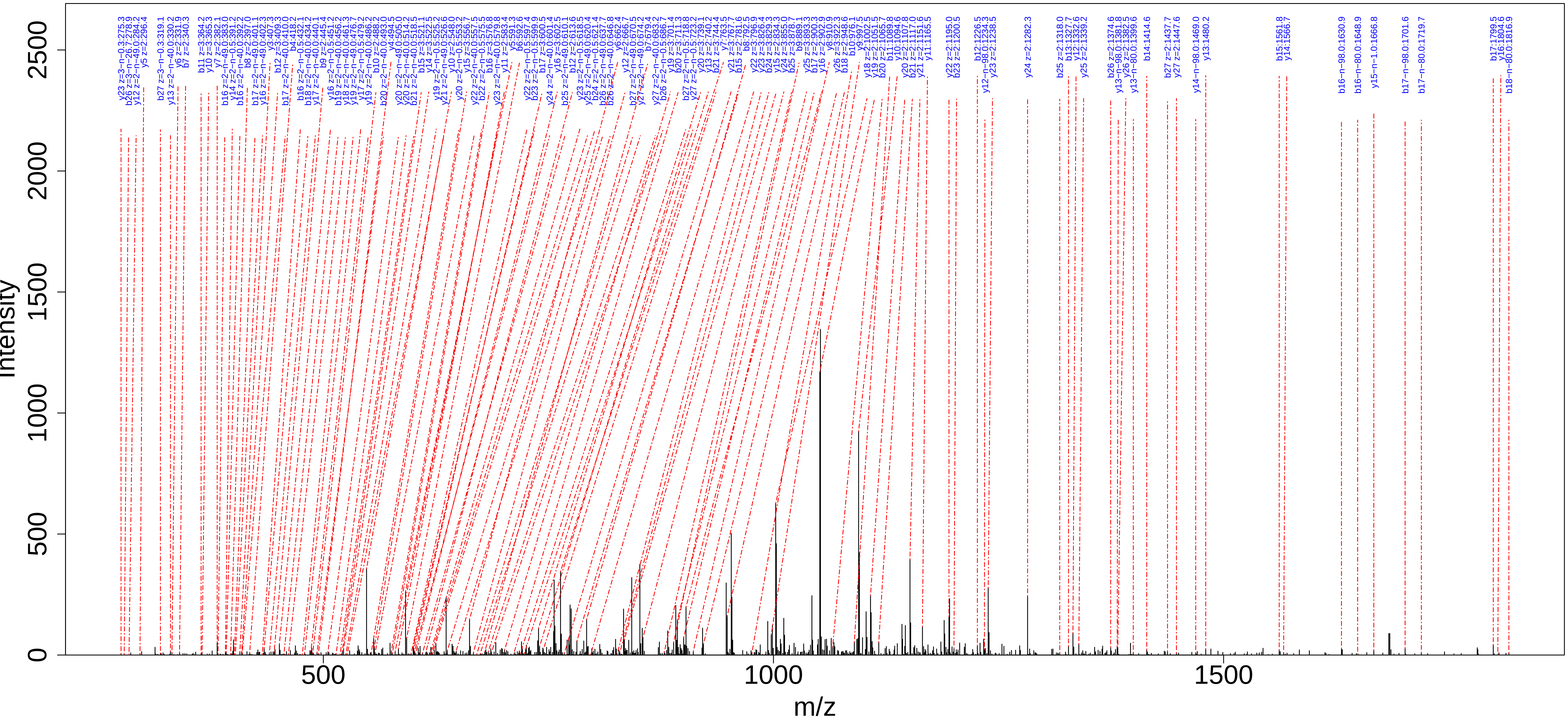

SFYQNSGRLSPQYENEIRQDTASESSDGEGR 10−S_Phospho z=+3 Raw scan=2790
TBCD4_HUMAN S725; ; Q= 0 ; score= 46.7 ; E= 0.0014 m/z=1196.5195

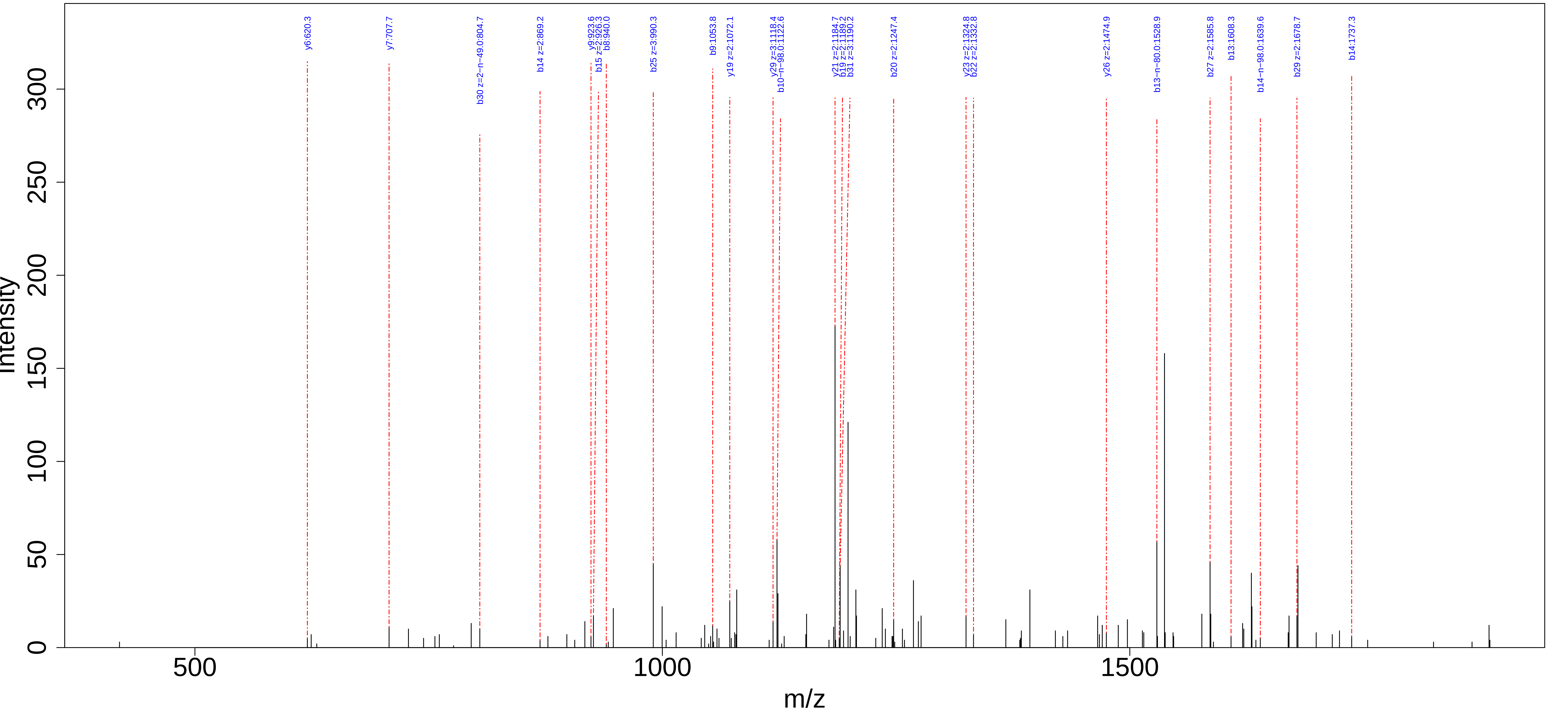

annotated raw MSMS spectra for assay:

Protein kinase: WNK1

Substrate: TBC1D4

sample name: TBCD4_WNK1_S4

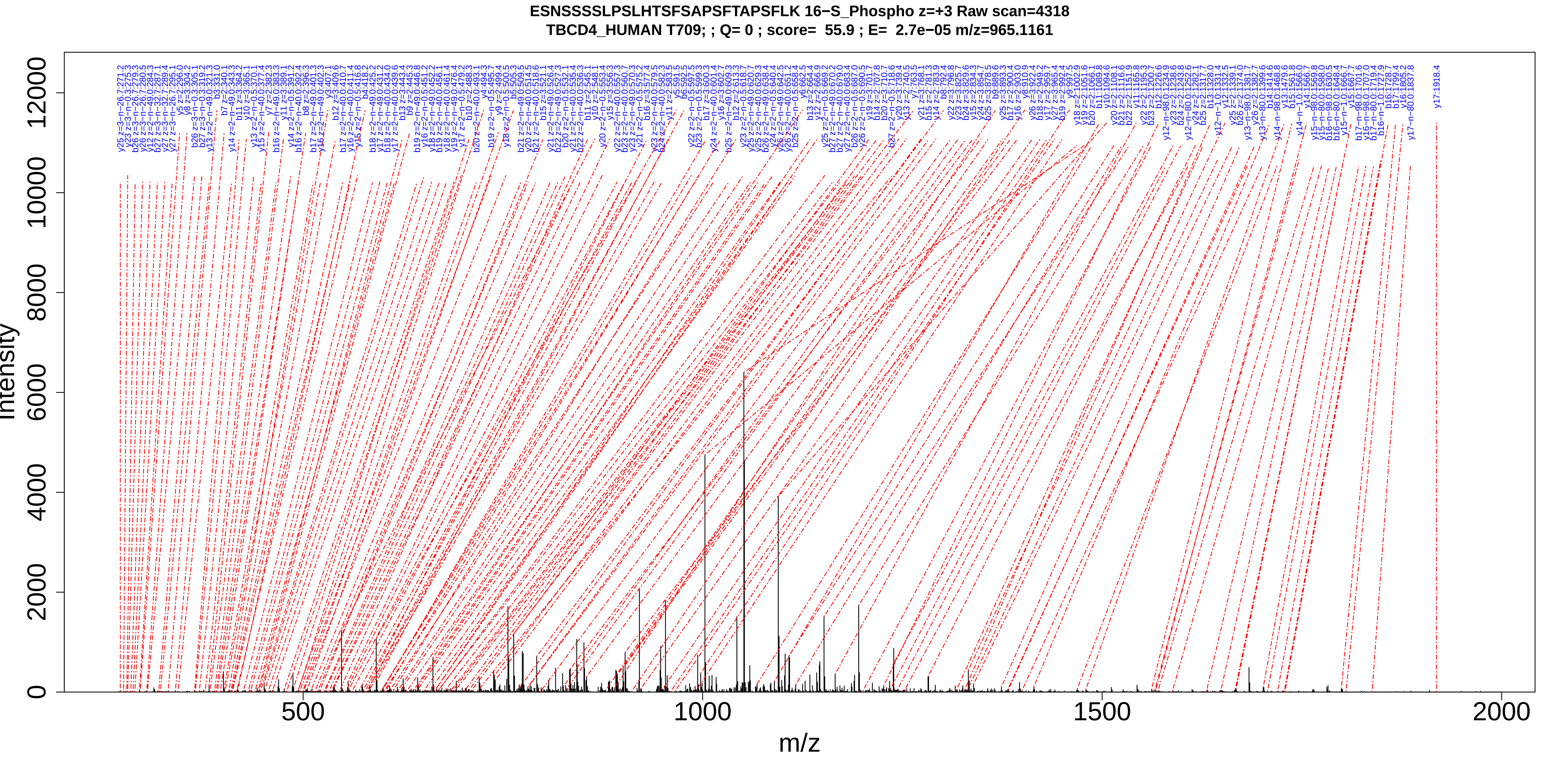
ESNSSSSLPSLHTSFSAPSFTAPSFLK 16−S_Phospho z=+3 Raw scan=4318
TBCD4_HUMAN T709; ; Q= 0 ; score= 55.9 ; E= 2.7e−05 m/z=965.1161
Intensity
m/z
0
2000
4000
6000
8000
10000
12000
500
1000
1500
2000

ESNSSSSLPSLHTSFSAPSFTAPSFLK 16−S_Phospho z=+3 Raw scan=4299
TBCD4_HUMAN S693; ; Q= 0 ; score= 55.9 ; E= 2.1e−05 m/z=965.1176

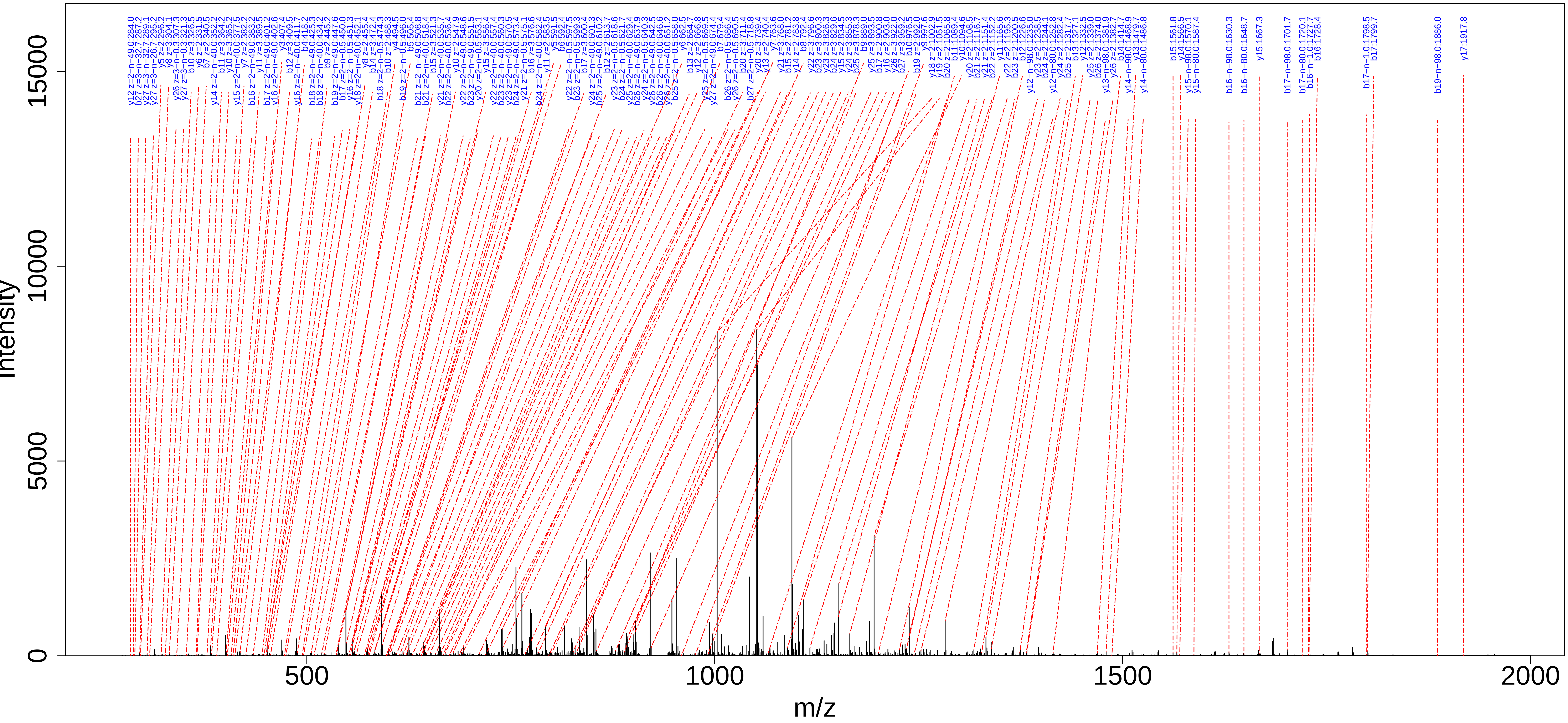

ESNSSSSLPSLHTSFSAPSFTAPSFLK 13−T_Phospho z=+3 Raw scan=4309
TBCD4_HUMAN S694; ; Q= 0 ; score= 55.6 ; E= 6.8e−05 m/z=965.1162

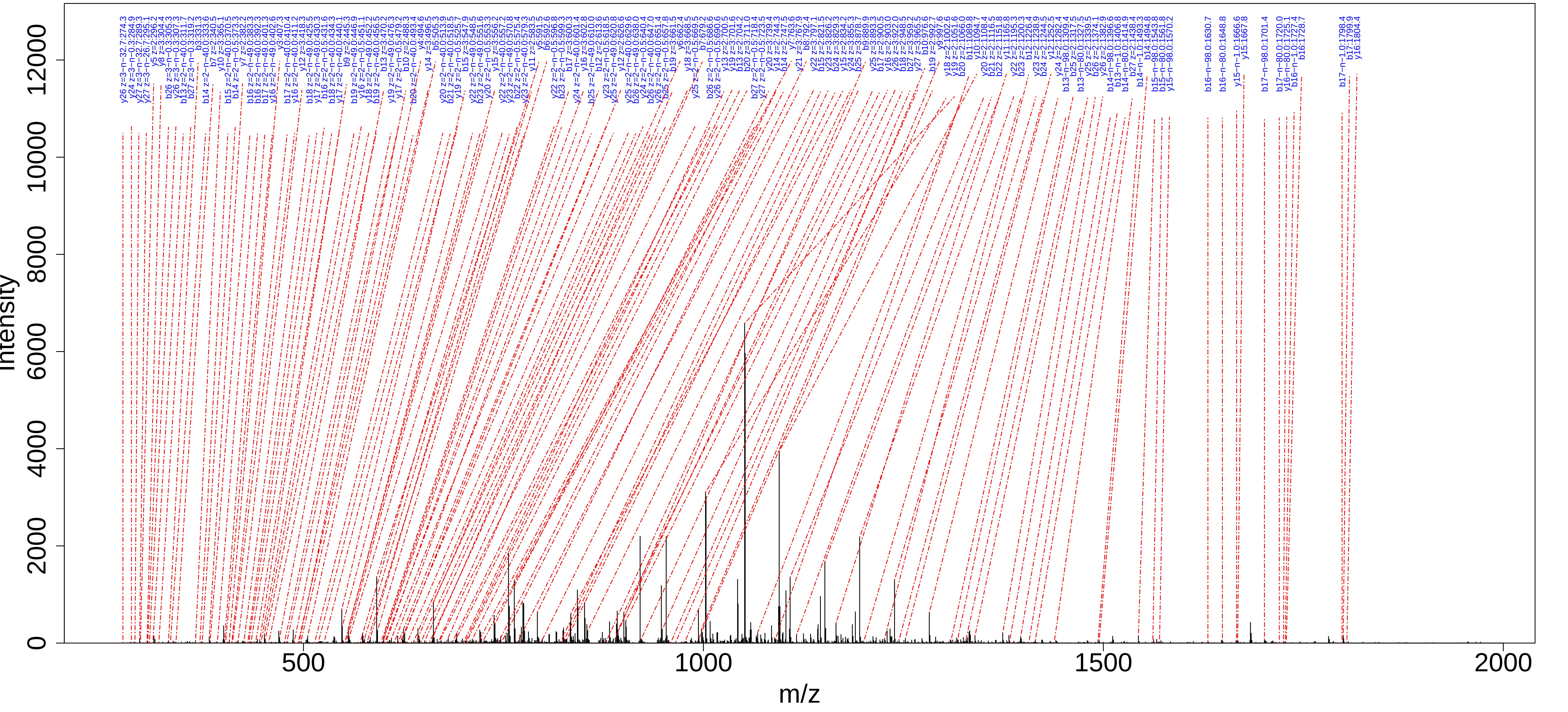

ESNSSSSLPSLHTSFSAPSFTAPSFLK 14−S_Phospho z=+3 Raw scan=4309
TBCD4_HUMAN S694; ; Q= 0 ; score= 55.6 ; E= 6.8e−05 m/z=965.1162

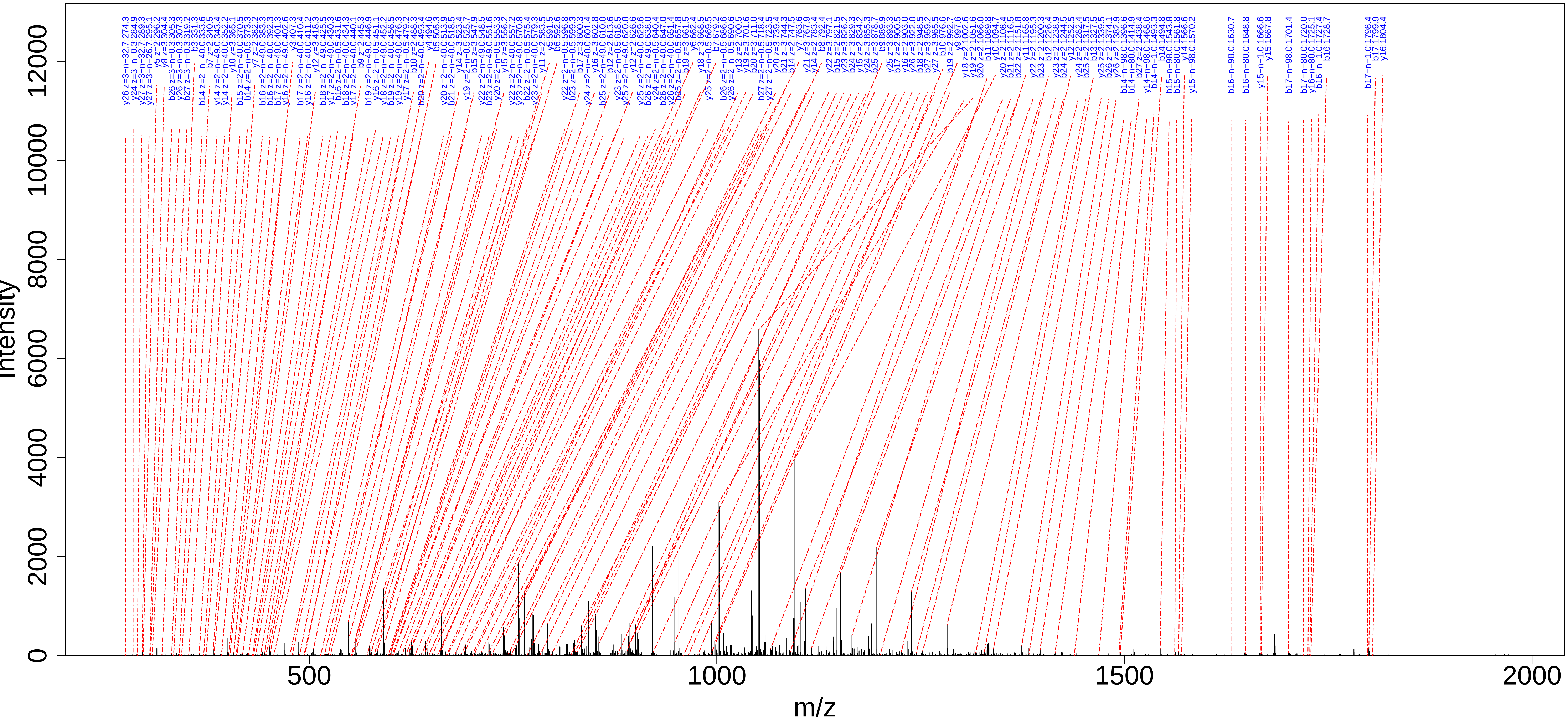

ESNSSSSLPSLHTSFSAPSFTAPSFLK 16−S_Phospho z=+3 Raw scan=4309
TBCD4_HUMAN S694; ; Q= 0 ; score= 55.6 ; E= 6.8e−05 m/z=965.1162

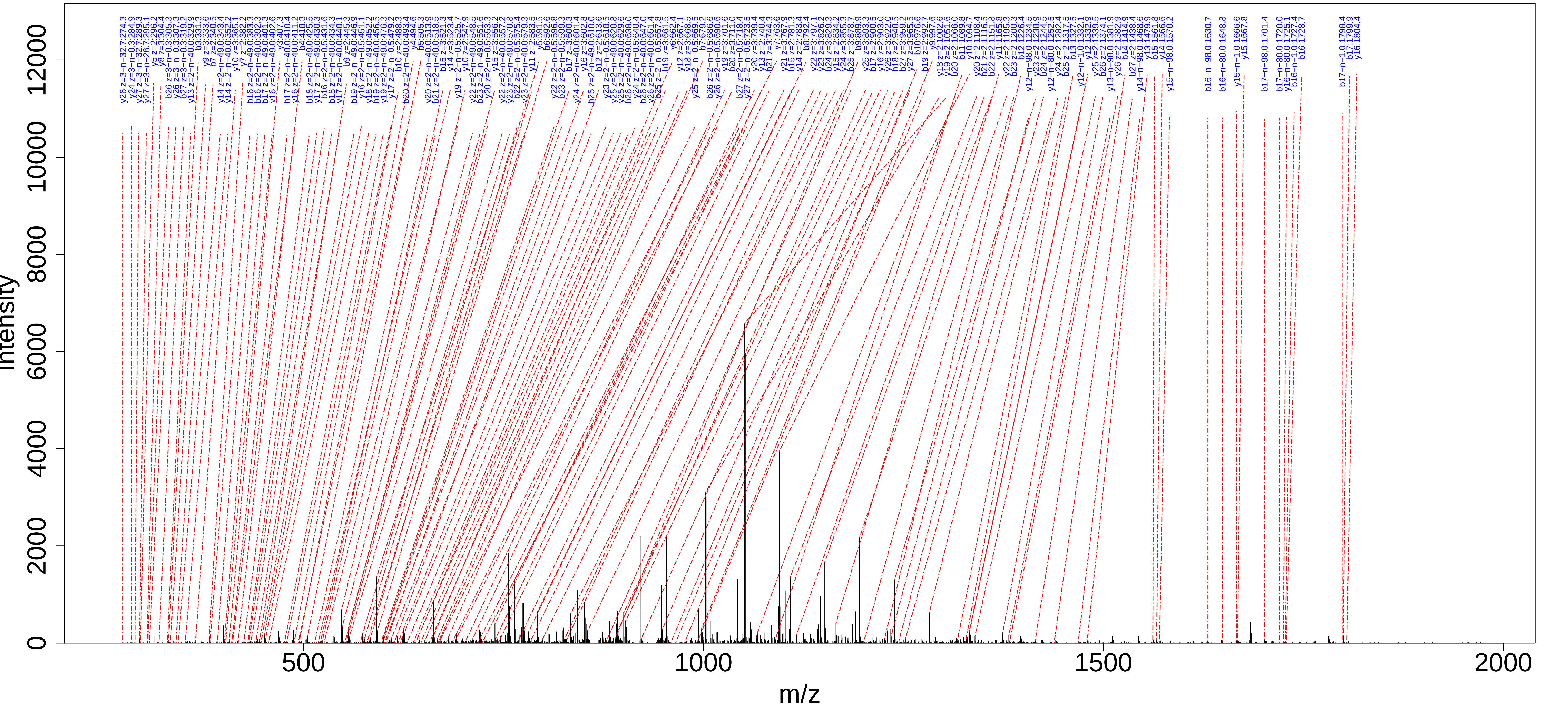

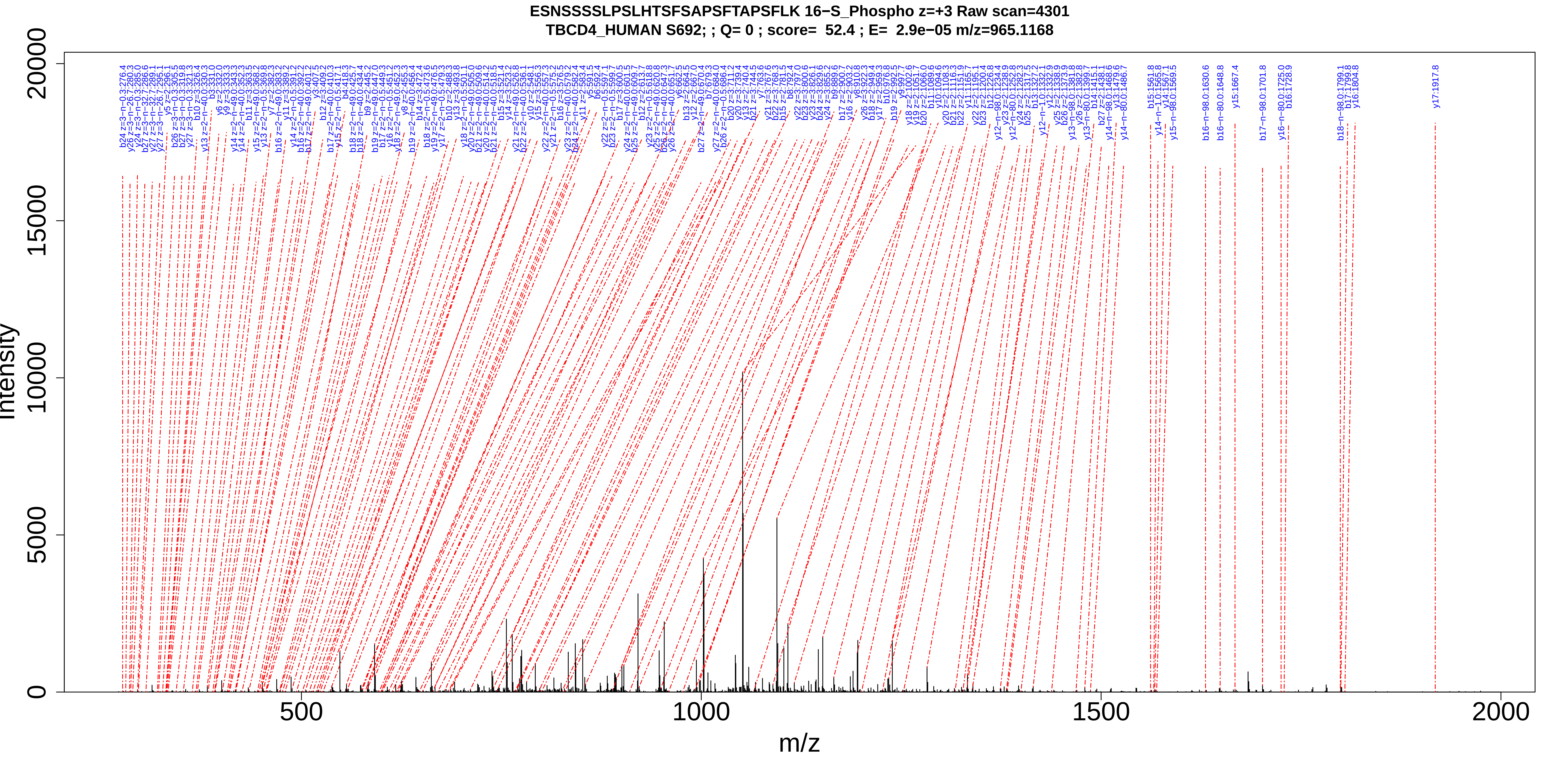
ESNSSSSLPSLHTSFSAPSFTAPSFLK 16−S_Phospho z=+3 Raw scan=4301
TBCD4_HUMAN S692; ; Q= 0 ; score= 52.4 ; E= 2.9e−05 m/z=965.1168
Intensity
0
5000
10000
15000
20000
500
1000
1500
2000
m/z

SFYQNSGRLSPQYENEIR 10−S_Phospho z=+3 Raw scan=2734
TBCD4_HUMAN S725; ; Q= 0.000254777070063694 ; score= 40.3 ; E= 0.019 m/z=756.6756

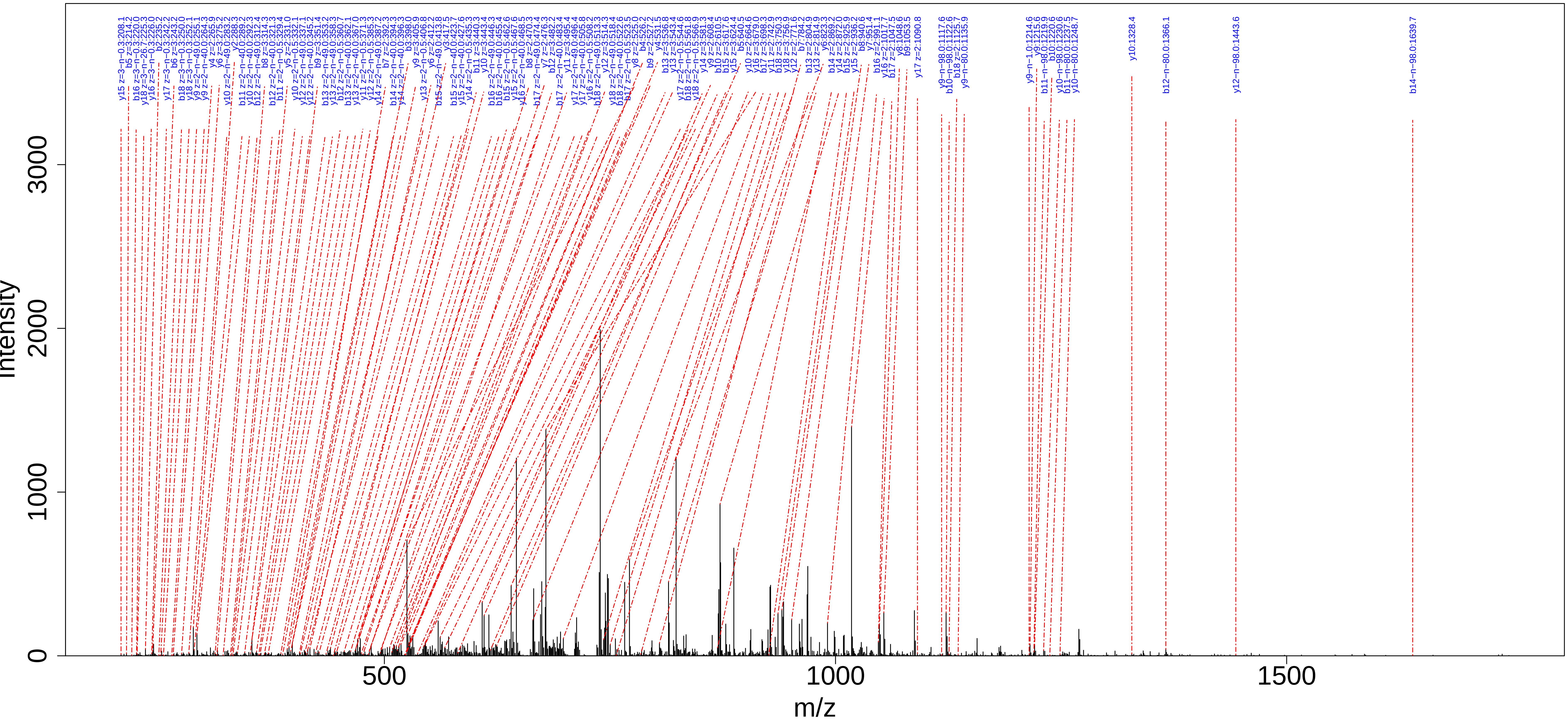

VTSGVKPASFDKVAIPEVK 9−S_Phospho z=+3 Raw scan=3083

WNK1_HUMAN T436; ; Q= 0.000252334090335604 ; score= 40.1 ; E= 0.022 m/z=684.6971

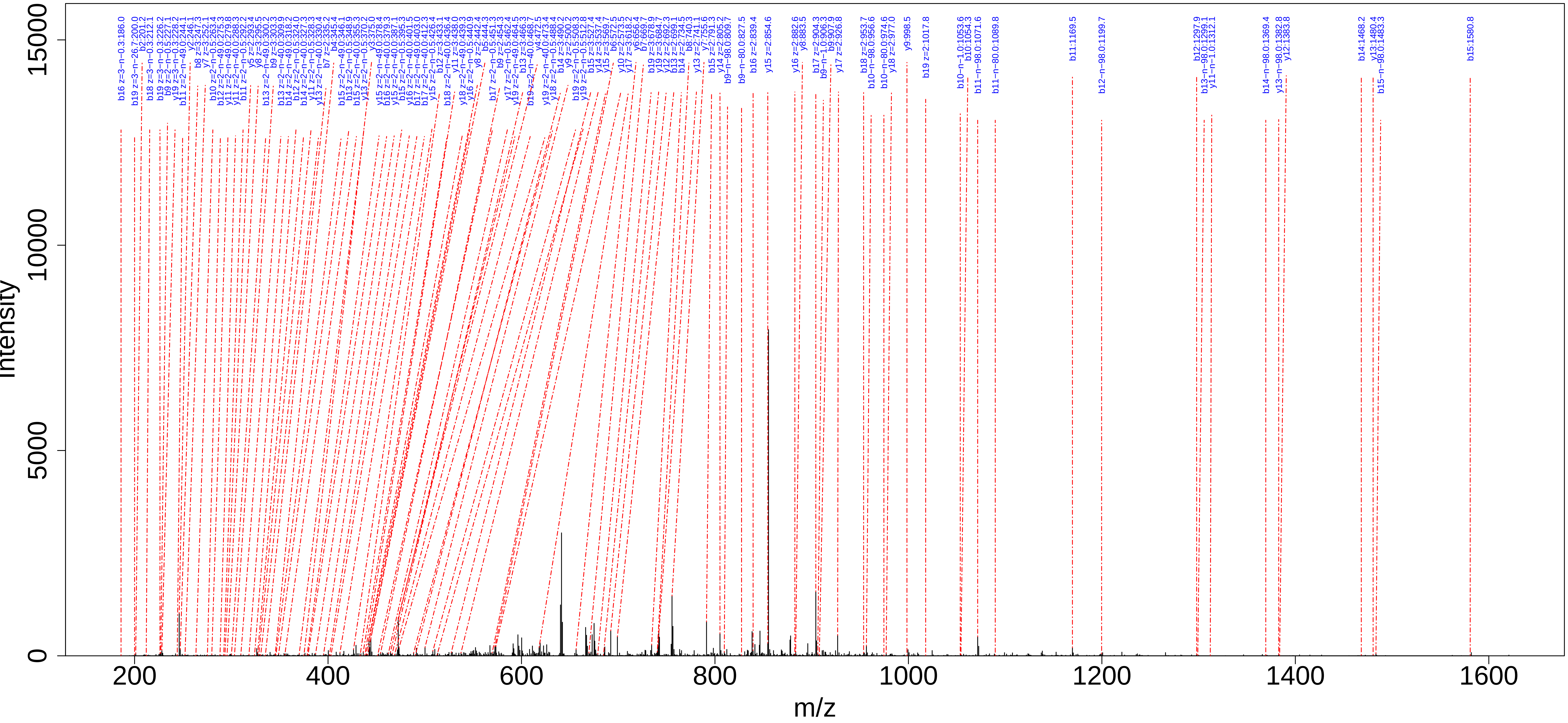

SFYQNSGRLSPQYENEIR 10−S_Phospho z=+3 Raw scan=2722
TBCD4_HUMAN S721; ; Q= 0.000242600679281902 ; score= 38.7 ; E= 0.023 m/z=756.6736

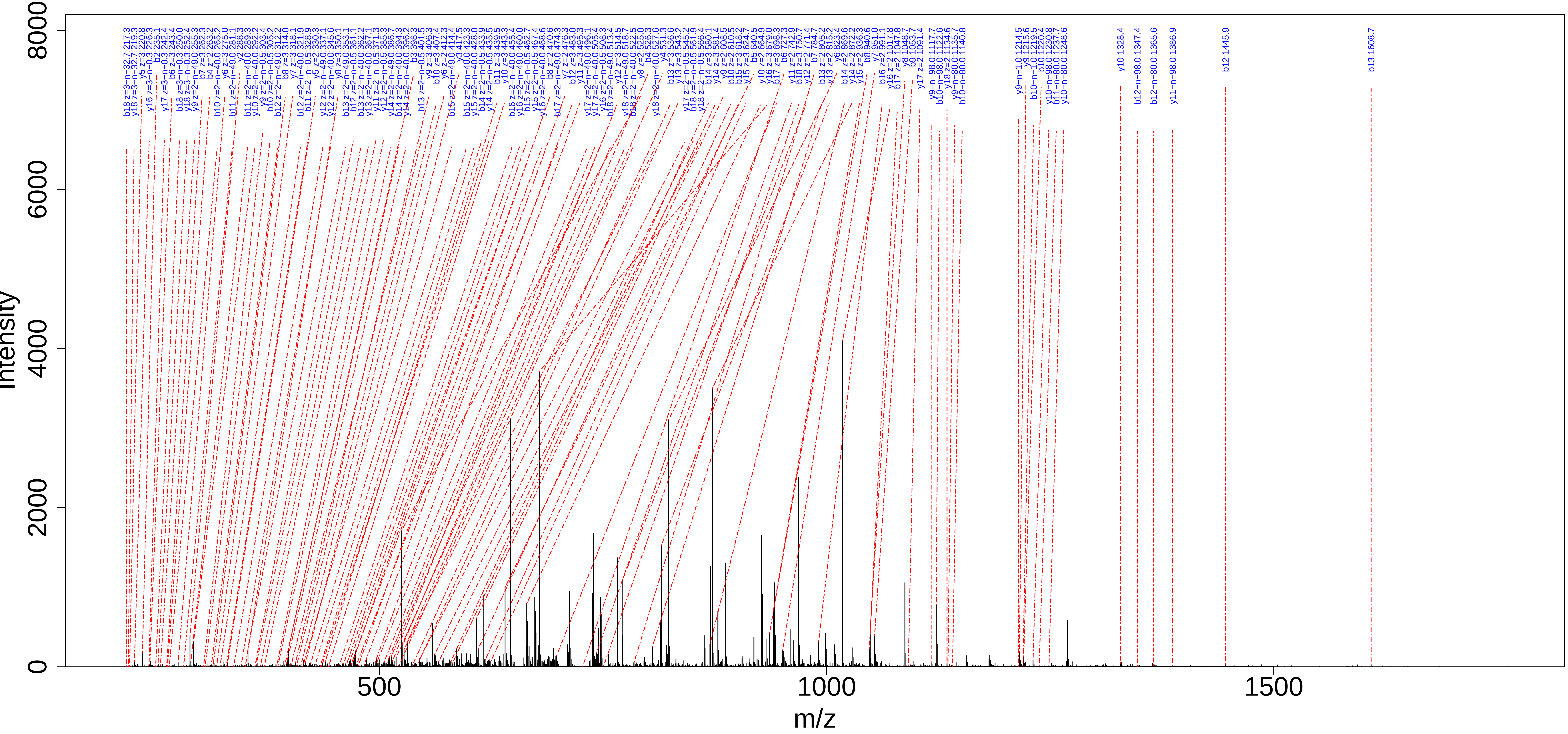

VTSGVKPASFDKVAIPEVK 9−S_Phospho z=+3 Raw scan=3068
WNK1_HUMAN S437; ; Q= 0.000214178624973228 ; score= 34.3 ; E= 0.07 m/z=684.6985

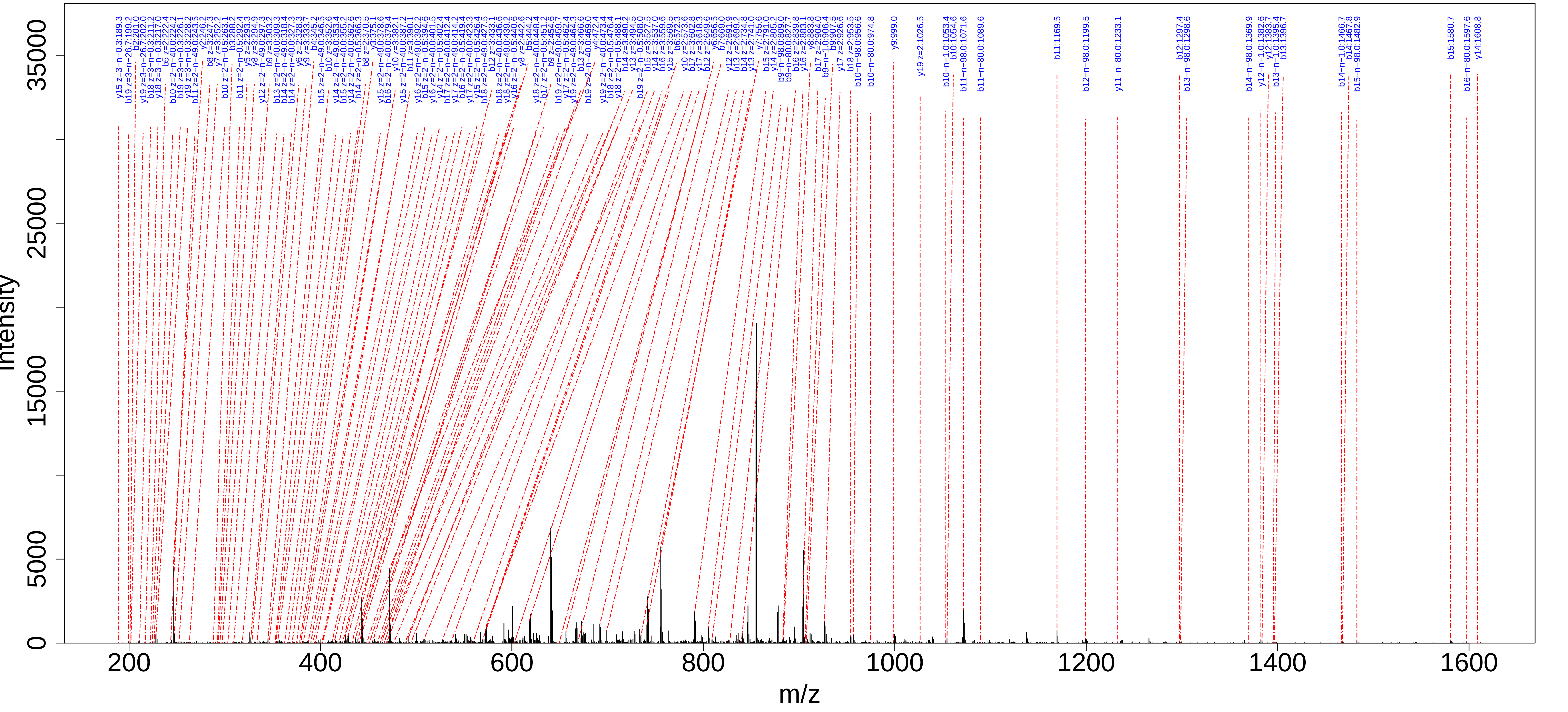

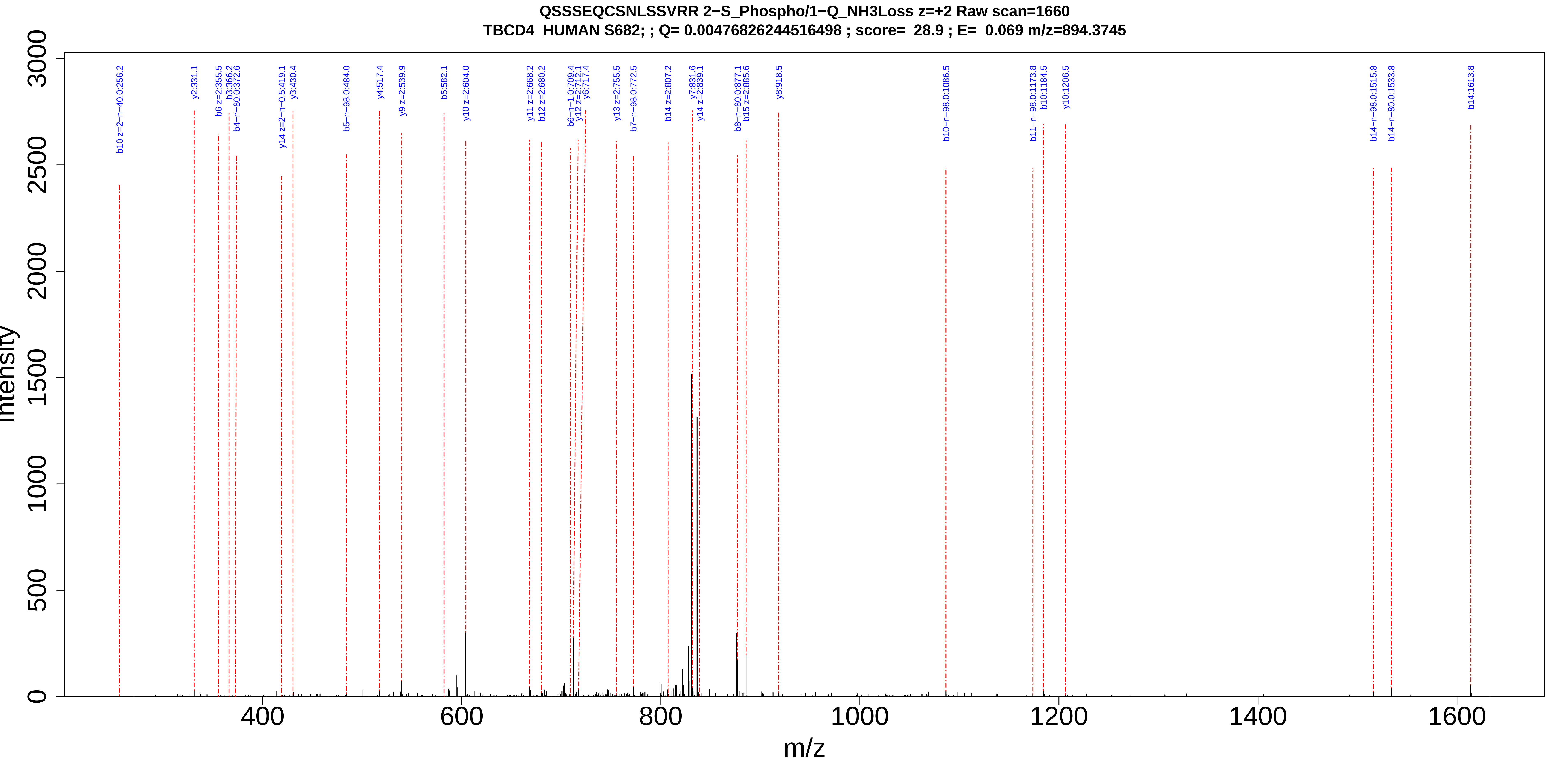
QSSSEQCSNLSSVRR 2−S_Phospho/1−Q_NH3Loss z=+2 Raw scan=1660
TBCD4_HUMAN S682; ; Q= 0.00476826244516498 ; score= 28.9 ; E= 0.069 m/z=894.3745
Intensity
m/z

QSSSEQCSNLSSVRR 3−S_Phospho/1−Q_NH3Loss z=+2 Raw scan=1660
TBCD4_HUMAN S682; ; Q= 0.0047673531655225 ; score= 28.9 ; E= 0.069 m/z=894.3745

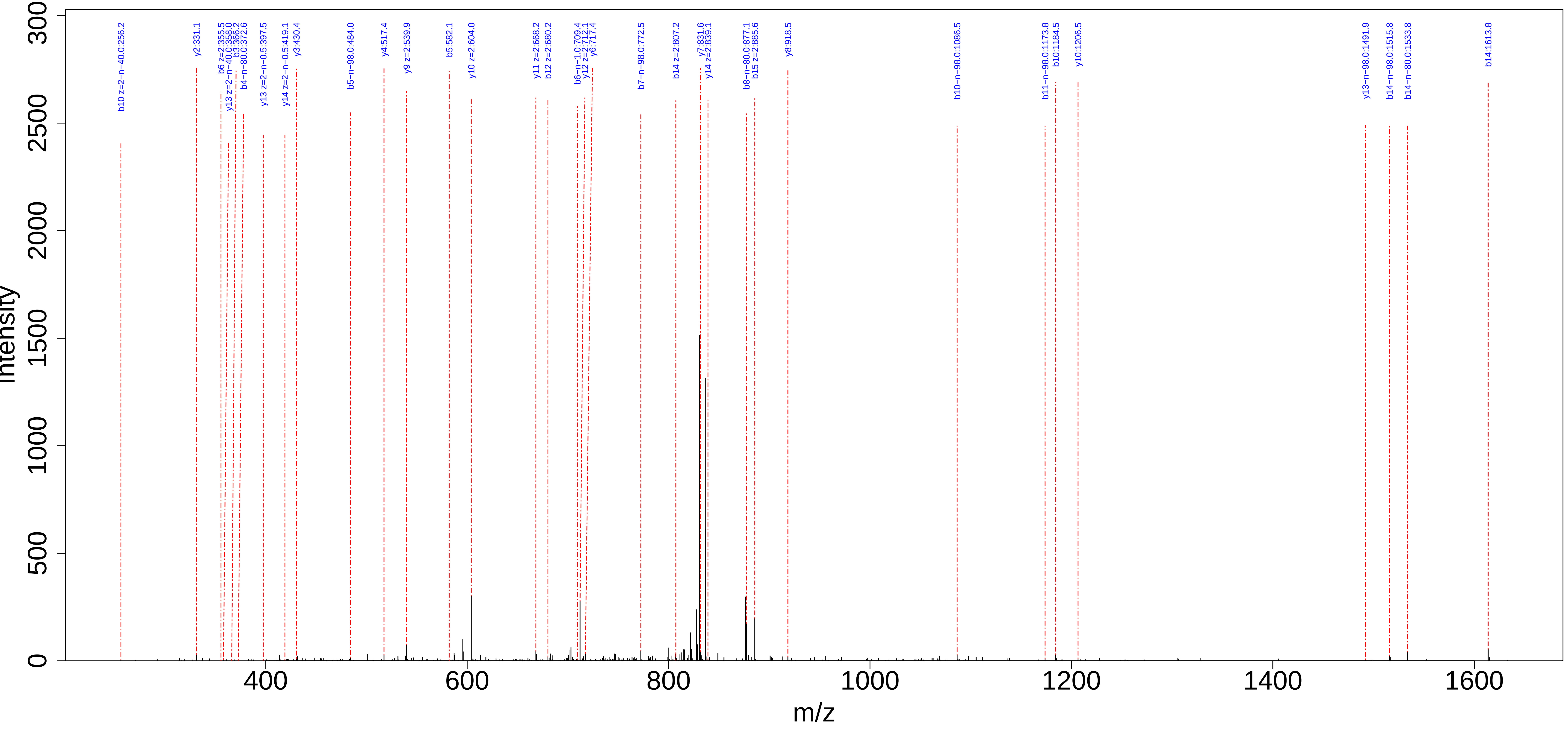

annotated raw MSMS spectra for assay:

Protein kinase: WNK1

Substrate: TBC1D4

sample name: WNK1_TBCD4_4

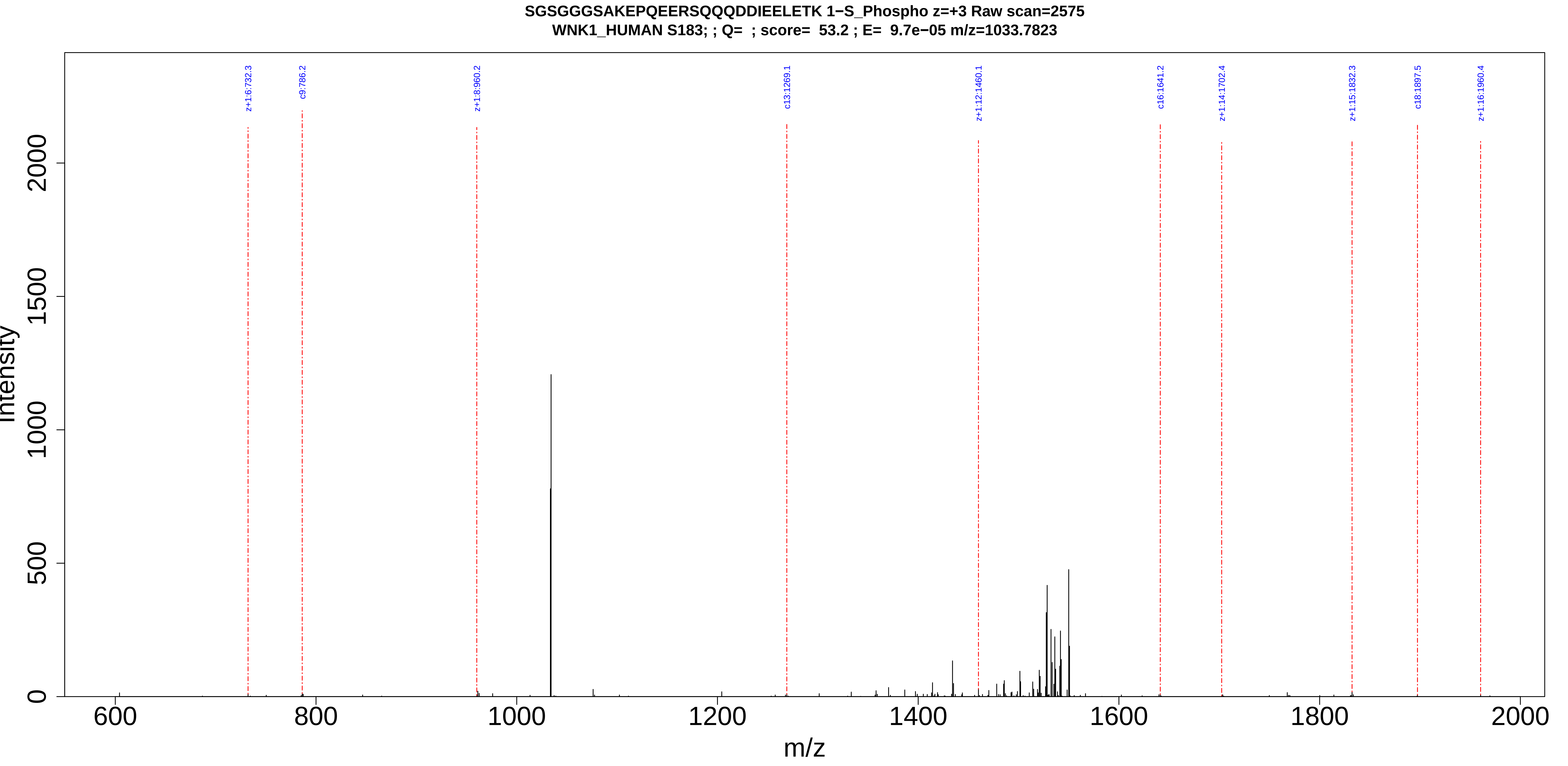

SGSGGGSAKEPQEERSQQQDDIEELETK 1−S_Phospho z=+3 Raw scan=2575
WNK1_HUMAN S183; ; Q= ; score= 53.2 ; E= 9.7e−05 m/z=1033.7823
z+1:6:732.3
c9:786.2
z+1:8:960.2
c13:1269.1
z+1:12:1460.1
c16:1641.2
z+1:14:1702.4
z+1:15:1832.3
c18:1897.5
z+1:16:1960.4
Intensity
0
500
1000
1500
2000
600
800
1000
1200
1400
1600
1800
2000
m/z

SGSGGGSAKEPQEERSQQQDDIEELETK 3−S_Phospho z=+3 Raw scan=2575
WNK1_HUMAN S185; ; Q= ; score= 53.2 ; E= 9.7e−05 m/z=1033.7823

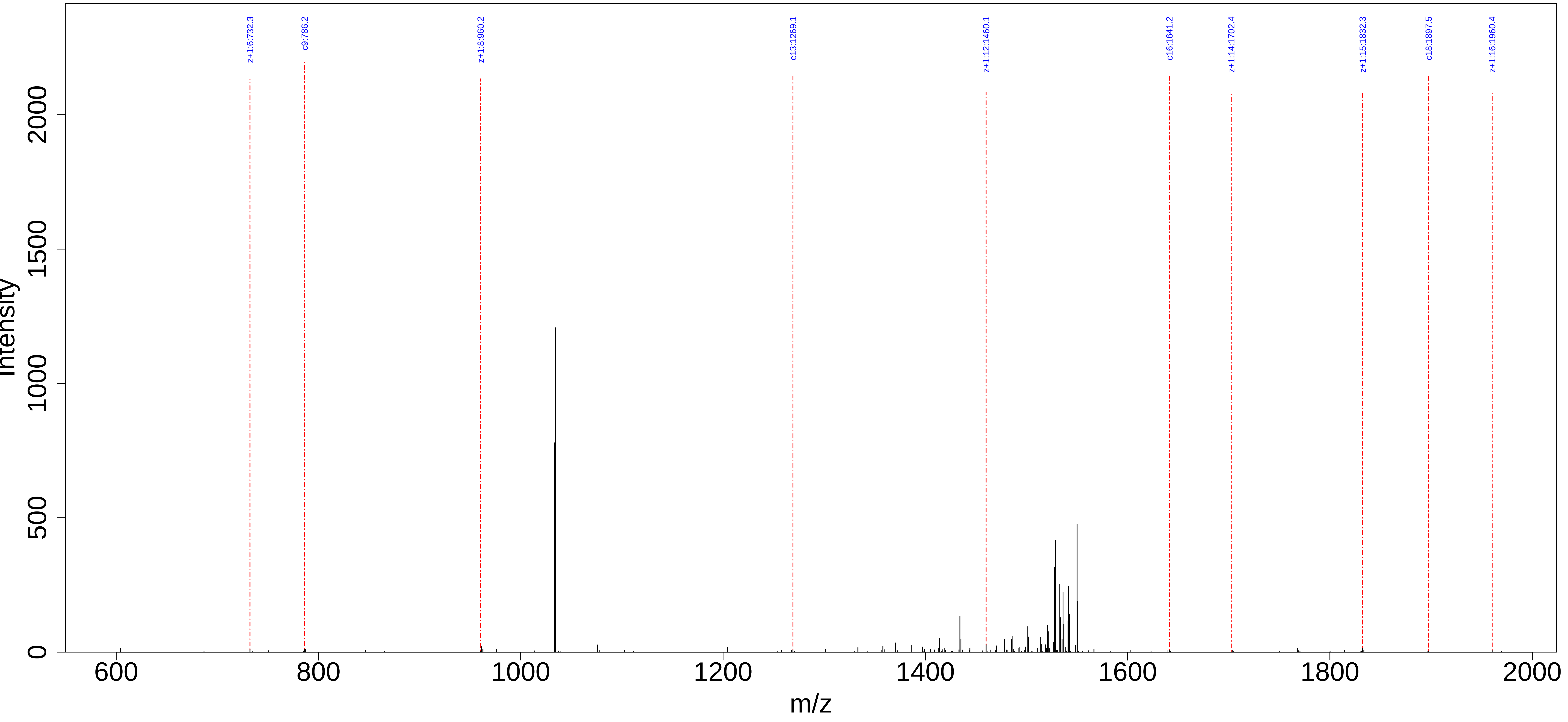

SGSGGGSAKEPQEERSQQQDDIEELETK 7−S_Phospho z=+3 Raw scan=2575
WNK1_HUMAN S189; ; Q= ; score= 53.2 ; E= 9.7e−05 m/z=1033.7823

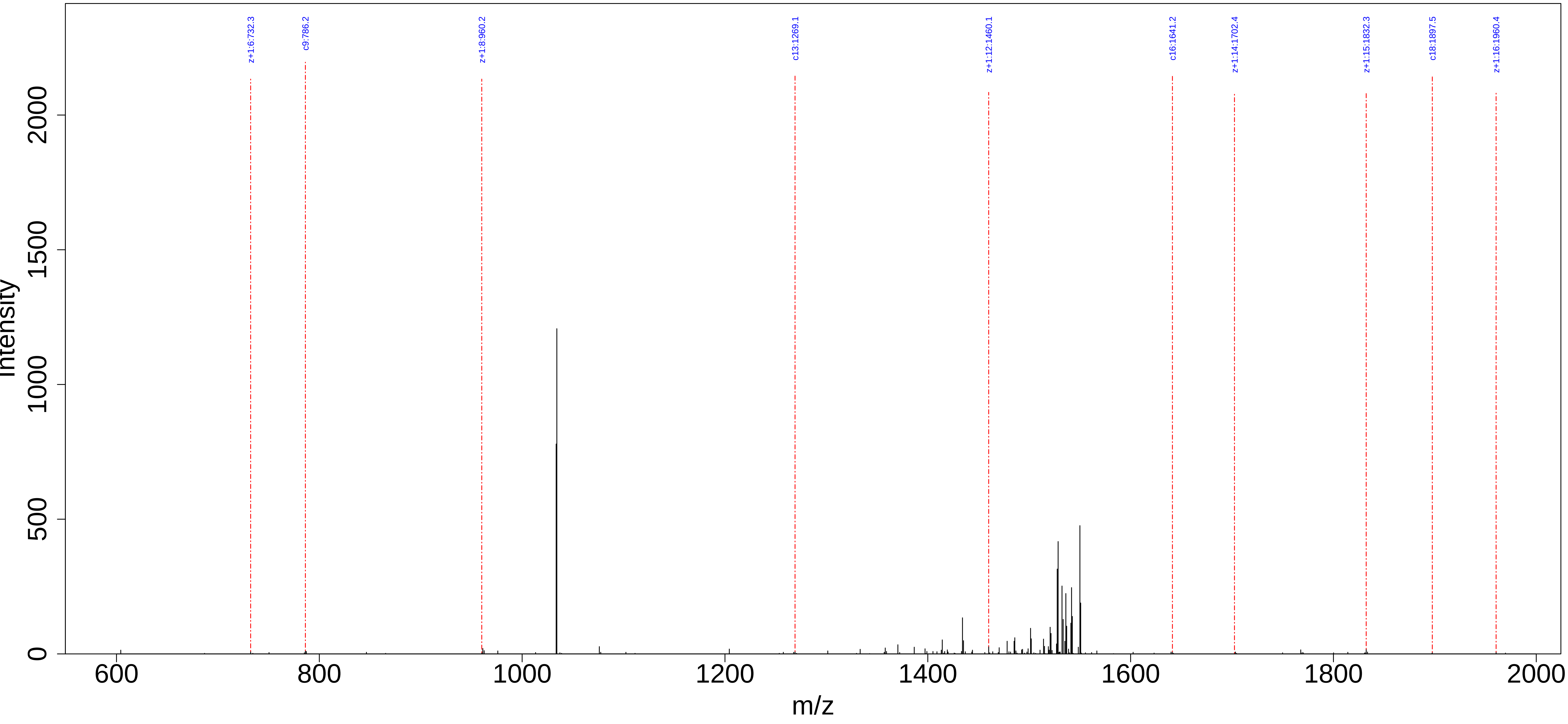

SGSGGGSAKEPQEERSQQQDDIEELETK 1−S_Phospho z=+3 Raw scan=2566
WNK1_HUMAN S183; ; Q= ; score= 51.6 ; E= 7e−05 m/z=1033.783

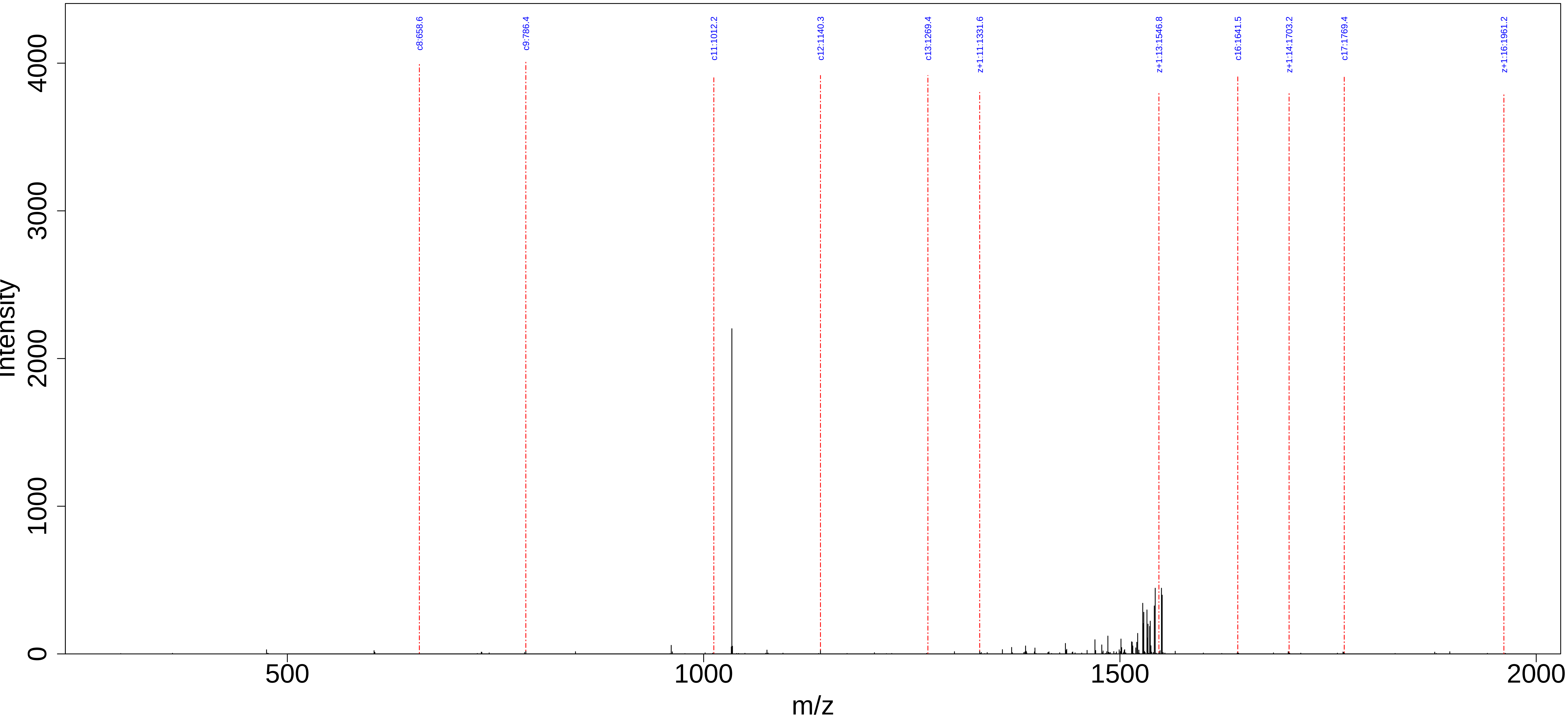

SGSGGGSAKEPQEERSQQQDDIEELETK 3−S_Phospho z=+3 Raw scan=2566
WNK1_HUMAN S185; ; Q= ; score= 51.6 ; E= 7e−05 m/z=1033.783

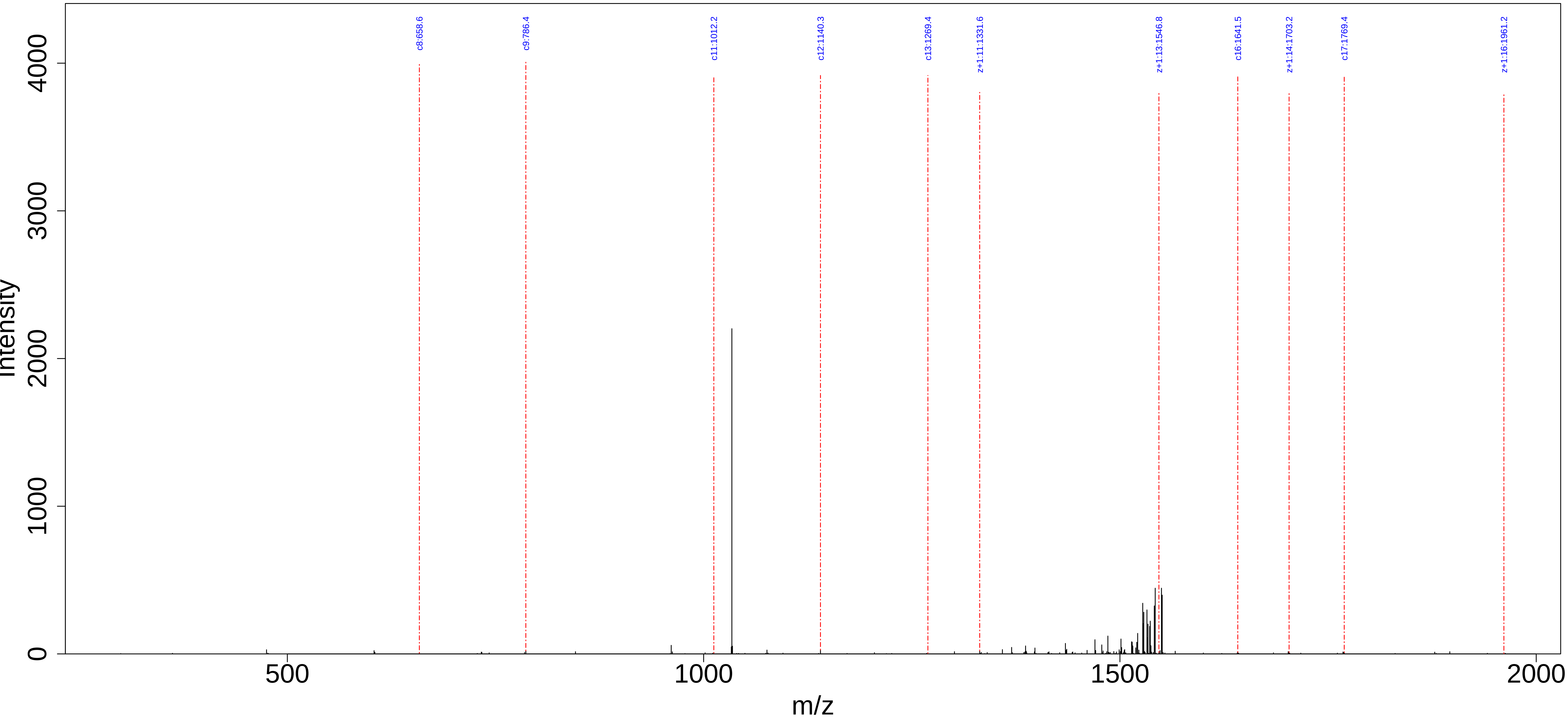

SGSGGGSAKEPQEERSQQQDDIEELETK 7−S_Phospho z=+3 Raw scan=2566
WNK1_HUMAN S189; ; Q= ; score= 51.6 ; E= 7e−05 m/z=1033.783

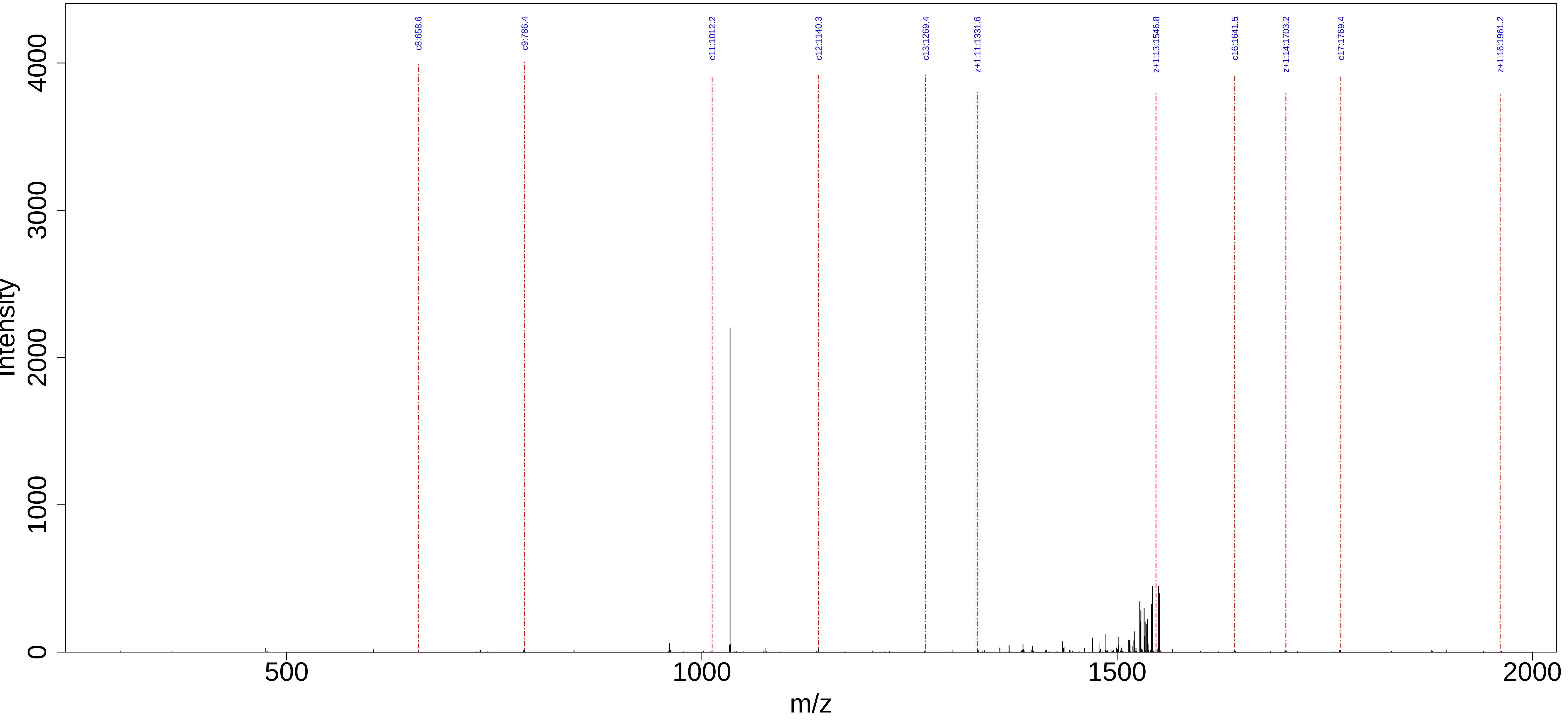

SGSGGGSAKEPQEERSQQQDDIEELETK 1−S_Phospho z=+3 Raw scan=2622
WNK1_HUMAN S183; ; Q= ; score= 29.3 ; E= 0.082 m/z=1033.7883

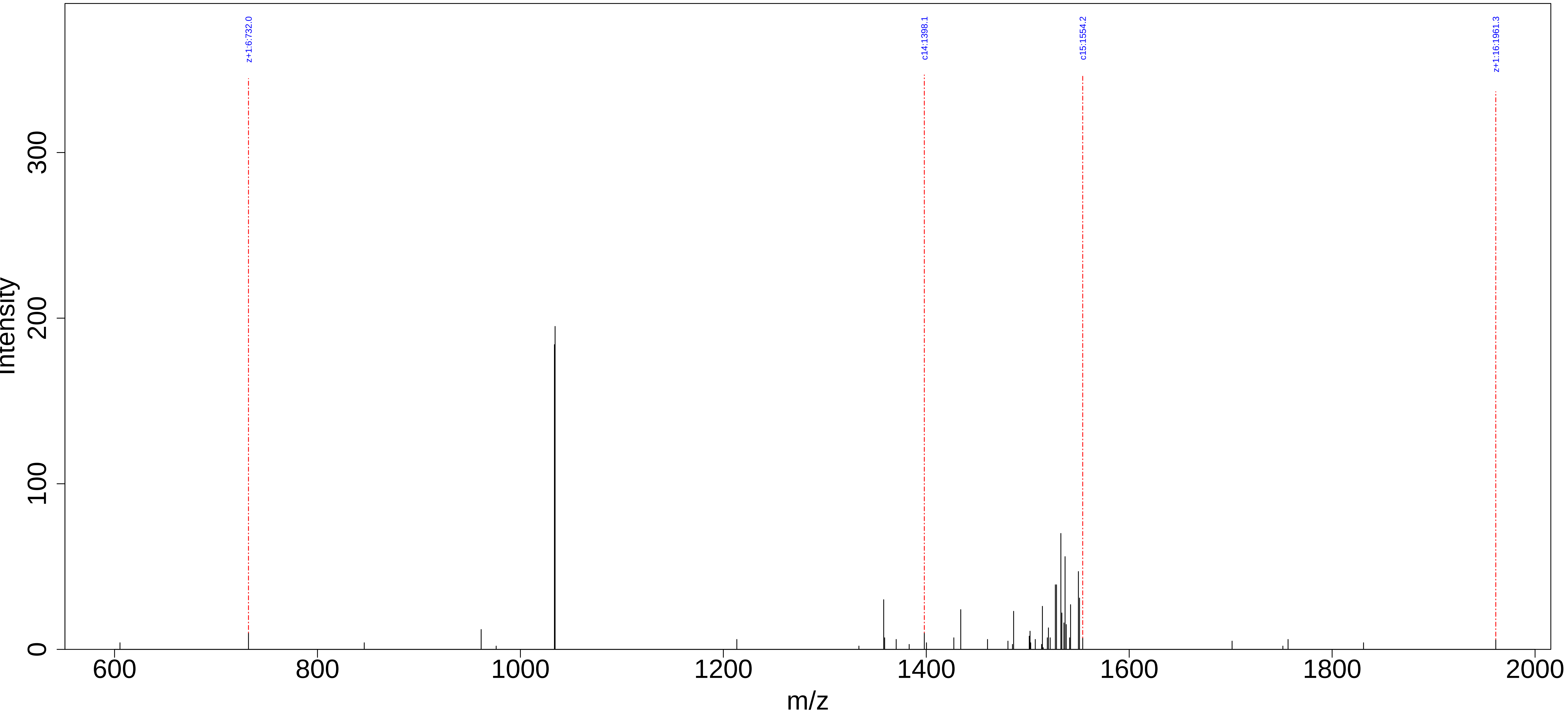

SGSGGGSAKEPQEERSQQQDDIEELETK 3−S_Phospho z=+3 Raw scan=2622
WNK1_HUMAN S185; ; Q= ; score= 29.3 ; E= 0.082 m/z=1033.7883

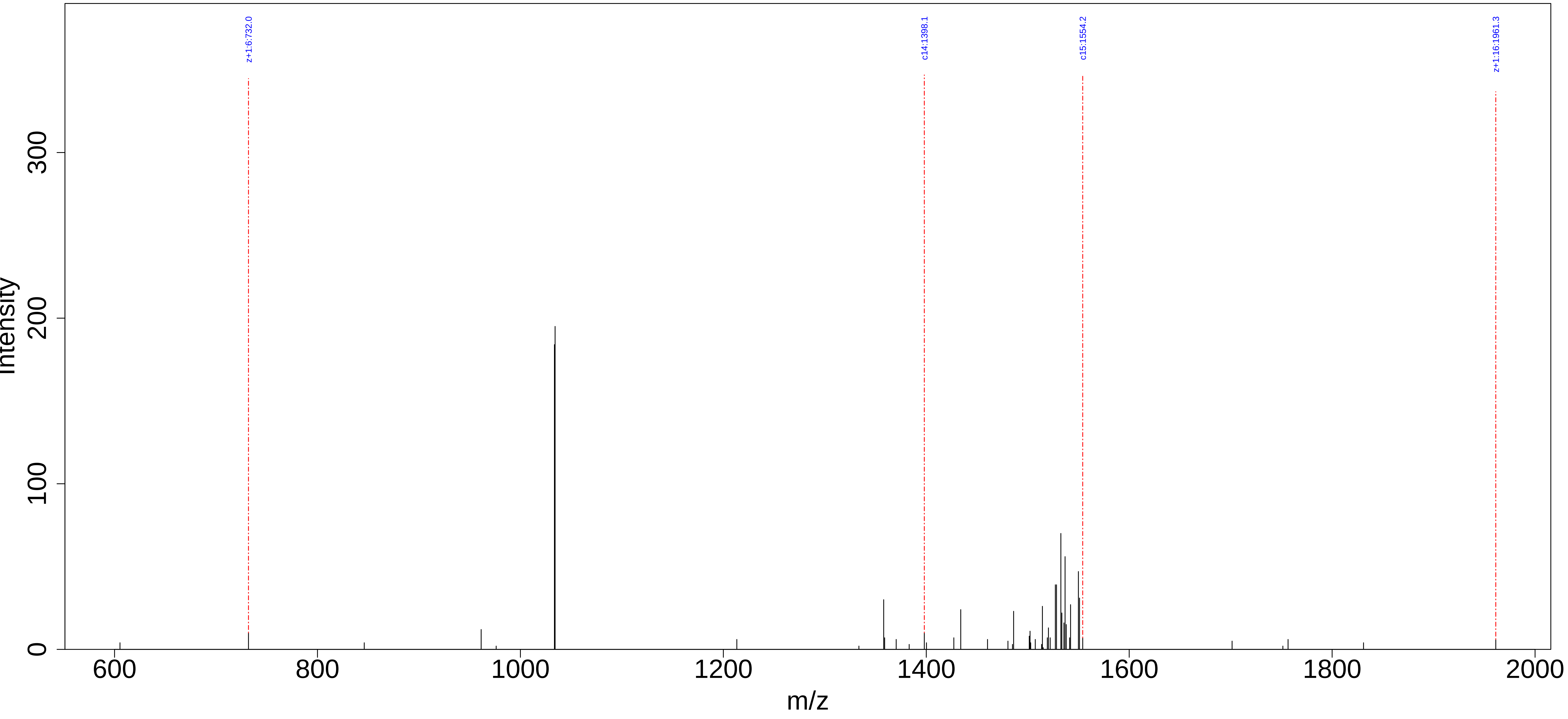

SGSGGGSAKEPQEERSQQQDDIEELETK 7−S_Phospho z=+3 Raw scan=2622
WNK1_HUMAN S189; ; Q= ; score= 29.3 ; E= 0.082 m/z=1033.7883

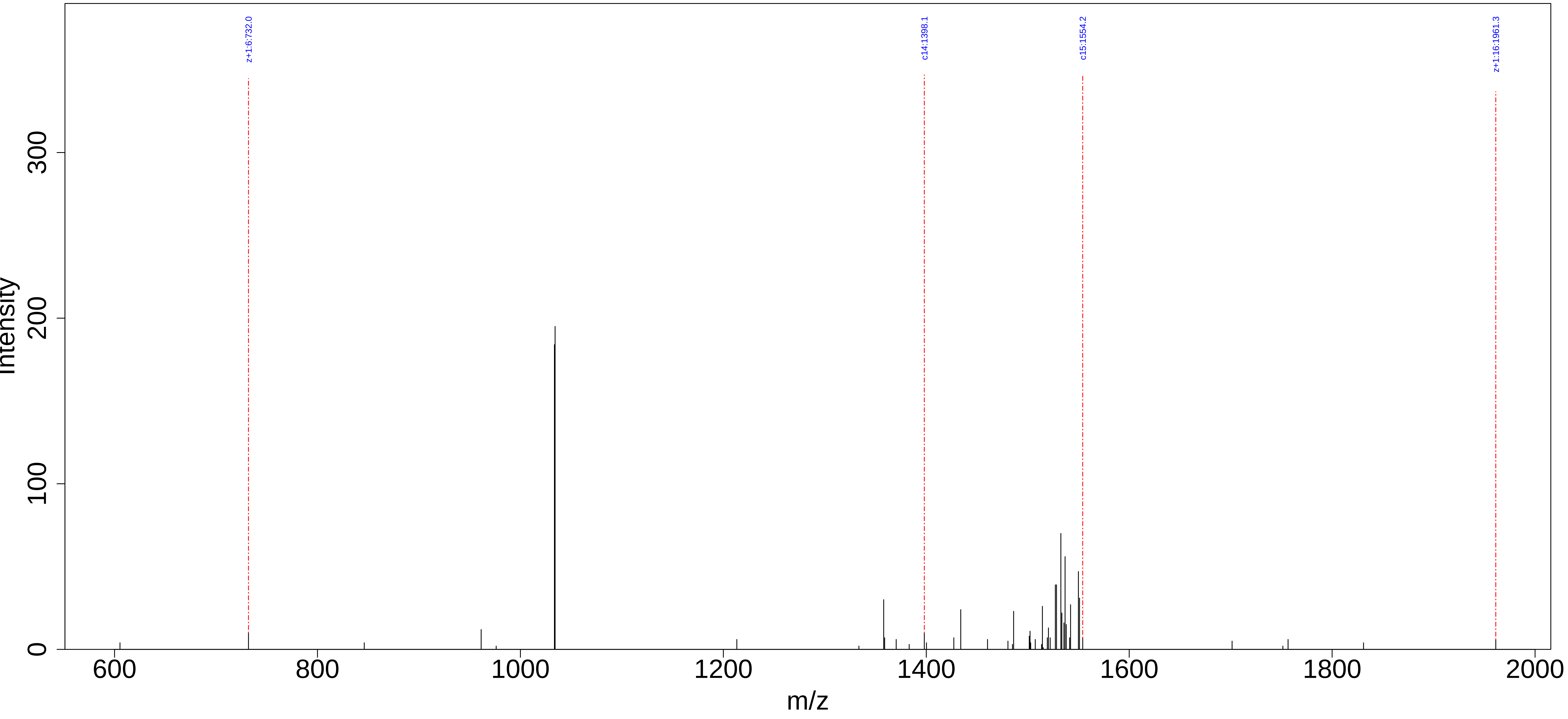

annotated raw MSMS spectra for assay:

Protein kinase: SGK1

Substrate: TBC1D1

sample name: TBCD1_SGK1_S5

ANTLSHFPIECQEPPQPAR 3−T_Phospho z=+3 Raw scan=2986

TBCD1_HUMAN T596; ; Q= 0 ; score= 76.5 ; E= 1.8e−11 m/z=758.0114

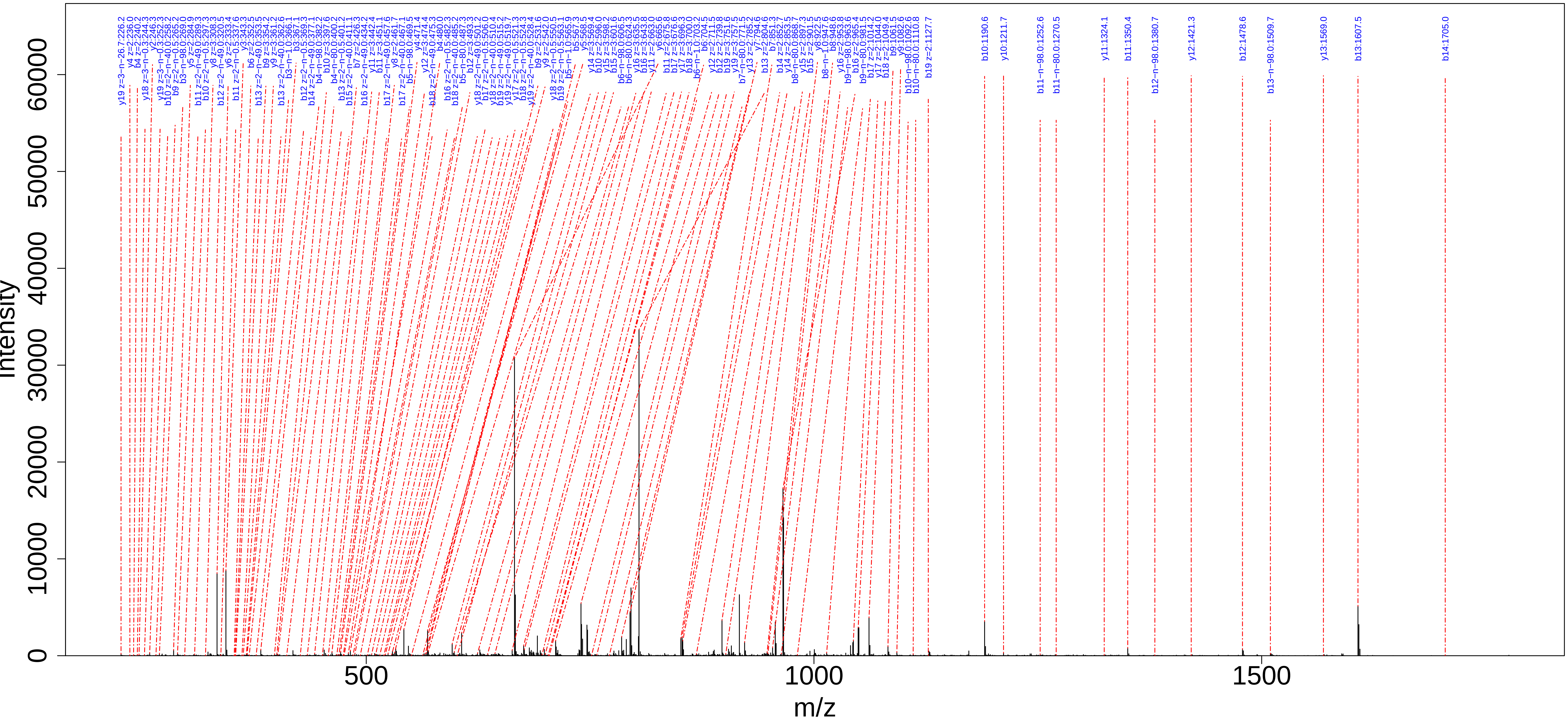

RANTLSHFPIECQEPPQPAR 4−T_Phospho z=+3 Raw scan=2683
TBCD1_HUMAN T596; ; Q= 0 ; score= 68.8 ; E= 8.3e−10 m/z=810.0457

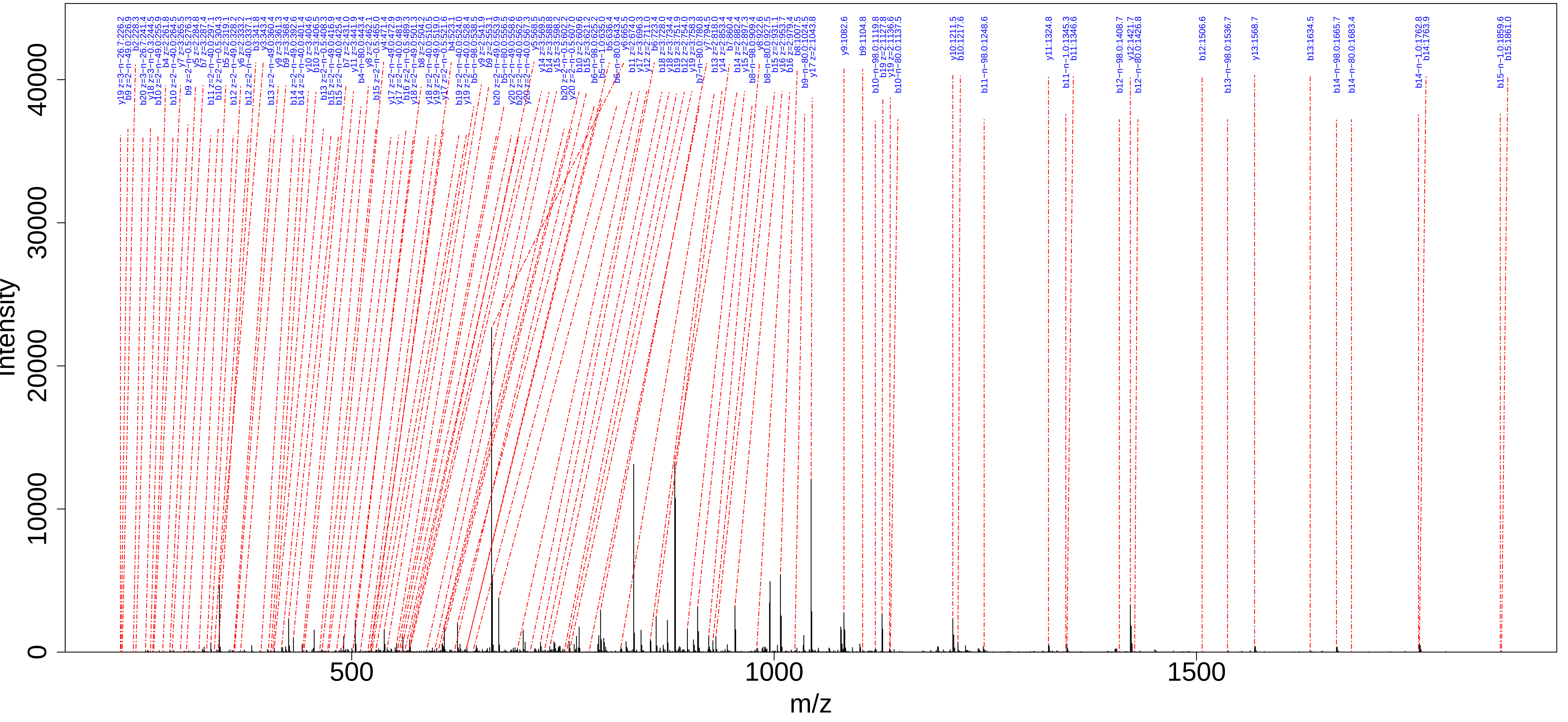

ANTLSHFPIECQEPPQPAR 3−T_Phospho z=+3 Raw scan=3016
TBCD1_HUMAN T596; ; Q= 0 ; score= 64.9 ; E= 1.8e−06 m/z=758.0107

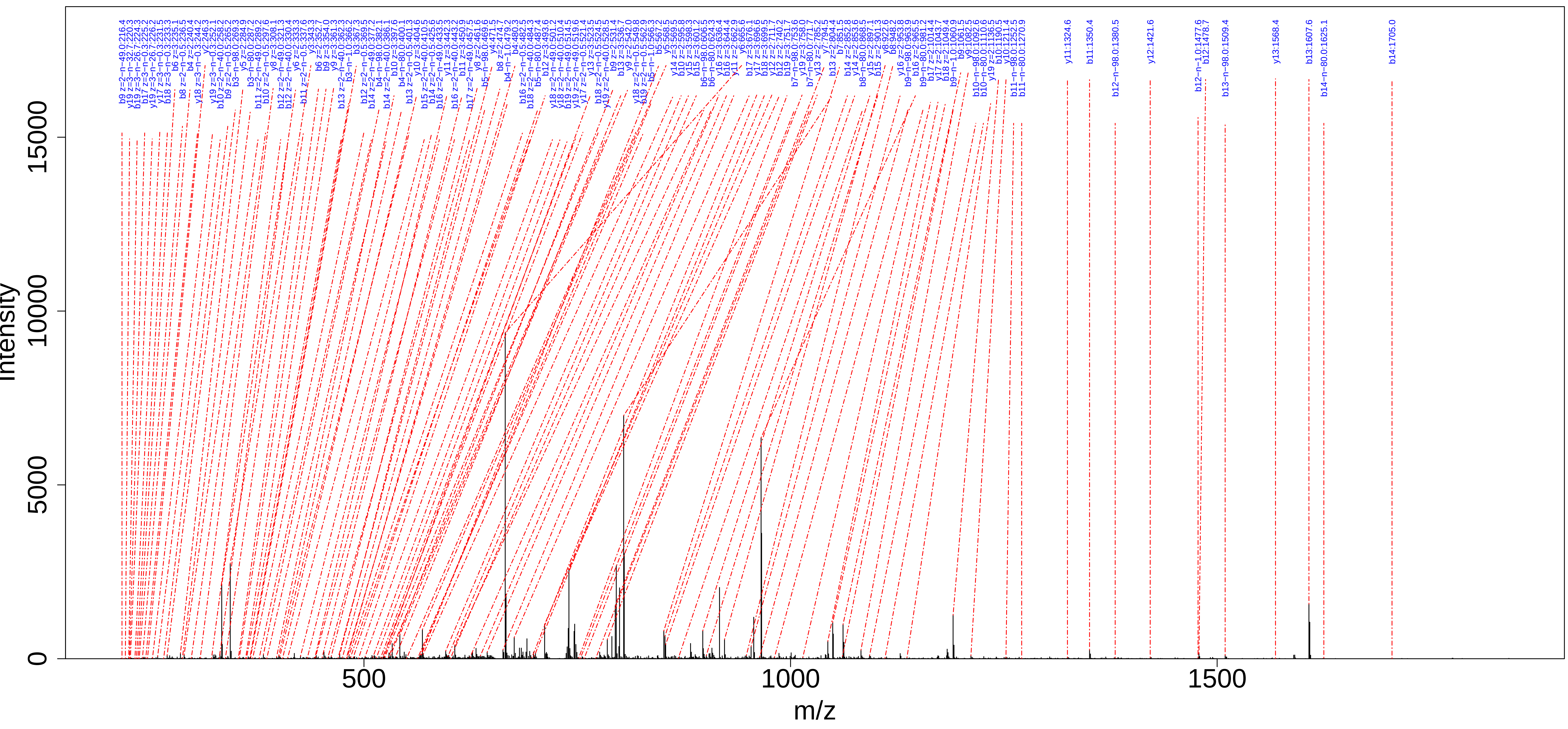

ANTLSHFPIECQEPPQPAR 3−T_Phospho z=+3 Raw scan=3000
TBCD1_HUMAN T596; ; Q= 0 ; score= 64.3 ; E= 8.3e−06 m/z=758.0113

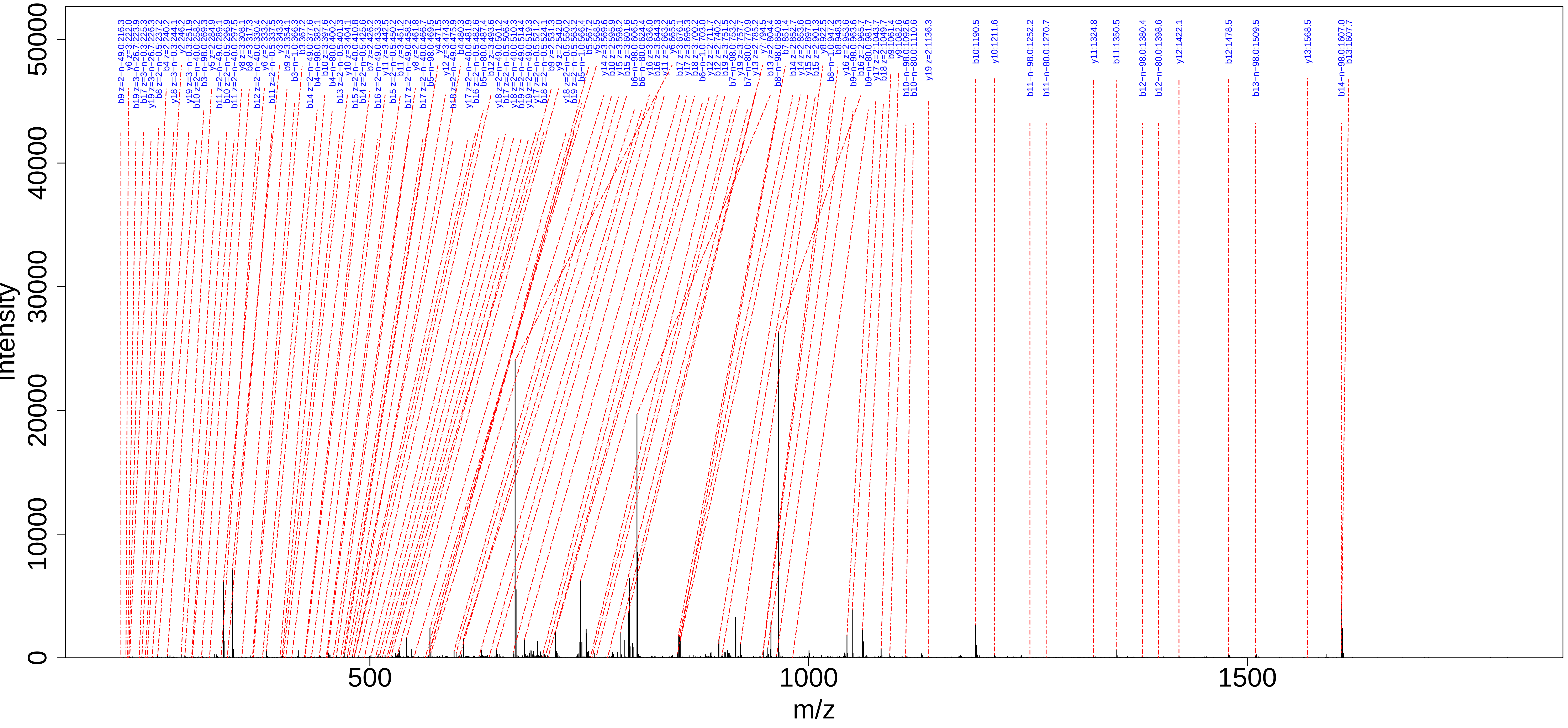

RANTLSHFPIECQEPPQPAR 4−T_Phospho z=+3 Raw scan=2675
TBCD1_HUMAN T596; ; Q= 0 ; score= 62.4 ; E= 1.9e−08 m/z=810.0463

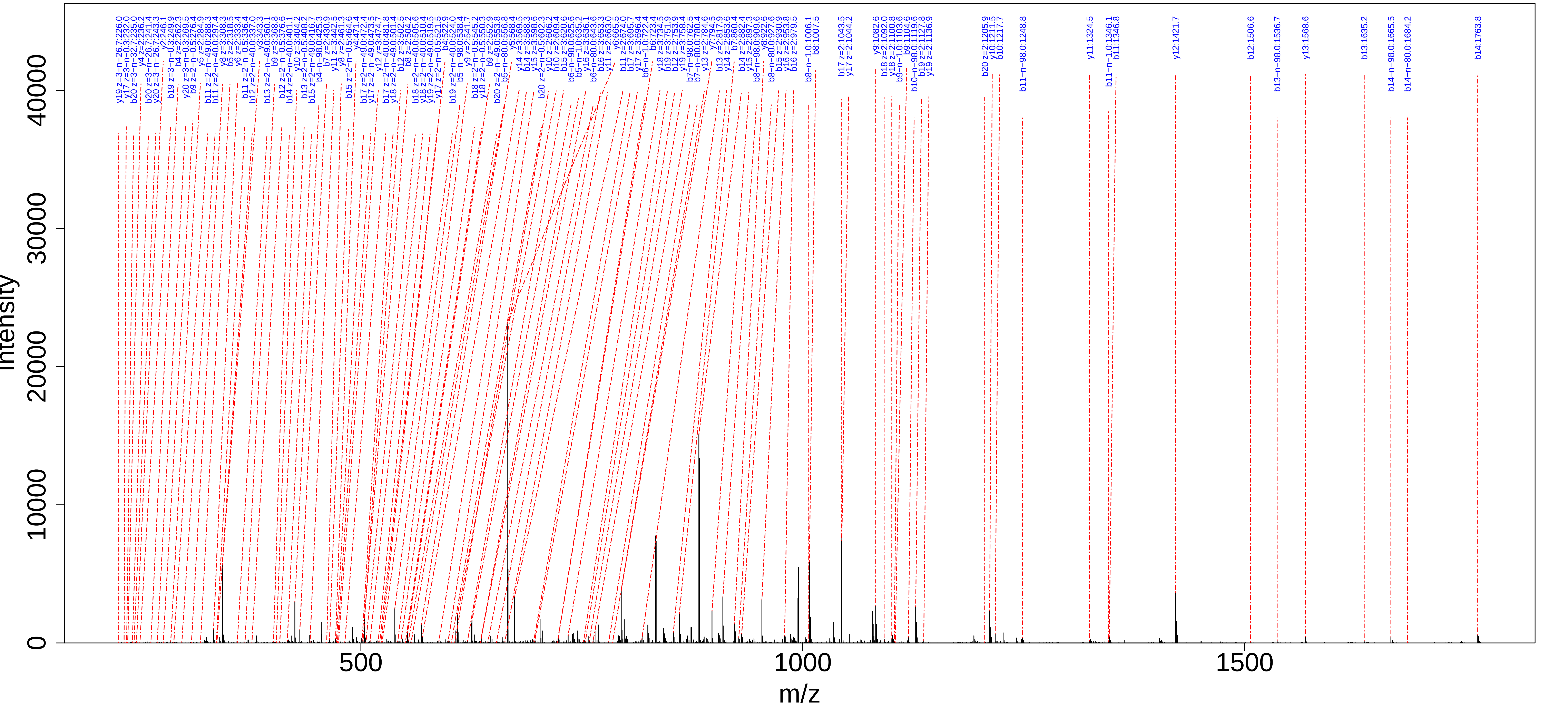

RANTLSHFPIECQEPPQPAR 4−T_Phospho z=+3 Raw scan=2693
TBCD1_HUMAN T596; ; Q= 0 ; score= 59.2 ; E= 6e−05 m/z=810.0469

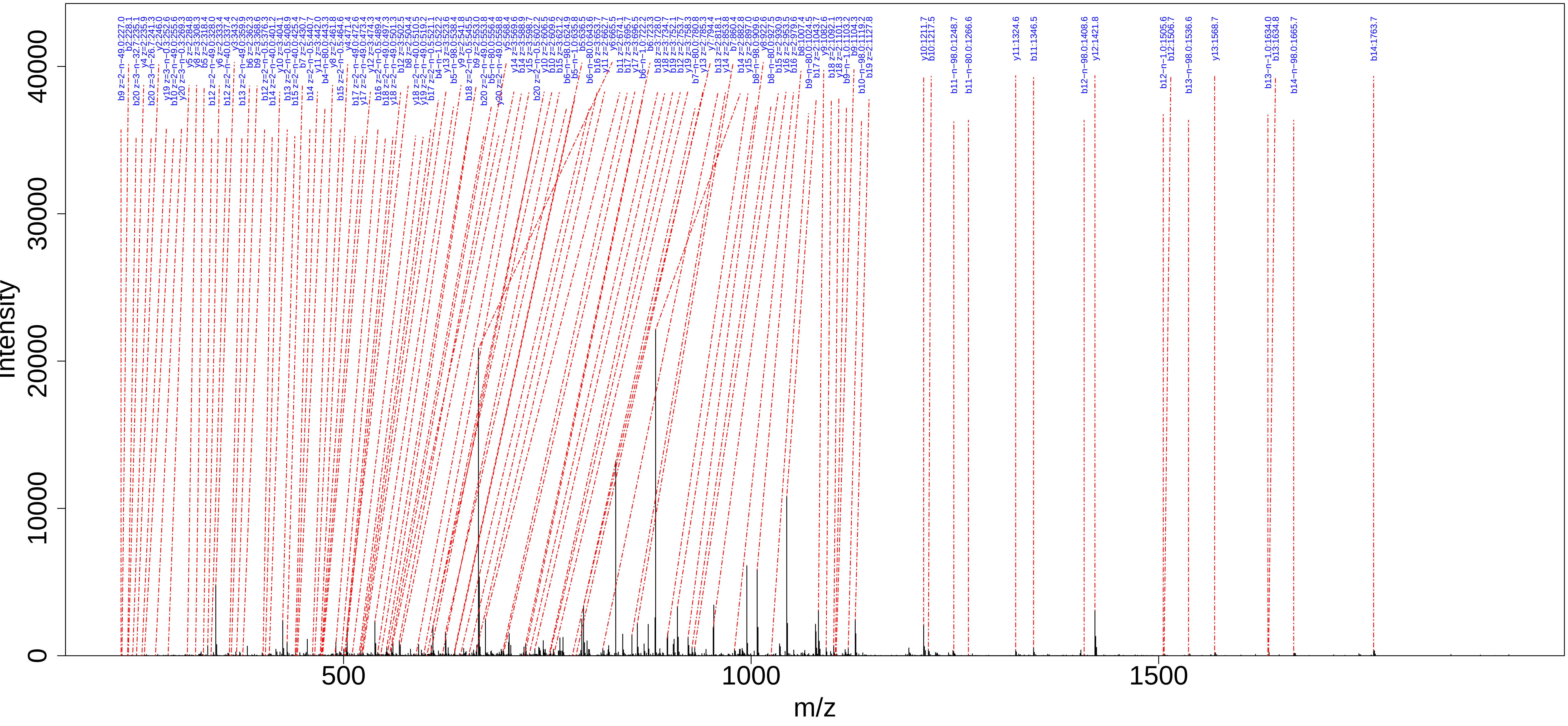

RANTLSHFPIECQEPPQPAR 6−S_Phospho z=+3 Raw scan=2693
TBCD1_HUMAN T596; ; Q= 0 ; score= 59.2 ; E= 6e−05 m/z=810.0469

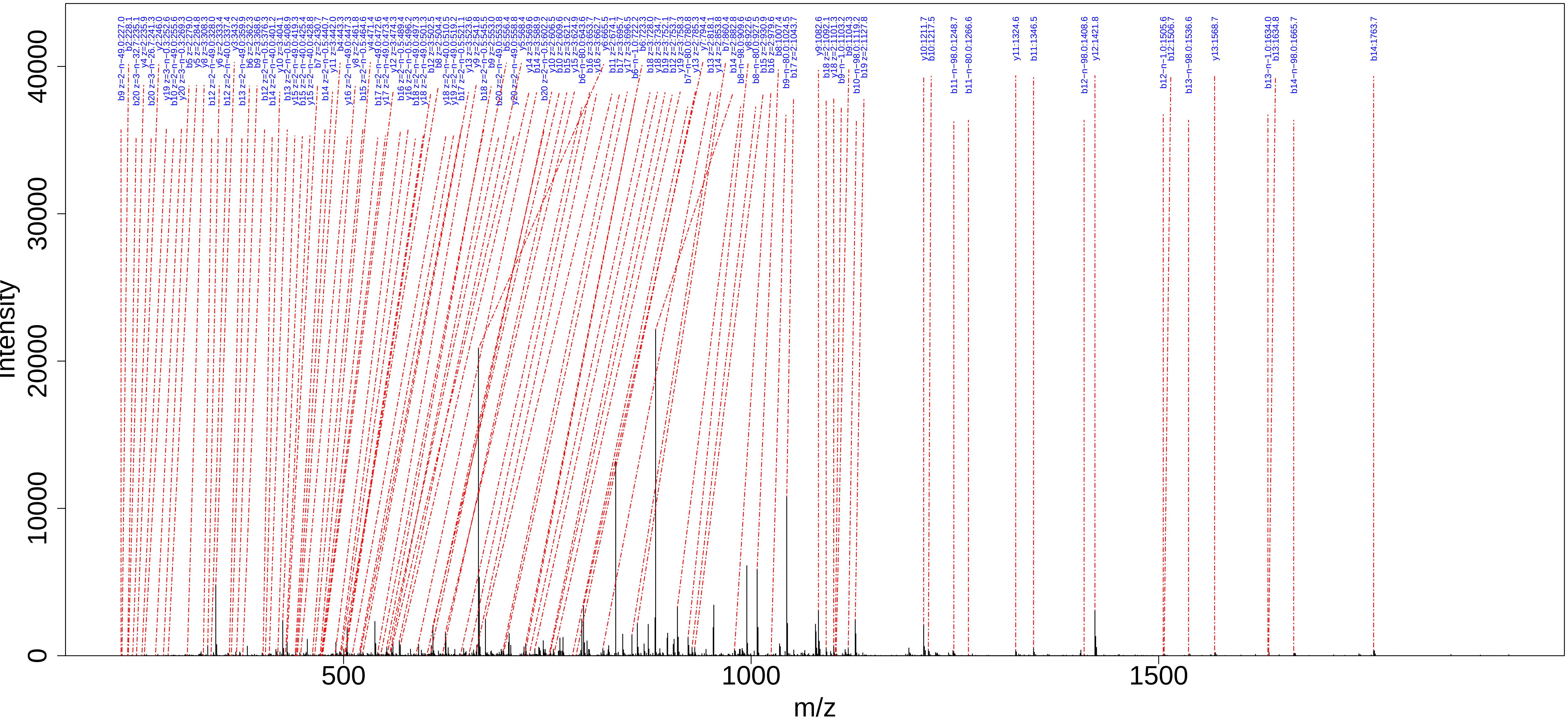

RANTLSHFPIECQEPPQPAR 4−T_Phospho z=+3 Raw scan=2671
TBCD1_HUMAN S598; ; Q= 0 ; score= 59.1 ; E= 3.8e−06 m/z=810.0461

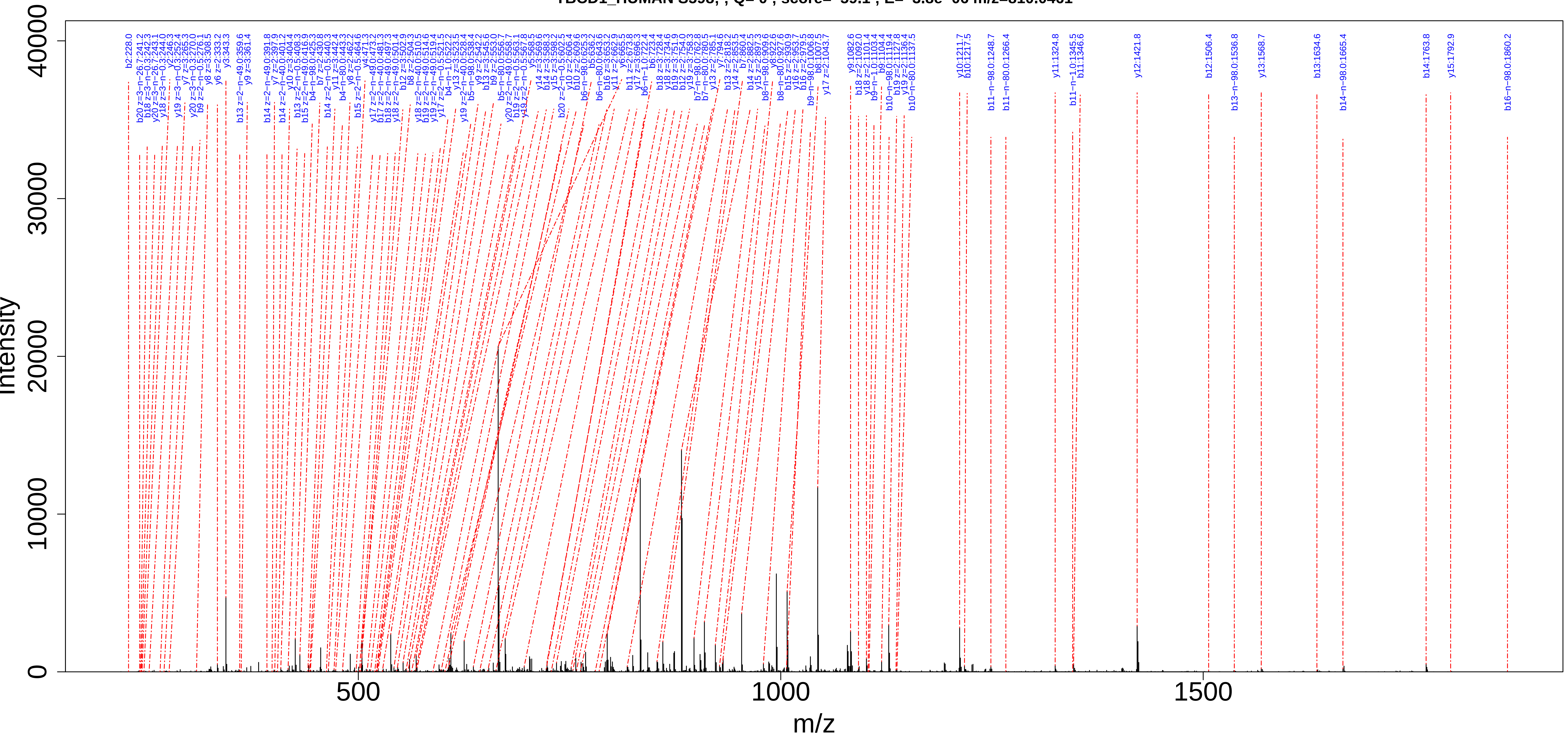

ANTLSHFPIECQEPPQPAR 3−T_Phospho z=+3 Raw scan=2992
TBCD1_HUMAN T596; ; Q= 0 ; score= 58.1 ; E= 6.1e−05 m/z=758.0126

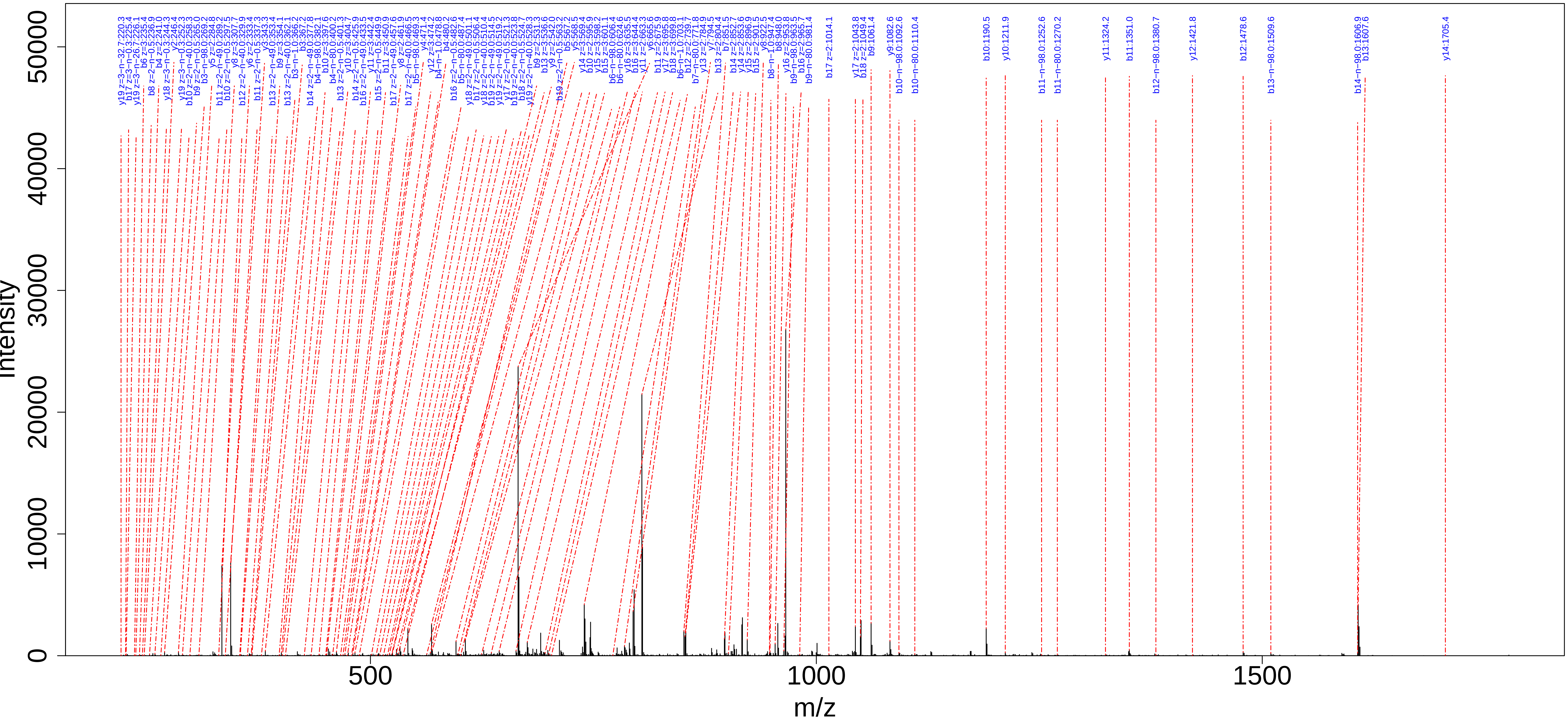

RANTLSHFPIECQEPPQPAR 4−T_Phospho z=+3 Raw scan=2666
TBCD1_HUMAN T596; ; Q= 0 ; score= 57.7 ; E= 5.3e−05 m/z=810.0444

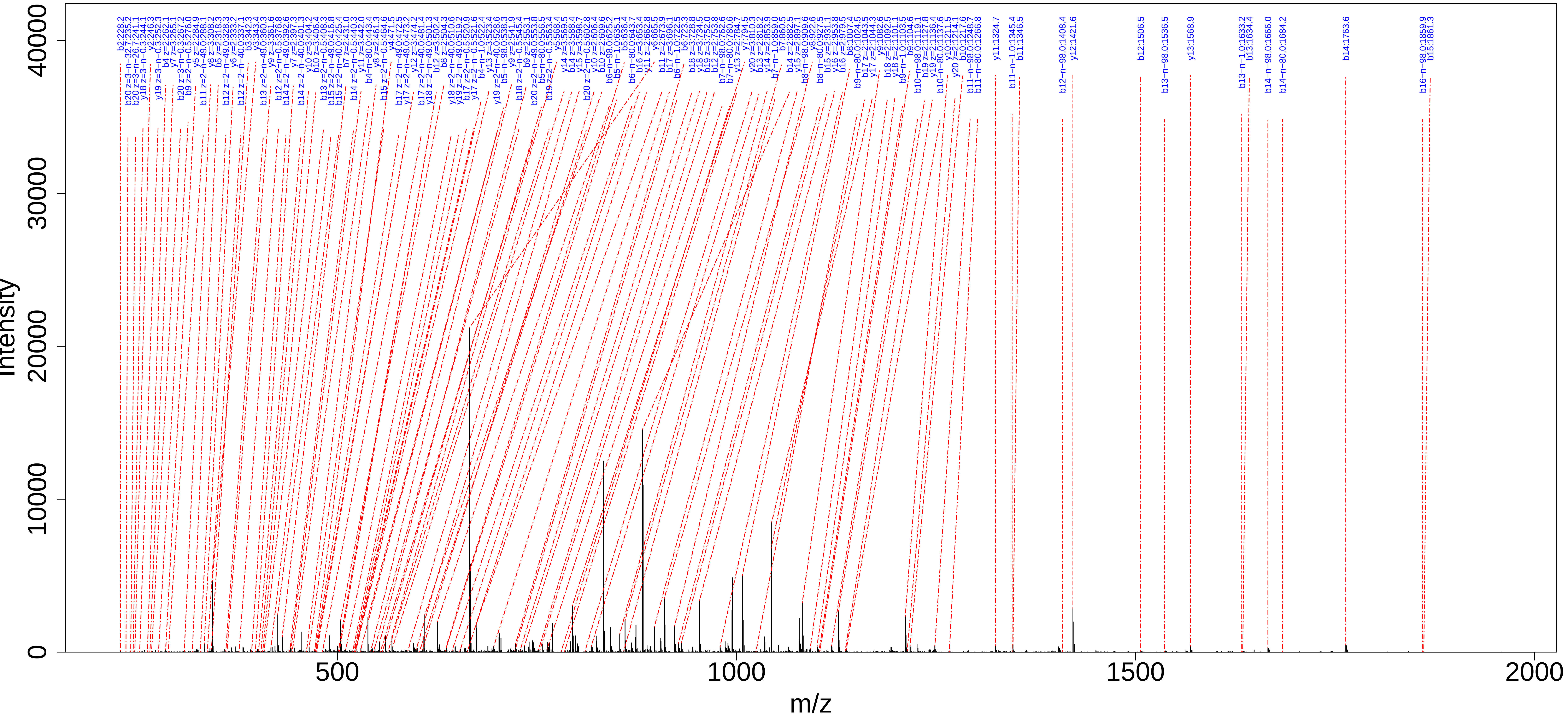

SLTESLESILSR 1−S_Phospho z=+2 Raw scan=4771
TBCD1_HUMAN T505; ; Q= 0 ; score= 53.1 ; E= 2.8e−05 m/z=707.8455

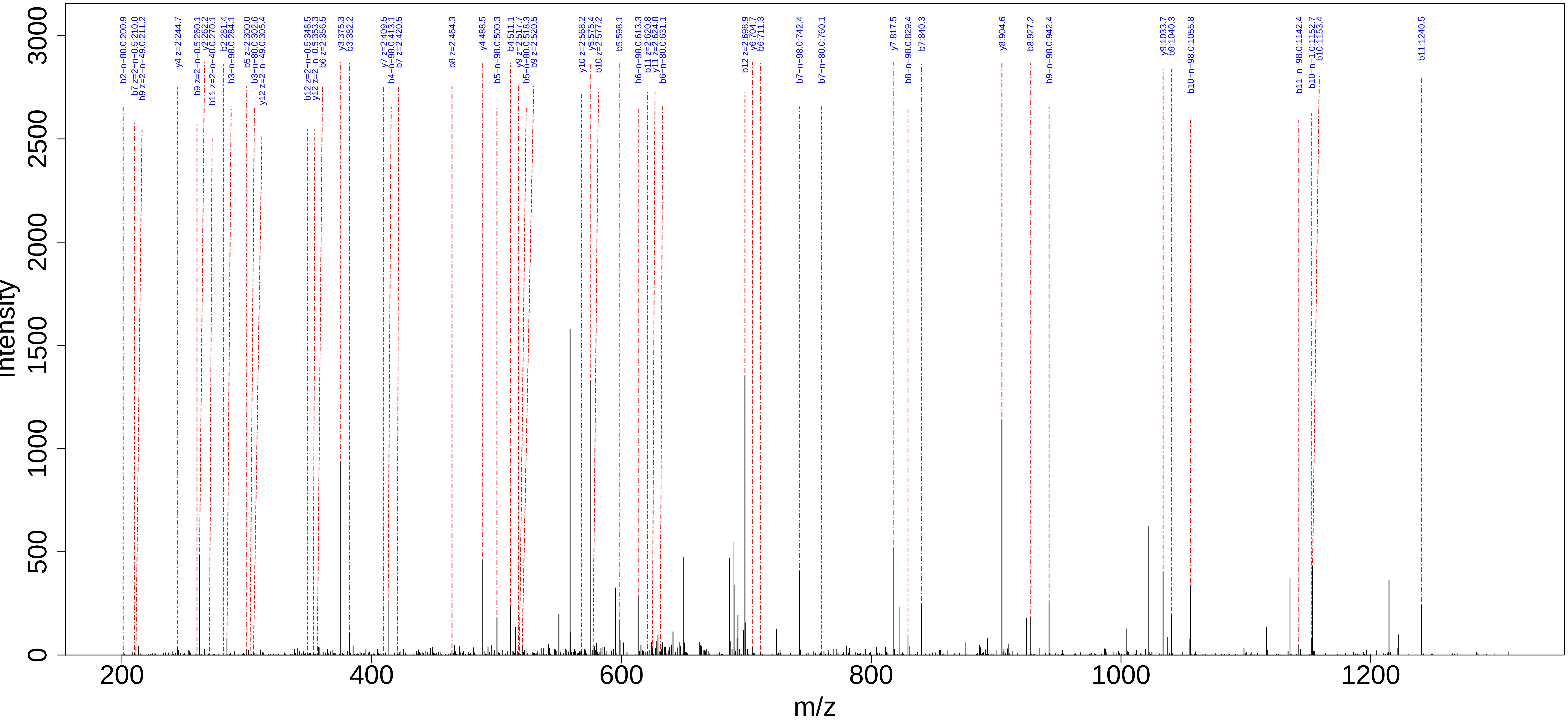

SLTESLESILSR 3−T_Phospho z=+2 Raw scan=4783
TBCD1_HUMAN S513; ; Q= 0 ; score= 53 ; E= 1.2e−05 m/z=707.845

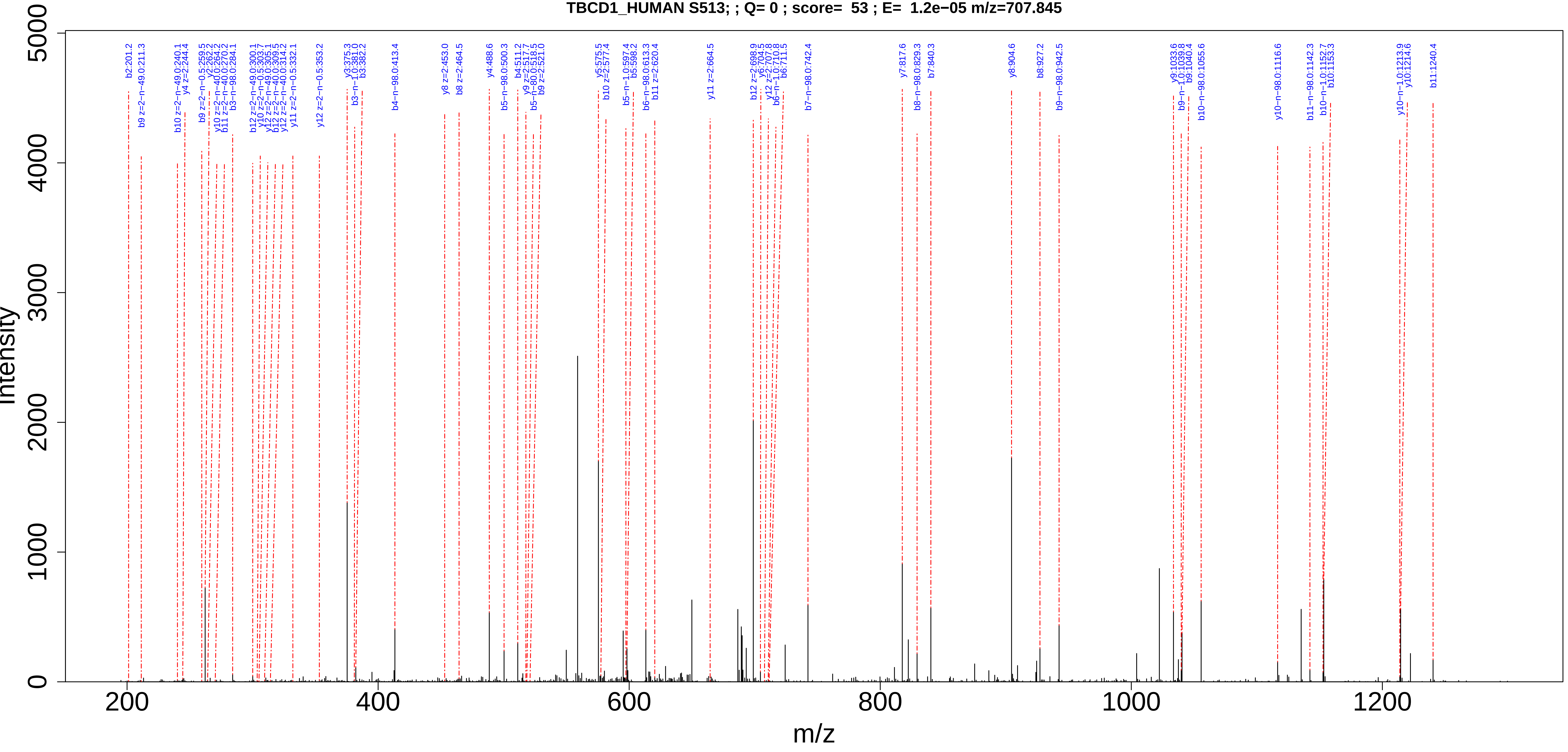

SLTESLESILSR 3−T_Phospho z=+2 Raw scan=4777
TBCD1_HUMAN T505; ; Q= 0 ; score= 53 ; E= 9.2e−07 m/z=707.8452

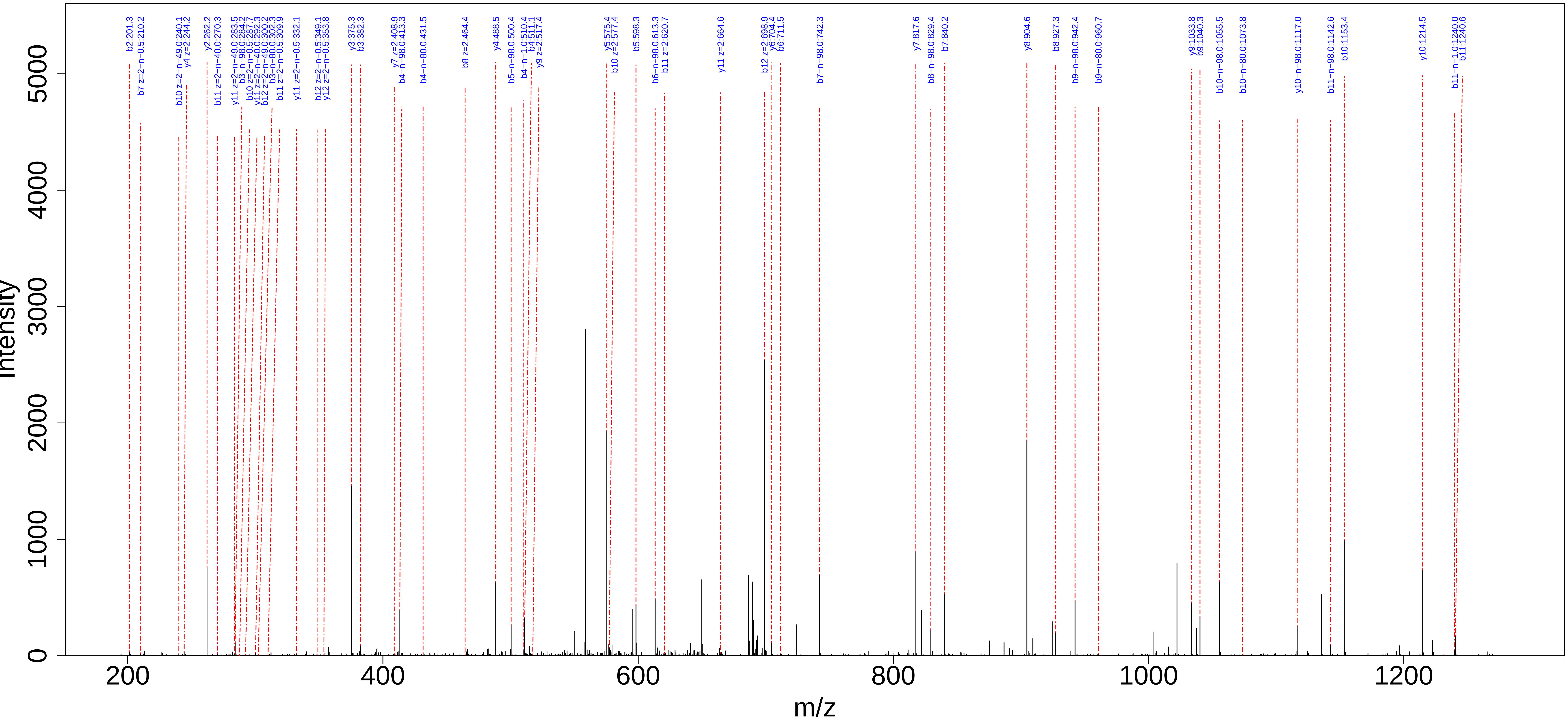

SLTESLESILSR 1−S_Phospho z=+2 Raw scan=4798
TBCD1_HUMAN S507; ; Q= 0 ; score= 49.7 ; E= 6.1e−05 m/z=707.844

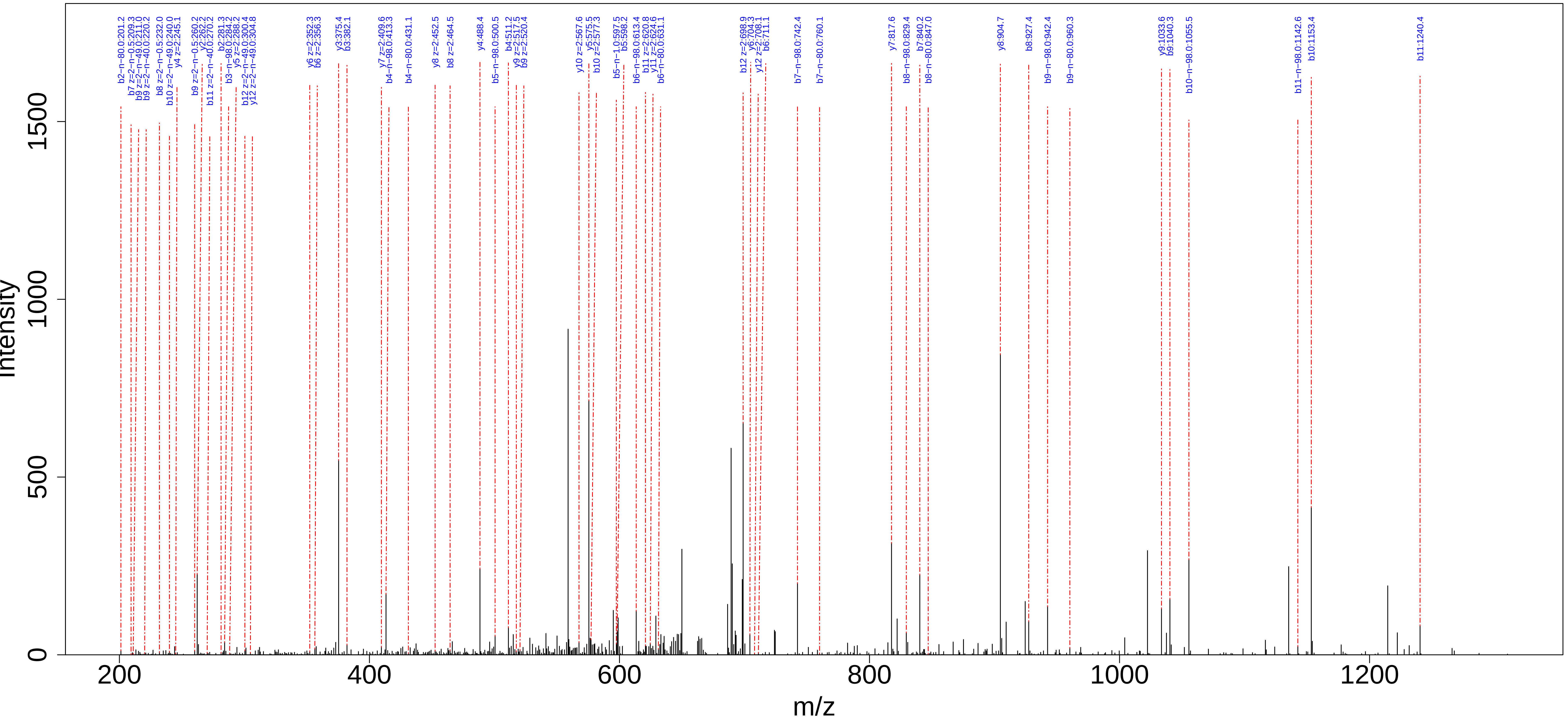

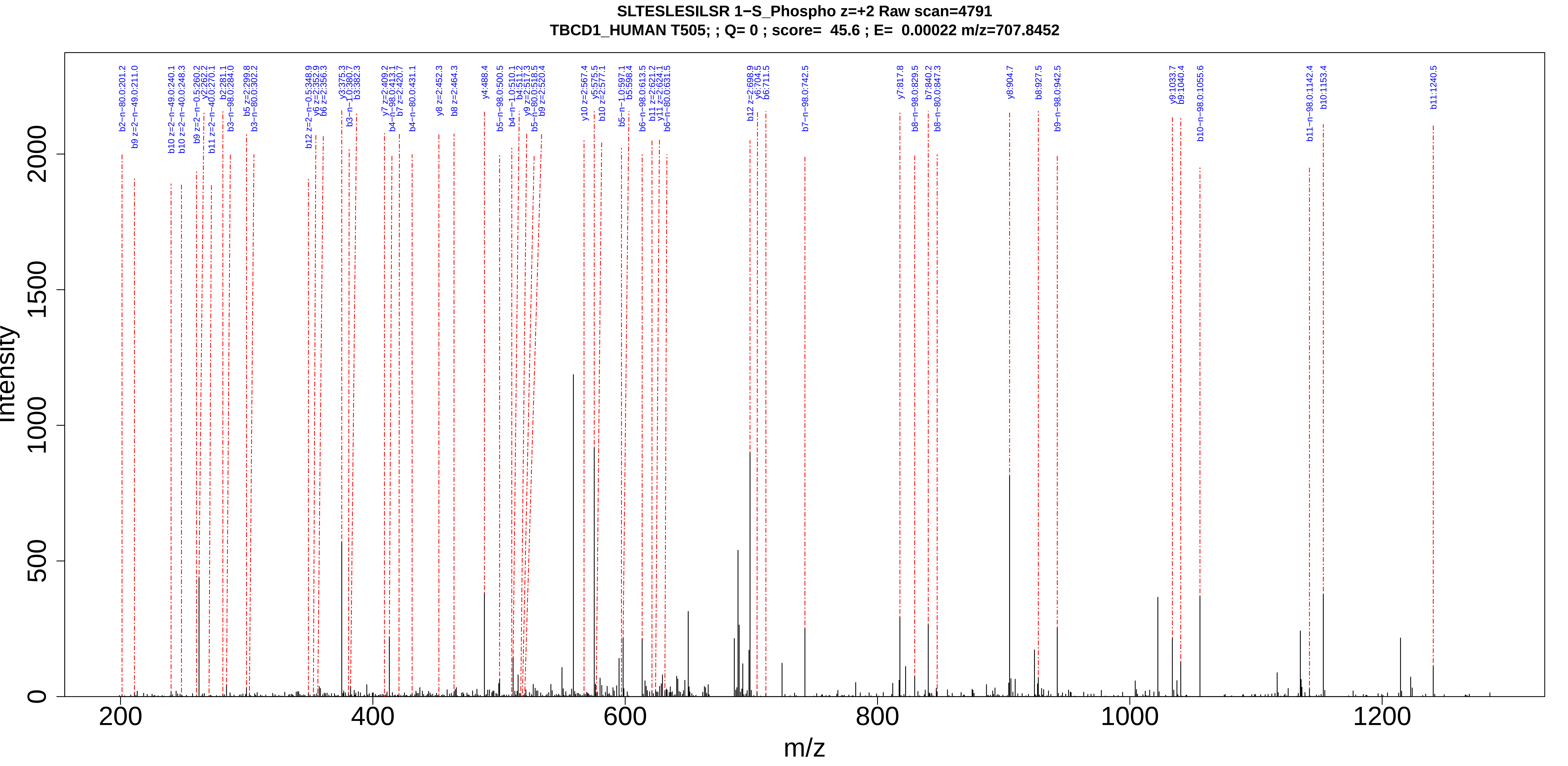
SLTESLESILSR 1−S_Phospho z=+2 Raw scan=4791
TBCD1_HUMAN T505; ; Q= 0 ; score= 45.6 ; E= 0.00022 m/z=707.8452
Intensity
m/z

SFSQPGLR 3−S_Phospho z=+2 Raw scan=2137
TBCD1_HUMAN S237; ; Q= 0 ; score= 44.7 ; E= 6e−05 m/z=486.2201

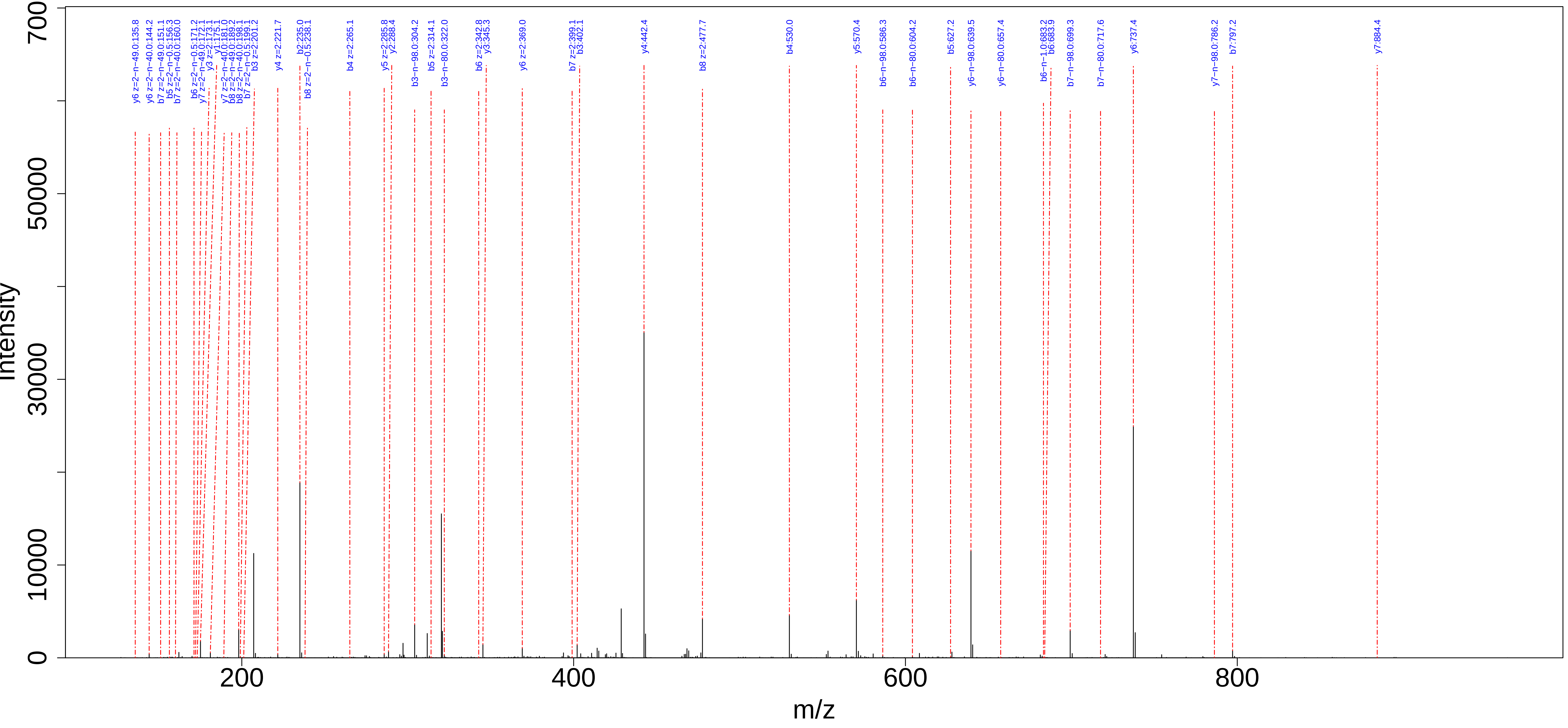

SFSQPGLR 3−S_Phospho z=+2 Raw scan=2143
TBCD1_HUMAN S237; ; Q= 0 ; score= 44.6 ; E= 6.4e−06 m/z=486.2201

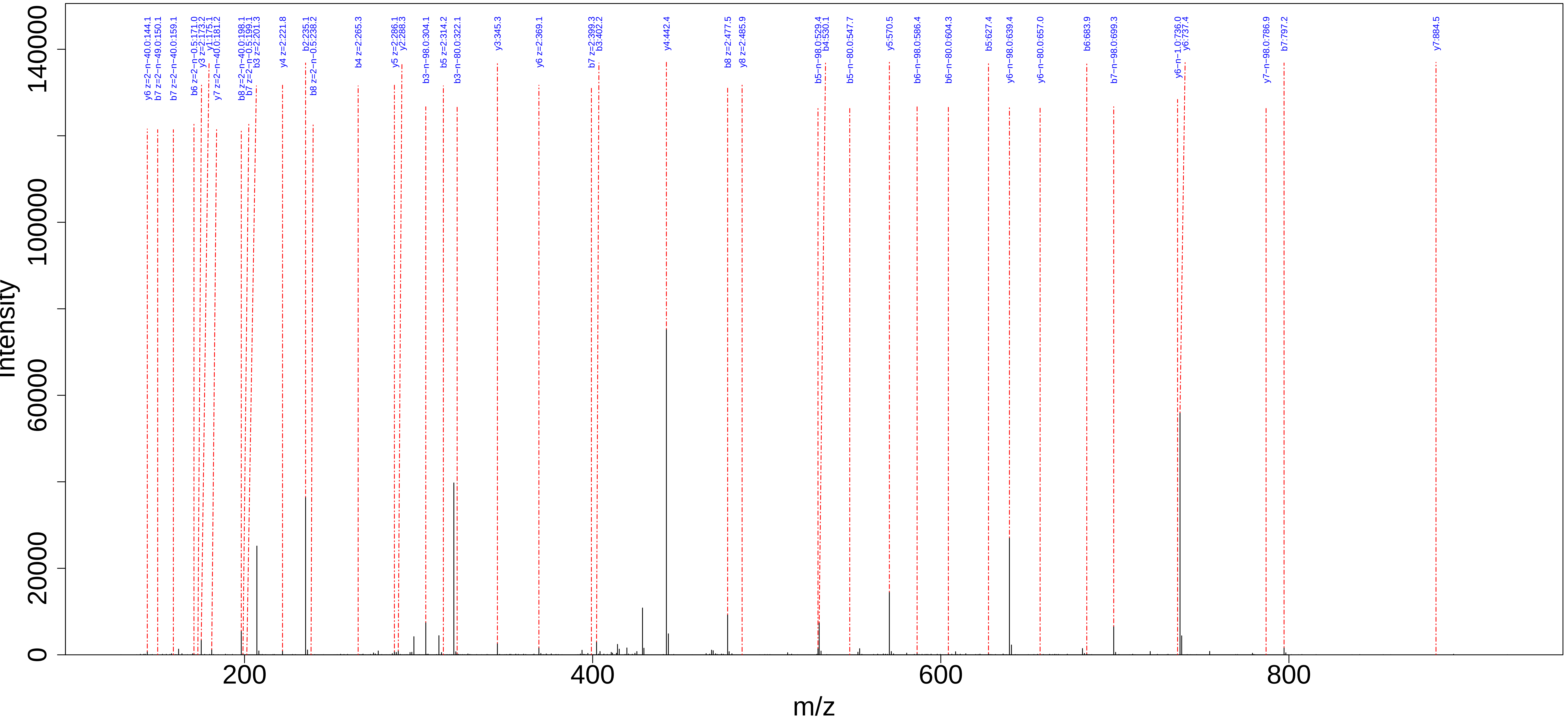

SFSQPGLR 3−S_Phospho z=+2 Raw scan=2149
TBCD1_HUMAN S237; ; Q= 0 ; score= 44.6 ; E= 2.9e−05 m/z=486.2202

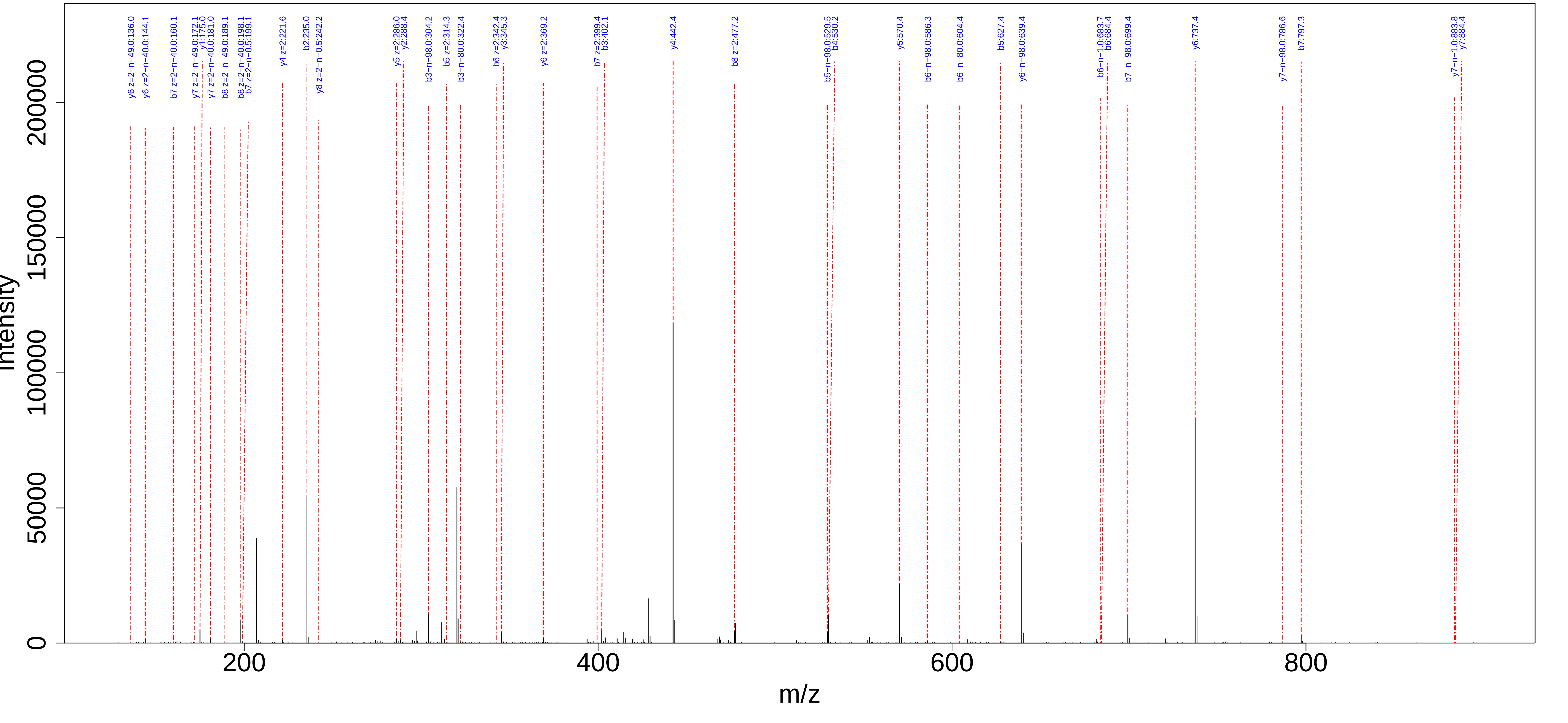

SFSQPGLR 3−S_Phospho z=+2 Raw scan=2184
TBCD1_HUMAN S237; ; Q= 0 ; score= 40.6 ; E= 0.00062 m/z=486.2197

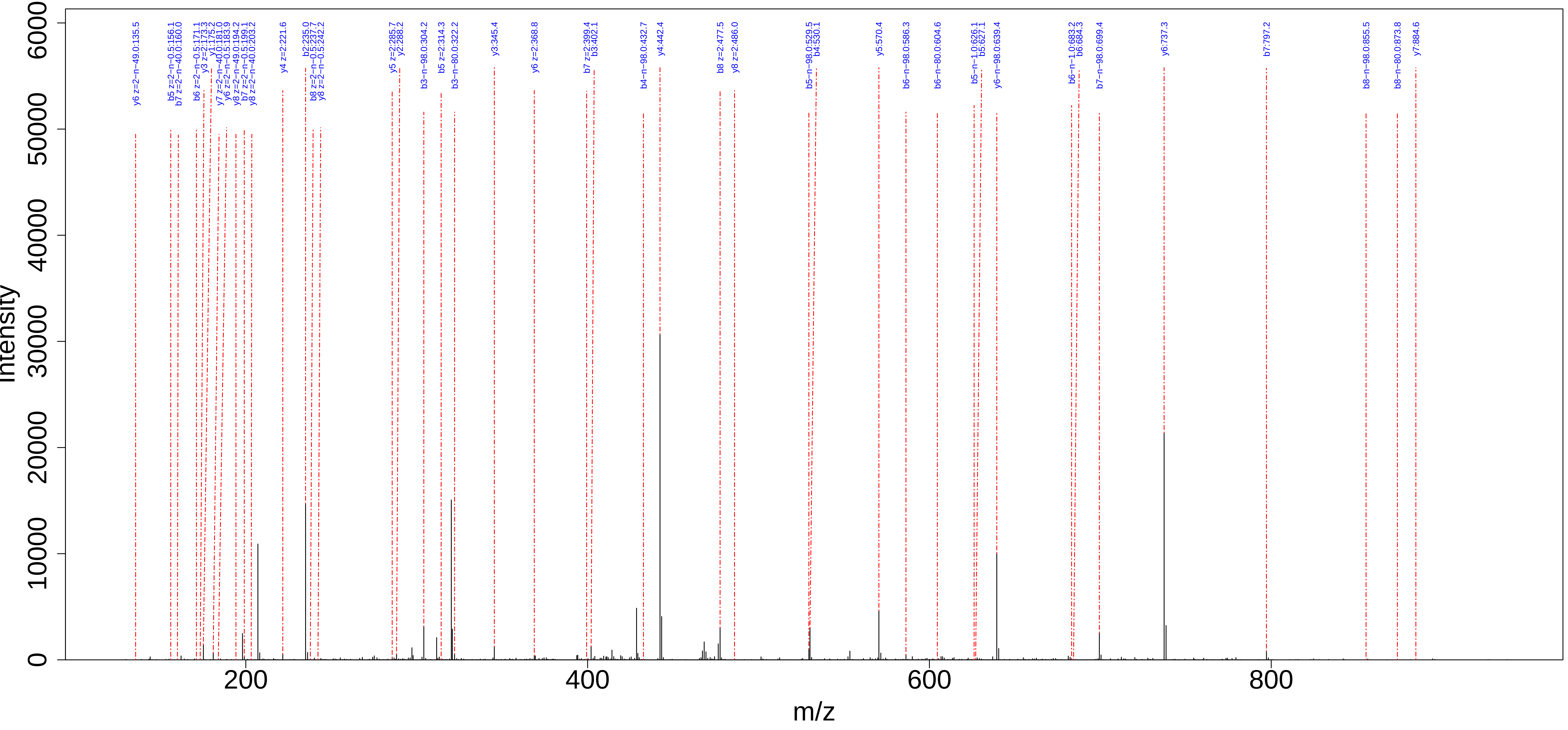

SFSQPGLR 3−S_Phospho z=+2 Raw scan=2163
TBCD1_HUMAN S237; ; Q= 0 ; score= 40.6 ; E= 1e−04 m/z=486.2208

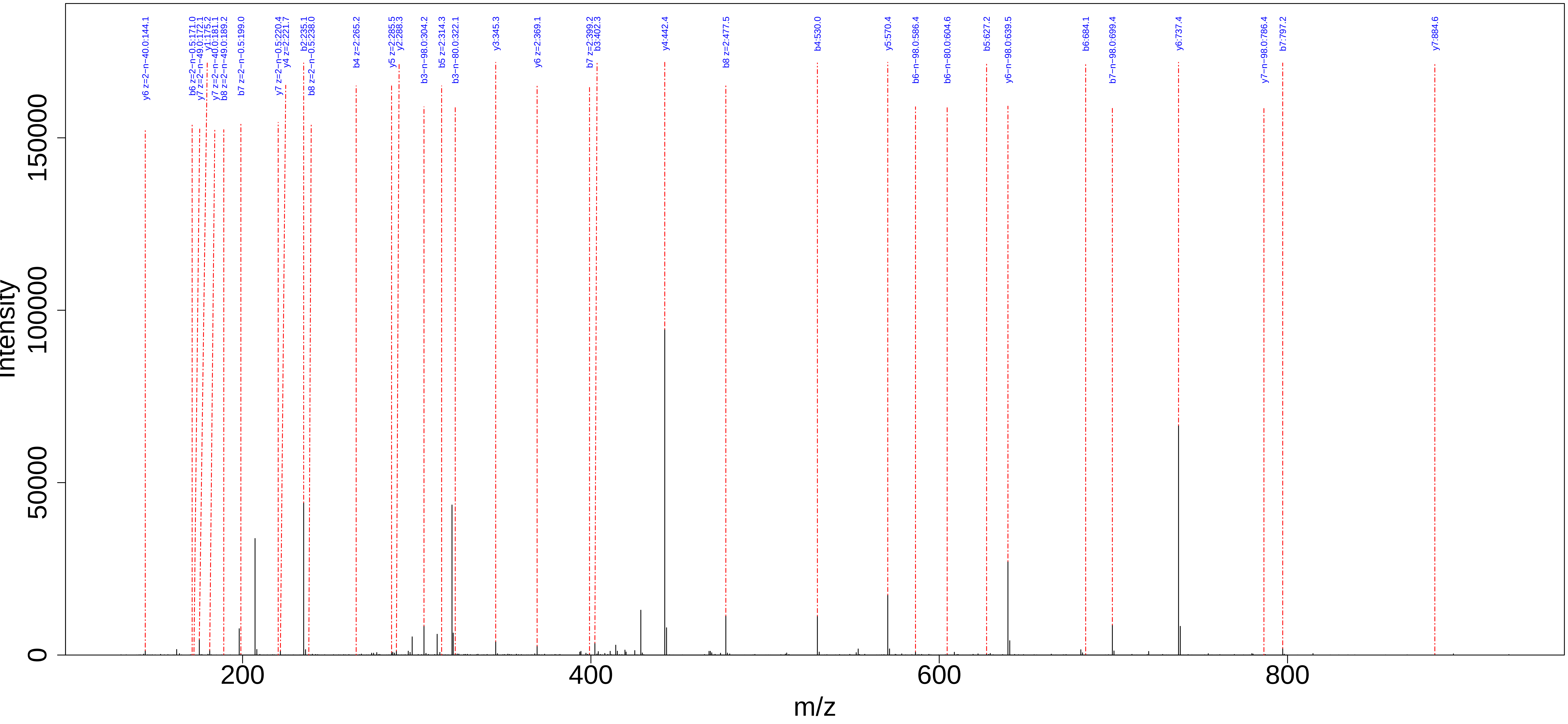

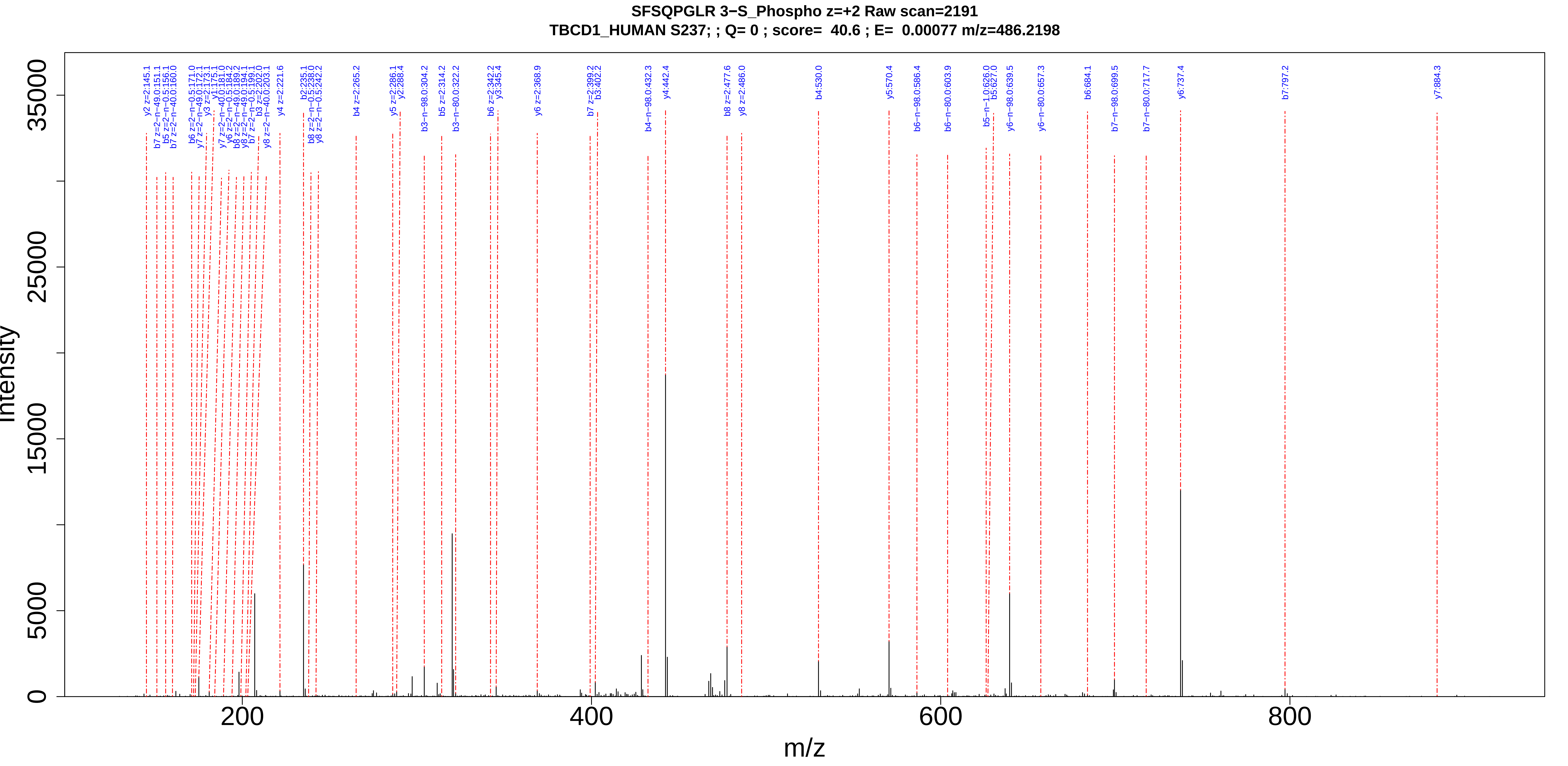

SFSQPGLR 3−S_Phospho z=+2 Raw scan=2191
TBCD1_HUMAN S237; ; Q= 0 ; score= 40.6 ; E= 0.00077 m/z=486.2198
Intensity
m/z

SFSQPGLR 3−S_Phospho z=+2 Raw scan=2177
TBCD1_HUMAN S235; ; Q= 0.00615006150061501 ; score= 36.8 ; E= 0.001 m/z=486.22

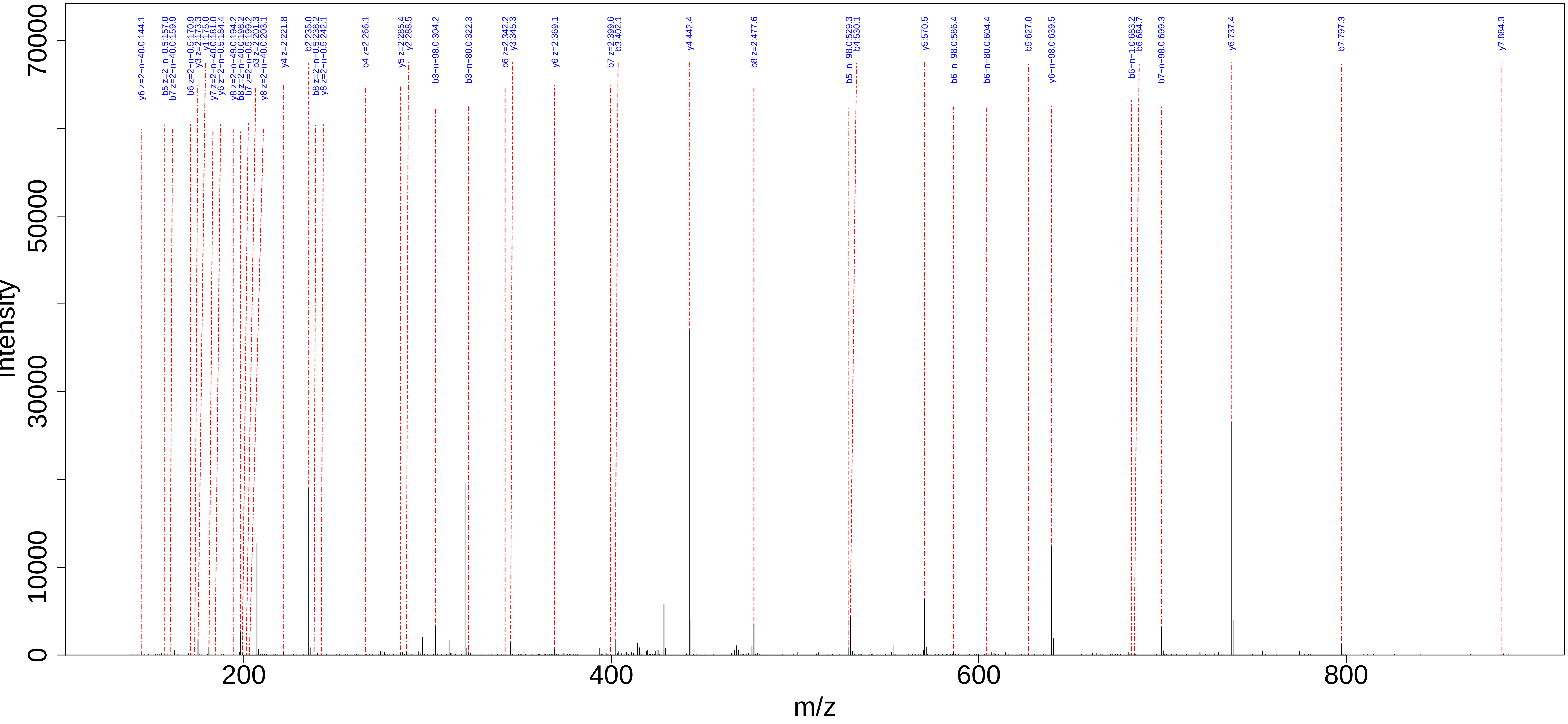

SFSQPGLR 3−S_Phospho z=+2 Raw scan=2156
TBCD1_HUMAN S237; ; Q= 0.00614817091915155 ; score= 36.8 ; E= 4e−04 m/z=486.2203

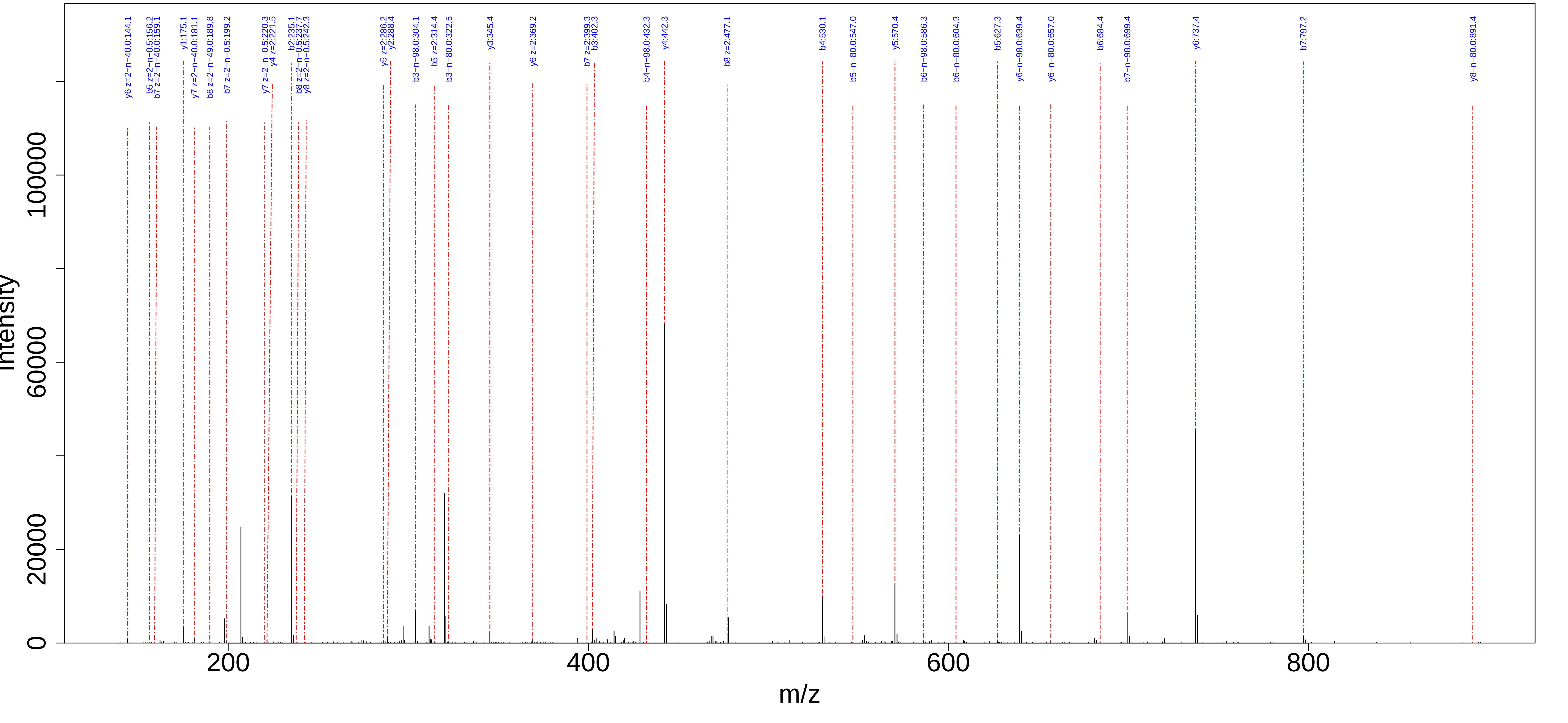

SFSQPGLR 3−S_Phospho z=+2 Raw scan=2170
TBCD1_HUMAN S237; ; Q= 0.00614628149969269 ; score= 36.8 ; E= 5e−04 m/z=486.2201

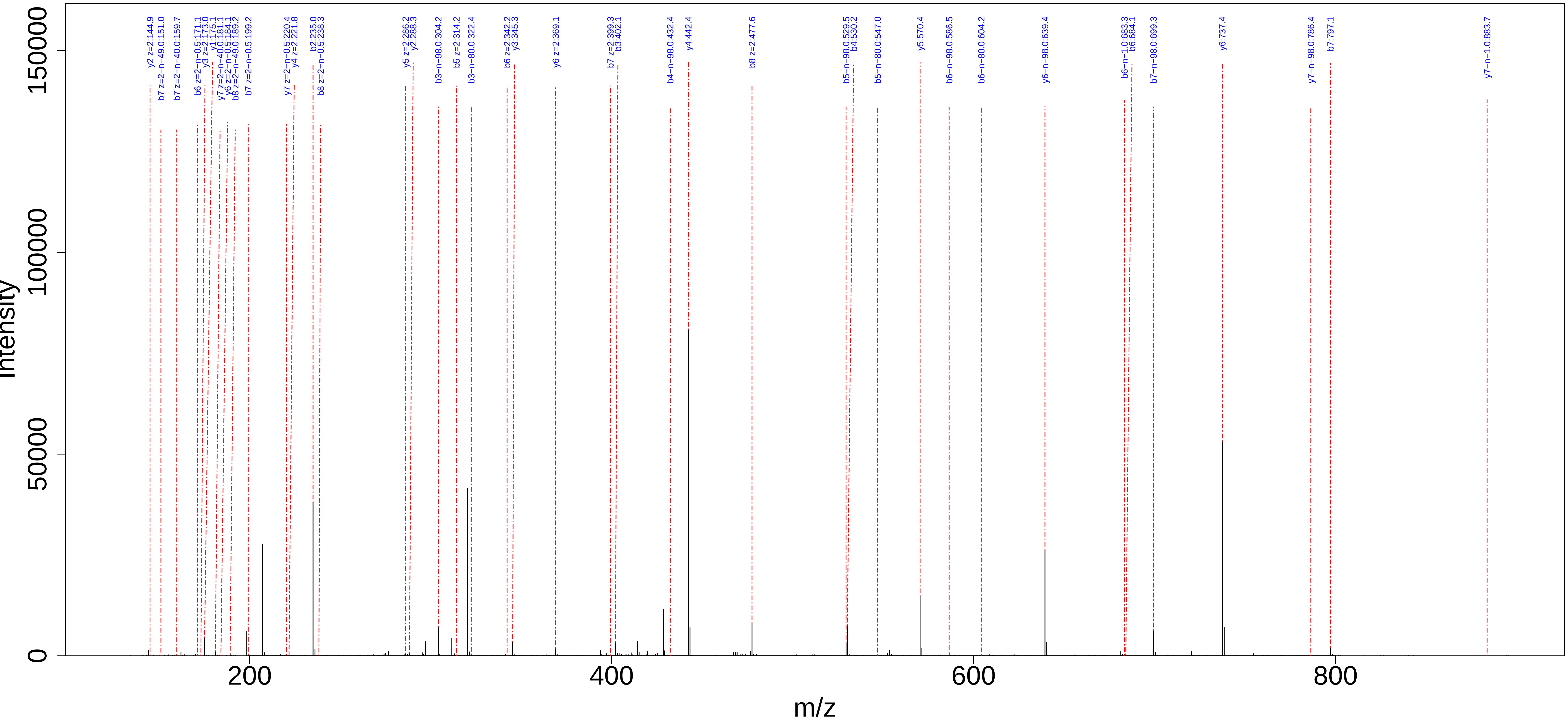

SFSQPGLR 3−S_Phospho z=+2 Raw scan=2198
TBCD1_HUMAN S237; ; Q= 0.00613873542050338 ; score= 36.7 ; E= 0.0013 m/z=486.2213

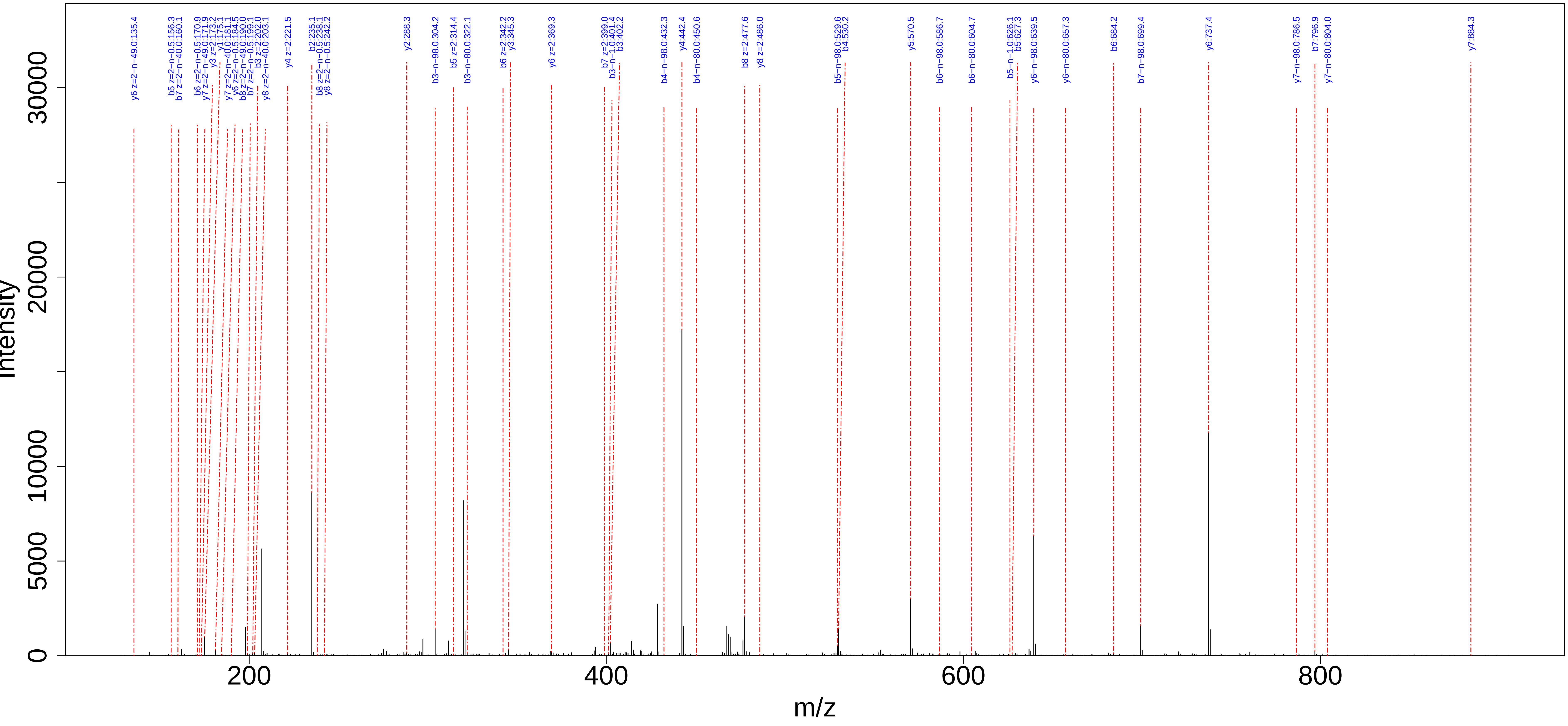

annotated raw MSMS spectra for assay:

Protein kinase: SGK1

Substrate: TBC1D4

sample name: TBCD4_SGK1_S6

TSSTCSNESLSVGGTSVTPR 1−T_Phospho z=+2 Raw scan=2386
TBCD4_HUMAN S764; ; Q= 0 ; score= 59.3 ; E= 6.3e−08 m/z=1053.9558

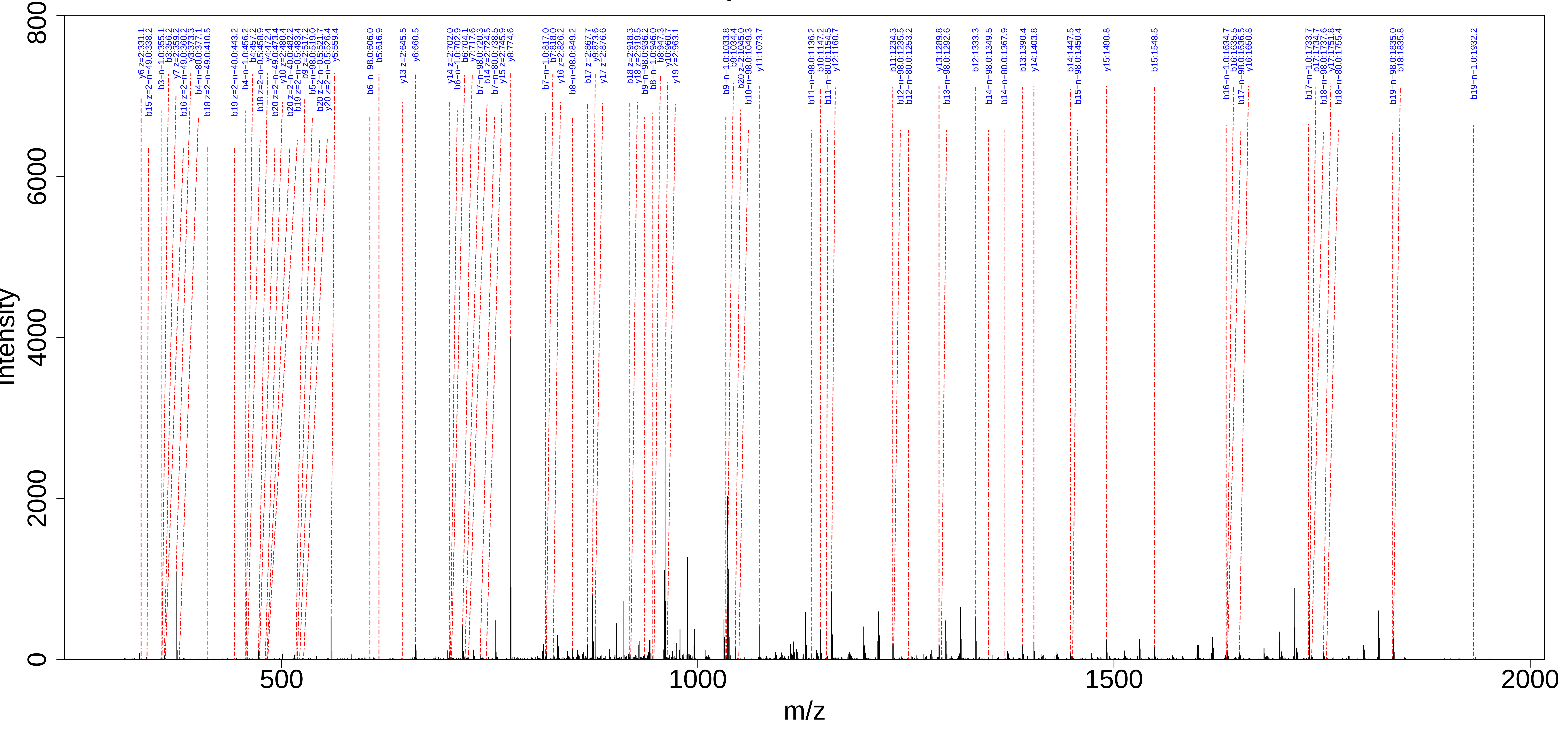

TSSTCSNESLSVGGTSVTPR 2−S_Phospho z=+2 Raw scan=2386
TBCD4_HUMAN S764; ; Q= 0 ; score= 59.3 ; E= 6.3e−08 m/z=1053.9558

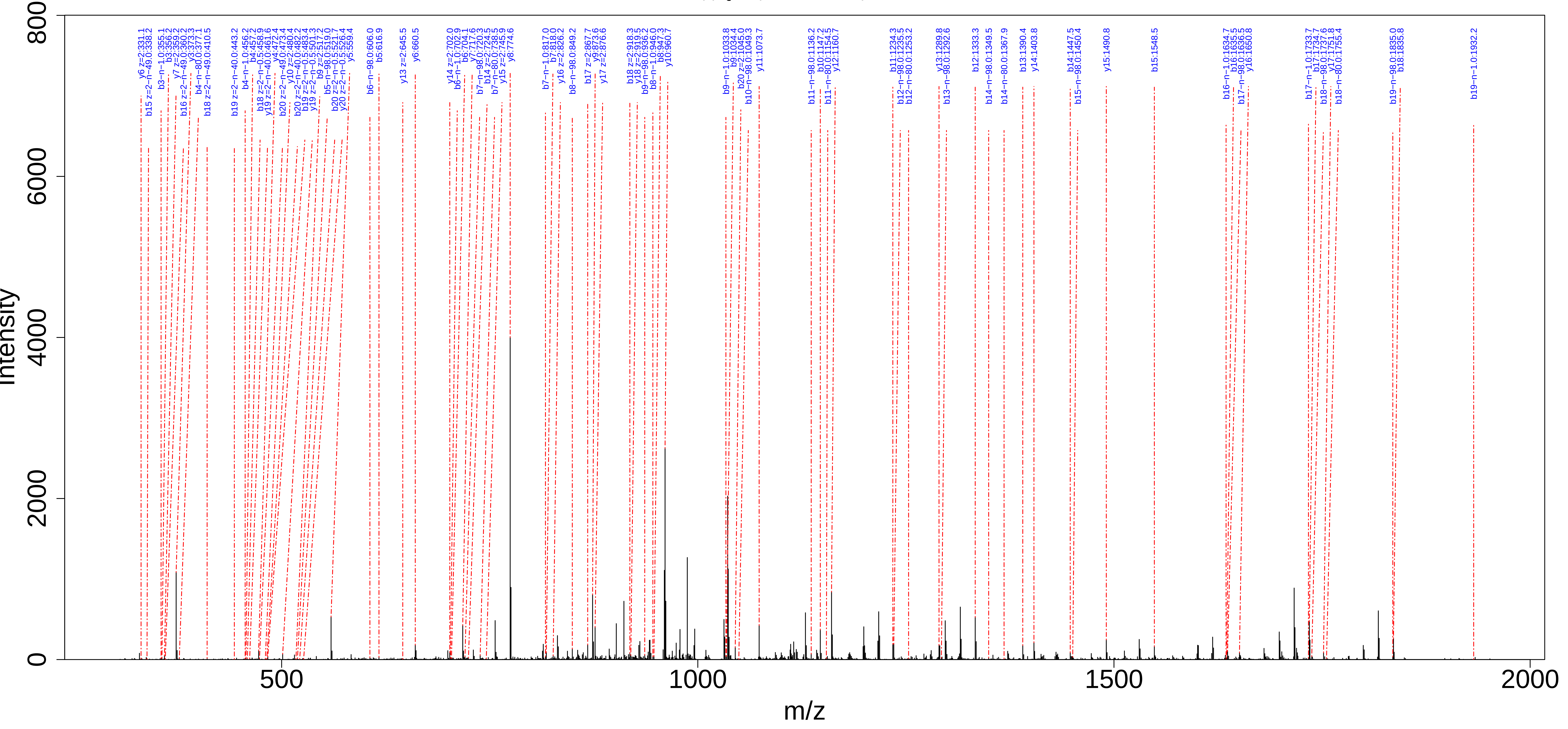

TSSTCSNESLSVGGTSVTPR 3−S_Phospho z=+2 Raw scan=2386
TBCD4_HUMAN S764; ; Q= 0 ; score= 59.3 ; E= 6.3e−08 m/z=1053.9558

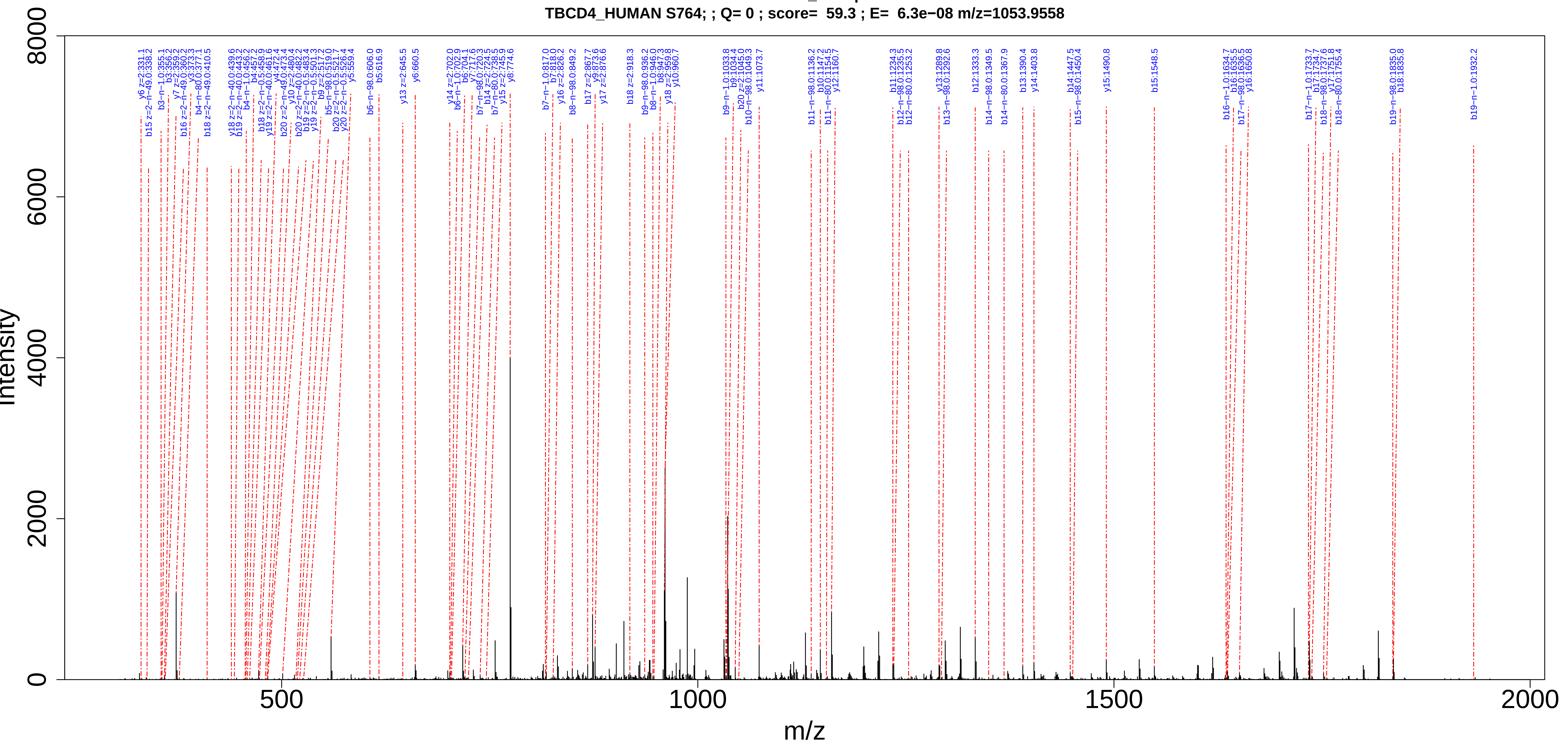

ESNSSSSLPSLHTSFSAPSFTAPSFLK 16−S_Phospho z=+3 Raw scan=4270
TBCD4_HUMAN S704; ; Q= 0 ; score= 57.8 ; E= 5.9e−05 m/z=965.1142

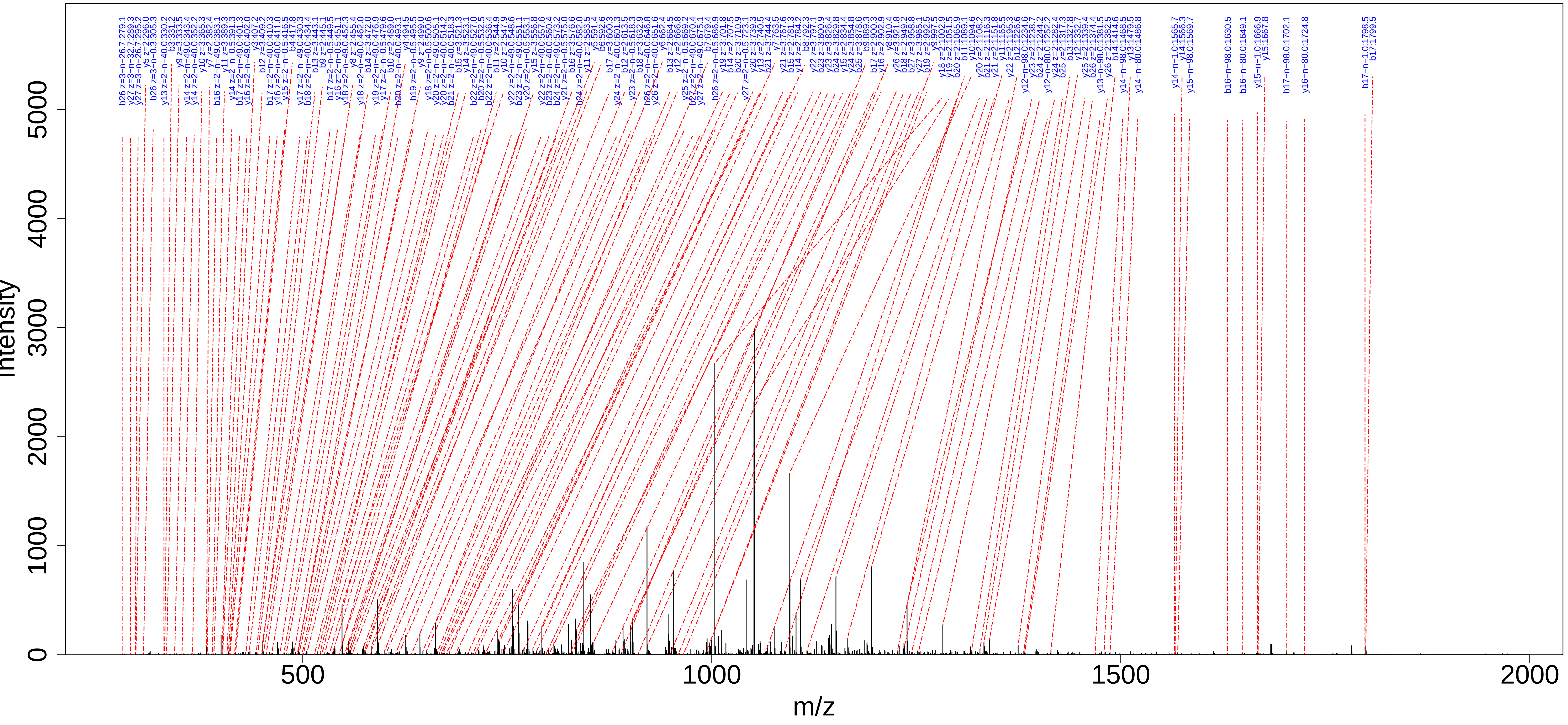

SLTSSLENIFSR 1−S_Phospho z=+2 Raw scan=4617
TBCD4_HUMAN S576; ; Q= 0 ; score= 57 ; E= 1.1e−06 m/z=717.3389

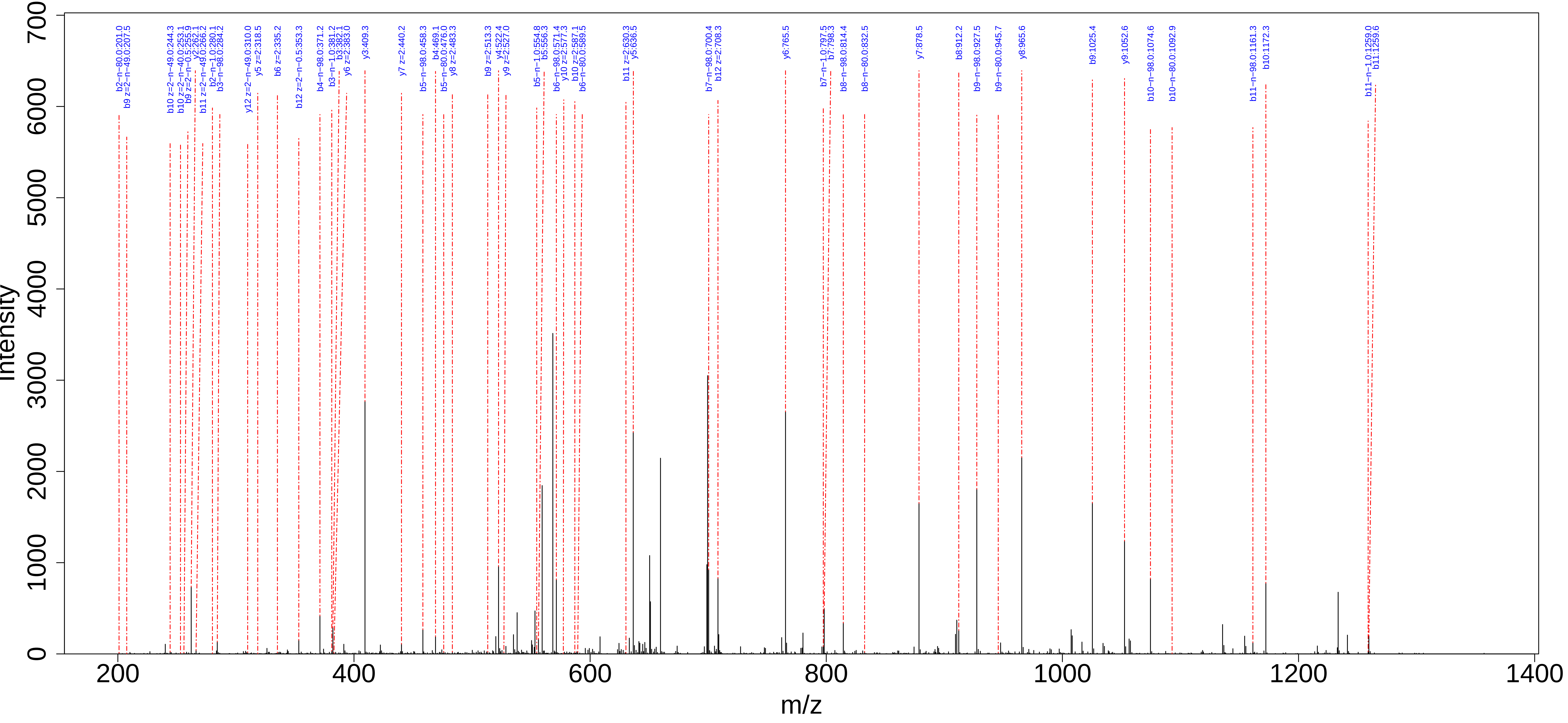

SLTSSLENIFSR 1−S_Phospho z=+2 Raw scan=4624
TBCD4_HUMAN S576; ; Q= 0 ; score= 56.7 ; E= 2.6e−06 m/z=717.3391

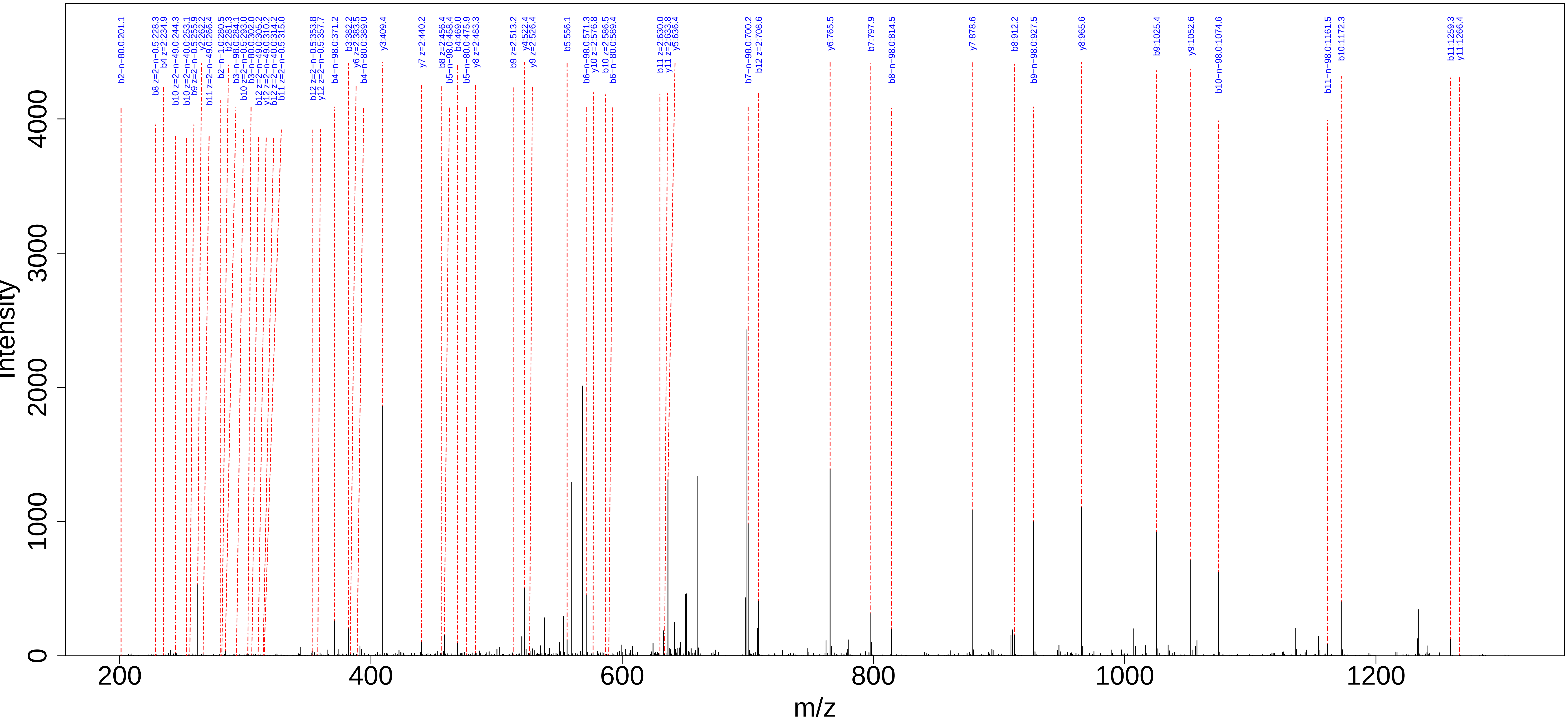

ESNSSSSLPSLHTSFSAPSFTAPSFLK 16−S_Phospho z=+3 Raw scan=4279
TBCD4_HUMAN S692; ; Q= 0 ; score= 55.9 ; E= 2.5e−05 m/z=965.1147

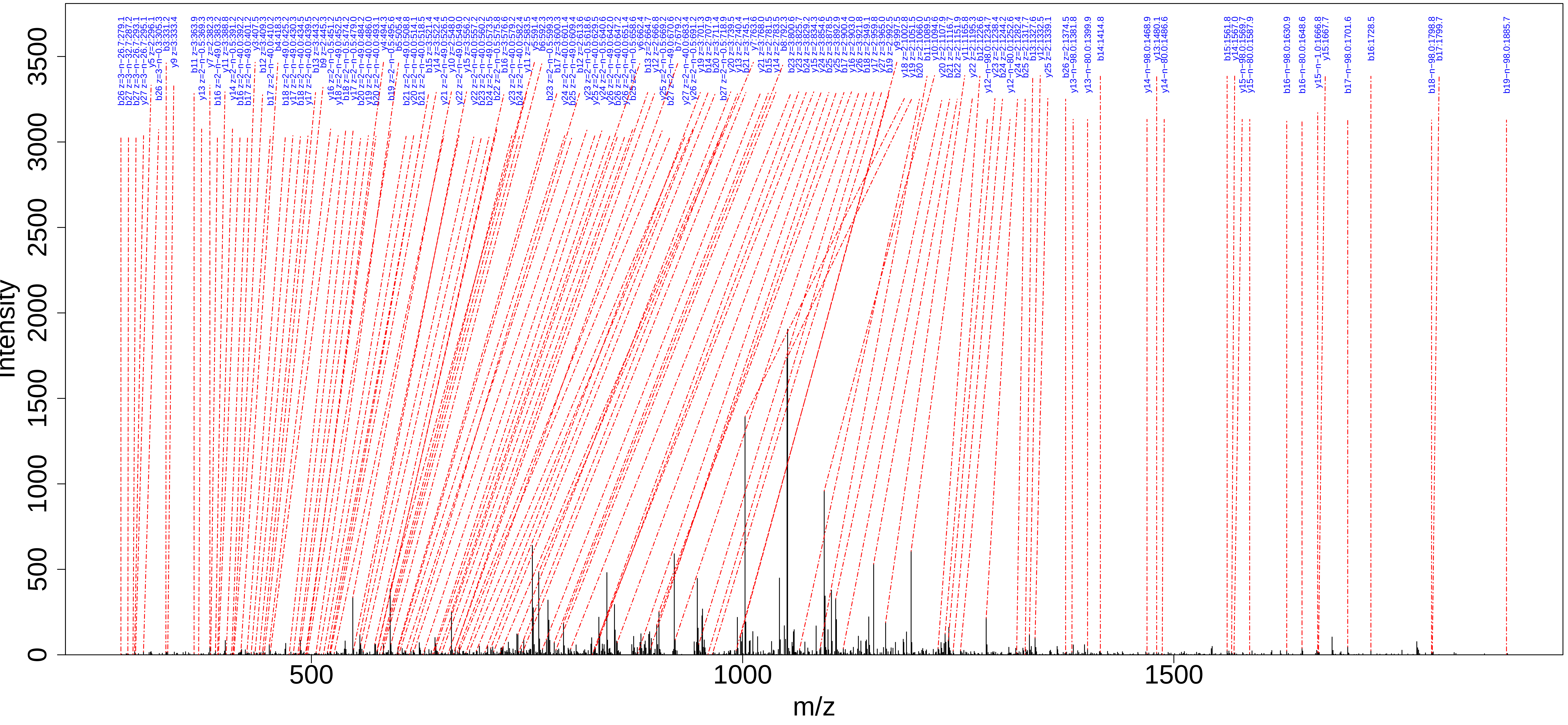

ESNSSSSLPSLHTSFSAPSFTAPSFLK 16−S_Phospho z=+3 Raw scan=4262
TBCD4_HUMAN S712; ; Q= 0 ; score= 55.8 ; E= 1.8e−05 m/z=965.1179

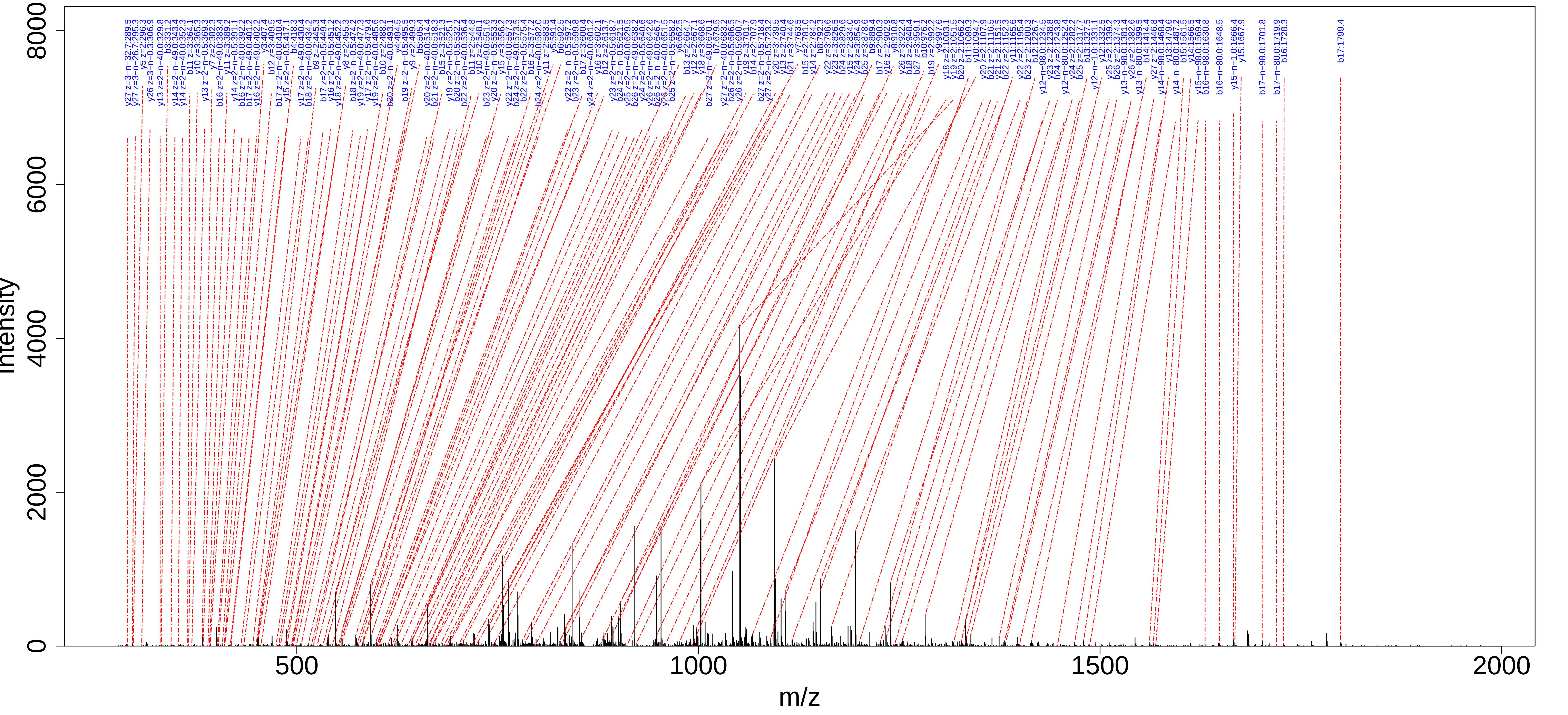

ESNSSSSLPSLHTSFSAPSFTAPSFLK 16−S_Phospho z=+3 Raw scan=4247

TBCD4_HUMAN S704; ; Q= 0 ; score= 55.8 ; E= 6.8e−05 m/z=965.1174

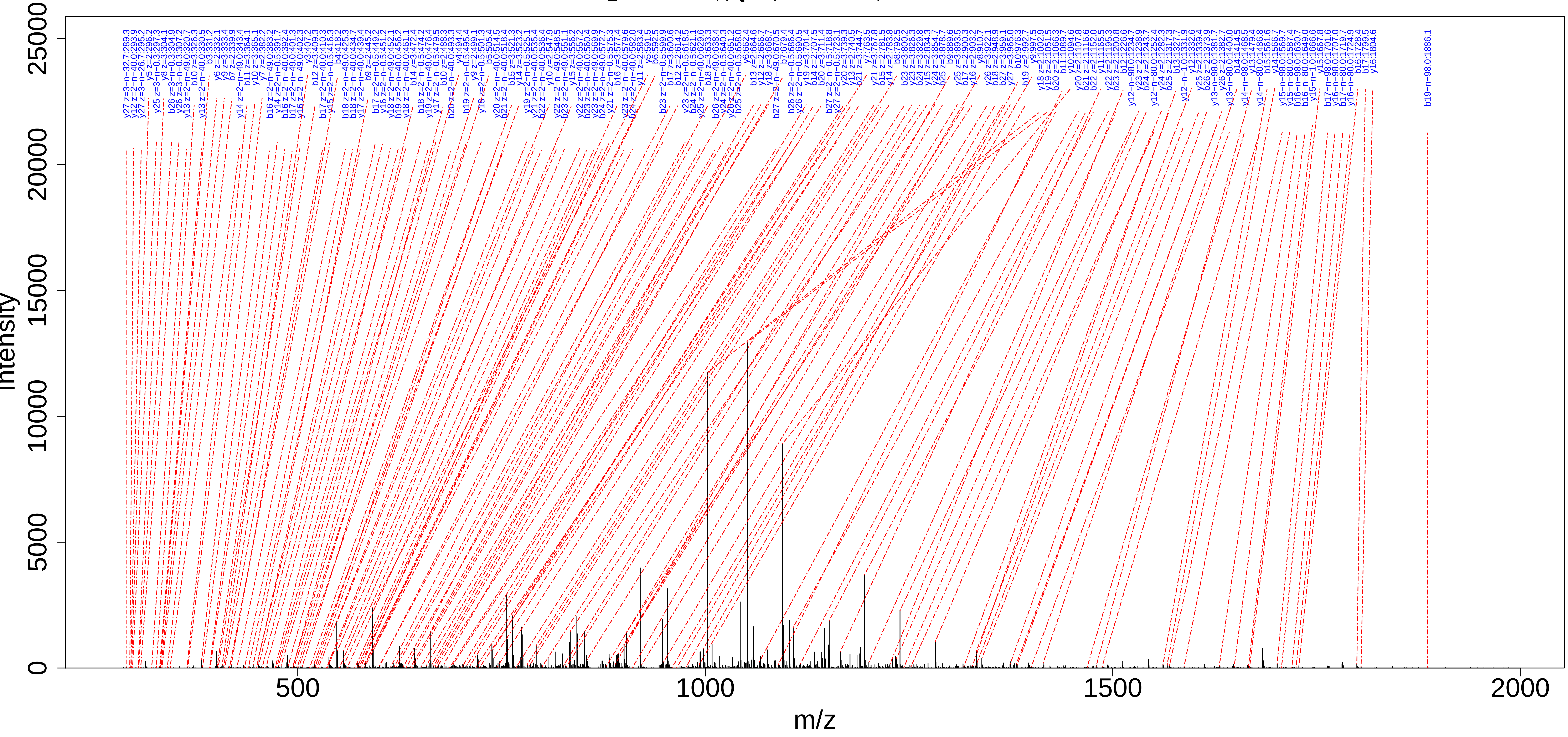

SLTSSLENIFSR 3−T_Phospho z=+2 Raw scan=4611
TBCD4_HUMAN S570; ; Q= 0 ; score= 52.5 ; E= 1.7e−05 m/z=717.3383

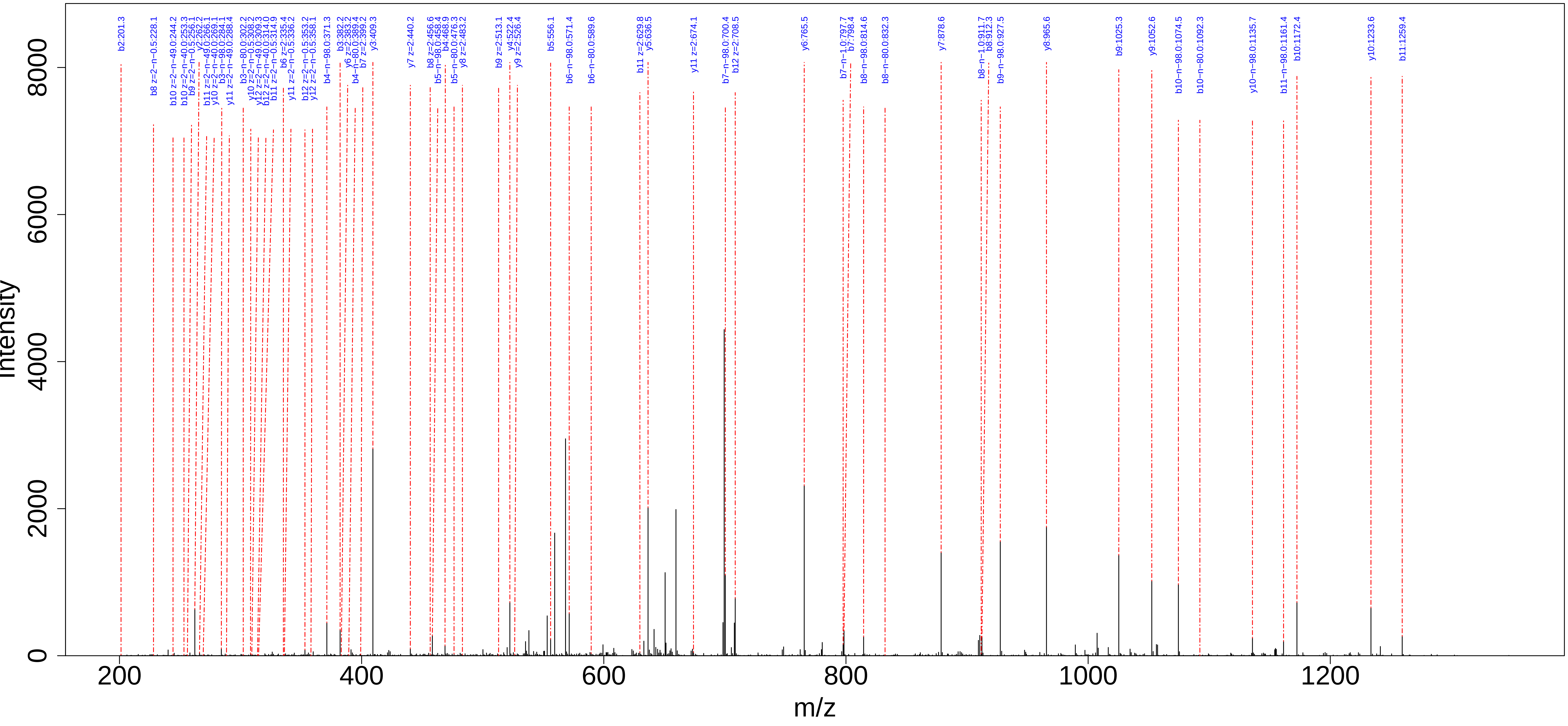

ESNSSSSLPSLHTSFSAPSFTAPSFLK 16−S_Phospho z=+3 Raw scan=4254
TBCD4_HUMAN S692; ; Q= 0 ; score= 51.7 ; E= 0.00047 m/z=965.1191

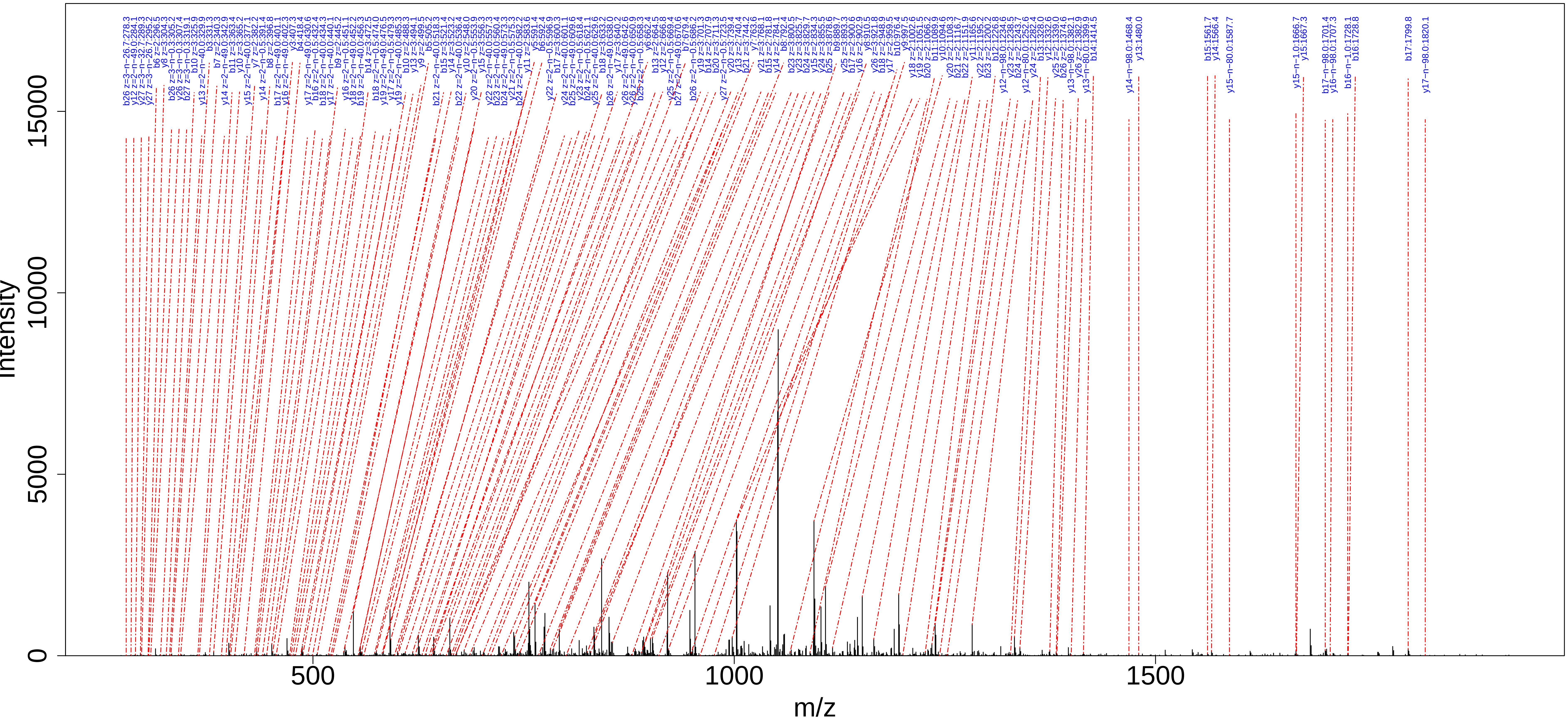

ESNSSSSLPSLHTSFSAPSFTAPSFLK 16−S_Phospho z=+3 Raw scan=4240
TBCD4_HUMAN S692; ; Q= 0 ; score= 50.5 ; E= 0.00033 m/z=965.1177

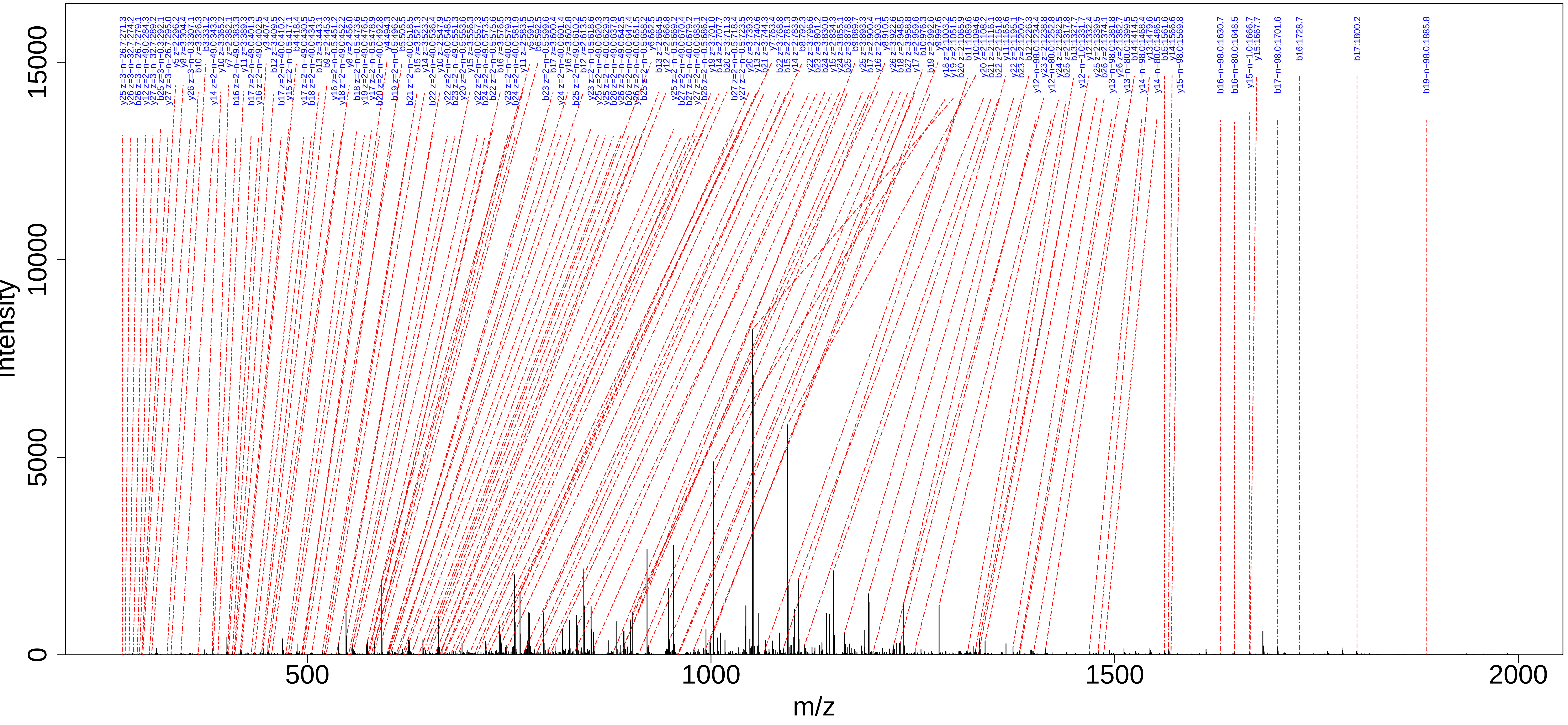

QSSSEQCSNLSSVR 3−S_Phospho z=+2 Raw scan=1572
TBCD4_HUMAN S681; ; Q= 0 ; score= 49.3 ; E= 4.6e−07 m/z=824.835

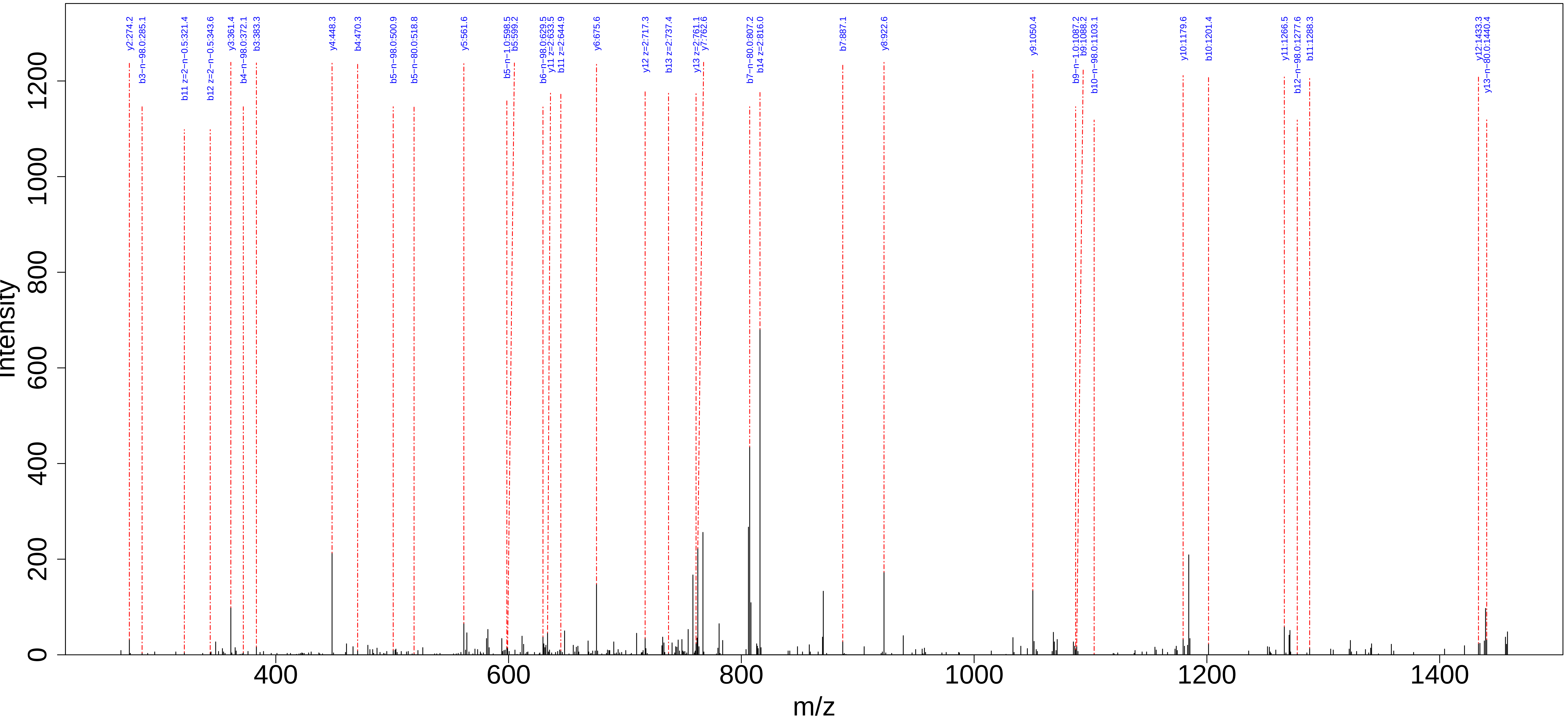

SLTSSLENIFSR 3−T_Phospho z=+2 Raw scan=4649
TBCD4_HUMAN S569; ; Q= 0 ; score= 48.7 ; E= 1.5e−05 m/z=717.3369

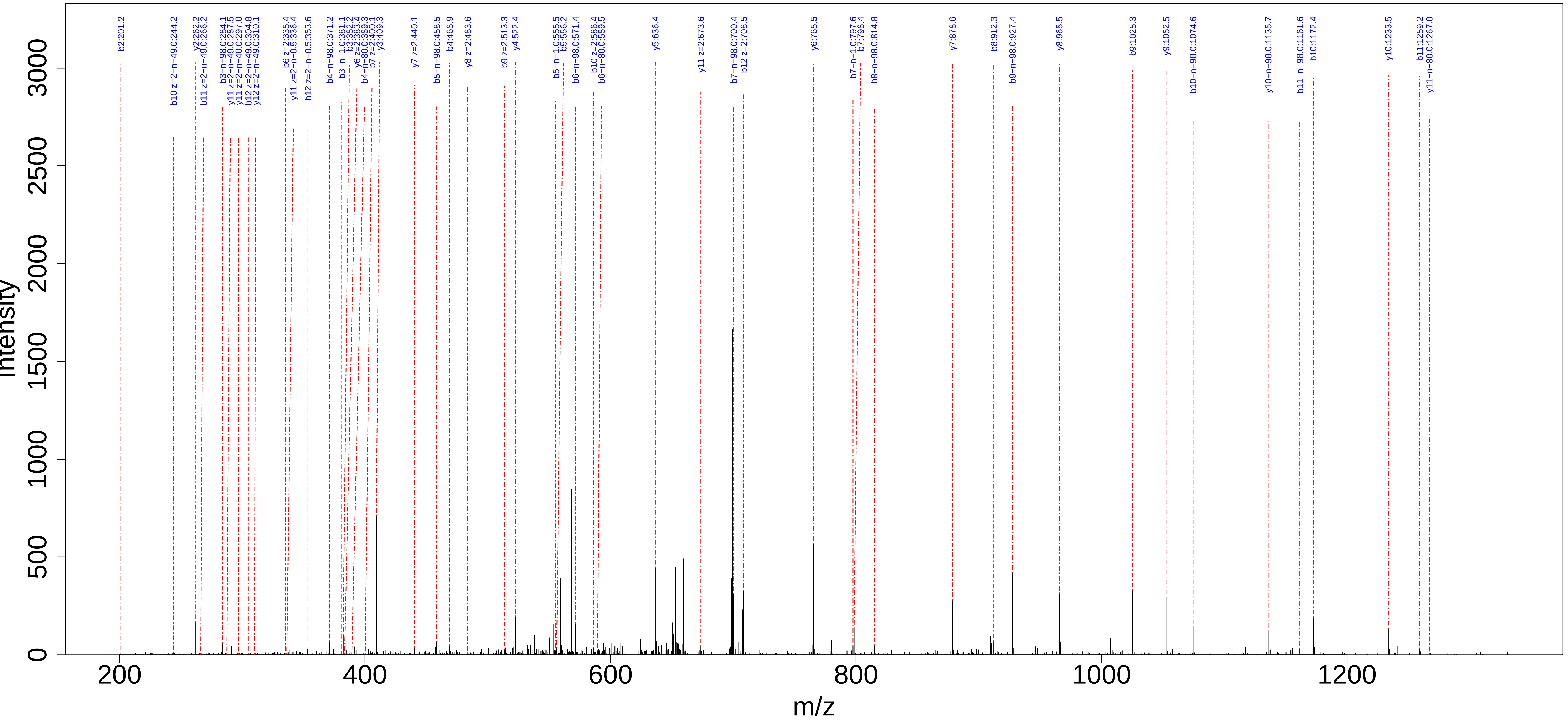

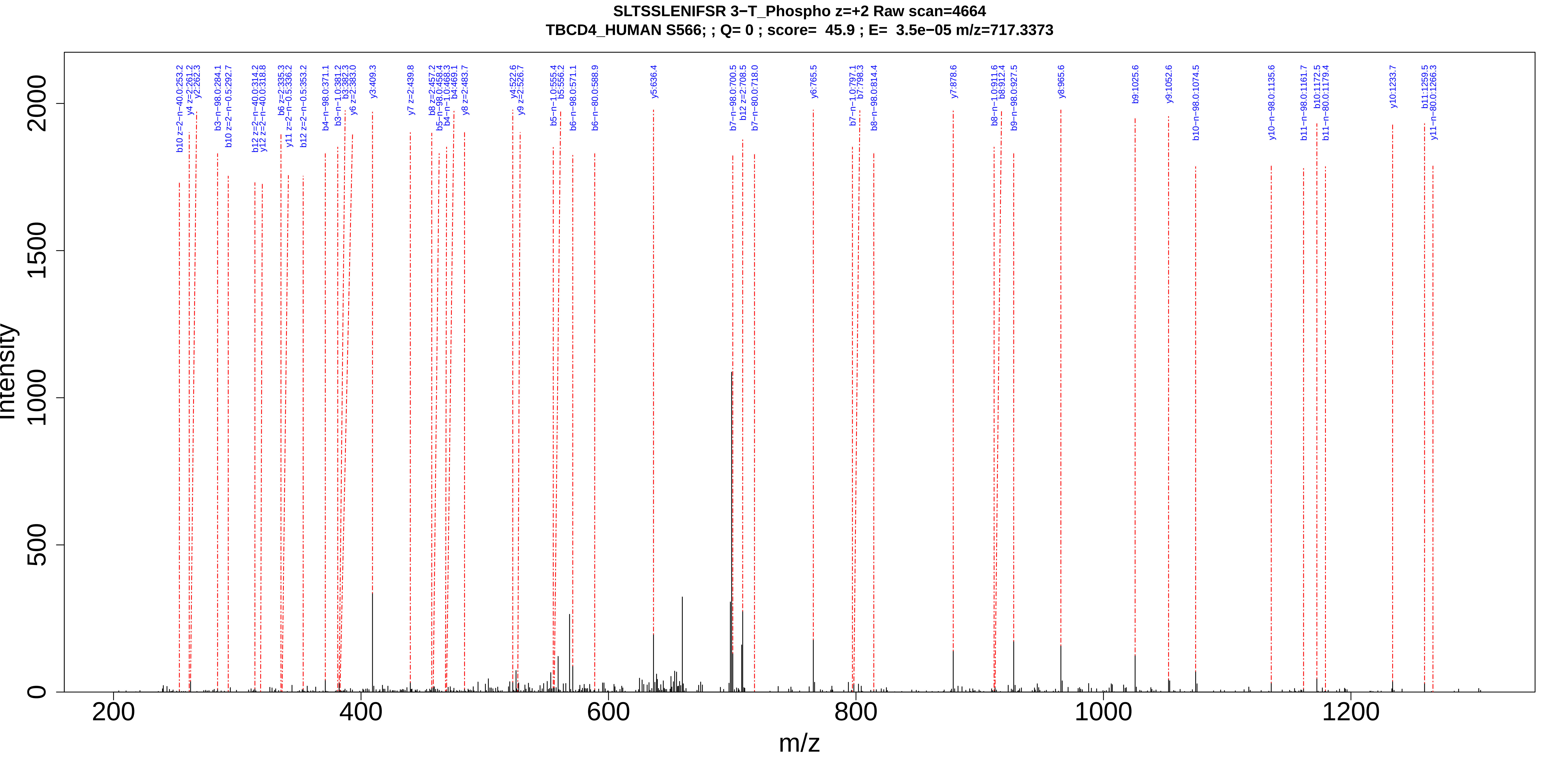

SLTSSLENIFSR 3−T_Phospho z=+2 Raw scan=4664
TBCD4_HUMAN S566; ; Q= 0 ; score= 45.9 ; E= 3.5e−05 m/z=717.3373
Intensity
m/z
0
500
1000
1500
2000
200
400
600
800
1000
1200
b10 z=2−n−40.0:253.2
y4 z=2:261.2
y2:262.3
b3−n−98.0:284.1
b10 z=2−n−0.5:292.7
b12 z=2−n−40.0:314.2
y12 z=2−n−40.0:318.8
b6 z=2:335.3
y11 z=2−n−0.5:336.2
b12 z=2−n−0.5:353.2
b4−n−98.0:371.1
b3−n−1.0:381.2
b3:382.3
y6 z=2:383.0
y3:409.3
y7 z=2:439.8
b8 z=2:457.2
b5−n−98.0:458.4
b4−n−1.0:468.3
b4:469.1
y8 z=2:483.7
y4:522.6
y9 z=2:526.7
b5−n−1.0:555.4
b5:556.2
b6−n−98.0:571.1
b6−n−80.0:588.9
y5:636.4
b7−n−98.0:700.5
b12 z=2:708.5
b7−n−80.0:718.0
y6:765.5
b7−n−1.0:797.1
b7:798.3
b8−n−98.0:814.4
y7:878.6
b8−n−1.0:911.6
b8:912.4
b9−n−98.0:927.5
y8:965.6
b9:1025.6
y9:1052.6
b10−n−98.0:1074.5
y10−n−98.0:1135.6
b11−n−98.0:1161.7
b10:1172.5
b11−n−80.0:1179.4
y10:1233.7
b11:1259.5
y11−n−80.0:1266.3

SLTSSLENIFSR 3−T_Phospho z=+2 Raw scan=4633
TBCD4_HUMAN S576; ; Q= 0 ; score= 45.8 ; E= 3.1e−05 m/z=717.3374

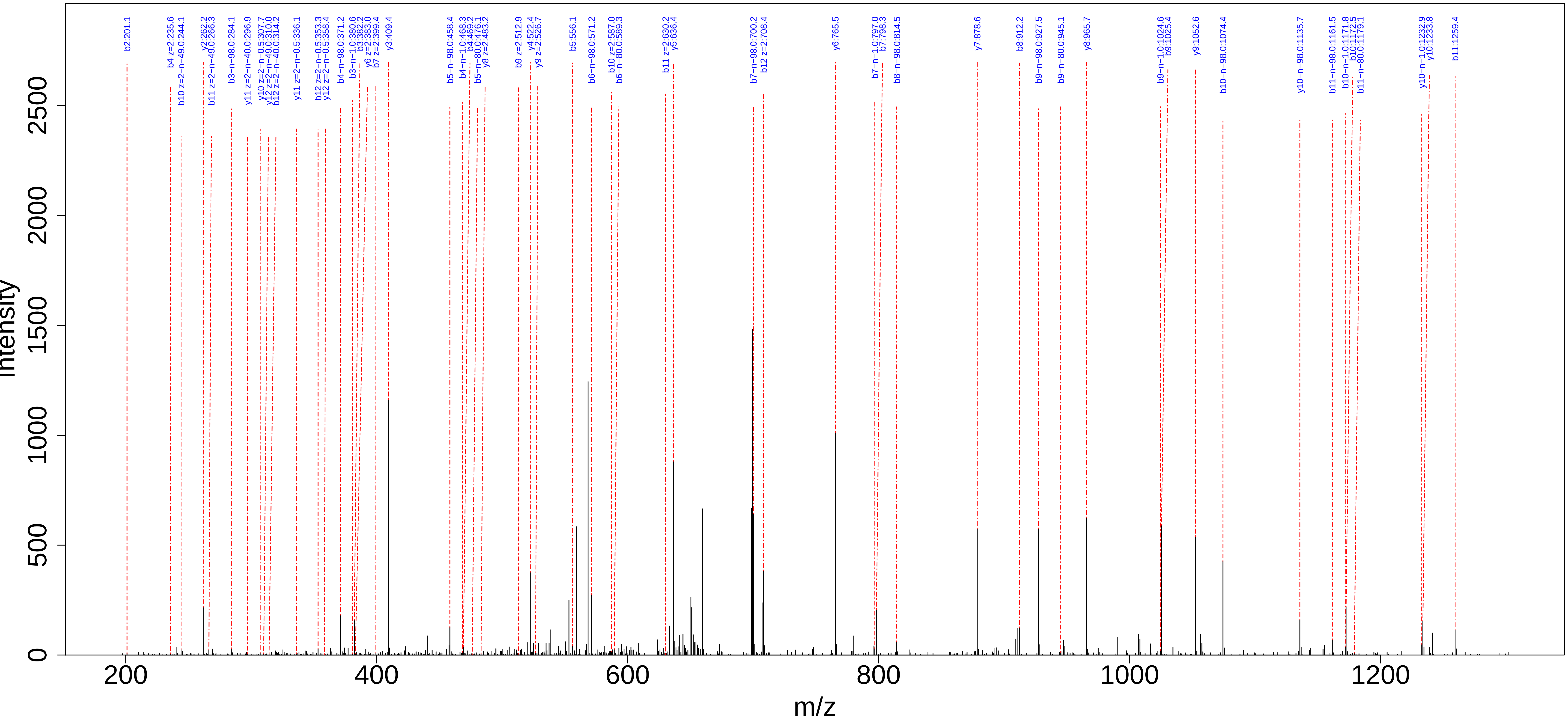

GRLGSVDSFER 5−S_Phospho z=+2 Raw scan=2420
TBCD4_HUMAN S591; ; Q= 0 ; score= 45.8 ; E= 0.0013 m/z=651.7958

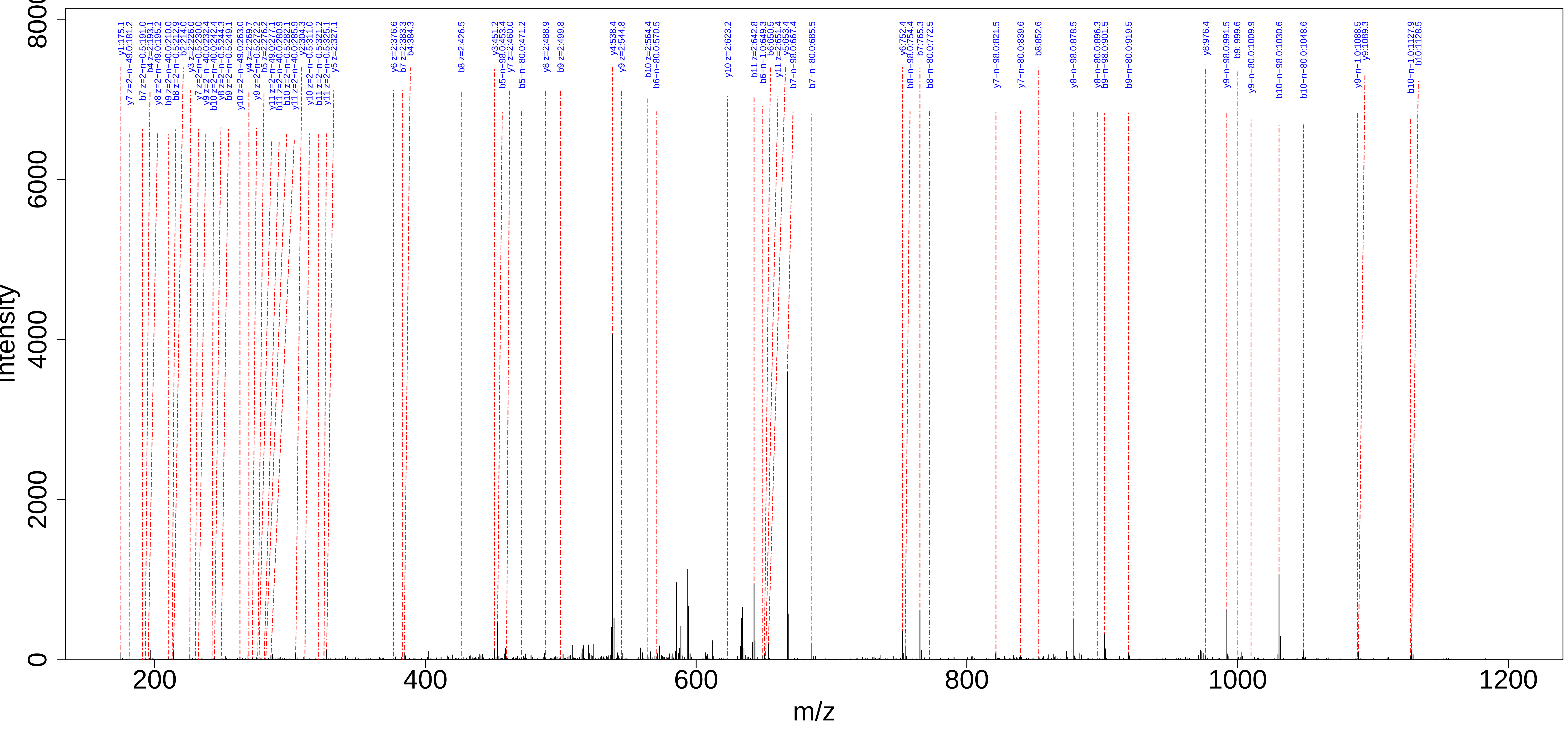

QSSSEQCSNLSSVR 2−S_Phospho z=+2 Raw scan=1565
TBCD4_HUMAN S673; ; Q= 0.000367376928728876 ; score= 42.4 ; E= 1.8e−05 m/z=824.8365

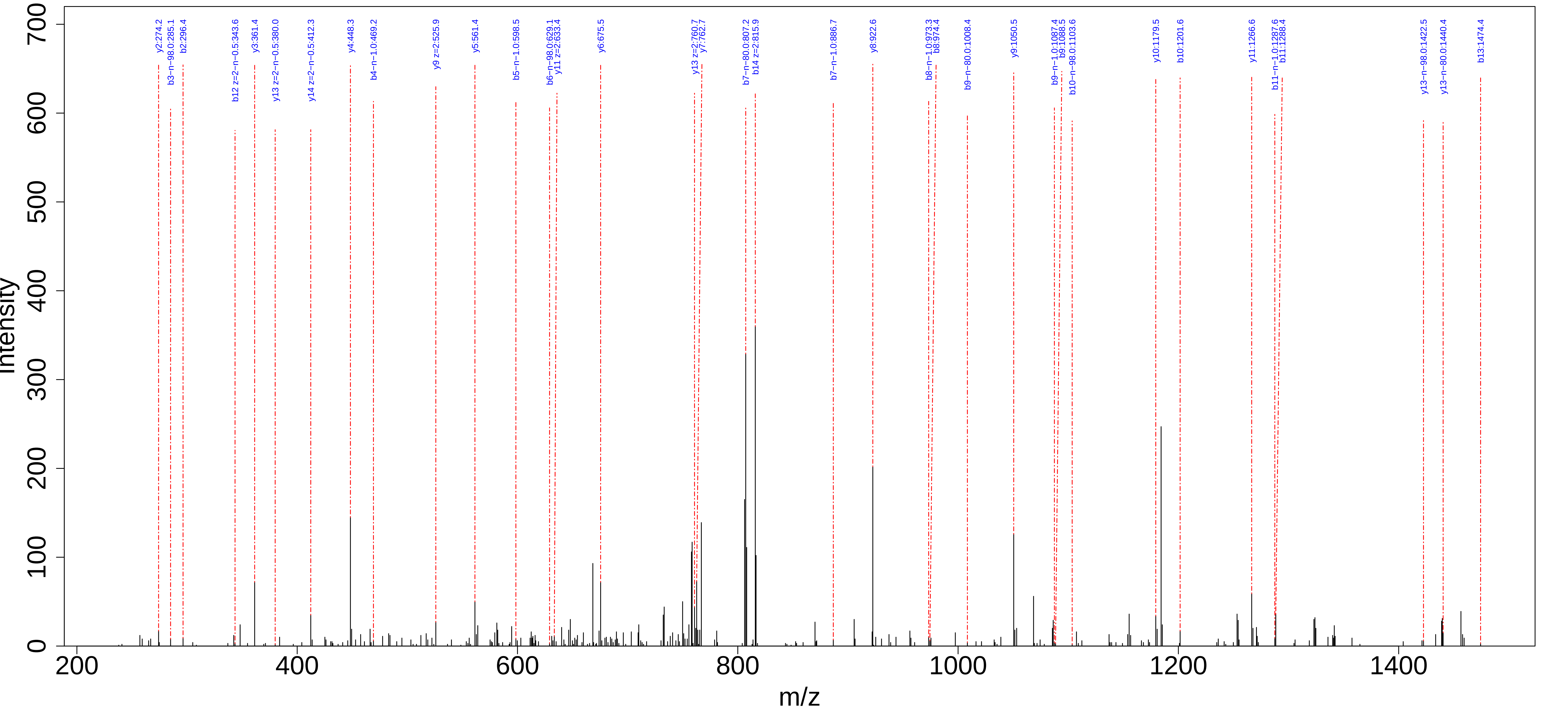

QSSSEQCSNLSSVR 3−S_Phospho z=+2 Raw scan=1565

TBCD4_HUMAN S673; ; Q= 0.000367242012486228 ; score= 42.4 ; E= 1.8e−05 m/z=824.8365

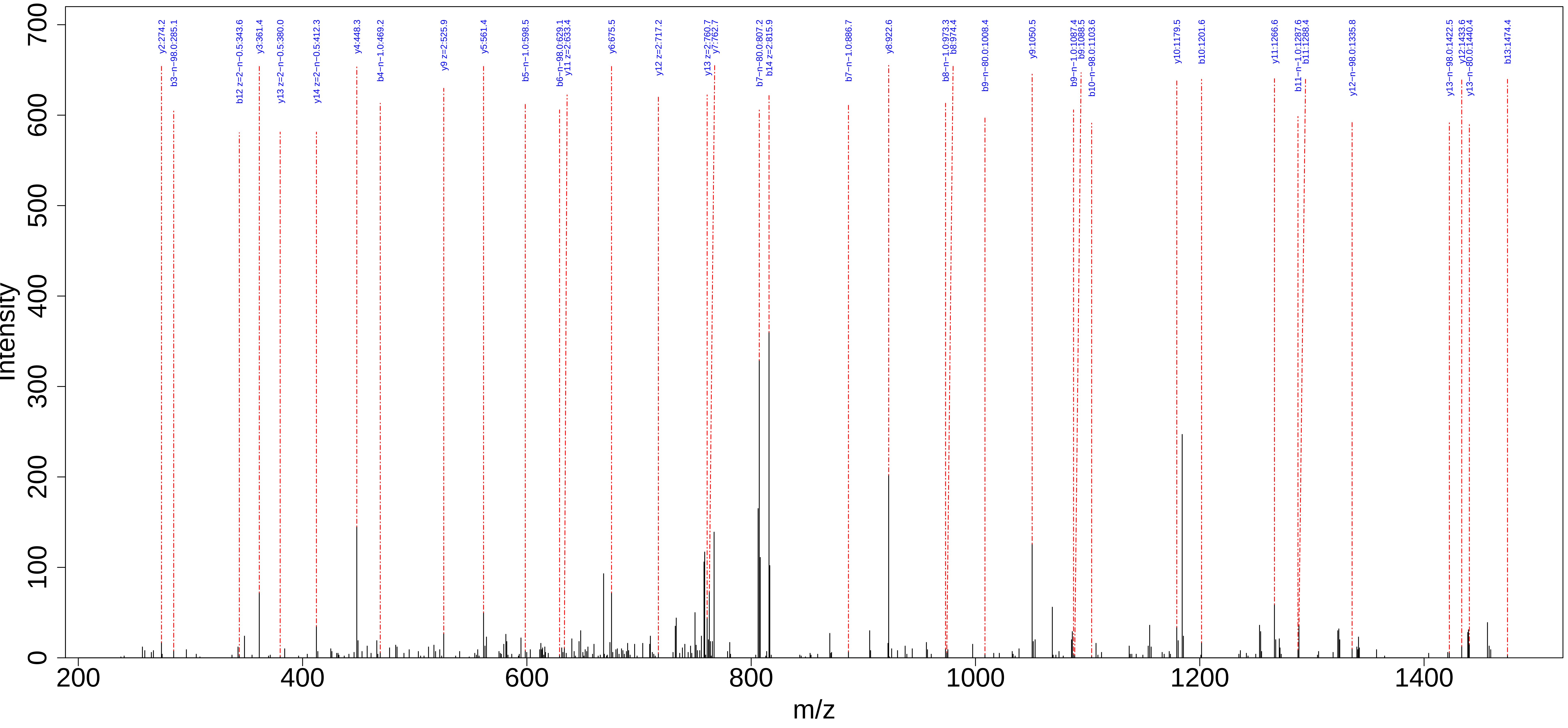

SLTSSLENIFSR 3−T_Phospho z=+2 Raw scan=4642
TBCD4_HUMAN S570; ; Q= 0.00590072891357168 ; score= 41.6 ; E= 0.0025 m/z=717.3376

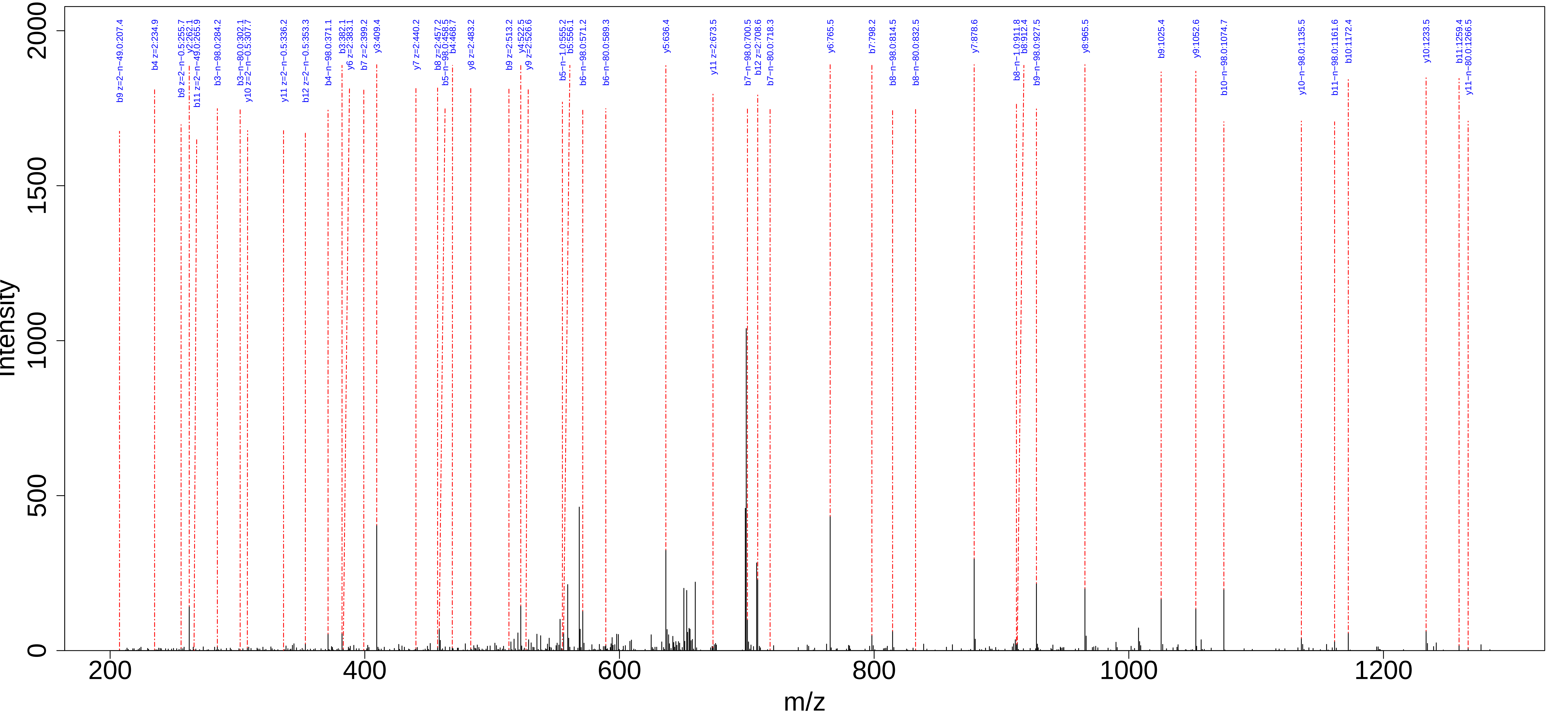

SLTSSLENIFSR 3−T_Phospho z=+2 Raw scan=4671
TBCD4_HUMAN S569; ; Q= 0.00560501153972964 ; score= 40.2 ; E= 0.0015 m/z=717.3375

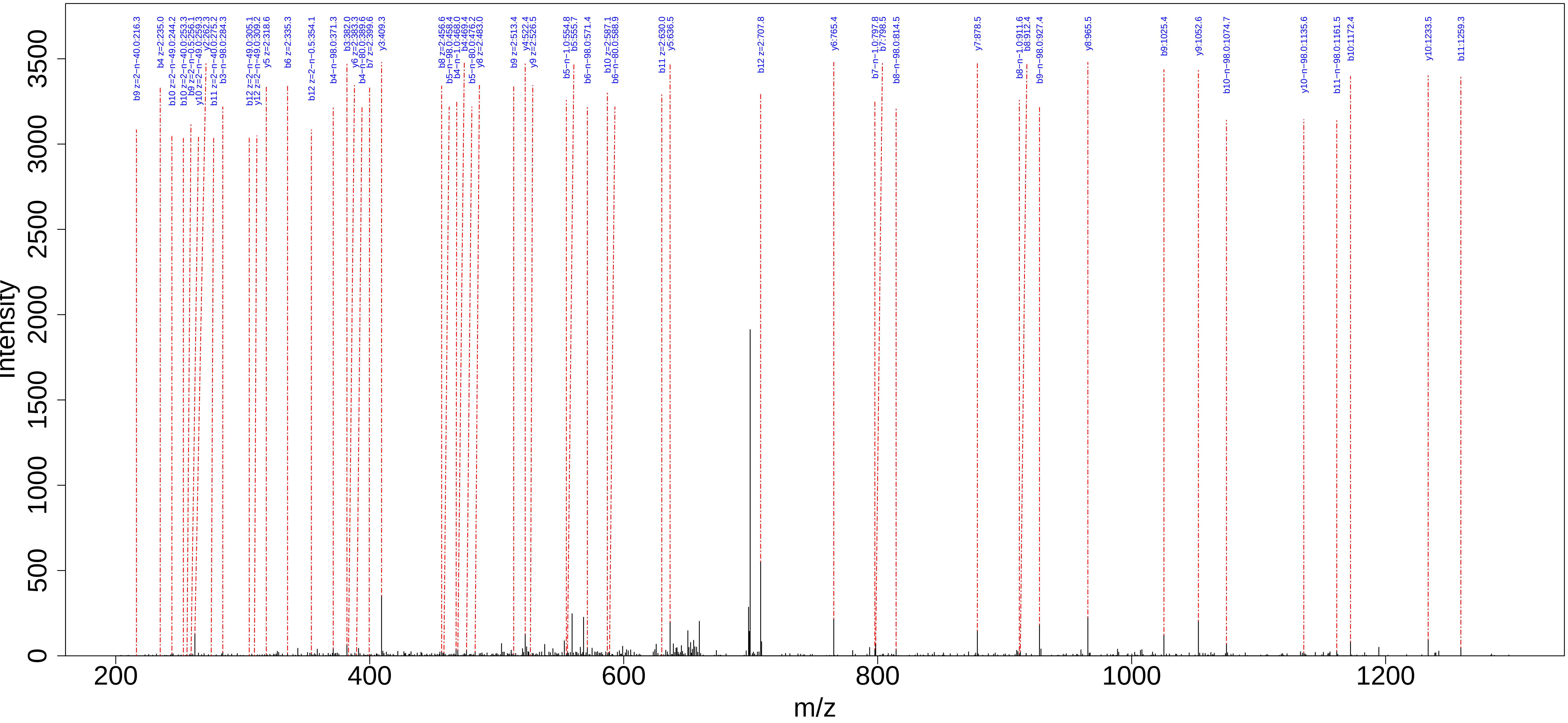

SLTSSLENIFSR 3−T_Phospho z=+2 Raw scan=4678

TBCD4_HUMAN T568; ; Q= 0.00523560209424084 ; score= 38.9 ; E= 0.001 m/z=717.3369

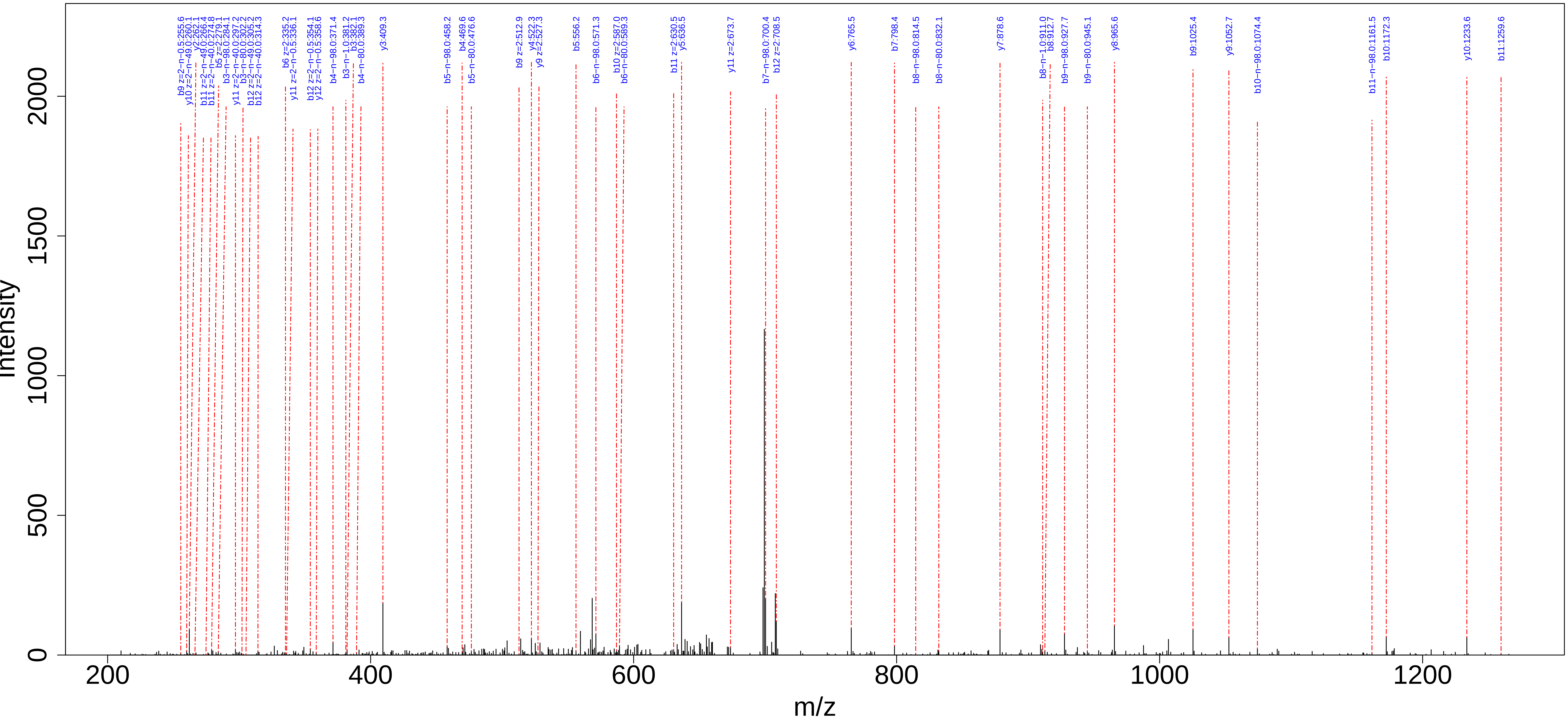

GRLGSVDSFER 5−S_Phospho z=+2 Raw scan=2390

TBCD4_HUMAN S591; ; Q= 0.0077542499254399 ; score= 38 ; E= 0.0091 m/z=651.796

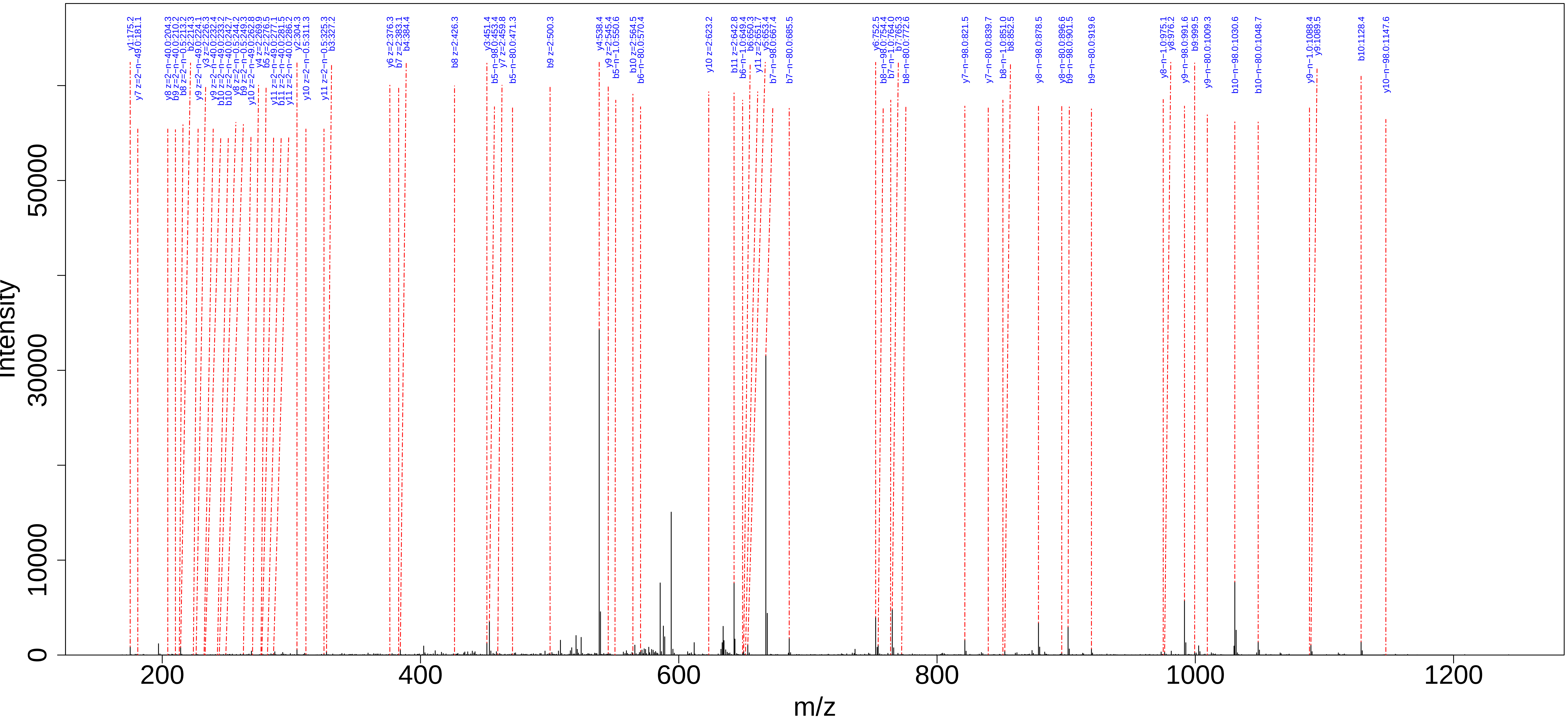

GRLGSVDSFER 5−S_Phospho z=+2 Raw scan=2406
TBCD4_HUMAN S591; ; Q= 0.00775193798449612 ; score= 38 ; E= 0.019 m/z=651.7952

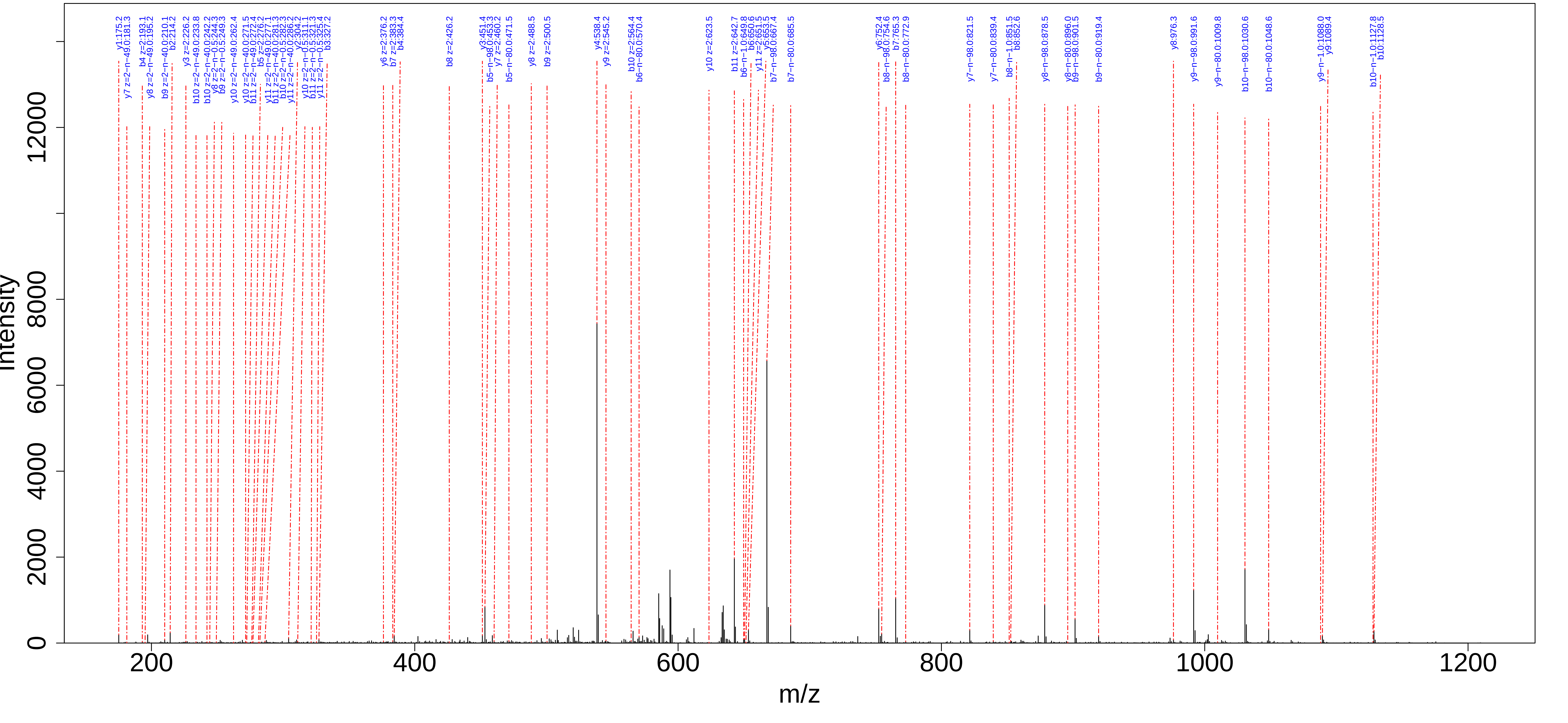

GRLGSVDSFER 5−S_Phospho z=+2 Raw scan=2369
TBCD4_HUMAN S591; ; Q= 0.00774962742175857 ; score= 38 ; E= 0.017 m/z=651.7973

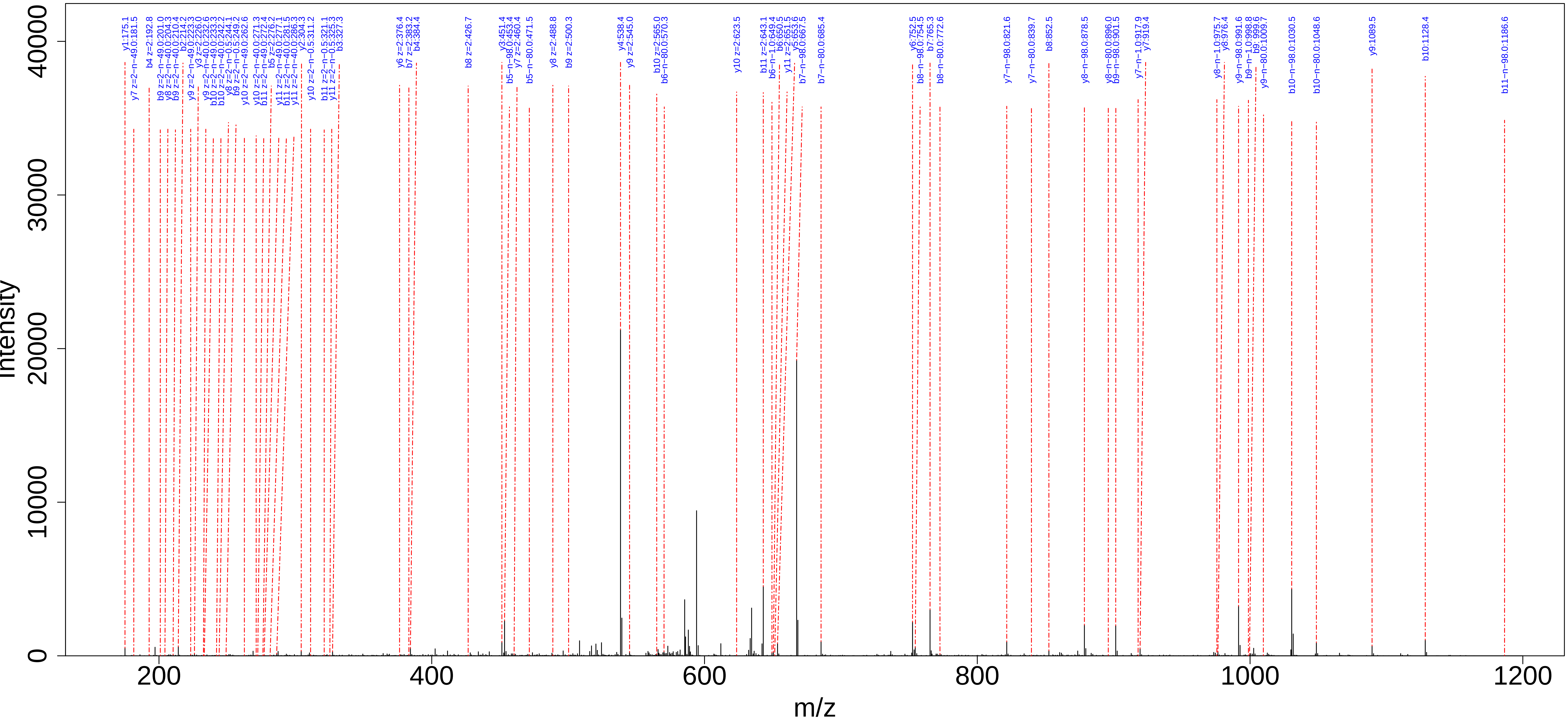

GRLGSVDSFER 5−S_Phospho z=+2 Raw scan=2382
TBCD4_HUMAN S591; ; Q= 0.00774731823599523 ; score= 38 ; E= 0.004 m/z=651.7959

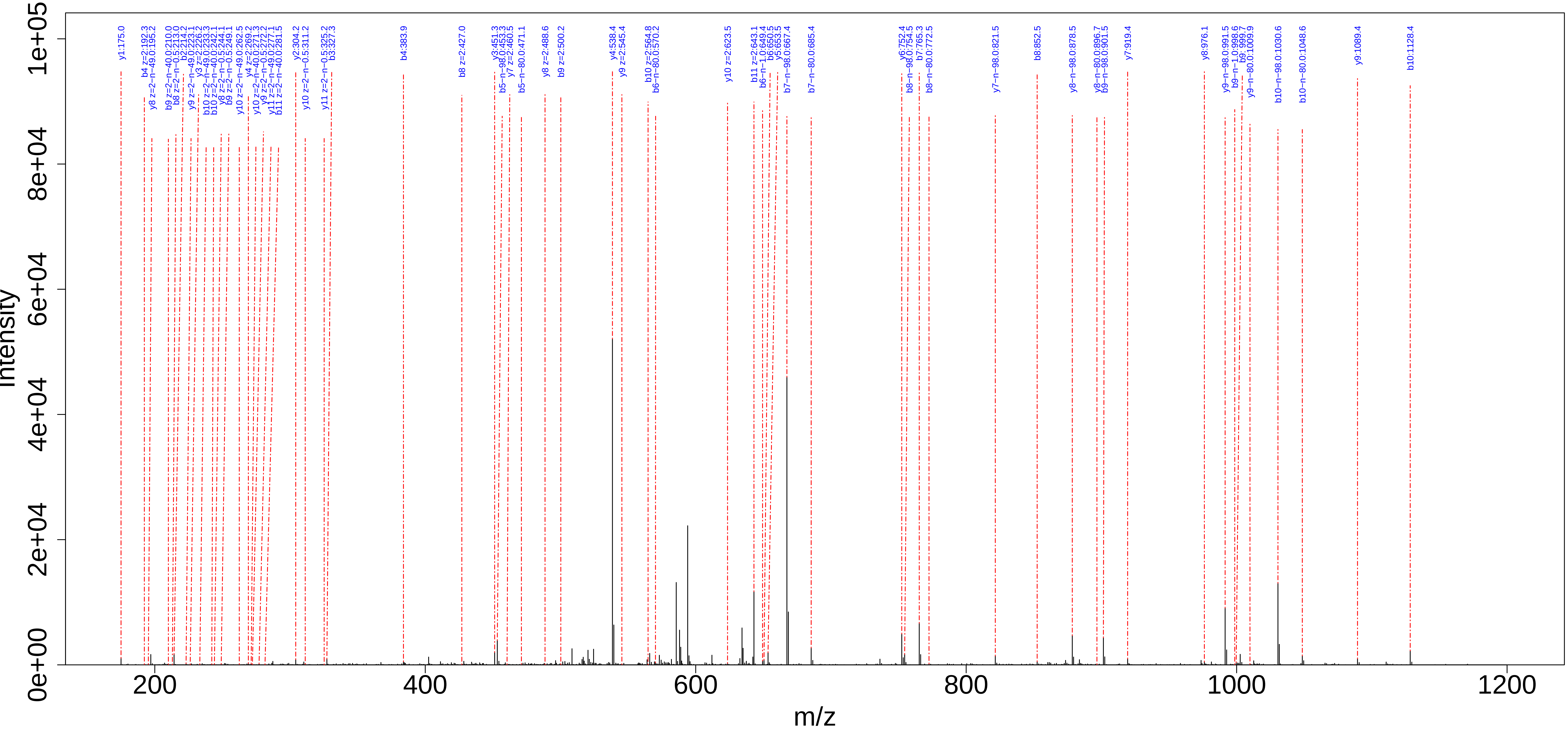

GRLGSVDSFER 5−S_Phospho z=+2 Raw scan=2375
TBCD4_HUMAN S591; ; Q= 0.00774501042597557 ; score= 38 ; E= 0.0014 m/z=651.7962

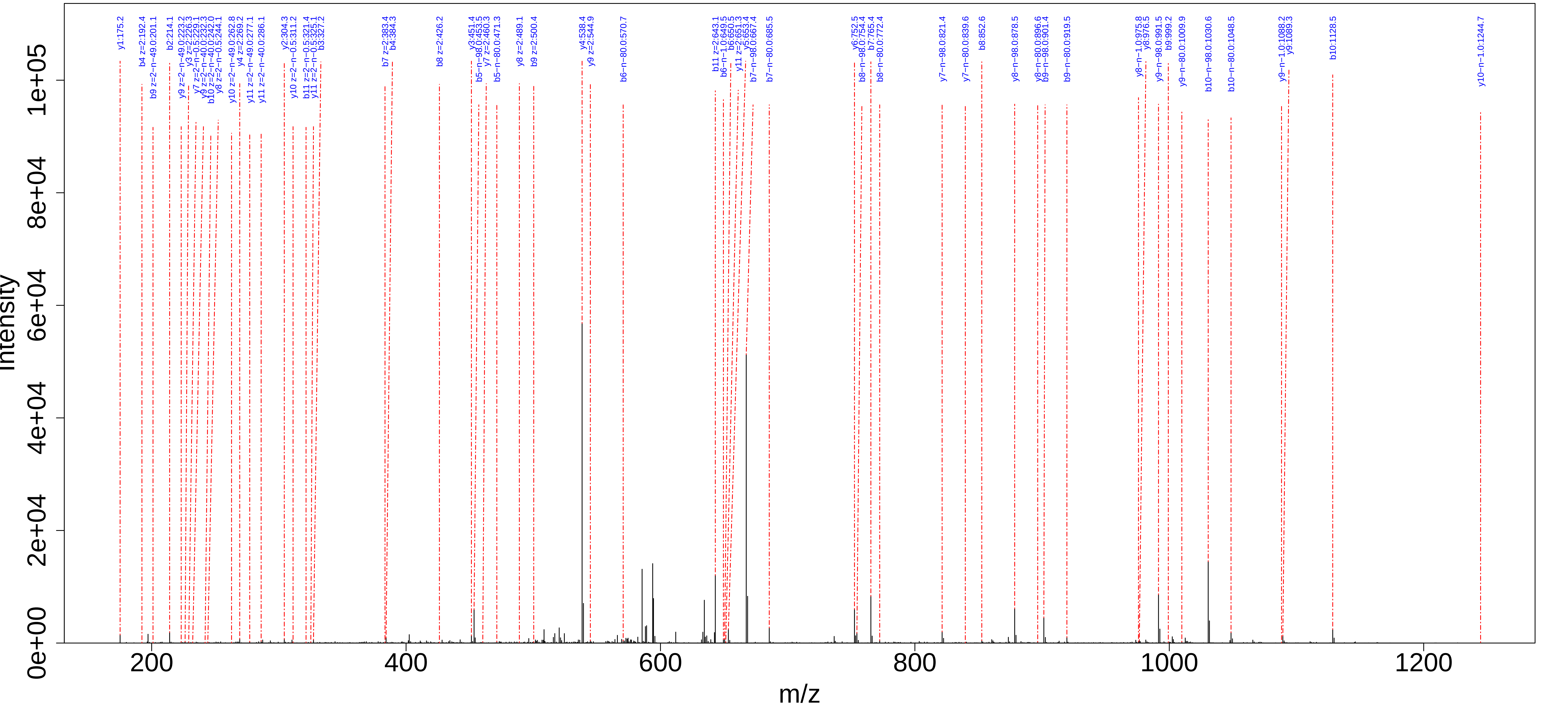

QSSSEQCSNLSSVR 3−S_Phospho z=+2 Raw scan=1558
TBCD4_HUMAN S681; ; Q= 0.00757355083017769 ; score= 37.1 ; E= 0.00019 m/z=824.8346

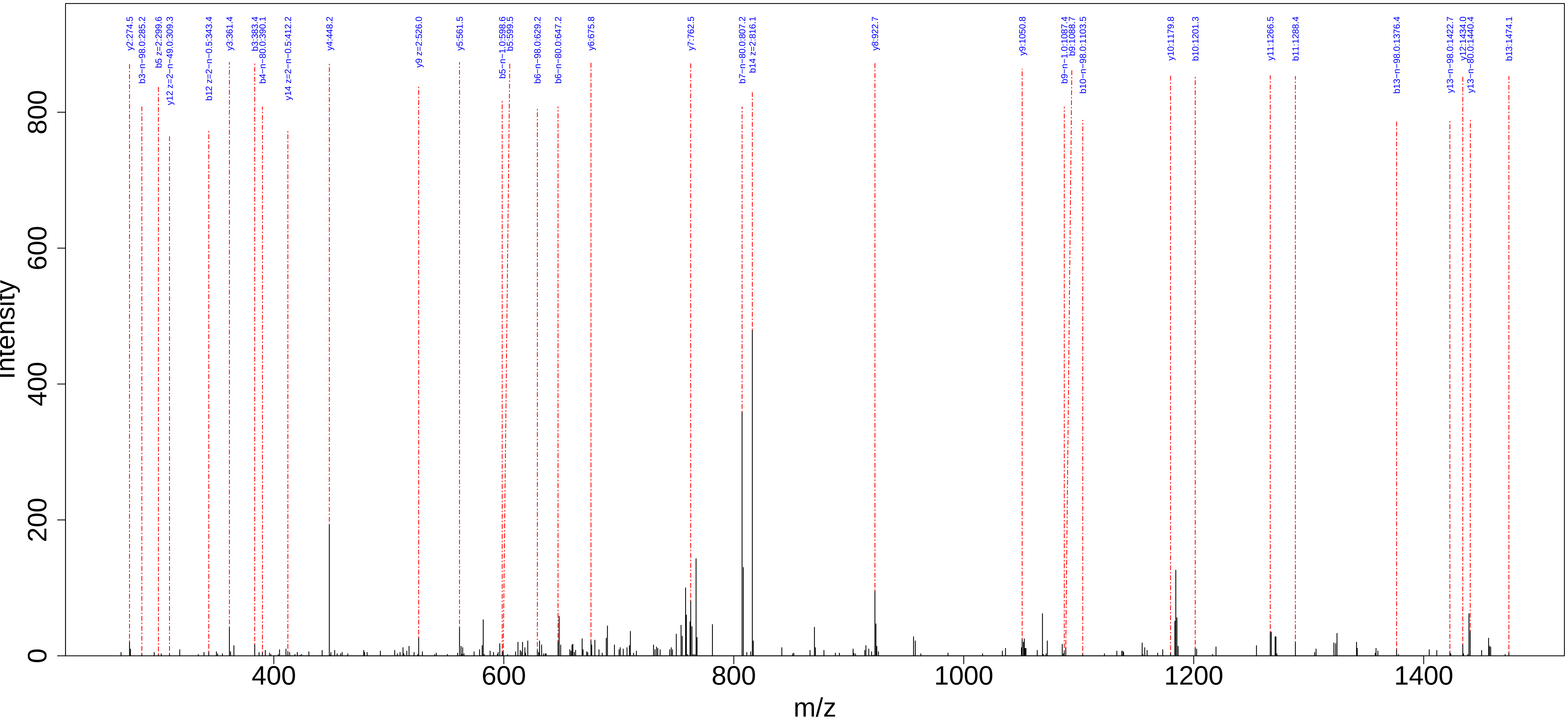

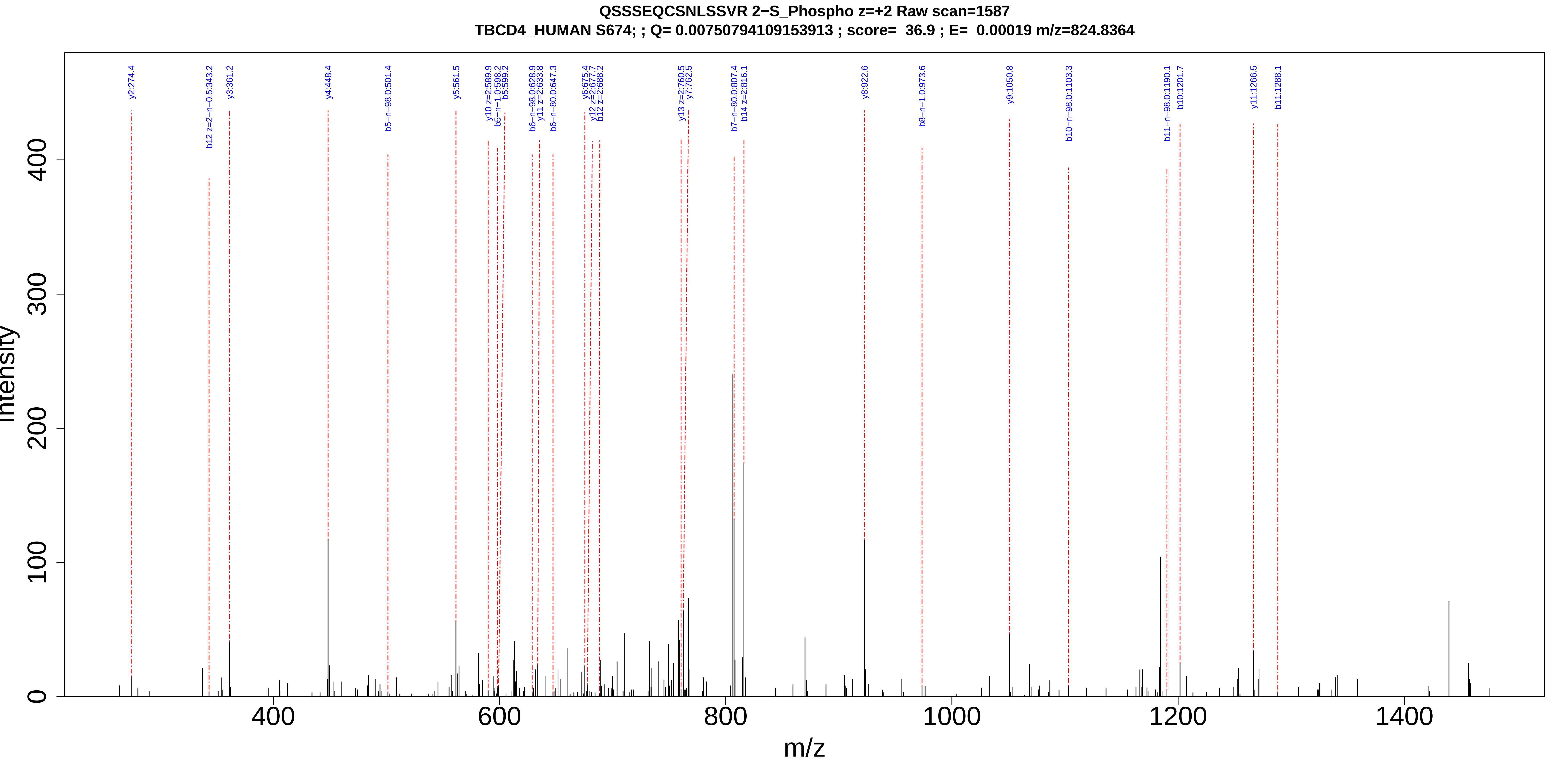

QSSSEQCSNLSSVR 2−S_Phospho z=+2 Raw scan=1587
TBCD4_HUMAN S674; ; Q= 0.00750794109153913 ; score= 36.9 ; E= 0.00019 m/z=824.8364
Intensity
m/z

QSSSEQCSNLSSVR 3−S_Phospho z=+2 Raw scan=1587
TBCD4_HUMAN S674; ; Q= 0.00750577367205543 ; score= 36.9 ; E= 0.00019 m/z=824.8364

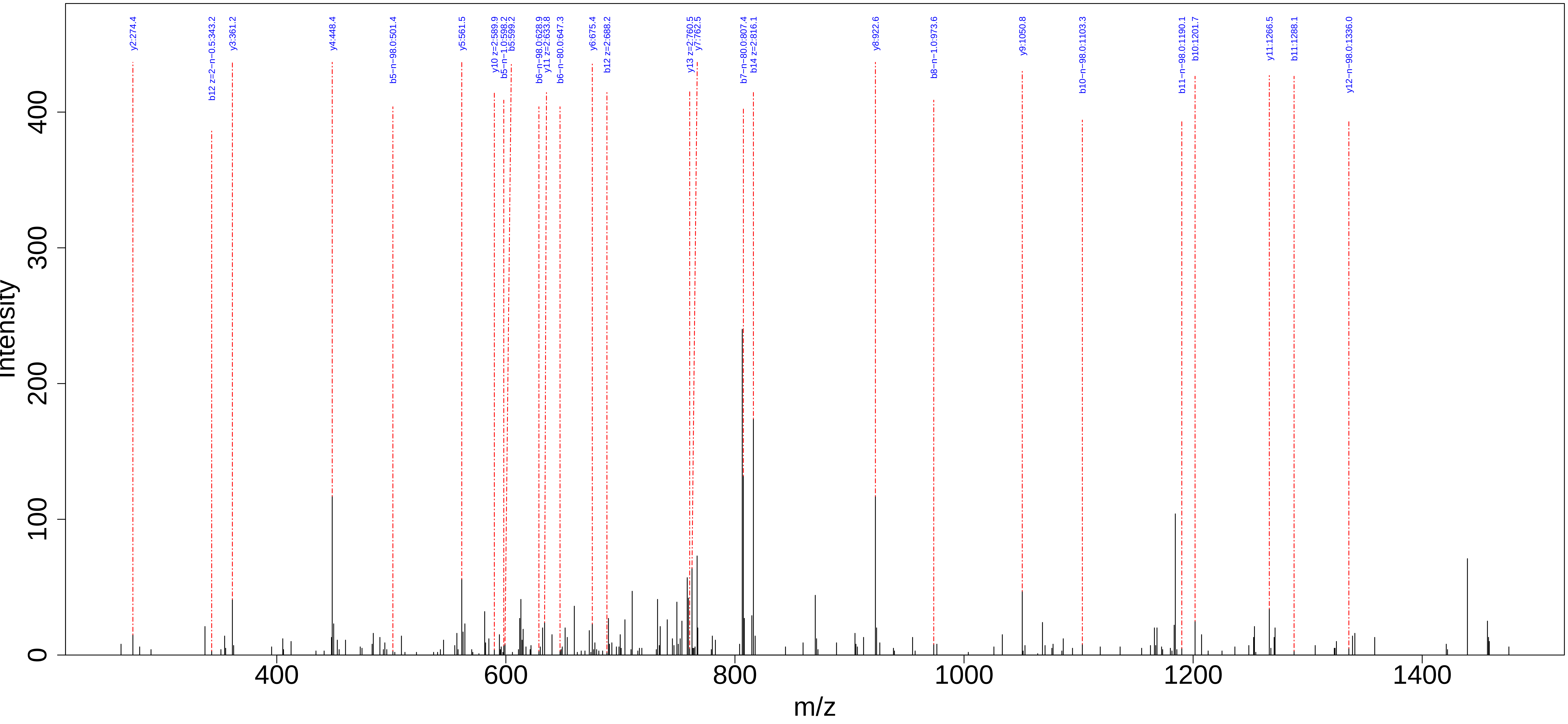

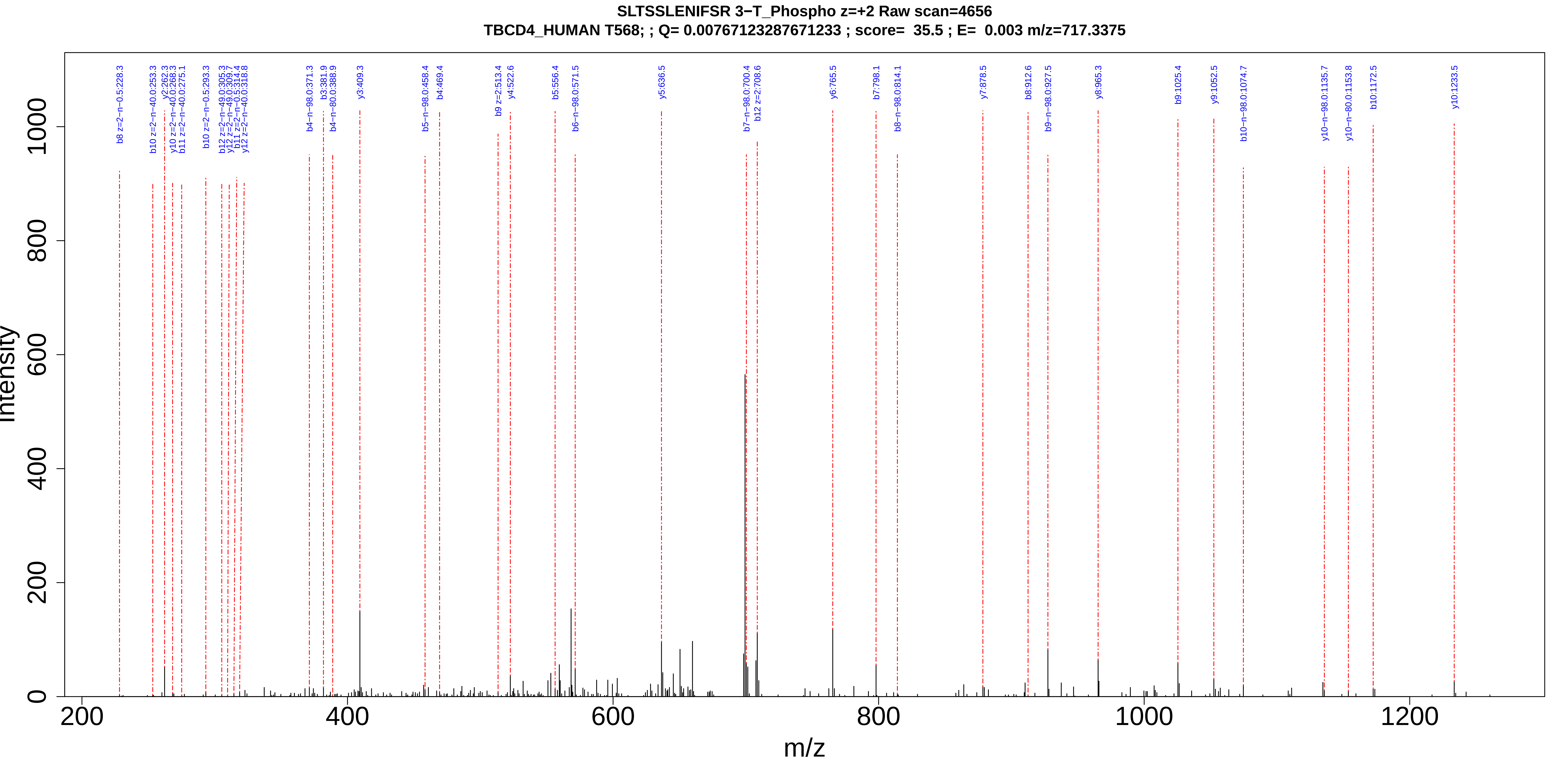

SLTSSLENIFSR 3−T_Phospho z=+2 Raw scan=4656
TBCD4_HUMAN T568; ; Q= 0.00767123287671233 ; score= 35.5 ; E= 0.003 m/z=717.3375
Intensity
m/z

LGSVDSFER 3−S_Phospho z=+2 Raw scan=2543
TBCD4_HUMAN S588; ; Q= 0.00785694933622325 ; score= 35.2 ; E= 0.0028 m/z=545.2341

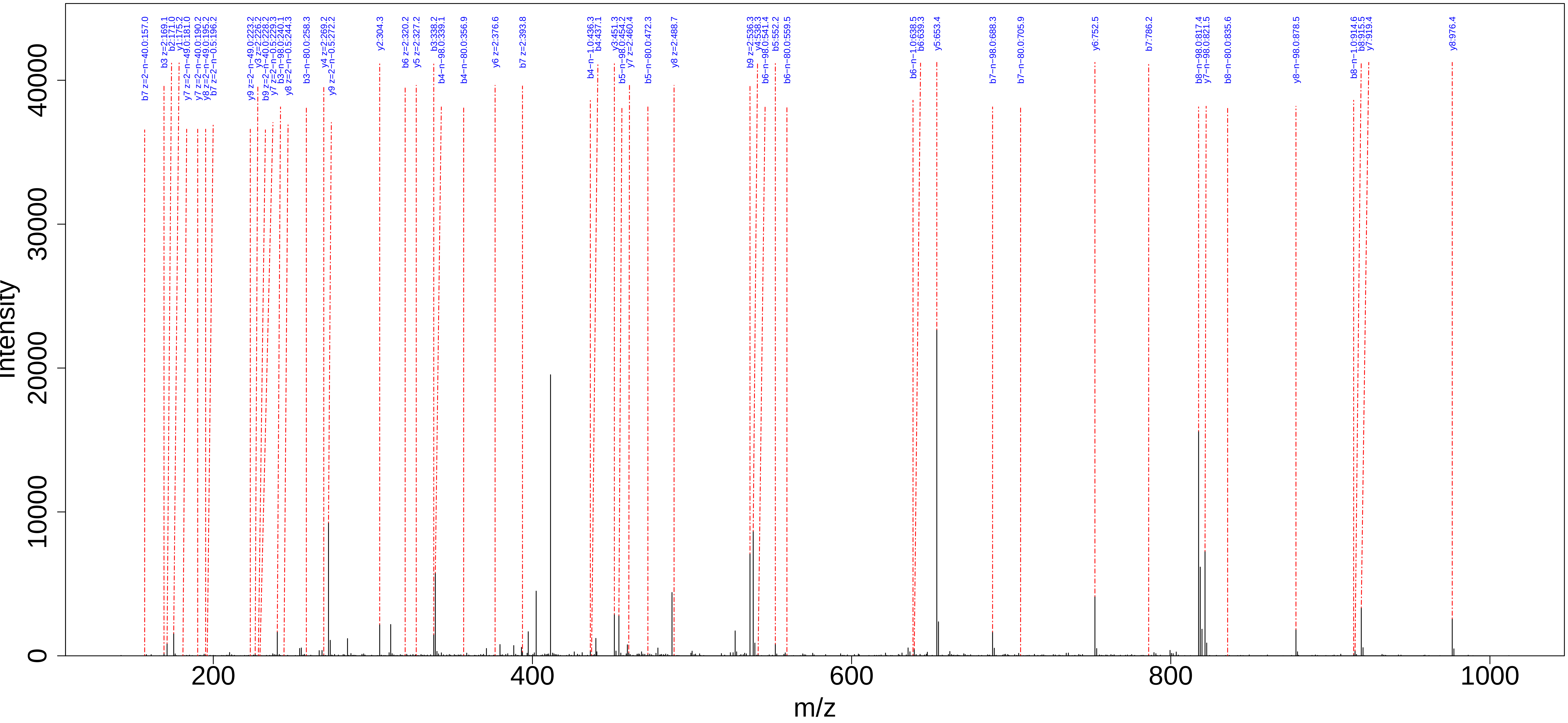

LGSVDSFER 3−S_Phospho z=+2 Raw scan=2551
TBCD4_HUMAN S591; ; Q= 0.00785482123510292 ; score= 35.2 ; E= 0.0063 m/z=545.2341

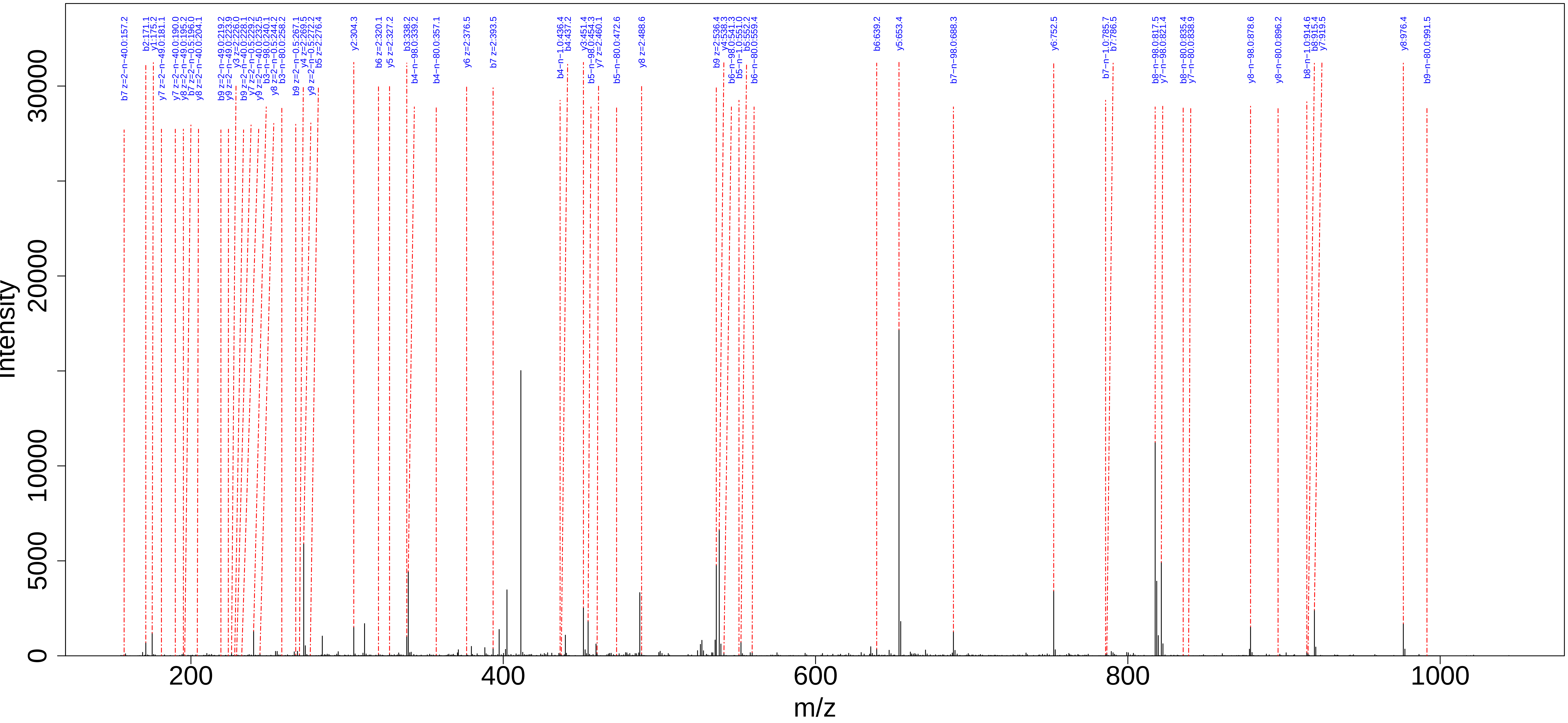

CVPAPGAGASGGTSPSATQPNPAVFIFEHK 14−S_Phospho z=+3 Raw scan=3922
TBCD4_HUMAN T105; ; Q= 0.00865914458147468 ; score= 34.3 ; E= 0.0013 m/z=1011.4744

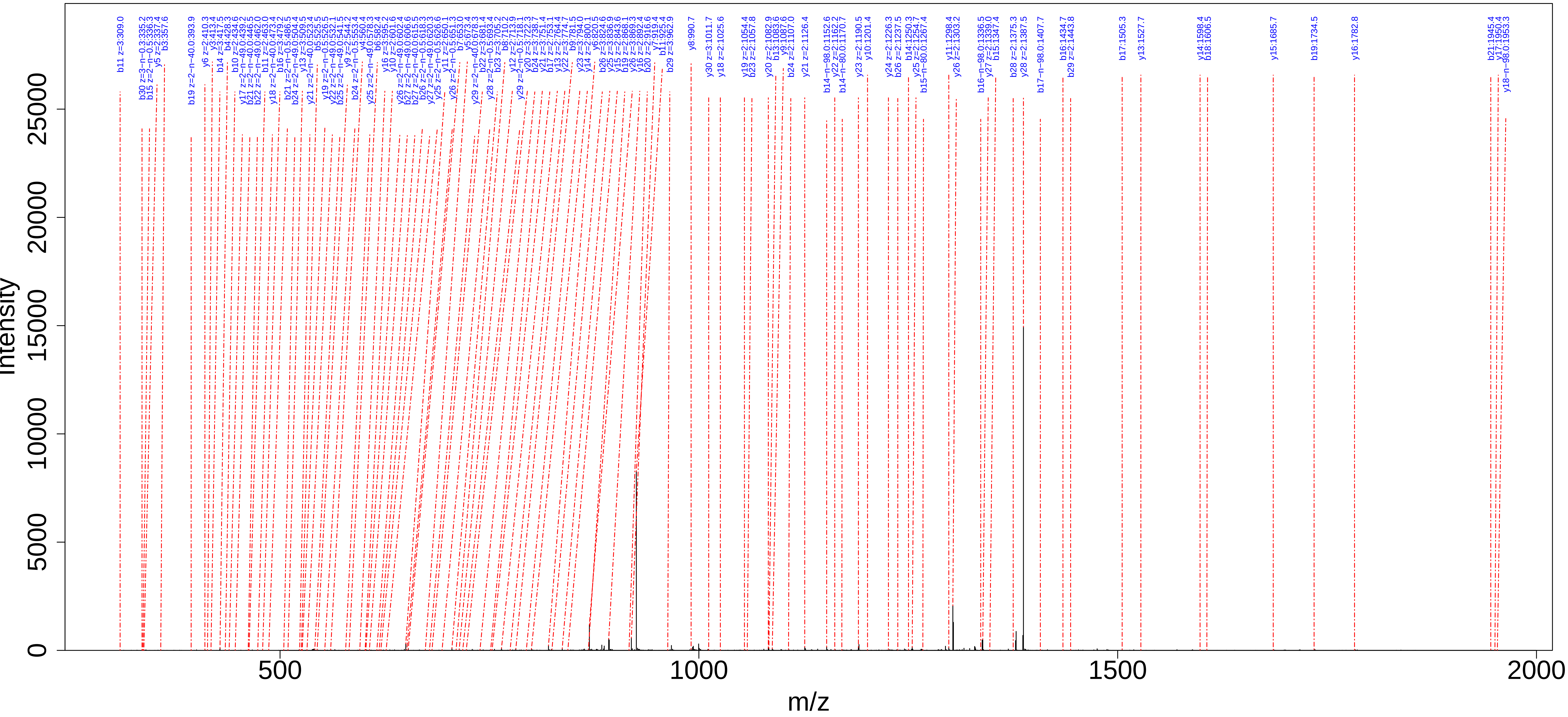

LGSVDSFER 3−S_Phospho z=+2 Raw scan=2536
TBCD4_HUMAN S591; ; Q= 0.00844637829536729 ; score= 32.9 ; E= 0.0099 m/z=545.2326

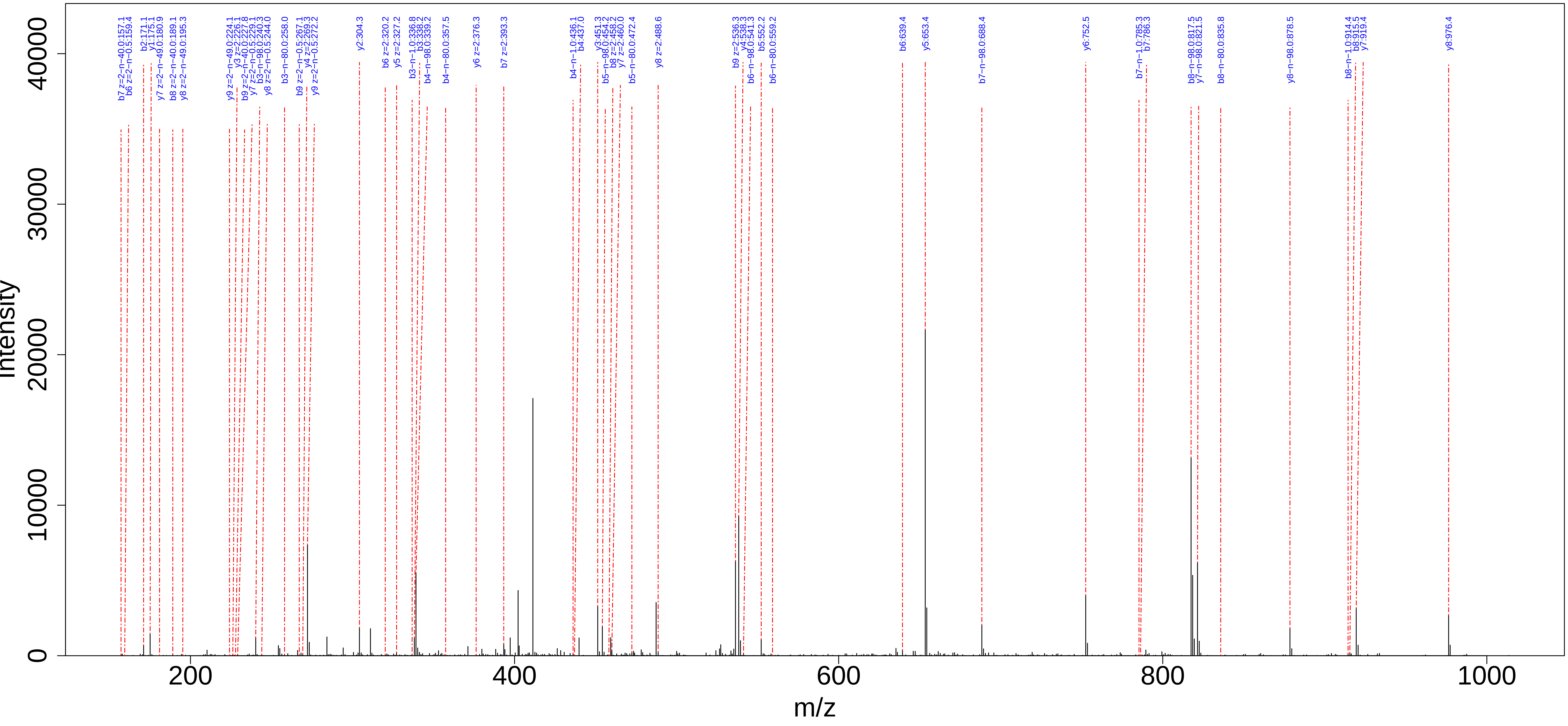

LGSVDSFER 3−S_Phospho z=+2 Raw scan=2558
TBCD4_HUMAN S591; ; Q= 0.00844421699078813 ; score= 32.9 ; E= 0.007 m/z=545.2335

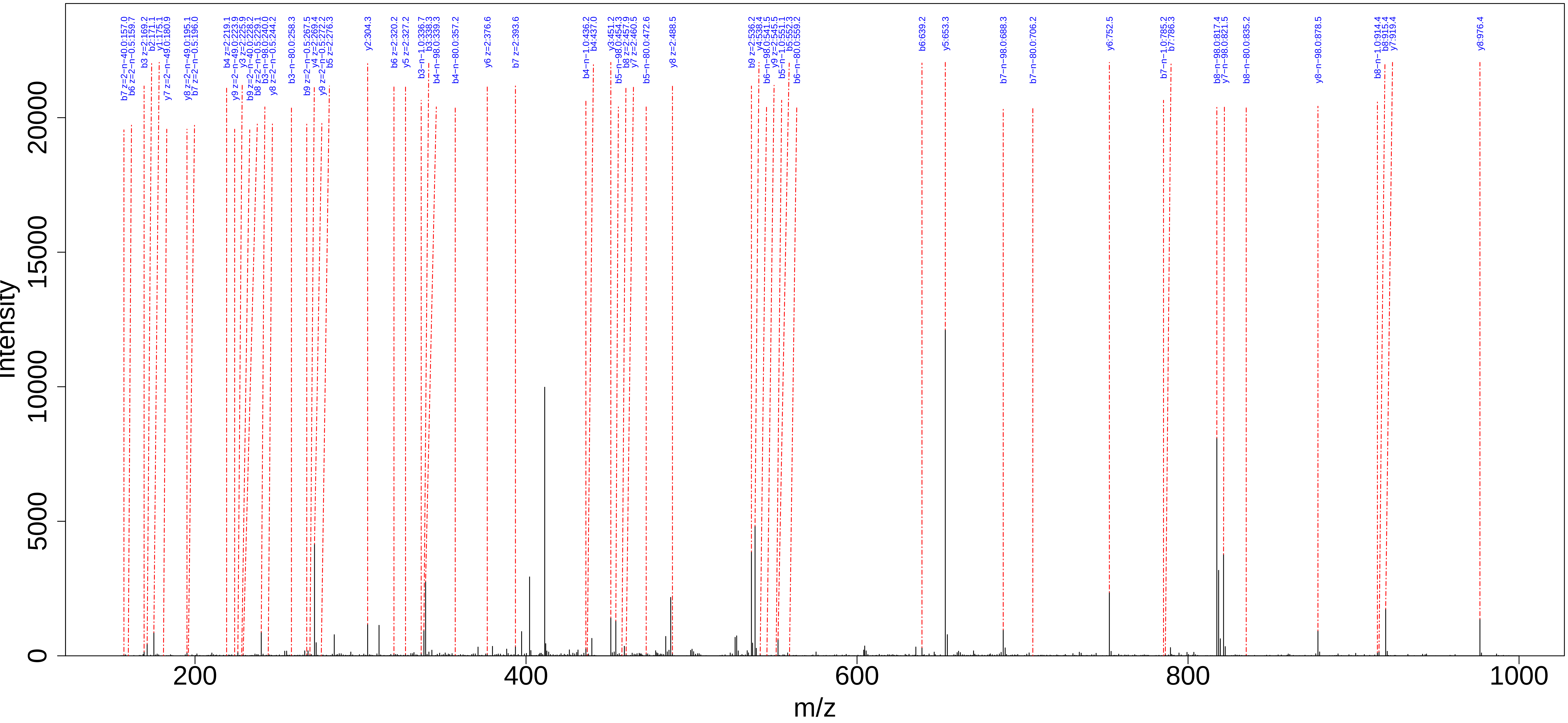

GRLGSVDSFER 5−S_Phospho z=+2 Raw scan=2399
TBCD4_HUMAN S591; ; Q= 0.00842481490936942 ; score= 32.8 ; E= 0.095 m/z=651.7943

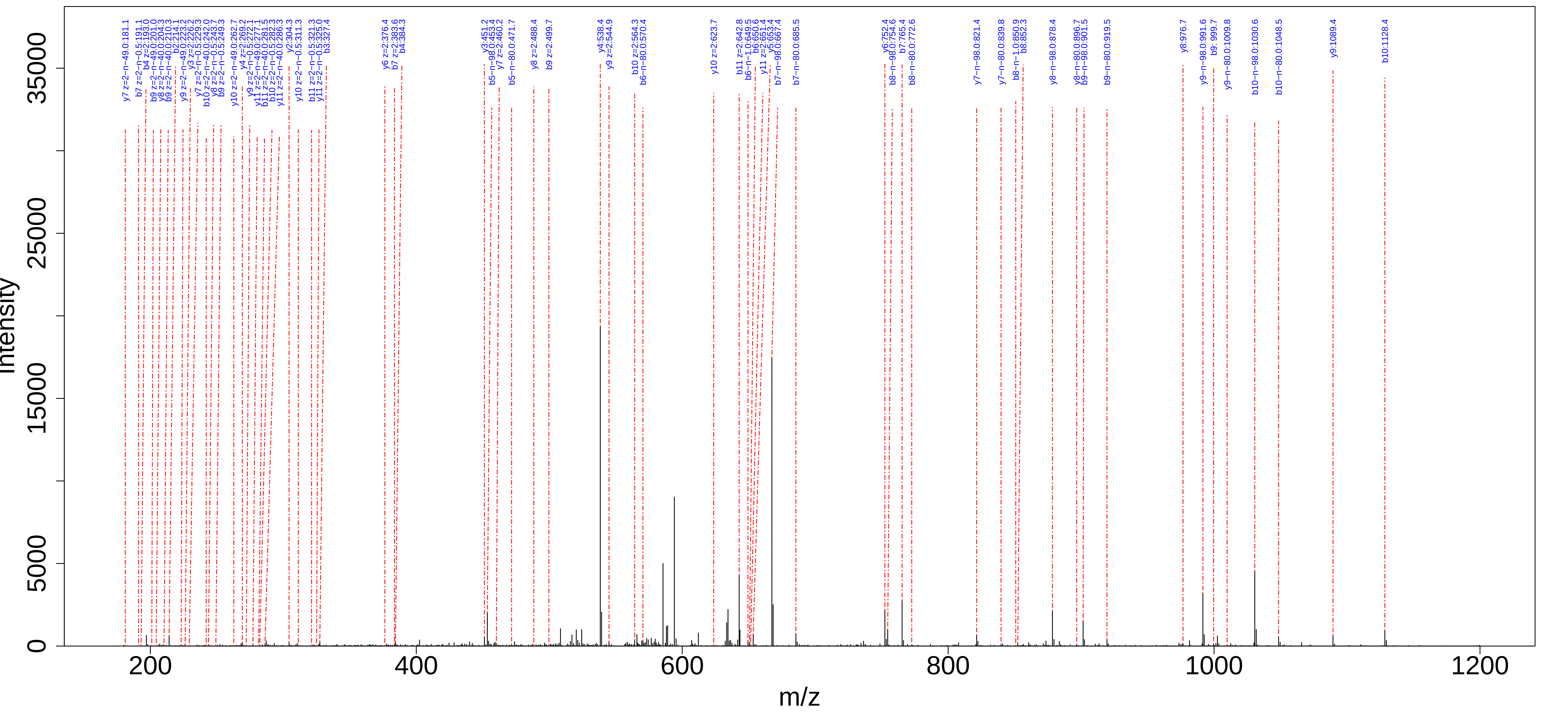

QSSSEQCSNLSSVR 3−S_Phospho z=+2 Raw scan=1579

TBCD4_HUMAN S681;S682; ; Q= 0.00940279542566709 ; score= 32.6 ; E= 0.0034 m/z=824.8359

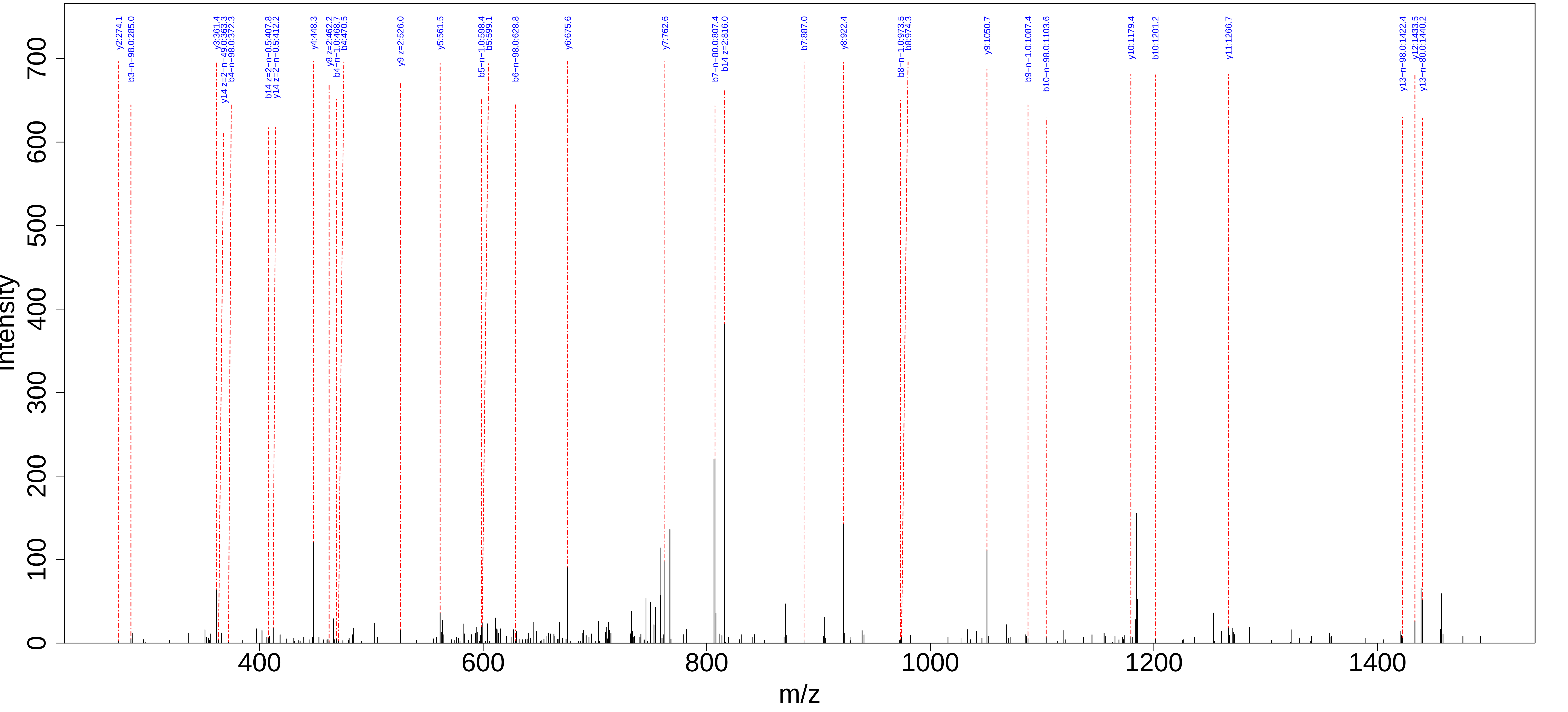

CVPAPGAGASGGTSPSATQPNPAVFIFEHK 14−S_Phospho z=+3 Raw scan=3908
TBCD4_HUMAN S102; ; Q= 0.00916522169928164 ; score= 31.9 ; E= 0.0025 m/z=1011.4715

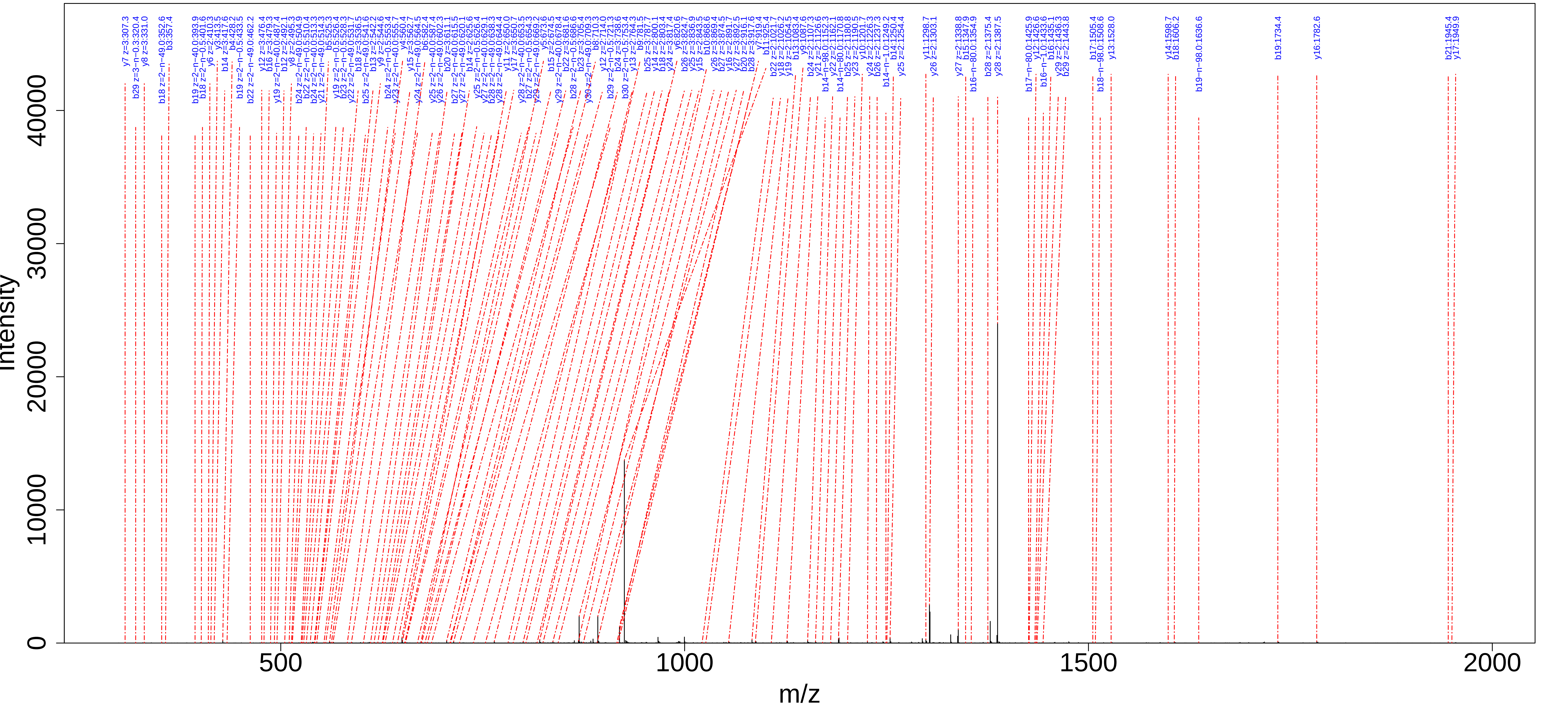

GRLGSVDSFER 5−S_Phospho z=+2 Raw scan=2414
TBCD4_HUMAN S591; ; Q= 0.0095331214861892 ; score= 31.5 ; E= 0.052 m/z=651.7969

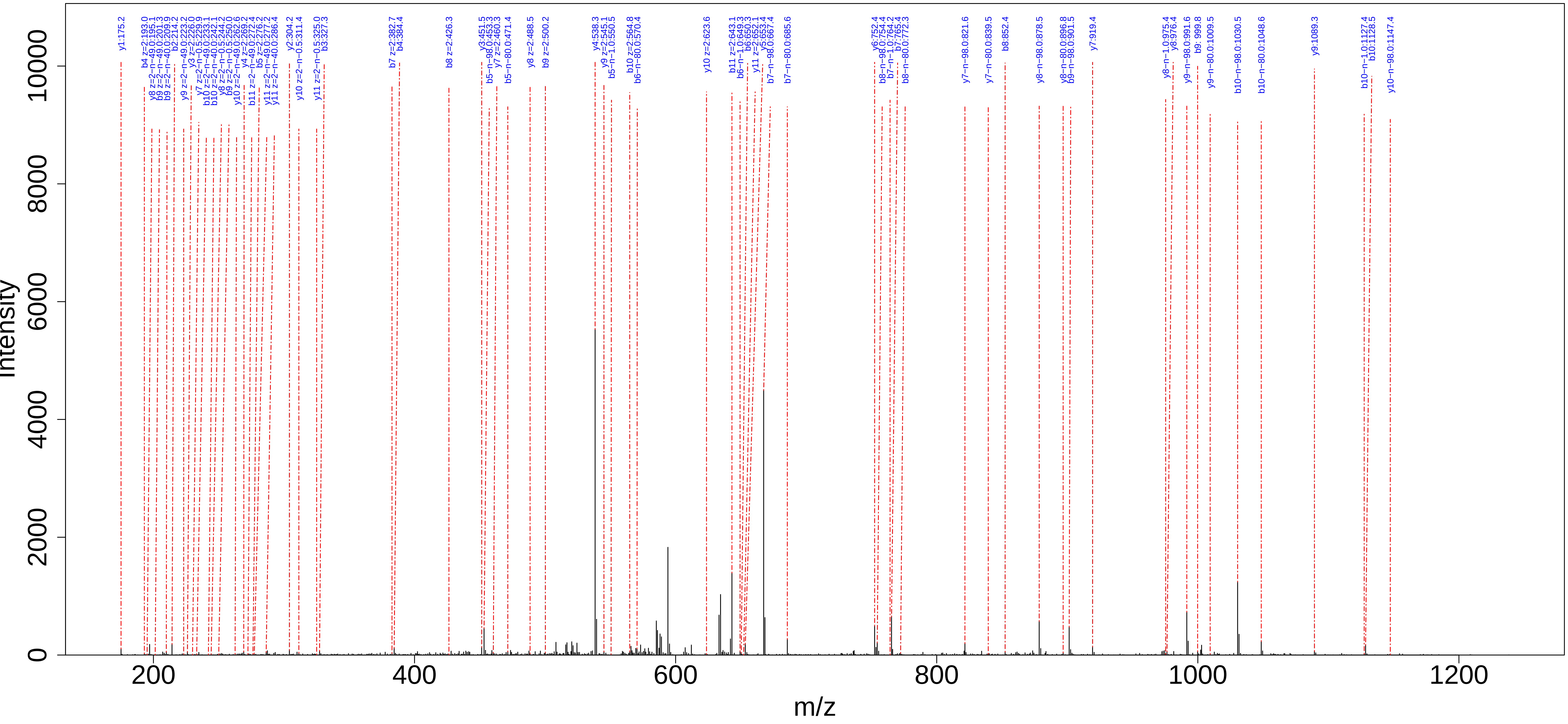

LGSVDSFER 3−S_Phospho z=+2 Raw scan=2566
TBCD4_HUMAN S588; ; Q= 0.00988904968644477 ; score= 31 ; E= 0.028 m/z=545.2343

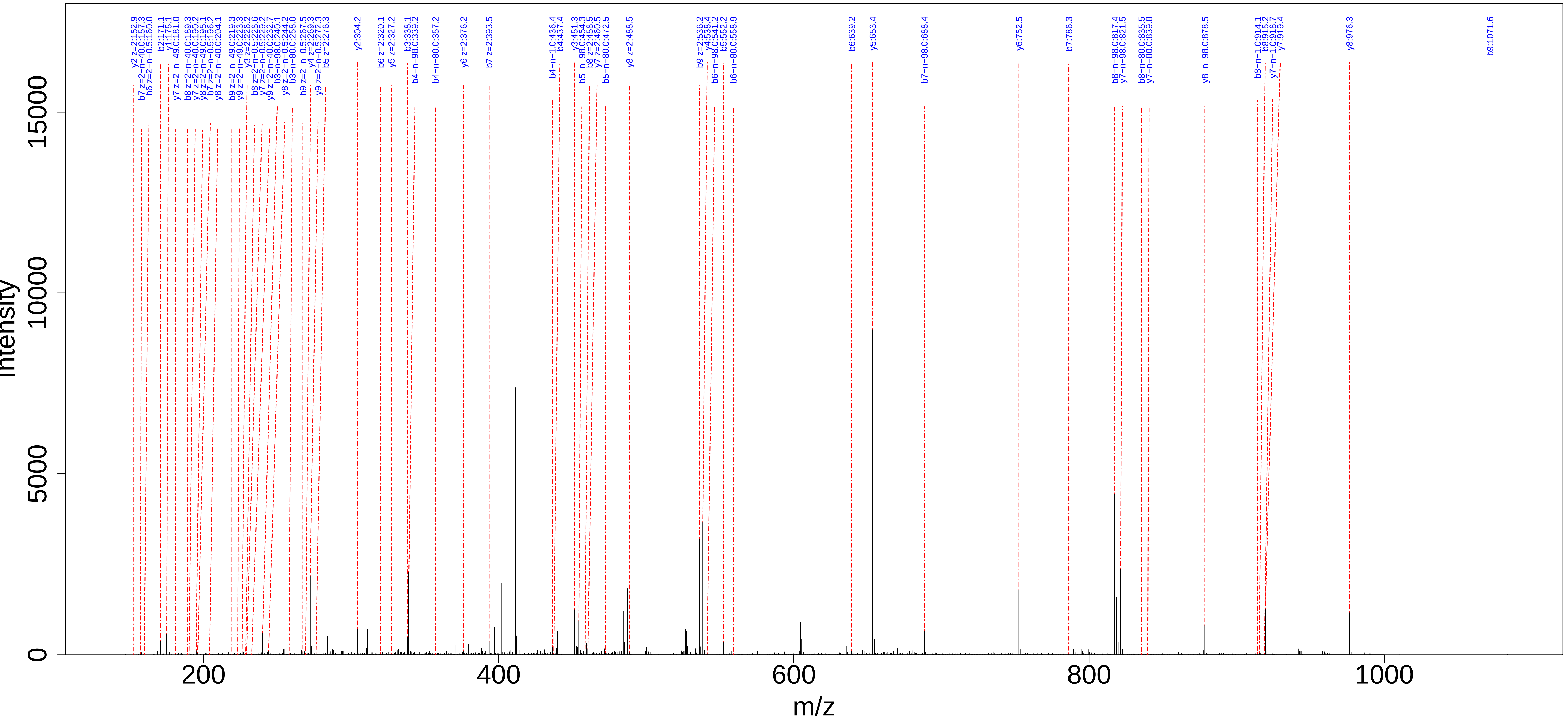

CVPAPGAGASGGTSPSATQPNPAVFIFEHK 14−S_Phospho z=+3 Raw scan=3915
TBCD4_HUMAN S102; ; Q= 0.0124267291910903 ; score= 28.5 ; E= 0.012 m/z=1011.4741

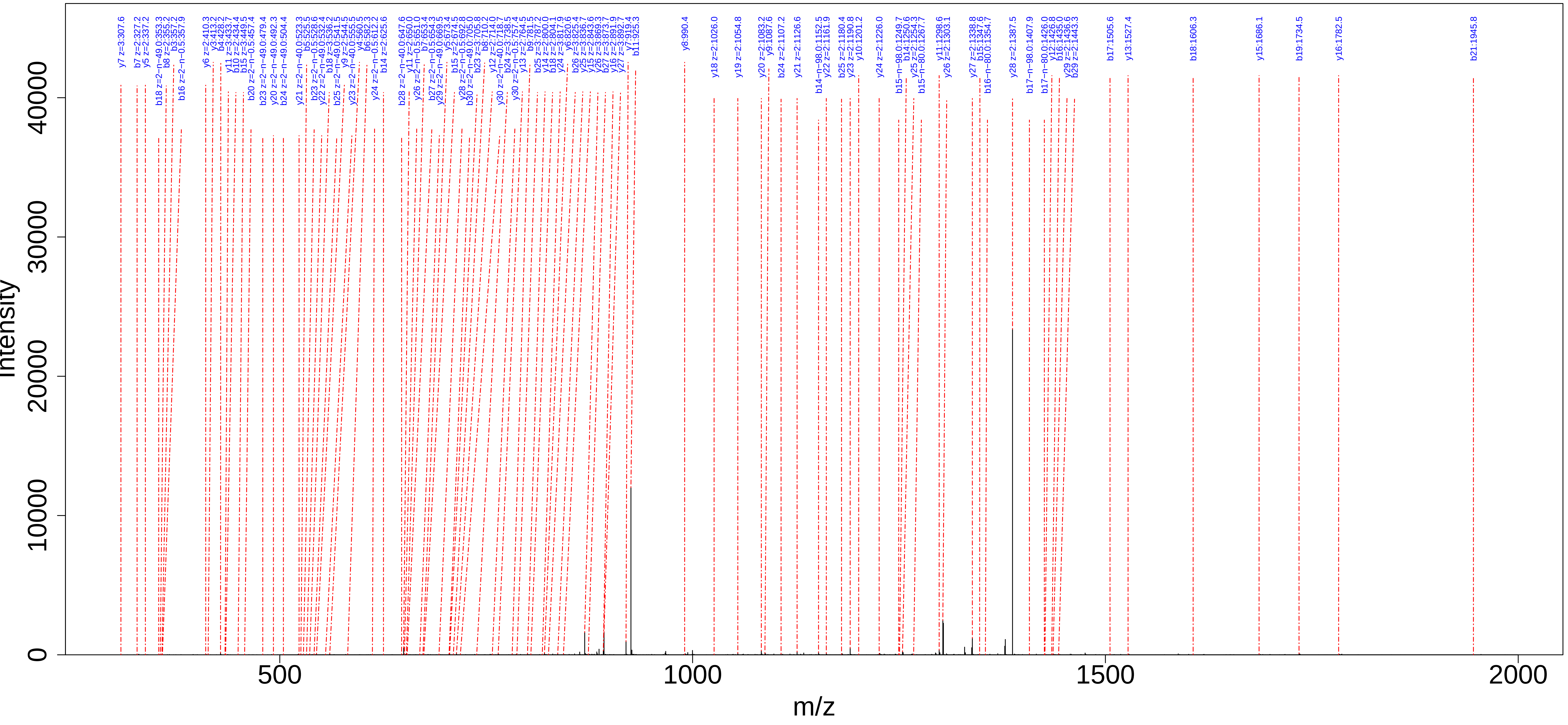

QSSSEQCSNLSSVR 2−S_Phospho z=+2 Raw scan=1602
TBCD4_HUMAN S682; ; Q= 0.0174913693901036 ; score= 24.6 ; E= 0.08 m/z=824.8347

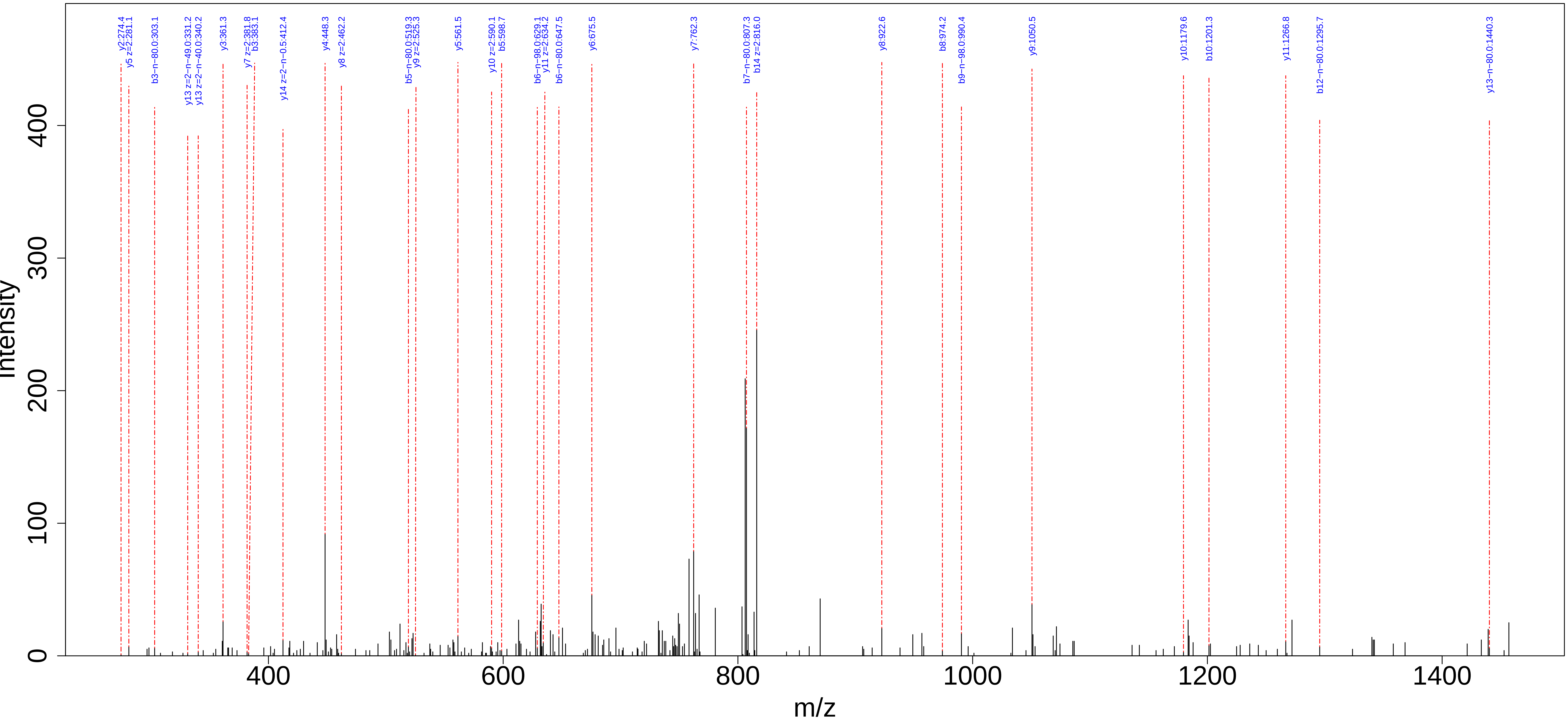

QSSSEQCSNLSSVR 3−S_Phospho z=+2 Raw scan=1602
TBCD4_HUMAN S682; ; Q= 0.0174873446847676 ; score= 24.6 ; E= 0.08 m/z=824.8347

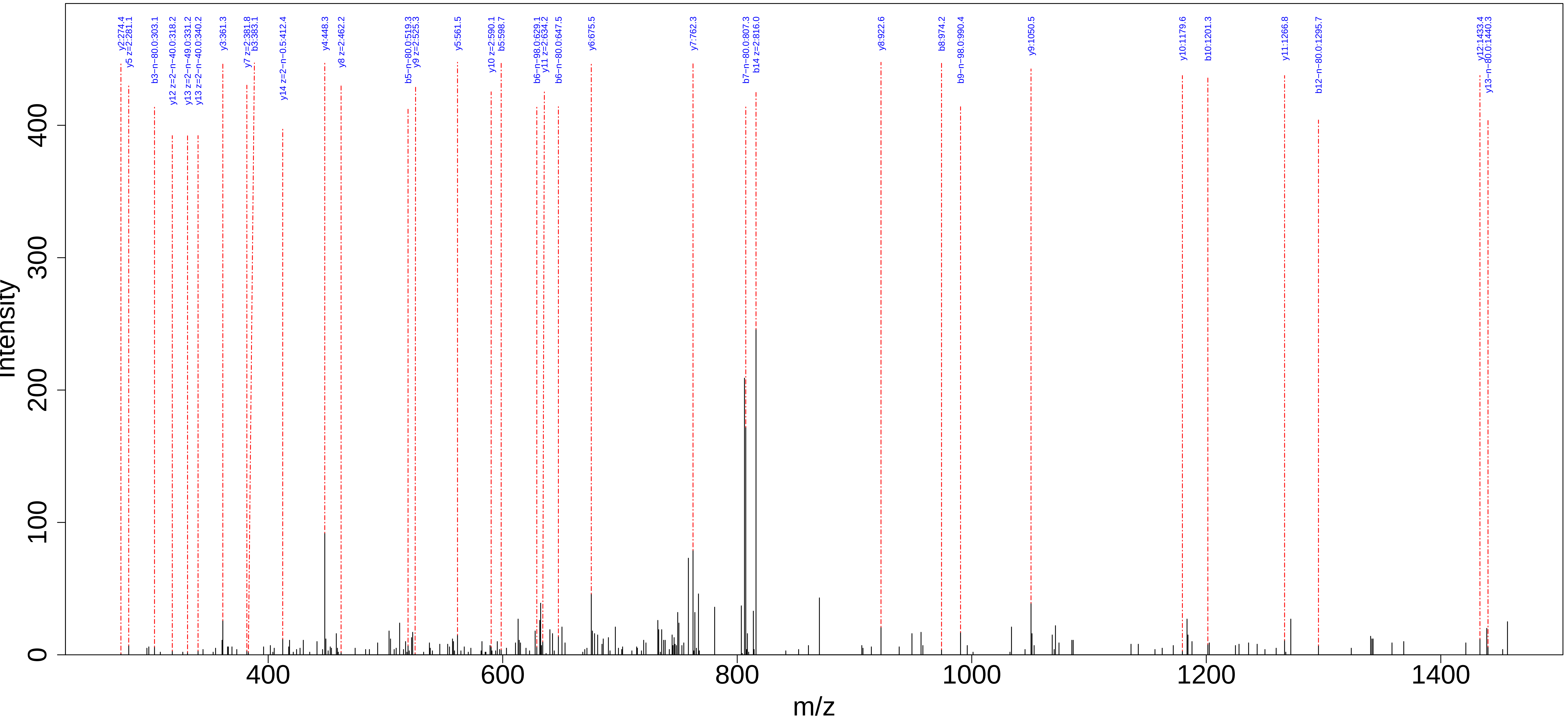

QSSSEQCSNLSSVR 4−S_Phospho z=+2 Raw scan=1602
TBCD4_HUMAN S682; ; Q= 0.0174833218311479 ; score= 24.6 ; E= 0.08 m/z=824.8347

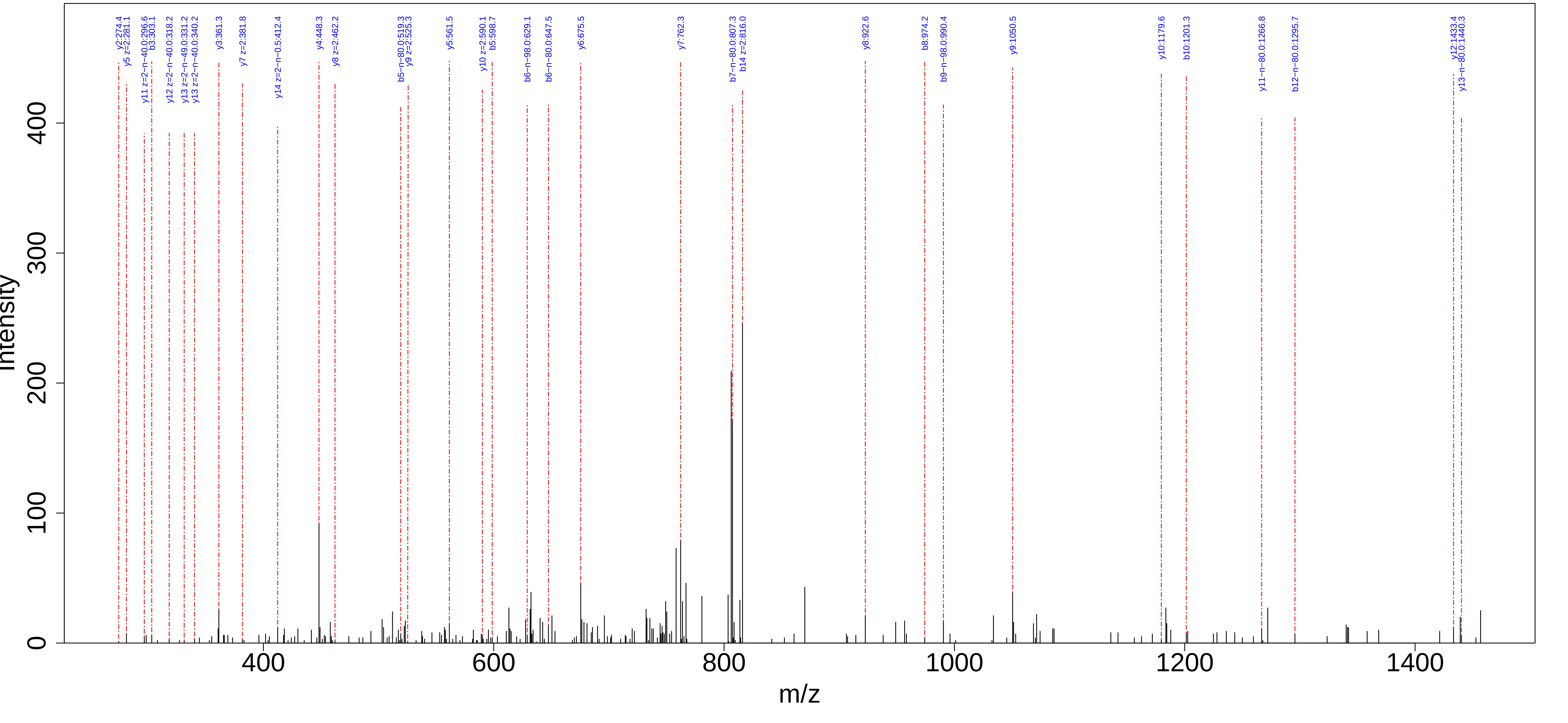

annotated raw MSMS spectra for assay:

Protein kinase: SGK3

Substrate: TBC1D1

sample name: TBCD1_SGK3_S7

ANTLSHFPIECQEPPQPAR 3−T_Phospho z=+3 Raw scan=2660
TBCD1_HUMAN T596; ; Q= 0 ; score= 51.6 ; E= 0.00012 m/z=758.0125

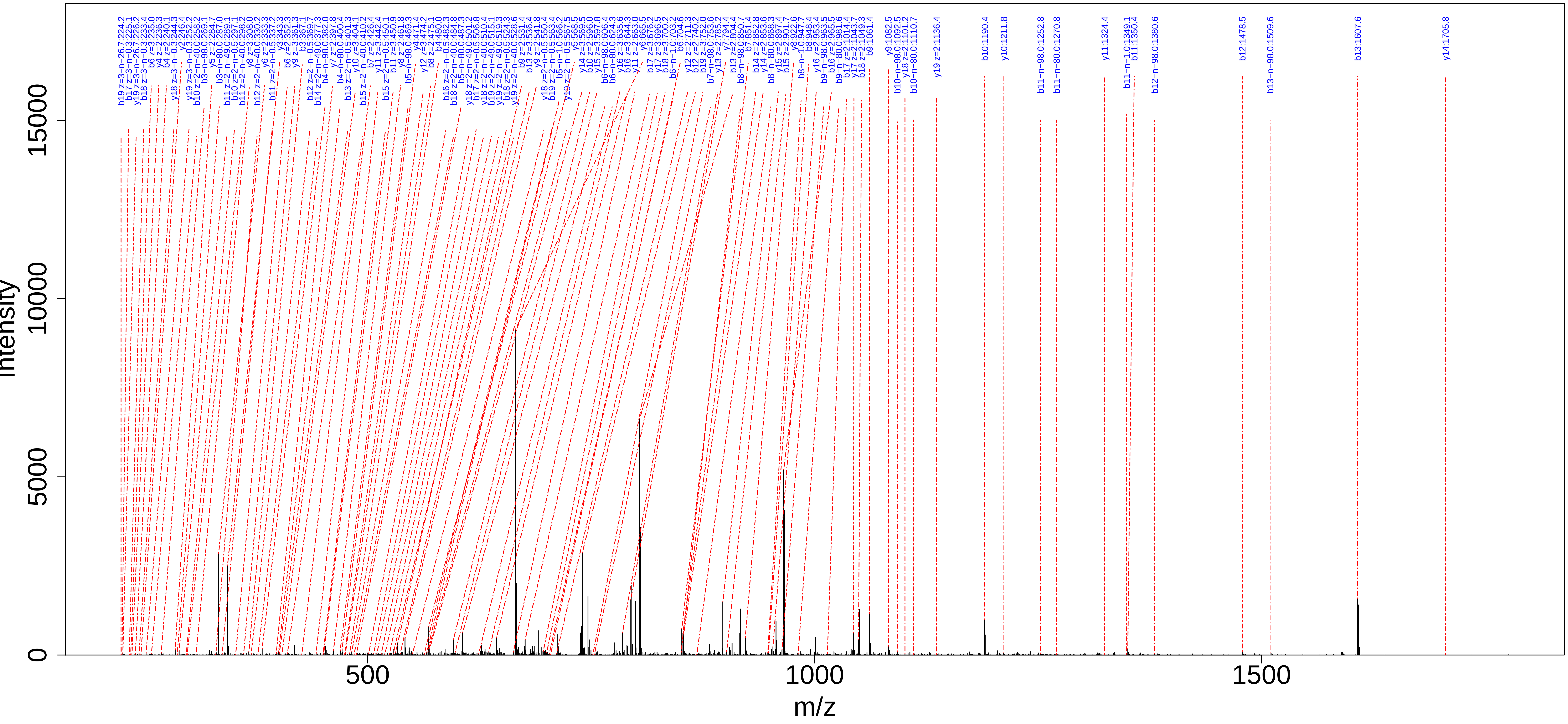

RANTLSHFPIECQEPPQPAR 4−T_Phospho z=+3 Raw scan=2395
TBCD1_HUMAN T596; ; Q= 0.0159730979403111 ; score= 37.3 ; E= 0.051 m/z=810.0451

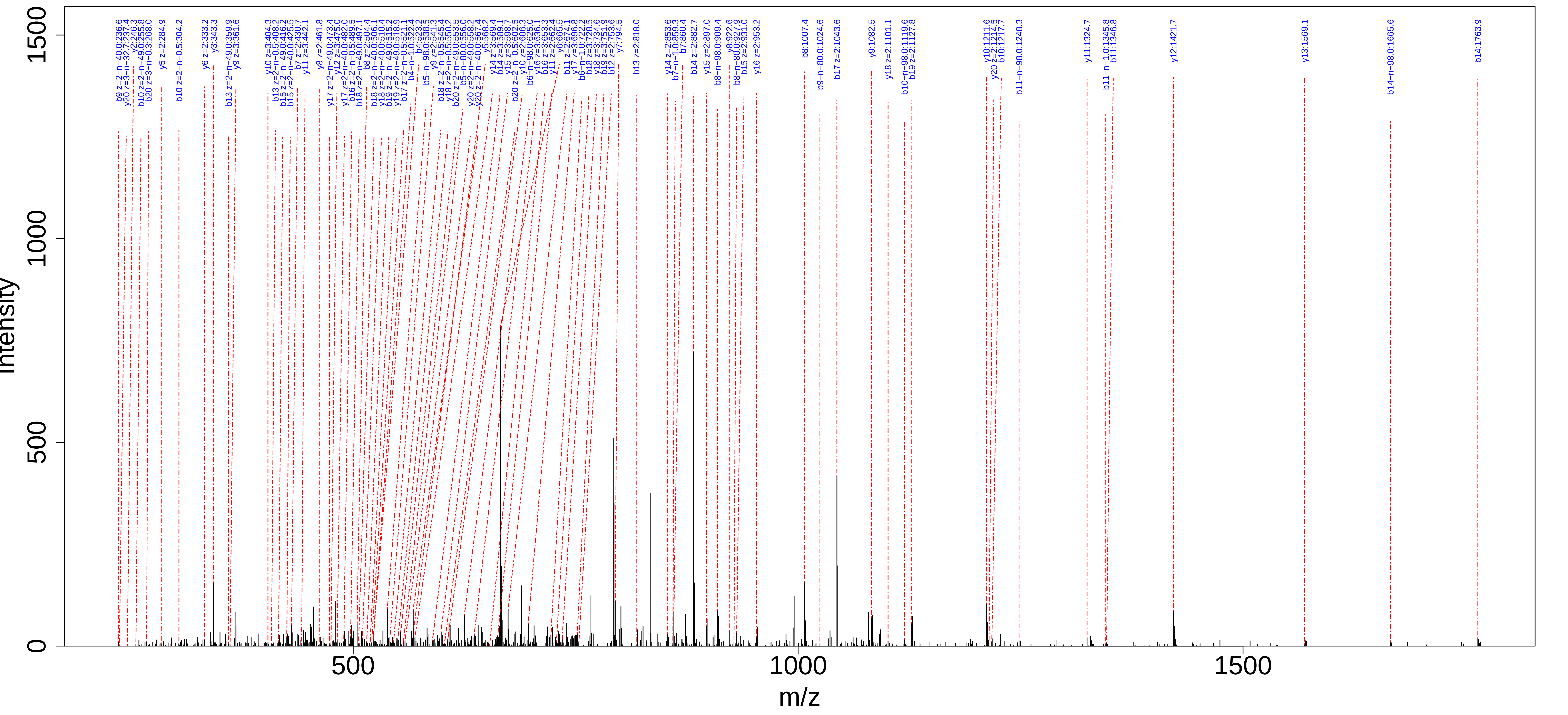

RANTLSHFPIECQEPPQPAR 6−S_Phospho z=+3 Raw scan=2395
TBCD1_HUMAN T596; ; Q= 0.0159663865546218 ; score= 37.3 ; E= 0.051 m/z=810.0451

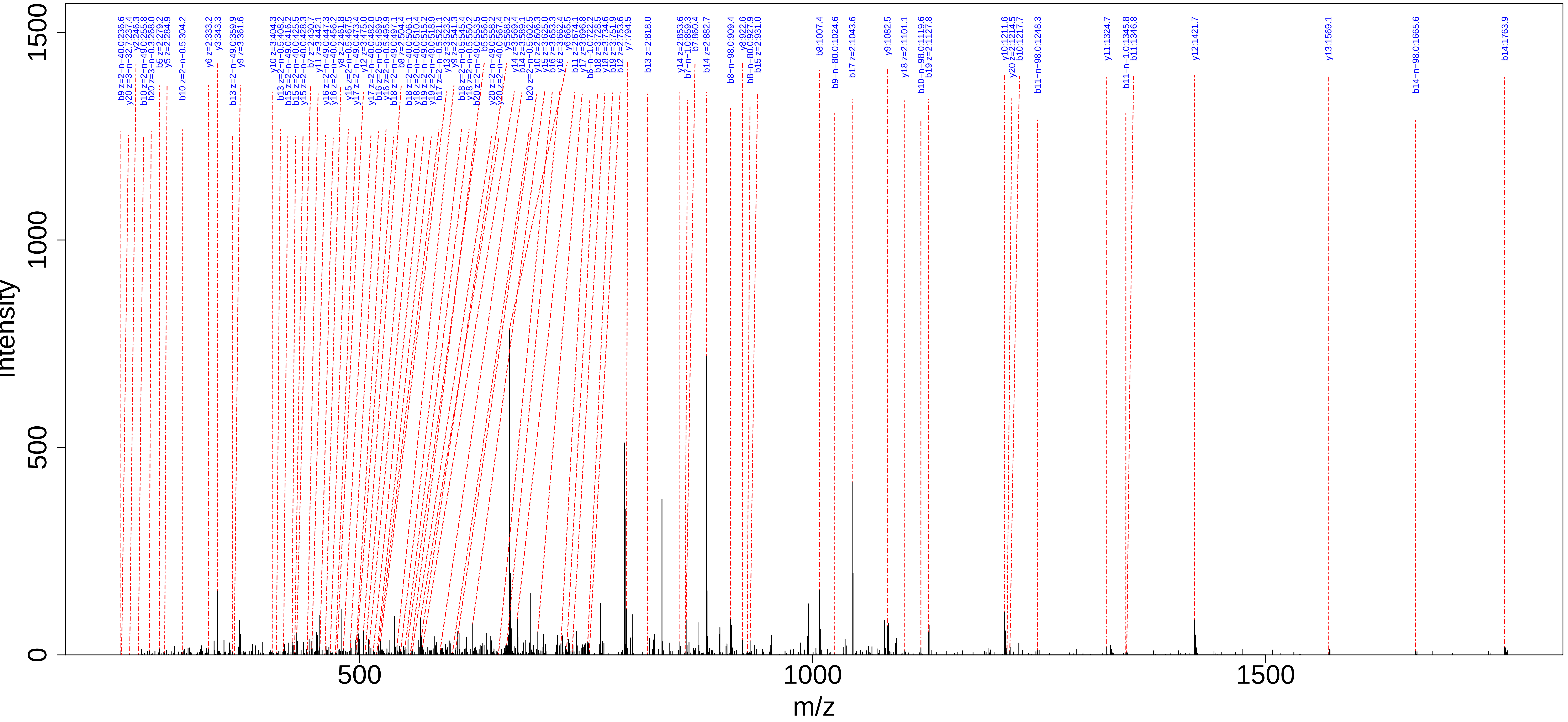

RANTLSHFPIECQEPPQPAR 4−T_Phospho z=+3 Raw scan=2385
TBCD1_HUMAN T596; ; Q= 0.0159128978224456 ; score= 37.2 ; E= 0.094 m/z=810.0463

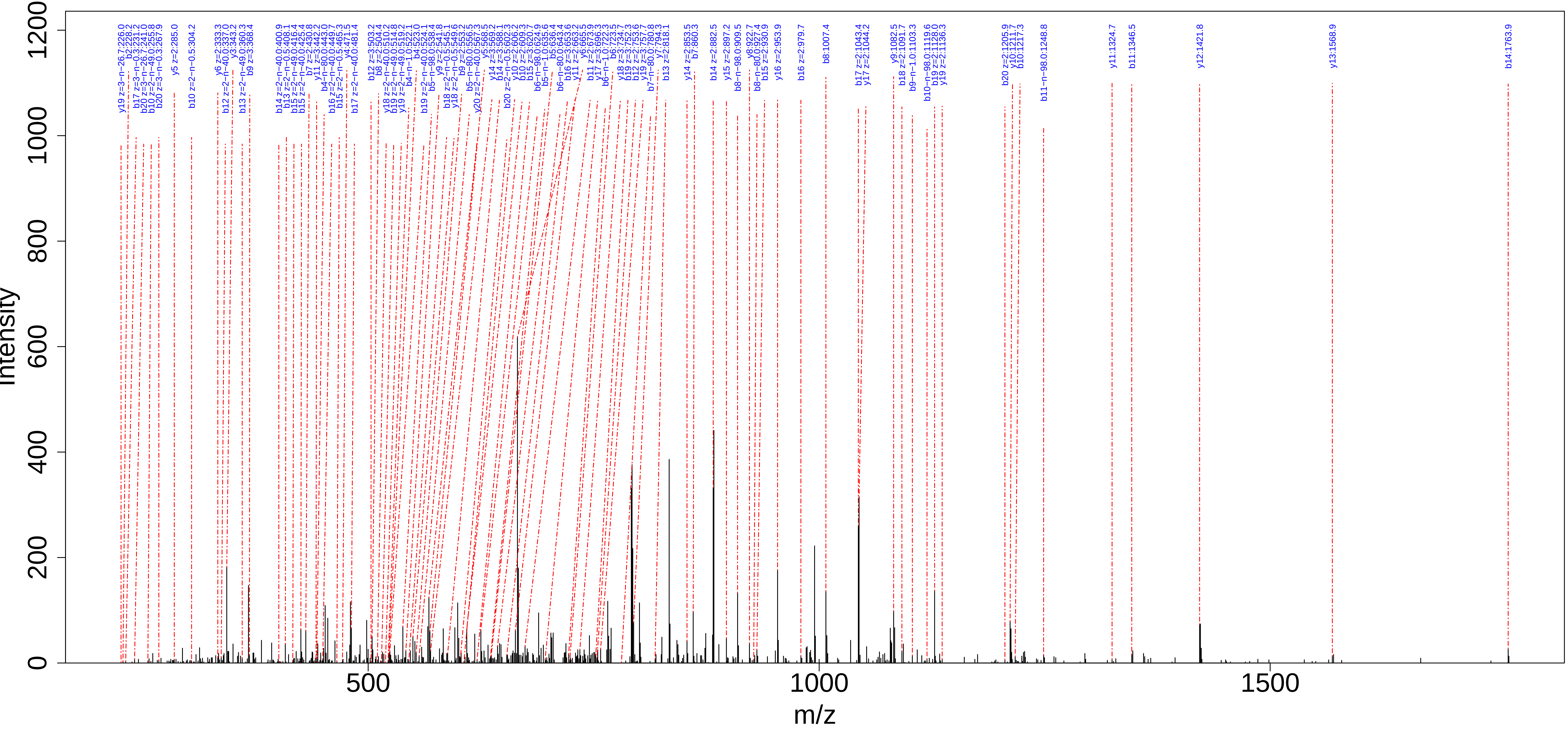

annotated raw MSMS spectra for assay:

Protein kinase: SGK3

Substrate: TBC1D4

sample name: TBCD4_SGK3_S8

ESNSSSSLPSLHTSFSAPSFTAPSFLK 16−S_Phospho z=+3 Raw scan=4246
TBCD4_HUMAN S712; ; Q= 0 ; score= 57.8 ; E= 4.9e−06 m/z=965.1166

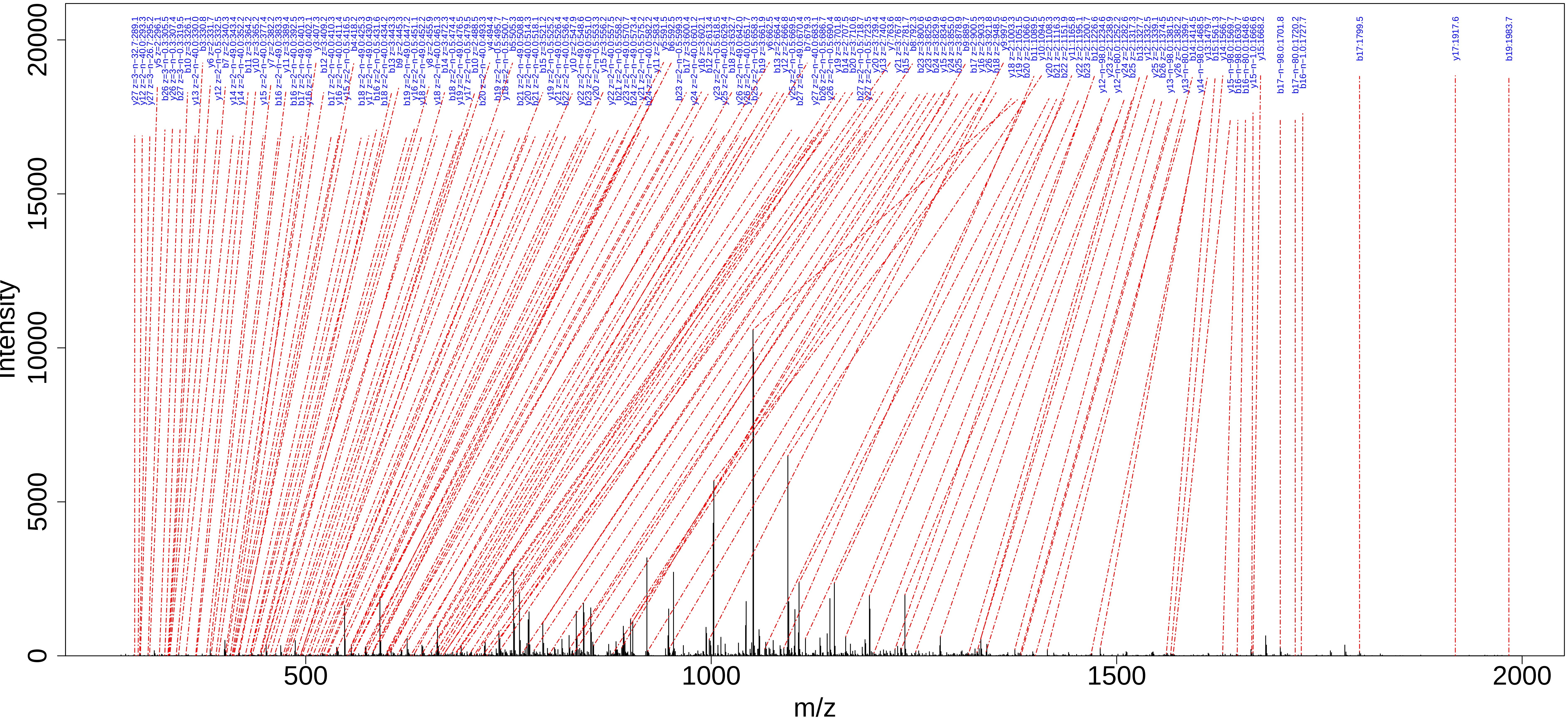

SLTSSLENIFSR 1−S_Phospho z=+2 Raw scan=4635
TBCD4_HUMAN S576; ; Q= 0 ; score= 55.9 ; E= 2.7e−07 m/z=717.3397

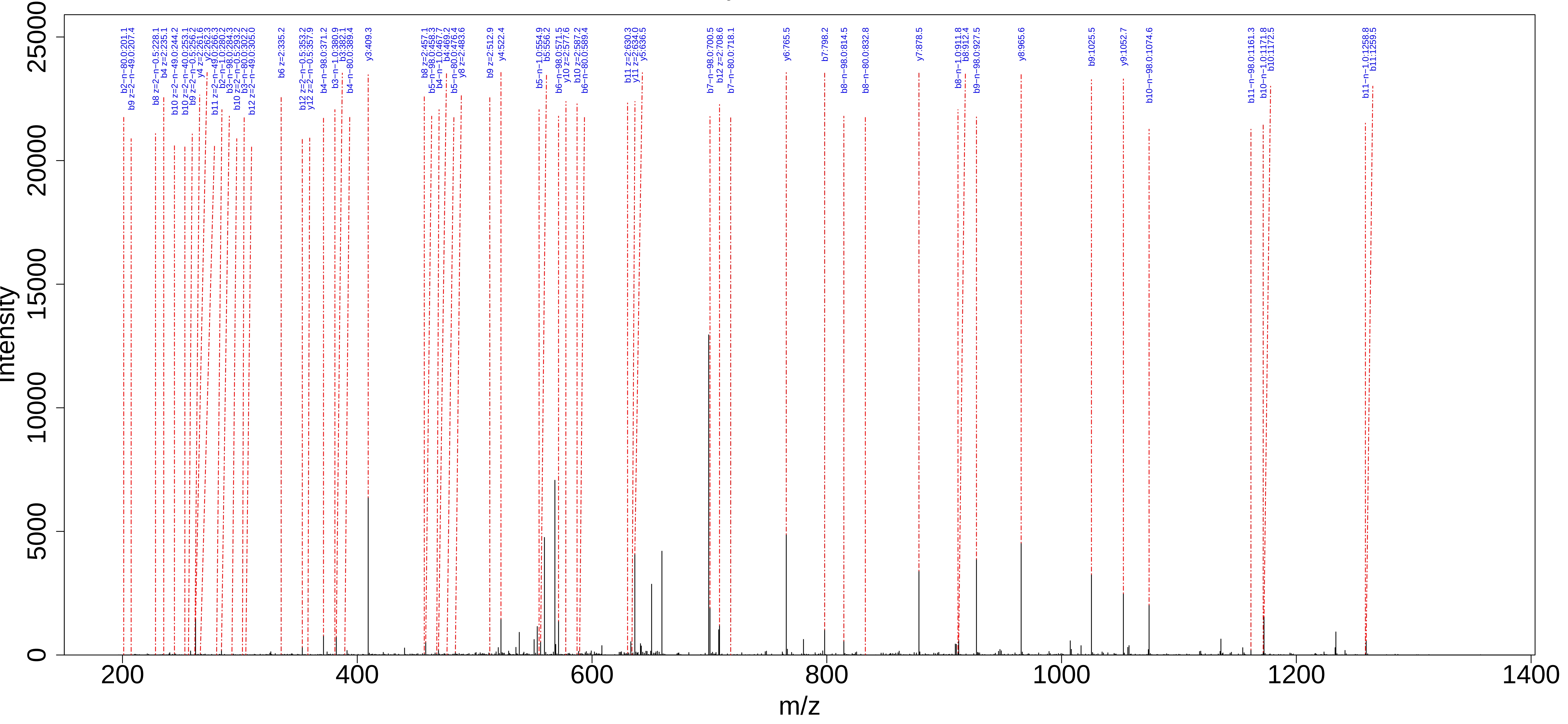

ESNSSSSLPSLHTSFSAPSFTAPSFLK 16−S_Phospho z=+3 Raw scan=4237
TBCD4_HUMAN S702; ; Q= 0 ; score= 55.9 ; E= 5.7e−05 m/z=965.1151

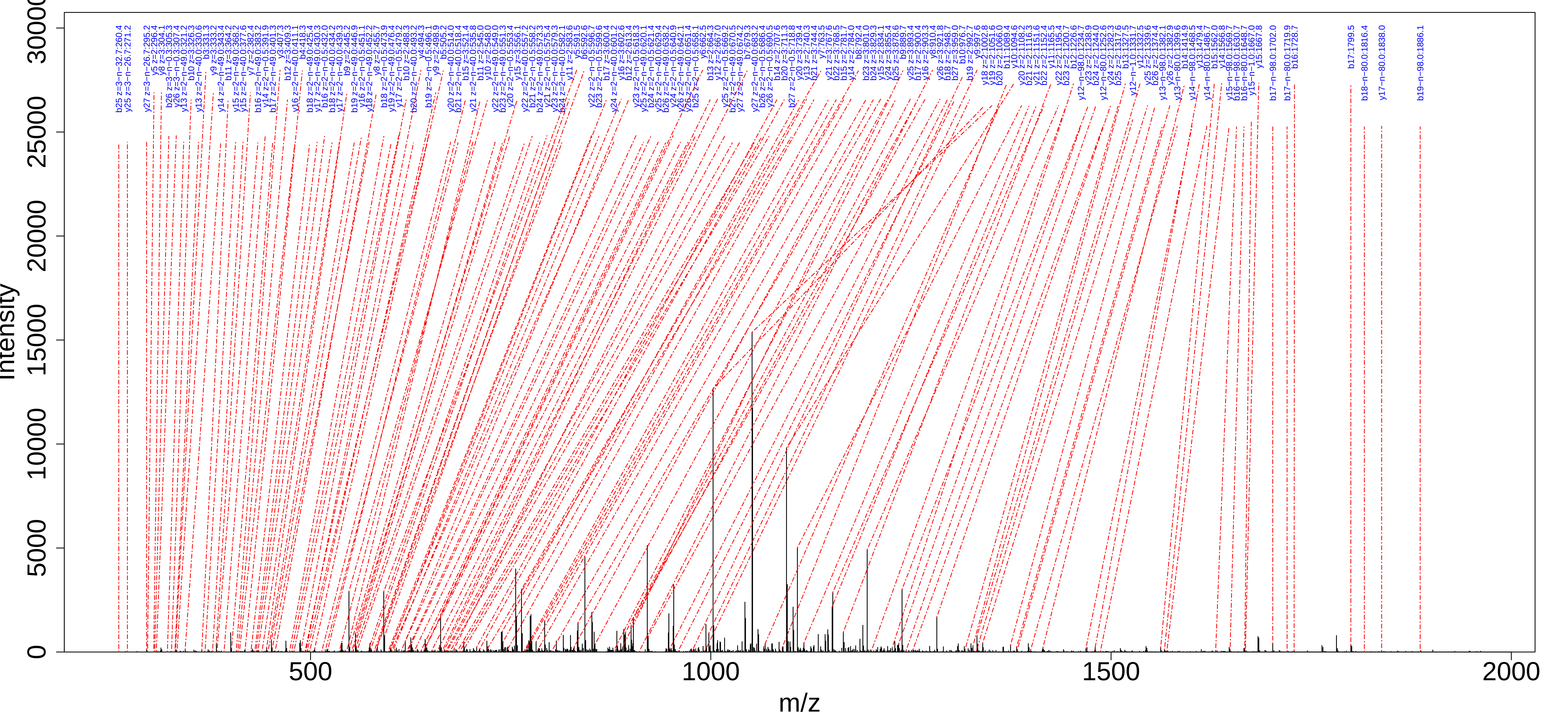

ESNSSSSLPSLHTSFSAPSFTAPSFLK 16−S_Phospho z=+3 Raw scan=4232

TBCD4_HUMAN S692; ; Q= 0 ; score= 52.6 ; E= 0.00018 m/z=965.1174

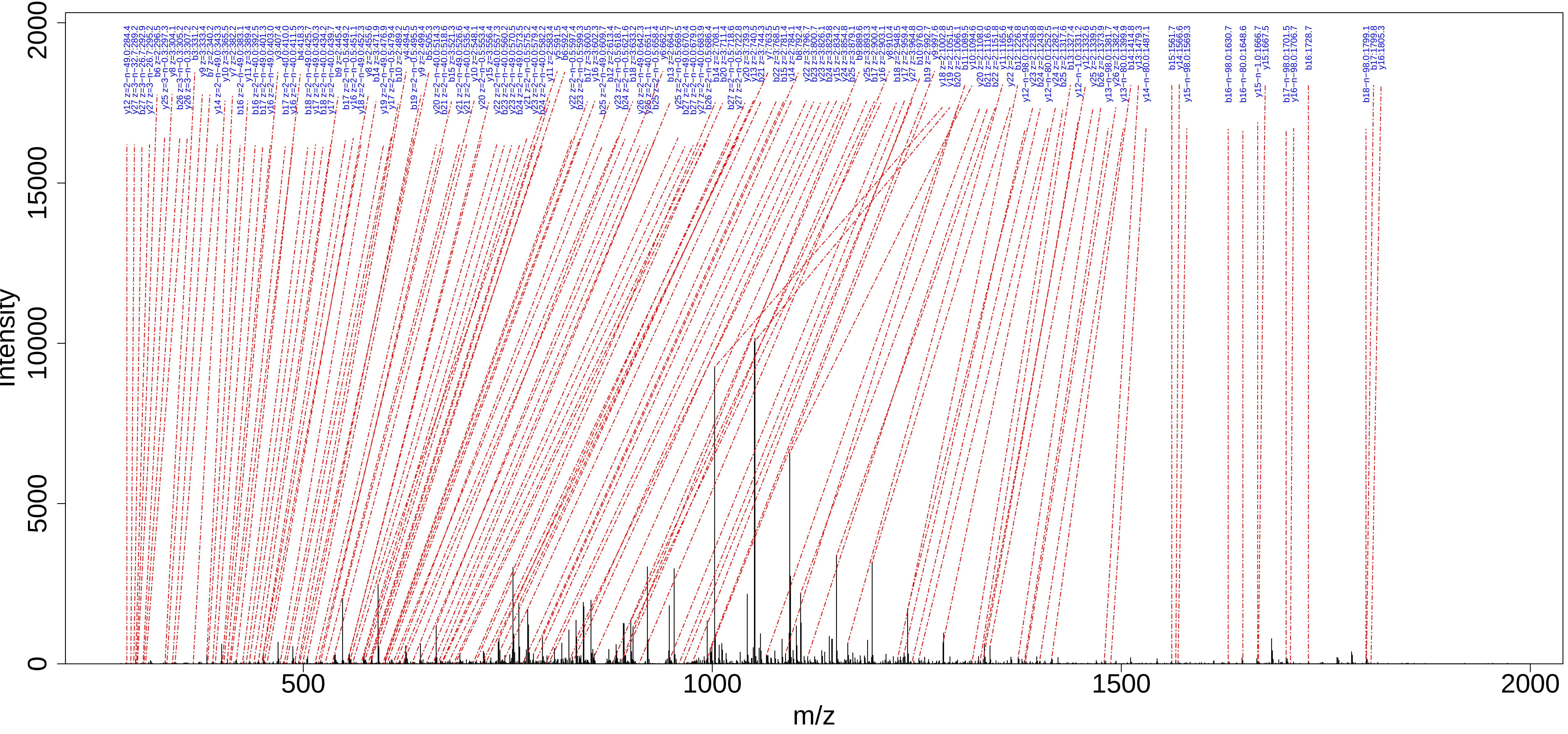

SLTSSLENIFSR 3−T_Phospho z=+2 Raw scan=4629
TBCD4_HUMAN T568; ; Q= 0 ; score= 51.6 ; E= 6e−05 m/z=717.338

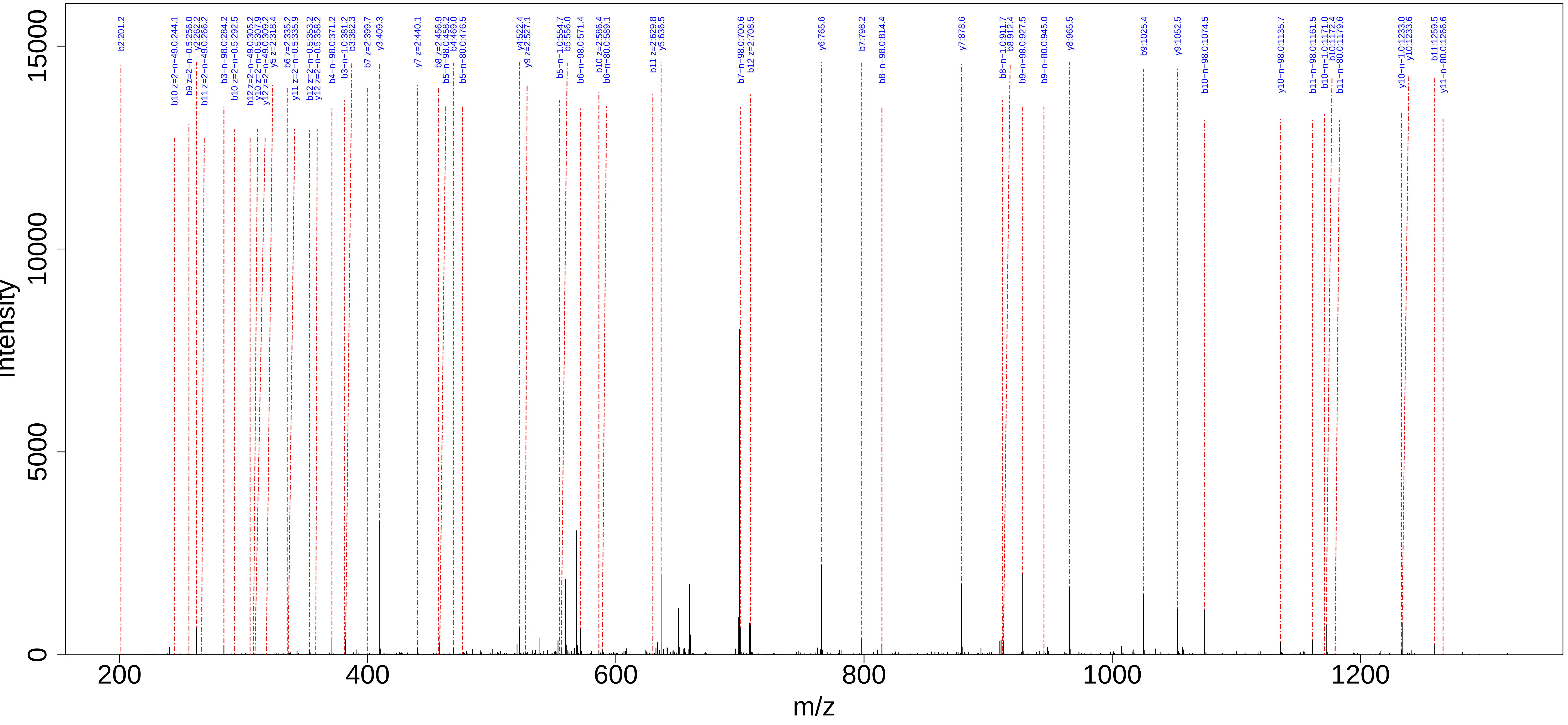

SLTSSLENIFSR 3−T_Phospho z=+2 Raw scan=4644
TBCD4_HUMAN S569; ; Q= 0 ; score= 48.6 ; E= 3.7e−05 m/z=717.3389

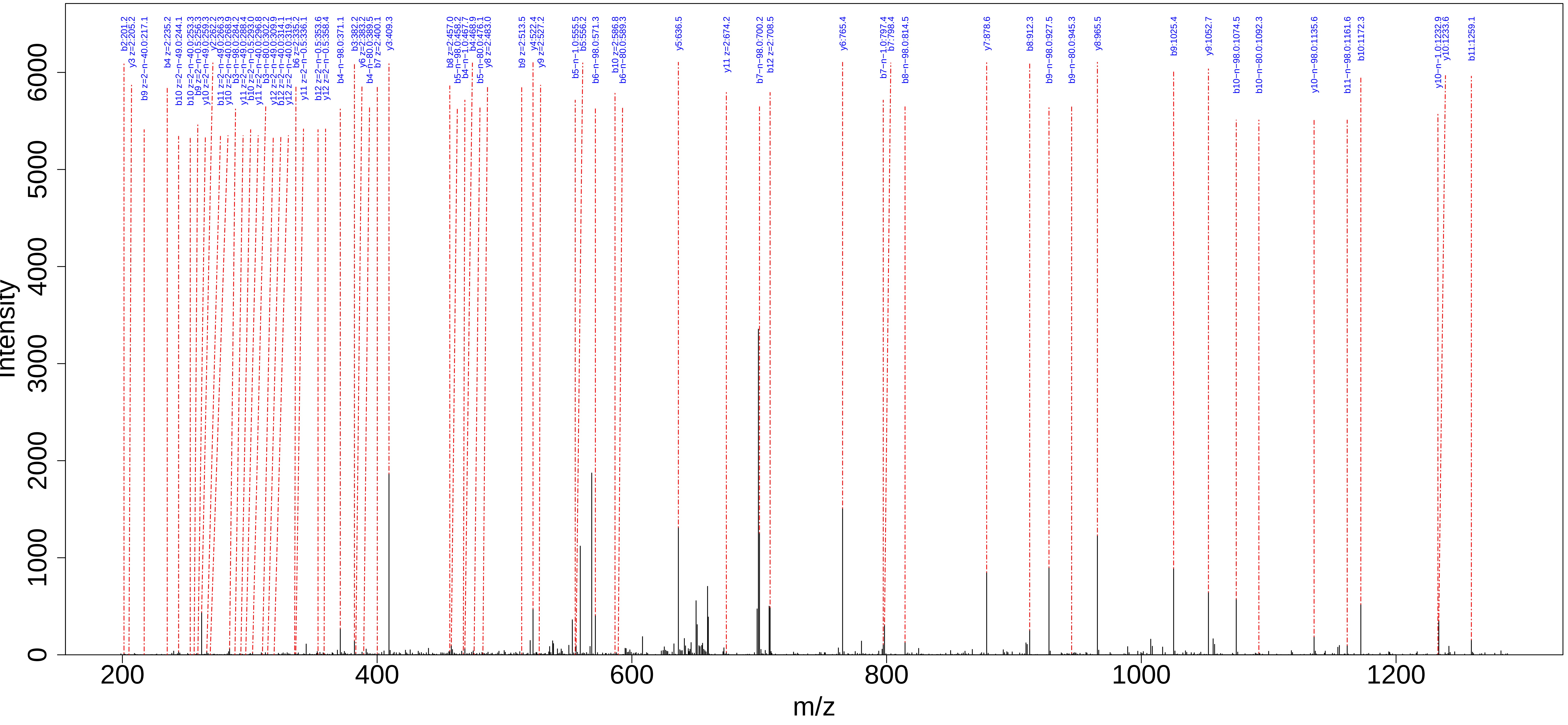

SFYQNSGRLSPQYENEIR 10−S_Phospho z=+3 Raw scan=2662
TBCD4_HUMAN S725; ; Q= 0 ; score= 47.4 ; E= 0.00025 m/z=756.674

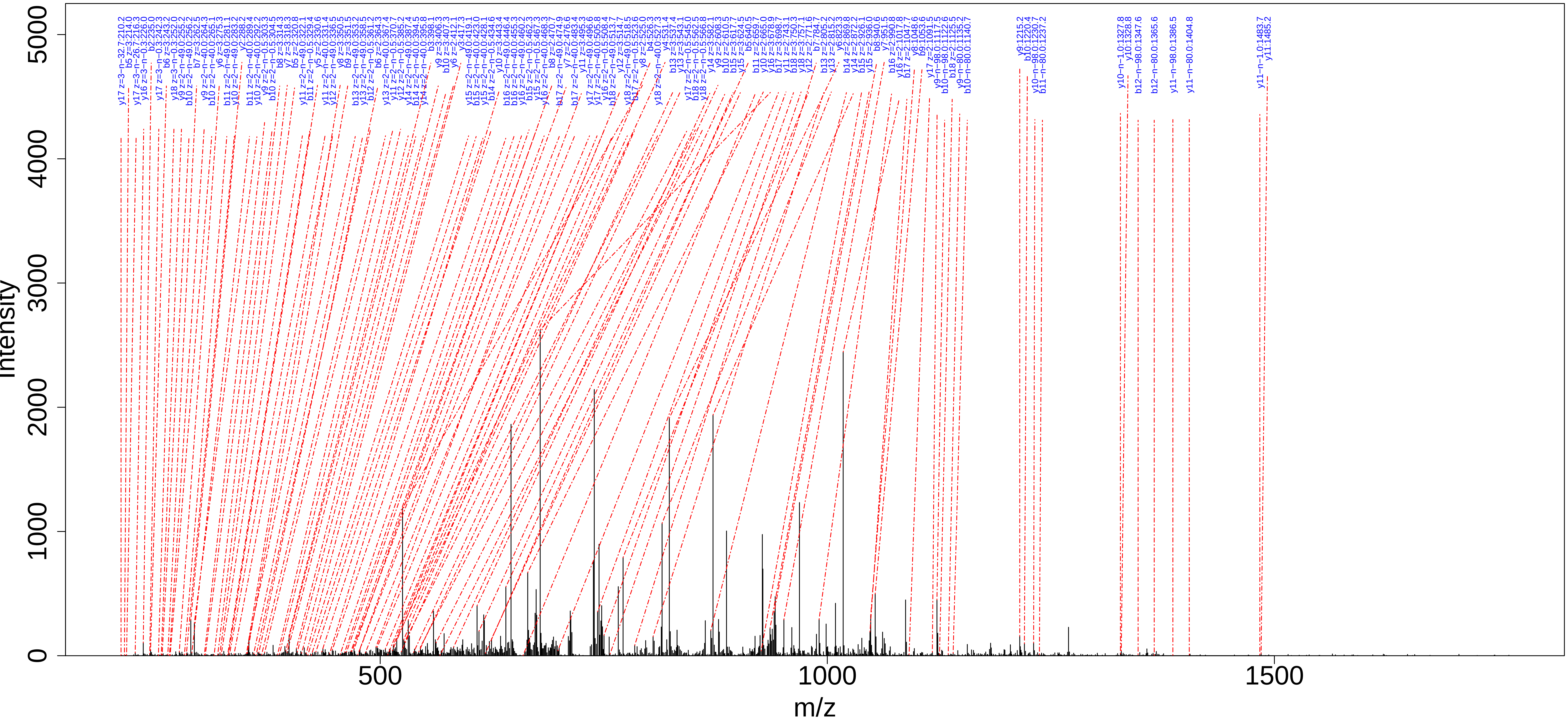

SFYQNSGRLSPQYENEIR 10−S_Phospho z=+3 Raw scan=2656
TBCD4_HUMAN S721; ; Q= 0 ; score= 47.3 ; E= 0.00029 m/z=756.6744

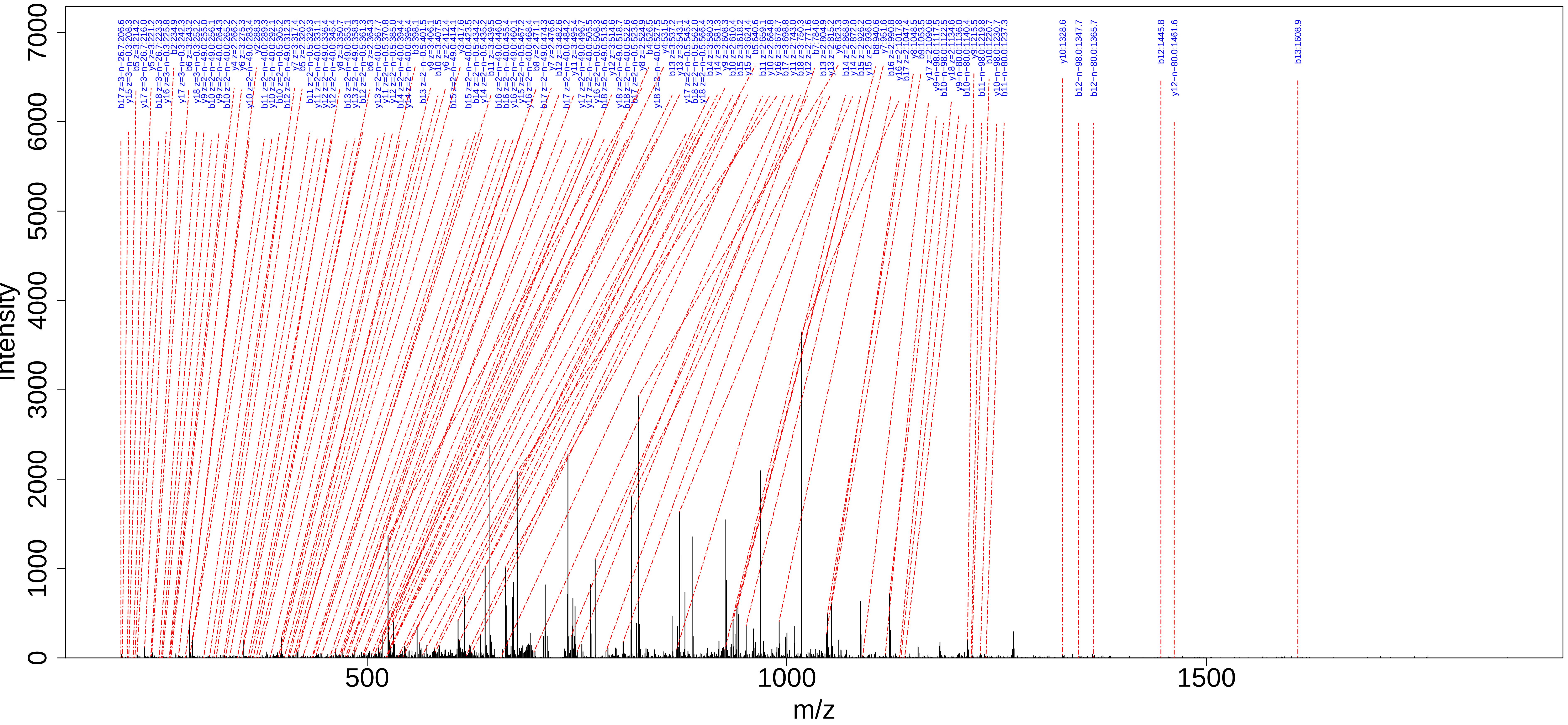

ESNSSSSLPSLHTSFSAPSFTAPSFLK 13−T_Phospho z=+3 Raw scan=4268
TBCD4_HUMAN S692; ; Q= 0.00359550561797753 ; score= 45.1 ; E= 0.0069 m/z=965.1161

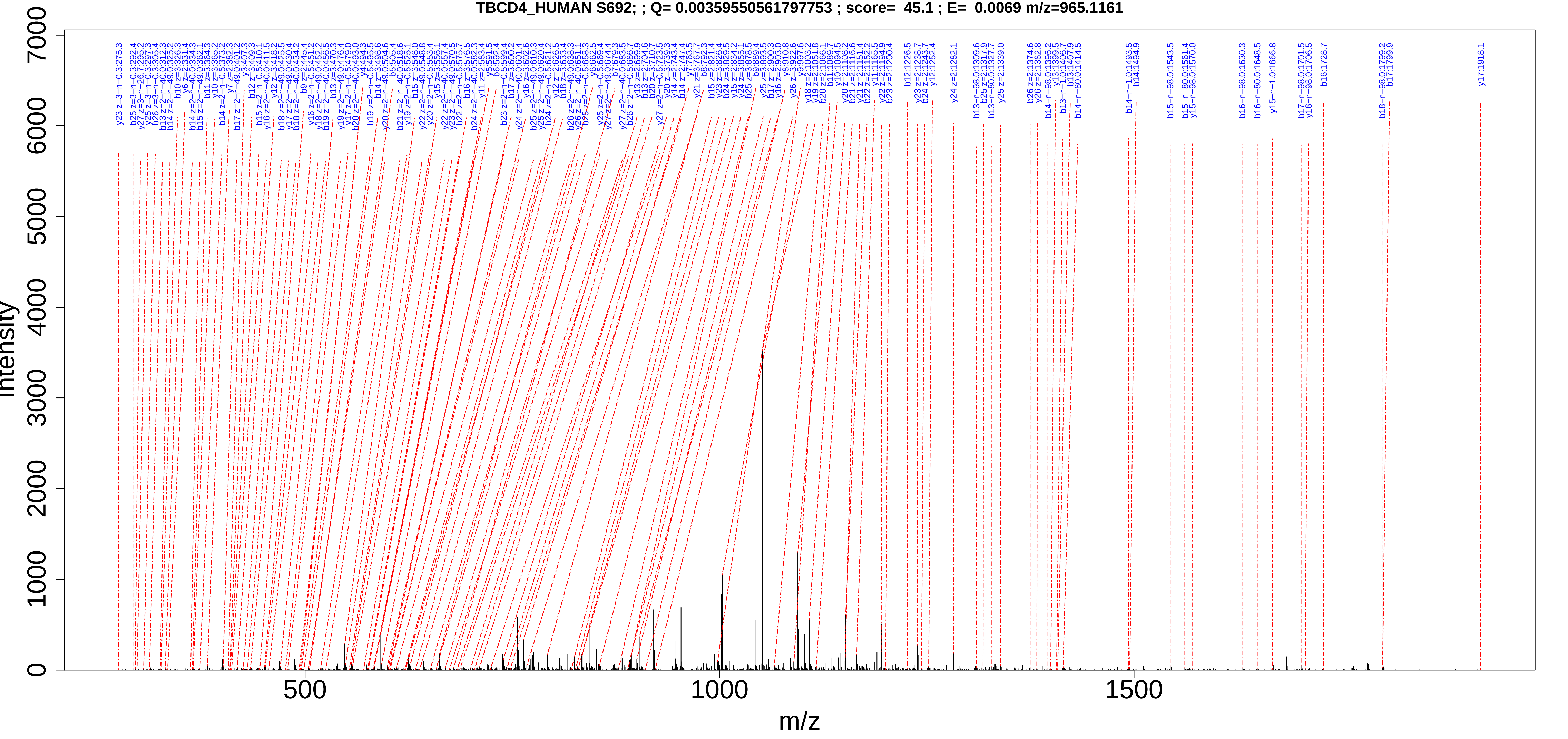

ESNSSSSLPSLHTSFSAPSFTAPSFLK 14−S_Phospho z=+3 Raw scan=4268
TBCD4_HUMAN S692; ; Q= 0.00359389038634322 ; score= 45.1 ; E= 0.0069 m/z=965.1161

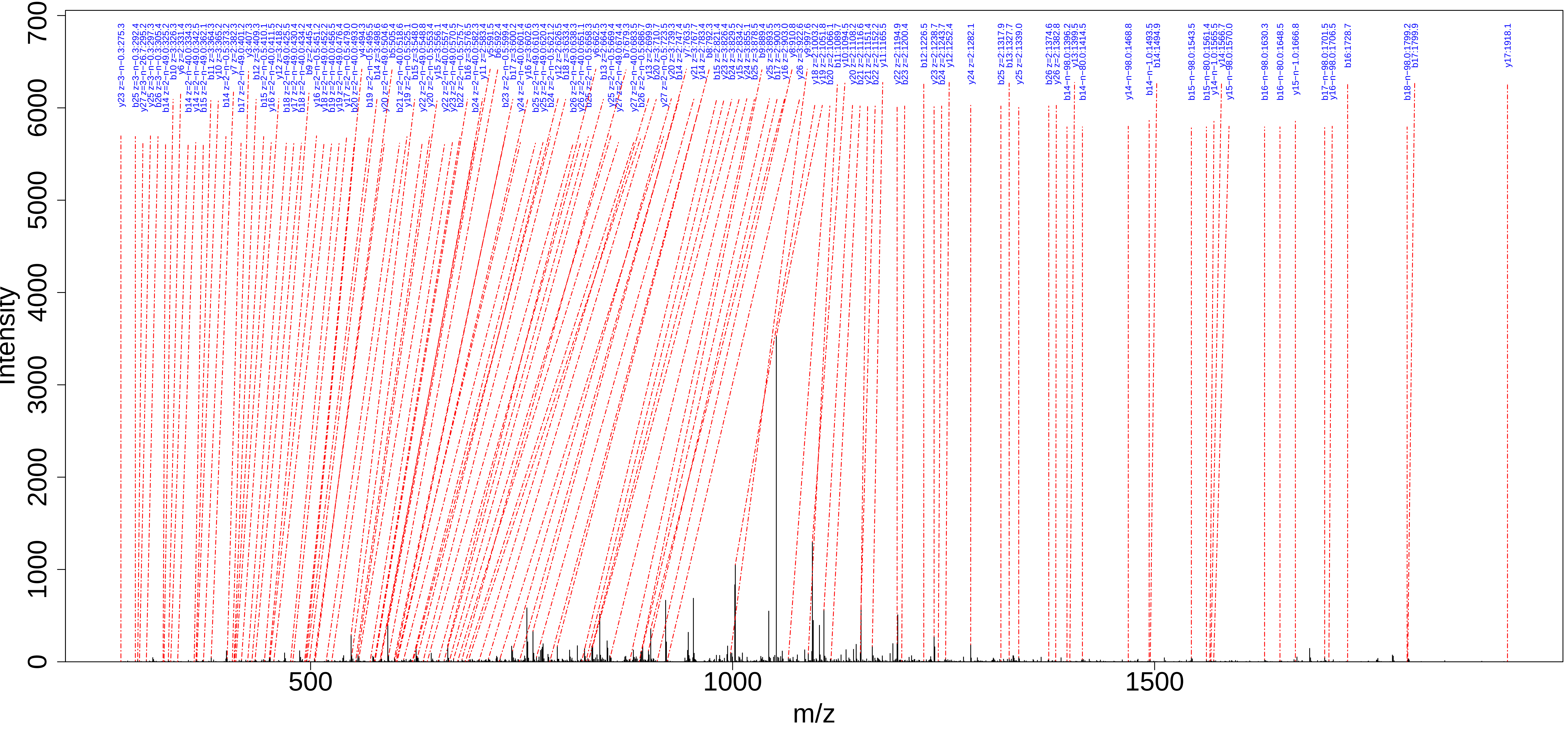

ESNSSSSLPSLHTSFSAPSFTAPSFLK 16−S_Phospho z=+3 Raw scan=4268
TBCD4_HUMAN S692; ; Q= 0.00359227660529861 ; score= 45.1 ; E= 0.0069 m/z=965.1161

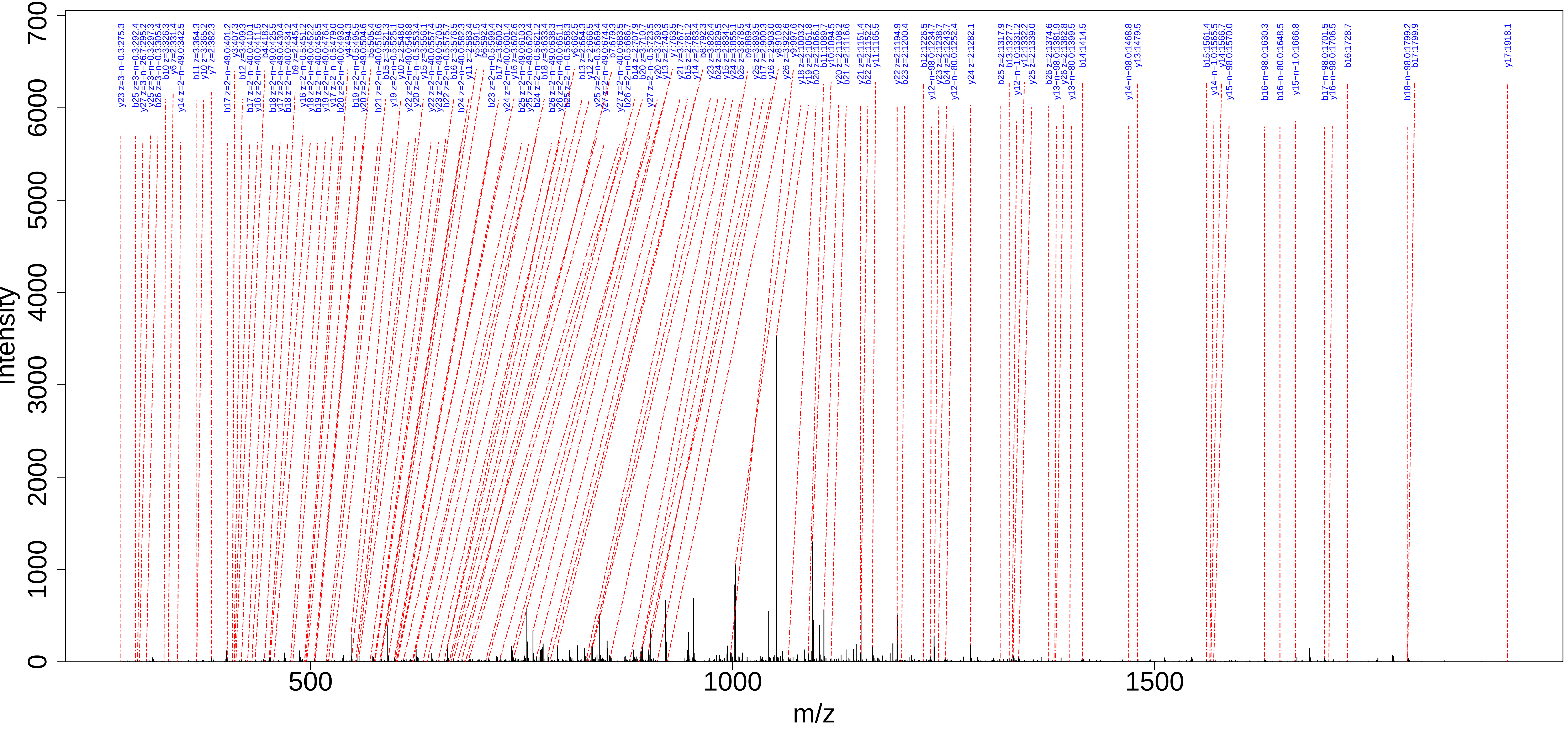

GRLGSVDSFER 5−S_Phospho z=+2 Raw scan=2310

TBCD4_HUMAN S591; ; Q= 0.00484066155707947 ; score= 41.8 ; E= 0.001 m/z=651.795

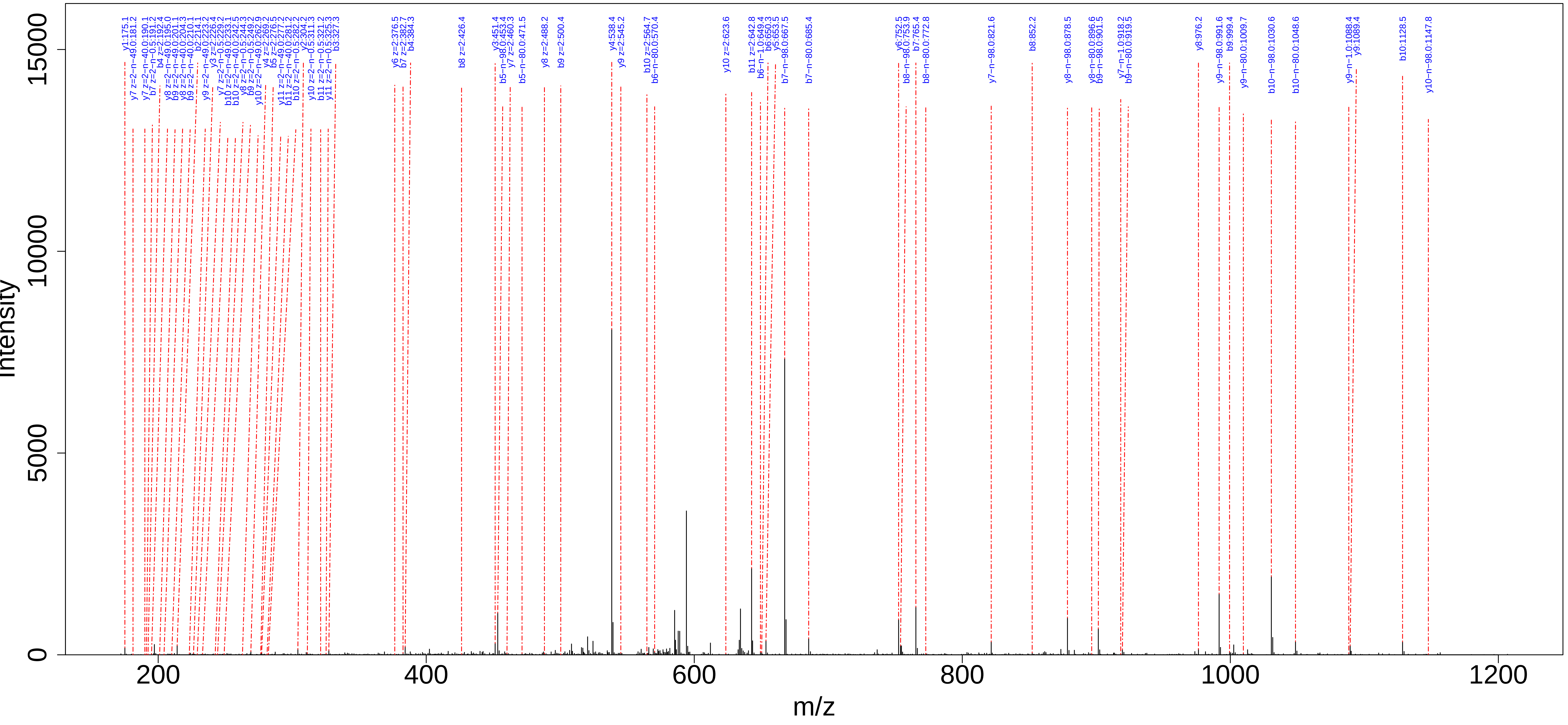

CVPAPGAGASGGTSPSATQPNPAVFIFEHK 14−S_Phospho z=+3 Raw scan=3877
TBCD4_HUMAN S102; ; Q= 0.00449269936353426 ; score= 39.3 ; E= 0.00018 m/z=1011.4725

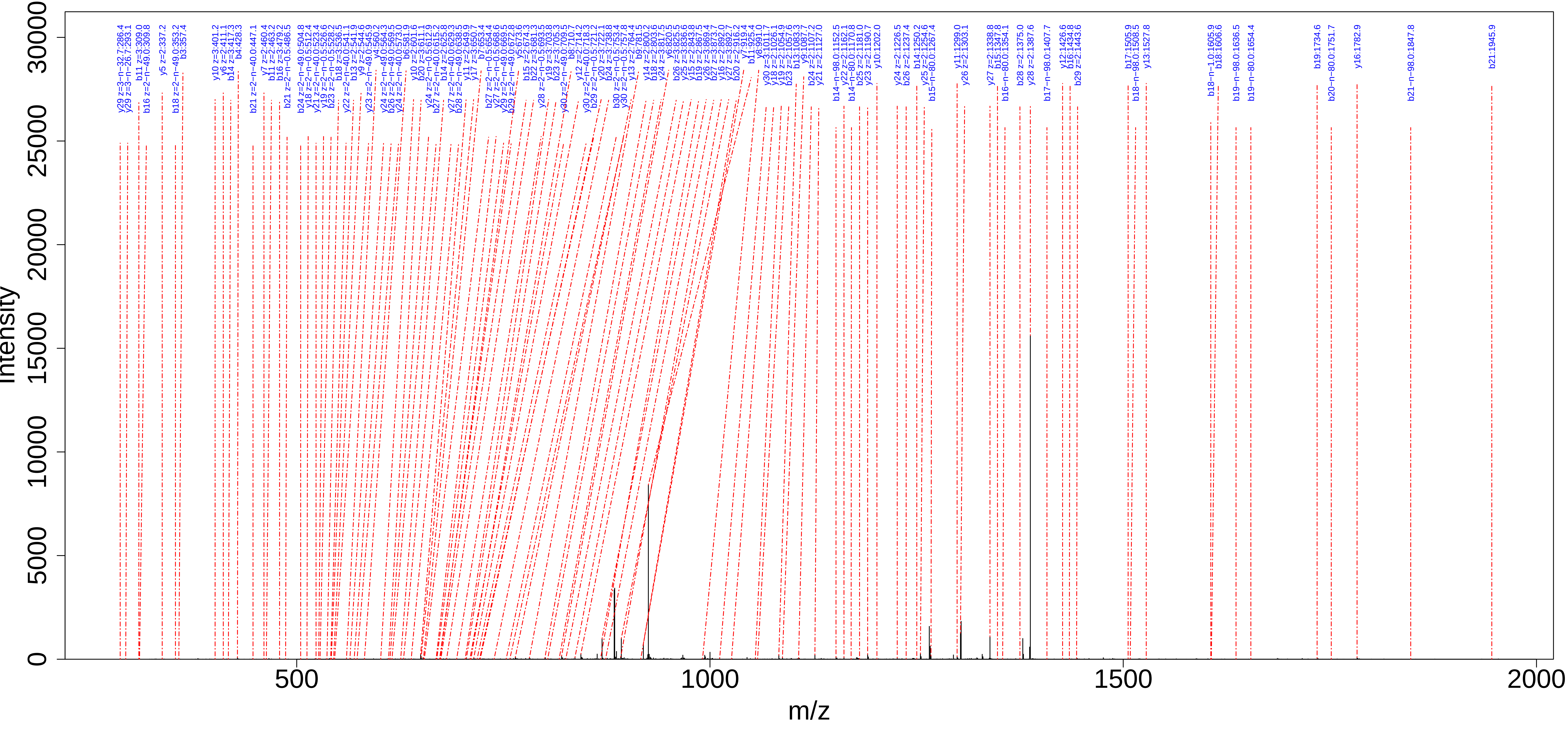

GRLGSVDSFER 5−S_Phospho z=+2 Raw scan=2283
TBCD4_HUMAN S591; ; Q= 0.0102292768959436 ; score= 38 ; E= 0.0037 m/z=651.796

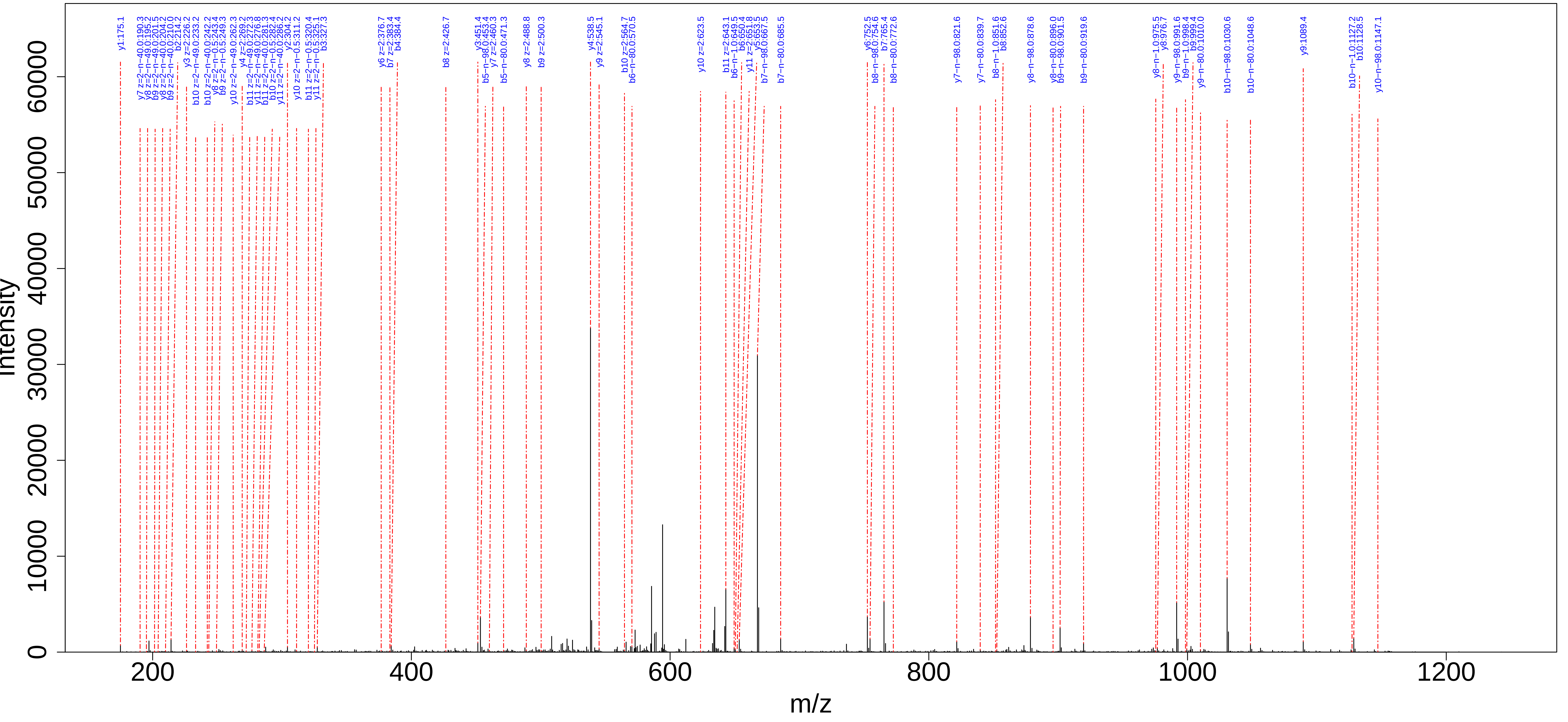

GRLGSVDSFER 5−S_Phospho z=+2 Raw scan=2294
TBCD4_HUMAN S591; ; Q= 0.0102256699576869 ; score= 38 ; E= 0.0021 m/z=651.7952

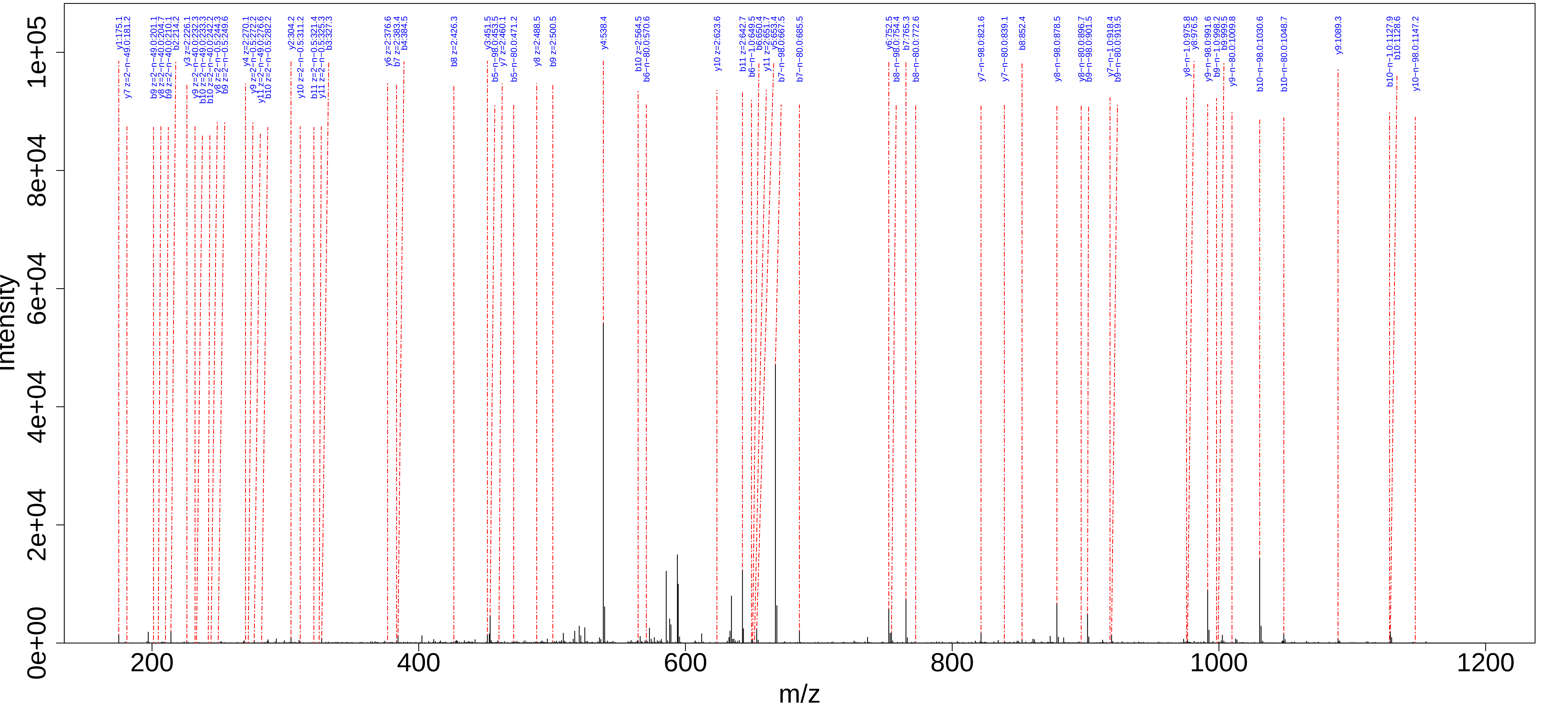

GRLGSVDSFER 5−S_Phospho z=+2 Raw scan=2301

TBCD4_HUMAN S591; ; Q= 0.0102220655622136 ; score= 38 ; E= 0.013 m/z=651.7939

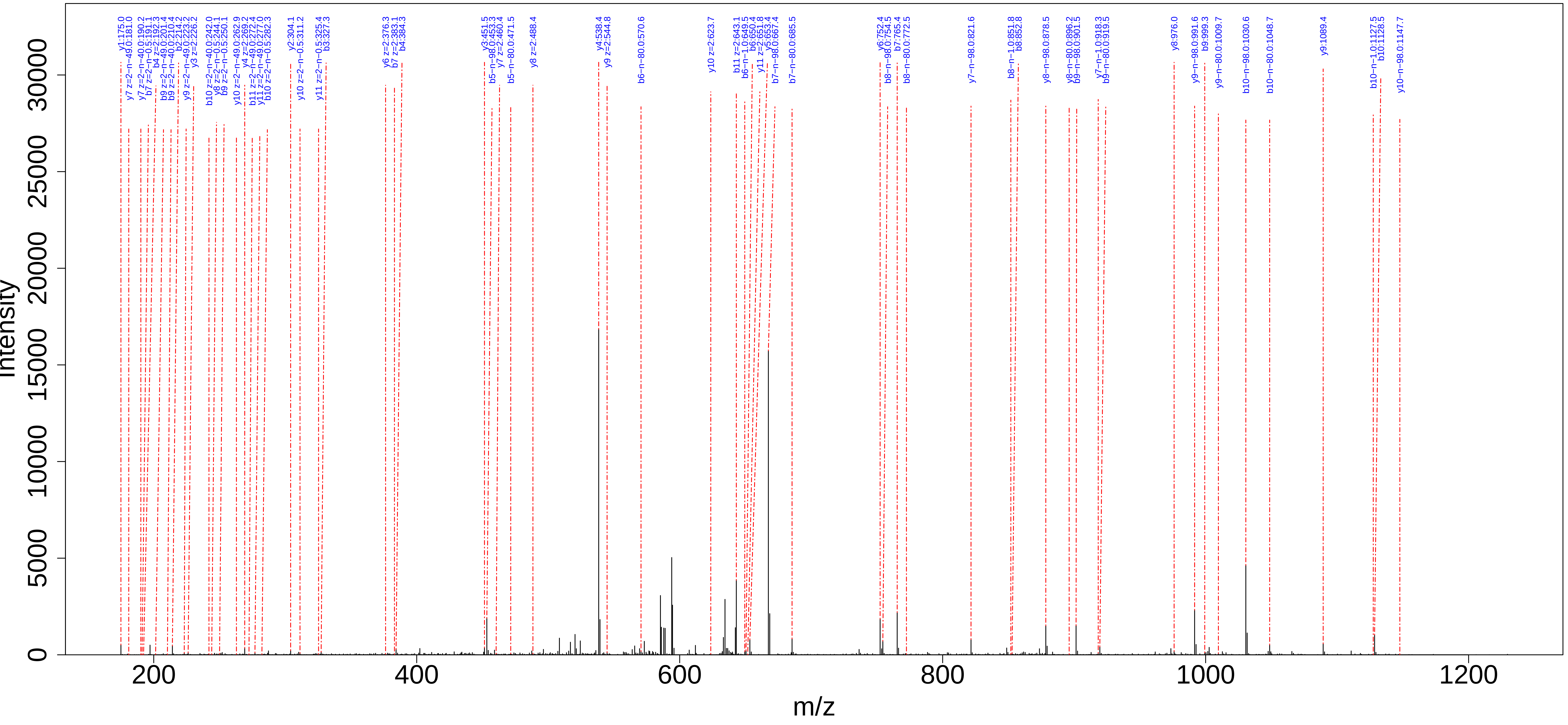

ESCPSVSIPSSDEHREK 5−S_Phospho z=+3 Raw scan=1659
SGK3_HUMAN S17; ; Q= 0.00988074957410562 ; score= 36.6 ; E= 0.011 m/z=675.2867

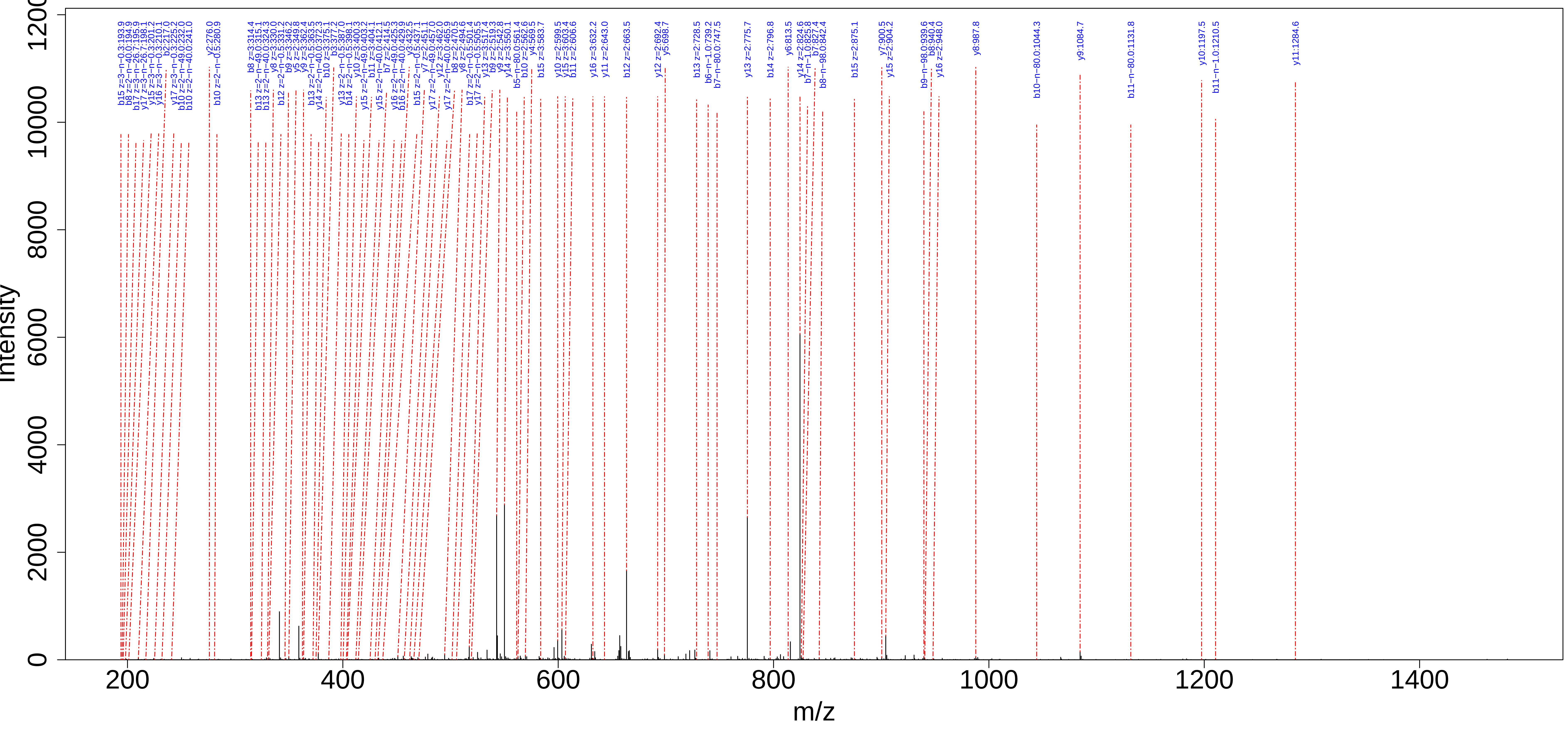

ESCPSVSIPSSDEHREK 10−S_Phospho z=+3 Raw scan=1699
SGK3_HUMAN S21; ; Q= 0.00956779940613659 ; score= 36.1 ; E= 0.069 m/z=675.2866

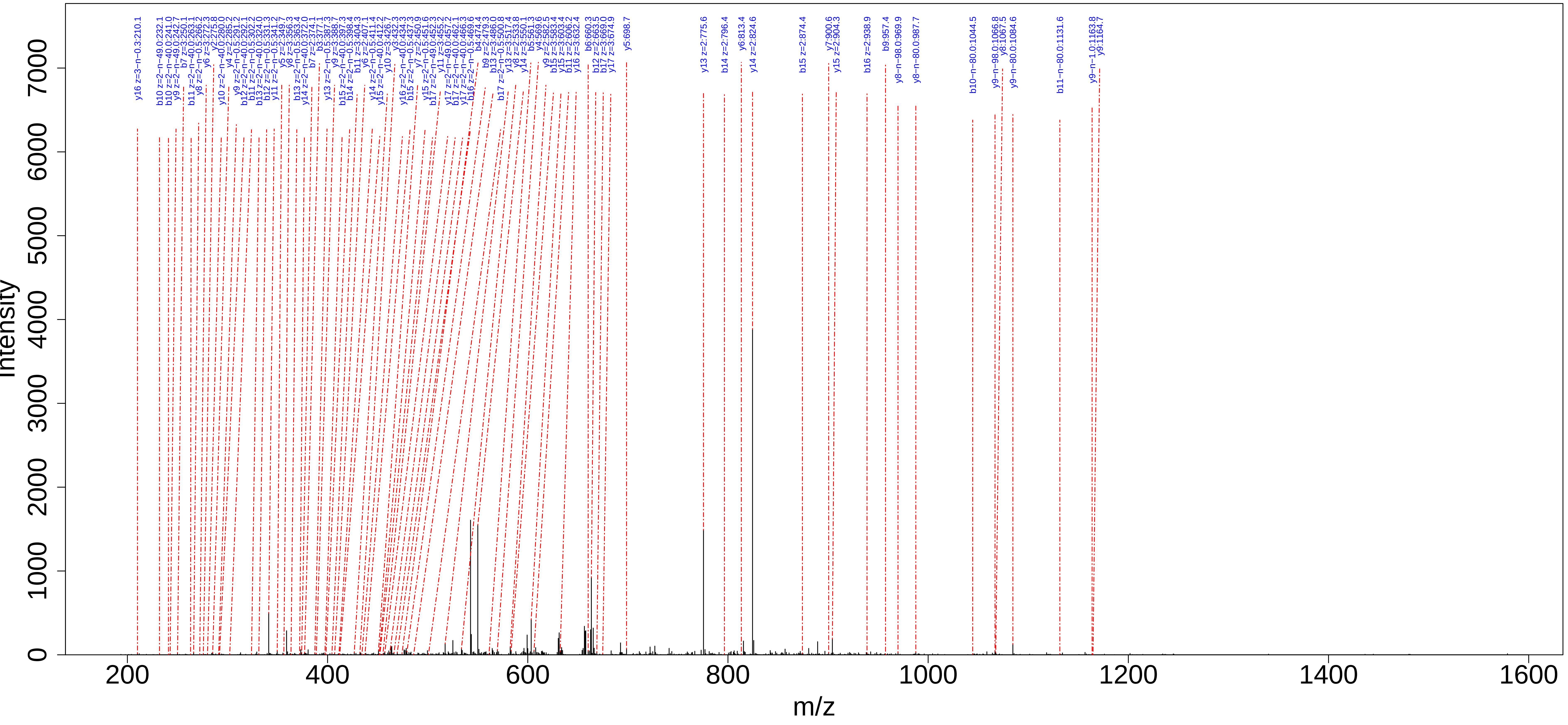

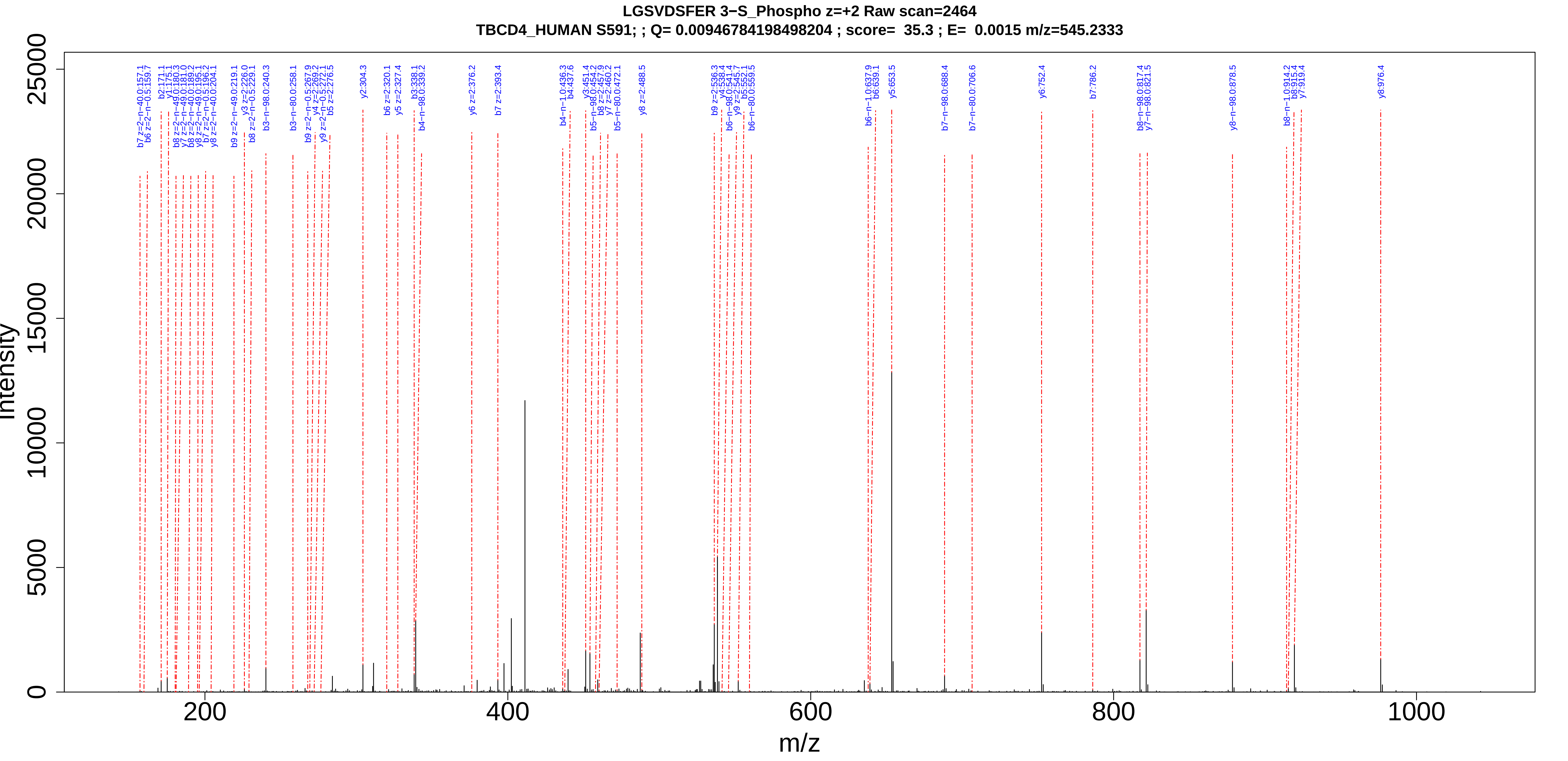

LGSVDSFER 3−S_Phospho z=+2 Raw scan=2464
TBCD4_HUMAN S591; ; Q= 0.00946784198498204 ; score= 35.3 ; E= 0.0015 m/z=545.2333
Intensity
0
5000
10000
15000
20000
25000
m/z
200
400
600
800
1000
b7 z=2−n−40.0:157.1
b6 z=2−n−0.5:159.7
b2:171.1
y1:175.1
b8 z=2−n−49.0:180.3
y7 z=2−n−49.0:181.0
b8 z=2−n−40.0:189.2
y8 z=2−n−49.0:195.1
b7 z=2−n−0.5:196.2
y8 z=2−n−40.0:204.1
b9 z=2−n−49.0:219.1
y3 z=2:226.0
b8 z=2−n−0.5:229.1
b3−n−98.0:240.3
b3−n−80.0:258.1
b9 z=2−n−0.5:267.9
y4 z=2:269.2
y9 z=2−n−0.5:272.1
b5 z=2:276.5
y2:304.3
b6 z=2:320.1
y5 z=2:327.4
b3:338.1
b4−n−98.0:339.2
y6 z=2:376.2
b7 z=2:393.4
b4−n−1.0:436.3
b4:437.6
y3:451.4
b5−n−98.0:454.2
b8 z=2:457.9
y7 z=2:460.2
b5−n−80.0:472.1
y8 z=2:488.5
b9 z=2:536.3
y4:538.4
b6−n−98.0:541.4
y9 z=2:545.7
b5:552.1
b6−n−80.0:559.5
b6−n−1.0:637.9
b6:639.1
y5:653.5
b7−n−98.0:688.4
b7−n−80.0:706.6
y6:752.4
b7:786.2
b8−n−98.0:817.4
y7−n−98.0:821.5
y8−n−98.0:878.5
b8−n−1.0:914.2
b8:915.4
y7:919.4
y8:976.4

LGSVDSFER 3−S_Phospho z=+2 Raw scan=2445

TBCD4_HUMAN S591; ; Q= 0.00946475195822454 ; score= 35.3 ; E= 0.0057 m/z=545.2329

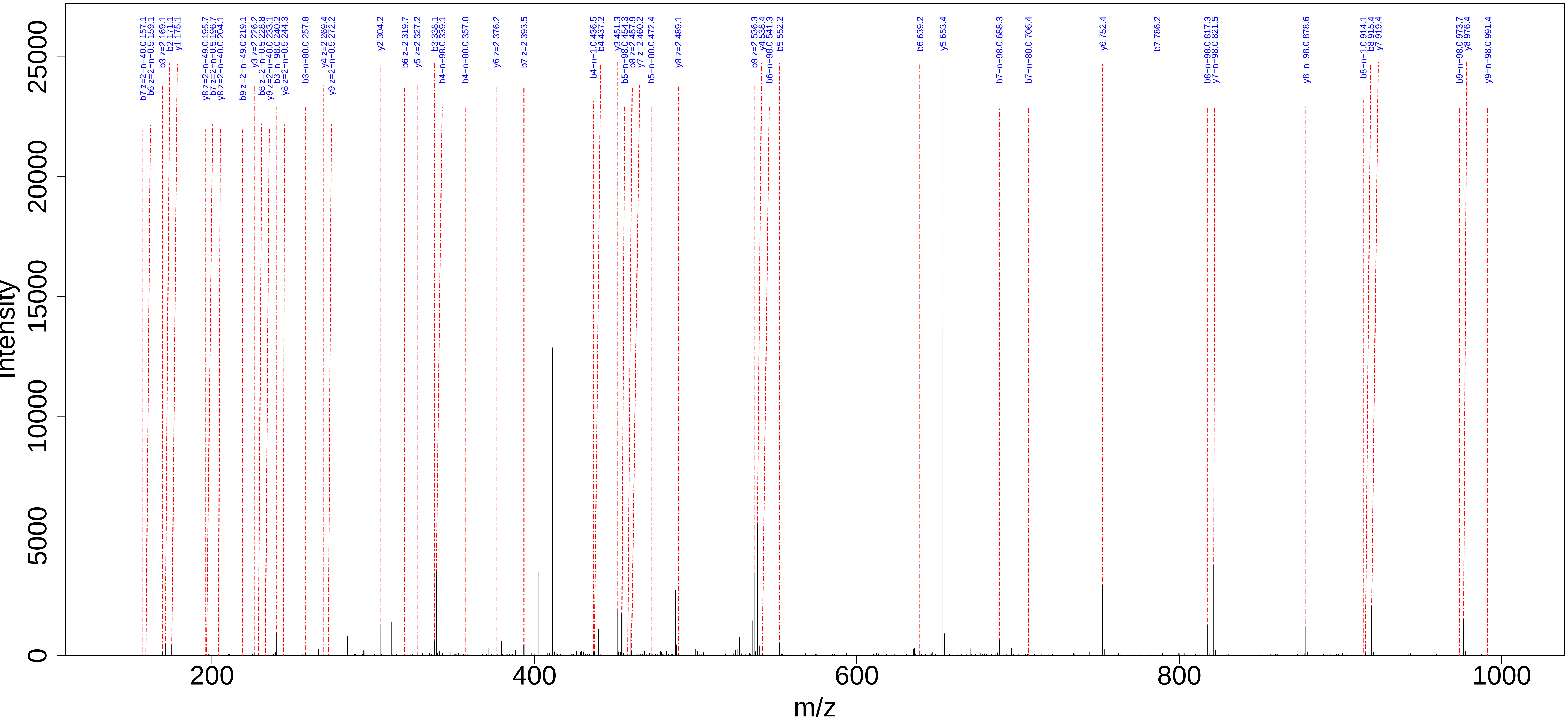

GRLGSVDSFER 5−S_Phospho z=+2 Raw scan=2325
TBCD4_HUMAN S591; ; Q= 0.00923566878980892 ; score= 34.4 ; E= 0.009 m/z=651.7945

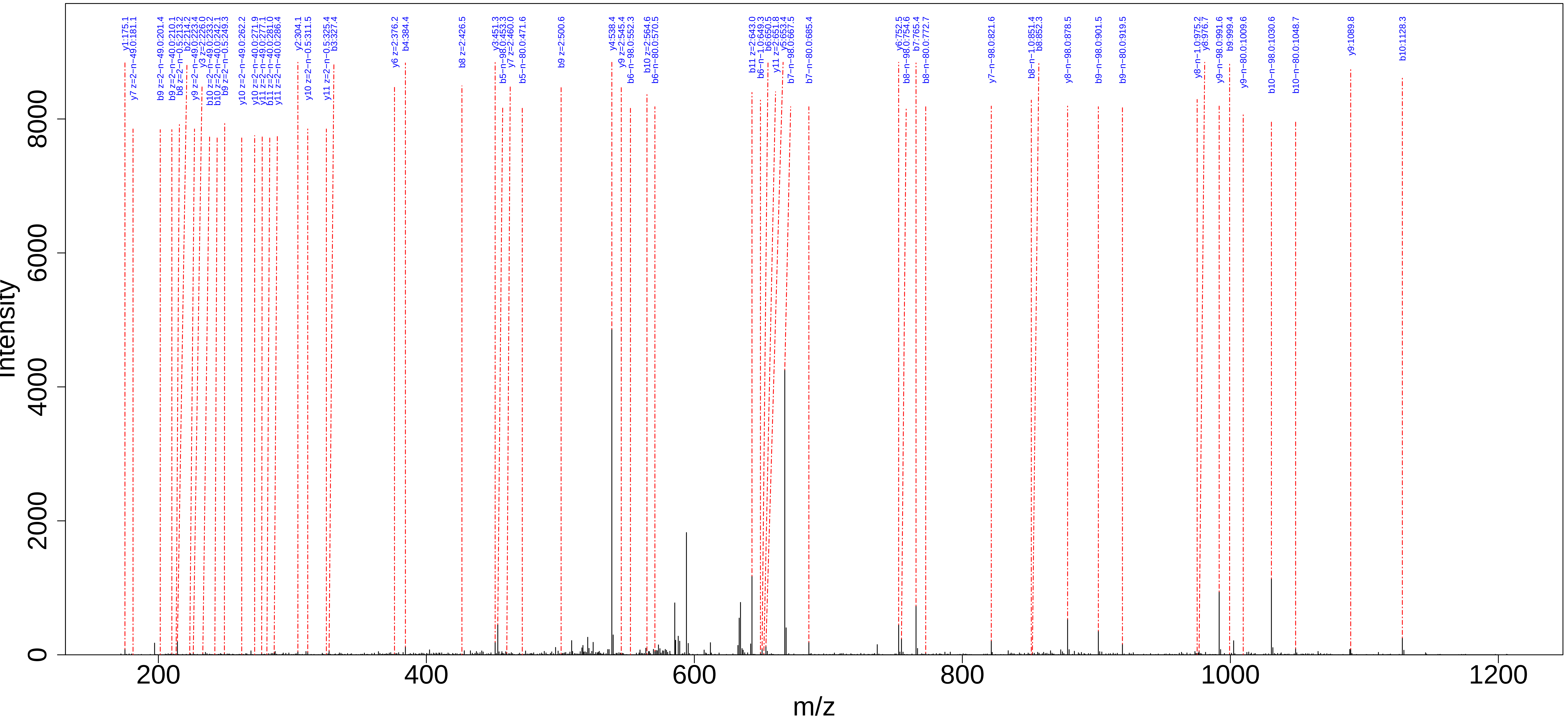

GRLGSVDSFER 5−S_Phospho z=+2 Raw scan=2287
TBCD4_HUMAN S591; ; Q= 0.00923272843043617 ; score= 34.4 ; E= 0.038 m/z=651.795

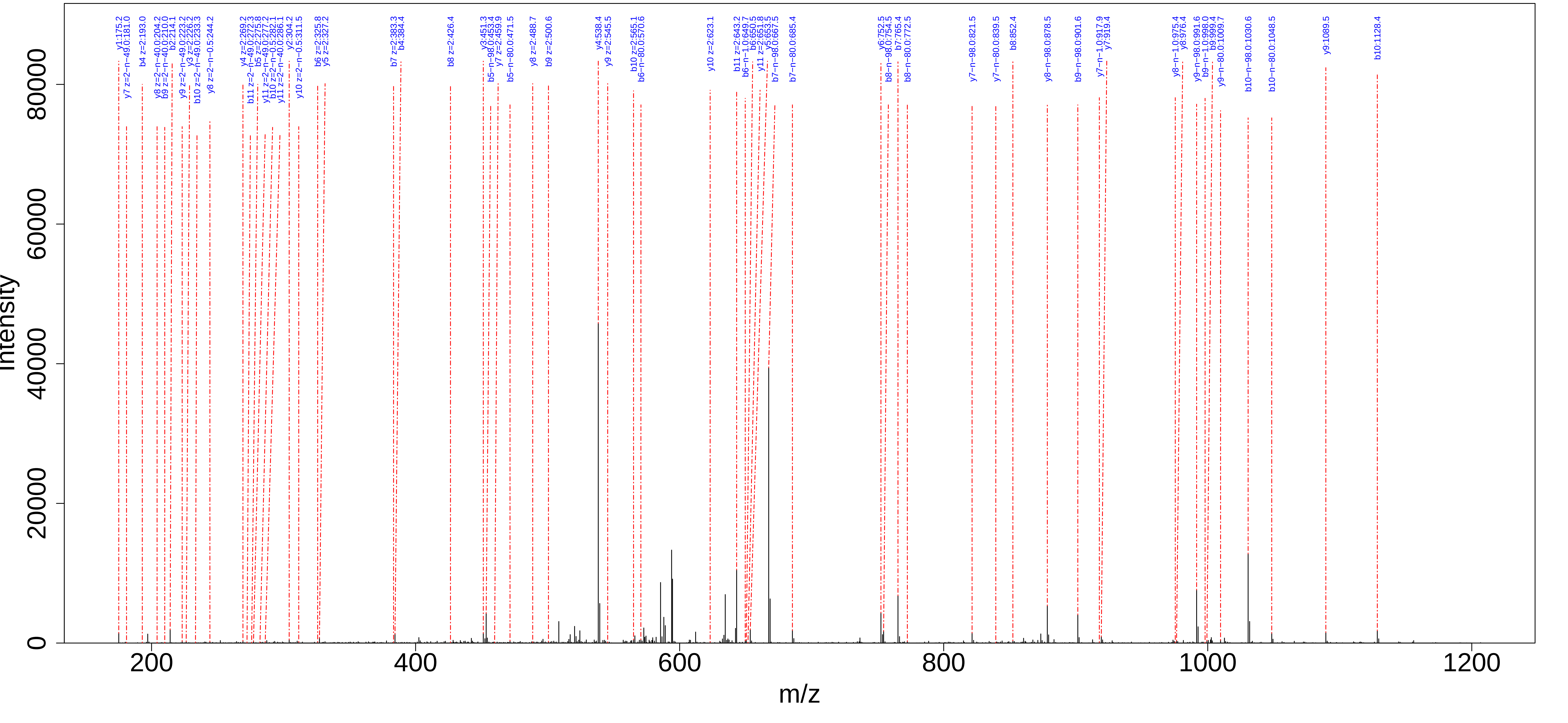

LGSVDSFER 3−S_Phospho z=+2 Raw scan=2481
TBCD4_HUMAN S591; ; Q= 0.0102230483271375 ; score= 32.9 ; E= 0.0087 m/z=545.2328

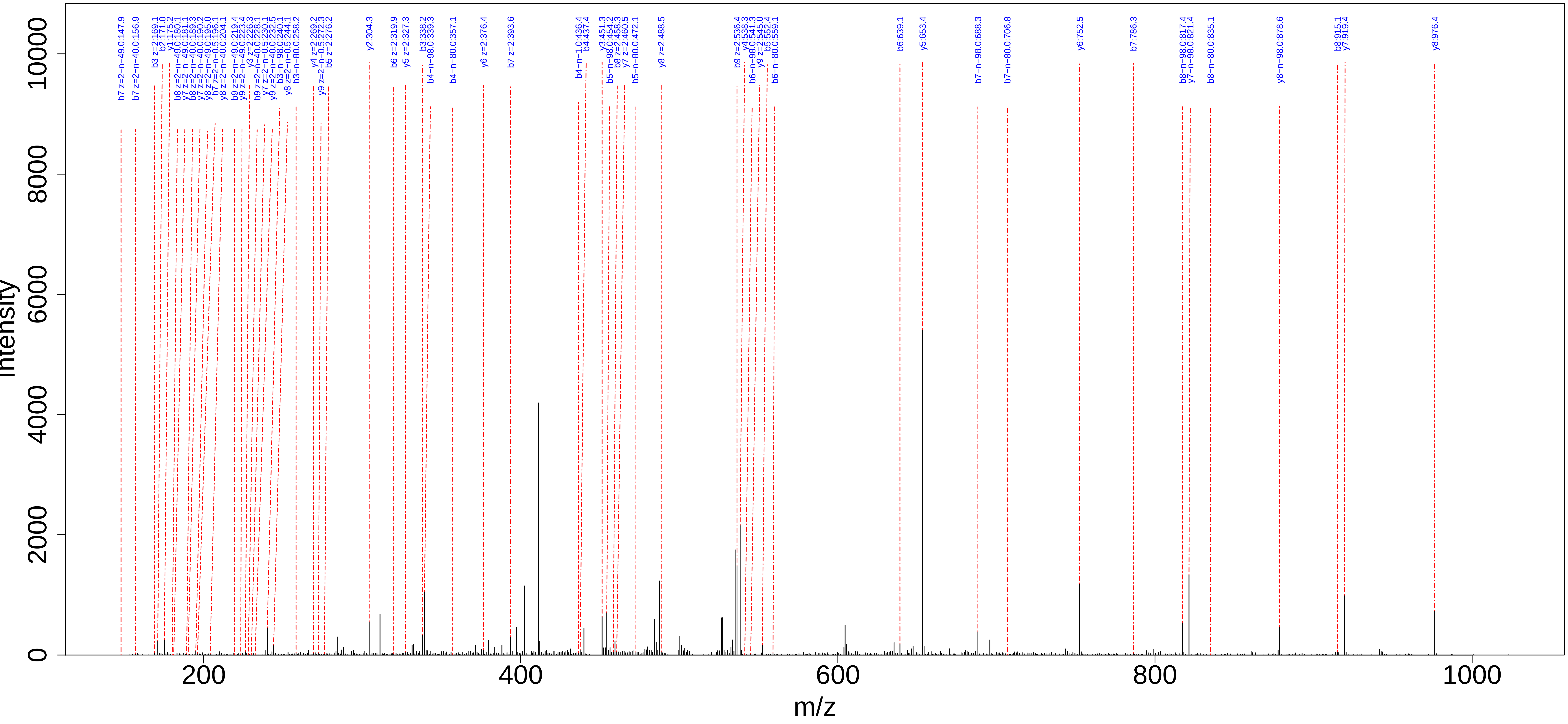

LGSVDSFER 3−S_Phospho z=+2 Raw scan=2455

TBCD4_HUMAN S591; ; Q= 0.0102198823165067 ; score= 32.9 ; E= 0.0022 m/z=545.2332

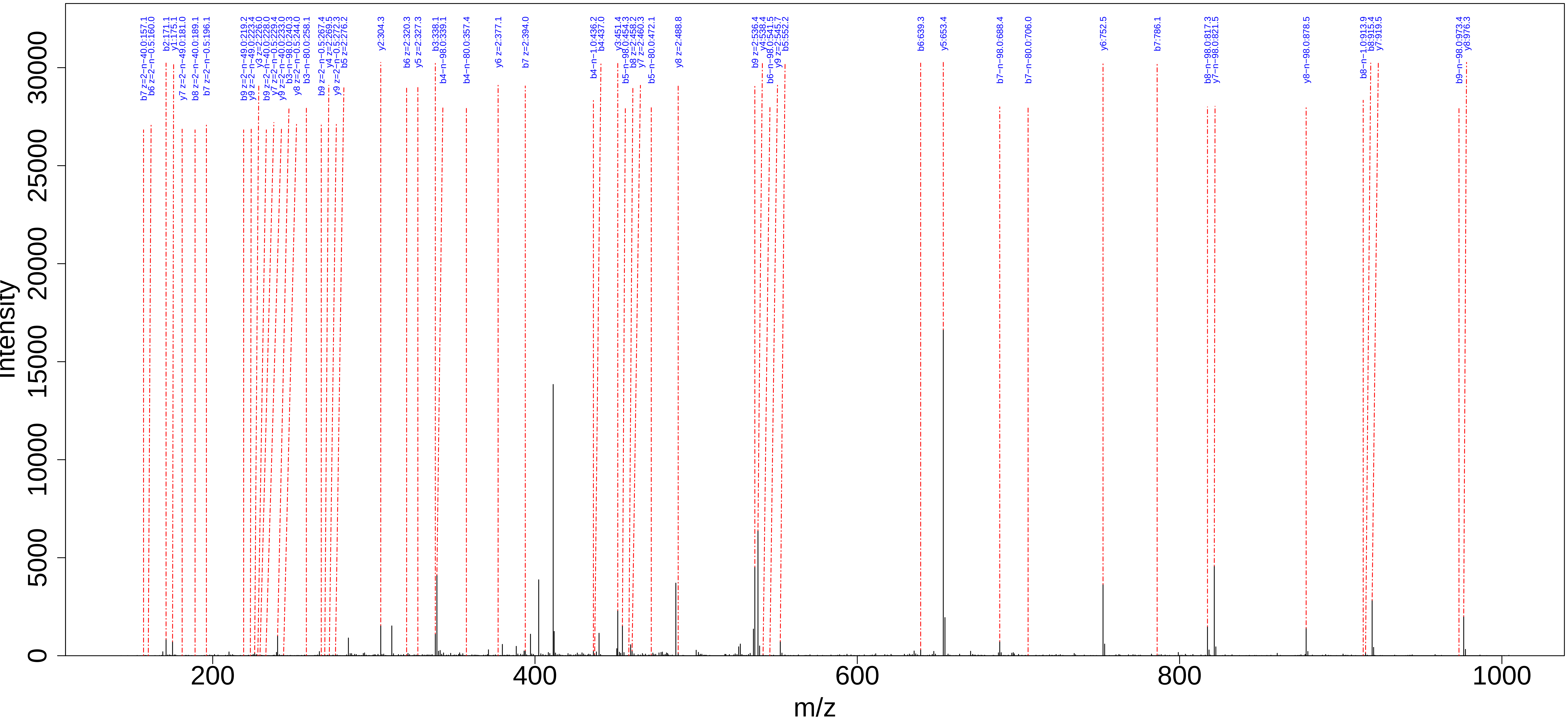

LGSVDSFER 3−S_Phospho z=+2 Raw scan=2473

TBCD4_HUMAN S591; ; Q= 0.0101600985221675 ; score= 32.8 ; E= 0.003 m/z=545.2334

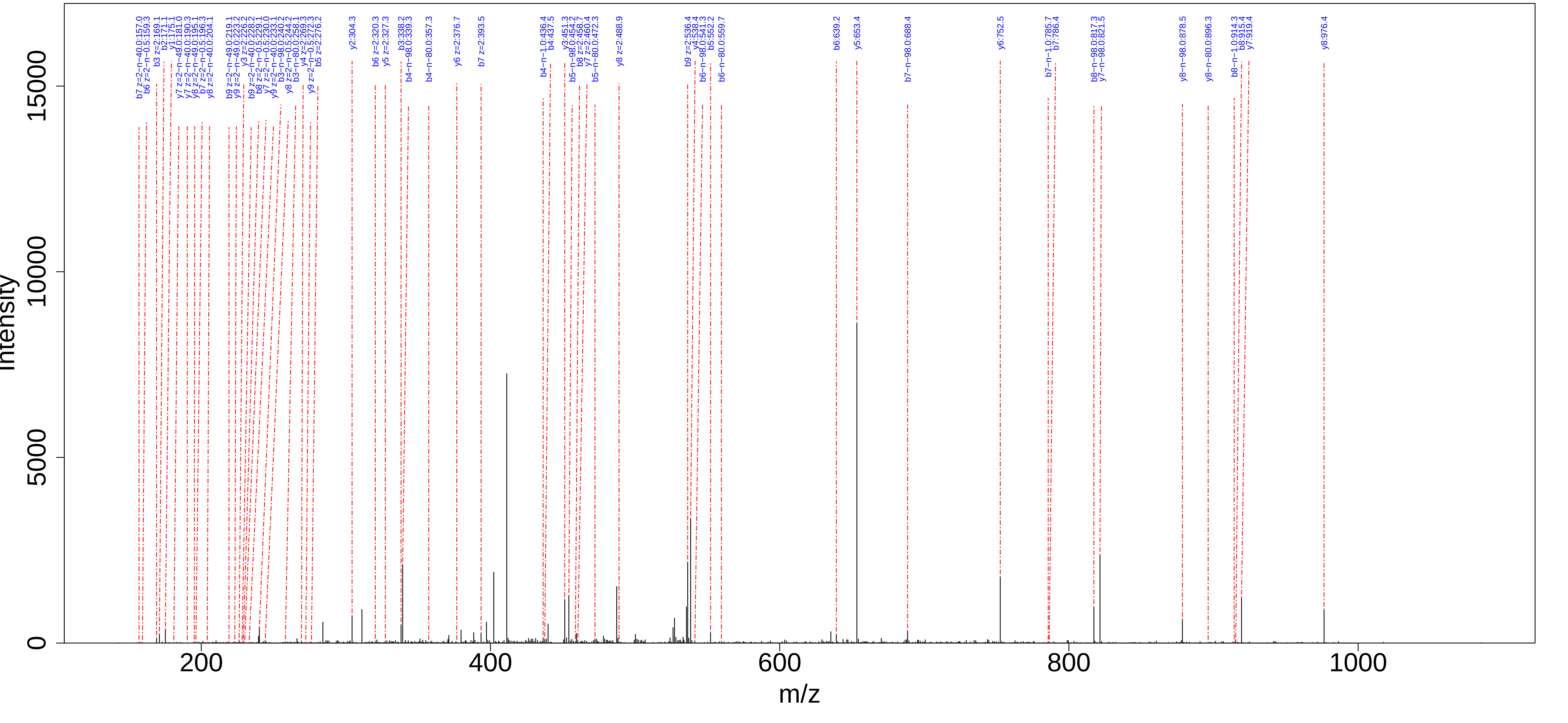

LGSVDSFER 3−S_Phospho z=+2 Raw scan=2449

TBCD4_HUMAN S591; ; Q= 0.0101569713758079 ; score= 32.8 ; E= 0.0034 m/z=545.2337

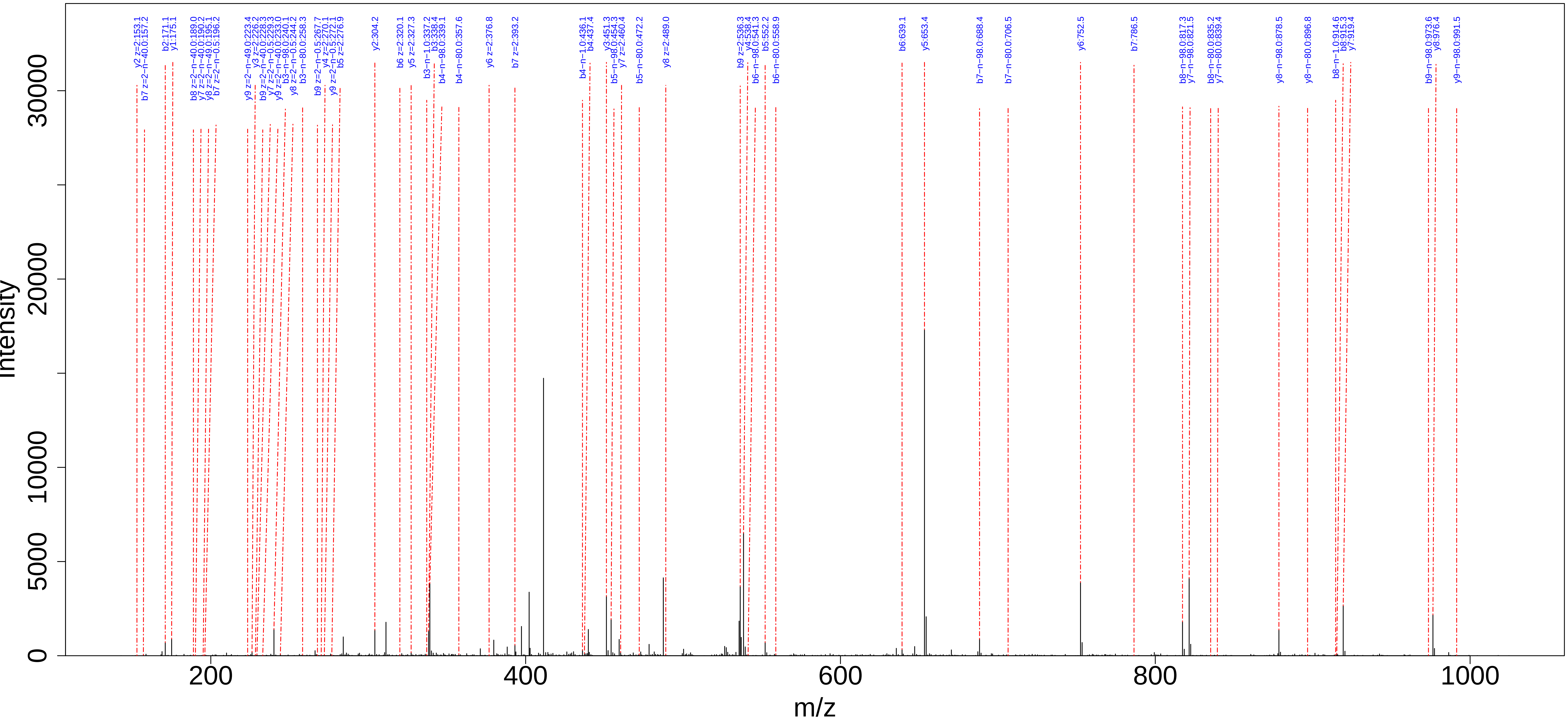

ESCPSVSIPSSDEHREK 5−S_Phospho z=+3 Raw scan=1663
SGK3_HUMAN S20; ; Q= 0.0100273473108478 ; score= 32.4 ; E= 0.073 m/z=675.2861

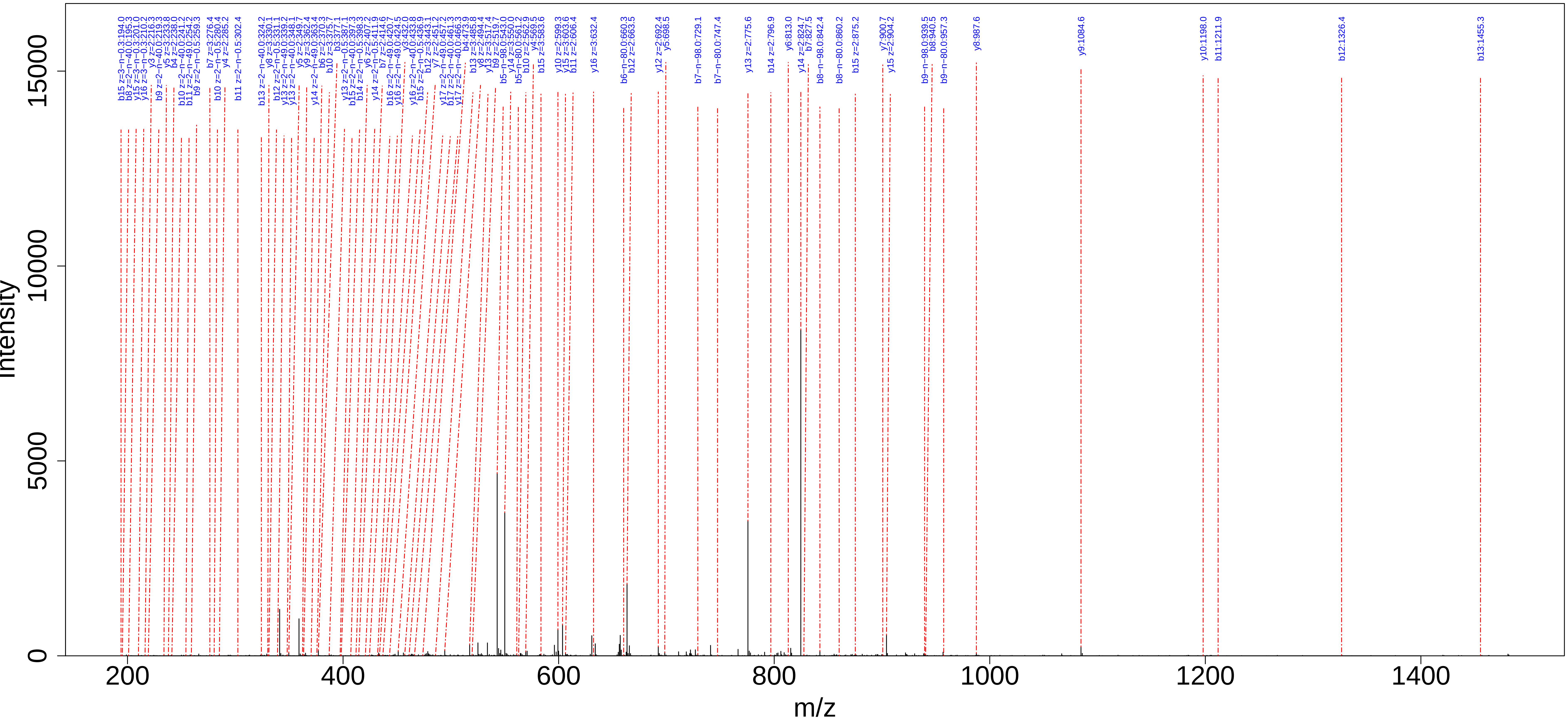

GRLGSVDSFER 5−S_Phospho z=+2 Raw scan=2332
TBCD4_HUMAN S588; ; Q= 0.00983313468414779 ; score= 31.5 ; E= 0.064 m/z=651.7938

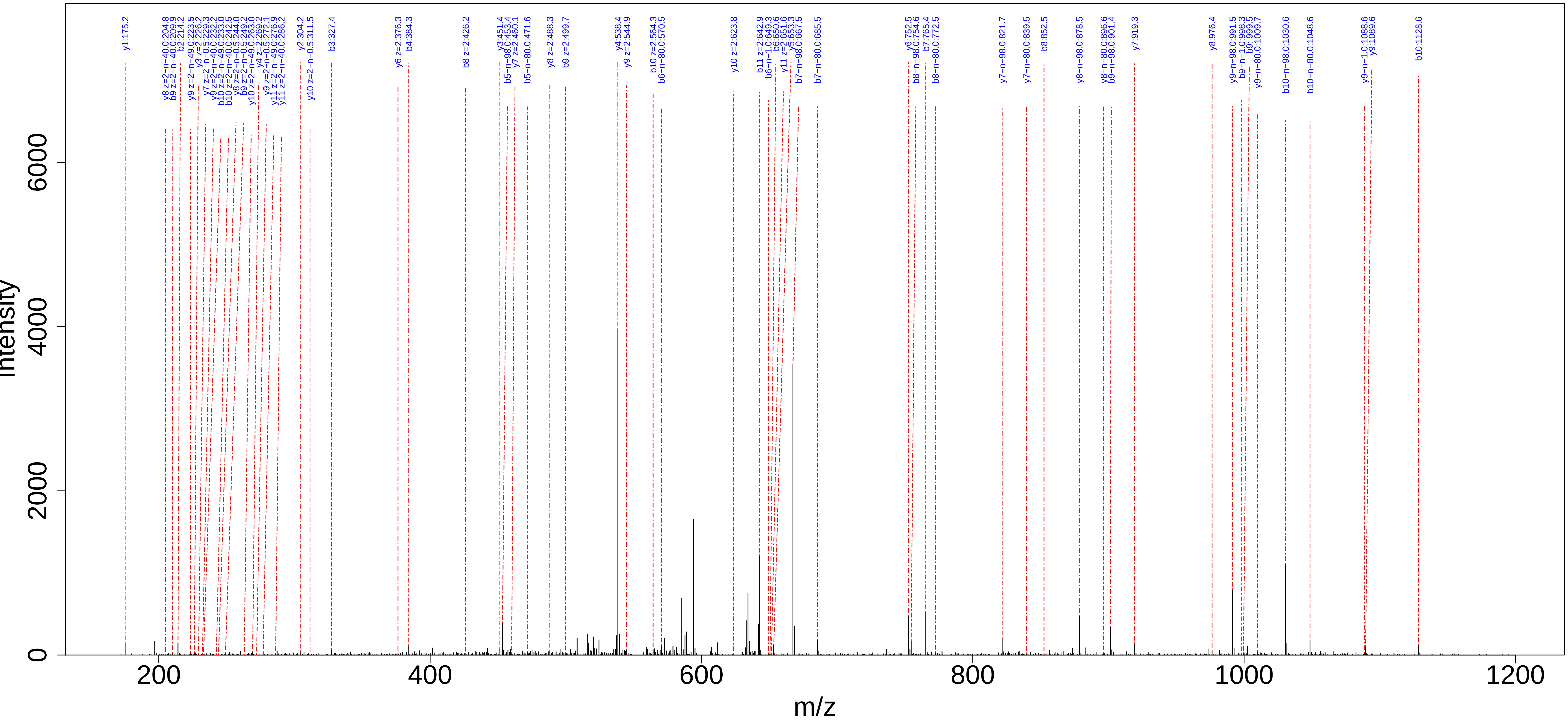

LGSVDSFER 3−S_Phospho z=+2 Raw scan=2496

TBCD4_HUMAN S591; ; Q= 0.00976331360946745 ; score= 31.2 ; E= 0.004 m/z=545.2333

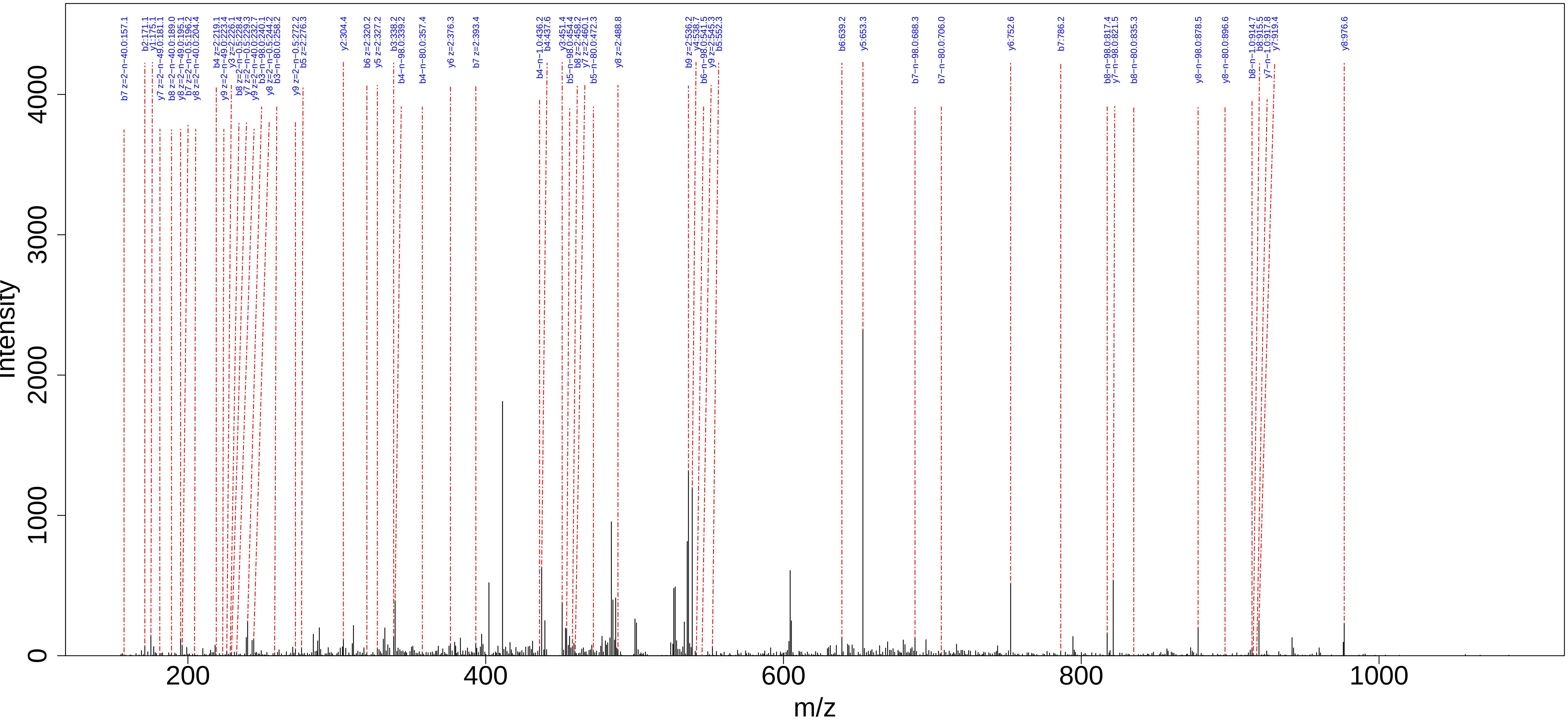

Supplement: Supplementary file 1 [file cells-15-01582-s001.zip › Supplementary Data S2_Mass spectrometry analysis.pdf]
