# Supplementary material for: WNK1 Phosphorylation Sites in TBC1D1 and TBC1D4 Modulate Cell-Surface Abundance of GLUT1
Source: Cells. 2026 Aug 31;15(17):1582. doi: 10.3390/cells15171582 (PMC13564549; doi:10.3390/cells15171582)

cells

MDPI

Article

WNK1 phosphorylation sites in TBC1D1 and TBC1D4 modulate cell surface expression of GLUT1 *

Andreia F. A. Henriques[1,2], Paulo Matos[1,2], Ana Sofia Carvalho[3], Mikel Azkargorta[4], Felix Elortza[4], Rune Matthiesen[3], Peter Jordan[1,2,*]

[1] Department of Human Genetics, National Health Institute 'Dr. Ricardo Jorge', 1649-016 Lisbon, Portugal;
[2] BioISI – Instituto de Biossistemas e Ciências Integrativas, Faculdade de Ciências, Universidade de Lisboa, 1749-016 Lisboa, Portugal;
[3] Computational and Experimental Biology Group, iNOVA4Health, NOVA Medical School, Faculdade de Ciências Médicas, Universidade NOVA de Lisboa, Lisboa, Portugal;
[4] Proteomics Platform, CIC bioGUNE, BRTA (Basque Research & Technology Alliance), CIBEREHD, Derio, Spain.

* Correspondence: peter.jordan@insa.min-saude.pt

Raw Western blot images used in the indicated Figures

Figure 1A

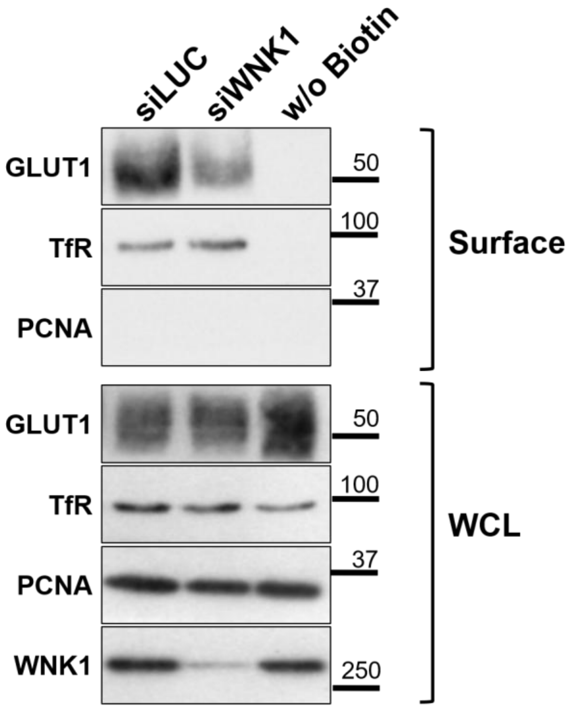

Surface

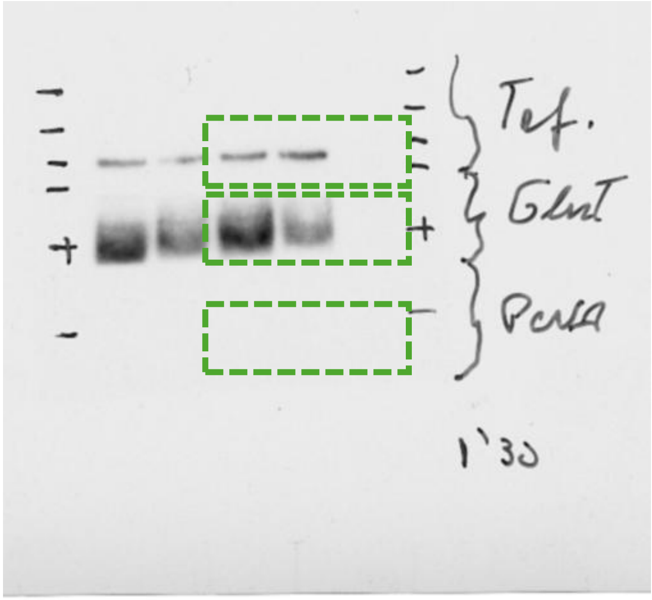

WCL

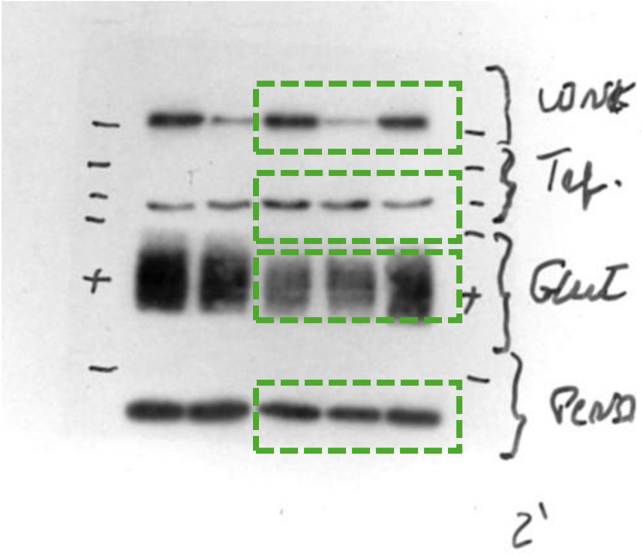

Figure 2A

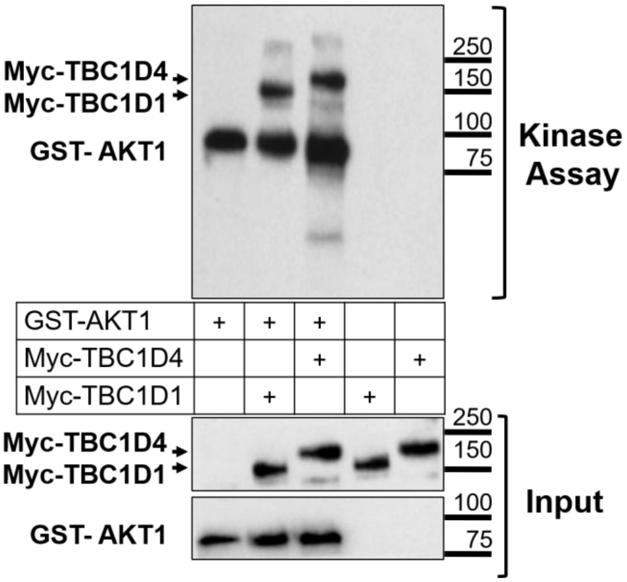

Kinase assay

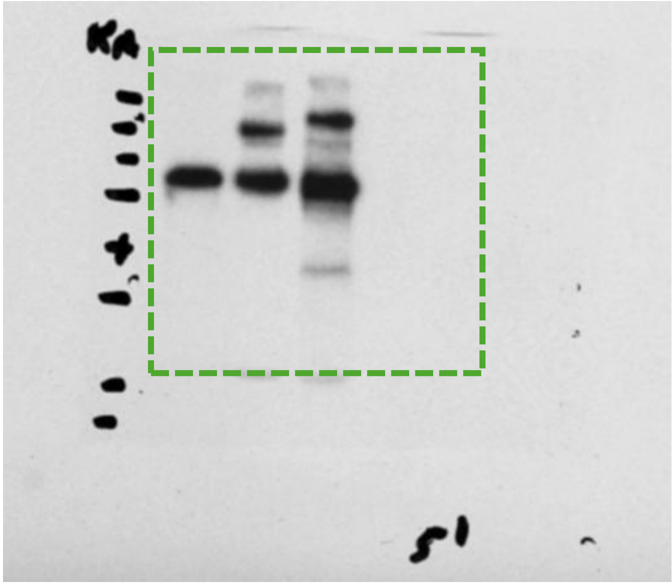

Input

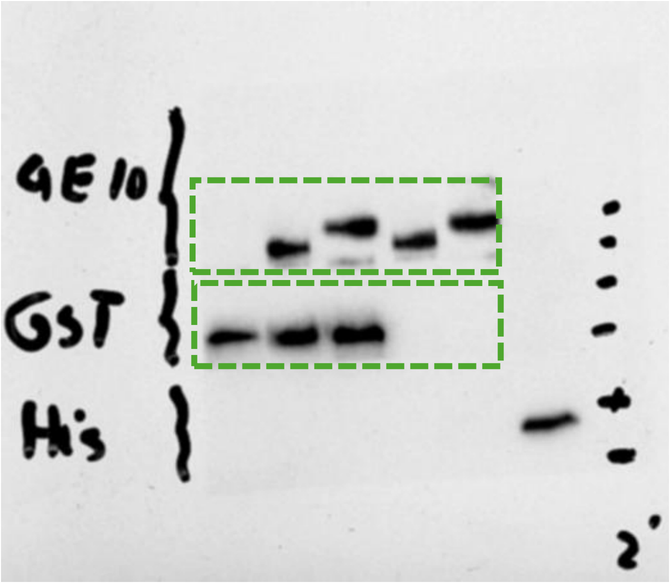

Figure 2B

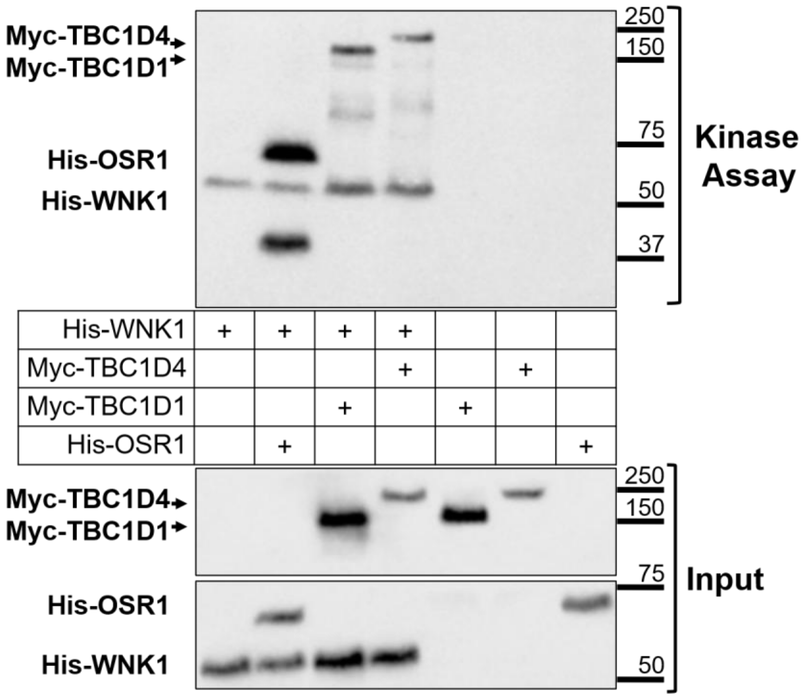

Kinase assay

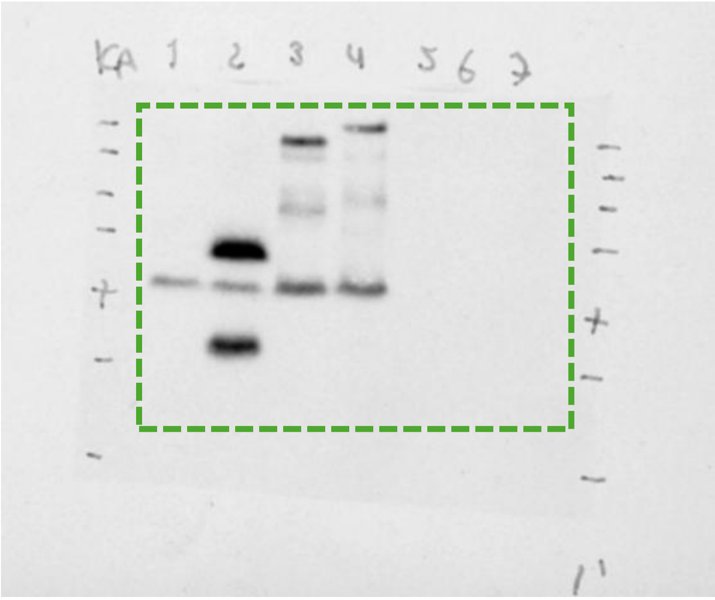

Input

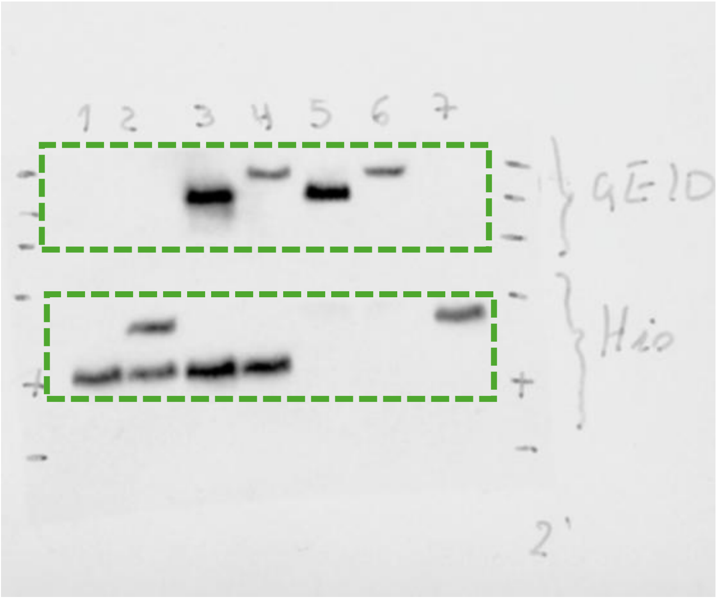

Figure 2C

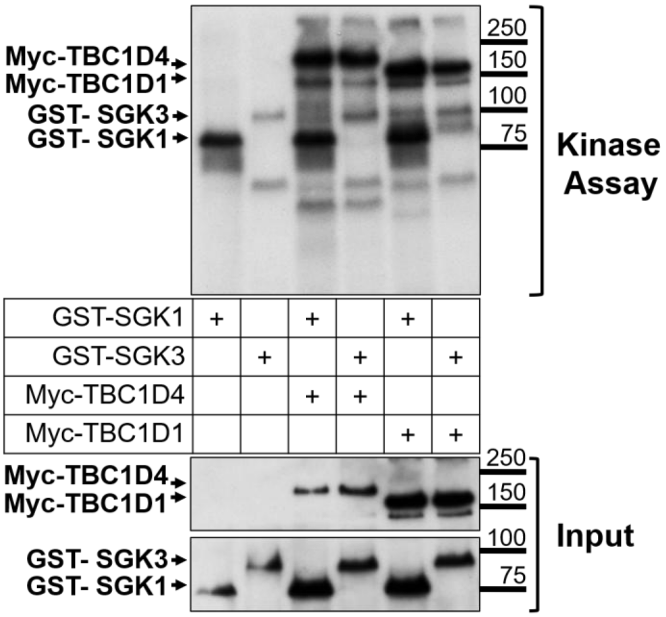

Kinase assay

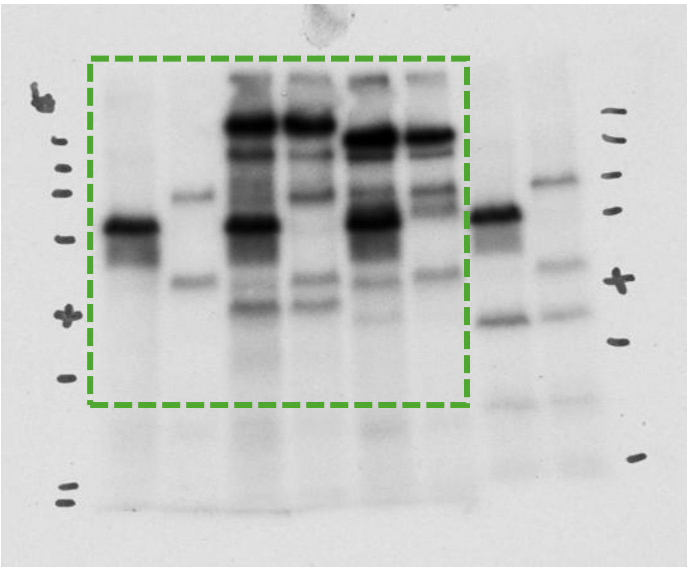

Input

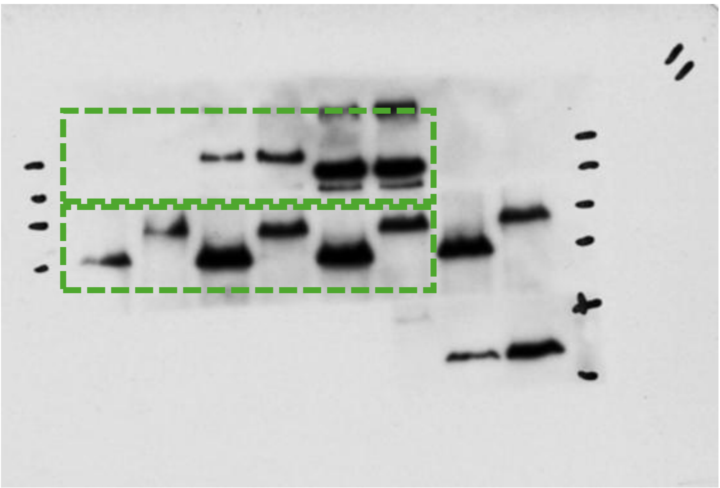

Figure 3A

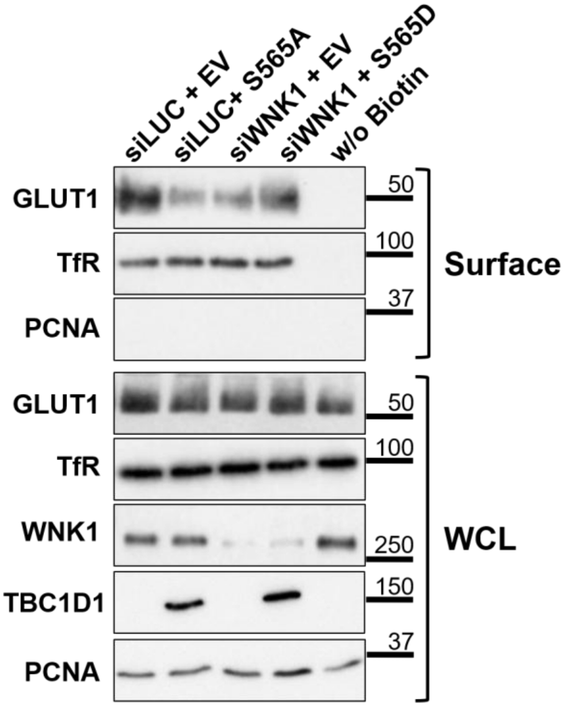

WCL

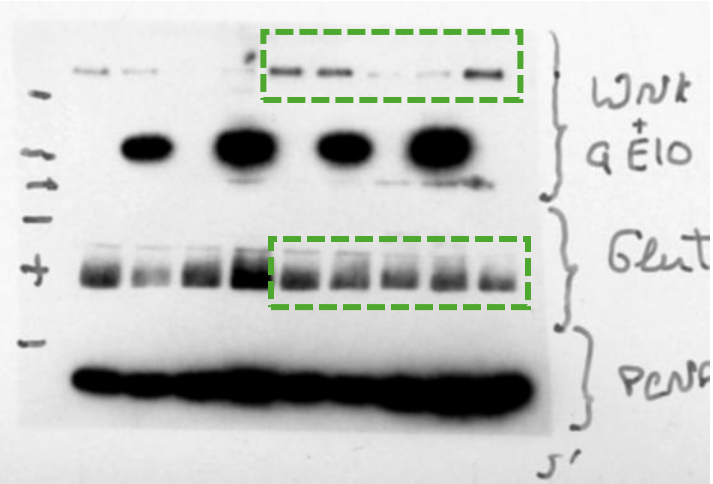

Surface

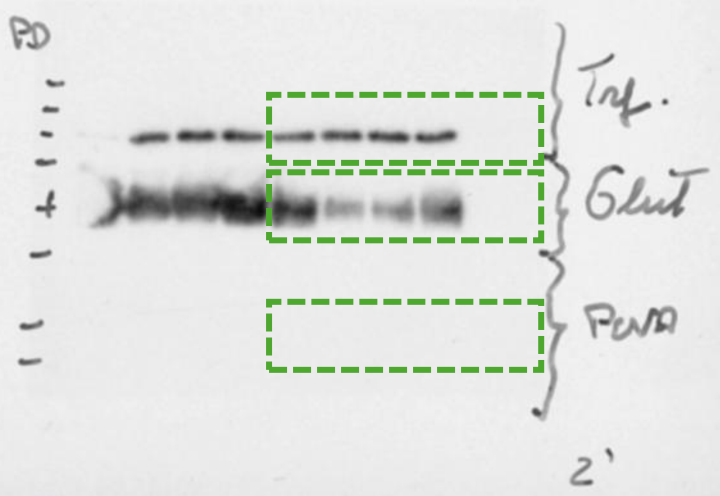

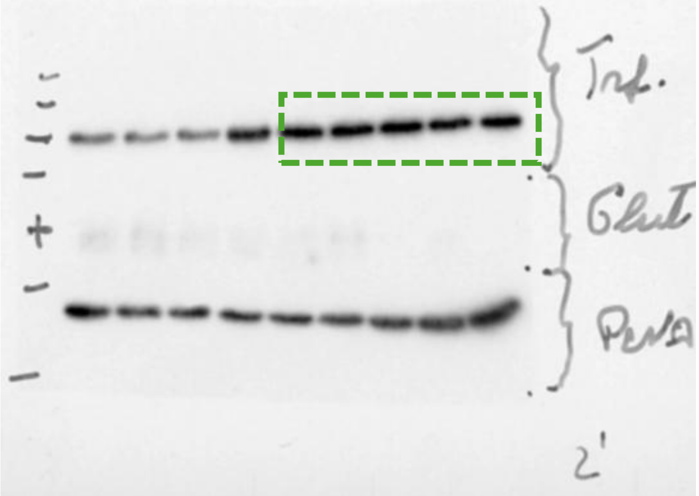

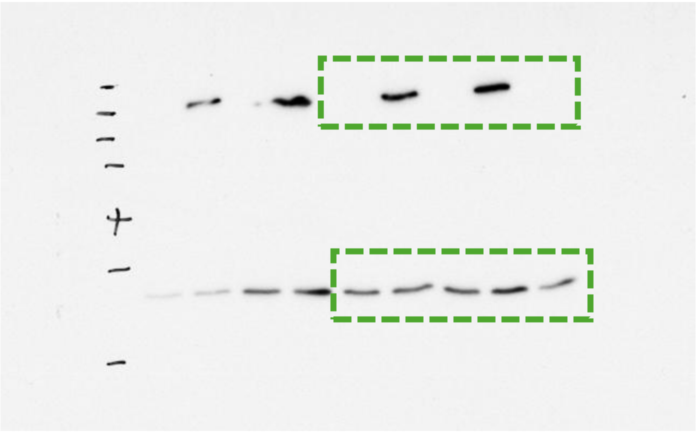

Figure 3B – Quantification of GLUT1 Surface abundance

$$Sample\ relative\ abundance = \frac{GLUT1_{sample}/TfR_{sample}}{GLUT1_{control}/TfR_{control}}$$

Replicate 1

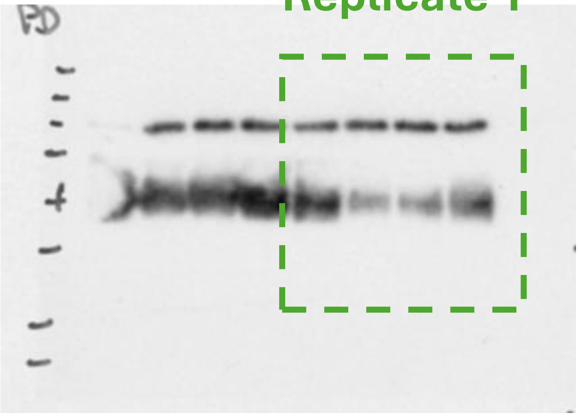

Normalized band intensity	Replicate 1	Replicate 2	Replicate 3
siLUC + EV	1.000	1.000	1.000
siLUC + S565A	0.324	0.483	0.493
siWNK1 + EV	0.374	0.514	0.366
siWNK1 + S565D	0.621	0.772	0.781

Replicate 2

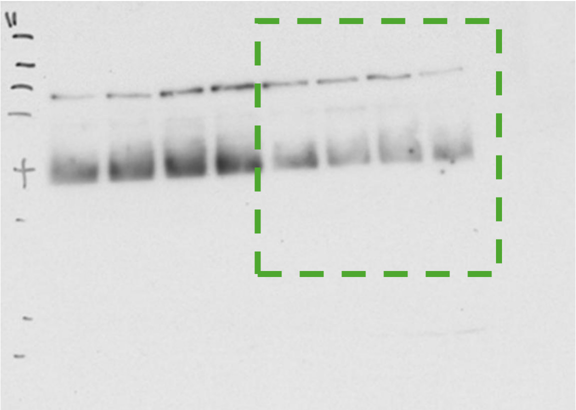

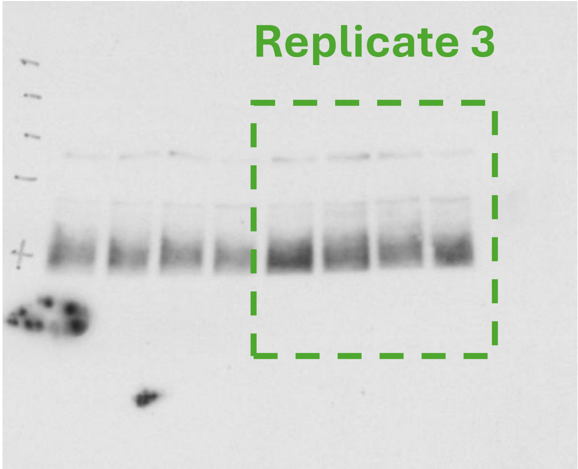

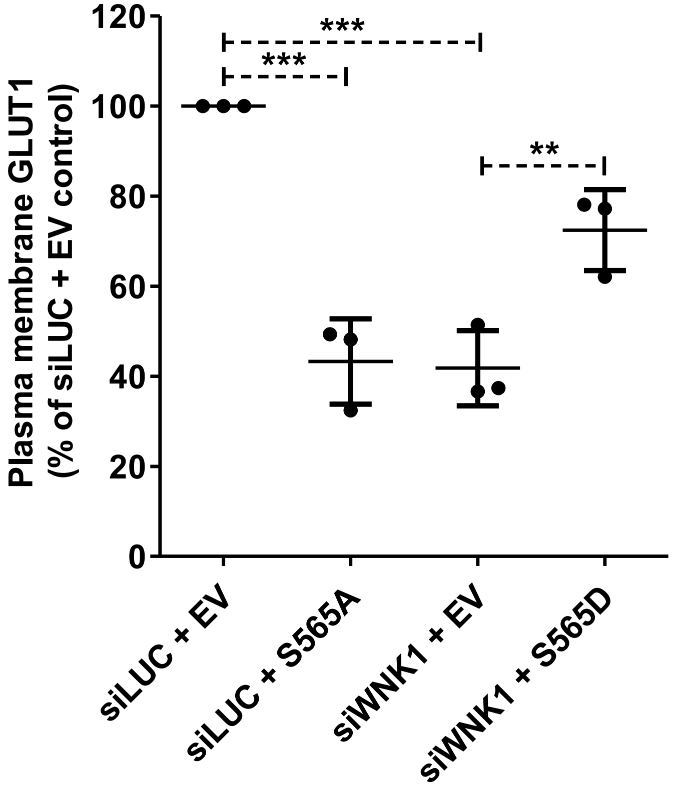

Figure 3C

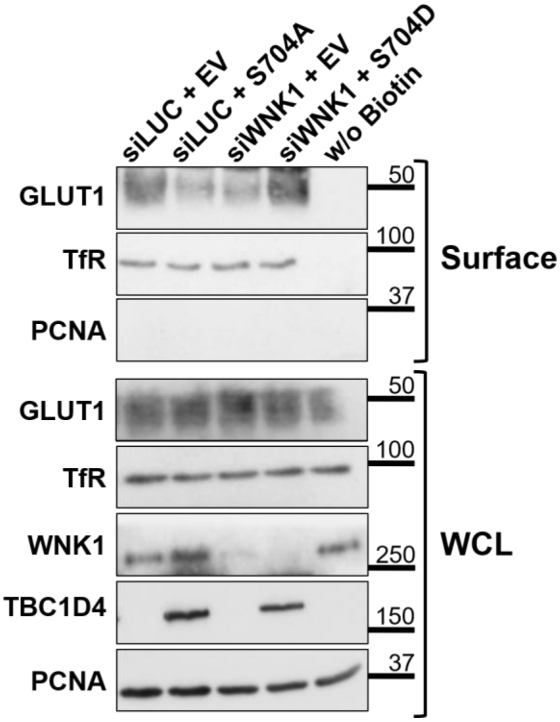

Surface

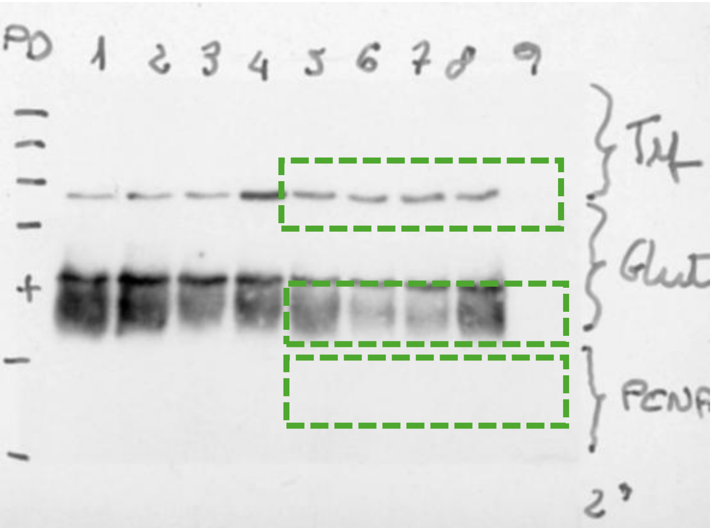

WCL

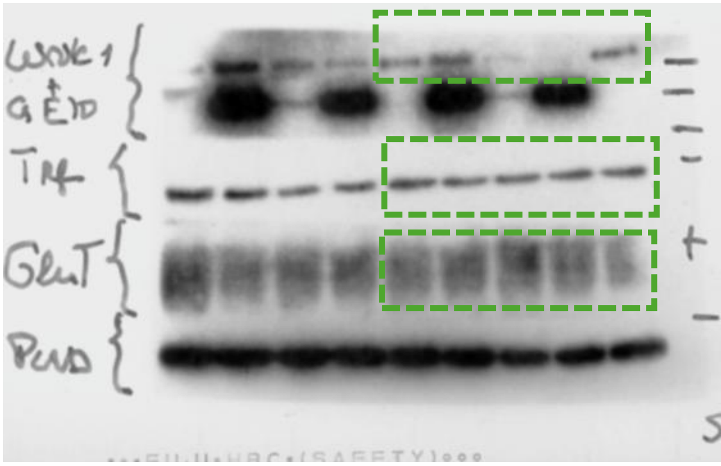

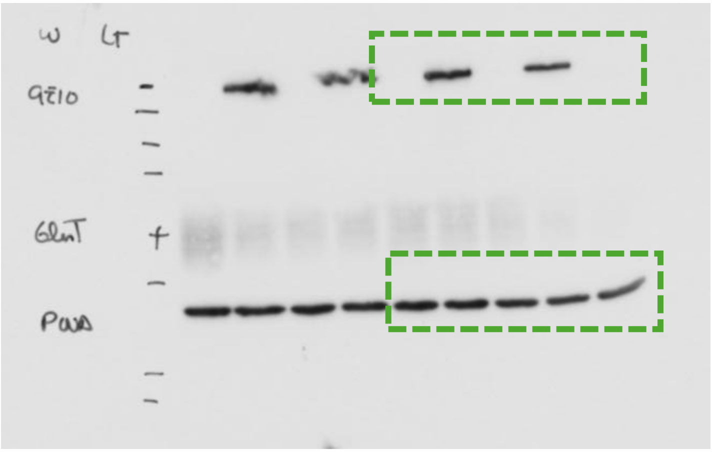

Figure 3D – Quantification of GLUT1 Surface abundance

$$Sample\ relative\ abundance = \frac{GLUT1_{sample}/TfR_{sample}}{GLUT1_{control}/TfR_{control}}$$

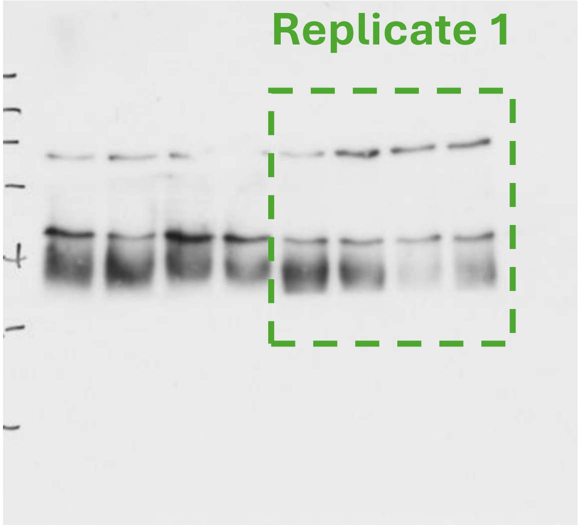

Normalized band Intensity	Replicate 1	Replicate2	Replicate 3
siLUC + EV	1.000	1.000	1.000
siLUC + S704A	0.407	0.533	0.681
siWNK1 + EV	0.387	0.306	0.478
siWNK1 + S704D	0.791	1.368	1.413

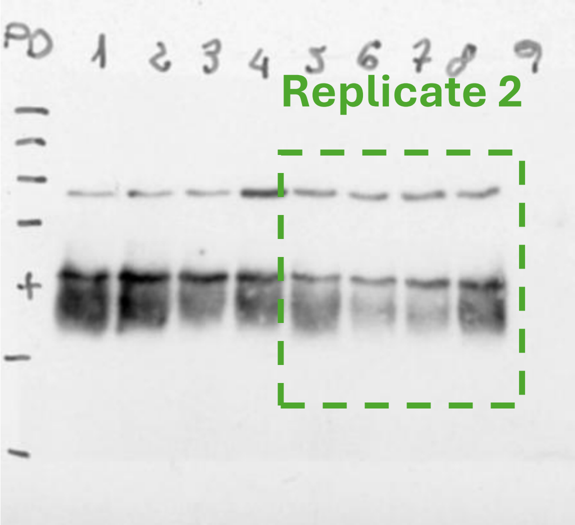

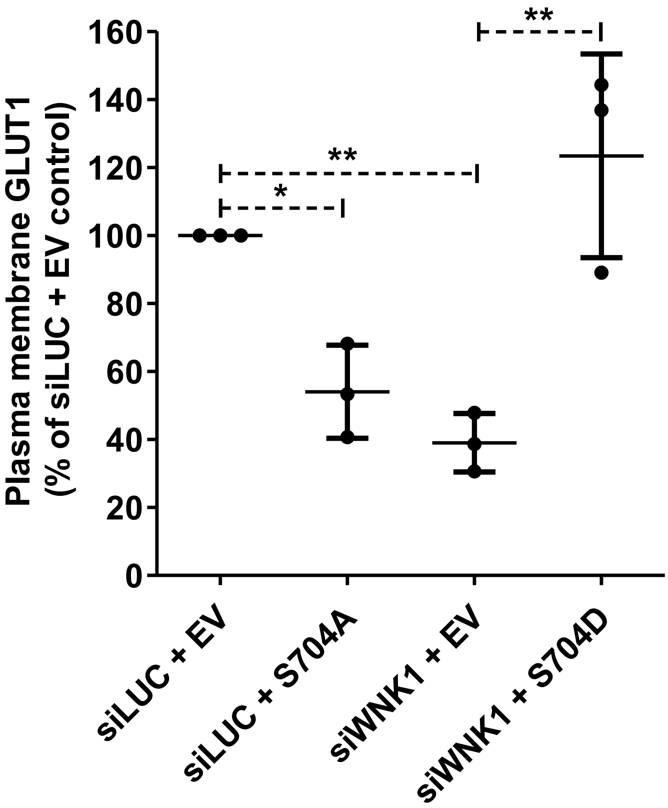

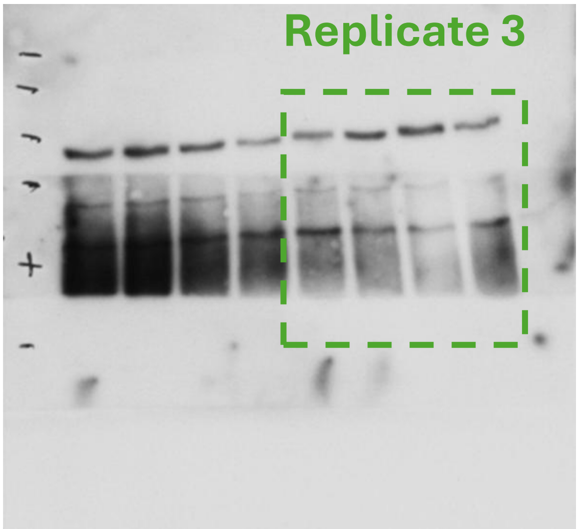

Supplement: Supplementary file 1 [file cells-15-01582-s001.zip › Supplementary Data S3_Original WB images_revised.pdf]
